# Comprehensive Computational Analysis of Honokiol Targets for Cell Cycle Inhibition and Immunotherapy in Metastatic Breast Cancer Stem Cells

**DOI:** 10.1155/2022/4172531

**Published:** 2022-07-08

**Authors:** Skolastika Skolastika, Naufa Hanif, Muthi Ikawati, Adam Hermawan

**Affiliations:** ^1^Laboratory of Macromolecular Engineering, Department of Pharmaceutical Chemistry, Faculty of Pharmacy, Universitas Gadjah Mada Sekip Utara II, 55281 Yogyakarta, Indonesia; ^2^Cancer Chemoprevention Research Center, Faculty of Pharmacy, Universitas Gadjah Mada Sekip Utara II, 55281 Yogyakarta, Indonesia

## Abstract

Breast cancer stem cells (BCSCs) play a critical role in chemoresistance, metastasis, and poor prognosis of breast cancer. BCSCs are mostly dormant, and therefore, activating them and modulating the cell cycle are important for successful therapy against BCSCs. The tumor microenvironment (TME) promotes BCSC survival and cancer progression, and targeting the TME can aid in successful immunotherapy. Honokiol (HNK), a bioactive polyphenol isolated from the bark and seed pods of *Magnolia spp*., is known to exert anticancer effects, such as inducing cell cycle arrest, inhibiting metastasis, and overcoming immunotherapy resistance in breast cancer cells. However, the molecular mechanisms of action of HNK in BCSCs, as well as its effects on the cell cycle, remain unclear. This study aimed to explore the potential targets and molecular mechanisms of HNK on metastatic BCSC (mBCSC)-cell cycle arrest and the impact of the TME. Using bioinformatics analyses, we predicted HNK protein targets from several databases and retrieved the genes differentially expressed in mBCSCs from the GEO database. The intersection between the differentially expressed genes (DEGs) and the HNK-targets was determined using a Venn diagram, and the results were analyzed using a protein-protein interaction network, hub gene selection, gene ontology and Kyoto Encyclopedia of Genes and Genomes pathway enrichment analyses, genetic alteration analysis, survival rate, and immune cell infiltration levels. Finally, the interaction between HNK and two HNK-targets regulating the cell cycle was analyzed using molecular docking analysis. The identified potential therapeutic targets of HNK (PTTH) included *CCND1*, *SIRT2*, *AURKB*, *VEGFA*, *HDAC1*, *CASP9*, *HSP90AA1*, and *HSP90AB1*, which can potentially inhibit the cell cycle of mBCSCs. Moreover, our results showed that PTTH could modulate the PI3K/Akt/mTOR and HIF1/NFkB/pathways. Overall, these findings highlight the potential of HNK as an immunotherapeutic agent for mBCSCs by modulating the tumor immune environment.

## 1. Introduction

Breast cancer was the most prevalent cancer in 2020 (in terms of new cases) and the leading cause of cancer-related deaths among females [1]. According to the World Health Organization, breast cancer has the highest incidence rate in Indonesia, with a mortality rate of 22,692 cases per year [1]. By 2040, the incidence is predicted to reach 89,512 cases [1]. Chemotherapy, along with surgery, radiation, and mastectomy, is the most common treatment [2]. Chemoresistance, or the insensitivity of cancer cells to drug therapy, is a major factor in the failure of chemotherapy against breast cancer.

Breast cancer stem cells (BCSCs) are one of the main factors driving chemoresistance, thereby contributing to poor prognosis and clinical outcomes [3–5]. BCSCs can develop into many cell types and repopulate heterogeneous tumors following conventional chemotherapy or radiotherapy [4, 6]. BCSCs are mostly dormant and therefore activating dormant cells, and modulating the cell cycle is important for achieving successful BCSCs therapy [7]. Recurrent tumors are highly aggressive, potentially cross-drug resistant, highly metastatic, and have a poor prognosis. A previous study demonstrated that immune cells such as CD8+ lymphocytes induce epithelial to mesenchymal transition of BCSCs [8] Moreover, the tumor microenvironment (TME) promotes BCSC survival and cancer progression [9], and hence it can prevent the success of immunotherapy [10] The use of combination therapy, in which both chemotherapy and natural compounds are used to target metastatic BCSCs (mBCSCs), could be a successful approach to overcome chemoresistance and achieve clinical success in treating breast cancer.

**Figure 1:**
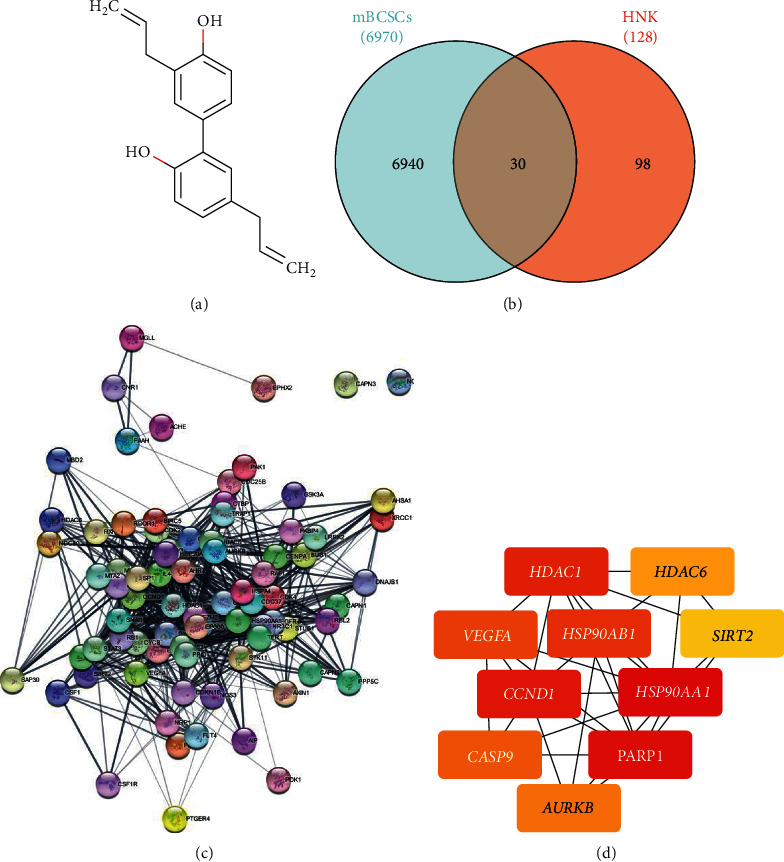
(a) Structure of honokiol. (b) Venn diagram of potential therapeutic targets of honokiol (PTTH) in breast cancer stem cells (BCSCs). (c) Protein-protein interaction (PPI) network of honokiol and the interacting proteins. (d) Top-10 hub genes determined according to the Maximal Clique Centrality (MCC) score.

Honokiol (HNK; 3,5-di-(2-propenyl)-1,1′-biphenyl-2,2′-diol, Figure 1(a)) is a bioactive polyphenol isolated from the bark and seed pods of *Magnolia spp*., that is widely used in traditional Asian medicine [11]. HNK controls various intracellular signaling pathways involved in cancer, including those related to nuclear factor kappa B (NF-*κ*B), signal transducers and activators of transcription 3 (STAT3), epidermal growth factor receptor (EGFR), and mammalian targets of rapamycin (mTOR) [12]. HNK-mediated cell cycle arrest is achieved via the downregulation of cyclin D1, an inhibition of cyclin E1, cyclin-dependent kinase 2, cyclin-dependent kinase 4, cMYC, and RB, CSK/EGFR signaling, and the upregulation of p27 and p21 [13, 14]. HNK has shown to inhibit matrix metalloproteinases, thereby reducing cell migration, invasion, and metastasis, while also regulating VEGFR signally, exerting an anti-angiogenic effect [15, 16]. In addition, HNK has been reported to successfully inhibit the pluripotency factors POU5F1, Nanog, and SOX2, and to abolish the BCSC-like phenotype [17–20] (p11). Moreover, HNK decreases drug resistance by inhibiting P-gp regulation and by enhancing apoptosis [21]. In addition, HNK also inhibits the PI3K/mTOR pathway, contributing to circumventing immunotherapeutic resistance in glioma and breast cancer cells [22]. Even though increasing research has evaluated the effects of HNK in the cell cycle, BCSCs, and metastasis, the molecular mechanisms underlying its effects on metastatic BCSC cell cycle axis and immunotherapy have not been elucidated.

This study aimed to explore the molecular mechanisms underlying HNK-mediated mBCSC-cell cycle arrest, as well as to assess the impact of this compound on the immune environment using bioinformatics studies.

## 2. Materials and Methods

### 2.1. Data Collection and Differentially Expressed Genes' (DEGs) Identification

Proteins that interact with HNK were searched using STITCH (https://stitch.embl.de), [23]. Swisstargetprediction (https://www.swisstargetprediction.ch), [24] canSAR Black (https://cansarblack.icr.ac.uk/) [25], and SEA (https://sea.bkslab.org/) [26]. The retrieved proteins were considered as HNK-mediated proteins (HMPs) and were included in the subsequent analyses. The microarray data of metastatic breast cancer stem cells were collected from the GEO database (https://www.ncbi.nlm.nih.gov/geo) using keywords such as metastatic breast cancer stem cells, and *Homo sapiens*. The inclusion criteria were: use of patient samples or patient-derived xenografts; focus on metastatic breast cancer; characterization of breast cancer stem cells; and clear description of the identity of samples in the GSE datasets. The exclusion criteria included: use of breast cancer cell lines; no emphasis on metastatic breast cancer; no characterization of breast cancer stem cells; and ambiguity around the identity of samples in the GSE datasets. One GSE Dataset (GSE151191) was selected among the 62 datasets for this study (Supplementary Figure 1). GEO2R, a web-based interactive program (https://www.ncbi.nlm.nih.gov/geo/geo2r) that compares two groups of samples under the same conditions, was used to identify the DEGs between primary and metastatic tumors, on the basis of the following criteria for significance: *P* < 0.05 and log |Fold Change| > 1. Using [27], the overlapping proteins between the HMP and those encoded by the DEGs were identified and further analyzed using a protein-protein interaction (PPI) network.

### 2.2. Construction of the PPI Network

The PPI was constructed and displayed using STRING-DB v11.0 and Cytoscape software, respectively [28, 29]. Proteins included in the top-10 rank according to the Maximal Clique Centrality (MCC) score determined by the Cyto-Hubba plugin were considered hub genes [30].

The hub genes were subjected to GO and KEGG enrichment analyses using the tools [31] and WebGestalt [32]. Statistical significance was set at *P* < 0.05.

### 2.3. Genetic Alterations Analysis

The proteins nominated by GO and KEGG enrichment analyses and an in-depth literature study on the hub genes were used to determine the potential therapeutic targets of HNK (PTTH). In this study, genes encoding PTTH such as *CCND1*, *SIRT2*, *AURKB*, *VEGFA*, *HDAC1*, *CASP9*, *HSP90AA1*, and *HSP90AB1* were screened for genetic changes in all breast cancer studies available in the cBio-portal database ((https://www.cbioportal.org) [33]. The studies with the highest number of genetic changes were selected for analysis further connectivity.

### 2.4. Survival Rate and Immune Cell Infiltration Level

The online database Gene Expression Profiling Interactive Analysis (GEPIA, https://gepia.cancer-pku.cn) was utilized to analyze the contribution of PTTH to the overall survival (OS) [34]. Tumor Immune Estimation Resource (TIMER) (https://cistrome.shinyapps.io/timer/) was used to analyze the correlation between PTTH expression levels and immune cell infiltration level [35].

### 2.5. Validation of the mRNA and Protein Expression Levels of PTTH

The mRNA and protein expression levels of PTTH were determined using TNMPlot and Human Protein Atlas (HPA). Differentially expressed genes and mRNA levels in tumor, normal, and metastatic tissues were analyzed using TNMplot (https://www.tnmplot.com/) [36], in which the database utilized data from GEO or RNA-seq libraries from The Cancer Genome Atlas (TCGA), Therapeutically Applicable Research to Generate Effective Treatments (TARGET), and The Genotype-Tissue Expression (GTEx). The protein expression levels were analyzed using the Human Protein Atlas (HPA) (https://www.proteinatlas.org/) [37], an online database that contains a wide range of transcriptomic and proteomic data from various tissues and cells.

### 2.6. Molecular Docking

To predict the binding properties of HNK to AURKB and RAC-1 through molecular docking, computational prediction was conducted on a Windows 10 operating system, Intel Core (TM) i5-10th Gen with 8 GB of RAM. MOE 2010 (licensed from Faculty of Pharmacy UGM) was used for docking simulation, RMSD-docking score calculation, and visualization interaction. The PDB IDs of the proteins AURKB and RAC-1 (3ZCW and 3TH5, respectively) were searched for in https://rcsb.org. The HNK structure was obtained from PubChem, subjected to conformational search, and minimized in the MOE using Energy Minimize Menu. For the docking simulation setting, London dG was used for both Rescoring 1 and Rescoring 2. Triangle Matcher was used for score function and placement setting, and Forcefield was used to refine the docking results from 30 retained settings. The results of this method will determine which conformation has the lowest binding interaction between the ligand and its receptor.

## 3. Results

### 3.1. DEG and HMP Identification

The DEGs are considered to be the molecular drivers and/or molecular biomarkers of various phenotypes [38]. The identification of DEGs was carried out to determine the genes that act as biomolecular markers of metastatic breast cancer stem cells (mBCSCs). In total, 6,970 DEGs in the GSE 151191 dataset were found to be up/downregulated in metastatic breast cancer stem cells, according to the adjusted *P* value of <0.05, and a |logFC| ≥ 1.0 (Supplementary Table 1). Subsequently, proteins that interact directly and/or indirectly with HNK, referred to as HMPs, were identified. A total of 128 HMPs were retrieved from Swisstargetprediction, STITCH, canSAR Black, and SEA (Supplementary Table 2). Finally, 30 overlapping genes (OGs), including 18 upregulated and 12 downregulated genes, were identified to be both HMP and DEGs (Figure 1(b); Table 1).

### 3.2. PPI Network

To deepen our understanding of the interactions between the 30 OGs, we constructed a PPI network. The network contained 40 nodes and 134 edges, with an average node degree of 6.7, an average local clustering coefficient of 0.618, and a PPI enrichment value < 1.0e-16 (Figure 1(c)). Further analysis identified hub genes within the PPI network (Figure 1(d)), which included NAD-dependent deacetylase sirtuin 2 (SIRT2), cyclin D1 (CCND1), serine/threonine-protein kinase Aurora-B (AURKB), vascular endothelial growth factor A (VEGFA), histone deacetylase 1 (HDAC1), caspase 9 (CASP9), heat shock protein HSP 90-alpha (HSP90AA1), and heat shock protein HSP 90-beta (HSP90AB1) (Table 2).

### 3.3. GO and KEGG Pathway Enrichment Analysis

**Figure 2:**
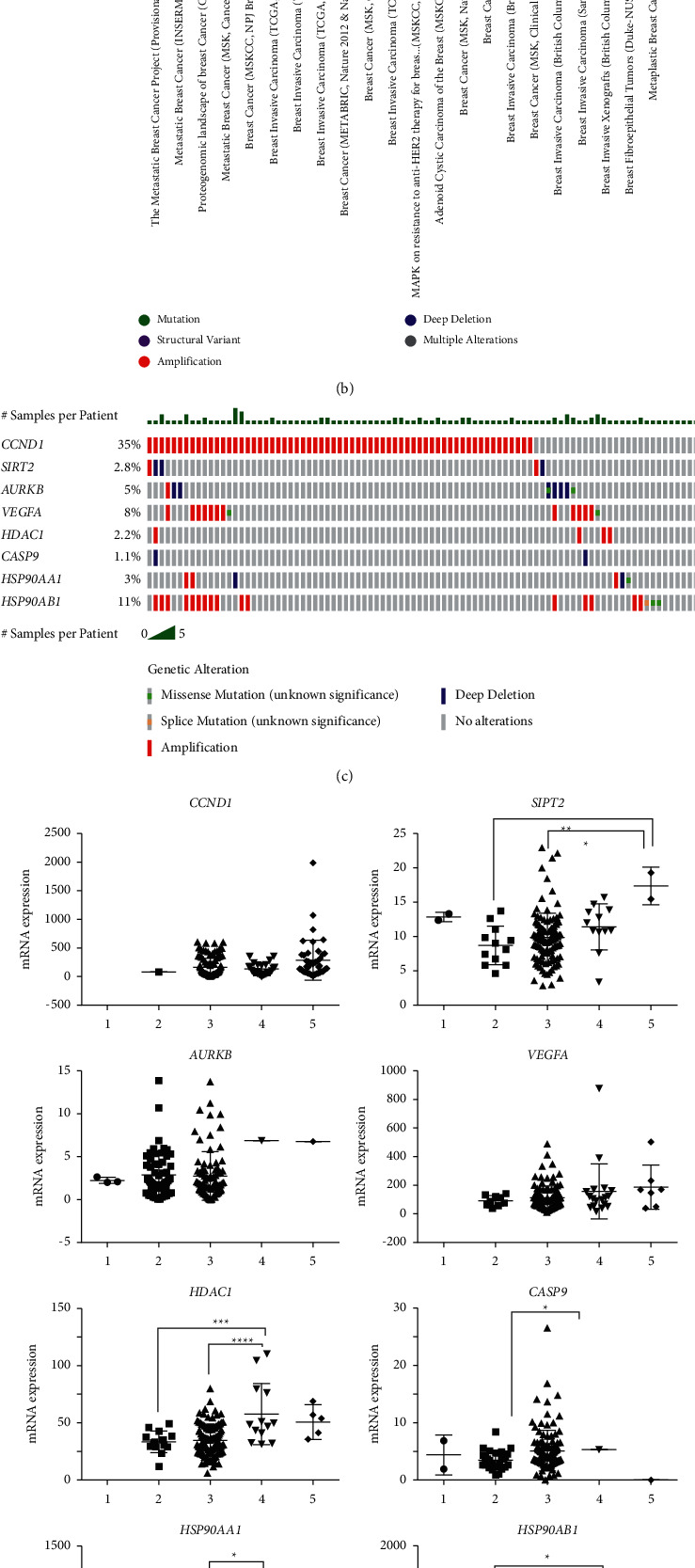
(a) Gene ontology (GO) analysis of potential therapeutic targets of honokiol (PTTH) results using WebGestalt. (b) Overview of genetic alterations in *CCND1, SIRT2, AURKB, VEGFA, HDAC1, CASP9, HSP90AA1*, and *HSP90AB1* based in samples from several breast cancer studies. (c) Oncoprint analysis of *CCND1, SIRT2, AURKB, VEGFA, HDAC1, CASP9, HSP90AA1*, and HSP90AB1 on The Metastatic Breast Cancer Project (Provisional, February 2020) dataset. (d) mRNA expression levels of *CCND1, SIRT2, AURKB, VEGFA, HDAC1, CASP9, HSP90AA1*, and *HSP90AB1* on the Metastatic Breast Cancer Project (Provisional, February 2020) as analyzed using cBioportal. 1: deep deletion, 2: shallow deletion, 3: diploid; 4: gain; 5: amplification. Statistical analyses were done by one-way ANOVA using Tukey's multiple comparison test. The symbol ^*∗*^or^*∗∗*^or^*∗∗∗*^or^*∗∗∗∗*^ symbolizes *P* < 0.05 or *P* < 0.01 or *P* < 0.001 or *P* < 0.001, respectively. (e) Pathways related to genetic alterations predicted by cBioportal. The results showed that genetic alterations of the PTTH disrupted the pathways regulating the cell cycle.

The functions of the OGs were further investigated using GO and KEGG pathway enrichment analyses. The biological processes in which these OGs were implicated are summarized in Figure 2(a). Among the identified biological processes, cell communication, metabolic process, cellular component organization, multicellular organismal process, developmental process, response to stimulus, and biological regulation are strongly linked to cancer progression. According to the enrichment analysis of cellular components, the OGs were abundant in the nucleus, cytosol, membrane-enclosed lumen, and protein-containing complex (Figure 2(a)). Finally, the OGs were enriched in the molecular function protein binding (Figure 2(a)). KEGG pathway enrichment analysis demonstrated that the OGs were particularly enriched in the PI3K-Akt signaling pathway, pathways associated with cancer, and the regulation of the cell cycle (Supplementary Table 3).

### 3.4. Genetics Alteration Analysis

Eight genes (*SIRT2*, *CCND1*, *AURKB*, *VEGFA*, *HDAC1*, *CASP9*, *HSP90AA1*, and *HSP90AB1*) that play an essential role in the growth and development of mBCSC were selected from hub genes and referred to as potential therapeutic targets of honokiol (PTTH). Genetic variation within these genes was analyzed using cBioportal. The breast cancer study with the highest number of genetic changes was selected for further analysis (Figure 2(b)). Oncoprint was used to determine the percentage of PTTH gene alterations in patients with mBC. Genetic alterations in PTTH ranged from 1.1% to 35% in the 180 patient samples analyzed (Figure 2(c)), with amplification being the most common gene alteration. The genes that were most frequently mutated were *CCND1* (35%), *HSP90AB1* (11%), and *VEGFA* (8%). Mutual exclusivity analysis showed that *VEGFA* mutations significantly co-occurred with *HSP90AB1* mutations (Table 3). Copy number alterations (CNAs) are particularly common in cancer and play a significant role in its development and progression. CNA status can be homozygously deleted (shallow deletion), heterozygously deleted (deep deletion), diploid, gained (amplification event with relatively few copies), or amplified (amplification event with many copies). CNAs analysis revealed that *SIRT2* mRNA expression was lower in shallow deletion cases and higher in amplification cases than in diploids (normal/without change) (Figure 2(d)). *HDAC1* and *HSP90AB1* mRNA expression was lower in cases with shallow deletions and higher in cases with gain. *HSP90AA1* mRNA expression was lower in patients with gain than in those with diploid gain. *CASP9* mRNA expression was lower in the gain than in the in shallow deletion cases, but not significantly different from that in diploid cases. All CNAs other than those mentioned were not differently expressed. Finally, the cBioportal pathway analysis showed that the cell cycle pathway is the main pathway that is disrupted by PTTH genetic alterations. Among the genes involved in the regulation of cell pathway, *CCND1*, encoding cyclin D1, was identified as PTTH (Figure 2(e)).

### 3.5. Survival Rate and Immune Cell Infiltration Level

**Figure 3:**
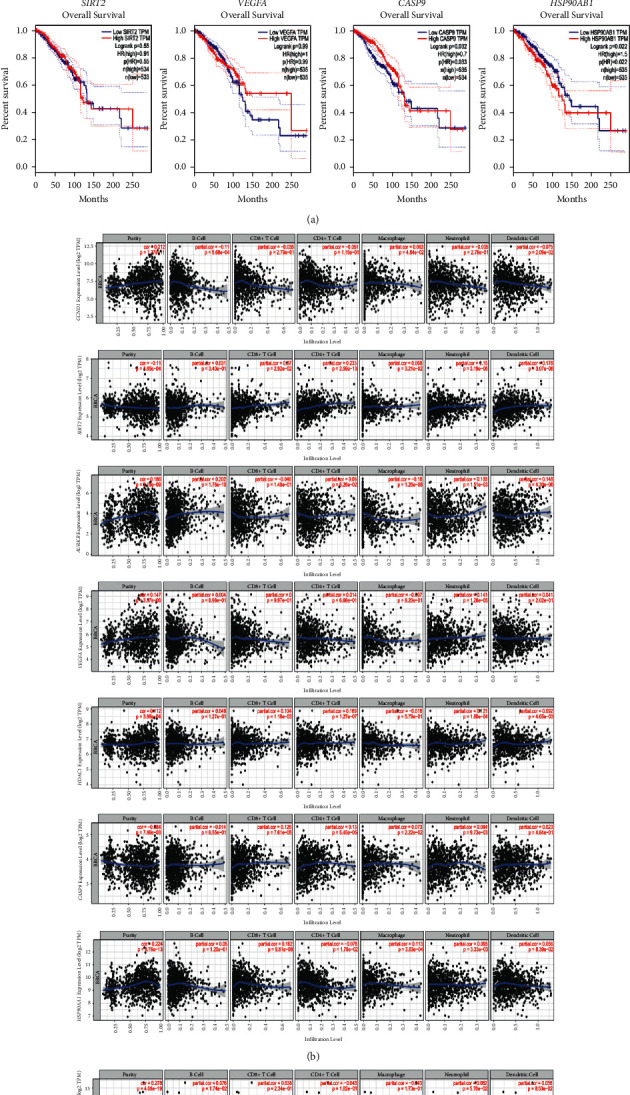
(a) Association between the expression levels of *CCND1, SIRT2, AURKB, VEGFA, HDAC1, CASP9,HSP90AA1*, and *HSP90AB1* and the overall survival in the breast cancer samples from The Cancer Genome Atlas. (b) Correlation analysis between the expression levels of *CCND1, SIRT2, AURKB, VEGFA, HDAC1, CASP9, HSP90AA1*, and *HSP90AB1* and the infiltration levels of B cells, CD8+ T cells, CD4+ T cells, macrophages, neutrophils, and dendritic cells.

To assess the clinical value of PTTH genes' expression levels, we examined whether they are associated with the OS or prognosis of patients with breast cancer. Low expression levels of *CASP9* and *HSP90AB1* were significantly associated with poor OS (*P* < 0.05) (Figure 3(a)). To understand the role of the immune microenvironment in the development and prognosis of patients with BRCA mutations, we analyzed the correlation between the expression levels of PTTH and immunocyte infiltration. The expression level of PTTH was either positively or negatively related to the infiltration level of different immune cells, indicating that PTTH modulated the immunologic microenvironment by influencing immune cell infiltration. The expression levels of *CCND1*, *VEGFA*, *AURKB*, *HDAC1*, *HSP90AA1*, and *HSP90AB1* were positively correlated with immune cell infiltration levels, whereas the expression levels of *SIRT2* and *CASP9* were negatively correlated with the tumor purity of BRCA (Figure 3(b)). Additionally, the B cells' infiltration level was positively correlated with the expression levels of *HSP90AB1* and *AURKB*, and negatively correlated with the expression level of *CCND1*. Moreover, we observed a positive correlation between CD8+ T cells' infiltration levels and *SIRT2*, *HDAC1*, *CASP9*, and *HSP90AA1* expression levels, and between CD4+ T cells' infiltration levels and *SIRT2*, *HDAC1*, and *CASP9* expression levels. However, CD4+ T cells' infiltration levels were negatively correlated with *HSP90AA1* expression levels. Infiltration levels of macrophages were positively correlated with *CCND1*, *SIRT2*, *CASP9*, and *HSP90AA1* expression levels, whereas they were negatively correlated with *AURKB* expression levels. Neutrophil infiltration levels were positively correlated with *SIRT2*, *AURKB*, *VEGFA*, *HDAC1*, *CASP9*, *HSP90AA1*, and *HSP90AB1*. Dendritic cell infiltration levels were negatively correlated with *CCND1* expression levels and positively correlated with *SIRT2*, *AURKB*, and *HDAC1* expression levels. Other non-mentioned data are not statistically significant.

### 3.6. Validation of the mRNA Expression Level and the Protein Expression Level of PTTH

**Figure 4:**
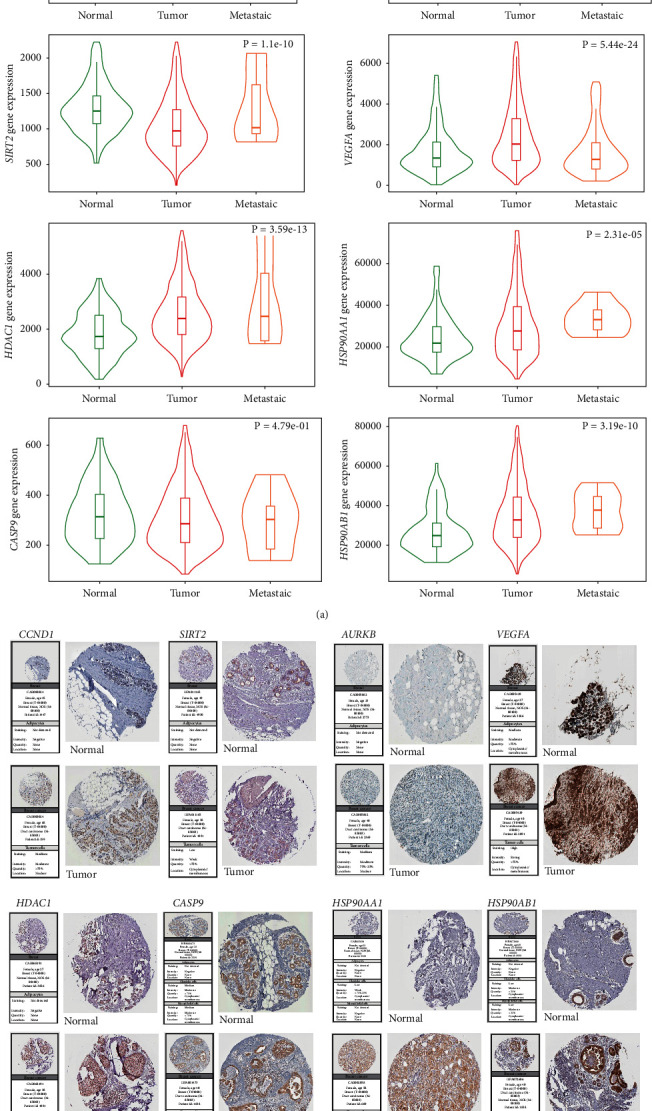
mRNA and the protein expression levels of *CCND1, SIRT2, AURKB, VEGFA, HDAC1, CASP9,HSP90AA1*, and *HSP90AB1*. (a) mRNA expression levels in the Cancer Genome Atlas (TCGA). (b) Protein expression levels in normal and tumor breast tissues retrieved from the Human Protein Atlas (HPA).

The expression levels of *CCND1*, *AURKB*, *HDAC1*, *VEGFA*, *HSP90AA1*, and *HSP90AB1* were increased in the BC tissue, and even higher in mBC (Figure 4(a)). *CASP9* expression levels were not significantly different between normal, tumor, and metastatic breast cancer tissues. Interestingly, *SIRT2* expression was decreased in breast cancer tissues, but increased in metastatic tissues compared to normal tissues. These results were supported by immunohistochemical data from HPA, that showed that CCND1, AURKB, and HDAC1 were overexpressed in the nucleus, while SIRT2, VEGFA, HSP90AA1, and HSP90AB1 were overexpressed in the cytoplasmic/membranous region (Figure 4(b)). Finally, CASP9 was not differentially expressed between the normal tissue and the tumor tissue.

### 3.7. Molecular Docking

**Figure 5:**
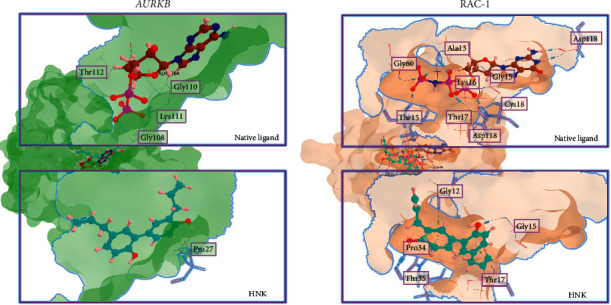
Visualization of molecular docking of honokiol (HNK) toward AURKB and RAC-1.

Molecular docking analysis revealed that AURKB and RAC-1 could bind to their respective native ligands and to HNK (Figure 5). The affinity of the interaction between these proteins and HNK was similar to that of their natural ligands. The interaction between AURKB and its native ligand ADP was stronger than that between *AURKB* and HNK according to the docking score (−12.89 and−8.68, respectively, Table 4). Furthermore, ADP interacted with several amino acids of AURKB, such as Gly108, Gly110, Lys111, and Thr112, while HNK interacted with only one amino acid (Pro27). Likewise, the binding interaction between Rac-1 and its native ligand (phosphoaminophosphonic acid-guanylate ester/GNP; docking score −21.38) was stronger than that between Rac-1 and HNK (docking score −18.17). This was a result of the number of amino acids that the compounds interacted with and the distance between the interacting amino acids and the compound. For instance, the distance between Thr17 and GNP was much closer (1.82 Å) than that with HNK (3.35). However, despite the lower affinity of the interaction, HNK could potentially compete with the native ligands to inhibit the function of these proteins.

## 4. Discussion

This study identified eight PTTHs, including *CCND1*, *SIRT2*, *AURKB*, *VEGFA*, *HDAC1*, *CASP9*, *HSP90AA1*, and *HSP90AB1*. The expression levels of these genes were strongly associated with immune infiltration levels. The tumor microenvironment influences angiogenesis and the immune response, and has long been recognized as a primary determinant of long-term tumor progression [39–41]. In addition, it can greatly impact the effectiveness of immunotherapy, highlighting the need of its further understanding [42].

Cyclin D1, encoded by *CCND1*, is considered an oncogene that promotes cell proliferation, growth, angiogenesis, and resistance to chemotherapy and radiotherapy [43, 44]. In this study, we revealed that *CCND1* expression was positively correlated with BRCA purity and macrophage infiltration levels, and negatively correlated with B cell and dendritic cell infiltration levels. Many studies have shown that tumor-associated macrophages play an important role in the proliferation, invasion, angiogenesis, and metastasis of human breast carcinoma, and that increased macrophage tumor infiltration confers metastatic potential and is associated with poor prognosis in breast cancer [42]. Our findings are in line with those of Pestell et al., who demonstrated that cyclin D1 expression was increased in human cancer stroma, and promoted tumor inflammation, angiogenesis, and stem cell expansion in advanced breast cancer [41]. Interestingly, a previous study reported that HNK could inhibit cyclin D1 expression [14].

SIRT2, an NAD-dependent histone deacetylase, has been suggested to be a promising therapeutic target in cancer treatment [47]. *SIRT2* is thought to affect carcinogenesis in a context-dependent manner, affecting epigenetic pathways implicated in cancer initiation, development, and progression [48–50]. *SIRT2* expression level was negatively correlated with BRCA purity, but positively correlated with CD8+ T cell, CD4+ T cell, macrophages, neutrophils, and dendritic cells' infiltration levels. These results confirm those of previous studies, in which *SIRT2* expression level and CD8+ T cell infiltration level were positively correlated in breast cancer patients [51]. In addition, systemic SIRT2 has been suggested to promote tumor development by suppressing NK cells [42]. Interestingly, *SIRT2* expression was significantly lower in breast cancer than in normal breast tissue, suggesting that *SIRT2* may act as a tumor suppressor during the initiation of tumorigenesis. Moreover, a previous study reported that high *SIRT2* expression in advanced tumor tissues is associated with poor prognosis, suggesting that *SIRT2* may function as an oncogene [50].

VEGFA is a cytokine that promotes vascular development and the formation of new blood vessels from pre-existing vascular networks during embryogenesis [52–54]. In addition, VEGFA can also be released by cancer and stromal cells [55]. In several murine and human cancer models, it has been demonstrated that VEGFA stimulates the tumor-initiating epithelial–mesenchymal transition and metastasis, and that *VEGFA* expression levels are positively correlated with BRAC purity and neutrophil infiltration levels [56–63]. In line with these results, another study reported that patients with mBC had higher levels of circulating VEGFA than patients without metastases [64]. VEGF can stimulate neutrophil migration through the activation of VEGFR1, [65] and can prevent dendritic cells from maturing, resulting in cytotoxic T cells' inactivation [66]. Tregs, tumor-associated macrophages, and myeloid-derived suppressor cells are all highly induced by VEGF, resulting in an immunosuppressive TME [67]. Furthermore, VEGF increases the expression of PD-1 on CD8+ CTLs and Tregs in a VEGFR2-dependent manner, [64] as well as the expression of Fas ligand, interleukin (IL)-10, and prostaglandin E3, leading to cytotoxic T cells' depletion [65]. Hence, VEGF-A can be used as a biomarker for immune-targeting therapy in breast cancer patients [66].

Histone deacetylase 1 (HDAC1) is overexpressed in breast cancer cells and human breast cancer tissues and can trigger the proliferation and migration of these cells via activation of Snail/IL-8 signals [41]. *HDAC1* suppression has been reported to reduce the invasion of breast cancer cells by inhibiting matrix metalloproteinase-9, [67] and to reduce PD-L1 and HLA-DR expression and Treg frequency in triple negative breast cancer [68]. In our study, there was a positive correlation between *HDAC1* expression levels and tumor purity, and CD8+ T cells', CD4+ T cells', neutrophils', and dendritic cells' infilitration levels.

HSP90*α*, encoded by *HSP90AA1*, is the stress-inducible isoform of HSP90. Previous studies have shown that high expression levels of HSP90 (HSP90*α* and HSP90*β*) increase the likelihood of recurrence and distant metastases in triple negative and ER+/HER2-breast cancer, and are associated with higher mortality [69]. Overexpression of HSP90 in human breast cancer cells has been linked to enhanced cell proliferation [42] and metastasis, [70] as well as to short OS and aggressive clinicopathological characteristics, such as high clinical stage, large tumors, and lymph node involvement [71]. Lin et al. reported that elevated *HSP90AB1* expression was linked to a better overall survival of ER- and Basal-like breast cancer patients [55]. However, we found that high *HSP90AB1* expression was associated with poor prognosis in BRCA patients. Additionally, the expression of *HSP90AA1* and *HSP90AB1* was positively correlated with tumor purity; and the expression of *HSP90AA1* was positively related to CD8+ T cells, macrophages, and neutrophils, but negatively correlated with CD4+ T cells. Further studies for exploring the infiltration of CD8+, macrophages, neutrophils, CD4+, and the effects of HNK on *HSP90AA1* and *HSP90AB1* are warranted.

*CASP9* encodes caspase-9, an initiator of the intrinsic apoptosis pathway [79]. When the apoptosome, a multimolecular complex comprising cytochrome c and the apoptotic peptidase activating factor 1 (Apaf-1), is formed, it cleaves pro-caspase-9, forming caspase-9, triggering the caspase activation cascade by activating executor caspases, including caspase 3 and caspase 7 to cleave other cellular targets [80].

The aurora kinase family includes Aurora kinase B (AURKB), a mitotic serine/threonine protein kinase, and aurora kinase A (AURKA), which is a member of the Chromosomal Passenger Complex (CPC). The CPC plays a role in cell cycle progression and is a prognostic marker of breast cancer arising from *BRCA2* mutation [81]. Deregulation of AURKB is observed in several tumors, and its overexpression is frequently linked to tumor cell invasion, metastasis, and drug resistance [82]. Hence, AURKB has emerged as an attractive drug target for the development of small-molecule inhibitors [83].

RAC1 and AURKB were selected for in-depth molecular docking analysis because they were connected to the PTTH-mBCSCs-cell cycle axis. The Rho-GTPase family, including Rho, Rac1, and Cdc42, regulates the cytoskeleton [84] and thus modulates cell motility, migration, and invasion [85]. Rac-1/Cdc42 activation can induce cell growth by activating the PAK1/cyclin D1 pathway, or cell death by activating the PAK1/Akt/BAD pathway [86]. Rho has been suggested to be a potential therapeutic target, since Rho and *VEGFA* crosstalk leads to cancer progression and metastasis [87].

**Figure 6:**
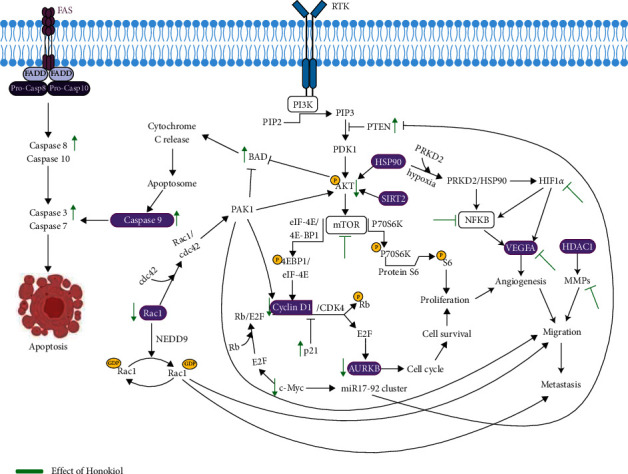
Proposed mechanism of honokiol (HNK) in mBCSCs (created with https://BioRender.com).

This study revealed the potential targets and molecular mechanisms of HNK on the cell cycle of mBCSCs (Figure 6). It is known that HNK exerts anticancer effects by suppressing angiogenesis, migration, invasion, and proliferation in a variety of cancer cell lines and tumor models [12]. HNK inhibits the cell cycle via the PI3K/Akt/mTOR pathway by upregulating PTEN and P21, and suppressing p-Akt, cyclin D/CDK4, c-Myc, Rac1, and *AURKB* [13, 14, 22, 71]. Angiogenesis is inhibited through the HIF1/NFkB pathway, which is activated under hypoxic conditions and blocks the release of VEGF. Immune infiltration analysis showed that HNK is correlated with *VEGFA* inhibition, suggesting HNK can effectively block VEGFR2. HNK was also found to reduce HIF-induced VEGFR/VEGF activation and inhibit matrix metalloproteinases activity and cell migration [88]. In addition, HNK can induce apoptosis through the upregulation of BAD, caspase-9, caspase-3, and caspase-8 [89]. In the tumor microenvironment, oncogenic drivers such as *β*-catenin, STAT3, PI3K/PTEN/AKT/mTOR, p53, NF-kB, and RAS/RAF/MAPK are activated to suppress the production of chemokines, reduce the recruitment of dendritic cells, macrophages, T cells, and NK cells to tumor sites, and to suppress the immune system of these immunocytes [90]. Furthermore, tumor-intrinsic signaling can cause tumor cells to express PD-L1, resulting in T cell dysfunction in the tumor microenvironment. This study highlights the potential of HNK as an immunotherapeutic agent for mBCSCs by modulating the tumor immune environment. However, the results of this study were obtained through bioinformatics studies; therefore, further in vitro, in vivo, and clinical trials are needed to validate the findings.

## 5. Conclusions

This study identified eight PTTHs consisting of *CCND1*, *SIRT2*, *AURKB*, *VEGFA*, *HDAC1*, *CASP9*, *HSP90AA1*, and *HSP90AB1*, which can inhibit the mBCSC-cell cycle axis. In addition, PTTHs may regulate the PI3K/Akt/mTOR and HIF1/NFkB pathways. This study is speeding up the development of HNK as anti-mBCSCs by targeting certain genes. However, this study have several limitations; for example, the targets of HNK are predicted from database. Other additional machine learning algorithm will provide more validated candidates of HNK targets. Another limitation of this study is that we used a bioinformatics approach; therefore, more needs to be explored further for validation and clarification in laboratory experiments.

## Figures and Tables

**Table 1:** Potential therapeutic targets of honokiol (PTTH) in metastatic breast cancer stem cells (mBCSCs).

No.	Protein symbol	Protein name	Database
1	*CASP9*	Caspase 9	STITCH
2	IL4	Interleukin 4	STITCH
3	PDK1	Pyruvate dehydrogenase kinase isoform 1	Swisstargetprediction
4	*CCND1*	Cyclin D1	STITCH
5	CSF1R	Macrophage colony stimulating factor receptor	Swisstargetprediction
6	TRAP1	Heat shock protein 75 kDa, mitochondrial	Swisstargetprediction
7	VEGFA	Vascular endothelial growth factor A	STITCH
8	HDAC8	Histone deacetylase 8	Swisstargetprediction
9	CAPN1	Calpain 1	STITCH
10	PARP1	Poly (ADP-ribose) polymerase-1	Swisstargetprediction
11	MGLL	Monoglyceride lipase	Swisstargetprediction
12	NQO2	Quinone reductase 2	Swisstargetprediction
13	RXRA	Retinoid X receptor alpha	canSAR Black
14	CNR1	Cannabinoid receptor 1	Swisstargetprediction
15	*HSP90AB1*	Heat shock protein HSP 90-beta	Swisstargetprediction
16	AURKB	Serine/threonine-protein kinase Aurora-B	Swisstargetprediction
17	PTGER4	Prostanoid EP4 receptor	Swisstargetprediction
18	*HSP90AA1*	Heat shock protein HSP 90-alpha	Swisstargetprediction
19	DYRK1A	Dual-specificity tyrosine-phosphorylation regulated kinase 1A	Swisstargetprediction
20	GSK3A	Glycogen synthase kinase-3 alpha	Swisstargetprediction
21	ACHE	Acetylcholinesterase	Swisstargetprediction
22	PAK1	Serine/threonine-protein kinase PAK 1	Swisstargetprediction
23	FAAH	Anandamide amidohydrolase	Swisstargetprediction
24	CAPN2	Calpain 2	STITCH
25	CAPN3	Calpain 3	STITCH
26	*HDAC1*	Histone deacetylase 1	Swisstargetprediction
27	EPHX2	Epoxide hydratase	Swisstargetprediction
28	CDC25B	Dual specificity phosphatase Cdc25B	Swisstargetprediction
29	HDAC6	Histone deacetylase 6	Swisstargetprediction
30	SIRT2	NAD-dependent deacetylase sirtuin 2	Swisstargetprediction

**Table 2:** Top-10 hub genes by Maximal Clique Centrality (MCC) score, as analyzed by CytoHubba.

Rank	Gene symbol	MCC score
1	HSP90AA1	132
2	PARP1	102
3	*CCND1*	98
4	HDAC1	67
5	HSP90AB1	62
6	VEGFA	53
7	CASP9	28
8	AURKB	26
9	HDAC6	24
10	SIRT2	18

**Table 3:** Mutual exclusivity analysis' results of the potential therapeutic targets of honokiol (PTTH).

A	B	Log2 odds ratio	*P* value	Tendency
*HSP90AB1*	*VEGFA*	>3	<0.001	Co-occurrence

**Table 4:** Molecular docking results of honokiol (HNK) toward AURKB and RAC-1.

Protein, PDB ID	Native ligand							Honokiol				
	S	RMSD (Å)	LA	AA	BT	D	S	RMSD (Å)	LA	AA	BT	D
AURKB, PDB ID : 3ZCW	**−12.89**	1.39	O O O O	Gly108 Gly110 Lys111 Thr112	ScD ScD ScD ScD	1.95 1.91 2.11 2.24	−8.68	1.10	C	Pro27	ArH	2.75
RAC-1, PDB ID : 3TH5	**−21.38**	0.48	O O O O O O O O H H	Ala13 **Gly15** Lys16 Lys16 **Thr17** Cys18 **Thr35** Gly60 Asp118 Asp118	ScD ScD ScD ScD ScD ScD ScD ScD ScD ScD	2.20 2.49 1.73 1.95 1.90 1.79 1.82 2.37 2.09 2.13	−18.17	1.86	C O C C O C	Gly12 **Gly15** **Thr17** Pro34 **Thr35** **Thr35**	ArH ScD ArH ArH ScA ArH	4.01 1.83 3.35 3.96 2.14 2.70

S: docking score, RMSD: root mean square deviation, LA: ligand atom, AA: amino acid, BT: binding type, D: distance, ScD: sidechain donor, ScA: sidechain acceptor, ArH: arene H, and BbD: backbone donor.

## Data Availability

The data generated or analyzed during this study are included within the article and its supplementary files.

## Supplementary Materials

Supplementary Materials
*Supplementary Table 1*. Differentially expressed genes (DEGs) in metastatic breast cancer stem cells (mBCSCs) from the GSE 151191 dataset. *Supplementary Table 2*. Honokiol (HNK)-mediated proteins (HMPs), as retrieved from Swiss target prediction, STITCH, canSAR Black, and SEA. *Supplementary Table 3*. KEGG pathway enrichment analysis of OGs from both HMP and DEGs. *Supplementary Figure 1*. Flowchart for the screening of datasets.
