# Supplementary material for: Comprehensive Computational Analysis of Honokiol Targets for Cell Cycle Inhibition and Immunotherapy in Metastatic Breast Cancer Stem Cells
Source: Evid Based Complement Alternat Med. 2022 Jul 8;2022:4172531. doi: 10.1155/2022/4172531 (PMC9286982; doi:10.1155/2022/4172531)

Supplementary Table 1. Differentially expresses genes (DEGs) in metastatic breast cancer stem cells (mBCSCs) from the GSE 151191 dataset.

ID	adj.P.Val	P.Value	logFC	GENE_SYMB	GENE_NAME	Remarks
A_33_P324	0.000483	1.16E-06	8.5	TAF5L	TAF5-like RNA polymerase II, p300/CBP-associated factor (PCAF)-asso	Upregulated
A_33_P324	0.000483	1.12E-06	8.08	BEX2	brain expressed X-linked 2	Upregulated
A_33_P347	0.000483	4.55E-07	7.9	ZBTB8A	zinc finger and BTB domain containing 8A	Upregulated
A_33_P367	0.000483	9.26E-07	7.62	LOC283887	uncharacterized LOC283887	Upregulated
A_21_P000	0.000483	4.25E-07	7.35	SNORD12	small nucleolar RNA, C/D box 12	Upregulated
A_33_P320	0.000483	3.31E-07	7.24	ZNF623	zinc finger protein 623	Upregulated
A_23_P413	0.000888	0.00006	7.23	ZNF330	zinc finger protein 330	Upregulated
A_23_P422	0.000483	1.83E-06	7.07	IER3	immediate early response 3	Upregulated
A_33_P323	0.000707	0.0000344	6.92	MT1G	metallothionein 1G	Upregulated
A_24_P254	0.000483	1.42E-06	6.87	ARHGEF9	Cdc42 guanine nucleotide exchange factor (GEF) 9	Upregulated
A_23_P303	0.000483	1.73E-06	6.85	P4HA2	prolyl 4-hydroxylase, alpha polypeptide II	Upregulated
A_21_P001	0.000483	1.51E-06	6.8	STK32C	serine/threonine kinase 32C	Upregulated
A_23_P126	0.000483	4.73E-07	6.79	CTH	cystathionine gamma-lyase	Upregulated
A_22_P000	0.000483	2.12E-06	6.76	lnc-RP11-116	lnc-RP11-116D17.1.1-10:1	Upregulated
A_24_P360	0.000483	6.44E-07	6.74	PDE7A	phosphodiesterase 7A	Upregulated
A_23_P273	0.000483	2.23E-06	6.67	KDSR	3-ketodihydrosphingosine reductase	Upregulated
A_23_P117	0.000483	1.11E-06	6.65	LYSMD4	LysM, putative peptidoglycan-binding, domain containing 4	Upregulated
A_22_P000	0.001049	0.0000884	6.61	lnc-MTMR9-1	lnc-MTMR9-1:1	Upregulated
A_23_P424	0.000483	4.87E-07	6.57	ERCC6L2	excision repair cross-complementation group 6-like 2	Upregulated
A_23_P894	0.000483	1.24E-06	6.54	CCL2	chemokine (C-C motif) ligand 2	Upregulated
A_24_P701	0.000483	4.43E-07	6.52	DTWD1	DTW domain containing 1	Upregulated
A_22_P000	0.000483	5.57E-07	6.52	LOC1005075	uncharacterized LOC100507564	Upregulated
A_23_P991	0.000483	2.47E-06	6.48	DRAM1	DNA-damage regulated autophagy modulator 1	Upregulated
A_33_P340	0.000483	2.54E-06	6.47	PNKP	polynucleotide kinase 3'-phosphatase	Upregulated
A_33_P335	0.000614	0.0000235	6.47	TRIM31	tripartite motif containing 31	Upregulated
A_22_P000	0.000483	3.03E-06	6.45	PIGL	phosphatidylinositol glycan anchor biosynthesis, class L	Upregulated
A_24_P778	0.000483	4.76E-07	6.4	RSL1D1	ribosomal L1 domain containing 1	Upregulated
A_24_P252	0.000483	1.28E-06	6.39	CXCR5	chemokine (C-X-C motif) receptor 5	Upregulated
A_23_P212	0.000534	0.0000136	6.38	HTT	huntingtin	Upregulated
A_33_P323	0.000837	0.0000526	6.38	ZNF100	zinc finger protein 100	Upregulated
A_21_P001	0.001353	0.000149	6.32	lnc-MAPK8IP2	lnc-MAPK8IP2-1:5	Upregulated
A_23_P128	0.000483	3.82E-06	6.27	KDELC1	KDEL (Lys-Asp-Glu-Leu) containing 1	Upregulated
A_24_P944	0.000483	2.91E-06	6.25	MAPKBP1	mitogen-activated protein kinase binding protein 1	Upregulated

A_23_P368	0.000483	2.66E-06	6.22	LRRC75A	leucine rich repeat containing 75A	Upregulated
A_33_P341	0.000483	2.88E-06	6.22	RHEB	Ras homolog enriched in brain	Upregulated
A_23_P368	0.000483	3.01E-06	6.18	GPRC5A	G protein-coupled receptor, class C, group 5, member A	Upregulated
A_24_P770	0.000483	3.29E-06	6.18	KMO	kynurenine 3-monooxygenase (kynurenine 3-hydroxylase)	Upregulated
A_23_P577	0.000483	1.54E-06	6.14	PXYLP1	2-phosphoxylose phosphatase 1	Upregulated
A_22_P000	0.000483	3.07E-06	6.1	LOC1005074	uncharacterized LOC100507487	Upregulated
A_22_P000	0.000483	3.25E-06	6.08	KMT2E-AS1	KMT2E antisense RNA 1 (head to head)	Upregulated
A_24_P334	0.000578	0.0000192	6.07	GPR141	G protein-coupled receptor 141	Upregulated
A_23_P948	0.000483	1.04E-06	6.04	F2	coagulation factor II (thrombin)	Upregulated
A_23_P297	0.000483	7.24E-07	6.03	SGOL1	shugoshin-like 1 (S. pombe)	Upregulated
A_22_P000	0.000483	2.45E-06	5.98	lnc-HNRNPA3	lnc-HNRNPA3-1:1	Upregulated
A_33_P335	0.000483	6.5E-07	5.94	LCTL	lactase-like	Upregulated
A_22_P000	0.001006	0.0000809	5.9	LOC1019293	uncharacterized LOC101929340	Upregulated
A_23_P124	0.000483	8.23E-07	5.86	COQ2	coenzyme Q2 4-hydroxybenzoate polyprenyltransferase	Upregulated
A_22_P000	0.000483	8.88E-07	5.86	lnc-ARNTL-1	lnc-ARNTL-1:1	Upregulated
A_24_P682	0.000483	3.29E-06	5.86	TRIM4	tripartite motif containing 4	Upregulated
A_32_P360	0.000483	0.0000036	5.82	DNHD1	dynein heavy chain domain 1	Upregulated
A_23_P130	0.000483	9.53E-07	5.79	KCTD1	potassium channel tetramerization domain containing 1	Upregulated
A_24_P213	0.000483	4.42E-06	5.76	SEMA6A	sema domain, transmembrane domain (TM), and cytoplasmic domain, (s	Upregulated
A_33_P337	0.000483	6.96E-07	5.75	GREB1	growth regulation by estrogen in breast cancer 1	Upregulated
A_33_P341	0.000526	0.0000123	5.75	BNIP3	BCL2/adenovirus E1B 19kDa interacting protein 3	Upregulated
A_23_P126	0.000978	0.0000754	5.74	NOL9	nucleolar protein 9	Upregulated
A_23_P127	0.000483	9.36E-07	5.73	IL2RA	interleukin 2 receptor, alpha	Upregulated
A_22_P000	0.0017	0.000226	5.73	lnc-ZBTB10-3	lnc-ZBTB10-3:1	Upregulated
A_33_P326	0.000489	0.0000074	5.72	SH3GLB1	SH3-domain GRB2-like endophilin B1	Upregulated
A_23_P385	0.001299	0.000139	5.72	PLCD4	phospholipase C, delta 4	Upregulated
A_22_P000	0.000526	0.000012	5.71	CDK3	cyclin-dependent kinase 3	Upregulated
A_22_P000	0.000565	0.0000169	5.71	lnc-TCEANC2	lnc-TCEANC2-1:1	Upregulated
A_23_P716	0.000848	0.0000541	5.7	FANCG	Fanconi anemia, complementation group G	Upregulated
A_23_P204	0.000483	4.95E-06	5.69	FARP1	FERM, RhoGEF (ARHGEF) and pleckstrin domain protein 1 (chondrocyt	Upregulated
A_33_P329	0.000486	6.64E-06	5.69	CEP55	centrosomal protein 55kDa	Upregulated
A_22_P000	0.000486	7.14E-06	5.66	lnc-PALLD-2	lnc-PALLD-2:1	Upregulated
A_24_P941	0.000483	7.51E-07	5.65	ZNF425	zinc finger protein 425	Upregulated
A_33_P334	0.000691	0.0000307	5.65	WDR60	WD repeat domain 60	Upregulated
A_24_P226	0.000483	4.22E-06	5.64	KIAA0368	KIAA0368	Upregulated
A_23_P335	0.000728	0.000038	5.64	ZNF721	zinc finger protein 721	Upregulated
A_22_P000	0.000483	4.72E-06	5.63	LRIG2	leucine-rich repeats and immunoglobulin-like domains 2	Upregulated

A_23_P533	0.000483	5.56E-06	5.63	TMEM177	transmembrane protein 177	Upregulated
A_23_P357	0.000483	7.86E-07	5.59	PNKD	paroxysmal nonkinesigenic dyskinesia	Upregulated
A_21_P000	0.000483	4.42E-06	5.59	EFTUD1	elongation factor Tu GTP binding domain containing 1	Upregulated
A_23_P341	0.000483	3.05E-06	5.58	WBP5	WW domain binding protein 5	Upregulated
A_23_P164	0.000483	1.81E-06	5.57	LIN7B	lin-7 homolog B (C. elegans)	Upregulated
A_21_P000	0.000611	0.0000232	5.57	PAXBP1-AS1	PAXBP1 antisense RNA 1	Upregulated
A_23_P449	0.000483	3.15E-06	5.53	DSC2	desmocollin 2	Upregulated
A_33_P323	0.000483	5.41E-06	5.53	HDGF	hepatoma-derived growth factor	Upregulated
A_21_P001	0.001372	0.000152	5.53	TMEM165	transmembrane protein 165	Upregulated
A_33_P681	0.000604	0.0000226	5.52	lnc-GTF2F2-2	lnc-GTF2F2-2:22	Upregulated
A_23_P109	0.000483	8.75E-07	5.49	SDC4	syndecan 4	Upregulated
A_24_P242	0.000483	6.07E-06	5.49	HS2ST1	heparan sulfate 2-O-sulfotransferase 1	Upregulated
A_22_P000	0.000484	6.45E-06	5.48	lnc-HIST2H2A	lnc-HIST2H2AA3-1:1	Upregulated
A_33_P322	0.000762	0.0000419	5.47	COQ3	coenzyme Q3 methyltransferase	Upregulated
A_23_P386	0.001051	0.0000887	5.47	DOLPP1	dolichyldiphosphatase 1	Upregulated
A_21_P000	0.000483	5.29E-06	5.44	ZNF587B	zinc finger protein 587B	Upregulated
A_23_P715	0.00124	0.000126	5.4	CSPP1	centrosome and spindle pole associated protein 1	Upregulated
A_24_P943	0.00056	0.0000161	5.38	SCUBE3	signal peptide, CUB domain, EGF-like 3	Upregulated
A_21_P000	0.000961	0.000072	5.37	lnc-MBOAT4-:	lnc-MBOAT4-2:1	Upregulated
A_33_P328	0.000483	1.95E-06	5.35	SLC16A1	solute carrier family 16 (monocarboxylate transporter), member 1	Upregulated
A_23_P363	0.000788	0.0000458	5.35	HSPA4L	heat shock 70kDa protein 4-like	Upregulated
A_23_P980	0.000483	9.13E-07	5.34	SIRT1	sirtuin 1	Upregulated
A_33_P331	0.000483	2.19E-06	5.34	STAG3	stromal antigen 3	Upregulated
A_24_P370	0.000483	5.77E-06	5.34	ATG10	autophagy related 10	Upregulated
A_24_P812	0.000483	2.45E-06	5.33	PPP6C	protein phosphatase 6, catalytic subunit	Upregulated
A_33_P371	0.00056	0.0000164	5.31	MEGF8	multiple EGF-like-domains 8	Upregulated
A_21_P001	0.000705	0.0000333	5.31	LOC1019299	uncharacterized LOC101929947	Upregulated
A_24_P984	0.000483	1.02E-06	5.3	HSPA5	heat shock 70kDa protein 5 (glucose-regulated protein, 78kDa)	Upregulated
A_23_P470	0.000483	3.89E-06	5.3	DHX32	DEAH (Asp-Glu-Ala-His) box polypeptide 32	Upregulated
A_21_P001	0.000483	9.93E-07	5.29	USP32P2	ubiquitin specific peptidase 32 pseudogene 2	Upregulated
A_24_P116	0.000483	1.33E-06	5.27	STAT3	signal transducer and activator of transcription 3 (acute-phase response	Upregulated
A_23_P420	0.000483	1.03E-06	5.26	PGBD4	piggyBac transposable element derived 4	Upregulated
A_22_P000	0.000483	5.51E-06	5.26	ADAT1	adenosine deaminase, tRNA-specific 1	Upregulated
A_23_P940	0.000483	1.17E-06	5.25	ANKRD46	ankyrin repeat domain 46	Upregulated
A_23_P134	0.000483	5.57E-06	5.23	CNKSR3	CNKSR family member 3	Upregulated
A_23_P357	0.000483	3.93E-06	5.22	GFOD2	glucose-fructose oxidoreductase domain containing 2	Upregulated
A_23_P365	0.001023	0.000084	5.21	FAM161B	family with sequence similarity 161, member B	Upregulated

A_24_P194	0.000483	0.000001	5.2	ZNF275	zinc finger protein 275	Upregulated
A_33_P337	0.000603	0.000022	5.2	TMEM106A	transmembrane protein 106A	Upregulated
A_33_P334	0.000483	3.94E-06	5.19	ZNF276	zinc finger protein 276	Upregulated
A_22_P000	0.000483	5.98E-06	5.19	lnc-KATNA1-2	lnc-KATNA1-2:1	Upregulated
A_23_P167	0.000483	2.21E-06	5.17	CYFIP2	cytoplasmic FMR1 interacting protein 2	Upregulated
A_32_P176	0.000521	0.0000106	5.17	LINC00239	long intergenic non-protein coding RNA 239	Upregulated
A_21_P000	0.001263	0.000132	5.17	lnc-SDPR-1	lnc-SDPR-1:1	Upregulated
A_33_P328	0.000483	5.66E-06	5.15	FUT4	fucosyltransferase 4 (alpha (1,3) fucosyltransferase, myeloid-specific)	Upregulated
A_33_P342	0.000506	8.67E-06	5.14	LOC1001320	uncharacterized LOC100132071	Upregulated
A_33_P681	0.000575	0.0000175	5.12	lnc-GTF2F2-2	lnc-GTF2F2-2:19	Upregulated
A_33_P328	0.001043	0.0000873	5.1	GZF1	GDNF-inducible zinc finger protein 1	Upregulated
A_23_P256	0.000483	6.38E-06	5.09	HPSE	heparanase	Upregulated
A_23_P134	0.000483	1.09E-06	5.08	PHF14	PHD finger protein 14	Upregulated
A_19_P003	0.001525	0.000186	5.08	SMIM13	small integral membrane protein 13	Upregulated
A_23_P762	0.000483	4.36E-06	5.07	PRR4	proline rich 4 (lacrimal)	Upregulated
A_22_P000	0.000483	4.55E-06	5.06	SLC37A4	solute carrier family 37 (glucose-6-phosphate transporter), member 4	Upregulated
A_33_P370	0.001226	0.000124	5.05	LINC00662	long intergenic non-protein coding RNA 662	Upregulated
A_21_P001	0.000515	9.41E-06	5.04	LINC01353	long intergenic non-protein coding RNA 1353	Upregulated
A_33_P326	0.000483	1.68E-06	5.02	RUFY2	RUN and FYVE domain containing 2	Upregulated
A_23_P429	0.000589	0.0000206	5.02	KCNQ1	potassium channel, voltage gated KQT-like subfamily Q, member 1	Upregulated
A_32_P593	0.001067	0.0000927	5.02	HIVEP3	human immunodeficiency virus type I enhancer binding protein 3	Upregulated
A_33_P336	0.000487	7.24E-06	5.01	BCAR1	breast cancer anti-estrogen resistance 1	Upregulated
A_23_P114	0.001022	0.0000837	5.01	SEMA4C	sema domain, immunoglobulin domain (Ig), transmembrane domain (TM	Upregulated
A_24_P849	0.000483	0.0000013	5	RPL22	ribosomal protein L22	Upregulated
A_33_P348	0.000483	0.0000014	5	ANKRD26P3	ankyrin repeat domain 26 pseudogene 3	Upregulated
A_33_P341	0.000483	1.18E-06	4.99	HSPA1B	heat shock 70kDa protein 1B	Upregulated
A_23_P111	0.000483	3.89E-06	4.99	GNG11	guanine nucleotide binding protein (G protein), gamma 11	Upregulated
A_33_P382	0.000483	4.97E-06	4.99	RABL6	RAB, member RAS oncogene family-like 6	Upregulated
A_24_P129	0.000513	8.98E-06	4.99	KCNIP2	Kv channel interacting protein 2	Upregulated
A_33_P342	0.00096	0.0000717	4.97	CNST	consortin, connexin sorting protein	Upregulated
A_22_P000	0.001032	0.0000854	4.96	KCNIP2-AS1	KCNIP2 antisense RNA 1	Upregulated
A_32_P177	0.000483	2.72E-06	4.95	STX17-AS1	STX17 antisense RNA 1	Upregulated
A_33_P322	0.000521	0.0000106	4.95	JAM2	junctional adhesion molecule 2	Upregulated
A_24_P701	0.000537	0.0000141	4.95	MYH11	myosin, heavy chain 11, smooth muscle	Upregulated
A_22_P000	0.000486	6.96E-06	4.94	lnc-TCF19-1	lnc-TCF19-1:4	Upregulated
A_22_P000	0.000483	1.79E-06	4.93	lnc-NUDT13-1	lnc-NUDT13-1:1	Upregulated
A_33_P330	0.000483	4.69E-06	4.93	DCUN1D2	DCN1, defective in cullin neddylation 1, domain containing 2	Upregulated

A_21_P001	0.000526	0.0000128	4.93	DDX11L16	DEAD/H (Asp-Glu-Ala-Asp/His) box helicase 11 like 16	Upregulated
A_23_P170	0.000532	0.0000134	4.93	IL36G	interleukin 36, gamma	Upregulated
A_24_P927	0.000483	0.0000061	4.92	C2CD3	C2 calcium-dependent domain containing 3	Upregulated
A_32_P137	0.000483	0.0000052	4.91	EFCAB7	EF-hand calcium binding domain 7	Upregulated
A_21_P000	0.000483	4.55E-06	4.9	LOC440896	uncharacterized LOC440896	Upregulated
A_23_P217	0.001341	0.000146	4.9	CTPS1	CTP synthase 1	Upregulated
A_21_P001	0.000483	1.51E-06	4.87	XLOC_l2_002311		Upregulated
A_22_P000	0.000483	5.06E-06	4.87	LOC1019270	uncharacterized LOC101927018	Upregulated
A_33_P321	0.000488	7.28E-06	4.87	LOC1001293	chromosome X open reading frame 69-like	Upregulated
A_23_P344	0.000502	8.31E-06	4.87	SLC35A3	solute carrier family 35 (UDP-N-acetylglucosamine (UDP-GlcNAc) transpo	Upregulated
A_23_P996	0.000486	7.14E-06	4.86	SLC7A7	solute carrier family 7 (amino acid transporter light chain, y+L system), m	Upregulated
A_24_P267	0.000483	1.49E-06	4.85	ZNF585A	zinc finger protein 585A	Upregulated
A_24_P483	0.000828	0.0000517	4.85	CWC25	CWC25 spliceosome-associated protein homolog (S. cerevisiae)	Upregulated
A_23_P388	0.000502	0.0000084	4.83	ZNF552	zinc finger protein 552	Upregulated
A_24_P339	0.000483	1.42E-06	4.82	ARSG	arylsulfatase G	Upregulated
A_22_P000	0.000701	0.0000329	4.81	lnc-CEP41-1	lnc-CEP41-1:1	Upregulated
A_23_P273	0.002688	0.00048	4.81	EMILIN2	elastin microfibril interfacer 2	Upregulated
A_33_P329	0.000483	5.41E-06	4.78	GRASP	GRP1 (general receptor for phosphoinositides 1)-associated scaffold pro	Upregulated
A_33_P325	0.000739	0.000039	4.77	LOC1001306	uncharacterized LOC100130642	Upregulated
A_33_P323	0.000546	0.0000148	4.75	MAP3K14-AS	MAP3K14 antisense RNA 1	Upregulated
A_24_P822	0.000706	0.0000334	4.75	HS6ST1	heparan sulfate 6-O-sulfotransferase 1	Upregulated
A_23_P823	0.000743	0.0000397	4.75	BBS9	Bardet-Biedl syndrome 9	Upregulated
A_33_P337	0.000483	4.56E-06	4.74	WAC	WW domain containing adaptor with coiled-coil	Upregulated
A_33_P327	0.000483	1.81E-06	4.73	TRMT10A	tRNA methyltransferase 10 homolog A (S. cerevisiae)	Upregulated
A_23_P512	0.000483	5.71E-06	4.73	ZNF436	zinc finger protein 436	Upregulated
A_23_P138	0.000954	0.0000705	4.73	STYK1	serine/threonine/tyrosine kinase 1	Upregulated
A_22_P000	0.001178	0.000114	4.72	LOC643339	uncharacterized LOC643339	Upregulated
A_23_P662	0.000483	1.78E-06	4.7	MT1M	metallothionein 1M	Upregulated
A_22_P000	0.000525	0.000011	4.7	lnc-SGSM2-1	lnc-SGSM2-1:1	Upregulated
A_23_P904	0.000588	0.0000204	4.7	PBX4	pre-B-cell leukemia homeobox 4	Upregulated
A_24_P341	0.000765	0.0000425	4.7	GBA2	glucosidase, beta (bile acid) 2	Upregulated
A_33_P339	0.000483	3.25E-06	4.69	C17orf96	chromosome 17 open reading frame 96	Upregulated
A_21_P000	0.000526	0.0000127	4.69	lnc-C1orf21-3	lnc-C1orf21-3:1	Upregulated
A_33_P326	0.000483	2.26E-06	4.68	SNX1	sorting nexin 1	Upregulated
A_23_P459	0.000483	4.42E-06	4.68	TFB2M	transcription factor B2, mitochondrial	Upregulated
A_22_P000	0.000497	0.0000076	4.68	lnc-C10orf103	lnc-C10orf103-1:1	Upregulated
A_24_P113	0.000483	1.95E-06	4.67	ATAD5	ATPase family, AAA domain containing 5	Upregulated

A_33_P337	0.000483	1.95E-06	4.67	ENSA	endosulfine alpha	Upregulated
A_23_P991	0.00076	0.0000416	4.67	CCDC77	coiled-coil domain containing 77	Upregulated
A_23_P161	0.001092	0.0000975	4.67	FOSL1	FOS-like antigen 1	Upregulated
A_23_P111	0.000483	1.81E-06	4.64	HSPA1A	heat shock 70kDa protein 1A	Upregulated
A_33_P323	0.000525	0.0000115	4.64	STK3	serine/threonine kinase 3	Upregulated
A_32_P904	0.000483	4.86E-06	4.63	STXBP4	syntaxin binding protein 4	Upregulated
A_23_P200	0.000537	0.0000138	4.63	HILPDA	hypoxia inducible lipid droplet-associated	Upregulated
A_23_P120	0.000483	2.96E-06	4.62	ACSS1	acyl-CoA synthetase short-chain family member 1	Upregulated
A_23_P353	0.000483	3.23E-06	4.61	C2CD2L	C2CD2-like	Upregulated
A_23_P145	0.000526	0.0000124	4.61	STAG3	stromal antigen 3	Upregulated
A_23_P163	0.001088	0.0000968	4.61	POLE2	polymerase (DNA directed), epsilon 2, accessory subunit	Upregulated
A_23_P152	0.000483	2.56E-06	4.6	PLEKHM1	pleckstrin homology domain containing, family M (with RUN domain) mer	Upregulated
A_23_P151	0.000695	0.0000313	4.6	C12orf43	chromosome 12 open reading frame 43	Upregulated
A_23_P665	0.002044	0.000313	4.6	SMG8	SMG8 nonsense mediated mRNA decay factor	Upregulated
A_22_P000	0.001108	0.000101	4.59	lnc-ERH-1	lnc-ERH-1:1	Upregulated
A_23_P421	0.000483	2.49E-06	4.58	FAM83H	family with sequence similarity 83, member H	Upregulated
A_33_P335	0.000662	0.0000279	4.58	TXNRD1	thioredoxin reductase 1	Upregulated
A_33_P330	0.000483	1.75E-06	4.57	DOPEY1	dopey family member 1	Upregulated
A_33_P331	0.000483	0.0000064	4.56	IMMT	inner membrane protein, mitochondrial	Upregulated
A_23_P145	0.000486	7.16E-06	4.56	PPP1R17	protein phosphatase 1, regulatory subunit 17	Upregulated
A_24_P873	0.000515	9.22E-06	4.56	BCR	breakpoint cluster region	Upregulated
A_21_P001	0.000625	0.0000246	4.56	lnc-MAPK8IP2	lnc-MAPK8IP2-1:12	Upregulated
A_33_P335	0.001957	0.000289	4.56	SARS	seryl-tRNA synthetase	Upregulated
A_21_P000	0.000483	2.31E-06	4.55	MT1E	metallothionein 1E	Upregulated
A_23_P202	0.000515	0.0000094	4.54	KBTBD4	kelch repeat and BTB (POZ) domain containing 4	Upregulated
A_23_P349	0.000527	0.0000129	4.53	FCHO2	FCH domain only 2	Upregulated
A_23_P340	0.000538	0.0000143	4.53	THAP3	THAP domain containing, apoptosis associated protein 3	Upregulated
A_23_P144	0.000741	0.0000394	4.53	PDLIM7	PDZ and LIM domain 7 (enigma)	Upregulated
A_33_P324	0.00219	0.000352	4.53	CCNE2	cyclin E2	Upregulated
A_24_P816	0.003373	0.00068	4.53	UBE2Q2P1	ubiquitin-conjugating enzyme E2Q family member 2 pseudogene 1	Upregulated
A_22_P000	0.000483	2.55E-06	4.52	lnc-SPAG1-3	lnc-SPAG1-3:4	Upregulated
A_32_P602	0.000483	1.96E-06	4.51	ING5	inhibitor of growth family, member 5	Upregulated
A_23_P131	0.000483	3.79E-06	4.51	CD302	CD302 molecule	Upregulated
A_33_P342	0.000483	0.0000046	4.51	XLOC_l2_000407		Upregulated
A_22_P000	0.000526	0.0000126	4.51	lnc-RSPRY1-1	lnc-RSPRY1-1:1	Upregulated
A_21_P000	0.000765	0.0000423	4.51	lnc-AL035696	lnc-AL035696.1-5:1	Upregulated
A_33_P333	0.000483	2.02E-06	4.5	NRARP	NOTCH-regulated ankyrin repeat protein	Upregulated

A_23_P698	0.000483	2.22E-06	4.5	ZNF717	zinc finger protein 717	Upregulated
A_21_P000	0.000486	7.17E-06	4.5	lnc-HERPUD2	lnc-HERPUD2-4:1	Upregulated
A_33_P332	0.000485	0.0000065	4.49	TOMM34	translocase of outer mitochondrial membrane 34	Upregulated
A_33_P333	0.000515	9.49E-06	4.48	MOSPD1	motile sperm domain containing 1	Upregulated
A_33_P332	0.000687	0.0000302	4.48	PLAGL2	pleiomorphic adenoma gene-like 2	Upregulated
A_32_P197	0.002351	0.000391	4.47	EIF2A	eukaryotic translation initiation factor 2A, 65kDa	Upregulated
A_23_P151	0.00321	0.000633	4.47	TMEM132C	transmembrane protein 132C	Upregulated
A_24_P898	0.000485	6.51E-06	4.46	TRAF1	TNF receptor-associated factor 1	Upregulated
A_33_P329	0.000483	3.67E-06	4.45	LOC1005055	uncharacterized LOC100505530	Upregulated
A_33_P325	0.000525	0.0000115	4.45	DOCK9	dedicator of cytokinesis 9	Upregulated
A_32_P538	0.000625	0.0000246	4.45	PAQR7	progestin and adipoQ receptor family member VII	Upregulated
A_23_P162	0.000925	0.0000652	4.44	KLK1	kallikrein 1	Upregulated
A_21_P001	0.000526	0.0000117	4.43	lnc-KIF3A-1	lnc-KIF3A-1:4	Upregulated
A_33_P337	0.000498	7.73E-06	4.42	SESTD1	SEC14 and spectrin domains 1	Upregulated
A_24_P231	0.000515	9.86E-06	4.42	GIGYF1	GRB10 interacting GYF protein 1	Upregulated
A_33_P321	0.000483	2.16E-06	4.41	CAB39L	calcium binding protein 39-like	Upregulated
A_24_P253	0.000483	3.13E-06	4.41	PIGL	phosphatidylinositol glycan anchor biosynthesis, class L	Upregulated
A_33_P333	0.000646	0.0000265	4.41	LCORL	ligand dependent nuclear receptor corepressor-like	Upregulated
A_22_P000	0.000483	5.63E-06	4.39	STX18-AS1	STX18 antisense RNA 1 (head to head)	Upregulated
A_22_P000	0.000522	0.0000108	4.39	LOC1027237	uncharacterized LOC102723766	Upregulated
A_22_P000	0.000486	6.67E-06	4.38	SLCO5A1	solute carrier organic anion transporter family, member 5A1	Upregulated
A_23_P973	0.000483	3.36E-06	4.37	CASP9	caspase 9, apoptosis-related cysteine peptidase	Upregulated
A_23_P630	0.000806	0.0000485	4.37	MSTO1	misato 1, mitochondrial distribution and morphology regulator	Upregulated
A_24_P261	0.001596	0.0002	4.37	MTMR9	myotubularin related protein 9	Upregulated
A_22_P000	0.000483	0.0000019	4.36	lnc-MBOAT1-!	lnc-MBOAT1-5:1	Upregulated
A_23_P503	0.000521	0.0000104	4.36	LGALS13	lectin, galactoside-binding, soluble, 13	Upregulated
A_24_P944	0.000487	7.23E-06	4.33	KLHL6	kelch-like family member 6	Upregulated
A_33_P340	0.000577	0.000018	4.33	ST8SIA6	ST8 alpha-N-acetyl-neuraminide alpha-2,8-sialyltransferase 6	Upregulated
A_24_P941	0.000483	1.96E-06	4.32	HAUS6	HAUS augmin-like complex, subunit 6	Upregulated
A_33_P340	0.000515	9.49E-06	4.32	PPM1F	protein phosphatase, Mg2+/Mn2+ dependent, 1F	Upregulated
A_22_P000	0.000979	0.0000755	4.32	lnc-TP53I11-5	lnc-TP53I11-5:1	Upregulated
A_23_P268	0.000513	0.0000089	4.31	SOX9	SRY (sex determining region Y)-box 9	Upregulated
A_23_P135	0.000525	0.0000111	4.31	ZNF627	zinc finger protein 627	Upregulated
A_21_P000	0.000483	2.45E-06	4.3	LOC1019295	uncharacterized LOC101929506	Upregulated
A_24_P415	0.000483	3.52E-06	4.3	SEC61A2	Sec61 alpha 2 subunit (S. cerevisiae)	Upregulated
A_33_P326	0.0011	0.0000993	4.3	MTMR7	myotubularin related protein 7	Upregulated
A_23_P151	0.000525	0.0000115	4.29	CSRNP2	cysteine-serine-rich nuclear protein 2	Upregulated

A_23_P102	0.000559	0.0000155	4.29	NUP35	nucleoporin 35kDa	Upregulated
A_24_P148	0.001105	0.0001	4.29	SH3BP5	SH3-domain binding protein 5 (BTK-associated)	Upregulated
A_22_P000	0.00308	0.000591	4.29	lnc-STAT3-1	lnc-STAT3-1:1	Upregulated
A_32_P250	0.000483	3.44E-06	4.28	RDH10	retinol dehydrogenase 10 (all-trans)	Upregulated
A_23_P453	0.000498	7.84E-06	4.28	XK	X-linked Kx blood group	Upregulated
A_33_P331	0.001121	0.000104	4.28	MIR17HG	miR-17-92 cluster host gene (non-protein coding)	Upregulated
A_24_P358	0.001035	0.0000858	4.27	GPATCH11	G patch domain containing 11	Upregulated
A_23_P635	0.003109	0.000602	4.27	PMF1	polyamine-modulated factor 1	Upregulated
A_23_P132	0.000483	5.72E-06	4.26	FAM114A1	family with sequence similarity 114, member A1	Upregulated
A_32_P151	0.000526	0.0000118	4.26	ZNF398	zinc finger protein 398	Upregulated
A_19_P003	0.000483	2.14E-06	4.25	PTPN14	protein tyrosine phosphatase, non-receptor type 14	Upregulated
A_21_P000	0.001004	0.0000805	4.25	lnc-UBLCP1-8	lnc-UBLCP1-8:1	Upregulated
A_33_P327	0.002535	0.000439	4.25	DIS3L2	DIS3 like 3'-5' exoribonuclease 2	Upregulated
A_33_P323	0.000521	0.0000106	4.24	MINOS1-NBL	MINOS1-NBL1 readthrough	Upregulated
A_24_P630	0.000565	0.0000169	4.24	IL1R2	interleukin 1 receptor, type II	Upregulated
A_24_P294	0.00071	0.0000353	4.24	SYNJ2	synaptojanin 2	Upregulated
A_22_P000	0.000483	5.12E-06	4.23	lnc-NGDN-1	lnc-NGDN-1:1	Upregulated
A_33_P320	0.000855	0.0000554	4.23	ZNF345	zinc finger protein 345	Upregulated
A_33_P373	0.001103	0.0000998	4.23	LOC1005062	uncharacterized LOC100506282	Upregulated
A_22_P000	0.001346	0.000147	4.23	lnc-RIC8A-1	lnc-RIC8A-1:1	Upregulated
A_23_P277	0.001394	0.000157	4.23	SPINT2	serine peptidase inhibitor, Kunitz type, 2	Upregulated
A_24_P194	0.000483	2.23E-06	4.22	CCDC134	coiled-coil domain containing 134	Upregulated
A_24_P944	0.000752	0.0000408	4.22	TMTC3	transmembrane and tetratricopeptide repeat containing 3	Upregulated
A_33_P328	0.001687	0.000221	4.22	RNPC3	RNA-binding region (RNP1, RRM) containing 3	Upregulated
A_23_P709	0.000483	2.75E-06	4.21	AGA	aspartylglucosaminidase	Upregulated
A_24_P376	0.000483	2.97E-06	4.21	CCNL2	cyclin L2	Upregulated
A_23_P157	0.000515	9.87E-06	4.21	AP4M1	adaptor-related protein complex 4, mu 1 subunit	Upregulated
A_33_P326	0.00064	0.0000259	4.21	FZR1	fizzy/cell division cycle 20 related 1 (Drosophila)	Upregulated
A_23_P447	0.000483	2.71E-06	4.2	CSRP2	cysteine and glycine-rich protein 2	Upregulated
A_24_P528	0.000483	2.92E-06	4.2	ENDOU	endonuclease, polyU-specific	Upregulated
A_33_P331	0.000483	3.22E-06	4.2	MTERF4	mitochondrial transcription termination factor 4	Upregulated
A_22_P000	0.000945	0.0000691	4.2	LOC1019272	uncharacterized LOC101927257	Upregulated
A_24_P106	0.000995	0.000079	4.2	WDR36	WD repeat domain 36	Upregulated
A_23_P377	0.000883	0.0000591	4.19	CIRBP	cold inducible RNA binding protein	Upregulated
A_33_P339	0.001459	0.000172	4.19	IL4	interleukin 4	Upregulated
A_22_P000	0.000483	3.79E-06	4.17	lnc-VPRBP-1	lnc-VPRBP-1:1	Upregulated
A_19_P008	0.000526	0.0000126	4.17	OTUD6B-AS1	OTUD6B antisense RNA 1 (head to head)	Upregulated

A_23_P301	0.00056	0.0000165	4.17	MED27	mediator complex subunit 27	Upregulated
A_23_P193	0.000575	0.0000177	4.17	LRRC16A	leucine rich repeat containing 16A	Upregulated
A_21_P000	0.000752	0.0000408	4.17	SNORA29	small nucleolar RNA, H/ACA box 29	Upregulated
A_22_P000	0.001955	0.000288	4.17	LLPH-AS1	LLPH antisense RNA 1 (head to head)	Upregulated
A_21_P000	0.000695	0.0000315	4.16	lnc-ASMT-5	lnc-ASMT-5:1	Upregulated
A_33_P325	0.000908	0.0000628	4.16	NSG1	neuron specific gene family member 1	Upregulated
A_21_P000	0.000908	0.0000628	4.16	SNORA21	small nucleolar RNA, H/ACA box 21	Upregulated
A_33_P329	0.000598	0.0000214	4.15	MYO1E	myosin IE	Upregulated
A_24_P374	0.000925	0.0000653	4.15	PDK1	pyruvate dehydrogenase kinase, isozyme 1	Upregulated
A_33_P329	0.000483	2.61E-06	4.14	MRPL37	mitochondrial ribosomal protein L37	Upregulated
A_23_P255	0.000483	6.29E-06	4.14	PAFAH1B2	platelet-activating factor acetylhydrolase 1b, catalytic subunit 2 (30kDa)	Upregulated
A_24_P314	0.000483	2.41E-06	4.12	TUBB2B	tubulin, beta 2B class IIb	Upregulated
A_33_P336	0.000516	0.00001	4.12	SCARNA10	small Cajal body-specific RNA 10	Upregulated
A_23_P895	0.000516	0.0000101	4.12	KIAA0753	KIAA0753	Upregulated
A_24_P393	0.001142	0.000108	4.12	DNAJB4	DnaJ (Hsp40) homolog, subfamily B, member 4	Upregulated
A_21_P000	0.000498	7.88E-06	4.11	lnc-TMEM116	lnc-TMEM116-1:1	Upregulated
A_33_P322	0.000532	0.0000133	4.11	ID4	inhibitor of DNA binding 4, dominant negative helix-loop-helix protein	Upregulated
A_24_P933	0.001346	0.000147	4.11	ABI2	abl-interactor 2	Upregulated
A_23_P650	0.000709	0.0000349	4.1	EID3	EP300 interacting inhibitor of differentiation 3	Upregulated
A_24_P940	0.000909	0.000063	4.1	CNOT6	CCR4-NOT transcription complex, subunit 6	Upregulated
A_23_P139	0.003147	0.000612	4.1	SLC11A2	solute carrier family 11 (proton-coupled divalent metal ion transporter), m	Upregulated
A_33_P322	0.00058	0.0000196	4.09	ARHGAP26	Rho GTPase activating protein 26	Upregulated
A_24_P212	0.00069	0.0000304	4.09	C9orf156	chromosome 9 open reading frame 156	Upregulated
A_23_P131	0.001385	0.000154	4.09	UBXN2A	UBX domain protein 2A	Upregulated
A_22_P000	0.003481	0.000712	4.09	lnc-SDF2-1	lnc-SDF2-1:1	Upregulated
A_23_P217	0.000486	6.81E-06	4.08	DCAF10	DDB1 and CUL4 associated factor 10	Upregulated
A_33_P328	0.000504	8.58E-06	4.08	SLTM	SAFB-like, transcription modulator	Upregulated
A_23_P698	0.000525	0.0000114	4.07	AGPAT9	1-acylglycerol-3-phosphate O-acyltransferase 9	Upregulated
A_22_P000	0.000534	0.0000135	4.07	TAPT1-AS1	TAPT1 antisense RNA 1 (head to head)	Upregulated
A_21_P000	0.000592	0.0000208	4.07	LINC00278	long intergenic non-protein coding RNA 278	Upregulated
A_23_P795	0.0027	0.000483	4.07	IL1B	interleukin 1, beta	Upregulated
A_21_P001	0.000483	2.66E-06	4.06	CLCN3	chloride channel, voltage-sensitive 3	Upregulated
A_22_P000	0.000526	0.0000118	4.06	lnc-CCDC7-2	lnc-CCDC7-2:1	Upregulated
A_23_P375	0.00056	0.0000163	4.06	NPTN	neuroplastin	Upregulated
A_33_P323	0.000674	0.0000291	4.06	MBTD1	mbt domain containing 1	Upregulated
A_24_P402	0.000483	6.08E-06	4.05	CRNKL1	crooked neck pre-mRNA splicing factor 1	Upregulated
A_23_P124	0.001092	0.0000974	4.05	ISY1	ISY1 splicing factor homolog (S. cerevisiae)	Upregulated

A_19_P003	0.000483	3.08E-06	4.04	LOC1019297	uncharacterized LOC101929709	Upregulated
A_23_P123	0.000515	9.96E-06	4.04	GSTM3	glutathione S-transferase mu 3 (brain)	Upregulated
A_33_P321	0.000525	0.0000113	4.04	AEN	apoptosis enhancing nuclease	Upregulated
A_24_P318	0.000537	0.0000139	4.04	ZNF337	zinc finger protein 337	Upregulated
A_23_P423	0.000702	0.000033	4.04	FAM171B	family with sequence similarity 171, member B	Upregulated
A_23_P842	0.001959	0.00029	4.04	OTP	orthopedia homeobox	Upregulated
A_33_P325	0.000498	7.76E-06	4.03	CNTRL	centriolin	Upregulated
A_23_P118	0.000741	0.0000396	4.03	PLK1	polo-like kinase 1	Upregulated
A_33_P323	0.001314	0.000141	4.03	CCNG1	cyclin G1	Upregulated
A_33_P341	0.003584	0.000745	4.03	SLC35F1	solute carrier family 35, member F1	Upregulated
A_33_P323	0.000483	0.0000026	4.02	MT1X	metallothionein 1X	Upregulated
A_23_P111	0.000521	0.0000102	4.01	SRRT	serrate, RNA effector molecule	Upregulated
A_22_P000	0.000746	0.0000402	4.01	lnc-CUBN-1	lnc-CUBN-1:1	Upregulated
A_23_P104	0.000701	0.0000328	4	SPA17	sperm autoantigenic protein 17	Upregulated
A_22_P000	0.000483	5.82E-06	3.99	lnc-SPATA17	lnc-SPATA17-2:1	Upregulated
A_24_P365	0.000705	0.0000332	3.99	TCF3	transcription factor 3	Upregulated
A_33_P326	0.001081	0.0000952	3.99	BAD	BCL2-associated agonist of cell death	Upregulated
A_33_P326	0.002309	0.00038	3.99	C5orf49	chromosome 5 open reading frame 49	Upregulated
A_33_P337	0.000513	9.02E-06	3.98	FOXO3	forkhead box O3	Upregulated
A_22_P000	0.000526	0.0000122	3.98	SLC38A6	solute carrier family 38, member 6	Upregulated
A_23_P347	0.000575	0.0000177	3.98	COA7	cytochrome c oxidase assembly factor 7 (putative)	Upregulated
A_19_P003	0.000584	0.00002	3.98	lnc-LRIG2-4	lnc-LRIG2-4:3	Upregulated
A_22_P000	0.000604	0.0000225	3.98	lnc-RTN2-1	lnc-RTN2-1:1	Upregulated
A_23_P355	0.001341	0.000146	3.98	USP54	ubiquitin specific peptidase 54	Upregulated
A_23_P109	0.00053	0.0000131	3.97	HPS4	Hermansky-Pudlak syndrome 4	Upregulated
A_33_P331	0.000542	0.0000145	3.97	CA5B	carbonic anhydrase VB, mitochondrial	Upregulated
A_33_P327	0.000866	0.0000572	3.97	THAP9	THAP domain containing 9	Upregulated
A_23_P149	0.000629	0.0000249	3.96	THEM4	thioesterase superfamily member 4	Upregulated
A_22_P000	0.001752	0.000237	3.96	CLUAP1	clusterin associated protein 1	Upregulated
A_21_P001	0.001162	0.000111	3.95	lnc-WDR34-1	lnc-WDR34-1:1	Upregulated
A_23_P488	0.00062	0.0000241	3.94	TMOD2	tropomodulin 2 (neuronal)	Upregulated
A_21_P001	0.001029	0.0000848	3.94	LRRC37BP1	leucine rich repeat containing 37B pseudogene 1	Upregulated
A_23_P120	0.000515	9.44E-06	3.93	HMOX1	heme oxygenase (decycling) 1	Upregulated
A_23_P212	0.00056	0.0000157	3.93	LARS2	leucyl-tRNA synthetase 2, mitochondrial	Upregulated
A_21_P000	0.000639	0.0000257	3.93	lnc-NLGN4Y-1	lnc-NLGN4Y-1:1	Upregulated
A_23_P989	0.000483	3.84E-06	3.92	CCNT1	cyclin T1	Upregulated
A_23_P393	0.000483	4.04E-06	3.92	PDXDC2P	pyridoxal-dependent decarboxylase domain containing 2, pseudogene	Upregulated

A_33_P334	0.000701	0.0000328	3.92	PLCE1	phospholipase C, epsilon 1	Upregulated
A_24_P338	0.001101	0.0000995	3.92	ARL1	ADP-ribosylation factor-like 1	Upregulated
A_23_P106	0.001116	0.000102	3.92	SEMA7A	semaphorin 7A, GPI membrane anchor (John Milton Hagen blood group	Upregulated
A_22_P000	0.001477	0.000175	3.92	lnc-ACOT1-3	lnc-ACOT1-3:2	Upregulated
A_24_P349	0.000483	3.31E-06	3.91	PCDHGA12	protocadherin gamma subfamily A, 12	Upregulated
A_24_P270	0.001925	0.00028	3.91	CRK	v-crk avian sarcoma virus CT10 oncogene homolog	Upregulated
A_24_P291	0.000603	0.0000221	3.9	SYTL3	synaptotagmin-like 3	Upregulated
A_32_P482	0.000707	0.0000346	3.9	ZNF100	zinc finger protein 100	Upregulated
A_24_P185	0.000711	0.0000356	3.9	UPRT	uracil phosphoribosyltransferase (FUR1) homolog (S. cerevisiae)	Upregulated
A_32_P181	0.000515	9.93E-06	3.89	ST7-AS1	ST7 antisense RNA 1	Upregulated
A_23_P128	0.000522	0.0000108	3.89	BATF	basic leucine zipper transcription factor, ATF-like	Upregulated
A_21_P001	0.000526	0.0000122	3.89	LOC1019286	uncharacterized LOC101928658	Upregulated
A_23_P821	0.000786	0.0000454	3.89	SMPD2	sphingomyelin phosphodiesterase 2, neutral membrane (neutral sphingo	Upregulated
A_24_P363	0.000577	0.0000179	3.88	MAK	male germ cell-associated kinase	Upregulated
A_33_P342	0.000642	0.0000262	3.88	ZC3H3	zinc finger CCCH-type containing 3	Upregulated
A_24_P186	0.001328	0.000144	3.88	MTERF4	mitochondrial transcription termination factor 4	Upregulated
A_33_P329	0.000489	7.35E-06	3.87	C17orf53	chromosome 17 open reading frame 53	Upregulated
A_21_P000	0.000527	0.000013	3.87	TFAP2A-AS1	TFAP2A antisense RNA 1	Upregulated
A_33_P322	0.000515	9.94E-06	3.86	CAAP1	caspase activity and apoptosis inhibitor 1	Upregulated
A_23_P205	0.000662	0.0000278	3.86	EXD2	exonuclease 3'-5' domain containing 2	Upregulated
A_23_P346	0.002226	0.000361	3.86	PIK3CB	phosphatidylinositol-4,5-bisphosphate 3-kinase, catalytic subunit beta	Upregulated
A_24_P232	0.000537	0.000014	3.85	APBB1IP	amyloid beta (A4) precursor protein-binding, family B, member 1 interact	Upregulated
A_23_P312	0.000855	0.0000553	3.85	PILRA	paired immunoglobin-like type 2 receptor alpha	Upregulated
A_23_P504	0.000995	0.0000786	3.85	KANK2	KN motif and ankyrin repeat domains 2	Upregulated
A_23_P202	0.001243	0.000128	3.85	CCND1	cyclin D1	Upregulated
A_22_P000	0.000483	3.56E-06	3.84	lnc-ALDH7A1	lnc-ALDH7A1-1:1	Upregulated
A_33_P331	0.000741	0.0000396	3.84	C16orf62	chromosome 16 open reading frame 62	Upregulated
A_33_P330	0.001153	0.000109	3.84	LRP6	low density lipoprotein receptor-related protein 6	Upregulated
A_23_P367	0.001067	0.0000927	3.83	FBXL14	F-box and leucine-rich repeat protein 14	Upregulated
A_23_P397	0.000483	4.46E-06	3.82	GSTM2	glutathione S-transferase mu 2 (muscle)	Upregulated
A_33_P336	0.000771	0.0000432	3.82	DNAJB5	DnaJ (Hsp40) homolog, subfamily B, member 5	Upregulated
A_33_P331	0.000855	0.0000552	3.82	ZNF789	zinc finger protein 789	Upregulated
A_24_P364	0.001163	0.000112	3.82	SERGEF	secretion regulating guanine nucleotide exchange factor	Upregulated
A_23_P333	0.002006	0.000302	3.82	TTLL11	tubulin tyrosine ligase-like family member 11	Upregulated
A_24_P341	0.000483	5.09E-06	3.81	SMIM20	small integral membrane protein 20	Upregulated
A_33_P338	0.000486	0.0000071	3.81	KIF26B	kinesin family member 26B	Upregulated
A_23_P140	0.000709	0.0000349	3.81	ZNF263	zinc finger protein 263	Upregulated

A_22_P000	0.001181	0.000115	3.81	lnc-C6orf228-	lnc-C6orf228-3:1	Upregulated
A_33_P338	0.000846	0.0000539	3.8	ELK1	ELK1, member of ETS oncogene family	Upregulated
A_24_P405	0.00056	0.0000157	3.79	TRIP6	thyroid hormone receptor interactor 6	Upregulated
A_23_P433	0.000697	0.0000319	3.79	OR7E91P	olfactory receptor, family 7, subfamily E, member 91 pseudogene	Upregulated
A_24_P101	0.000537	0.000014	3.78	EXOC5	exocyst complex component 5	Upregulated
A_33_P336	0.000577	0.0000182	3.78	lnc-COPZ2-1	lnc-COPZ2-1:1	Upregulated
A_33_P339	0.000483	3.53E-06	3.77	PLSCR3	phospholipid scramblase 3	Upregulated
A_33_P321	0.000513	0.0000089	3.77	LNP1	leukemia NUP98 fusion partner 1	Upregulated
A_33_P337	0.001085	0.0000958	3.77	LOC1001332	GALI1870	Upregulated
A_23_P339	0.002128	0.000337	3.77	LIN52	lin-52 DREAM MuvB core complex component	Upregulated
A_23_P122	0.000483	3.27E-06	3.76	MCOLN3	mucolipin 3	Upregulated
A_23_P364	0.000575	0.0000178	3.76	VPS33A	vacuolar protein sorting 33 homolog A (S. cerevisiae)	Upregulated
A_33_P344	0.000577	0.0000182	3.76	lnc-WDR1-1	lnc-WDR1-1:2	Upregulated
A_23_P218	0.000483	0.0000038	3.75	CPT1B	carnitine palmitoyltransferase 1B (muscle)	Upregulated
A_23_P159	0.000515	9.79E-06	3.75	BEX1	brain expressed, X-linked 1	Upregulated
A_33_P340	0.001094	0.0000977	3.75	CTNNA1	catenin (cadherin-associated protein), alpha 1, 102kDa	Upregulated
A_23_P642	0.001542	0.000189	3.75	ZW10	zw10 kinetochore protein	Upregulated
A_22_P000	0.000483	0.0000064	3.74	lnc-RBMS1-2	lnc-RBMS1-2:1	Upregulated
A_33_P333	0.000512	8.82E-06	3.74	TTLL5	tubulin tyrosine ligase-like family member 5	Upregulated
A_33_P325	0.000675	0.0000293	3.74	ZNF286B	zinc finger protein 286B	Upregulated
A_23_P218	0.003175	0.000622	3.74	ZNF343	zinc finger protein 343	Upregulated
A_33_P340	0.00096	0.0000715	3.73	NAV1	neuron navigator 1	Upregulated
A_24_P164	0.001798	0.000248	3.73	DSCR3	Down syndrome critical region 3	Upregulated
A_23_P116	0.00058	0.0000196	3.71	USP1	ubiquitin specific peptidase 1	Upregulated
A_23_P837	0.003258	0.000646	3.71	ING2	inhibitor of growth family, member 2	Upregulated
A_24_P479	0.003295	0.000658	3.71	UBE2QL1	ubiquitin-conjugating enzyme E2Q family-like 1	Upregulated
A_21_P001	0.000483	4.06E-06	3.7	XLOC_l2_013457		Upregulated
A_23_P129	0.000483	4.24E-06	3.7	CALML4	calmodulin-like 4	Upregulated
A_33_P322	0.000525	0.0000115	3.7	LINC01347	long intergenic non-protein coding RNA 1347	Upregulated
A_21_P001	0.002907	0.00054	3.7	SMAD1-AS2	SMAD1 antisense RNA 2	Upregulated
A_33_P325	0.000809	0.000049	3.69	ATG2A	autophagy related 2A	Upregulated
A_22_P000	0.001084	0.0000955	3.69	lnc-DPY19L2-	lnc-DPY19L2-1:1	Upregulated
A_24_P101	0.001496	0.00018	3.69	THADA	thyroid adenoma associated	Upregulated
A_33_P329	0.000483	4.32E-06	3.68	PPIH	peptidylprolyl isomerase H (cyclophilin H)	Upregulated
A_22_P000	0.000502	8.46E-06	3.68	PDK3	pyruvate dehydrogenase kinase, isozyme 3	Upregulated
A_22_P000	0.00093	0.000066	3.68	GNG12-AS1	GNG12 antisense RNA 1	Upregulated
A_33_P327	0.002221	0.000359	3.68	AKAP14	A kinase (PRKA) anchor protein 14	Upregulated

A_22_P000	0.007055	0.00199	3.68	FSIP1	fibrous sheath interacting protein 1	Upregulated
A_23_P338	0.000483	4.91E-06	3.67	MAGED2	melanoma antigen family D, 2	Upregulated
A_23_P142	0.000483	5.93E-06	3.67	ZFYVE21	zinc finger, FYVE domain containing 21	Upregulated
A_23_P102	0.000973	0.0000746	3.67	WNT10A	wingless-type MMTV integration site family, member 10A	Upregulated
A_24_P177	0.001605	0.000203	3.67	PCDHGB8P	protocadherin gamma subfamily B, 8 pseudogene	Upregulated
A_33_P326	0.002945	0.000551	3.67	DDRGK1	DDRGK domain containing 1	Upregulated
A_23_P169	0.000537	0.0000142	3.66	TRUB2	TruB pseudouridine (psi) synthase family member 2	Upregulated
A_24_P241	0.002508	0.000432	3.66	ZNF160	zinc finger protein 160	Upregulated
A_21_P001	0.002788	0.000507	3.66	FBXO25	F-box protein 25	Upregulated
A_22_P000	0.000483	0.0000056	3.65	lnc-HIST4H4-	lnc-HIST4H4-1:1	Upregulated
A_32_P192	0.000483	0.0000058	3.65	GLT1D1	glycosyltransferase 1 domain containing 1	Upregulated
A_23_P591	0.000887	0.0000598	3.65	RXRB	retinoid X receptor, beta	Upregulated
A_23_P680	0.001002	0.0000803	3.65	PCGF1	polycomb group ring finger 1	Upregulated
A_21_P000	0.00213	0.000337	3.64	LOC1019272	uncharacterized LOC101927252	Upregulated
A_23_P705	0.000483	4.55E-06	3.63	VPS52	vacuolar protein sorting 52 homolog (S. cerevisiae)	Upregulated
A_23_P167	0.000589	0.0000204	3.63	PAQR3	progestin and adipoQ receptor family member III	Upregulated
A_23_P184	0.000701	0.0000326	3.63	CXCL9	chemokine (C-X-C motif) ligand 9	Upregulated
A_23_P144	0.000848	0.0000542	3.63	GNPDA1	glucosamine-6-phosphate deaminase 1	Upregulated
A_33_P340	0.001637	0.000211	3.63	SERINC4	serine incorporator 4	Upregulated
A_23_P668	0.005125	0.00126	3.63	SIRT7	sirtuin 7	Upregulated
A_23_P894	0.000483	3.86E-06	3.62	SLC35B1	solute carrier family 35, member B1	Upregulated
A_23_P112	0.001517	0.000184	3.62	TRIM32	tripartite motif containing 32	Upregulated
A_32_P717	0.000483	5.44E-06	3.61	FKBP4	FK506 binding protein 4, 59kDa	Upregulated
A_33_P329	0.000521	0.0000106	3.61	ST8SIA4	ST8 alpha-N-acetyl-neuraminide alpha-2,8-sialyltransferase 4	Upregulated
A_21_P001	0.000578	0.0000191	3.61	XLOC_l2_015213		Upregulated
A_21_P001	0.00096	0.0000717	3.61	MTHFD1L	methylenetetrahydrofolate dehydrogenase (NADP+ dependent) 1-like	Upregulated
A_23_P771	0.001297	0.000138	3.61	SORD	sorbitol dehydrogenase	Upregulated
A_22_P000	0.001453	0.000171	3.61	lnc-HMHB1-3	lnc-HMHB1-3:1	Upregulated
A_33_P334	0.000945	0.0000689	3.6	TBL1X	transducin (beta)-like 1X-linked	Upregulated
A_32_P118	0.001323	0.000143	3.6	C12orf65	chromosome 12 open reading frame 65	Upregulated
A_33_P335	0.001943	0.000284	3.6	SMARCAD1	SWI/SNF-related, matrix-associated actin-dependent regulator of chroma	Upregulated
A_23_P206	0.000483	4.05E-06	3.59	MT1E	metallothionein 1E	Upregulated
A_19_P008	0.000848	0.0000544	3.59	XLOC_l2_014579		Upregulated
A_22_P000	0.000483	4.11E-06	3.58	PPP3CB-AS1	PPP3CB antisense RNA 1 (head to head)	Upregulated
A_22_P000	0.000515	9.54E-06	3.58	lnc-PPP4R1-5	lnc-PPP4R1-5:1	Upregulated
A_22_P000	0.001262	0.000132	3.58	lnc-OIT3-1	lnc-OIT3-1:1	Upregulated
A_24_P468	0.001475	0.000175	3.58	RBBP4	retinoblastoma binding protein 4	Upregulated

A_23_P109	0.00062	0.0000241	3.57	THOC5	THO complex 5	Upregulated
A_33_P331	0.000945	0.0000691	3.57	MYB	v-myb avian myeloblastosis viral oncogene homolog	Upregulated
A_23_P358	0.001046	0.0000878	3.57	POU5F2	POU domain class 5, transcription factor 2	Upregulated
A_33_P332	0.002989	0.000564	3.57	RNF216	ring finger protein 216	Upregulated
A_33_P329	0.000483	4.23E-06	3.56	KCTD9	potassium channel tetramerization domain containing 9	Upregulated
A_23_P778	0.000601	0.0000218	3.56	TMEM88	transmembrane protein 88	Upregulated
A_23_P112	0.001386	0.000155	3.56	GNG10	guanine nucleotide binding protein (G protein), gamma 10	Upregulated
A_23_P746	0.002464	0.000419	3.56	G0S2	G0/G1 switch 2	Upregulated
A_23_P152	0.000483	4.95E-06	3.55	SNRNP25	small nuclear ribonucleoprotein 25kDa (U11/U12)	Upregulated
A_33_P333	0.000483	5.49E-06	3.55	MTMR10	myotubularin related protein 10	Upregulated
A_23_P203	0.00056	0.0000161	3.55	ACER3	alkaline ceramidase 3	Upregulated
A_23_P654	0.000707	0.0000343	3.55	HAUS4	HAUS augmin-like complex, subunit 4	Upregulated
A_23_P210	0.00226	0.00037	3.55	EPAS1	endothelial PAS domain protein 1	Upregulated
A_23_P161	0.000483	4.01E-06	3.54	ASAH2	N-acylsphingosine amidohydrolase (non-lysosomal ceramidase) 2	Upregulated
A_23_P204	0.000483	4.08E-06	3.54	NOP2	NOP2 nucleolar protein	Upregulated
A_23_P337	0.000526	0.0000116	3.54	PPFIBP1	PTPRF interacting protein, binding protein 1 (liprin beta 1)	Upregulated
A_33_P321	0.000957	0.0000709	3.54	PDCD4	programmed cell death 4 (neoplastic transformation inhibitor)	Upregulated
A_22_P000	0.00104	0.0000867	3.54	lnc-WIBG-1	lnc-WIBG-1:1	Upregulated
A_33_P321	0.001133	0.000105	3.54	MID1IP1	MID1 interacting protein 1	Upregulated
A_22_P000	0.0005	8.04E-06	3.53	lnc-LRMP-3	lnc-LRMP-3:1	Upregulated
A_24_P162	0.001244	0.000128	3.53	LINC00665	long intergenic non-protein coding RNA 665	Upregulated
A_23_P213	0.00126	0.000131	3.53	FGF1	fibroblast growth factor 1 (acidic)	Upregulated
A_22_P000	0.004269	0.000963	3.53	lnc-NDST1-1	lnc-NDST1-1:1	Upregulated
A_33_P328	0.000498	7.84E-06	3.51	LENG8	leukocyte receptor cluster (LRC) member 8	Upregulated
A_23_P108	0.000584	0.0000199	3.51	ANKLE2	ankyrin repeat and LEM domain containing 2	Upregulated
A_24_P135	0.000605	0.0000227	3.51	KCTD9	potassium channel tetramerization domain containing 9	Upregulated
A_24_P295	0.000788	0.0000457	3.51	HBS1L	HBS1-like translational GTPase	Upregulated
A_33_P331	0.000846	0.0000538	3.51	CCDC34	coiled-coil domain containing 34	Upregulated
A_23_P257	0.000994	0.0000775	3.51	PEX13	peroxisomal biogenesis factor 13	Upregulated
A_21_P000	0.001068	0.0000932	3.51	lnc-RNF144A-	lnc-RNF144A-3:1	Upregulated
A_23_P167	0.000486	7.07E-06	3.5	TMA16	translation machinery associated 16 homolog (S. cerevisiae)	Upregulated
A_23_P156	0.000515	9.26E-06	3.5	ZNF622	zinc finger protein 622	Upregulated
A_21_P000	0.000515	9.81E-06	3.5	lnc-ARRDC3-	lnc-ARRDC3-1:1	Upregulated
A_24_P415	0.000707	0.0000345	3.5	HPS1	Hermansky-Pudlak syndrome 1	Upregulated
A_33_P338	0.003543	0.000732	3.5	CYB5D1	cytochrome b5 domain containing 1	Upregulated
A_23_P930	0.000525	0.0000112	3.49	APH1A	APH1A gamma secretase subunit	Upregulated
A_33_P341	0.000575	0.0000178	3.49	GK	glycerol kinase	Upregulated

A_24_P354	0.000577	0.0000184	3.49	TAGAP	T-cell activation RhoGTPase activating protein	Upregulated
A_33_P335	0.000695	0.0000315	3.49	SMC2	structural maintenance of chromosomes 2	Upregulated
A_21_P001	0.001295	0.000138	3.49	TBC1D3P1-D	TBC1D3P1-DHX40P1 readthrough transcribed pseudogene	Upregulated
A_23_P941	0.000726	0.0000378	3.48	NEIL2	nei endonuclease VIII-like 2 (E. coli)	Upregulated
A_24_P135	0.001021	0.0000832	3.48	UEVLD	UEV and lactate/malate dehyrogenase domains	Upregulated
A_21_P001	0.003094	0.000595	3.48	LINC01237	long intergenic non-protein coding RNA 1237	Upregulated
A_33_P332	0.000483	6.22E-06	3.47	MOK	MOK protein kinase	Upregulated
A_23_P324	0.000502	0.0000084	3.47	SYNJ1	synaptojanin 1	Upregulated
A_24_P213	0.00056	0.0000164	3.47	MAN1A2	mannosidase, alpha, class 1A, member 2	Upregulated
A_23_P259	0.000641	0.0000259	3.47	TTK	TTK protein kinase	Upregulated
A_24_P393	0.000709	0.0000349	3.47	PACS2	phosphofurin acidic cluster sorting protein 2	Upregulated
A_23_P420	0.001265	0.000132	3.47	ORC3	origin recognition complex, subunit 3	Upregulated
A_23_P134	0.00239	0.000401	3.47	SLC18B1	solute carrier family 18, subfamily B, member 1	Upregulated
A_23_P132	0.000483	4.82E-06	3.46	ACAD9	acyl-CoA dehydrogenase family, member 9	Upregulated
A_23_P256	0.000526	0.0000123	3.46	AKIRIN1	akirin 1	Upregulated
A_23_P210	0.000483	6.32E-06	3.45	BCL2L1	BCL2-like 1	Upregulated
A_22_P000	0.00063	0.000025	3.45	LOC1027249	uncharacterized LOC102724901	Upregulated
A_23_P194	0.000661	0.0000277	3.45	STK19	serine/threonine kinase 19	Upregulated
A_22_P000	0.001031	0.0000852	3.45	lnc-DDX51-2	lnc-DDX51-2:1	Upregulated
A_23_P268	0.001203	0.00012	3.45	ARHGAP44	Rho GTPase activating protein 44	Upregulated
A_23_P800	0.001901	0.000274	3.45	PROCR	protein C receptor, endothelial	Upregulated
A_23_P115	0.000506	8.65E-06	3.44	TMEM125	transmembrane protein 125	Upregulated
A_22_P000	0.000589	0.0000205	3.44	lnc-WDR77-1	lnc-WDR77-1:1	Upregulated
A_23_P422	0.000887	0.0000597	3.44	SUV39H1	suppressor of variegation 3-9 homolog 1 (Drosophila)	Upregulated
A_23_P363	0.000571	0.0000173	3.43	C1RL	complement component 1, r subcomponent-like	Upregulated
A_23_P589	0.001256	0.00013	3.43	TBC1D22B	TBC1 domain family, member 22B	Upregulated
A_22_P000	0.001398	0.000158	3.43	MIR612	microRNA 612	Upregulated
A_33_P353	0.00399	0.000871	3.43	C5orf42	chromosome 5 open reading frame 42	Upregulated
A_21_P001	0.000634	0.0000252	3.42	NF1	neurofibromin 1	Upregulated
A_23_P202	0.001267	0.000133	3.42	CXCL12	chemokine (C-X-C motif) ligand 12	Upregulated
A_23_P153	0.000711	0.0000356	3.41	ZNF624	zinc finger protein 624	Upregulated
A_23_P110	0.001358	0.00015	3.41	CSF1R	colony stimulating factor 1 receptor	Upregulated
A_23_P321	0.002858	0.000527	3.41	DTWD1	DTW domain containing 1	Upregulated
A_33_P334	0.003909	0.000845	3.41	ANKRD20A12	ankyrin repeat domain 20 family, member A12, pseudogene	Upregulated
A_23_P559	0.005305	0.00133	3.41	PRR12	proline rich 12	Upregulated
A_22_P000	0.00077	0.0000432	3.4	lnc-ATP2A1-1	lnc-ATP2A1-1:1	Upregulated
A_33_P366	0.000814	0.0000501	3.4	AHCTF1	AT hook containing transcription factor 1	Upregulated

A_23_P170	0.00056	0.0000165	3.39	TMEM57	transmembrane protein 57	Upregulated
A_23_P354	0.000906	0.0000624	3.39	CISD1	CDGSH iron sulfur domain 1	Upregulated
A_33_P334	0.001261	0.000131	3.39	SIRPG	signal-regulatory protein gamma	Upregulated
A_21_P001	0.000513	9.01E-06	3.38	XLOC_l2_013189		Upregulated
A_32_P755	0.00056	0.0000159	3.38	TAMM41	TAM41, mitochondrial translocator assembly and maintenance protein, l	Upregulated
A_24_P280	0.001171	0.000113	3.38	LOC1001291	THAP domain containing, apoptosis associated protein 3 pseudogene	Upregulated
A_22_P000	0.000483	5.48E-06	3.37	lnc-TCIRG1-1	lnc-TCIRG1-1:1	Upregulated
A_24_P206	0.000486	7.04E-06	3.37	ZNF746	zinc finger protein 746	Upregulated
A_23_P256	0.000675	0.0000292	3.37	RNF219	ring finger protein 219	Upregulated
A_23_P356	0.000697	0.000032	3.37	RBM17	RNA binding motif protein 17	Upregulated
A_23_P566	0.000965	0.0000732	3.37	INO80B	INO80 complex subunit B	Upregulated
A_24_P181	0.0015	0.000181	3.37	B3GNTL1	UDP-GlcNAc:betaGal beta-1,3-N-acetylglucosaminyltransferase-like 1	Upregulated
A_22_P000	0.000502	8.38E-06	3.36	lnc-MDH1B-2	lnc-MDH1B-2:1	Upregulated
A_23_P121	0.000526	0.0000123	3.36	TWF2	twinfilin actin-binding protein 2	Upregulated
A_22_P000	0.0017	0.000226	3.36	lnc-SPSB4-1	lnc-SPSB4-1:1	Upregulated
A_21_P000	0.00433	0.000985	3.36	LOC1019275	uncharacterized LOC101927598	Upregulated
A_23_P428	0.006498	0.00178	3.36	EZH1	enhancer of zeste 1 polycomb repressive complex 2 subunit	Upregulated
A_23_P204	0.000486	7.04E-06	3.35	RAB35	RAB35, member RAS oncogene family	Upregulated
A_21_P000	0.000525	0.0000112	3.35	lnc-KIAA1919	lnc-KIAA1919-1:1	Upregulated
A_23_P202	0.000603	0.0000222	3.35	MADD	MAP-kinase activating death domain	Upregulated
A_24_P269	0.000693	0.000031	3.35	PLEKHA1	pleckstrin homology domain containing, family A (phosphoinositide bindi	Upregulated
A_33_P324	0.000718	0.0000366	3.35	C10orf128	chromosome 10 open reading frame 128	Upregulated
A_33_P326	0.001055	0.0000896	3.35	DYNLT1	dynein, light chain, Tctex-type 1	Upregulated
A_19_P008	0.001398	0.000158	3.35	LINC01355	long intergenic non-protein coding RNA 1355	Upregulated
A_21_P000	0.001812	0.000252	3.35	lnc-GATA3-3	lnc-GATA3-3:1	Upregulated
A_33_P681	0.003817	0.000816	3.35	PITRM1-AS1	PITRM1 antisense RNA 1	Upregulated
A_23_P365	0.000707	0.000034	3.34	ZNF783	zinc finger family member 783	Upregulated
A_22_P000	0.000483	0.0000061	3.33	lnc-C1QTNF5	lnc-C1QTNF5-1:1	Upregulated
A_23_P253	0.000526	0.0000121	3.33	GPR171	G protein-coupled receptor 171	Upregulated
A_33_P339	0.000664	0.0000281	3.33	C14orf28	chromosome 14 open reading frame 28	Upregulated
A_22_P000	0.002167	0.000346	3.33	lnc-CEP170-5	lnc-CEP170-5:1	Upregulated
A_22_P000	0.003766	0.0008	3.33	lnc-C18orf32-	lnc-C18orf32-1:1	Upregulated
A_23_P422	0.000521	0.0000103	3.32	PPIC	peptidylprolyl isomerase C (cyclophilin C)	Upregulated
A_33_P322	0.000597	0.0000214	3.32	ZNF197	zinc finger protein 197	Upregulated
A_24_P252	0.000814	0.0000499	3.32	CLP1	cleavage and polyadenylation factor I subunit 1	Upregulated
A_21_P000	0.000831	0.000052	3.32	lnc-C2orf27B-	lnc-C2orf27B-3:2	Upregulated
A_24_P137	0.001304	0.00014	3.32	GFM2	G elongation factor, mitochondrial 2	Upregulated

A_23_P334	0.00134	0.000146	3.32	TBC1D31	TBC1 domain family, member 31	Upregulated
A_24_P465	0.003715	0.000784	3.32	ZNRD1	zinc ribbon domain containing 1	Upregulated
A_32_P221	0.004205	0.00094	3.32	RABL3	RAB, member of RAS oncogene family-like 3	Upregulated
A_33_P339	0.004411	0.00101	3.32	PIP4K2A	phosphatidylinositol-5-phosphate 4-kinase, type II, alpha	Upregulated
A_22_P000	0.000483	6.31E-06	3.31	lnc-DCLRE1B	lnc-DCLRE1B-2:1	Upregulated
A_23_P963	0.000715	0.0000362	3.31	ERCC6L	excision repair cross-complementation group 6-like	Upregulated
A_22_P000	0.001291	0.000137	3.31	lnc-CHRNA3-2	lnc-CHRNA3-2:1	Upregulated
A_33_P331	0.005707	0.00147	3.31	TRAPPC10	trafficking protein particle complex 10	Upregulated
A_19_P003	0.000521	0.0000106	3.3	LINC01197	long intergenic non-protein coding RNA 1197	Upregulated
A_33_P332	0.000532	0.0000132	3.3	BACH1	BTB and CNC homology 1, basic leucine zipper transcription factor 1	Upregulated
A_23_P391	0.001139	0.000107	3.3	MANEAL	mannosidase, endo-alpha-like	Upregulated
A_23_P828	0.002572	0.000449	3.3	OSGIN2	oxidative stress induced growth inhibitor family member 2	Upregulated
A_21_P001	0.005795	0.00151	3.3	LOC1019299	uncharacterized LOC101929917	Upregulated
A_33_P331	0.000526	0.0000123	3.29	MOB1B	MOB kinase activator 1B	Upregulated
A_23_P154	0.000885	0.0000594	3.29	PTRH2	peptidyl-tRNA hydrolase 2	Upregulated
A_33_P331	0.000483	5.53E-06	3.28	MT1HL1	metallothionein 1H-like 1	Upregulated
A_23_P427	0.000498	7.94E-06	3.28	MT1L	metallothionein 1L (gene/pseudogene)	Upregulated
A_23_P173	0.000521	0.0000107	3.28	MRGBP	MRG/MORF4L binding protein	Upregulated
A_23_P900	0.000578	0.0000189	3.28	GPR108	G protein-coupled receptor 108	Upregulated
A_23_P555	0.000902	0.0000615	3.28	RNMT	RNA (guanine-7-) methyltransferase	Upregulated
A_33_P328	0.001047	0.0000882	3.28	SORBS1	sorbin and SH3 domain containing 1	Upregulated
A_23_P120	0.000483	5.91E-06	3.27	MCM3AP	minichromosome maintenance complex component 3 associated protein	Upregulated
A_33_P323	0.000526	0.0000121	3.27	LARP1B	La ribonucleoprotein domain family, member 1B	Upregulated
A_23_P141	0.000728	0.000038	3.27	NCOR1	nuclear receptor corepressor 1	Upregulated
A_23_P203	0.00089	0.0000602	3.27	NEU3	sialidase 3 (membrane sialidase)	Upregulated
A_33_P327	0.000944	0.0000687	3.27	NIPSNAP3B	nipsnap homolog 3B (C. elegans)	Upregulated
A_23_P241	0.000976	0.0000751	3.27	CCNJ	cyclin J	Upregulated
A_23_P259	0.000483	6.42E-06	3.26	LGMN	legumain	Upregulated
A_23_P134	0.000506	8.66E-06	3.26	GPNMB	glycoprotein (transmembrane) nmb	Upregulated
A_32_P791	0.000525	0.0000115	3.26	ANKRD20A12	ankyrin repeat domain 20 family, member A12, pseudogene	Upregulated
A_33_P340	0.000534	0.0000136	3.26	C9orf85	chromosome 9 open reading frame 85	Upregulated
A_23_P371	0.001043	0.0000873	3.26	DNM3	dynamin 3	Upregulated
A_23_P130	0.00122	0.000123	3.26	CEP89	centrosomal protein 89kDa	Upregulated
A_33_P323	0.001603	0.000202	3.26	PIN4	protein (peptidylprolyl cis/trans isomerase) NIMA-interacting, 4 (parvulin)	Upregulated
A_33_P341	0.000515	9.93E-06	3.25	CLCN5	chloride channel, voltage-sensitive 5	Upregulated
A_22_P000	0.000521	0.0000105	3.25	LOC1009964	uncharacterized LOC100996437	Upregulated
A_24_P348	0.000725	0.0000376	3.25	PLEKHA7	pleckstrin homology domain containing, family A member 7	Upregulated

A_23_P111	0.000779	0.0000442	3.25	MRPL32	mitochondrial ribosomal protein L32	Upregulated
A_23_P349	0.001398	0.000158	3.25	DIEXF	digestive organ expansion factor homolog (zebrafish)	Upregulated
A_33_P388	0.000498	0.0000078	3.24	ZCCHC10	zinc finger, CCHC domain containing 10	Upregulated
A_33_P341	0.000599	0.0000215	3.24	ZNF235	zinc finger protein 235	Upregulated
A_33_P327	0.000662	0.0000279	3.24	SYT6	synaptotagmin VI	Upregulated
A_33_P324	0.001356	0.000149	3.24	FOXO3	forkhead box O3	Upregulated
A_21_P000	0.008047	0.00238	3.24	BTN2A2	butyrophilin, subfamily 2, member A2	Upregulated
A_24_P401	0.000486	6.96E-06	3.23	TPH2	tryptophan hydroxylase 2	Upregulated
A_23_P160	0.000486	6.99E-06	3.23	LAMC2	laminin, gamma 2	Upregulated
A_24_P126	0.000939	0.0000679	3.23	CAV1	caveolin 1, caveolae protein, 22kDa	Upregulated
A_23_P416	0.000975	0.0000749	3.23	MS4A6E	membrane-spanning 4-domains, subfamily A, member 6E	Upregulated
A_23_P807	0.00125	0.000129	3.23	DIRC2	disrupted in renal carcinoma 2	Upregulated
A_33_P333	0.006885	0.00192	3.23	SNAPC5	small nuclear RNA activating complex, polypeptide 5, 19kDa	Upregulated
A_33_P333	0.000483	5.67E-06	3.22	OR7E37P	olfactory receptor, family 7, subfamily E, member 37 pseudogene	Upregulated
A_24_P350	0.000483	6.29E-06	3.22	PDPR	pyruvate dehydrogenase phosphatase regulatory subunit	Upregulated
A_23_P334	0.000609	0.000023	3.22	NRG4	neuregulin 4	Upregulated
A_23_P944	0.002726	0.00049	3.22	MELK	maternal embryonic leucine zipper kinase	Upregulated
A_23_P349	0.00071	0.0000354	3.21	FBXO41	F-box protein 41	Upregulated
A_33_P332	0.003684	0.000774	3.21	TNFRSF10D	tumor necrosis factor receptor superfamily, member 10d, decoy with trun	Upregulated
A_24_P143	0.004634	0.00109	3.21	SIRPB1	signal-regulatory protein beta 1	Upregulated
A_33_P747	0.000513	8.99E-06	3.2	THUMPD3-AS	THUMPD3 antisense RNA 1	Upregulated
A_19_P003	0.000575	0.0000176	3.2	LOC1019297	uncharacterized LOC101929709	Upregulated
A_33_P322	0.000675	0.0000293	3.2	OXER1	oxoeicosanoid (OXE) receptor 1	Upregulated
A_21_P001	0.000879	0.0000585	3.2	SNHG21	small nucleolar RNA host gene 21 (non-protein coding)	Upregulated
A_22_P000	0.00096	0.0000717	3.2	lnc-ZNF814-1	lnc-ZNF814-1:1	Upregulated
A_22_P000	0.001324	0.000143	3.2	lnc-PLEKHA3	lnc-PLEKHA3-2:2	Upregulated
A_21_P001	0.002008	0.000303	3.2	ANKRD20A1	ankyrin repeat domain 20 family, member A11, pseudogene	Upregulated
A_33_P338	0.004831	0.00115	3.2	LRRK2	leucine-rich repeat kinase 2	Upregulated
A_33_P327	0.000486	7.17E-06	3.19	RABEP1	rabaptin, RAB GTPase binding effector protein 1	Upregulated
A_32_P723	0.000494	7.52E-06	3.19	PRKAR1B	protein kinase, cAMP-dependent, regulatory, type I, beta	Upregulated
A_23_P317	0.001198	0.000118	3.19	AKAP10	A kinase (PRKA) anchor protein 10	Upregulated
A_33_P339	0.003012	0.000572	3.19	XLOC_l2_001559		Upregulated
A_33_P335	0.003511	0.00072	3.19	CSF1	colony stimulating factor 1 (macrophage)	Upregulated
A_23_P386	0.000515	9.27E-06	3.18	PPID	peptidylprolyl isomerase D	Upregulated
A_24_P244	0.000526	0.0000126	3.18	RNF41	ring finger protein 41, E3 ubiquitin protein ligase	Upregulated
A_21_P000	0.002944	0.00055	3.18	lnc-FRG1-5	lnc-FRG1-5:3	Upregulated
A_33_P330	0.003191	0.000627	3.18	USP47	ubiquitin specific peptidase 47	Upregulated

A_22_P000	0.003359	0.000677	3.18	lnc-C3orf23-2	lnc-C3orf23-2:1	Upregulated
A_23_P116	0.000527	0.0000129	3.17	C12orf10	chromosome 12 open reading frame 10	Upregulated
A_23_P112	0.001245	0.000128	3.17	NKRF	NFKB repressing factor	Upregulated
A_23_P354	0.000515	9.57E-06	3.16	PCDHGA8	protocadherin gamma subfamily A, 8	Upregulated
A_32_P445	0.000515	9.82E-06	3.16	LDHA	lactate dehydrogenase A	Upregulated
A_32_P403	0.00056	0.0000163	3.16	LOC389906	zinc finger protein 839 pseudogene	Upregulated
A_24_P334	0.00056	0.0000165	3.16	FN1	fibronectin 1	Upregulated
A_33_P327	0.000697	0.0000319	3.16	THAP3	THAP domain containing, apoptosis associated protein 3	Upregulated
A_23_P501	0.00283	0.000518	3.16	NDC80	NDC80 kinetochore complex component	Upregulated
A_33_P334	0.000486	7.17E-06	3.15	NUBPL	nucleotide binding protein-like	Upregulated
A_23_P236	0.00056	0.0000157	3.15	AMY1C	amylase, alpha 1C (salivary)	Upregulated
A_23_P689	0.001266	0.000133	3.15	MICALL1	MICAL-like 1	Upregulated
A_23_P141	0.001453	0.000171	3.15	UBE2M	ubiquitin-conjugating enzyme E2M	Upregulated
A_23_P880	0.001885	0.00027	3.15	LHFP	lipoma HMGIC fusion partner	Upregulated
A_33_P324	0.004995	0.00121	3.15	SLC4A5	solute carrier family 4 (sodium bicarbonate cotransporter), member 5	Upregulated
A_23_P403	0.005346	0.00134	3.15	JMJD7-PLA2(	JMJD7-PLA2G4B readthrough	Upregulated
A_32_P806	0.000639	0.0000256	3.14	ARL4A	ADP-ribosylation factor-like 4A	Upregulated
A_23_P316	0.000695	0.0000315	3.14	BUD31	BUD31 homolog (S. cerevisiae)	Upregulated
A_32_P927	0.000814	0.00005	3.14	STIP1	stress-induced phosphoprotein 1	Upregulated
A_24_P140	0.00104	0.0000866	3.14	LRRTM2	leucine rich repeat transmembrane neuronal 2	Upregulated
A_22_P000	0.001321	0.000142	3.14	TMEM161B-A	TMEM161B antisense RNA 1	Upregulated
A_22_P000	0.001753	0.000237	3.14	lnc-C2orf69-4	lnc-C2orf69-4:1	Upregulated
A_33_P350	0.001936	0.000283	3.14	LOC1027235	uncharacterized LOC102723564	Upregulated
A_23_P275	0.007746	0.00227	3.14	MCOLN1	mucolipin 1	Upregulated
A_24_P379	0.000526	0.0000124	3.13	MXD1	MAX dimerization protein 1	Upregulated
A_19_P003	0.000526	0.0000127	3.13	UBA6-AS1	UBA6 antisense RNA 1 (head to head)	Upregulated
A_23_P162	0.000535	0.0000137	3.13	ARNTL	aryl hydrocarbon receptor nuclear translocator-like	Upregulated
A_23_P151	0.000578	0.0000186	3.13	IL32	interleukin 32	Upregulated
A_23_P347	0.000635	0.0000254	3.13	SGPP1	sphingosine-1-phosphate phosphatase 1	Upregulated
A_22_P000	0.000786	0.0000454	3.13	LOC1027249	uncharacterized LOC102724915	Upregulated
A_33_P332	0.000825	0.0000513	3.13	ATXN1	ataxin 1	Upregulated
A_22_P000	0.00093	0.0000662	3.13	lnc-C1QBP-1	lnc-C1QBP-1:1	Upregulated
A_23_P107	0.003791	0.000807	3.13	FUT1	fucosyltransferase 1 (galactoside 2-alpha-L-fucosyltransferase, H blood	Upregulated
A_23_P166	0.000498	7.93E-06	3.12	PFDN4	prefoldin subunit 4	Upregulated
A_23_P116	0.000502	8.31E-06	3.12	USP35	ubiquitin specific peptidase 35	Upregulated
A_23_P113	0.000515	9.51E-06	3.12	EXOC1	exocyst complex component 1	Upregulated
A_23_P605	0.000525	0.0000115	3.12	DNAJC7	DnaJ (Hsp40) homolog, subfamily C, member 7	Upregulated

A_33_P335	0.000707	0.0000345	3.12	LOC1019291	uncharacterized LOC101929115	Upregulated
A_32_P961	0.00076	0.0000417	3.12	DPY19L1	dpy-19-like 1 (C. elegans)	Upregulated
A_23_P747	0.000869	0.0000575	3.12	C1orf54	chromosome 1 open reading frame 54	Upregulated
A_23_P309	0.001397	0.000157	3.12	N4BP2	NEDD4 binding protein 2	Upregulated
A_23_P136	0.001525	0.000186	3.12	PIGG	phosphatidylinositol glycan anchor biosynthesis, class G	Upregulated
A_23_P136	0.001668	0.000218	3.12	RPGR	retinitis pigmentosa GTPase regulator	Upregulated
A_21_P000	0.002002	0.000301	3.12	lnc-GRB7-1	lnc-GRB7-1:1	Upregulated
A_24_P290	0.00579	0.0015	3.12	DTX1	deltex 1, E3 ubiquitin ligase	Upregulated
A_24_P353	0.010673	0.00347	3.12	GALNT2	polypeptide N-acetylgalactosaminyltransferase 2	Upregulated
A_19_P003	0.000541	0.0000144	3.11	TUG1	taurine up-regulated 1 (non-protein coding)	Upregulated
A_33_P338	0.00056	0.0000164	3.11	WHSC1L1	Wolf-Hirschhorn syndrome candidate 1-like 1	Upregulated
A_23_P135	0.000578	0.0000191	3.11	PRSS3	protease, serine, 3	Upregulated
A_22_P000	0.000743	0.0000399	3.11	lnc-ANKUB1-1	lnc-ANKUB1-1:1	Upregulated
A_33_P323	0.000807	0.0000487	3.11	ZNF776	zinc finger protein 776	Upregulated
A_21_P000	0.000929	0.0000658	3.11	lnc-UBE2B-1	lnc-UBE2B-1:1	Upregulated
A_23_P144	0.001108	0.000101	3.11	EEFSEC	eukaryotic elongation factor, selenocysteine-tRNA-specific	Upregulated
A_24_P241	0.00152	0.000184	3.11	CABP2	calcium binding protein 2	Upregulated
A_24_P330	0.001889	0.000271	3.11	FRMD6	FERM domain containing 6	Upregulated
A_24_P217	0.003817	0.000816	3.11	TBRG1	transforming growth factor beta regulator 1	Upregulated
A_33_P329	0.000486	7.07E-06	3.1	AKIRIN1	akirin 1	Upregulated
A_23_P721	0.000709	0.0000348	3.1	SMPDL3A	sphingomyelin phosphodiesterase, acid-like 3A	Upregulated
A_23_P811	0.000711	0.0000355	3.1	ZBTB9	zinc finger and BTB domain containing 9	Upregulated
A_23_P350	0.000781	0.0000445	3.1	ZDHHC23	zinc finger, DHHC-type containing 23	Upregulated
A_33_P681	0.000991	0.000077	3.1	PCBP1-AS1	PCBP1 antisense RNA 1	Upregulated
A_33_P341	0.000498	7.77E-06	3.09	SLC17A5	solute carrier family 17 (acidic sugar transporter), member 5	Upregulated
A_23_P211	0.000515	9.74E-06	3.09	POFUT2	protein O-fucosyltransferase 2	Upregulated
A_23_P151	0.000532	0.0000134	3.09	MT1F	metallothionein 1F	Upregulated
A_24_P378	0.000785	0.0000452	3.09	DHRSX	dehydrogenase/reductase (SDR family) X-linked	Upregulated
A_22_P000	0.001949	0.000286	3.09	lnc-AC02106(	lnc-AC021066.1-1:1	Upregulated
A_33_P324	0.000498	0.0000079	3.08	PDE8A	phosphodiesterase 8A	Upregulated
A_22_P000	0.000526	0.0000123	3.08	lnc-GYG2-1	lnc-GYG2-1:1	Upregulated
A_23_P801	0.00056	0.0000164	3.08	WRB	tryptophan rich basic protein	Upregulated
A_22_P000	0.000695	0.0000313	3.08	LOC1019271	uncharacterized LOC101927124	Upregulated
A_23_P584	0.000942	0.0000684	3.08	FASTKD3	FAST kinase domains 3	Upregulated
A_23_P105	0.00126	0.000131	3.08	ME3	malic enzyme 3, NADP(+)-dependent, mitochondrial	Upregulated
A_33_P340	0.001626	0.000208	3.08	ZNF776	zinc finger protein 776	Upregulated
A_24_P887	0.001843	0.00026	3.08	LOXL3	lysyl oxidase-like 3	Upregulated

A_23_P645	0.005787	0.0015	3.08	PPME1	protein phosphatase methylesterase 1	Upregulated
A_24_P944	0.008145	0.00242	3.08	LRP2BP	LRP2 binding protein	Upregulated
A_22_P000	0.000486	6.92E-06	3.07	LOC1019270	uncharacterized LOC101927060	Upregulated
A_23_P345	0.000526	0.0000117	3.07	FASTKD2	FAST kinase domains 2	Upregulated
A_22_P000	0.00071	0.0000353	3.07	LOC1019270	uncharacterized LOC101927069	Upregulated
A_22_P000	0.001469	0.000173	3.07	lnc-UTS2D-1	lnc-UTS2D-1:1	Upregulated
A_23_P157	0.003147	0.000612	3.07	TNC	tenascin C	Upregulated
A_33_P332	0.005444	0.00138	3.07	PRO0628	uncharacterized LOC29053	Upregulated
A_33_P325	0.005592	0.00143	3.07	TIRAP	toll-interleukin 1 receptor (TIR) domain containing adaptor protein	Upregulated
A_22_P000	0.002171	0.000347	3.06	lnc-LDHD-1	lnc-LDHD-1:1	Upregulated
A_21_P001	0.002413	0.000407	3.06	XLOC_l2_013646		Upregulated
A_23_P147	0.005554	0.00142	3.06	TOR4A	torsin family 4, member A	Upregulated
A_33_P329	0.000488	7.28E-06	3.05	SBNO1	strawberry notch homolog 1 (Drosophila)	Upregulated
A_33_P335	0.000515	9.79E-06	3.05	PAGR1	PAXIP1 associated glutamate-rich protein 1	Upregulated
A_23_P321	0.001007	0.0000812	3.05	C9orf9	chromosome 9 open reading frame 9	Upregulated
A_19_P003	0.002957	0.000555	3.05	LOC1005071	uncharacterized LOC100507165	Upregulated
A_21_P000	0.000537	0.000014	3.04	SLC9A7	solute carrier family 9, subfamily A (NHE7, cation proton antiporter 7), m	Upregulated
A_23_P629	0.000904	0.0000617	3.04	UBASH3A	ubiquitin associated and SH3 domain containing A	Upregulated
A_33_P331	0.001408	0.000161	3.04	MC1R	melanocortin 1 receptor (alpha melanocyte stimulating hormone recepto	Upregulated
A_33_P324	0.001965	0.000292	3.04	SCAMP1	secretory carrier membrane protein 1	Upregulated
A_23_P398	0.000563	0.0000167	3.03	PWWP2A	PWWP domain containing 2A	Upregulated
A_24_P529	0.00063	0.000025	3.03	BCAT1	branched chain amino-acid transaminase 1, cytosolic	Upregulated
A_23_P384	0.000728	0.0000381	3.03	TRAP1	TNF receptor-associated protein 1	Upregulated
A_24_P630	0.001046	0.0000878	3.03	DFNB59	deafness, autosomal recessive 59	Upregulated
A_21_P001	0.001154	0.00011	3.03	INAFM2	InaF-motif containing 2	Upregulated
A_23_P106	0.000513	0.000009	3.02	MT2A	metallothionein 2A	Upregulated
A_22_P000	0.000657	0.0000274	3.02	lnc-AP00089	lnc-AP000892.4.1-1:1	Upregulated
A_22_P000	0.000664	0.0000281	3.02	lnc-TAT-1	lnc-TAT-1:1	Upregulated
A_23_P620	0.000693	0.0000309	3.02	SCG5	secretogranin V (7B2 protein)	Upregulated
A_23_P669	0.001088	0.0000968	3.02	GAREM	GRB2 associated, regulator of MAPK1	Upregulated
A_22_P000	0.001767	0.000241	3.02	LINC01032	long intergenic non-protein coding RNA 1032	Upregulated
A_23_P145	0.009439	0.00295	3.02	BYSL	bystin-like	Upregulated
A_23_P414	0.000515	9.58E-06	3.01	TTC17	tetratricopeptide repeat domain 17	Upregulated
A_33_P321	0.000605	0.0000227	3.01	AFMID	arylformamidase	Upregulated
A_24_P435	0.000928	0.0000656	3.01	MAD2L1BP	MAD2L1 binding protein	Upregulated
A_21_P001	0.001399	0.000159	3.01	XLOC_l2_010511		Upregulated
A_23_P169	0.00421	0.000943	3.01	HTR2B	5-hydroxytryptamine (serotonin) receptor 2B, G protein-coupled	Upregulated

A_33_P323	0.000502	8.46E-06	3	MCOLN3	mucolipin 3	Upregulated
A_22_P000	0.000525	0.0000113	3	VPS11	vacuolar protein sorting 11 homolog (S. cerevisiae)	Upregulated
A_22_P000	0.000526	0.0000122	3	lnc-FAM78A-1	lnc-FAM78A-1:1	Upregulated
A_22_P000	0.000792	0.0000464	3	HCG25	HLA complex group 25 (non-protein coding)	Upregulated
A_23_P354	0.000824	0.0000513	3	ZNF567	zinc finger protein 567	Upregulated
A_33_P681	0.000938	0.0000676	3	PCBP1-AS1	PCBP1 antisense RNA 1	Upregulated
A_32_P168	0.001414	0.000162	3	CASK	calcium/calmodulin-dependent serine protein kinase (MAGUK family)	Upregulated
A_23_P216	0.001519	0.000184	3	ZBTB5	zinc finger and BTB domain containing 5	Upregulated
A_23_P328	0.002631	0.000465	3	NEURL3	neuralized E3 ubiquitin protein ligase 3	Upregulated
A_24_P230	0.000498	7.81E-06	2.99	IL2RA	interleukin 2 receptor, alpha	Upregulated
A_23_P211	0.000566	0.0000171	2.99	PRMT2	protein arginine methyltransferase 2	Upregulated
A_24_P143	0.000621	0.0000243	2.99	SPIDR	scaffolding protein involved in DNA repair	Upregulated
A_23_P342	0.000736	0.0000387	2.99	STK35	serine/threonine kinase 35	Upregulated
A_22_P000	0.001439	0.000166	2.99	lnc-KLF6-2	lnc-KLF6-2:1	Upregulated
A_33_P321	0.001569	0.000195	2.99	LOC1009966	uncharacterized LOC100996662	Upregulated
A_24_P485	0.006425	0.00175	2.99	SIGLEC5	sialic acid binding Ig-like lectin 5	Upregulated
A_21_P001	0.000578	0.0000186	2.98	ZNF518A	zinc finger protein 518A	Upregulated
A_23_P139	0.000645	0.0000264	2.98	CDK8	cyclin-dependent kinase 8	Upregulated
A_33_P348	0.000783	0.000045	2.98	NLN	neurolysin (metallopeptidase M3 family)	Upregulated
A_33_P324	0.000792	0.0000461	2.98	APOA1BP	apolipoprotein A-I binding protein	Upregulated
A_23_P140	0.001256	0.00013	2.98	NGRN	neugrin, neurite outgrowth associated	Upregulated
A_24_P234	0.001715	0.00023	2.98	MXD4	MAX dimerization protein 4	Upregulated
A_33_P324	0.00227	0.000372	2.98	DLGAP1-AS5	DLGAP1 antisense RNA 5	Upregulated
A_33_P334	0.003733	0.00079	2.98	SYNPO2L	synaptopodin 2-like	Upregulated
A_21_P000	0.000701	0.0000327	2.97	lnc-SNTG2-4	lnc-SNTG2-4:4	Upregulated
A_23_P993	0.000798	0.0000472	2.97	TNFSF11	tumor necrosis factor (ligand) superfamily, member 11	Upregulated
A_23_P140	0.006528	0.00179	2.97	GOLGA8O	golgin A8 family, member O	Upregulated
A_24_P303	0.000712	0.0000358	2.96	HNRNPA0	heterogeneous nuclear ribonucleoprotein A0	Upregulated
A_22_P000	0.001821	0.000255	2.96	TRAF3IP2-AS	TRAF3IP2 antisense RNA 1	Upregulated
A_33_P326	0.002666	0.000474	2.96	ABAT	4-aminobutyrate aminotransferase	Upregulated
A_33_P327	0.005993	0.00158	2.96	ACOXL	acyl-CoA oxidase-like	Upregulated
A_23_P167	0.000515	9.55E-06	2.95	C4orf29	chromosome 4 open reading frame 29	Upregulated
A_23_P152	0.000521	0.0000107	2.95	ALOX12	arachidonate 12-lipoxygenase	Upregulated
A_24_P129	0.000795	0.0000469	2.95	TMEM245	transmembrane protein 245	Upregulated
A_23_P253	0.000814	0.00005	2.95	LRRC17	leucine rich repeat containing 17	Upregulated
A_21_P000	0.014383	0.00512	2.95	lnc-SHISA3-1	lnc-SHISA3-1:1	Upregulated
A_33_P321	0.000521	0.0000104	2.94	SPECC1L	sperm antigen with calponin homology and coiled-coil domains 1-like	Upregulated

A_24_P382	0.000522	0.0000108	2.94	POLR3K	polymerase (RNA) III (DNA directed) polypeptide K, 12.3 kDa	Upregulated
A_23_P128	0.000526	0.000012	2.94	GTPBP4	GTP binding protein 4	Upregulated
A_21_P001	0.000578	0.0000192	2.94	LOC1019293	uncharacterized LOC101929309	Upregulated
A_23_P208	0.000746	0.0000402	2.94	ZNF137P	zinc finger protein 137, pseudogene	Upregulated
A_33_P325	0.001037	0.0000862	2.94	CRB3	crumbs family member 3	Upregulated
A_33_P325	0.001596	0.0002	2.94	HAPLN3	hyaluronan and proteoglycan link protein 3	Upregulated
A_22_P000	0.004889	0.00118	2.94	ZNF518A	zinc finger protein 518A	Upregulated
A_23_P127	0.000515	9.98E-06	2.93	PRRG4	proline rich Gla (G-carboxyglutamic acid) 4 (transmembrane)	Upregulated
A_24_P371	0.000534	0.0000136	2.93	MRPL53	mitochondrial ribosomal protein L53	Upregulated
A_23_P772	0.000587	0.0000202	2.93	CRTC3	CREB regulated transcription coactivator 3	Upregulated
A_33_P352	0.000786	0.0000454	2.93	KDM2B	lysine (K)-specific demethylase 2B	Upregulated
A_22_P000	0.001023	0.000084	2.93	TOPORS-AS1	TOPORS antisense RNA 1	Upregulated
A_21_P000	0.001132	0.000105	2.93	SNORD33	small nucleolar RNA, C/D box 33	Upregulated
A_33_P333	0.002664	0.000473	2.93	LAMB3	laminin, beta 3	Upregulated
A_23_P386	0.003775	0.000802	2.93	FAM110A	family with sequence similarity 110, member A	Upregulated
A_23_P926	0.004499	0.00104	2.93	HELQ	helicase, POLQ-like	Upregulated
A_22_P000	0.000577	0.0000182	2.92	KIZ	kizuna centrosomal protein	Upregulated
A_33_P329	0.000619	0.0000238	2.92	CYP1B1	cytochrome P450, family 1, subfamily B, polypeptide 1	Upregulated
A_33_P323	0.000783	0.0000448	2.92	ANKRD50	ankyrin repeat domain 50	Upregulated
A_23_P309	0.000783	0.0000448	2.92	ZNF777	zinc finger protein 777	Upregulated
A_23_P322	0.000938	0.0000675	2.92	TOPORS	topoisomerase I binding, arginine/serine-rich, E3 ubiquitin protein ligase	Upregulated
A_23_P115	0.000938	0.0000675	2.92	TRIM11	tripartite motif containing 11	Upregulated
A_24_P339	0.002799	0.00051	2.92	PRSS21	protease, serine, 21 (testisin)	Upregulated
A_33_P322	0.005204	0.00129	2.92	ST7L	suppression of tumorigenicity 7 like	Upregulated
A_33_P340	0.006202	0.00166	2.92	ZNF555	zinc finger protein 555	Upregulated
A_33_P336	0.006567	0.0018	2.92	CORO1B	coronin, actin binding protein, 1B	Upregulated
A_33_P327	0.000515	9.66E-06	2.91	PPP1R26	protein phosphatase 1, regulatory subunit 26	Upregulated
A_33_P332	0.00056	0.0000161	2.91	ANO10	anoctamin 10	Upregulated
A_24_P327	0.000578	0.0000187	2.91	STIP1	stress-induced phosphoprotein 1	Upregulated
A_24_P191	0.000614	0.0000235	2.91	NAB1	NGFI-A binding protein 1 (EGR1 binding protein 1)	Upregulated
A_22_P000	0.001022	0.0000835	2.91	lnc-MRPS7-2	lnc-MRPS7-2:1	Upregulated
A_23_P120	0.001208	0.00012	2.91	MCFD2	multiple coagulation factor deficiency 2	Upregulated
A_23_P257	0.001437	0.000166	2.91	GEM	GTP binding protein overexpressed in skeletal muscle	Upregulated
A_24_P418	0.00176	0.000239	2.91	GPX7	glutathione peroxidase 7	Upregulated
A_33_P320	0.003433	0.000697	2.91	ZNF623	zinc finger protein 623	Upregulated
A_33_P328	0.000642	0.0000262	2.9	ZNF765	zinc finger protein 765	Upregulated
A_23_P485	0.000857	0.0000559	2.9	ARHGAP5	Rho GTPase activating protein 5	Upregulated

A_21_P000	0.001197	0.000118	2.9	lnc-ASMT-2	lnc-ASMT-2:1	Upregulated
A_22_P000	0.002043	0.000313	2.9	VPS11	vacuolar protein sorting 11 homolog (S. cerevisiae)	Upregulated
A_33_P332	0.008614	0.00262	2.9	LOC1001314	uncharacterized LOC100131432	Upregulated
A_23_P322	0.000526	0.0000127	2.89	PPAPDC1B	phosphatidic acid phosphatase type 2 domain containing 1B	Upregulated
A_23_P628	0.000537	0.0000141	2.89	YRDC	yrdC N(6)-threonylcarbamoyltransferase domain containing	Upregulated
A_33_P321	0.000559	0.0000155	2.89	TNIK	TRAF2 and NCK interacting kinase	Upregulated
A_33_P341	0.000585	0.0000202	2.89	ZNF592	zinc finger protein 592	Upregulated
A_23_P388	0.000596	0.000021	2.89	ZC3H12C	zinc finger CCCH-type containing 12C	Upregulated
A_23_P207	0.000658	0.0000276	2.89	WDR45B	WD repeat domain 45B	Upregulated
A_22_P000	0.001266	0.000132	2.89	LINC00847	long intergenic non-protein coding RNA 847	Upregulated
A_32_P139	0.003101	0.000599	2.89	ABL2	ABL proto-oncogene 2, non-receptor tyrosine kinase	Upregulated
A_23_P134	0.000515	9.55E-06	2.88	PLIN2	perilipin 2	Upregulated
A_22_P000	0.000541	0.0000144	2.88	RORA-AS1	RORA antisense RNA 1	Upregulated
A_22_P000	0.000695	0.0000316	2.88	lnc-LRP11-1	lnc-LRP11-1:1	Upregulated
A_22_P000	0.000731	0.0000383	2.88	ARHGAP31-A	ARHGAP31 antisense RNA 1	Upregulated
A_24_P119	0.000731	0.0000383	2.88	BLZF1	basic leucine zipper nuclear factor 1	Upregulated
A_24_P480	0.001227	0.000124	2.88	DOK4	docking protein 4	Upregulated
A_23_P324	0.002369	0.000396	2.88	KLHL7	kelch-like family member 7	Upregulated
A_22_P000	0.003834	0.000822	2.88	lnc-NKX6-3-2	lnc-NKX6-3-2:1	Upregulated
A_33_P681	0.000521	0.0000107	2.87	THUMPD3-AS	THUMPD3 antisense RNA 1	Upregulated
A_21_P001	0.000526	0.0000122	2.87	LINC01347	long intergenic non-protein coding RNA 1347	Upregulated
A_33_P336	0.00055	0.000015	2.87	RPS6KB1	ribosomal protein S6 kinase, 70kDa, polypeptide 1	Upregulated
A_32_P116	0.000578	0.0000191	2.87	LINC00094	long intergenic non-protein coding RNA 94	Upregulated
A_22_P000	0.000647	0.0000267	2.87	lnc-GNAT2-1	lnc-GNAT2-1:1	Upregulated
A_33_P322	0.000647	0.0000267	2.87	XPO5	exportin 5	Upregulated
A_19_P008	0.001462	0.000172	2.87	KPNA4	karyopherin alpha 4 (importin alpha 3)	Upregulated
A_24_P316	0.001674	0.000218	2.87	AQR	aquarius intron-binding spliceosomal factor	Upregulated
A_24_P860	0.002308	0.00038	2.87	PAIP2B	poly(A) binding protein interacting protein 2B	Upregulated
A_24_P836	0.002804	0.000511	2.87	MMS22L	MMS22-like, DNA repair protein	Upregulated
A_21_P001	0.005749	0.00149	2.87	N6AMT1	N-6 adenine-specific DNA methyltransferase 1 (putative)	Upregulated
A_22_P000	0.006874	0.00192	2.87	DHRS4-AS1	DHRS4 antisense RNA 1	Upregulated
A_33_P324	0.002868	0.000529	2.86	CIZ1	CDKN1A interacting zinc finger protein 1	Upregulated
A_24_P144	0.003032	0.000578	2.86	RNF145	ring finger protein 145	Upregulated
A_22_P000	0.010028	0.00321	2.86	lnc-C6orf228-	lnc-C6orf228-3:1	Upregulated
A_19_P008	0.000521	0.0000103	2.85	CASC15	cancer susceptibility candidate 15 (non-protein coding)	Upregulated
A_33_P325	0.000603	0.0000224	2.85	PDSS1	prenyl (decaprenyl) diphosphate synthase, subunit 1	Upregulated
A_23_P367	0.000848	0.0000542	2.85	TAPBPL	TAP binding protein-like	Upregulated

A_33_P322	0.001421	0.000163	2.85	AKNA	AT-hook transcription factor	Upregulated
A_23_P728	0.00356	0.000737	2.85	ELMOD2	ELMO/CED-12 domain containing 2	Upregulated
A_23_P137	0.000515	9.75E-06	2.84	PIR	pirin (iron-binding nuclear protein)	Upregulated
A_23_P133	0.000884	0.0000593	2.84	CDK7	cyclin-dependent kinase 7	Upregulated
A_23_P800	0.001589	0.000199	2.84	E2F1	E2F transcription factor 1	Upregulated
A_23_P257	0.002608	0.000459	2.84	CENPB	centromere protein B, 80kDa	Upregulated
A_33_P340	0.003079	0.00059	2.84	MUC22	mucin 22	Upregulated
A_24_P276	0.005388	0.00136	2.84	SNIP1	Smad nuclear interacting protein 1	Upregulated
A_21_P000	0.005683	0.00146	2.84	LRRC75A-AS	LRRC75A antisense RNA 1	Upregulated
A_24_P694	0.009539	0.003	2.84	lnc-ZNF236-1	lnc-ZNF236-1:5	Upregulated
A_23_P351	0.000521	0.0000103	2.83	SKIL	SKI-like proto-oncogene	Upregulated
A_33_P325	0.000675	0.0000293	2.83	COG4	component of oligomeric golgi complex 4	Upregulated
A_33_P333	0.001087	0.0000965	2.83	ZNF669	zinc finger protein 669	Upregulated
A_24_P124	0.001157	0.00011	2.83	VEGFA	vascular endothelial growth factor A	Upregulated
A_23_P970	0.001532	0.000187	2.83	FBXO6	F-box protein 6	Upregulated
A_33_P329	0.00275	0.000497	2.83	HDAC8	histone deacetylase 8	Upregulated
A_33_P342	0.003843	0.000825	2.83	ZNF790	zinc finger protein 790	Upregulated
A_33_P331	0.0141	0.00498	2.83	FXN	frataxin	Upregulated
A_23_P252	0.000527	0.000013	2.82	SCAP	SREBF chaperone	Upregulated
A_23_P122	0.000555	0.0000153	2.82	ZSCAN9	zinc finger and SCAN domain containing 9	Upregulated
A_21_P001	0.00056	0.0000156	2.82	lnc-MRPL39-4	lnc-MRPL39-4:1	Upregulated
A_23_P159	0.00075	0.0000405	2.82	SLC35E3	solute carrier family 35, member E3	Upregulated
A_33_P324	0.001162	0.000112	2.82	MNT	MAX network transcriptional repressor	Upregulated
A_22_P000	0.002568	0.000448	2.82	HPYR1	Helicobacter pylori responsive 1 (non-protein coding)	Upregulated
A_33_P325	0.002832	0.000519	2.82	VSIG10	V-set and immunoglobulin domain containing 10	Upregulated
A_24_P392	0.004383	0.001	2.82	ING1	inhibitor of growth family, member 1	Upregulated
A_24_P998	0.000515	9.33E-06	2.81	ZNF223	zinc finger protein 223	Upregulated
A_23_P528	0.000555	0.0000153	2.81	BUD13	BUD13 homolog (S. cerevisiae)	Upregulated
A_24_P145	0.00121	0.000121	2.81	SERINC2	serine incorporator 2	Upregulated
A_32_P779	0.00189	0.000271	2.81	UTP11L	UTP11-like, U3 small nucleolar ribonucleoprotein (yeast)	Upregulated
A_24_P184	0.003094	0.000596	2.81	CHD2	chromodomain helicase DNA binding protein 2	Upregulated
A_32_P151	0.000521	0.0000106	2.8	KRT18	keratin 18, type I	Upregulated
A_23_P210	0.000686	0.0000301	2.8	LGALSL	lectin, galactoside-binding-like	Upregulated
A_23_P138	0.000961	0.000072	2.8	CAPN1	calpain 1, (mu/I) large subunit	Upregulated
A_23_P416	0.000526	0.0000126	2.79	JADE2	jade family PHD finger 2	Upregulated
A_23_P164	0.000537	0.0000142	2.79	RNASEH2A	ribonuclease H2, subunit A	Upregulated
A_22_P000	0.001525	0.000186	2.79	lnc-DERA-1	lnc-DERA-1:3	Upregulated

A_23_P133	0.001983	0.000296	2.79	GPANK1	G patch domain and ankyrin repeats 1	Upregulated
A_23_P418	0.002101	0.000327	2.79	BCL2L2	BCL2-like 2	Upregulated
A_23_P256	0.002166	0.000345	2.79	FAM167A-AS	FAM167A antisense RNA 1	Upregulated
A_23_P145	0.003343	0.000672	2.79	DNAH8	dynein, axonemal, heavy chain 8	Upregulated
A_33_P340	0.005545	0.00141	2.79	VAMP4	vesicle-associated membrane protein 4	Upregulated
A_23_P153	0.000575	0.0000176	2.78	ZNF492	zinc finger protein 492	Upregulated
A_23_P136	0.000602	0.0000219	2.78	FBXO8	F-box protein 8	Upregulated
A_21_P000	0.000755	0.0000412	2.78	SNORD104	small nucleolar RNA, C/D box 104	Upregulated
A_23_P124	0.000781	0.0000445	2.78	FER	fer (fps/fes related) tyrosine kinase	Upregulated
A_22_P000	0.000781	0.0000445	2.78	lnc-DUOX2-2	lnc-DUOX2-2:1	Upregulated
A_23_P336	0.000912	0.0000636	2.78	TOR1AIP2	torsin A interacting protein 2	Upregulated
A_33_P336	0.001032	0.0000853	2.78	STAMBP	STAM binding protein	Upregulated
A_21_P001	0.001303	0.000139	2.78	TNPO1	transportin 1	Upregulated
A_24_P355	0.001626	0.000208	2.78	SLC25A25	solute carrier family 25 (mitochondrial carrier; phosphate carrier), membe	Upregulated
A_22_P000	0.004073	0.000895	2.78	HMBOX1	homeobox containing 1	Upregulated
A_23_P120	0.005219	0.00129	2.78	TASP1	taspase, threonine aspartase, 1	Upregulated
A_23_P825	0.008351	0.00251	2.78	PEG10	paternally expressed 10	Upregulated
A_33_P352	0.009659	0.00305	2.78	KDM2B	lysine (K)-specific demethylase 2B	Upregulated
A_32_P103	0.000521	0.0000107	2.77	FAM92A1	family with sequence similarity 92, member A1	Upregulated
A_24_P729	0.000525	0.0000115	2.77	AGAP9	ArfGAP with GTPase domain, ankyrin repeat and PH domain 9	Upregulated
A_23_P133	0.000527	0.0000128	2.77	SLC29A1	solute carrier family 29 (equilibrative nucleoside transporter), member 1	Upregulated
A_24_P229	0.000577	0.0000182	2.77	ARRDC2	arrestin domain containing 2	Upregulated
A_33_P335	0.000597	0.0000212	2.77	SIAH2	siah E3 ubiquitin protein ligase 2	Upregulated
A_19_P008	0.000883	0.0000591	2.77	NUDT3	nudix (nucleoside diphosphate linked moiety X)-type motif 3	Upregulated
A_22_P000	0.001013	0.000082	2.77	lnc-C13orf15-	lnc-C13orf15-1:1	Upregulated
A_33_P321	0.001139	0.000107	2.77	PRKRIR	protein-kinase, interferon-inducible double stranded RNA dependent inh	Upregulated
A_22_P000	0.003094	0.000595	2.77	MIR17HG	miR-17-92 cluster host gene (non-protein coding)	Upregulated
A_23_P126	0.005267	0.00131	2.77	CENPL	centromere protein L	Upregulated
A_23_P433	0.000526	0.0000121	2.76	SPRTN	SprT-like N-terminal domain	Upregulated
A_33_P338	0.000526	0.0000122	2.76	SFMBT2	Scm-like with four mbt domains 2	Upregulated
A_33_P331	0.000526	0.0000123	2.76	AHR	aryl hydrocarbon receptor	Upregulated
A_21_P000	0.000709	0.0000351	2.76	lnc-KIAA1383	lnc-KIAA1383-1:1	Upregulated
A_23_P792	0.001122	0.000104	2.76	ACVR1	activin A receptor, type I	Upregulated
A_24_P548	0.001763	0.00024	2.76	KLHL8	kelch-like family member 8	Upregulated
A_24_P161	0.009311	0.0029	2.76	SPDYE2	speedy/RINGO cell cycle regulator family member E2	Upregulated
A_33_P326	0.00056	0.0000165	2.75	CDK8	cyclin-dependent kinase 8	Upregulated
A_23_P409	0.000645	0.0000264	2.75	POLR1D	polymerase (RNA) I polypeptide D, 16kDa	Upregulated

A_23_P119	0.000646	0.0000265	2.75	KEAP1	kelch-like ECH-associated protein 1	Upregulated
A_21_P001	0.000809	0.0000491	2.75	lnc-MAPK8IP2	lnc-MAPK8IP2-1:14	Upregulated
A_21_P000	0.002944	0.00055	2.75	LOC1027246	uncharacterized LOC102724687	Upregulated
A_23_P257	0.000603	0.0000225	2.74	LOH12CR1	loss of heterozygosity, 12, chromosomal region 1	Upregulated
A_23_P306	0.000995	0.000079	2.74	CXorf40B	chromosome X open reading frame 40B	Upregulated
A_23_P165	0.001062	0.0000906	2.74	FARSB	phenylalanyl-tRNA synthetase, beta subunit	Upregulated
A_24_P336	0.001198	0.000118	2.74	HTRA2	HtrA serine peptidase 2	Upregulated
A_33_P326	0.00187	0.000267	2.74	ZFAT	zinc finger and AT hook domain containing	Upregulated
A_23_P696	0.00195	0.000286	2.74	OTUD4	OTU deubiquitinase 4	Upregulated
A_32_P719	0.003355	0.000675	2.74	DSTYK	dual serine/threonine and tyrosine protein kinase	Upregulated
A_22_P000	0.000526	0.0000123	2.73	MT1B	metallothionein 1B	Upregulated
A_24_P361	0.000526	0.0000126	2.73	PROSER3	proline and serine rich 3	Upregulated
A_23_P207	0.000705	0.0000333	2.73	RBM18	RNA binding motif protein 18	Upregulated
A_21_P000	0.000939	0.0000679	2.73	ADORA2A-AS	ADORA2A antisense RNA 1	Upregulated
A_33_P339	0.001321	0.000142	2.73	FAM178A	family with sequence similarity 178, member A	Upregulated
A_21_P001	0.001462	0.000172	2.73	BMS1P5	BMS1 pseudogene 5	Upregulated
A_23_P657	0.001689	0.000222	2.73	MCTP2	multiple C2 domains, transmembrane 2	Upregulated
A_24_P693	0.001747	0.000236	2.73	CASD1	CAS1 domain containing 1	Upregulated
A_23_P171	0.002118	0.000333	2.73	MAL	mal, T-cell differentiation protein	Upregulated
A_33_P325	0.0033	0.000659	2.73	CMA1	chymase 1, mast cell	Upregulated
A_22_P000	0.005359	0.00135	2.73	LOC439933	uncharacterized LOC439933	Upregulated
A_22_P000	0.007735	0.00226	2.73	lnc-RP11-366	lnc-RP11-366L20.2.1-1:1	Upregulated
A_23_P668	0.010804	0.00352	2.73	GEMIN4	gem (nuclear organelle) associated protein 4	Upregulated
A_22_P000	0.000525	0.0000111	2.72	lnc-LENG9-1	lnc-LENG9-1:3	Upregulated
A_23_P203	0.00056	0.0000159	2.72	API5	apoptosis inhibitor 5	Upregulated
A_22_P000	0.000646	0.0000265	2.72	lnc-TRAPPC4	lnc-TRAPPC4-1:1	Upregulated
A_21_P000	0.000866	0.0000572	2.72	lnc-HIST1H2A	lnc-HIST1H2AI-2:1	Upregulated
A_24_P743	0.003909	0.000845	2.72	BBIP1	BBSome interacting protein 1	Upregulated
A_24_P198	0.006469	0.00177	2.72	CDK12	cyclin-dependent kinase 12	Upregulated
A_23_P131	0.000526	0.0000118	2.71	C2orf47	chromosome 2 open reading frame 47	Upregulated
A_23_P126	0.000597	0.0000213	2.71	KIF21B	kinesin family member 21B	Upregulated
A_23_P215	0.00063	0.000025	2.71	WDR91	WD repeat domain 91	Upregulated
A_24_P320	0.000695	0.0000317	2.71	TRMT44	tRNA methyltransferase 44 homolog (S. cerevisiae)	Upregulated
A_33_P332	0.000707	0.0000342	2.71	BMP2K	BMP2 inducible kinase	Upregulated
A_23_P279	0.000709	0.0000349	2.71	PDCD2L	programmed cell death 2-like	Upregulated
A_21_P000	0.004962	0.0012	2.71	SNORD52	small nucleolar RNA, C/D box 52	Upregulated
A_22_P000	0.000532	0.0000132	2.7	LINC00851	long intergenic non-protein coding RNA 851	Upregulated

A_21_P001	0.001851	0.000262	2.7	LOC1019292	uncharacterized LOC101929266	Upregulated
A_23_P638	0.001873	0.000267	2.7	SAMD8	sterile alpha motif domain containing 8	Upregulated
A_23_P325	0.003667	0.000769	2.7	ZNF134	zinc finger protein 134	Upregulated
A_24_P332	0.007718	0.00225	2.7	SSH1	slingshot protein phosphatase 1	Upregulated
A_23_P120	0.000578	0.0000187	2.69	SPEN	spen family transcriptional repressor	Upregulated
A_22_P000	0.001265	0.000132	2.69	lnc-AC09229!	lnc-AC092295.7.1-2:1	Upregulated
A_33_P330	0.001328	0.000144	2.69	STRADB	STE20-related kinase adaptor beta	Upregulated
A_22_P000	0.001469	0.000173	2.69	lnc-EFCAB5-1	lnc-EFCAB5-1:1	Upregulated
A_33_P322	0.00429	0.00097	2.69	STK17A	serine/threonine kinase 17a	Upregulated
A_24_P372	0.006054	0.0016	2.69	RNF141	ring finger protein 141	Upregulated
A_23_P110	0.000639	0.0000257	2.68	MGST2	microsomal glutathione S-transferase 2	Upregulated
A_33_P324	0.003304	0.000661	2.68	RAPGEF6	Rap guanine nucleotide exchange factor (GEF) 6	Upregulated
A_19_P003	0.004274	0.000965	2.68	lnc-NR5A2-1	lnc-NR5A2-1:2	Upregulated
A_33_P324	0.008398	0.00253	2.68	LOC606724	coronin, actin binding protein, 1A pseudogene	Upregulated
A_33_P337	0.010065	0.00322	2.68	FAM196B	family with sequence similarity 196, member B	Upregulated
A_32_P306	0.012752	0.00437	2.68	ETV5	ets variant 5	Upregulated
A_24_P918	0.001595	0.0002	2.67	TMTC3	transmembrane and tetratricopeptide repeat containing 3	Upregulated
A_24_P380	0.001826	0.000256	2.67	LSMEM1	leucine-rich single-pass membrane protein 1	Upregulated
A_23_P135	0.003966	0.000863	2.67	AFG3L2	AFG3-like AAA ATPase 2	Upregulated
A_23_P250	0.005296	0.00132	2.67	PRKCE	protein kinase C, epsilon	Upregulated
A_23_P376	0.000526	0.0000116	2.66	TNF	tumor necrosis factor	Upregulated
A_23_P192	0.000526	0.0000127	2.66	TUBB2A	tubulin, beta 2A class IIa	Upregulated
A_22_P000	0.000529	0.000013	2.66	lnc-LOH12CR	lnc-LOH12CR1-1:1	Upregulated
A_23_P129	0.000581	0.0000197	2.66	IL4	interleukin 4	Upregulated
A_23_P255	0.000775	0.0000437	2.66	LHFPL2	lipoma HMGIC fusion partner-like 2	Upregulated
A_33_P325	0.002499	0.000429	2.66	ST6GALNAC(	ST6 (alpha-N-acetyl-neuraminyl-2,3-beta-galactosyl-1,3)-N-acetylgalacto	Upregulated
A_24_P158	0.002989	0.000564	2.66	DTX4	deltex 4, E3 ubiquitin ligase	Upregulated
A_33_P324	0.009077	0.00281	2.66	IGFL3	IGF-like family member 3	Upregulated
A_21_P000	0.010702	0.00348	2.66	SEC24B-AS1	SEC24B antisense RNA 1	Upregulated
A_21_P001	0.00056	0.0000161	2.65	NUTM2B-AS1	NUTM2B antisense RNA 1	Upregulated
A_23_P133	0.00072	0.0000371	2.65	RIOK2	RIO kinase 2	Upregulated
A_22_P000	0.000788	0.0000459	2.65	LMO7-AS1	LMO7 antisense RNA 1	Upregulated
A_23_P208	0.001043	0.0000873	2.65	ADCK4	aarF domain containing kinase 4	Upregulated
A_24_P759	0.00124	0.000126	2.65	APOL6	apolipoprotein L, 6	Upregulated
A_21_P000	0.003687	0.000776	2.65	LARGE-AS1	LARGE antisense RNA 1	Upregulated
A_24_P918	0.00509	0.00124	2.65	WHAMMP3	WAS protein homolog associated with actin, golgi membranes and micro	Upregulated
A_23_P343	0.000563	0.0000168	2.64	RSRP1	arginine/serine-rich protein 1	Upregulated

A_19_P003	0.000635	0.0000253	2.64	PPP4R1L	protein phosphatase 4, regulatory subunit 1-like (pseudogene)	Upregulated
A_23_P718	0.000695	0.0000312	2.64	PBX3	pre-B-cell leukemia homeobox 3	Upregulated
A_24_P317	0.000788	0.0000458	2.64	TMEM8B	transmembrane protein 8B	Upregulated
A_23_P414	0.001226	0.000124	2.64	FLCN	folliculin	Upregulated
A_33_P326	0.004254	0.000958	2.64	PAAF1	proteasomal ATPase-associated factor 1	Upregulated
A_23_P716	0.004786	0.00114	2.64	PAX5	paired box 5	Upregulated
A_24_P128	0.000575	0.0000176	2.63	ATF2	activating transcription factor 2	Upregulated
A_23_P153	0.001462	0.000172	2.63	TGIF1	TGFB-induced factor homeobox 1	Upregulated
A_23_P256	0.002757	0.000499	2.63	VBP1	von Hippel-Lindau binding protein 1	Upregulated
A_33_P333	0.000526	0.000012	2.62	SEC61A2	Sec61 alpha 2 subunit (S. cerevisiae)	Upregulated
A_33_P341	0.000575	0.0000177	2.62	AIG1	androgen-induced 1	Upregulated
A_23_P364	0.000814	0.0000499	2.62	SLX4IP	SLX4 interacting protein	Upregulated
A_23_P365	0.000963	0.0000727	2.62	PRKAG1	protein kinase, AMP-activated, gamma 1 non-catalytic subunit	Upregulated
A_24_P318	0.00097	0.0000739	2.62	RPUSD4	RNA pseudouridylate synthase domain containing 4	Upregulated
A_23_P109	0.001023	0.000084	2.62	ZBTB11	zinc finger and BTB domain containing 11	Upregulated
A_24_P296	0.001714	0.000229	2.62	LINC00052	long intergenic non-protein coding RNA 52	Upregulated
A_33_P339	0.001873	0.000267	2.62	FAM188A	family with sequence similarity 188, member A	Upregulated
A_33_P327	0.00275	0.000497	2.62	GOSR1	golgi SNAP receptor complex member 1	Upregulated
A_23_P569	0.004115	0.000909	2.62	REL	v-rel avian reticuloendotheliosis viral oncogene homolog	Upregulated
A_33_P330	0.005749	0.00149	2.62	TLE4	transducin-like enhancer of split 4	Upregulated
A_23_P204	0.005796	0.00151	2.62	CAB39L	calcium binding protein 39-like	Upregulated
A_22_P000	0.000577	0.0000184	2.61	LOC1027238	uncharacterized LOC102723809	Upregulated
A_24_P454	0.000582	0.0000198	2.61	XCL1	chemokine (C motif) ligand 1	Upregulated
A_23_P136	0.00072	0.000037	2.61	WIPF2	WAS/WASL interacting protein family, member 2	Upregulated
A_23_P150	0.000899	0.0000611	2.61	RPP38	ribonuclease P/MRP 38kDa subunit	Upregulated
A_23_P896	0.001643	0.000212	2.61	IL7	interleukin 7	Upregulated
A_33_P326	0.002931	0.000547	2.61	ZNF280B	zinc finger protein 280B	Upregulated
A_22_P000	0.007492	0.00216	2.61	lnc-DDX31-1	lnc-DDX31-1:1	Upregulated
A_33_P323	0.008491	0.00257	2.61	FBXL18	F-box and leucine-rich repeat protein 18	Upregulated
A_23_P710	0.010451	0.00338	2.61	IL6	interleukin 6	Upregulated
A_33_P336	0.000577	0.0000186	2.6	P2RX4	purinergic receptor P2X, ligand gated ion channel, 4	Upregulated
A_23_P420	0.000578	0.0000187	2.6	NOD2	nucleotide-binding oligomerization domain containing 2	Upregulated
A_23_P980	0.000641	0.000026	2.6	CUTC	cutC copper transporter	Upregulated
A_23_P145	0.000684	0.00003	2.6	TSPAN12	tetraspanin 12	Upregulated
A_21_P001	0.002024	0.000307	2.6	XLOC_l2_005997		Upregulated
A_33_P336	0.012007	0.00405	2.6	PRPF31	pre-mRNA processing factor 31	Upregulated
A_22_P000	0.000707	0.0000337	2.59	lnc-KCNJ4-2	lnc-KCNJ4-2:1	Upregulated

A_33_P328	0.000783	0.0000449	2.59	CDPF1	cysteine-rich, DPF motif domain containing 1	Upregulated
A_33_P332	0.000938	0.0000678	2.59	SGPL1	sphingosine-1-phosphate lyase 1	Upregulated
A_22_P000	0.000954	0.0000705	2.59	lnc-COIL-3	lnc-COIL-3:1	Upregulated
A_33_P323	0.004433	0.00102	2.59	LOC257396	uncharacterized LOC257396	Upregulated
A_33_P323	0.005187	0.00128	2.59	LINC01144	long intergenic non-protein coding RNA 1144	Upregulated
A_23_P104	0.000578	0.0000192	2.58	VPS51	vacuolar protein sorting 51 homolog (S. cerevisiae)	Upregulated
A_23_P421	0.000648	0.0000269	2.58	TNFAIP2	tumor necrosis factor, alpha-induced protein 2	Upregulated
A_33_P333	0.001291	0.000137	2.58	SNORA62	small nucleolar RNA, H/ACA box 62	Upregulated
A_23_P107	0.003847	0.000826	2.58	CEP76	centrosomal protein 76kDa	Upregulated
A_24_P929	0.000601	0.0000217	2.57	AP4E1	adaptor-related protein complex 4, epsilon 1 subunit	Upregulated
A_23_P135	0.000658	0.0000275	2.57	PPP2R2D	protein phosphatase 2, regulatory subunit B, delta	Upregulated
A_33_P327	0.001015	0.0000824	2.57	ZNF75A	zinc finger protein 75a	Upregulated
A_21_P000	0.001612	0.000205	2.57	LHX4-AS1	LHX4 antisense RNA 1	Upregulated
A_33_P333	0.00204	0.000312	2.57	LOC1001298	uncharacterized LOC100129846	Upregulated
A_21_P000	0.00356	0.000737	2.57	lnc-APLN-1	lnc-APLN-1:1	Upregulated
A_33_P336	0.000532	0.0000134	2.56	TUBB2A	tubulin, beta 2A class IIa	Upregulated
A_32_P701	0.000538	0.0000143	2.56	TSPAN15	tetraspanin 15	Upregulated
A_22_P000	0.000559	0.0000154	2.56	lnc-ERMP1-1	lnc-ERMP1-1:1	Upregulated
A_23_P206	0.000575	0.0000178	2.56	USP31	ubiquitin specific peptidase 31	Upregulated
A_23_P349	0.000578	0.0000189	2.56	RIMKLA	ribosomal modification protein rimK-like family member A	Upregulated
A_33_P328	0.000848	0.0000544	2.56	CDPF1	cysteine-rich, DPF motif domain containing 1	Upregulated
A_33_P329	0.000904	0.0000617	2.56	CDKN2C	cyclin-dependent kinase inhibitor 2C (p18, inhibits CDK4)	Upregulated
A_23_P146	0.00097	0.0000739	2.56	BAG4	BCL2-associated athanogene 4	Upregulated
A_21_P000	0.001278	0.000135	2.56	lnc-XRCC2-2	lnc-XRCC2-2:1	Upregulated
A_22_P000	0.001606	0.000203	2.56	lnc-ZBTB10-3	lnc-ZBTB10-3:1	Upregulated
A_23_P122	0.002101	0.000327	2.56	GFOD1	glucose-fructose oxidoreductase domain containing 1	Upregulated
A_23_P784	0.002505	0.000431	2.56	ZNF350	zinc finger protein 350	Upregulated
A_32_P444	0.000578	0.000019	2.55	INPP1	inositol polyphosphate-1-phosphatase	Upregulated
A_24_P130	0.000603	0.0000222	2.55	LDLRAD4	low density lipoprotein receptor class A domain containing 4	Upregulated
A_23_P204	0.000654	0.0000272	2.55	NDRG1	N-myc downstream regulated 1	Upregulated
A_32_P112	0.000853	0.000055	2.55	FAM27E2	family with sequence similarity 27, member E2	Upregulated
A_24_P150	0.000938	0.0000672	2.55	GNA13	guanine nucleotide binding protein (G protein), alpha 13	Upregulated
A_23_P160	0.001291	0.000137	2.55	GLMN	glomulin, FKBP associated protein	Upregulated
A_32_P831	0.005846	0.00152	2.55	BRI3BP	BRI3 binding protein	Upregulated
A_23_P301	0.013704	0.0048	2.55	KLF3-AS1	KLF3 antisense RNA 1	Upregulated
A_32_P840	0.000561	0.0000166	2.54	TIMM23B	translocase of inner mitochondrial membrane 23 homolog B (yeast)	Upregulated
A_23_P365	0.000566	0.0000171	2.54	BCKDK	branched chain ketoacid dehydrogenase kinase	Upregulated

A_23_P503	0.001176	0.000114	2.54	SLC24A4	solute carrier family 24 (sodium/potassium/calcium exchanger), member	Upregulated
A_23_P139	0.001522	0.000185	2.54	SERPING1	serpin peptidase inhibitor, clade G (C1 inhibitor), member 1	Upregulated
A_19_P003	0.001709	0.000229	2.54	SNHG11	small nucleolar RNA host gene 11 (non-protein coding)	Upregulated
A_33_P323	0.001733	0.000233	2.54	PIP5KL1	phosphatidylinositol-4-phosphate 5-kinase-like 1	Upregulated
A_24_P416	0.001962	0.000291	2.54	KIAA0195	KIAA0195	Upregulated
A_33_P321	0.003393	0.000687	2.54	LINC01061	long intergenic non-protein coding RNA 1061	Upregulated
A_23_P737	0.004528	0.00105	2.54	LAGE3	L antigen family, member 3	Upregulated
A_23_P251	0.005487	0.00139	2.54	GPC2	glypican 2	Upregulated
A_21_P000	0.015102	0.00547	2.54	TTF2	transcription termination factor, RNA polymerase II	Upregulated
A_21_P001	0.00056	0.0000161	2.53	XLOC_l2_015596		Upregulated
A_23_P162	0.000577	0.0000183	2.53	CCDC91	coiled-coil domain containing 91	Upregulated
A_33_P333	0.000701	0.0000328	2.53	FCRL5	Fc receptor-like 5	Upregulated
A_23_P201	0.000758	0.0000414	2.53	SNX27	sorting nexin family member 27	Upregulated
A_22_P000	0.000837	0.0000526	2.53	lnc-SYN3-4	lnc-SYN3-4:1	Upregulated
A_22_P000	0.000945	0.0000689	2.53	lnc-COG3-2	lnc-COG3-2:1	Upregulated
A_23_P261	0.000963	0.0000725	2.53	DET1	de-etiolated homolog 1 (Arabidopsis)	Upregulated
A_23_P461	0.001349	0.000148	2.53	MED8	mediator complex subunit 8	Upregulated
A_33_P330	0.001784	0.000245	2.53	MRPL32	mitochondrial ribosomal protein L32	Upregulated
A_24_P290	0.008968	0.00276	2.53	FAM86B3P	family with sequence similarity 86, member A pseudogene	Upregulated
A_22_P000	0.01127	0.00373	2.53	lnc-PHF3-2	lnc-PHF3-2:1	Upregulated
A_33_P334	0.00056	0.0000158	2.52	SOCS4	suppressor of cytokine signaling 4	Upregulated
A_24_P337	0.000991	0.000077	2.52	DCTD	dCMP deaminase	Upregulated
A_23_P278	0.001098	0.0000985	2.52	GPATCH1	G patch domain containing 1	Upregulated
A_21_P000	0.001098	0.0000985	2.52	lnc-PLEKHA5	lnc-PLEKHA5-4:1	Upregulated
A_23_P467	0.001158	0.000111	2.52	ERF	Ets2 repressor factor	Upregulated
A_23_P371	0.001353	0.000149	2.52	KLF12	Kruppel-like factor 12	Upregulated
A_23_P972	0.00067	0.0000288	2.51	ZNF691	zinc finger protein 691	Upregulated
A_22_P000	0.001009	0.0000815	2.51	LOC1049683	ncRNA	Upregulated
A_23_P404	0.001054	0.0000895	2.51	GRPEL2	GrpE-like 2, mitochondrial (E. coli)	Upregulated
A_33_P335	0.001222	0.000123	2.51	ARG1	arginase 1	Upregulated
A_24_P208	0.001693	0.000223	2.51	IL18R1	interleukin 18 receptor 1	Upregulated
A_32_P760	0.002078	0.000321	2.51	CTDSPL2	CTD (carboxy-terminal domain, RNA polymerase II, polypeptide A) small	Upregulated
A_33_P339	0.003549	0.000733	2.51	lnc-PCSK1N-1	lnc-PCSK1N-1:1	Upregulated
A_19_P003	0.005278	0.00132	2.51	LINC00882	long intergenic non-protein coding RNA 882	Upregulated
A_23_P360	0.012751	0.00437	2.51	PLD6	phospholipase D family, member 6	Upregulated
A_33_P341	0.012906	0.00444	2.51	TRIM16	tripartite motif containing 16	Upregulated
A_24_P140	0.000563	0.0000168	2.5	NMT1	N-myristoyltransferase 1	Upregulated

A_24_P128	0.000578	0.0000189	2.5	ZNF79	zinc finger protein 79	Upregulated
A_23_P344	0.000594	0.0000209	2.5	HDGFRP3	hepatoma-derived growth factor, related protein 3	Upregulated
A_22_P000	0.00062	0.000024	2.5	lnc-FOXN1-1	lnc-FOXN1-1:1	Upregulated
A_23_P250	0.000991	0.000077	2.5	LOC1019287	uncharacterized LOC101928710	Upregulated
A_23_P984	0.001188	0.000116	2.5	SIDT2	SID1 transmembrane family, member 2	Upregulated
A_22_P000	0.002058	0.000316	2.5	KIZ	kizuna centrosomal protein	Upregulated
A_23_P174	0.002319	0.000383	2.5	PCED1A	PC-esterase domain containing 1A	Upregulated
A_24_P561	0.003041	0.000581	2.5	CREBL2	cAMP responsive element binding protein-like 2	Upregulated
A_23_P391	0.006915	0.00194	2.5	PLXDC1	plexin domain containing 1	Upregulated
A_23_P102	0.00055	0.000015	2.49	RSPH1	radial spoke head 1 homolog (Chlamydomonas)	Upregulated
A_32_P182	0.000578	0.0000194	2.49	AIDA	axin interactor, dorsalization associated	Upregulated
A_23_P345	0.000585	0.00002	2.49	GFPT1	glutamine--fructose-6-phosphate transaminase 1	Upregulated
A_32_P158	0.000601	0.0000218	2.49	LINC00998	long intergenic non-protein coding RNA 998	Upregulated
A_23_P315	0.000695	0.0000311	2.49	R3HDM4	R3H domain containing 4	Upregulated
A_24_P336	0.000695	0.0000316	2.49	PNO1	partner of NOB1 homolog (S. cerevisiae)	Upregulated
A_33_P326	0.001445	0.000169	2.49	ZNF503	zinc finger protein 503	Upregulated
A_22_P000	0.002785	0.000506	2.49	lnc-ZNHIT2-1	lnc-ZNHIT2-1:1	Upregulated
A_23_P184	0.003258	0.000646	2.49	PTPN13	protein tyrosine phosphatase, non-receptor type 13 (APO-1/CD95 (Fas)	Upregulated
A_33_P340	0.004102	0.000904	2.49	TRAF4	TNF receptor-associated factor 4	Upregulated
A_23_P829	0.011739	0.00394	2.49	OGN	osteoglycin	Upregulated
A_23_P379	0.00056	0.0000164	2.48	MT1B	metallothionein 1B	Upregulated
A_24_P929	0.000578	0.0000193	2.48	MKNK2	MAP kinase interacting serine/threonine kinase 2	Upregulated
A_23_P341	0.000585	0.0000202	2.48	POP1	processing of precursor 1, ribonuclease P/MRP subunit (S. cerevisiae)	Upregulated
A_24_P416	0.000603	0.0000223	2.48	APOL3	apolipoprotein L, 3	Upregulated
A_32_P228	0.001555	0.000192	2.48	PPP4R2	protein phosphatase 4, regulatory subunit 2	Upregulated
A_23_P334	0.001559	0.000193	2.48	B3GALNT2	beta-1,3-N-acetylgalactosaminyltransferase 2	Upregulated
A_23_P329	0.001694	0.000224	2.48	DDX10	DEAD (Asp-Glu-Ala-Asp) box polypeptide 10	Upregulated
A_23_P155	0.003804	0.000812	2.48	APOL6	apolipoprotein L, 6	Upregulated
A_33_P333	0.004681	0.0011	2.48	PRKDC	protein kinase, DNA-activated, catalytic polypeptide	Upregulated
A_21_P001	0.004796	0.00114	2.48	XLOC_l2_015760		Upregulated
A_24_P299	0.00056	0.0000162	2.47	PASK	PAS domain containing serine/threonine kinase	Upregulated
A_24_P374	0.000857	0.0000558	2.47	STAG3L2	stromal antigen 3-like 2 (pseudogene)	Upregulated
A_23_P141	0.001047	0.0000881	2.47	ZNF544	zinc finger protein 544	Upregulated
A_22_P000	0.001067	0.0000929	2.47	UBXN7-AS1	UBXN7 antisense RNA 1	Upregulated
A_23_P673	0.001069	0.0000936	2.47	ZNF136	zinc finger protein 136	Upregulated
A_33_P323	0.001158	0.000111	2.47	TTF1	transcription termination factor, RNA polymerase I	Upregulated
A_23_P170	0.003002	0.000568	2.47	SBSPON	somatomedin B and thrombospondin, type 1 domain containing	Upregulated

A_33_P341	0.007806	0.00229	2.47	SNORA59B	small nucleolar RNA, H/ACA box 59B	Upregulated
A_23_P548	0.00056	0.0000162	2.46	MT1A	metallothionein 1A	Upregulated
A_33_P330	0.000561	0.0000166	2.46	SNAP23	synaptosomal-associated protein, 23kDa	Upregulated
A_23_P238	0.000603	0.0000224	2.46	SLC30A1	solute carrier family 30 (zinc transporter), member 1	Upregulated
A_33_P329	0.00062	0.0000241	2.46	LRRC37B	leucine rich repeat containing 37B	Upregulated
A_33_P331	0.000641	0.000026	2.46	CA5BP1	carbonic anhydrase VB pseudogene 1	Upregulated
A_33_P342	0.000695	0.0000316	2.46	KDM4A	lysine (K)-specific demethylase 4A	Upregulated
A_33_P332	0.000868	0.0000574	2.46	SDHC	succinate dehydrogenase complex, subunit C, integral membrane prote	Upregulated
A_23_P101	0.00124	0.000127	2.46	ZNF667	zinc finger protein 667	Upregulated
A_21_P001	0.001787	0.000246	2.46	LOC403323	uncharacterized LOC403323	Upregulated
A_23_P204	0.002459	0.000417	2.46	RNFT2	ring finger protein, transmembrane 2	Upregulated
A_33_P335	0.00338	0.000682	2.46	LCE1B	late cornified envelope 1B	Upregulated
A_22_P000	0.00509	0.00124	2.46	lnc-UVRAG-2	lnc-UVRAG-2:1	Upregulated
A_23_P195	0.010345	0.00334	2.46	PGM3	phosphoglucomutase 3	Upregulated
A_33_P321	0.011349	0.00376	2.46	NOTCH2NL	notch 2 N-terminal like	Upregulated
A_22_P000	0.000577	0.0000181	2.45	LOC1019277	uncharacterized LOC101927761	Upregulated
A_23_P488	0.00062	0.0000239	2.45	GALK2	galactokinase 2	Upregulated
A_23_P169	0.00082	0.0000508	2.45	ZNF608	zinc finger protein 608	Upregulated
A_33_P340	0.000904	0.0000618	2.45	SEL1L	sel-1 suppressor of lin-12-like (C. elegans)	Upregulated
A_21_P001	0.001173	0.000113	2.45	ANKRD30A	ankyrin repeat domain 30A	Upregulated
A_24_P839	0.001996	0.000299	2.45	RPP14	ribonuclease P/MRP 14kDa subunit	Upregulated
A_24_P515	0.003321	0.000666	2.45	CCNL2	cyclin L2	Upregulated
A_23_P127	0.003321	0.000666	2.45	ZNF214	zinc finger protein 214	Upregulated
A_33_P339	0.012939	0.00446	2.45	CTU1	cytosolic thiouridylase subunit 1	Upregulated
A_21_P000	0.00062	0.000024	2.44	lnc-SCRG1-1	lnc-SCRG1-1:4	Upregulated
A_21_P001	0.000707	0.000034	2.44	LOC1019280	uncharacterized LOC101928054	Upregulated
A_24_P598	0.00096	0.0000717	2.44	RNF216P1	ring finger protein 216 pseudogene 1	Upregulated
A_33_P336	0.000995	0.0000785	2.44	APTX	aprataxin	Upregulated
A_33_P321	0.001006	0.0000808	2.44	SF3A1	splicing factor 3a, subunit 1, 120kDa	Upregulated
A_23_P368	0.001029	0.000085	2.44	LILRA6	leukocyte immunoglobulin-like receptor, subfamily A (with TM domain), m	Upregulated
A_21_P001	0.001062	0.0000914	2.44	CRCP	CGRP receptor component	Upregulated
A_24_P481	0.004207	0.000941	2.44	DPY19L4	dpy-19-like 4 (C. elegans)	Upregulated
A_33_P323	0.004743	0.00112	2.44	ZNF266	zinc finger protein 266	Upregulated
A_32_P169	0.005408	0.00136	2.44	OTUD5	OTU deubiquitinase 5	Upregulated
A_23_P463	0.005592	0.00143	2.44	RCC1	regulator of chromosome condensation 1	Upregulated
A_23_P385	0.011144	0.00367	2.44	KCTD6	potassium channel tetramerization domain containing 6	Upregulated
A_33_P335	0.000588	0.0000204	2.43	C6orf48	chromosome 6 open reading frame 48	Upregulated

A_22_P000	0.000707	0.0000344	2.43	FAM110A	family with sequence similarity 110, member A	Upregulated
A_33_P337	0.000801	0.0000478	2.43	LINC00862	long intergenic non-protein coding RNA 862	Upregulated
A_21_P000	0.001051	0.0000888	2.43	lnc-FUT8-1	lnc-FUT8-1:1	Upregulated
A_24_P271	0.00112	0.000103	2.43	C18orf32	chromosome 18 open reading frame 32	Upregulated
A_24_P730	0.001203	0.00012	2.43	TTC12	tetratricopeptide repeat domain 12	Upregulated
A_23_P931	0.003041	0.00058	2.43	SPIN1	spindlin 1	Upregulated
A_23_P130	0.007036	0.00199	2.43	OR1A2	olfactory receptor, family 1, subfamily A, member 2	Upregulated
A_23_P230	0.009133	0.00283	2.43	TTC4	tetratricopeptide repeat domain 4	Upregulated
A_23_P207	0.009923	0.00316	2.43	ARL17B	ADP-ribosylation factor-like 17B	Upregulated
A_23_P313	0.010371	0.00335	2.43	CEP295	centrosomal protein 295kDa	Upregulated
A_24_P380	0.014369	0.00511	2.43	EIF5A2	eukaryotic translation initiation factor 5A2	Upregulated
A_33_P334	0.000577	0.0000185	2.42	BRI3	brain protein I3	Upregulated
A_32_P254	0.000978	0.0000754	2.42	SLC12A2	solute carrier family 12 (sodium/potassium/chloride transporter), member	Upregulated
A_23_P100	0.000995	0.0000786	2.42	AVEN	apoptosis, caspase activation inhibitor	Upregulated
A_24_P765	0.002039	0.000312	2.42	WASF2	WAS protein family, member 2	Upregulated
A_23_P505	0.004962	0.0012	2.42	KCNK6	potassium channel, two pore domain subfamily K, member 6	Upregulated
A_22_P000	0.007726	0.00226	2.42	LOC1019275	uncharacterized LOC101927556	Upregulated
A_23_P500	0.015953	0.00586	2.42	ABCA6	ATP-binding cassette, sub-family A (ABC1), member 6	Upregulated
A_33_P337	0.00056	0.0000161	2.41	N4BP2L2-IT2	N4BPL2 intronic transcript 2 (non-protein coding)	Upregulated
A_23_P322	0.000601	0.0000218	2.41	GPR55	G protein-coupled receptor 55	Upregulated
A_33_P380	0.00067	0.0000288	2.41	FNIP2	folliculin interacting protein 2	Upregulated
A_24_P291	0.000852	0.0000547	2.41	USP4	ubiquitin specific peptidase 4 (proto-oncogene)	Upregulated
A_33_P329	0.001443	0.000168	2.41	VMAC	vimentin-type intermediate filament associated coiled-coil protein	Upregulated
A_23_P707	0.011014	0.00361	2.41	AHI1	Abelson helper integration site 1	Upregulated
A_23_P799	0.011361	0.00377	2.41	ATRN	attractin	Upregulated
A_33_P323	0.01307	0.00452	2.41	AURKAPS1	aurora kinase A pseudogene 1	Upregulated
A_23_P152	0.022837	0.00916	2.41	POLR3E	polymerase (RNA) III (DNA directed) polypeptide E (80kD)	Upregulated
A_24_P462	0.000563	0.0000167	2.4	CENPW	centromere protein W	Upregulated
A_21_P000	0.000577	0.0000185	2.4	lnc-DERA-1	lnc-DERA-1:1	Upregulated
A_24_P109	0.000621	0.0000243	2.4	USP37	ubiquitin specific peptidase 37	Upregulated
A_33_P331	0.000637	0.0000255	2.4	ADRB1	adrenoceptor beta 1	Upregulated
A_24_P925	0.000707	0.0000338	2.4	GM2A	GM2 ganglioside activator	Upregulated
A_33_P326	0.000998	0.0000798	2.4	MRPS25	mitochondrial ribosomal protein S25	Upregulated
A_21_P000	0.001066	0.0000922	2.4	lnc-FAM70A-1	lnc-FAM70A-1:5	Upregulated
A_33_P326	0.002442	0.000413	2.4	WDR19	WD repeat domain 19	Upregulated
A_22_P000	0.005707	0.00147	2.4	PDP2	pyruvate dehyrogenase phosphatase catalytic subunit 2	Upregulated
A_23_P209	0.000596	0.0000211	2.39	SMC6	structural maintenance of chromosomes 6	Upregulated

A_22_P000	0.000707	0.0000339	2.39	lnc-TMEM242	lnc-TMEM242-2:1	Upregulated
A_33_P329	0.000711	0.0000356	2.39	PARP1	poly (ADP-ribose) polymerase 1	Upregulated
A_21_P000	0.000836	0.0000524	2.39	LOC1046135	uncharacterized LOC104613533	Upregulated
A_33_P337	0.000912	0.0000634	2.39	CDC37L1	cell division cycle 37-like 1	Upregulated
A_23_P258	0.000998	0.0000795	2.39	DNAAF5	dynein, axonemal, assembly factor 5	Upregulated
A_23_P152	0.00112	0.000103	2.39	TRIP4	thyroid hormone receptor interactor 4	Upregulated
A_24_P419	0.001442	0.000167	2.39	ZNF248	zinc finger protein 248	Upregulated
A_23_P217	0.001617	0.000206	2.39	SPHAR	S-phase response (cyclin related)	Upregulated
A_24_P943	0.004648	0.00109	2.39	PIKFYVE	phosphoinositide kinase, FYVE finger containing	Upregulated
A_22_P000	0.005462	0.00138	2.39	lnc-PLD6-1	lnc-PLD6-1:1	Upregulated
A_23_P328	0.008458	0.00255	2.39	ZNF519	zinc finger protein 519	Upregulated
A_33_P330	0.000575	0.0000177	2.38	PPM1D	protein phosphatase, Mg2+/Mn2+ dependent, 1D	Upregulated
A_33_P322	0.000578	0.0000192	2.38	H2BFM	H2B histone family, member M	Upregulated
A_21_P001	0.00067	0.0000289	2.38	AK4	adenylate kinase 4	Upregulated
A_23_P213	0.000743	0.0000399	2.38	F2R	coagulation factor II (thrombin) receptor	Upregulated
A_33_P328	0.000933	0.0000666	2.38	GNN	Grp94 neighboring nucleotidase pseudogene	Upregulated
A_33_P327	0.001115	0.000102	2.38	LEO1	Leo1, Paf1/RNA polymerase II complex component, homolog (S. cerevi	Upregulated
A_23_P688	0.001193	0.000117	2.38	DRG1	developmentally regulated GTP binding protein 1	Upregulated
A_23_P385	0.002592	0.000455	2.38	ZFP1	ZFP1 zinc finger protein	Upregulated
A_23_P301	0.002965	0.000557	2.38	LSAMP	limbic system-associated membrane protein	Upregulated
A_33_P332	0.003601	0.00075	2.38	AZI2	5-azacytidine induced 2	Upregulated
A_33_P333	0.000603	0.000022	2.37	LOC1001304	uncharacterized LOC100130463	Upregulated
A_23_P900	0.000684	0.00003	2.37	TMEM205	transmembrane protein 205	Upregulated
A_23_P121	0.000701	0.0000326	2.37	CXCL13	chemokine (C-X-C motif) ligand 13	Upregulated
A_22_P000	0.000712	0.000036	2.37	LOC1005056	uncharacterized LOC100505622	Upregulated
A_24_P915	0.000766	0.0000428	2.37	C9orf91	chromosome 9 open reading frame 91	Upregulated
A_23_P287	0.000819	0.0000506	2.37	KLC1	kinesin light chain 1	Upregulated
A_23_P338	0.001029	0.000085	2.37	BCDIN3D	BCDIN3 domain containing	Upregulated
A_33_P341	0.001187	0.000116	2.37	GNAI3	guanine nucleotide binding protein (G protein), alpha inhibiting activity p	Upregulated
A_23_P178	0.001523	0.000185	2.37	SEC14L2	SEC14-like 2 (S. cerevisiae)	Upregulated
A_33_P325	0.001626	0.000208	2.37	TMEM255B	transmembrane protein 255B	Upregulated
A_23_P307	0.001626	0.000208	2.37	WHSC1	Wolf-Hirschhorn syndrome candidate 1	Upregulated
A_23_P251	0.009612	0.00303	2.37	CDCA7	cell division cycle associated 7	Upregulated
A_22_P000	0.009874	0.00314	2.37	CLIP1-AS1	CLIP1 antisense RNA 1	Upregulated
A_21_P000	0.000578	0.0000187	2.36	SNORD53	small nucleolar RNA, C/D box 53	Upregulated
A_23_P393	0.000645	0.0000264	2.36	TFPI2	tissue factor pathway inhibitor 2	Upregulated
A_23_P167	0.000712	0.0000358	2.36	MAPK9	mitogen-activated protein kinase 9	Upregulated

A_33_P331	0.000776	0.0000439	2.36	CHD1L	chromodomain helicase DNA binding protein 1-like	Upregulated
A_22_P000	0.010809	0.00353	2.36	MIRLET7DHG	MIRLET7D host gene (non-protein coding)	Upregulated
A_21_P001	0.016444	0.00611	2.36	XLOC_l2_009332		Upregulated
A_21_P001	0.000579	0.0000195	2.35	ANKRD20A2	ankyrin repeat domain 20 family, member A2	Upregulated
A_23_P303	0.00063	0.0000251	2.35	CCNH	cyclin H	Upregulated
A_23_P162	0.000707	0.0000344	2.35	VDR	vitamin D (1,25- dihydroxyvitamin D3) receptor	Upregulated
A_33_P338	0.000709	0.000035	2.35	FMNL1	formin-like 1	Upregulated
A_33_P326	0.001022	0.0000838	2.35	SP140	SP140 nuclear body protein	Upregulated
A_24_P360	0.001178	0.000114	2.35	DIP2C	DIP2 disco-interacting protein 2 homolog C (Drosophila)	Upregulated
A_24_P791	0.001863	0.000265	2.35	SCAMP4	secretory carrier membrane protein 4	Upregulated
A_33_P333	0.00317	0.00062	2.35	ARHGAP11A	Rho GTPase activating protein 11A	Upregulated
A_22_P000	0.005802	0.00151	2.35	LINC01063	long intergenic non-protein coding RNA 1063	Upregulated
A_23_P154	0.010321	0.00333	2.35	HIRA	histone cell cycle regulator	Upregulated
A_24_P210	0.013566	0.00474	2.35	SCG3	secretogranin III	Upregulated
A_24_P194	0.018624	0.00711	2.35	SHANK3	SH3 and multiple ankyrin repeat domains 3	Upregulated
A_33_P378	0.000603	0.0000224	2.34	NAV2-AS4	NAV2 antisense RNA 4	Upregulated
A_33_P328	0.000654	0.0000273	2.34	LOC91450	uncharacterized LOC91450	Upregulated
A_22_P000	0.000707	0.000034	2.34	lnc-C11orf41-	lnc-C11orf41-1:1	Upregulated
A_23_P109	0.000755	0.0000411	2.34	PTTG1IP	pituitary tumor-transforming 1 interacting protein	Upregulated
A_23_P343	0.000786	0.0000453	2.34	CCR7	chemokine (C-C motif) receptor 7	Upregulated
A_23_P685	0.000995	0.0000785	2.34	MCM8	minichromosome maintenance complex component 8	Upregulated
A_24_P187	0.000995	0.0000785	2.34	MRPS27	mitochondrial ribosomal protein S27	Upregulated
A_21_P000	0.000995	0.0000785	2.34	RAB11B-AS1	RAB11B antisense RNA 1	Upregulated
A_33_P336	0.001698	0.000225	2.34	LOC1005071	uncharacterized LOC100507195	Upregulated
A_23_P103	0.003056	0.000584	2.34	MAN1C1	mannosidase, alpha, class 1C, member 1	Upregulated
A_22_P000	0.008535	0.00258	2.34	lnc-ALX4-7	lnc-ALX4-7:1	Upregulated
A_22_P000	0.010057	0.00322	2.34	lnc-PXDNL-1	lnc-PXDNL-1:1	Upregulated
A_23_P209	0.014668	0.00527	2.34	ZNF302	zinc finger protein 302	Upregulated
A_33_P321	0.00058	0.0000195	2.33	TMEM167B	transmembrane protein 167B	Upregulated
A_21_P000	0.000583	0.0000198	2.33	lnc-FRG1-2	lnc-FRG1-2:1	Upregulated
A_23_P331	0.000602	0.0000218	2.33	IPO7	importin 7	Upregulated
A_33_P332	0.000707	0.0000338	2.33	TOMM34	translocase of outer mitochondrial membrane 34	Upregulated
A_23_P583	0.000707	0.0000342	2.33	FIP1L1	factor interacting with PAPOLA and CPSF1	Upregulated
A_32_P215	0.000798	0.0000472	2.33	CCDC58	coiled-coil domain containing 58	Upregulated
A_22_P000	0.000848	0.0000541	2.33	lnc-TPX2-1	lnc-TPX2-1:1	Upregulated
A_21_P000	0.001123	0.000104	2.33	SNORA27	small nucleolar RNA, H/ACA box 27	Upregulated
A_33_P340	0.001123	0.000104	2.33	TMEM192	transmembrane protein 192	Upregulated

A_23_P107	0.001355	0.000149	2.33	ZNF324	zinc finger protein 324	Upregulated
A_33_P333	0.00176	0.000239	2.33	FANK1	fibronectin type III and ankyrin repeat domains 1	Upregulated
A_23_P997	0.002124	0.000335	2.33	CDKL1	cyclin-dependent kinase-like 1 (CDC2-related kinase)	Upregulated
A_23_P398	0.002523	0.000436	2.33	GOLGA2P7	golgin A2 pseudogene 7	Upregulated
A_21_P000	0.004983	0.00121	2.33	SNORD67	small nucleolar RNA, C/D box 67	Upregulated
A_32_P215	0.007704	0.00225	2.33	ACACA	acetyl-CoA carboxylase alpha	Upregulated
A_23_P373	0.00861	0.00262	2.33	CWC22	CWC22 spliceosome-associated protein	Upregulated
A_24_P177	0.009659	0.00305	2.33	LONP2	lon peptidase 2, peroxisomal	Upregulated
A_22_P000	0.010107	0.00324	2.33	lnc-EXTL3-5	lnc-EXTL3-5:1	Upregulated
A_23_P341	0.000625	0.0000246	2.32	FAM50A	family with sequence similarity 50, member A	Upregulated
A_22_P000	0.000884	0.0000593	2.32	lnc-RPS27A-1	lnc-RPS27A-1:1	Upregulated
A_33_P324	0.00109	0.000097	2.32	BAAT	bile acid CoA:amino acid N-acyltransferase	Upregulated
A_23_P158	0.001108	0.000101	2.32	ACADSB	acyl-CoA dehydrogenase, short/branched chain	Upregulated
A_21_P000	0.001118	0.000103	2.32	CAMTA1	calmodulin binding transcription activator 1	Upregulated
A_33_P341	0.001295	0.000137	2.32	FSCN1	fascin actin-bundling protein 1	Upregulated
A_22_P000	0.002361	0.000393	2.32	lnc-OBFC2A-1	lnc-OBFC2A-1:1	Upregulated
A_23_P379	0.002968	0.000558	2.32	GNRHR2	gonadotropin-releasing hormone (type 2) receptor 2, pseudogene	Upregulated
A_24_P141	0.003244	0.000643	2.32	CAMK2G	calcium/calmodulin-dependent protein kinase II gamma	Upregulated
A_33_P330	0.00386	0.000831	2.32	HDAC2	histone deacetylase 2	Upregulated
A_33_P325	0.005197	0.00128	2.32	SLC9A7	solute carrier family 9, subfamily A (NHE7, cation proton antiporter 7), m	Upregulated
A_33_P326	0.008018	0.00237	2.32	NALCN	sodium leak channel, non selective	Upregulated
A_23_P317	0.000596	0.000021	2.31	PKIA	protein kinase (cAMP-dependent, catalytic) inhibitor alpha	Upregulated
A_33_P330	0.000658	0.0000275	2.31	SENP1	SUMO1/sentrin specific peptidase 1	Upregulated
A_23_P497	0.000709	0.0000348	2.31	MRPL27	mitochondrial ribosomal protein L27	Upregulated
A_24_P484	0.000913	0.0000637	2.31	RNMT	RNA (guanine-7-) methyltransferase	Upregulated
A_23_P390	0.001858	0.000264	2.31	SPATA13	spermatogenesis associated 13	Upregulated
A_21_P001	0.004281	0.000967	2.31	LOC1019288	uncharacterized LOC101928865	Upregulated
A_23_P649	0.009898	0.00315	2.31	USP5	ubiquitin specific peptidase 5 (isopeptidase T)	Upregulated
A_32_P216	0.00062	0.0000239	2.3	PDXDC2P	pyridoxal-dependent decarboxylase domain containing 2, pseudogene	Upregulated
A_23_P360	0.000691	0.0000306	2.3	SSRP1	structure specific recognition protein 1	Upregulated
A_21_P001	0.000722	0.0000374	2.3	XLOC_l2_002729		Upregulated
A_23_P996	0.001062	0.0000912	2.3	G2E3	G2/M-phase specific E3 ubiquitin protein ligase	Upregulated
A_33_P328	0.001069	0.0000934	2.3	EFR3B	EFR3 homolog B (S. cerevisiae)	Upregulated
A_24_P311	0.002168	0.000346	2.3	PACRGL	PARK2 co-regulated-like	Upregulated
A_24_P169	0.003315	0.000664	2.3	ZNF160	zinc finger protein 160	Upregulated
A_21_P001	0.005821	0.00152	2.3	NUTM2B-AS1	NUTM2B antisense RNA 1	Upregulated
A_33_P323	0.006202	0.00166	2.3	LOC1001302	LP2209	Upregulated

A_23_P388	0.012585	0.0043	2.3	SLC22A15	solute carrier family 22, member 15	Upregulated
A_23_P880	0.000658	0.0000276	2.29	CARKD	carbohydrate kinase domain containing	Upregulated
A_32_P171	0.000693	0.0000309	2.29	UBE2S	ubiquitin-conjugating enzyme E2S	Upregulated
A_23_P207	0.000852	0.0000547	2.29	STAT5A	signal transducer and activator of transcription 5A	Upregulated
A_23_P306	0.001678	0.000219	2.29	MCM9	minichromosome maintenance complex component 9	Upregulated
A_22_P000	0.001815	0.000253	2.29	ORC3	origin recognition complex, subunit 3	Upregulated
A_21_P000	0.002043	0.000313	2.29	lnc-C5orf17-6	lnc-C5orf17-6:1	Upregulated
A_24_P364	0.002091	0.000324	2.29	STX2	syntaxin 2	Upregulated
A_22_P000	0.002337	0.000387	2.29	GPR107	G protein-coupled receptor 107	Upregulated
A_33_P325	0.003258	0.000646	2.29	C14orf80	chromosome 14 open reading frame 80	Upregulated
A_19_P008	0.003335	0.00067	2.29	PSMD14	proteasome (prosome, macropain) 26S subunit, non-ATPase, 14	Upregulated
A_33_P321	0.008968	0.00276	2.29	lnc-CHADL-3	lnc-CHADL-3:1	Upregulated
A_24_P940	0.010809	0.00353	2.29	C17orf104	chromosome 17 open reading frame 104	Upregulated
A_24_P720	0.016422	0.0061	2.29	KIDINS220	kinase D-interacting substrate, 220kDa	Upregulated
A_32_P714	0.017675	0.00667	2.29	NCAPD3	non-SMC condensin II complex, subunit D3	Upregulated
A_33_P340	0.000585	0.0000201	2.28	DCTN1	dynactin 1	Upregulated
A_23_P145	0.000608	0.000023	2.28	MALSU1	mitochondrial assembly of ribosomal large subunit 1	Upregulated
A_33_P321	0.000663	0.0000281	2.28	PCF11	PCF11 cleavage and polyadenylation factor subunit	Upregulated
A_23_P145	0.000794	0.0000467	2.28	CCNC	cyclin C	Upregulated
A_23_P434	0.000961	0.000072	2.28	HN1L	hematological and neurological expressed 1-like	Upregulated
A_33_P333	0.00104	0.0000865	2.28	WBP11	WW domain binding protein 11	Upregulated
A_22_P000	0.001134	0.000106	2.28	lnc-LRRC36-1	lnc-LRRC36-1:1	Upregulated
A_23_P216	0.001603	0.000202	2.28	SLC1A1	solute carrier family 1 (neuronal/epithelial high affinity glutamate transpo	Upregulated
A_21_P000	0.001954	0.000288	2.28	PET117	PET117 homolog (S. cerevisiae)	Upregulated
A_19_P008	0.002544	0.000441	2.28	LOC400958	uncharacterized LOC400958	Upregulated
A_32_P122	0.003012	0.000572	2.28	MSANTD3	Myb/SANT-like DNA-binding domain containing 3	Upregulated
A_23_P459	0.004328	0.000984	2.28	FBXO2	F-box protein 2	Upregulated
A_23_P267	0.008041	0.00238	2.28	CD300C	CD300c molecule	Upregulated
A_22_P000	0.008644	0.00263	2.28	lnc-ZDHHC9-1	lnc-ZDHHC9-1:1	Upregulated
A_24_P935	0.000697	0.0000321	2.27	BCAT1	branched chain amino-acid transaminase 1, cytosolic	Upregulated
A_33_P328	0.000814	0.0000501	2.27	MGC72080	MGC72080 pseudogene	Upregulated
A_24_P294	0.000926	0.0000654	2.27	PLA1A	phospholipase A1 member A	Upregulated
A_33_P341	0.000964	0.0000729	2.27	LOC339192	uncharacterized LOC339192	Upregulated
A_23_P118	0.000974	0.0000748	2.27	HSD17B7	hydroxysteroid (17-beta) dehydrogenase 7	Upregulated
A_23_P484	0.001029	0.0000848	2.27	RAD51B	RAD51 paralog B	Upregulated
A_22_P000	0.0014	0.000159	2.27	FXR1	fragile X mental retardation, autosomal homolog 1	Upregulated
A_24_P932	0.003208	0.000632	2.27	HMBOX1	homeobox containing 1	Upregulated

A_33_P322	0.003291	0.000656	2.27	DLG1	discs, large homolog 1 (Drosophila)	Upregulated
A_33_P340	0.000601	0.0000217	2.26	TMPPE	transmembrane protein with metallophosphoesterase domain	Upregulated
A_33_P330	0.000701	0.0000327	2.26	DCAF12L2	DDB1 and CUL4 associated factor 12-like 2	Upregulated
A_23_P153	0.00072	0.0000371	2.26	ICAM1	intercellular adhesion molecule 1	Upregulated
A_23_P132	0.000899	0.000061	2.26	SIDT1	SID1 transmembrane family, member 1	Upregulated
A_33_P322	0.000995	0.0000787	2.26	EIF2AK2	eukaryotic translation initiation factor 2-alpha kinase 2	Upregulated
A_33_P336	0.001062	0.0000911	2.26	NOL4L	nucleolar protein 4-like	Upregulated
A_24_P380	0.001478	0.000176	2.26	FKBP5	FK506 binding protein 5	Upregulated
A_33_P339	0.001622	0.000207	2.26	KLHL12	kelch-like family member 12	Upregulated
A_33_P323	0.002555	0.000444	2.26	NFYA	nuclear transcription factor Y, alpha	Upregulated
A_23_P967	0.003096	0.000597	2.26	LRRC47	leucine rich repeat containing 47	Upregulated
A_21_P001	0.00388	0.000837	2.26	lnc-ZNF280D-	lnc-ZNF280D-1:1	Upregulated
A_23_P219	0.005332	0.00134	2.26	RGS3	regulator of G-protein signaling 3	Upregulated
A_33_P370	0.005372	0.00135	2.26	SNX24	sorting nexin 24	Upregulated
A_33_P327	0.012267	0.00417	2.26	HEATR5A	HEAT repeat containing 5A	Upregulated
A_32_P813	0.000597	0.0000213	2.25	FAHD2A	fumarylacetoacetate hydrolase domain containing 2A	Upregulated
A_33_P320	0.000603	0.0000222	2.25	IARS	isoleucyl-tRNA synthetase	Upregulated
A_33_P346	0.000801	0.0000476	2.25	DNAJC3	DnaJ (Hsp40) homolog, subfamily C, member 3	Upregulated
A_33_P325	0.000805	0.0000484	2.25	ZYG11A	zyg-11 family member A, cell cycle regulator	Upregulated
A_22_P000	0.000824	0.0000513	2.25	lnc-TFB2M-1	lnc-TFB2M-1:1	Upregulated
A_23_P345	0.000908	0.0000628	2.25	SDCCAG8	serologically defined colon cancer antigen 8	Upregulated
A_21_P000	0.000908	0.0000628	2.25	SNORA61	small nucleolar RNA, H/ACA box 61	Upregulated
A_24_P839	0.001007	0.0000812	2.25	SNRPC	small nuclear ribonucleoprotein polypeptide C	Upregulated
A_24_P404	0.018699	0.00715	2.25	PCYT2	phosphate cytidylyltransferase 2, ethanolamine	Upregulated
A_24_P287	0.000675	0.0000294	2.24	CHMP1A	charged multivesicular body protein 1A	Upregulated
A_33_P323	0.000994	0.0000776	2.24	INPP5F	inositol polyphosphate-5-phosphatase F	Upregulated
A_23_P210	0.001468	0.000173	2.24	ARFGAP1	ADP-ribosylation factor GTPase activating protein 1	Upregulated
A_32_P197	0.002634	0.000465	2.24	FAM76B	family with sequence similarity 76, member B	Upregulated
A_33_P326	0.002846	0.000523	2.24	RFT1	RFT1 homolog (S. cerevisiae)	Upregulated
A_33_P339	0.005746	0.00149	2.24	AP1S2	adaptor-related protein complex 1, sigma 2 subunit	Upregulated
A_33_P327	0.006452	0.00176	2.24	NOL4L	nucleolar protein 4-like	Upregulated
A_21_P000	0.006452	0.00176	2.24	SNORD127	small nucleolar RNA, C/D box 127	Upregulated
A_23_P500	0.009182	0.00285	2.24	TUB	tubby bipartite transcription factor	Upregulated
A_23_P168	0.000697	0.0000321	2.23	RBM28	RNA binding motif protein 28	Upregulated
A_24_P317	0.000755	0.0000411	2.23	SORBS1	sorbin and SH3 domain containing 1	Upregulated
A_33_P335	0.000805	0.0000484	2.23	RBAK	RB-associated KRAB zinc finger	Upregulated
A_33_P324	0.000813	0.0000495	2.23	OR4C6	olfactory receptor, family 4, subfamily C, member 6	Upregulated

A_23_P314	0.001007	0.0000812	2.23	ZNF280C	zinc finger protein 280C	Upregulated
A_24_P409	0.001552	0.000192	2.23	MIR3654	microRNA 3654	Upregulated
A_33_P329	0.001835	0.000258	2.23	HMBOX1	homeobox containing 1	Upregulated
A_23_P900	0.001856	0.000263	2.23	DNAJB1	DnaJ (Hsp40) homolog, subfamily B, member 1	Upregulated
A_24_P121	0.002681	0.000477	2.23	TMEM86B	transmembrane protein 86B	Upregulated
A_33_P328	0.003232	0.000639	2.23	SDF4	stromal cell derived factor 4	Upregulated
A_33_P327	0.003723	0.000787	2.23	UPF2	UPF2 regulator of nonsense transcripts homolog (yeast)	Upregulated
A_33_P337	0.005095	0.00125	2.23	FMO5	flavin containing monooxygenase 5	Upregulated
A_23_P749	0.005232	0.0013	2.23	URB2	URB2 ribosome biogenesis 2 homolog (S. cerevisiae)	Upregulated
A_24_P226	0.010168	0.00327	2.23	MGLL	monoglyceride lipase	Upregulated
A_33_P368	0.011343	0.00376	2.23	MAGED2	melanoma antigen family D, 2	Upregulated
A_22_P000	0.013889	0.00488	2.23	LOC1019271	uncharacterized LOC101927156	Upregulated
A_19_P003	0.021337	0.00841	2.23	LINC01021	long intergenic non-protein coding RNA 1021	Upregulated
A_22_P000	0.000605	0.0000228	2.22	lnc-ABCC3-1	lnc-ABCC3-1:1	Upregulated
A_33_P339	0.000642	0.0000262	2.22	PCMT1	protein-L-isoaspartate (D-aspartate) O-methyltransferase	Upregulated
A_23_P321	0.000697	0.000032	2.22	INADL	InaD-like (Drosophila)	Upregulated
A_24_P157	0.000801	0.0000478	2.22	TNFAIP3	tumor necrosis factor, alpha-induced protein 3	Upregulated
A_23_P309	0.00129	0.000137	2.22	ZSCAN25	zinc finger and SCAN domain containing 25	Upregulated
A_24_P281	0.001443	0.000168	2.22	GNPTAB	N-acetylglucosamine-1-phosphate transferase, alpha and beta subunits	Upregulated
A_24_P414	0.002889	0.000536	2.22	ALG3	ALG3, alpha-1,3- mannosyltransferase	Upregulated
A_24_P399	0.033718	0.0149	2.22	CENPM	centromere protein M	Upregulated
A_19_P008	0.000603	0.0000224	2.21	CDCA7L	cell division cycle associated 7-like	Upregulated
A_23_P109	0.00071	0.0000353	2.21	PRNP	prion protein	Upregulated
A_32_P163	0.001203	0.00012	2.21	SCD	stearoyl-CoA desaturase (delta-9-desaturase)	Upregulated
A_22_P000	0.001415	0.000162	2.21	LINC00641	long intergenic non-protein coding RNA 641	Upregulated
A_24_P104	0.003032	0.000578	2.21	RHOF	ras homolog family member F (in filopodia)	Upregulated
A_23_P115	0.012283	0.00418	2.21	PHYH	phytanoyl-CoA 2-hydroxylase	Upregulated
A_22_P000	0.000634	0.0000253	2.2	lnc-ANP32E-1	lnc-ANP32E-1:1	Upregulated
A_33_P346	0.000707	0.0000344	2.2	BFSP2-AS1	BFSP2 antisense RNA 1	Upregulated
A_23_P642	0.000741	0.0000395	2.2	ZPR1	ZPR1 zinc finger	Upregulated
A_23_P106	0.00083	0.0000519	2.2	MRPS23	mitochondrial ribosomal protein S23	Upregulated
A_33_P329	0.001055	0.0000899	2.2	MCTS2P	malignant T cell amplified sequence 2, pseudogene	Upregulated
A_23_P116	0.001097	0.0000982	2.2	MSANTD4	Myb/SANT-like DNA-binding domain containing 4 with coiled-coils	Upregulated
A_21_P001	0.001571	0.000196	2.2	ZNF605	zinc finger protein 605	Upregulated
A_22_P000	0.001887	0.00027	2.2	POFUT1	protein O-fucosyltransferase 1	Upregulated
A_33_P330	0.002853	0.000526	2.2	ZNFX1	zinc finger, NFX1-type containing 1	Upregulated
A_22_P000	0.003153	0.000613	2.2	SNHG15	small nucleolar RNA host gene 15 (non-protein coding)	Upregulated

A_33_P334	0.012122	0.00411	2.2	PBX3	pre-B-cell leukemia homeobox 3	Upregulated
A_33_P330	0.016795	0.00627	2.2	ANKFY1	ankyrin repeat and FYVE domain containing 1	Upregulated
A_33_P328	0.000612	0.0000234	2.19	C14orf2	chromosome 14 open reading frame 2	Upregulated
A_23_P738	0.000618	0.0000237	2.19	TCEAL1	transcription elongation factor A (SII)-like 1	Upregulated
A_23_P250	0.000802	0.000048	2.19	CDKN2AIPNL	CDKN2A interacting protein N-terminal like	Upregulated
A_33_P333	0.001797	0.000248	2.19	ARHGAP11B	Rho GTPase activating protein 11B	Upregulated
A_33_P321	0.002016	0.000305	2.19	ZBTB8OS	zinc finger and BTB domain containing 8 opposite strand	Upregulated
A_23_P135	0.013768	0.00483	2.19	SLCO5A1	solute carrier organic anion transporter family, member 5A1	Upregulated
A_24_P822	0.018003	0.00683	2.19	TMEM198	transmembrane protein 198	Upregulated
A_33_P353	0.00072	0.0000371	2.18	UBE2E4P	ubiquitin-conjugating enzyme E2E 4 pseudogene	Upregulated
A_24_P179	0.000883	0.000059	2.18	TPR	translocated promoter region, nuclear basket protein	Upregulated
A_22_P000	0.00094	0.0000681	2.18	lnc-C1QTNF8	lnc-C1QTNF8-4:1	Upregulated
A_23_P997	0.001216	0.000122	2.18	PNMA1	paraneoplastic Ma antigen 1	Upregulated
A_23_P340	0.001481	0.000177	2.18	RAB2A	RAB2A, member RAS oncogene family	Upregulated
A_21_P000	0.001953	0.000287	2.18	lnc-FAM182B	lnc-FAM182B-2:1	Upregulated
A_23_P379	0.003094	0.000596	2.18	FMNL3	formin-like 3	Upregulated
A_33_P324	0.003109	0.000601	2.18	N4BP2L1	NEDD4 binding protein 2-like 1	Upregulated
A_24_P269	0.005942	0.00156	2.18	SPRY4	sprouty homolog 4 (Drosophila)	Upregulated
A_23_P622	0.006094	0.00162	2.18	SCAF4	SR-related CTD-associated factor 4	Upregulated
A_33_P340	0.00811	0.00241	2.18	TCTEX1D4	Tctex1 domain containing 4	Upregulated
A_32_P206	0.000662	0.0000278	2.17	CKS1B	CDC28 protein kinase regulatory subunit 1B	Upregulated
A_24_P401	0.000678	0.0000296	2.17	ARHGAP17	Rho GTPase activating protein 17	Upregulated
A_33_P329	0.000695	0.0000314	2.17	CDCA7	cell division cycle associated 7	Upregulated
A_23_P391	0.000806	0.0000486	2.17	LIG1	ligase I, DNA, ATP-dependent	Upregulated
A_32_P526	0.000905	0.0000622	2.17	LPIN1	lipin 1	Upregulated
A_24_P958	0.00091	0.0000631	2.17	NPTN	neuroplastin	Upregulated
A_32_P869	0.000912	0.0000635	2.17	KGFLP2	keratinocyte growth factor-like protein 2	Upregulated
A_21_P001	0.000971	0.0000742	2.17	IGSF3	immunoglobulin superfamily, member 3	Upregulated
A_33_P325	0.001443	0.000168	2.17	DOK3	docking protein 3	Upregulated
A_22_P000	0.001476	0.000175	2.17	LOC1019277	uncharacterized LOC101927787	Upregulated
A_23_P345	0.002114	0.000331	2.17	SDCCAG8	serologically defined colon cancer antigen 8	Upregulated
A_33_P339	0.002342	0.000389	2.17	NREP	neuronal regeneration related protein	Upregulated
A_24_P336	0.00402	0.000879	2.17	RASSF5	Ras association (RalGDS/AF-6) domain family member 5	Upregulated
A_23_P714	0.004219	0.000946	2.17	WDYHV1	WDYHV motif containing 1	Upregulated
A_32_P203	0.005923	0.00155	2.17	ZNF30	zinc finger protein 30	Upregulated
A_21_P000	0.015222	0.00552	2.17	SCARNA8	small Cajal body-specific RNA 8	Upregulated
A_33_P323	0.000705	0.0000332	2.16	ASUN	asunder spermatogenesis regulator	Upregulated

A_23_P114	0.000949	0.0000697	2.16	MECR	mitochondrial trans-2-enoyl-CoA reductase	Upregulated
A_23_P145	0.000995	0.0000788	2.16	CEP162	centrosomal protein 162kDa	Upregulated
A_24_P636	0.001055	0.0000898	2.16	CCDC84	coiled-coil domain containing 84	Upregulated
A_23_P158	0.001066	0.0000923	2.16	SLC16A3	solute carrier family 16 (monocarboxylate transporter), member 3	Upregulated
A_21_P000	0.001603	0.000202	2.16	TMEM41B	transmembrane protein 41B	Upregulated
A_23_P207	0.001991	0.000298	2.16	KSR1	kinase suppressor of ras 1	Upregulated
A_32_P545	0.002454	0.000416	2.16	USP41	ubiquitin specific peptidase 41	Upregulated
A_33_P341	0.003304	0.00066	2.16	OR10A6	olfactory receptor, family 10, subfamily A, member 6 (gene/pseudogene	Upregulated
A_21_P000	0.003535	0.000729	2.16	lnc-AKR1E2-5	lnc-AKR1E2-5:1	Upregulated
A_23_P653	0.000664	0.0000283	2.15	GLRX5	glutaredoxin 5	Upregulated
A_23_P256	0.00074	0.0000394	2.15	SMARCA5	SWI/SNF related, matrix associated, actin dependent regulator of chrom	Upregulated
A_33_P332	0.000814	0.0000497	2.15	MCUR1	mitochondrial calcium uniporter regulator 1	Upregulated
A_33_P323	0.001397	0.000158	2.15	WDR44	WD repeat domain 44	Upregulated
A_23_P362	0.001534	0.000188	2.15	CSTF1	cleavage stimulation factor, 3' pre-RNA, subunit 1, 50kDa	Upregulated
A_23_P355	0.0017	0.000226	2.15	AFG3L1P	AFG3-like AAA ATPase 1, pseudogene	Upregulated
A_22_P000	0.002221	0.000359	2.15	LOC1005075	uncharacterized LOC100507577	Upregulated
A_22_P000	0.003572	0.000742	2.15	lnc-M6PR-3	lnc-M6PR-3:1	Upregulated
A_24_P226	0.007055	0.00199	2.15	CEP120	centrosomal protein 120kDa	Upregulated
A_23_P268	0.008594	0.00261	2.15	TUBD1	tubulin, delta 1	Upregulated
A_23_P212	0.000677	0.0000295	2.14	CEP135	centrosomal protein 135kDa	Upregulated
A_24_P360	0.000682	0.0000298	2.14	RNASET2	ribonuclease T2	Upregulated
A_23_P928	0.000832	0.0000522	2.14	LOC389765	kinesin family member 27 pseudogene	Upregulated
A_24_P169	0.000875	0.0000581	2.14	MAML2	mastermind-like 2 (Drosophila)	Upregulated
A_33_P338	0.001087	0.0000965	2.14	ORC2	origin recognition complex, subunit 2	Upregulated
A_23_P791	0.001595	0.0002	2.14	USE1	unconventional SNARE in the ER 1 homolog (S. cerevisiae)	Upregulated
A_23_P614	0.00304	0.00058	2.14	MSRA	methionine sulfoxide reductase A	Upregulated
A_23_P882	0.007422	0.00213	2.14	EMC9	ER membrane protein complex subunit 9	Upregulated
A_21_P000	0.00791	0.00233	2.14	TTC3P1	tetratricopeptide repeat domain 3 pseudogene 1	Upregulated
A_21_P000	0.00895	0.00275	2.14	lnc-GALC-3	lnc-GALC-3:4	Upregulated
A_23_P634	0.000695	0.0000315	2.13	GPSM2	G-protein signaling modulator 2	Upregulated
A_24_P329	0.000764	0.0000423	2.13	ORAI2	ORAI calcium release-activated calcium modulator 2	Upregulated
A_23_P101	0.000824	0.0000512	2.13	DOT1L	DOT1-like histone H3K79 methyltransferase	Upregulated
A_21_P000	0.001098	0.0000988	2.13	SNORA37	small nucleolar RNA, H/ACA box 37	Upregulated
A_33_P383	0.001661	0.000216	2.13	LOC401433	uncharacterized LOC401433	Upregulated
A_32_P158	0.002174	0.000348	2.13	ALG1L	ALG1, chitobiosyldiphosphodolichol beta-mannosyltransferase-like	Upregulated
A_32_P328	0.002685	0.000479	2.13	WDR33	WD repeat domain 33	Upregulated
A_22_P000	0.012507	0.00427	2.13	lnc-NPY2R-1	lnc-NPY2R-1:1	Upregulated

A_23_P171	0.000653	0.0000271	2.12	PHF6	PHD finger protein 6	Upregulated
A_23_P500	0.000679	0.0000296	2.12	TNFRSF8	tumor necrosis factor receptor superfamily, member 8	Upregulated
A_24_P732	0.000695	0.0000312	2.12	ATP2A2	ATPase, Ca++ transporting, cardiac muscle, slow twitch 2	Upregulated
A_23_P204	0.000707	0.0000335	2.12	DNM1L	dynamin 1-like	Upregulated
A_33_P371	0.000783	0.0000449	2.12	MIR146A	microRNA 146a	Upregulated
A_23_P134	0.000971	0.0000743	2.12	SOD2	superoxide dismutase 2, mitochondrial	Upregulated
A_23_P618	0.001154	0.00011	2.12	BAIAP2	BAI1-associated protein 2	Upregulated
A_23_P254	0.001695	0.000224	2.12	TATDN1	TatD DNase domain containing 1	Upregulated
A_24_P945	0.002104	0.000328	2.12	CYP4V2	cytochrome P450, family 4, subfamily V, polypeptide 2	Upregulated
A_22_P000	0.0038	0.000811	2.12	SAMSN1-AS1	SAMSN1 antisense RNA 1	Upregulated
A_24_P166	0.01198	0.00404	2.12	ZNRF1	zinc and ring finger 1, E3 ubiquitin protein ligase	Upregulated
A_24_P944	0.000733	0.0000385	2.11	SETD5	SET domain containing 5	Upregulated
A_33_P326	0.000779	0.0000443	2.11	NCK2	NCK adaptor protein 2	Upregulated
A_24_P941	0.000908	0.0000628	2.11	SPAST	spastin	Upregulated
A_22_P000	0.001375	0.000153	2.11	lnc-MFN1-1	lnc-MFN1-1:1	Upregulated
A_33_P336	0.001808	0.000251	2.11	CLN5	ceroid-lipofuscinosis, neuronal 5	Upregulated
A_32_P150	0.001808	0.000251	2.11	PPM1D	protein phosphatase, Mg2+/Mn2+ dependent, 1D	Upregulated
A_22_P000	0.002389	0.000401	2.11	SLC2A13	solute carrier family 2 (facilitated glucose transporter), member 13	Upregulated
A_21_P000	0.002532	0.000438	2.11	lnc-SLC12A8-	lnc-SLC12A8-1:1	Upregulated
A_33_P336	0.002738	0.000493	2.11	PIK3R2	phosphoinositide-3-kinase, regulatory subunit 2 (beta)	Upregulated
A_33_P322	0.003614	0.000754	2.11	LOC440896	uncharacterized LOC440896	Upregulated
A_21_P000	0.004101	0.000903	2.11	lnc-CD180-7	lnc-CD180-7:1	Upregulated
A_23_P121	0.005497	0.00139	2.11	HS3ST1	heparan sulfate (glucosamine) 3-O-sulfotransferase 1	Upregulated
A_21_P001	0.005759	0.00149	2.11	IGSF11-AS1	IGSF11 antisense RNA 1	Upregulated
A_22_P000	0.006321	0.00171	2.11	LOC1027239	uncharacterized LOC102723924	Upregulated
A_23_P986	0.008818	0.0027	2.11	HPS5	Hermansky-Pudlak syndrome 5	Upregulated
A_22_P000	0.014897	0.00537	2.11	lnc-CDK17-1	lnc-CDK17-1:1	Upregulated
A_24_P177	0.000806	0.0000486	2.1	PPP1R3F	protein phosphatase 1, regulatory subunit 3F	Upregulated
A_21_P000	0.000812	0.0000494	2.1	TMEM217	transmembrane protein 217	Upregulated
A_24_P243	0.000814	0.0000497	2.1	ZNF277	zinc finger protein 277	Upregulated
A_33_P324	0.001013	0.000082	2.1	PSG9	pregnancy specific beta-1-glycoprotein 9	Upregulated
A_21_P000	0.001244	0.000128	2.1	THRIL	TNF and HNRNPL related immunoregulatory long non-coding RNA	Upregulated
A_33_P354	0.00134	0.000146	2.1	PHF20L1	PHD finger protein 20-like 1	Upregulated
A_23_P717	0.001533	0.000187	2.1	MAMDC4	MAM domain containing 4	Upregulated
A_33_P327	0.001543	0.000189	2.1	CNOT6L	CCR4-NOT transcription complex, subunit 6-like	Upregulated
A_33_P326	0.001724	0.000231	2.1	CEP68	centrosomal protein 68kDa	Upregulated
A_24_P670	0.001907	0.000275	2.1	CLK2	CDC-like kinase 2	Upregulated

A_33_P332	0.007348	0.0021	2.1	PQBP1	polyglutamine binding protein 1	Upregulated
A_22_P000	0.018135	0.00689	2.1	lnc-OSBPL9-2	lnc-OSBPL9-2:1	Upregulated
A_24_P283	0.000717	0.0000364	2.09	PCMT1	protein-L-isoaspartate (D-aspartate) O-methyltransferase	Upregulated
A_23_P387	0.000842	0.0000534	2.09	ZNF830	zinc finger protein 830	Upregulated
A_23_P415	0.000927	0.0000655	2.09	HARS2	histidyl-tRNA synthetase 2, mitochondrial	Upregulated
A_23_P758	0.000954	0.0000704	2.09	TCF7	transcription factor 7 (T-cell specific, HMG-box)	Upregulated
A_24_P338	0.000957	0.0000709	2.09	STOML2	stomatin (EPB72)-like 2	Upregulated
A_33_P359	0.002568	0.000448	2.09	OR7E12P	olfactory receptor, family 7, subfamily E, member 12 pseudogene	Upregulated
A_23_P430	0.007133	0.00202	2.09	MTERF3	mitochondrial transcription termination factor 3	Upregulated
A_24_P370	0.014157	0.00502	2.09	ZNF230	zinc finger protein 230	Upregulated
A_22_P000	0.000695	0.0000313	2.08	NBPF11	neuroblastoma breakpoint family, member 11	Upregulated
A_33_P332	0.000752	0.0000409	2.08	CEND1	cell cycle exit and neuronal differentiation 1	Upregulated
A_22_P000	0.001047	0.000088	2.08	DONSON	downstream neighbor of SON	Upregulated
A_24_P106	0.001624	0.000207	2.08	WHSC1L1	Wolf-Hirschhorn syndrome candidate 1-like 1	Upregulated
A_21_P000	0.001634	0.00021	2.08	lnc-TULP4-1	lnc-TULP4-1:1	Upregulated
A_21_P001	0.001958	0.000289	2.08	XLOC_l2_013460		Upregulated
A_33_P320	0.001979	0.000295	2.08	PTAR1	protein prenyltransferase alpha subunit repeat containing 1	Upregulated
A_33_P340	0.004662	0.0011	2.08	PLET1	placenta expressed transcript 1	Upregulated
A_22_P000	0.006282	0.00169	2.08	SOX2-OT	SOX2 overlapping transcript	Upregulated
A_32_P123	0.006586	0.00181	2.08	TTC39C	tetratricopeptide repeat domain 39C	Upregulated
A_22_P000	0.009032	0.00279	2.08	lnc-FKBP14-1	lnc-FKBP14-1:1	Upregulated
A_21_P000	0.011018	0.00361	2.08	SNORD111B	small nucleolar RNA, C/D box 111B	Upregulated
A_22_P000	0.014247	0.00506	2.08	TTTY22	testis-specific transcript, Y-linked 22 (non-protein coding)	Upregulated
A_21_P001	0.000673	0.0000291	2.07	RPS6KB1	ribosomal protein S6 kinase, 70kDa, polypeptide 1	Upregulated
A_32_P109	0.000701	0.0000328	2.07	SFSWAP	splicing factor, suppressor of white-apricot family	Upregulated
A_23_P818	0.000706	0.0000334	2.07	DYNLT1	dynein, light chain, Tctex-type 1	Upregulated
A_24_P225	0.000707	0.0000338	2.07	MOB4	MOB family member 4, phocein	Upregulated
A_22_P000	0.000777	0.000044	2.07	LOC400541	uncharacterized LOC400541	Upregulated
A_24_P555	0.000966	0.0000734	2.07	PHF23	PHD finger protein 23	Upregulated
A_32_P132	0.000995	0.0000784	2.07	ZYG11A	zyg-11 family member A, cell cycle regulator	Upregulated
A_23_P121	0.0011	0.0000991	2.07	RAD18	RAD18 E3 ubiquitin protein ligase	Upregulated
A_23_P679	0.001555	0.000192	2.07	KLF7	Kruppel-like factor 7 (ubiquitous)	Upregulated
A_33_P329	0.001686	0.000221	2.07	TPRN	taperin	Upregulated
A_23_P212	0.002381	0.000399	2.07	ACAD11	acyl-CoA dehydrogenase family, member 11	Upregulated
A_32_P506	0.00249	0.000426	2.07	lnc-AP1S2-2	lnc-AP1S2-2:1	Upregulated
A_23_P521	0.005019	0.00122	2.07	ZNF124	zinc finger protein 124	Upregulated
A_23_P351	0.014529	0.00519	2.07	CLDN9	claudin 9	Upregulated

A_21_P000	0.021045	0.00827	2.07	lnc-PLP1-1	lnc-PLP1-1:1	Upregulated
A_22_P000	0.000709	0.0000349	2.06	lnc-SUMO1-1	lnc-SUMO1-1:1	Upregulated
A_23_P928	0.000913	0.0000639	2.06	RBM22	RNA binding motif protein 22	Upregulated
A_22_P000	0.000963	0.0000726	2.06	lnc-CD59-1	lnc-CD59-1:1	Upregulated
A_24_P230	0.001068	0.0000931	2.06	MIER1	mesoderm induction early response 1, transcriptional regulator	Upregulated
A_24_P169	0.001419	0.000163	2.06	ABCC4	ATP-binding cassette, sub-family C (CFTR/MRP), member 4	Upregulated
A_33_P322	0.004577	0.00107	2.06	SLC17A9	solute carrier family 17 (vesicular nucleotide transporter), member 9	Upregulated
A_33_P335	0.007112	0.00202	2.06	IFITM10	interferon induced transmembrane protein 10	Upregulated
A_32_P129	0.016044	0.0059	2.06	ERMARD	ER membrane-associated RNA degradation	Upregulated
A_33_P361	0.000698	0.0000322	2.05	ZBTB10	zinc finger and BTB domain containing 10	Upregulated
A_23_P134	0.000764	0.0000423	2.05	EPB41L2	erythrocyte membrane protein band 4.1-like 2	Upregulated
A_23_P411	0.000971	0.0000742	2.05	PPP4R2	protein phosphatase 4, regulatory subunit 2	Upregulated
A_33_P338	0.001214	0.000122	2.05	ZNF233	zinc finger protein 233	Upregulated
A_23_P105	0.001289	0.000136	2.05	WBP11	WW domain binding protein 11	Upregulated
A_33_P330	0.002015	0.000304	2.05	SETDB1	SET domain, bifurcated 1	Upregulated
A_23_P365	0.003018	0.000573	2.05	ITGA5	integrin, alpha 5 (fibronectin receptor, alpha polypeptide)	Upregulated
A_22_P000	0.004158	0.000924	2.05	lnc-C3orf71-1	lnc-C3orf71-1:2	Upregulated
A_24_P235	0.004677	0.0011	2.05	ABCA1	ATP-binding cassette, sub-family A (ABC1), member 1	Upregulated
A_33_P322	0.012488	0.00426	2.05	ARHGAP27	Rho GTPase activating protein 27	Upregulated
A_33_P325	0.000675	0.0000293	2.04	ITSN1	intersectin 1 (SH3 domain protein)	Upregulated
A_21_P001	0.0007	0.0000324	2.04	XLOC_l2_000727		Upregulated
A_23_P743	0.000739	0.000039	2.04	SCYL3	SCY1-like 3 (S. cerevisiae)	Upregulated
A_23_P128	0.000752	0.0000408	2.04	SOCS2	suppressor of cytokine signaling 2	Upregulated
A_33_P336	0.000776	0.0000439	2.04	MANEAL	mannosidase, endo-alpha-like	Upregulated
A_24_P665	0.000776	0.0000439	2.04	SRP9	signal recognition particle 9kDa	Upregulated
A_33_P331	0.000839	0.000053	2.04	FSTL3	follistatin-like 3 (secreted glycoprotein)	Upregulated
A_33_P336	0.001134	0.000106	2.04	C10orf88	chromosome 10 open reading frame 88	Upregulated
A_23_P478	0.001249	0.000129	2.04	STAT6	signal transducer and activator of transcription 6, interleukin-4 induced	Upregulated
A_23_P556	0.001398	0.000158	2.04	SFT2D3	SFT2 domain containing 3	Upregulated
A_33_P324	0.002681	0.000477	2.04	NFIC	nuclear factor I/C (CCAAT-binding transcription factor)	Upregulated
A_22_P000	0.004321	0.000981	2.04	lnc-RP11-33N	lnc-RP11-33N16.1.1-2:1	Upregulated
A_21_P000	0.016242	0.006	2.04	lnc-NHLRC3-1	lnc-NHLRC3-1:1	Upregulated
A_24_P319	0.000728	0.0000379	2.03	F11R	F11 receptor	Upregulated
A_24_P214	0.000883	0.0000591	2.03	TREML2	triggering receptor expressed on myeloid cells-like 2	Upregulated
A_32_P182	0.000991	0.0000768	2.03	ZNF511	zinc finger protein 511	Upregulated
A_23_P412	0.001197	0.000118	2.03	CLDN12	claudin 12	Upregulated
A_33_P328	0.001638	0.000211	2.03	SNX10	sorting nexin 10	Upregulated

A_23_P360	0.001657	0.000215	2.03	NEURL4	neuralized E3 ubiquitin protein ligase 4	Upregulated
A_33_P327	0.001677	0.000219	2.03	C20orf196	chromosome 20 open reading frame 196	Upregulated
A_22_P000	0.001784	0.000245	2.03	lnc-FAM13B-1	lnc-FAM13B-1:1	Upregulated
A_23_P406	0.001949	0.000286	2.03	MOSPD2	motile sperm domain containing 2	Upregulated
A_24_P186	0.00284	0.000522	2.03	ANAPC1	anaphase promoting complex subunit 1	Upregulated
A_22_P000	0.003265	0.000648	2.03	lnc-ZBTB32-1	lnc-ZBTB32-1:1	Upregulated
A_33_P341	0.004318	0.00098	2.03	ABL2	ABL proto-oncogene 2, non-receptor tyrosine kinase	Upregulated
A_33_P327	0.004411	0.00101	2.03	DOPEY1	dopey family member 1	Upregulated
A_23_P845	0.004467	0.00103	2.03	TOMM40L	translocase of outer mitochondrial membrane 40 homolog (yeast)-like	Upregulated
A_23_P369	0.004732	0.00112	2.03	RANBP10	RAN binding protein 10	Upregulated
A_23_P374	0.005741	0.00148	2.03	UBE2D4	ubiquitin-conjugating enzyme E2D 4 (putative)	Upregulated
A_22_P000	0.006654	0.00184	2.03	lnc-OR1Q1-1	lnc-OR1Q1-1:1	Upregulated
A_33_P327	0.021889	0.00869	2.03	NUP43	nucleoporin 43kDa	Upregulated
A_23_P111	0.000702	0.000033	2.02	BAG6	BCL2-associated athanogene 6	Upregulated
A_32_P634	0.000811	0.0000492	2.02	MAP2K4	mitogen-activated protein kinase kinase 4	Upregulated
A_23_P121	0.000938	0.0000672	2.02	FLVCR1	feline leukemia virus subgroup C cellular receptor 1	Upregulated
A_22_P000	0.002211	0.000356	2.02	PLLP	plasmolipin	Upregulated
A_23_P427	0.003564	0.000738	2.02	SPPL3	signal peptide peptidase like 3	Upregulated
A_33_P332	0.006155	0.00164	2.02	PLXNB2	plexin B2	Upregulated
A_23_P184	0.000707	0.0000339	2.01	MRPL3	mitochondrial ribosomal protein L3	Upregulated
A_32_P996	0.000867	0.0000573	2.01	HSF2	heat shock transcription factor 2	Upregulated
A_21_P001	0.000908	0.0000627	2.01	LOC389906	zinc finger protein 839 pseudogene	Upregulated
A_24_P888	0.001078	0.0000947	2.01	COX7A2L	cytochrome c oxidase subunit VIIa polypeptide 2 like	Upregulated
A_24_P101	0.012945	0.00446	2.01	CNOT1	CCR4-NOT transcription complex, subunit 1	Upregulated
A_23_P719	0.021038	0.00826	2.01	ERAL1	Era-like 12S mitochondrial rRNA chaperone 1	Upregulated
A_23_P207	0.000711	0.0000356	2	KIAA0100	KIAA0100	Upregulated
A_23_P300	0.000931	0.0000663	2	NFKB1	nuclear factor of kappa light polypeptide gene enhancer in B-cells 1	Upregulated
A_23_P212	0.000963	0.0000726	2	UBA7	ubiquitin-like modifier activating enzyme 7	Upregulated
A_32_P252	0.001193	0.000117	2	ISCA1	iron-sulfur cluster assembly 1	Upregulated
A_23_P160	0.001381	0.000154	2	GLRX2	glutaredoxin 2	Upregulated
A_32_P181	0.0016	0.000201	2	DXO	decapping exoribonuclease	Upregulated
A_23_P164	0.001663	0.000217	2	MED26	mediator complex subunit 26	Upregulated
A_23_P106	0.00185	0.000262	2	SULT1A2	sulfotransferase family, cytosolic, 1A, phenol-preferring, member 2	Upregulated
A_32_P199	0.002391	0.000402	2	SPTSSB	serine palmitoyltransferase, small subunit B	Upregulated
A_23_P781	0.003005	0.00057	2	ALDOC	aldolase C, fructose-bisphosphate	Upregulated
A_33_P337	0.006931	0.00194	2	DTNA	dystrobrevin, alpha	Upregulated
A_23_P198	0.008714	0.00266	2	IQCE	IQ motif containing E	Upregulated

A_23_P312	0.018595	0.0071	2	ARSK	arylsulfatase family, member K	Upregulated
A_23_P152	0.000709	0.0000349	1.99	DDX42	DEAD (Asp-Glu-Ala-Asp) box helicase 42	Upregulated
A_23_P803	0.000735	0.0000386	1.99	TOMM22	translocase of outer mitochondrial membrane 22 homolog (yeast)	Upregulated
A_23_P206	0.000885	0.0000595	1.99	KIAA0020	KIAA0020	Upregulated
A_23_P321	0.000938	0.0000673	1.99	LHX2	LIM homeobox 2	Upregulated
A_23_P510	0.001177	0.000114	1.99	ZNF142	zinc finger protein 142	Upregulated
A_23_P250	0.001222	0.000123	1.99	ISOC1	isochorismatase domain containing 1	Upregulated
A_24_P456	0.00335	0.000674	1.99	UTS2	urotensin 2	Upregulated
A_24_P117	0.003815	0.000815	1.99	INO80C	INO80 complex subunit C	Upregulated
A_33_P350	0.004238	0.000953	1.99	ZDHHC8P1	zinc finger, DHHC-type containing 8 pseudogene 1	Upregulated
A_23_P211	0.007089	0.00201	1.99	NRIP1	nuclear receptor interacting protein 1	Upregulated
A_24_P295	0.010142	0.00326	1.99	TSTD2	thiosulfate sulfurtransferase (rhodanese)-like domain containing 2	Upregulated
A_21_P000	0.000712	0.000036	1.98	SNORD93	small nucleolar RNA, C/D box 93	Upregulated
A_21_P000	0.000764	0.0000423	1.98	lnc-CLEC18B	lnc-CLEC18B-1:1	Upregulated
A_24_P192	0.000801	0.0000477	1.98	RALA	v-ral simian leukemia viral oncogene homolog A (ras related)	Upregulated
A_23_P384	0.001016	0.0000825	1.98	PHF23	PHD finger protein 23	Upregulated
A_23_P322	0.001149	0.000109	1.98	FAM177A1	family with sequence similarity 177, member A1	Upregulated
A_33_P336	0.001297	0.000138	1.98	NXT2	nuclear transport factor 2-like export factor 2	Upregulated
A_33_P338	0.001521	0.000184	1.98	DDB2	damage-specific DNA binding protein 2, 48kDa	Upregulated
A_22_P000	0.001831	0.000258	1.98	lnc-NT5DC2-1	lnc-NT5DC2-1:1	Upregulated
A_24_P115	0.012088	0.00409	1.98	EIF4EBP2	eukaryotic translation initiation factor 4E binding protein 2	Upregulated
A_23_P364	0.015515	0.00566	1.98	MIS18BP1	MIS18 binding protein 1	Upregulated
A_32_P170	0.000809	0.000049	1.97	TRAF3IP1	TNF receptor-associated factor 3 interacting protein 1	Upregulated
A_23_P801	0.001532	0.000187	1.97	RRP1	ribosomal RNA processing 1	Upregulated
A_24_P172	0.001543	0.000189	1.97	UBE2O	ubiquitin-conjugating enzyme E2O	Upregulated
A_21_P000	0.001562	0.000194	1.97	lnc-TRIP10-1	lnc-TRIP10-1:1	Upregulated
A_24_P398	0.002078	0.000322	1.97	COQ7	coenzyme Q7 homolog, ubiquinone (yeast)	Upregulated
A_21_P000	0.002101	0.000327	1.97	lnc-AKR1E2-5	lnc-AKR1E2-5:7	Upregulated
A_21_P001	0.015689	0.00573	1.97	LINC01127	long intergenic non-protein coding RNA 1127	Upregulated
A_24_P391	0.020261	0.00789	1.97	KCTD10	potassium channel tetramerization domain containing 10	Upregulated
A_33_P322	0.000741	0.0000396	1.96	HSD17B7	hydroxysteroid (17-beta) dehydrogenase 7	Upregulated
A_21_P000	0.001053	0.0000891	1.96	LINC01506	long intergenic non-protein coding RNA 1506	Upregulated
A_33_P329	0.001863	0.000265	1.96	ABCC5	ATP-binding cassette, sub-family C (CFTR/MRP), member 5	Upregulated
A_23_P150	0.001935	0.000282	1.96	C11orf68	chromosome 11 open reading frame 68	Upregulated
A_21_P000	0.002896	0.000538	1.96	SNORD96A	small nucleolar RNA, C/D box 96A	Upregulated
A_33_P330	0.004447	0.00102	1.96	SZT2	seizure threshold 2 homolog (mouse)	Upregulated
A_22_P000	0.012184	0.00413	1.96	lnc-NR2C2-1	lnc-NR2C2-1:1	Upregulated

A_23_P585	0.021337	0.00841	1.96	ERCC8	excision repair cross-complementation group 8	Upregulated
A_23_P126	0.000725	0.0000376	1.95	RAB29	RAB29, member RAS oncogene family	Upregulated
A_23_P130	0.000765	0.0000425	1.95	UTP18	UTP18 small subunit (SSU) processome component homolog (yeast)	Upregulated
A_23_P157	0.00082	0.0000508	1.95	DENND4C	DENN/MADD domain containing 4C	Upregulated
A_23_P149	0.0011	0.000099	1.95	COQ9	coenzyme Q9	Upregulated
A_24_P288	0.001432	0.000165	1.95	PIGA	phosphatidylinositol glycan anchor biosynthesis, class A	Upregulated
A_23_P255	0.00332	0.000666	1.95	TMEM185A	transmembrane protein 185A	Upregulated
A_32_P164	0.004602	0.00108	1.95	MRPL35	mitochondrial ribosomal protein L35	Upregulated
A_23_P217	0.005598	0.00143	1.95	COL4A6	collagen, type IV, alpha 6	Upregulated
A_23_P136	0.006443	0.00176	1.95	PDCD1	programmed cell death 1	Upregulated
A_23_P316	0.008344	0.00251	1.95	CCAR2	cell cycle and apoptosis regulator 2	Upregulated
A_22_P000	0.016221	0.00599	1.95	lnc-OIT3-1	lnc-OIT3-1:1	Upregulated
A_21_P001	0.017522	0.0066	1.95	XLOC_l2_015213		Upregulated
A_21_P001	0.021277	0.00838	1.95	CECR7	cat eye syndrome chromosome region, candidate 7 (non-protein coding	Upregulated
A_23_P202	0.000737	0.0000388	1.94	ABLIM1	actin binding LIM protein 1	Upregulated
A_24_P343	0.000766	0.0000428	1.94	DHFR	dihydrofolate reductase	Upregulated
A_33_P327	0.000781	0.0000446	1.94	ARID3A	AT rich interactive domain 3A (BRIGHT-like)	Upregulated
A_33_P341	0.000826	0.0000515	1.94	CLCC1	chloride channel CLIC-like 1	Upregulated
A_33_P330	0.000846	0.0000538	1.94	RBM20	RNA binding motif protein 20	Upregulated
A_33_P327	0.000887	0.0000596	1.94	NINJ1	ninjurin 1	Upregulated
A_24_P100	0.001137	0.000107	1.94	ZNF486	zinc finger protein 486	Upregulated
A_21_P000	0.00144	0.000167	1.94	lnc-AC004980	lnc-AC004980.12.1-1:1	Upregulated
A_21_P000	0.001691	0.000222	1.94	RBM34	RNA binding motif protein 34	Upregulated
A_24_P225	0.001698	0.000225	1.94	RRM2	ribonucleotide reductase M2	Upregulated
A_23_P250	0.003998	0.000873	1.94	WRN	Werner syndrome, RecQ helicase-like	Upregulated
A_33_P323	0.005643	0.00145	1.94	OR52E2	olfactory receptor, family 52, subfamily E, member 2	Upregulated
A_33_P321	0.000765	0.0000426	1.93	C5orf42	chromosome 5 open reading frame 42	Upregulated
A_33_P339	0.000798	0.0000472	1.93	CLLU1OS	chronic lymphocytic leukemia up-regulated 1 opposite strand	Upregulated
A_23_P134	0.000839	0.0000531	1.93	BNIP3L	BCL2/adenovirus E1B 19kDa interacting protein 3-like	Upregulated
A_33_P331	0.000848	0.0000542	1.93	GCH1	GTP cyclohydrolase 1	Upregulated
A_23_P411	0.000888	0.00006	1.93	SPRYD4	SPRY domain containing 4	Upregulated
A_23_P133	0.001163	0.000112	1.93	ALPK1	alpha-kinase 1	Upregulated
A_33_P321	0.001349	0.000148	1.93	NET1	neuroepithelial cell transforming 1	Upregulated
A_22_P000	0.001479	0.000176	1.93	lnc-C20orf94-	lnc-C20orf94-1:1	Upregulated
A_33_P337	0.001861	0.000265	1.93	PRICKLE3	prickle homolog 3 (Drosophila)	Upregulated
A_23_P454	0.001959	0.00029	1.93	GLA	galactosidase, alpha	Upregulated
A_33_P337	0.002243	0.000366	1.93	MRS2	MRS2 magnesium transporter	Upregulated

A_21_P001	0.003187	0.000626	1.93	HERC2P4	hect domain and RLD 2 pseudogene 4	Upregulated
A_22_P000	0.003388	0.000684	1.93	lnc-SLC12A7-	lnc-SLC12A7-1:9	Upregulated
A_23_P204	0.006166	0.00165	1.93	GIT2	G protein-coupled receptor kinase interacting ArfGAP 2	Upregulated
A_33_P333	0.012106	0.0041	1.93	SCYL2	SCY1-like 2 (S. cerevisiae)	Upregulated
A_33_P338	0.024076	0.0098	1.93	TRIM61	tripartite motif containing 61	Upregulated
A_23_P983	0.000827	0.0000516	1.92	BIRC3	baculoviral IAP repeat containing 3	Upregulated
A_23_P361	0.000986	0.0000763	1.92	CCND3	cyclin D3	Upregulated
A_23_P125	0.001022	0.0000837	1.92	ACOT9	acyl-CoA thioesterase 9	Upregulated
A_22_P000	0.001037	0.0000862	1.92	XIRP2-AS1	XIRP2 antisense RNA 1	Upregulated
A_23_P922	0.00104	0.0000866	1.92	TSC22D2	TSC22 domain family, member 2	Upregulated
A_23_P161	0.001046	0.0000879	1.92	OR7E24	olfactory receptor, family 7, subfamily E, member 24	Upregulated
A_23_P112	0.001085	0.0000957	1.92	ALG13	ALG13, UDP-N-acetylglucosaminyltransferase subunit	Upregulated
A_23_P103	0.001121	0.000104	1.92	LRRC8C	leucine rich repeat containing 8 family, member C	Upregulated
A_24_P187	0.001322	0.000142	1.92	RPAIN	RPA interacting protein	Upregulated
A_23_P383	0.001386	0.000155	1.92	HIC2	hypermethylated in cancer 2	Upregulated
A_23_P134	0.001763	0.00024	1.92	DUSP4	dual specificity phosphatase 4	Upregulated
A_23_P647	0.001959	0.00029	1.92	HCAR3	hydroxycarboxylic acid receptor 3	Upregulated
A_21_P001	0.001991	0.000298	1.92	XLOC_l2_007835		Upregulated
A_23_P379	0.00203	0.000309	1.92	PIGW	phosphatidylinositol glycan anchor biosynthesis, class W	Upregulated
A_23_P413	0.002693	0.000481	1.92	LYAR	Ly1 antibody reactive	Upregulated
A_32_P113	0.003684	0.000774	1.92	ZNF561	zinc finger protein 561	Upregulated
A_23_P373	0.004849	0.00116	1.92	PPFIBP1	PTPRF interacting protein, binding protein 1 (liprin beta 1)	Upregulated
A_33_P331	0.005444	0.00138	1.92	HSP90AB4P	heat shock protein 90kDa alpha (cytosolic), class B member 4, pseudog	Upregulated
A_23_P115	0.006071	0.00161	1.92	ZNF684	zinc finger protein 684	Upregulated
A_23_P430	0.00674	0.00187	1.92	ZBTB3	zinc finger and BTB domain containing 3	Upregulated
A_22_P000	0.011699	0.00392	1.92	CPB2-AS1	CPB2 antisense RNA 1	Upregulated
A_33_P339	0.019143	0.00737	1.92	CALML4	calmodulin-like 4	Upregulated
A_21_P000	0.028604	0.0122	1.92	lnc-GBP5-2	lnc-GBP5-2:2	Upregulated
A_22_P000	0.00093	0.000066	1.91	lnc-WDR45L-	lnc-WDR45L-1:1	Upregulated
A_22_P000	0.00108	0.000095	1.91	lnc-PEAK1.1-	lnc-PEAK1.1-2:1	Upregulated
A_33_P336	0.00134	0.000146	1.91	ATR	ATR serine/threonine kinase	Upregulated
A_23_P806	0.001403	0.000159	1.91	MFN1	mitofusin 1	Upregulated
A_24_P368	0.001719	0.00023	1.91	SLC25A26	solute carrier family 25 (S-adenosylmethionine carrier), member 26	Upregulated
A_33_P321	0.001808	0.000251	1.91	UPF2	UPF2 regulator of nonsense transcripts homolog (yeast)	Upregulated
A_22_P000	0.002	0.0003	1.91	SNHG15	small nucleolar RNA host gene 15 (non-protein coding)	Upregulated
A_23_P168	0.002167	0.000346	1.91	POMZP3	POM121 and ZP3 fusion	Upregulated
A_22_P000	0.002188	0.000351	1.91	GRAMD1A	GRAM domain containing 1A	Upregulated

A_22_P000	0.002631	0.000465	1.91	LOC1019276	uncharacterized LOC101927640	Upregulated
A_33_P327	0.002966	0.000557	1.91	CHM	choroideremia (Rab escort protein 1)	Upregulated
A_33_P330	0.004482	0.00104	1.91	CEP57L1	centrosomal protein 57kDa-like 1	Upregulated
A_33_P336	0.004946	0.00119	1.91	TNKS1BP1	tankyrase 1 binding protein 1, 182kDa	Upregulated
A_33_P337	0.007062	0.002	1.91	SESTD1	SEC14 and spectrin domains 1	Upregulated
A_24_P276	0.007622	0.00222	1.91	LRRC42	leucine rich repeat containing 42	Upregulated
A_33_P327	0.00825	0.00247	1.91	PHF20L1	PHD finger protein 20-like 1	Upregulated
A_23_P110	0.011822	0.00398	1.91	TRAF3IP2	TRAF3 interacting protein 2	Upregulated
A_23_P202	0.012661	0.00433	1.91	NOLC1	nucleolar and coiled-body phosphoprotein 1	Upregulated
A_23_P379	0.021637	0.00856	1.91	DHCR24	24-dehydrocholesterol reductase	Upregulated
A_33_P334	0.022799	0.00914	1.91	GINS4	GINS complex subunit 4 (Sld5 homolog)	Upregulated
A_24_P143	0.000839	0.0000532	1.9	PTBP1	polypyrimidine tract binding protein 1	Upregulated
A_22_P000	0.000847	0.000054	1.9	lnc-RP11-582	lnc-RP11-582J16.5.1-1:1	Upregulated
A_23_P152	0.000848	0.0000544	1.9	BCL2A1	BCL2-related protein A1	Upregulated
A_23_P102	0.001132	0.000105	1.9	MRPL53	mitochondrial ribosomal protein L53	Upregulated
A_19_P003	0.001134	0.000106	1.9	LINC-PINT	long intergenic non-protein coding RNA, p53 induced transcript	Upregulated
A_22_P000	0.001139	0.000107	1.9	LOC1019291	uncharacterized LOC101929124	Upregulated
A_24_P238	0.001141	0.000108	1.9	NSA2	NSA2 ribosome biogenesis homolog (S. cerevisiae)	Upregulated
A_23_P565	0.001453	0.00017	1.9	GEMIN6	gem (nuclear organelle) associated protein 6	Upregulated
A_24_P305	0.001637	0.000211	1.9	TMEM50B	transmembrane protein 50B	Upregulated
A_32_P451	0.0017	0.000226	1.9	IL6ST	interleukin 6 signal transducer	Upregulated
A_23_P550	0.00193	0.000281	1.9	FTSJ3	FtsJ homolog 3 (E. coli)	Upregulated
A_24_P408	0.002275	0.000373	1.9	ZNF587B	zinc finger protein 587B	Upregulated
A_33_P324	0.003028	0.000576	1.9	C2CD2	C2 calcium-dependent domain containing 2	Upregulated
A_23_P904	0.003228	0.000638	1.9	SARS2	seryl-tRNA synthetase 2, mitochondrial	Upregulated
A_33_P337	0.004217	0.000945	1.9	ZNF81	zinc finger protein 81	Upregulated
A_33_P329	0.004813	0.00115	1.9	TCEAL8	transcription elongation factor A (SII)-like 8	Upregulated
A_23_P320	0.005114	0.00125	1.9	SRXN1	sulfiredoxin 1	Upregulated
A_23_P553	0.006095	0.00162	1.9	KLHL11	kelch-like family member 11	Upregulated
A_23_P336	0.011364	0.00377	1.9	NOC2L	nucleolar complex associated 2 homolog (S. cerevisiae)	Upregulated
A_23_P912	0.028617	0.0122	1.9	CASS4	Cas scaffolding protein family member 4	Upregulated
A_33_P330	0.000763	0.000042	1.89	DGKZ	diacylglycerol kinase, zeta	Upregulated
A_21_P001	0.000858	0.0000561	1.89	LOC1019284	serine/threonine-protein kinase tousled-like 2-like	Upregulated
A_32_P366	0.000933	0.0000666	1.89	JAZF1	JAZF zinc finger 1	Upregulated
A_24_P982	0.001235	0.000126	1.89	EBAG9	estrogen receptor binding site associated, antigen, 9	Upregulated
A_23_P247	0.001437	0.000166	1.89	TTC9C	tetratricopeptide repeat domain 9C	Upregulated
A_24_P359	0.001544	0.00019	1.89	SLC6A6	solute carrier family 6 (neurotransmitter transporter), member 6	Upregulated

A_23_P129	0.001563	0.000194	1.89	DUSP3	dual specificity phosphatase 3	Upregulated
A_23_P301	0.00167	0.000218	1.89	ZNF572	zinc finger protein 572	Upregulated
A_24_P140	0.003709	0.000782	1.89	PXK	PX domain containing serine/threonine kinase	Upregulated
A_22_P000	0.005249	0.0013	1.89	lnc-COL6A2-1	lnc-COL6A2-1:1	Upregulated
A_33_P329	0.007299	0.00209	1.89	SPATA13	spermatogenesis associated 13	Upregulated
A_24_P416	0.012225	0.00415	1.89	PEX12	peroxisomal biogenesis factor 12	Upregulated
A_32_P263	0.000777	0.000044	1.88	POGLUT1	protein O-glucosyltransferase 1	Upregulated
A_33_P334	0.000863	0.0000566	1.88	ZNF43	zinc finger protein 43	Upregulated
A_23_P256	0.000876	0.0000583	1.88	FBXO30	F-box protein 30	Upregulated
A_23_P151	0.000938	0.0000678	1.88	LIMA1	LIM domain and actin binding 1	Upregulated
A_23_P252	0.001076	0.0000943	1.88	APTX	aprataxin	Upregulated
A_33_P329	0.001548	0.000191	1.88	CCDC174	coiled-coil domain containing 174	Upregulated
A_21_P001	0.002158	0.000343	1.88	LINC00869	long intergenic non-protein coding RNA 869	Upregulated
A_23_P756	0.002804	0.000511	1.88	HYOU1	hypoxia up-regulated 1	Upregulated
A_21_P000	0.003421	0.000694	1.88	LINC00673	long intergenic non-protein coding RNA 673	Upregulated
A_33_P341	0.003914	0.000847	1.88	ADAMTSL1	ADAMTS-like 1	Upregulated
A_33_P323	0.014445	0.00515	1.88	HMGB3	high mobility group box 3	Upregulated
A_23_P814	0.024907	0.0102	1.88	MAT2B	methionine adenosyltransferase II, beta	Upregulated
A_22_P000	0.025043	0.0103	1.88	lnc-IL6-2	lnc-IL6-2:2	Upregulated
A_32_P252	0.000786	0.0000454	1.87	HSPD1	heat shock 60kDa protein 1 (chaperonin)	Upregulated
A_23_P141	0.001314	0.000141	1.87	TNFSF13B	tumor necrosis factor (ligand) superfamily, member 13b	Upregulated
A_24_P317	0.001356	0.000149	1.87	LY6E	lymphocyte antigen 6 complex, locus E	Upregulated
A_33_P335	0.001529	0.000186	1.87	CSF1	colony stimulating factor 1 (macrophage)	Upregulated
A_23_P206	0.001692	0.000223	1.87	PRC1	protein regulator of cytokinesis 1	Upregulated
A_22_P000	0.002638	0.000466	1.87	lnc-NCL-2	lnc-NCL-2:1	Upregulated
A_33_P325	0.003875	0.000834	1.87	WDR5B	WD repeat domain 5B	Upregulated
A_21_P000	0.004955	0.00119	1.87	LOC1027244	uncharacterized LOC102724458	Upregulated
A_32_P118	0.013292	0.00461	1.87	DHRS4-AS1	DHRS4 antisense RNA 1	Upregulated
A_23_P255	0.014932	0.00539	1.87	RP9	retinitis pigmentosa 9 (autosomal dominant)	Upregulated
A_23_P130	0.021912	0.0087	1.87	ERCC2	excision repair cross-complementation group 2	Upregulated
A_24_P276	0.028727	0.0122	1.87	SPACA5	sperm acrosome associated 5	Upregulated
A_23_P140	0.000762	0.0000419	1.86	WBP4	WW domain binding protein 4	Upregulated
A_33_P357	0.000938	0.0000677	1.86	LOC158863	uncharacterized LOC158863	Upregulated
A_33_P334	0.00098	0.0000757	1.86	PIKFYVE	phosphoinositide kinase, FYVE finger containing	Upregulated
A_33_P330	0.001299	0.000139	1.86	BLCAP	bladder cancer associated protein	Upregulated
A_33_P337	0.001499	0.00018	1.86	LOC392364	nuclear pore associated protein 1 pseudogene	Upregulated
A_21_P001	0.001633	0.00021	1.86	LOC1019269	uncharacterized LOC101926936	Upregulated

A_23_P431	0.003032	0.000578	1.86	C14orf80	chromosome 14 open reading frame 80	Upregulated
A_19_P008	0.003155	0.000615	1.86	SNHG20	small nucleolar RNA host gene 20 (non-protein coding)	Upregulated
A_33_P321	0.011144	0.00367	1.86	GALNT6	polypeptide N-acetylgalactosaminyltransferase 6	Upregulated
A_23_P103	0.019852	0.0077	1.86	OSCP1	organic solute carrier partner 1	Upregulated
A_33_P328	0.034167	0.0151	1.86	ATP9B	ATPase, class II, type 9B	Upregulated
A_23_P438	0.000766	0.0000428	1.85	LAX1	lymphocyte transmembrane adaptor 1	Upregulated
A_24_P253	0.000793	0.0000466	1.85	SLC7A1	solute carrier family 7 (cationic amino acid transporter, y+ system), memb	Upregulated
A_23_P161	0.000863	0.0000566	1.85	CWF19L2	CWF19-like 2, cell cycle control (S. pombe)	Upregulated
A_24_P124	0.001036	0.000086	1.85	TRAM2	translocation associated membrane protein 2	Upregulated
A_23_P226	0.001107	0.000101	1.85	VAMP7	vesicle-associated membrane protein 7	Upregulated
A_33_P334	0.001216	0.000122	1.85	TMEM45A	transmembrane protein 45A	Upregulated
A_23_P757	0.001754	0.000238	1.85	MYRF	myelin regulatory factor	Upregulated
A_23_P218	0.002224	0.00036	1.85	NARF	nuclear prelamin A recognition factor	Upregulated
A_22_P000	0.002528	0.000438	1.85	lnc-FAM40A-1	lnc-FAM40A-1:1	Upregulated
A_23_P512	0.004335	0.000986	1.85	PARS2	prolyl-tRNA synthetase 2, mitochondrial (putative)	Upregulated
A_33_P341	0.005502	0.0014	1.85	NCCRP1	non-specific cytotoxic cell receptor protein 1 homolog (zebrafish)	Upregulated
A_23_P999	0.005797	0.00151	1.85	TIPIN	TIMELESS interacting protein	Upregulated
A_33_P332	0.006632	0.00183	1.85	ESCO2	establishment of sister chromatid cohesion N-acetyltransferase 2	Upregulated
A_33_P326	0.007887	0.00232	1.85	ZNF616	zinc finger protein 616	Upregulated
A_32_P480	0.008458	0.00255	1.85	EYS	eyes shut homolog (Drosophila)	Upregulated
A_23_P589	0.010635	0.00346	1.85	NQO2	NAD(P)H dehydrogenase, quinone 2	Upregulated
A_21_P001	0.012852	0.00442	1.85	LINC01128	long intergenic non-protein coding RNA 1128	Upregulated
A_23_P298	0.000781	0.0000446	1.84	USO1	USO1 vesicle transport factor	Upregulated
A_32_P109	0.000814	0.0000499	1.84	ANKRD20A12	ankyrin repeat domain 20 family, member A12, pseudogene	Upregulated
A_24_P150	0.000824	0.0000513	1.84	KLHL18	kelch-like family member 18	Upregulated
A_33_P323	0.000853	0.000055	1.84	MLLT4	myeloid/lymphoid or mixed-lineage leukemia (trithorax homolog, Drosoph	Upregulated
A_33_P323	0.000912	0.0000636	1.84	NSDHL	NAD(P) dependent steroid dehydrogenase-like	Upregulated
A_21_P000	0.000974	0.0000748	1.84	RAB6C-AS1	RAB6C antisense RNA 1	Upregulated
A_23_P395	0.001004	0.0000806	1.84	KDM3A	lysine (K)-specific demethylase 3A	Upregulated
A_23_P845	0.001067	0.0000928	1.84	LFNG	LFNG O-fucosylpeptide 3-beta-N-acetylglucosaminyltransferase	Upregulated
A_23_P397	0.002459	0.000417	1.84	KLC4	kinesin light chain 4	Upregulated
A_24_P206	0.0032	0.00063	1.84	PDE1C	phosphodiesterase 1C, calmodulin-dependent 70kDa	Upregulated
A_33_P333	0.004149	0.00092	1.84	PDLIM5	PDZ and LIM domain 5	Upregulated
A_23_P373	0.004819	0.00115	1.84	PATL1	protein associated with topoisomerase II homolog 1 (yeast)	Upregulated
A_23_P130	0.006012	0.00159	1.84	ELOF1	elongation factor 1 homolog (S. cerevisiae)	Upregulated
A_32_P440	0.006067	0.00161	1.84	C16orf72	chromosome 16 open reading frame 72	Upregulated
A_33_P329	0.006315	0.0017	1.84	WDR5	WD repeat domain 5	Upregulated

A_19_P008	0.007301	0.00209	1.84	LOC1019295	uncharacterized LOC101929580	Upregulated
A_24_P219	0.009235	0.00287	1.84	GPALPP1	GPALPP motifs containing 1	Upregulated
A_33_P326	0.015684	0.00573	1.84	ZNF280D	zinc finger protein 280D	Upregulated
A_33_P331	0.000837	0.0000526	1.83	CAB39	calcium binding protein 39	Upregulated
A_33_P338	0.000884	0.0000593	1.83	CDT1	chromatin licensing and DNA replication factor 1	Upregulated
A_33_P322	0.000901	0.0000613	1.83	ZNF331	zinc finger protein 331	Upregulated
A_23_P626	0.001171	0.000113	1.83	PPT1	palmitoyl-protein thioesterase 1	Upregulated
A_24_P217	0.001203	0.000119	1.83	MFAP3	microfibrillar-associated protein 3	Upregulated
A_22_P000	0.001266	0.000133	1.83	TSPAN33	tetraspanin 33	Upregulated
A_23_P144	0.001289	0.000136	1.83	EXOC5	exocyst complex component 5	Upregulated
A_23_P164	0.001476	0.000175	1.83	ZNF225	zinc finger protein 225	Upregulated
A_23_P130	0.001772	0.000242	1.83	TBC1D17	TBC1 domain family, member 17	Upregulated
A_21_P001	0.002128	0.000336	1.83	XLOC_l2_005179		Upregulated
A_33_P341	0.002579	0.000452	1.83	KLF1	Kruppel-like factor 1 (erythroid)	Upregulated
A_23_P520	0.011238	0.00371	1.83	TARBP1	TAR (HIV-1) RNA binding protein 1	Upregulated
A_23_P256	0.021599	0.00854	1.83	KBTBD7	kelch repeat and BTB (POZ) domain containing 7	Upregulated
A_23_P165	0.000828	0.0000517	1.82	SLC20A1	solute carrier family 20 (phosphate transporter), member 1	Upregulated
A_22_P000	0.000857	0.0000558	1.82	LOC1019277	uncharacterized LOC101927735	Upregulated
A_23_P873	0.000859	0.0000562	1.82	NAT10	N-acetyltransferase 10 (GCN5-related)	Upregulated
A_33_P342	0.000959	0.0000713	1.82	PPP1R3D	protein phosphatase 1, regulatory subunit 3D	Upregulated
A_33_P340	0.001267	0.000133	1.82	TRAK1	trafficking protein, kinesin binding 1	Upregulated
A_33_P331	0.001668	0.000217	1.82	PGAP1	post-GPI attachment to proteins 1	Upregulated
A_32_P107	0.004084	0.000898	1.82	SPATA33	spermatogenesis associated 33	Upregulated
A_22_P000	0.010272	0.00331	1.82	LOC1019278	uncharacterized LOC101927835	Upregulated
A_21_P001	0.000853	0.0000549	1.81	DBNDD2	dysbindin (dystrobrevin binding protein 1) domain containing 2	Upregulated
A_24_P407	0.000942	0.0000685	1.81	PPM1A	protein phosphatase, Mg2+/Mn2+ dependent, 1A	Upregulated
A_23_P110	0.000995	0.0000792	1.81	PPWD1	peptidylprolyl isomerase domain and WD repeat containing 1	Upregulated
A_23_P259	0.001062	0.0000909	1.81	SEC22A	SEC22 vesicle trafficking protein homolog A (S. cerevisiae)	Upregulated
A_22_P000	0.001087	0.0000965	1.81	lnc-KCNC2-1	lnc-KCNC2-1:1	Upregulated
A_33_P336	0.001107	0.000101	1.81	ERLIN2	ER lipid raft associated 2	Upregulated
A_22_P000	0.00165	0.000213	1.81	lnc-PHYHIP-2	lnc-PHYHIP-2:1	Upregulated
A_23_P106	0.004433	0.00102	1.81	L2HGDH	L-2-hydroxyglutarate dehydrogenase	Upregulated
A_23_P101	0.019256	0.00742	1.81	SLC5A5	solute carrier family 5 (sodium/iodide cotransporter), member 5	Upregulated
A_23_P147	0.000819	0.0000506	1.8	LYN	LYN proto-oncogene, Src family tyrosine kinase	Upregulated
A_21_P000	0.000837	0.0000527	1.8	RPL32	ribosomal protein L32	Upregulated
A_33_P325	0.000998	0.0000795	1.8	C12orf5	chromosome 12 open reading frame 5	Upregulated
A_23_P656	0.001235	0.000125	1.8	WARS	tryptophanyl-tRNA synthetase	Upregulated

A_33_P326	0.001254	0.00013	1.8	SLC17A5	solute carrier family 17 (acidic sugar transporter), member 5	Upregulated
A_22_P000	0.001563	0.000194	1.8	LOC1019272	uncharacterized LOC101927287	Upregulated
A_32_P194	0.002078	0.000322	1.8	CLEC16A	C-type lectin domain family 16, member A	Upregulated
A_23_P358	0.003716	0.000785	1.8	DNAJC16	DnaJ (Hsp40) homolog, subfamily C, member 16	Upregulated
A_23_P122	0.000817	0.0000503	1.79	AUTS2	autism susceptibility candidate 2	Upregulated
A_33_P335	0.000858	0.000056	1.79	IDH3B	isocitrate dehydrogenase 3 (NAD+) beta	Upregulated
A_32_P108	0.000904	0.0000618	1.79	AK4	adenylate kinase 4	Upregulated
A_23_P401	0.001021	0.0000833	1.79	CENPF	centromere protein F, 350/400kDa	Upregulated
A_32_P838	0.001262	0.000132	1.79	HEY1	hes-related family bHLH transcription factor with YRPW motif 1	Upregulated
A_33_P337	0.001813	0.000253	1.79	PAAF1	proteasomal ATPase-associated factor 1	Upregulated
A_33_P325	0.002293	0.000377	1.79	ACAT1	acetyl-CoA acetyltransferase 1	Upregulated
A_23_P201	0.00246	0.000418	1.79	PPOX	protoporphyrinogen oxidase	Upregulated
A_33_P336	0.004087	0.000899	1.79	HIST1H2AI	histone cluster 1, H2ai	Upregulated
A_23_P425	0.006045	0.0016	1.79	PPIL4	peptidylprolyl isomerase (cyclophilin)-like 4	Upregulated
A_22_P000	0.008304	0.00249	1.79	lnc-SGSH-1	lnc-SGSH-1:1	Upregulated
A_22_P000	0.009711	0.00307	1.79	lnc-DGAT2-1	lnc-DGAT2-1:1	Upregulated
A_21_P001	0.010554	0.00342	1.79	XLOC_l2_004563		Upregulated
A_21_P001	0.012623	0.00432	1.79	LOC1027246	uncharacterized LOC102724630	Upregulated
A_23_P361	0.016054	0.00591	1.79	RIMKLB	ribosomal modification protein rimK-like family member B	Upregulated
A_33_P332	0.025028	0.0103	1.79	ETV3	ets variant 3	Upregulated
A_21_P000	0.000858	0.000056	1.78	SNORD72	small nucleolar RNA, C/D box 72	Upregulated
A_33_P322	0.001067	0.0000927	1.78	IL17RA	interleukin 17 receptor A	Upregulated
A_32_P170	0.001198	0.000118	1.78	STAG3	stromal antigen 3	Upregulated
A_24_P302	0.001523	0.000185	1.78	CLCN6	chloride channel, voltage-sensitive 6	Upregulated
A_24_P376	0.001753	0.000237	1.78	UBA2	ubiquitin-like modifier activating enzyme 2	Upregulated
A_21_P001	0.001903	0.000274	1.78	XLOC_l2_005438		Upregulated
A_23_P651	0.002125	0.000336	1.78	SPRYD3	SPRY domain containing 3	Upregulated
A_21_P000	0.002889	0.000536	1.78	PCNA-AS1	PCNA antisense RNA 1	Upregulated
A_23_P233	0.005917	0.00155	1.78	EXO1	exonuclease 1	Upregulated
A_21_P000	0.006908	0.00193	1.78	lnc-IL6-1	lnc-IL6-1:1	Upregulated
A_21_P000	0.006908	0.00193	1.78	lnc-SLITRK6-	lnc-SLITRK6-12:2	Upregulated
A_22_P000	0.007111	0.00202	1.78	lnc-RP11-791	lnc-RP11-791J7.2.1-1:1	Upregulated
A_23_P148	0.019866	0.00771	1.78	NELFB	negative elongation factor complex member B	Upregulated
A_24_P491	0.026617	0.0111	1.78	EXD3	exonuclease 3'-5' domain containing 3	Upregulated
A_21_P000	0.045304	0.0213	1.78	lnc-ZNF727-2	lnc-ZNF727-2:1	Upregulated
A_33_P329	0.000866	0.0000571	1.77	RAB2B	RAB2B, member RAS oncogene family	Upregulated
A_33_P338	0.000995	0.0000786	1.77	ZNF331	zinc finger protein 331	Upregulated

A_21_P001	0.00106	0.0000904	1.77	CHTF8	CTF8, chromosome transmission fidelity factor 8 homolog (S. cerevisiae)	Upregulated
A_23_P209	0.001133	0.000106	1.77	NAB1	NGFI-A binding protein 1 (EGR1 binding protein 1)	Upregulated
A_23_P380	0.001347	0.000147	1.77	KIAA1279	KIAA1279	Upregulated
A_23_P790	0.001745	0.000235	1.77	LILRA3	leukocyte immunoglobulin-like receptor, subfamily A (without TM domain	Upregulated
A_21_P001	0.003191	0.000627	1.77	HIST1H4H	histone cluster 1, H4h	Upregulated
A_24_P341	0.004269	0.000963	1.77	EPG5	ectopic P-granules autophagy protein 5 homolog (C. elegans)	Upregulated
A_23_P397	0.005117	0.00126	1.77	FEZ2	fasciculation and elongation protein zeta 2 (zygin II)	Upregulated
A_22_P000	0.00628	0.00169	1.77	lnc-CNOT1-1	lnc-CNOT1-1:1	Upregulated
A_23_P312	0.013653	0.00477	1.77	ITGAX	integrin, alpha X (complement component 3 receptor 4 subunit)	Upregulated
A_32_P191	0.013665	0.00478	1.77	SETD8	SET domain containing (lysine methyltransferase) 8	Upregulated
A_23_P299	0.014597	0.00523	1.77	IL15	interleukin 15	Upregulated
A_24_P414	0.015734	0.00575	1.77	PLN	phospholamban	Upregulated
A_32_P121	0.02125	0.00837	1.77	IQCF1	IQ motif containing F1	Upregulated
A_21_P000	0.028707	0.0122	1.77	TMEM161B-A	TMEM161B antisense RNA 1	Upregulated
A_21_P001	0.037982	0.0173	1.77	POLR2J4	polymerase (RNA) II (DNA directed) polypeptide J4, pseudogene	Upregulated
A_19_P008	0.000858	0.000056	1.76	ANKRD33B	ankyrin repeat domain 33B	Upregulated
A_23_P252	0.000998	0.0000797	1.76	SLC2A8	solute carrier family 2 (facilitated glucose transporter), member 8	Upregulated
A_33_P328	0.001108	0.000101	1.76	LENG8	leukocyte receptor cluster (LRC) member 8	Upregulated
A_33_P788	0.00126	0.000131	1.76	TSIX	TSIX transcript, XIST antisense RNA	Upregulated
A_23_P381	0.001625	0.000207	1.76	SP2	Sp2 transcription factor	Upregulated
A_32_P224	0.001692	0.000223	1.76	FKBP15	FK506 binding protein 15, 133kDa	Upregulated
A_22_P000	0.002482	0.000424	1.76	LOC1019294	uncharacterized LOC101929441	Upregulated
A_23_P101	0.002851	0.000525	1.76	MRPL54	mitochondrial ribosomal protein L54	Upregulated
A_33_P322	0.002945	0.000551	1.76	LOC1001289	uncharacterized LOC100128908	Upregulated
A_23_P550	0.003472	0.000709	1.76	NOL11	nucleolar protein 11	Upregulated
A_33_P321	0.004478	0.00103	1.76	ABHD15	abhydrolase domain containing 15	Upregulated
A_23_P138	0.004962	0.0012	1.76	ARHGAP19	Rho GTPase activating protein 19	Upregulated
A_33_P323	0.016195	0.00598	1.76	TMEM234	transmembrane protein 234	Upregulated
A_33_P323	0.023572	0.00954	1.76	TET3	tet methylcytosine dioxygenase 3	Upregulated
A_23_P926	0.028278	0.012	1.76	ANKHD1	ankyrin repeat and KH domain containing 1	Upregulated
A_21_P000	0.030051	0.0129	1.76	lnc-TERF2IP-	lnc-TERF2IP-1:1	Upregulated
A_33_P337	0.032182	0.0141	1.76	NOM1	nucleolar protein with MIF4G domain 1	Upregulated
A_21_P000	0.060178	0.03	1.76	lnc-SORCS1-	lnc-SORCS1-1:1	Upregulated
A_24_P377	0.000951	0.00007	1.75	HSPA4	heat shock 70kDa protein 4	Upregulated
A_23_P387	0.001021	0.0000834	1.75	CASP2	caspase 2, apoptosis-related cysteine peptidase	Upregulated
A_23_P242	0.001096	0.000098	1.75	IDI2-AS1	IDI2 antisense RNA 1	Upregulated
A_21_P000	0.001161	0.000111	1.75	SNORA54	small nucleolar RNA, H/ACA box 54	Upregulated

A_23_P127	0.001187	0.000116	1.75	DNAJC1	DnaJ (Hsp40) homolog, subfamily C, member 1	Upregulated
A_33_P323	0.001363	0.00015	1.75	ZSWIM4	zinc finger, SWIM-type containing 4	Upregulated
A_23_P112	0.001387	0.000155	1.75	RMI1	RecQ mediated genome instability 1	Upregulated
A_33_P336	0.001394	0.000157	1.75	RTN3	reticulon 3	Upregulated
A_23_P160	0.001697	0.000225	1.75	SLC2A5	solute carrier family 2 (facilitated glucose/fructose transporter), member !	Upregulated
A_21_P000	0.002311	0.000381	1.75	RNU6ATAC	RNA, U6atac small nuclear (U12-dependent splicing)	Upregulated
A_23_P431	0.003239	0.000641	1.75	KBTBD8	kelch repeat and BTB (POZ) domain containing 8	Upregulated
A_23_P182	0.004452	0.00103	1.75	NPRL2	nitrogen permease regulator-like 2 (S. cerevisiae)	Upregulated
A_22_P000	0.004745	0.00113	1.75	LOC1019275	uncharacterized LOC101927543	Upregulated
A_23_P365	0.005136	0.00126	1.75	CAMTA2	calmodulin binding transcription activator 2	Upregulated
A_23_P159	0.005204	0.00129	1.75	GPR50	G protein-coupled receptor 50	Upregulated
A_33_P324	0.008227	0.00246	1.75	C3orf84	chromosome 3 open reading frame 84	Upregulated
A_22_P000	0.019642	0.0076	1.75	ALMS1-IT1	ALMS1 intronic transcript 1 (non-protein coding)	Upregulated
A_33_P323	0.022366	0.00892	1.75	BMP1	bone morphogenetic protein 1	Upregulated
A_23_P259	0.033269	0.0146	1.75	DNAL1	dynein, axonemal, light chain 1	Upregulated
A_33_P325	0.000836	0.0000525	1.74	MAPK8	mitogen-activated protein kinase 8	Upregulated
A_33_P329	0.00091	0.0000632	1.74	LPCAT3	lysophosphatidylcholine acyltransferase 3	Upregulated
A_33_P337	0.001043	0.0000874	1.74	CCL19	chemokine (C-C motif) ligand 19	Upregulated
A_23_P432	0.001067	0.0000926	1.74	SLC25A40	solute carrier family 25, member 40	Upregulated
A_32_P514	0.001101	0.0000996	1.74	UNK	unkempt family zinc finger	Upregulated
A_33_P331	0.00144	0.000167	1.74	TREM1	triggering receptor expressed on myeloid cells 1	Upregulated
A_22_P000	0.001663	0.000216	1.74	lnc-SGTB-2	lnc-SGTB-2:1	Upregulated
A_33_P361	0.002709	0.000486	1.74	PRKXP1	protein kinase, X-linked, pseudogene 1	Upregulated
A_23_P126	0.00275	0.000497	1.74	IL10	interleukin 10	Upregulated
A_23_P101	0.003476	0.00071	1.74	LPAR2	lysophosphatidic acid receptor 2	Upregulated
A_23_P657	0.00382	0.000817	1.74	DIS3L	DIS3 like exosome 3'-5' exoribonuclease	Upregulated
A_33_P324	0.00393	0.000851	1.74	HAAO	3-hydroxyanthranilate 3,4-dioxygenase	Upregulated
A_23_P206	0.005191	0.00128	1.74	MYH11	myosin, heavy chain 11, smooth muscle	Upregulated
A_23_P105	0.005382	0.00136	1.74	ROM1	retinal outer segment membrane protein 1	Upregulated
A_32_P103	0.000954	0.0000705	1.73	FAM60A	family with sequence similarity 60, member A	Upregulated
A_23_P203	0.00097	0.0000739	1.73	SMPD1	sphingomyelin phosphodiesterase 1, acid lysosomal	Upregulated
A_33_P323	0.000971	0.0000743	1.73	PREP	prolyl endopeptidase	Upregulated
A_23_P118	0.001014	0.0000821	1.73	TOP2A	topoisomerase (DNA) II alpha 170kDa	Upregulated
A_23_P338	0.001068	0.0000931	1.73	CD274	CD274 molecule	Upregulated
A_24_P115	0.001124	0.000104	1.73	BIRC2	baculoviral IAP repeat containing 2	Upregulated
A_22_P000	0.001476	0.000175	1.73	lnc-TNFRSF1	lnc-TNFRSF17-1:1	Upregulated
A_33_P333	0.001635	0.00021	1.73	TRPC2	transient receptor potential cation channel, subfamily C, member 2, pse	Upregulated

A_23_P258	0.001727	0.000232	1.73	MYL5	myosin, light chain 5, regulatory	Upregulated
A_23_P355	0.002219	0.000358	1.73	SYNPO2L	synaptopodin 2-like	Upregulated
A_33_P322	0.002957	0.000555	1.73	LRRC37A8P	leucine rich repeat containing 37, member A8, pseudogene	Upregulated
A_22_P000	0.00419	0.000934	1.73	PPIEL	peptidylprolyl isomerase E-like pseudogene	Upregulated
A_33_P326	0.00451	0.00105	1.73	OR2T34	olfactory receptor, family 2, subfamily T, member 34	Upregulated
A_33_P339	0.004595	0.00107	1.73	C15orf54	chromosome 15 open reading frame 54	Upregulated
A_23_P108	0.006203	0.00166	1.73	WDSUB1	WD repeat, sterile alpha motif and U-box domain containing 1	Upregulated
A_24_P192	0.007499	0.00217	1.73	WRNIP1	Werner helicase interacting protein 1	Upregulated
A_33_P334	0.022388	0.00893	1.73	RPS6KA2	ribosomal protein S6 kinase, 90kDa, polypeptide 2	Upregulated
A_22_P000	0.059603	0.0296	1.73	lnc-MYCT1-1	lnc-MYCT1-1:1	Upregulated
A_32_P215	0.000963	0.0000726	1.72	GPSM1	G-protein signaling modulator 1	Upregulated
A_33_P335	0.000963	0.0000726	1.72	LINC00992	long intergenic non-protein coding RNA 992	Upregulated
A_33_P334	0.001022	0.0000836	1.72	EEF2K	eukaryotic elongation factor 2 kinase	Upregulated
A_33_P338	0.001116	0.000102	1.72	CD274	CD274 molecule	Upregulated
A_33_P323	0.00124	0.000127	1.72	SRSF6	serine/arginine-rich splicing factor 6	Upregulated
A_23_P224	0.001453	0.00017	1.72	RP2	retinitis pigmentosa 2 (X-linked recessive)	Upregulated
A_23_P161	0.00161	0.000204	1.72	NAALAD2	N-acetylated alpha-linked acidic dipeptidase 2	Upregulated
A_23_P321	0.001687	0.000221	1.72	DENND5A	DENN/MADD domain containing 5A	Upregulated
A_21_P001	0.001688	0.000222	1.72	XLOC_l2_007135		Upregulated
A_33_P331	0.00174	0.000234	1.72	CMPK1	cytidine monophosphate (UMP-CMP) kinase 1, cytosolic	Upregulated
A_23_P149	0.001986	0.000297	1.72	LINC00115	long intergenic non-protein coding RNA 115	Upregulated
A_33_P328	0.002006	0.000302	1.72	ANKRD20A8I	ankyrin repeat domain 20 family, member A8, pseudogene	Upregulated
A_21_P000	0.003109	0.000601	1.72	SNORA18	small nucleolar RNA, H/ACA box 18	Upregulated
A_23_P115	0.003134	0.000609	1.72	VASH2	vasohibin 2	Upregulated
A_24_P210	0.00321	0.000633	1.72	PSMG3	proteasome (prosome, macropain) assembly chaperone 3	Upregulated
A_24_P156	0.003497	0.000716	1.72	EBF1	early B-cell factor 1	Upregulated
A_23_P131	0.004031	0.000882	1.72	ZBTB32	zinc finger and BTB domain containing 32	Upregulated
A_22_P000	0.00761	0.00221	1.72	lnc-IL21R-1	lnc-IL21R-1:1	Upregulated
A_24_P417	0.009799	0.00311	1.72	FLYWCH2	FLYWCH family member 2	Upregulated
A_21_P001	0.011157	0.00368	1.72	XLOC_l2_002281		Upregulated
A_24_P118	0.01385	0.00487	1.72	ATP5S	ATP synthase, H+ transporting, mitochondrial Fo complex, subunit s (fac	Upregulated
A_33_P339	0.014654	0.00526	1.72	LOC1001298	uncharacterized LOC100129884	Upregulated
A_33_P324	0.04057	0.0187	1.72	ZNF365	zinc finger protein 365	Upregulated
A_33_P323	0.000867	0.0000573	1.71	IL6ST	interleukin 6 signal transducer	Upregulated
A_33_P322	0.000905	0.000062	1.71	ATP13A3	ATPase type 13A3	Upregulated
A_21_P000	0.000905	0.0000621	1.71	lnc-IRF2-2	lnc-IRF2-2:3	Upregulated
A_23_P255	0.000925	0.0000651	1.71	GSN	gelsolin	Upregulated

A_23_P119	0.000959	0.0000714	1.71	URI1	URI1, prefoldin-like chaperone	Upregulated
A_23_P850	0.000961	0.000072	1.71	ZRSR2	zinc finger (CCCH type), RNA-binding motif and serine/arginine rich 2	Upregulated
A_23_P373	0.000995	0.0000791	1.71	PUS10	pseudouridylate synthase 10	Upregulated
A_23_P516	0.001054	0.0000894	1.71	MEF2D	myocyte enhancer factor 2D	Upregulated
A_23_P122	0.001203	0.000119	1.71	BRI3	brain protein I3	Upregulated
A_33_P339	0.001224	0.000124	1.71	ELP5	elongator acetyltransferase complex subunit 5	Upregulated
A_23_P233	0.001409	0.000161	1.71	RRP15	ribosomal RNA processing 15 homolog (S. cerevisiae)	Upregulated
A_21_P000	0.001481	0.000177	1.71	lnc-RP1-239B	lnc-RP1-239B22.1.1-1:2	Upregulated
A_23_P167	0.001561	0.000193	1.71	RNF145	ring finger protein 145	Upregulated
A_23_P345	0.001626	0.000208	1.71	GNL2	guanine nucleotide binding protein-like 2 (nucleolar)	Upregulated
A_33_P321	0.001785	0.000245	1.71	CDC6	cell division cycle 6	Upregulated
A_21_P001	0.002498	0.000429	1.71	KCTD21-AS1	KCTD21 antisense RNA 1	Upregulated
A_21_P001	0.002498	0.000429	1.71	XLOC_l2_011048		Upregulated
A_23_P361	0.002855	0.000526	1.71	GTF2H1	general transcription factor IIH, polypeptide 1, 62kDa	Upregulated
A_33_P331	0.003126	0.000606	1.71	TP73-AS1	TP73 antisense RNA 1	Upregulated
A_22_P000	0.006688	0.00185	1.71	lnc-DGKH-1	lnc-DGKH-1:1	Upregulated
A_23_P422	0.007029	0.00198	1.71	C9orf116	chromosome 9 open reading frame 116	Upregulated
A_33_P341	0.012219	0.00415	1.71	CBLN3	cerebellin 3 precursor	Upregulated
A_33_P331	0.016569	0.00616	1.71	HNRNPA0	heterogeneous nuclear ribonucleoprotein A0	Upregulated
A_22_P000	0.020197	0.00786	1.71	lnc-CAMK1G-	lnc-CAMK1G-1:6	Upregulated
A_19_P008	0.033028	0.0145	1.71	KTN1-AS1	KTN1 antisense RNA 1	Upregulated
A_23_P119	0.000912	0.0000635	1.7	EBI3	Epstein-Barr virus induced 3	Upregulated
A_33_P341	0.001062	0.0000912	1.7	NRBF2	nuclear receptor binding factor 2	Upregulated
A_23_P746	0.001161	0.000111	1.7	NUDC	nudC nuclear distribution protein	Upregulated
A_23_P368	0.001196	0.000118	1.7	NETO2	neuropilin (NRP) and tolloid (TLL)-like 2	Upregulated
A_33_P334	0.001297	0.000138	1.7	MAP4	microtubule-associated protein 4	Upregulated
A_23_P111	0.001321	0.000142	1.7	MAK16	MAK16 homolog (S. cerevisiae)	Upregulated
A_23_P132	0.001477	0.000175	1.7	MANF	mesencephalic astrocyte-derived neurotrophic factor	Upregulated
A_23_P768	0.001877	0.000268	1.7	PRMT5	protein arginine methyltransferase 5	Upregulated
A_22_P000	0.002491	0.000427	1.7	lnc-KLHL25-5	lnc-KLHL25-5:1	Upregulated
A_32_P133	0.00437	0.000999	1.7	DCAF10	DDB1 and CUL4 associated factor 10	Upregulated
A_33_P335	0.00437	0.000999	1.7	ZNF273	zinc finger protein 273	Upregulated
A_23_P423	0.004594	0.00107	1.7	RXRA	retinoid X receptor, alpha	Upregulated
A_23_P379	0.005162	0.00127	1.7	FAM63B	family with sequence similarity 63, member B	Upregulated
A_33_P323	0.005196	0.00128	1.7	INPP5F	inositol polyphosphate-5-phosphatase F	Upregulated
A_23_P926	0.005502	0.0014	1.7	DAP	death-associated protein	Upregulated
A_22_P000	0.008307	0.00249	1.7	lnc-WDR67-2	lnc-WDR67-2:1	Upregulated

A_23_P123	0.008661	0.00264	1.7	PDAP1	PDGFA associated protein 1	Upregulated
A_23_P216	0.008954	0.00276	1.7	SLC44A1	solute carrier family 44 (choline transporter), member 1	Upregulated
A_32_P711	0.009133	0.00283	1.7	SCAI	suppressor of cancer cell invasion	Upregulated
A_23_P503	0.01333	0.00464	1.7	FAM32A	family with sequence similarity 32, member A	Upregulated
A_24_P914	0.018847	0.00722	1.7	MKLN1	muskelin 1, intracellular mediator containing kelch motifs	Upregulated
A_32_P102	0.022757	0.00911	1.7	LOC541473	FK506 binding protein 6, 36kDa pseudogene	Upregulated
A_24_P340	0.027809	0.0117	1.7	ZNF621	zinc finger protein 621	Upregulated
A_23_P438	0.042467	0.0197	1.7	KRT24	keratin 24, type I	Upregulated
A_23_P863	0.001199	0.000119	1.69	IER5	immediate early response 5	Upregulated
A_23_P812	0.001216	0.000122	1.69	MRPS18C	mitochondrial ribosomal protein S18C	Upregulated
A_32_P163	0.001279	0.000135	1.69	SGMS1	sphingomyelin synthase 1	Upregulated
A_23_P118	0.001566	0.000195	1.69	GINS2	GINS complex subunit 2 (Psf2 homolog)	Upregulated
A_23_P546	0.00176	0.000239	1.69	KIF22	kinesin family member 22	Upregulated
A_33_P333	0.003543	0.000732	1.69	MSL3	male-specific lethal 3 homolog (Drosophila)	Upregulated
A_23_P213	0.00384	0.000824	1.69	PPARGC1B	peroxisome proliferator-activated receptor gamma, coactivator 1 beta	Upregulated
A_23_P103	0.005933	0.00156	1.69	YY1AP1	YY1 associated protein 1	Upregulated
A_21_P001	0.030178	0.013	1.69	CLUHP3	clustered mitochondria (cluA/CLU1) homolog pseudogene 3	Upregulated
A_32_P507	0.038787	0.0177	1.69	PI4KAP2	phosphatidylinositol 4-kinase, catalytic, alpha pseudogene 2	Upregulated
A_22_P000	0.000905	0.0000621	1.68	lnc-CCDC73-1	lnc-CCDC73-1:1	Upregulated
A_24_P530	0.000947	0.0000694	1.68	SPG7	spastic paraplegia 7 (pure and complicated autosomal recessive)	Upregulated
A_23_P218	0.001126	0.000105	1.68	CRYZL1	crystallin, zeta (quinone reductase)-like 1	Upregulated
A_24_P328	0.001151	0.000109	1.68	TRAPPC11	trafficking protein particle complex 11	Upregulated
A_23_P410	0.00121	0.000121	1.68	GNL3	guanine nucleotide binding protein-like 3 (nucleolar)	Upregulated
A_33_P324	0.001249	0.000129	1.68	DDX19A	DEAD (Asp-Glu-Ala-Asp) box polypeptide 19A	Upregulated
A_23_P252	0.002163	0.000344	1.68	MRPS26	mitochondrial ribosomal protein S26	Upregulated
A_21_P001	0.002221	0.000359	1.68	ZNF663P	zinc finger protein 663, pseudogene	Upregulated
A_23_P312	0.003448	0.000702	1.68	CCDC82	coiled-coil domain containing 82	Upregulated
A_23_P370	0.003701	0.000779	1.68	MCM4	minichromosome maintenance complex component 4	Upregulated
A_23_P211	0.004275	0.000965	1.68	OPA1	optic atrophy 1 (autosomal dominant)	Upregulated
A_23_P167	0.006568	0.0018	1.68	MGAT5	mannosyl (alpha-1,6-)-glycoprotein beta-1,6-N-acetyl-glucosaminyltransf	Upregulated
A_21_P000	0.009091	0.00281	1.68	SNORD107	small nucleolar RNA, C/D box 107	Upregulated
A_23_P503	0.009108	0.00282	1.68	TRIP10	thyroid hormone receptor interactor 10	Upregulated
A_23_P313	0.009711	0.00307	1.68	LRRN3	leucine rich repeat neuronal 3	Upregulated
A_23_P204	0.012732	0.00436	1.68	VEZT	vezatin, adherens junctions transmembrane protein	Upregulated
A_24_P819	0.013059	0.00451	1.68	SLC2A3	solute carrier family 2 (facilitated glucose transporter), member 3	Upregulated
A_33_P378	0.019638	0.0076	1.68	SBNO1	strawberry notch homolog 1 (Drosophila)	Upregulated
A_22_P000	0.023517	0.00951	1.68	lnc-SIAH1-1	lnc-SIAH1-1:1	Upregulated

A_33_P328	0.000908	0.0000626	1.67	CTSA	cathepsin A	Upregulated
A_23_P759	0.000954	0.0000703	1.67	TRAF6	TNF receptor-associated factor 6, E3 ubiquitin protein ligase	Upregulated
A_24_P151	0.000963	0.0000723	1.67	POF1B	premature ovarian failure, 1B	Upregulated
A_23_P914	0.000971	0.0000741	1.67	PSMA7	proteasome (prosome, macropain) subunit, alpha type, 7	Upregulated
A_23_P217	0.000991	0.0000773	1.67	GSTM4	glutathione S-transferase mu 4	Upregulated
A_33_P336	0.001133	0.000106	1.67	MED31	mediator complex subunit 31	Upregulated
A_21_P001	0.001139	0.000107	1.67	GOLGA8K	golgin A8 family, member K	Upregulated
A_33_P326	0.001226	0.000124	1.67	EPG5	ectopic P-granules autophagy protein 5 homolog (C. elegans)	Upregulated
A_23_P334	0.00131	0.00014	1.67	LY75	lymphocyte antigen 75	Upregulated
A_23_P170	0.001528	0.000186	1.67	E2F6	E2F transcription factor 6	Upregulated
A_33_P342	0.00192	0.000279	1.67	YTHDC2	YTH domain containing 2	Upregulated
A_33_P329	0.002442	0.000413	1.67	VPS52	vacuolar protein sorting 52 homolog (S. cerevisiae)	Upregulated
A_24_P889	0.002756	0.000499	1.67	XPC	xeroderma pigmentosum, complementation group C	Upregulated
A_21_P000	0.003094	0.000595	1.67	lnc-DNAJC19-	lnc-DNAJC19-2:2	Upregulated
A_33_P332	0.003456	0.000704	1.67	NTRK2	neurotrophic tyrosine kinase, receptor, type 2	Upregulated
A_21_P000	0.004864	0.00117	1.67	SNORD121A	small nucleolar RNA, C/D box 121A	Upregulated
A_23_P226	0.005292	0.00132	1.67	ARMCX1	armadillo repeat containing, X-linked 1	Upregulated
A_33_P325	0.010051	0.00322	1.67	GSTA4	glutathione S-transferase alpha 4	Upregulated
A_21_P000	0.025056	0.0103	1.67	lnc-SCEL-1	lnc-SCEL-1:1	Upregulated
A_23_P900	0.052165	0.0252	1.67	GCDH	glutaryl-CoA dehydrogenase	Upregulated
A_33_P326	0.001046	0.0000877	1.66	CLNK	cytokine-dependent hematopoietic cell linker	Upregulated
A_23_P930	0.001076	0.0000944	1.66	EXOC3	exocyst complex component 3	Upregulated
A_23_P115	0.001158	0.000111	1.66	EIF2B3	eukaryotic translation initiation factor 2B, subunit 3 gamma, 58kDa	Upregulated
A_23_P141	0.001179	0.000115	1.66	FAM222B	family with sequence similarity 222, member B	Upregulated
A_23_P103	0.001226	0.000124	1.66	EBNA1BP2	EBNA1 binding protein 2	Upregulated
A_24_P381	0.001243	0.000128	1.66	GLRX3	glutaredoxin 3	Upregulated
A_23_P390	0.001266	0.000133	1.66	NUPL1	nucleoporin like 1	Upregulated
A_22_P000	0.001702	0.000227	1.66	lnc-CRIPT-1	lnc-CRIPT-1:1	Upregulated
A_23_P130	0.002187	0.000351	1.66	ENO3	enolase 3 (beta, muscle)	Upregulated
A_24_P138	0.002246	0.000367	1.66	SNX20	sorting nexin 20	Upregulated
A_23_P737	0.00241	0.000406	1.66	MED12	mediator complex subunit 12	Upregulated
A_32_P802	0.003195	0.000629	1.66	DDX6	DEAD (Asp-Glu-Ala-Asp) box helicase 6	Upregulated
A_23_P385	0.004321	0.000981	1.66	STX12	syntaxin 12	Upregulated
A_19_P003	0.005243	0.0013	1.66	LOC1005068	uncharacterized LOC100506860	Upregulated
A_23_P138	0.0077	0.00225	1.66	ACTN3	actinin, alpha 3 (gene/pseudogene)	Upregulated
A_24_P333	0.03335	0.0147	1.66	KLK13	kallikrein-related peptidase 13	Upregulated
A_23_P380	0.033613	0.0148	1.66	ARPC4-TTLL:	ARPC4-TTLL3 readthrough	Upregulated

A_22_P000	0.000939	0.000068	1.65	CFLAR-AS1	CFLAR antisense RNA 1	Upregulated
A_23_P376	0.00114	0.000108	1.65	FAM21C	family with sequence similarity 21, member C	Upregulated
A_32_P175	0.001158	0.000111	1.65	HK2	hexokinase 2	Upregulated
A_23_P192	0.001551	0.000191	1.65	RPF2	ribosome production factor 2 homolog (S. cerevisiae)	Upregulated
A_33_P332	0.001776	0.000243	1.65	INSIG2	insulin induced gene 2	Upregulated
A_33_P342	0.002483	0.000425	1.65	CLEC16A	C-type lectin domain family 16, member A	Upregulated
A_23_P948	0.00293	0.000546	1.65	NUDT9	nudix (nucleoside diphosphate linked moiety X)-type motif 9	Upregulated
A_22_P000	0.003102	0.000599	1.65	RCL1	RNA terminal phosphate cyclase-like 1	Upregulated
A_23_P502	0.003386	0.000683	1.65	SEC22C	SEC22 vesicle trafficking protein homolog C (S. cerevisiae)	Upregulated
A_33_P325	0.004521	0.00105	1.65	EDN1	endothelin 1	Upregulated
A_23_P128	0.014348	0.0051	1.65	ENOX1	ecto-NOX disulfide-thiol exchanger 1	Upregulated
A_24_P405	0.015919	0.00584	1.65	TTC33	tetratricopeptide repeat domain 33	Upregulated
A_24_P413	0.000949	0.0000698	1.64	TP73	tumor protein p73	Upregulated
A_22_P000	0.001066	0.0000923	1.64	lnc-C17orf63-	lnc-C17orf63-1:1	Upregulated
A_21_P001	0.001158	0.000111	1.64	LOC1027246	uncharacterized LOC102724679	Upregulated
A_24_P151	0.001171	0.000113	1.64	KCNAB2	potassium channel, voltage gated subfamily A regulatory beta subunit 2	Upregulated
A_23_P629	0.001203	0.00012	1.64	ATP1B1	ATPase, Na+/K+ transporting, beta 1 polypeptide	Upregulated
A_24_P450	0.001437	0.000166	1.64	NPEPL1	aminopeptidase-like 1	Upregulated
A_33_P329	0.001454	0.000171	1.64	SRRT	serrate, RNA effector molecule	Upregulated
A_23_P481	0.001641	0.000212	1.64	TMEM106C	transmembrane protein 106C	Upregulated
A_33_P341	0.002001	0.0003	1.64	SRSF2	serine/arginine-rich splicing factor 2	Upregulated
A_24_P228	0.002117	0.000332	1.64	MRPL40	mitochondrial ribosomal protein L40	Upregulated
A_24_P294	0.003369	0.000679	1.64	SERTAD2	SERTA domain containing 2	Upregulated
A_21_P000	0.006457	0.00176	1.64	lnc-RNPC3-3	lnc-RNPC3-3:1	Upregulated
A_33_P341	0.011556	0.00386	1.64	GAS2L1	growth arrest-specific 2 like 1	Upregulated
A_23_P323	0.013308	0.00462	1.64	SLC5A12	solute carrier family 5 (sodium/monocarboxylate cotransporter), member	Upregulated
A_33_P337	0.031596	0.0137	1.64	TC2N	tandem C2 domains, nuclear	Upregulated
A_33_P333	0.000995	0.0000786	1.63	RNPC3	RNA-binding region (RNP1, RRM) containing 3	Upregulated
A_23_P818	0.001029	0.0000847	1.63	UBD	ubiquitin D	Upregulated
A_23_P127	0.001193	0.000117	1.63	TNNT3	troponin T type 3 (skeletal, fast)	Upregulated
A_33_P339	0.001553	0.000192	1.63	USE1	unconventional SNARE in the ER 1 homolog (S. cerevisiae)	Upregulated
A_24_P140	0.002199	0.000354	1.63	SORBS2	sorbin and SH3 domain containing 2	Upregulated
A_23_P120	0.002357	0.000392	1.63	TTC3	tetratricopeptide repeat domain 3	Upregulated
A_23_P947	0.002549	0.000442	1.63	TOR1B	torsin family 1, member B (torsin B)	Upregulated
A_23_P904	0.003737	0.000792	1.63	LILRA4	leukocyte immunoglobulin-like receptor, subfamily A (with TM domain), m	Upregulated
A_22_P000	0.003957	0.00086	1.63	lnc-HSPB8-2	lnc-HSPB8-2:1	Upregulated
A_24_P272	0.004251	0.000957	1.63	DENND1A	DENN/MADD domain containing 1A	Upregulated

A_21_P000	0.007789	0.00228	1.63	lnc-EFNB2-2	lnc-EFNB2-2:1	Upregulated
A_22_P000	0.013918	0.0049	1.63	lnc-NLRP12-1	lnc-NLRP12-1:1	Upregulated
A_21_P000	0.016327	0.00605	1.63	lnc-TSN-8	lnc-TSN-8:1	Upregulated
A_22_P000	0.027475	0.0116	1.63	lnc-TBC1D12-	lnc-TBC1D12-1:1	Upregulated
A_23_P953	0.033245	0.0146	1.63	RFC5	replication factor C (activator 1) 5, 36.5kDa	Upregulated
A_33_P342	0.059256	0.0294	1.63	PALLD	palladin, cytoskeletal associated protein	Upregulated
A_23_P100	0.000966	0.0000734	1.62	SUZ12	SUZ12 polycomb repressive complex 2 subunit	Upregulated
A_32_P545	0.000995	0.0000781	1.62	CCT6A	chaperonin containing TCP1, subunit 6A (zeta 1)	Upregulated
A_23_P924	0.001062	0.0000909	1.62	UTP3	UTP3, small subunit (SSU) processome component, homolog (S. cerevis	Upregulated
A_24_P438	0.00112	0.000103	1.62	C9orf40	chromosome 9 open reading frame 40	Upregulated
A_33_P340	0.001163	0.000112	1.62	TMF1	TATA element modulatory factor 1	Upregulated
A_23_P178	0.001697	0.000225	1.62	CD82	CD82 molecule	Upregulated
A_21_P000	0.001954	0.000288	1.62	LINC01005	long intergenic non-protein coding RNA 1005	Upregulated
A_32_P215	0.002124	0.000334	1.62	NPIPA1	nuclear pore complex interacting protein family, member A1	Upregulated
A_32_P144	0.002357	0.000392	1.62	NECAP1	NECAP endocytosis associated 1	Upregulated
A_23_P132	0.00308	0.000591	1.62	USP18	ubiquitin specific peptidase 18	Upregulated
A_32_P225	0.005121	0.00126	1.62	EIF3J-AS1	EIF3J antisense RNA 1 (head to head)	Upregulated
A_33_P341	0.006428	0.00175	1.62	SERPING1	serpin peptidase inhibitor, clade G (C1 inhibitor), member 1	Upregulated
A_24_P177	0.008783	0.00269	1.62	GEN1	GEN1 Holliday junction 5' flap endonuclease	Upregulated
A_23_P200	0.010812	0.00353	1.62	CEP85	centrosomal protein 85kDa	Upregulated
A_23_P843	0.000959	0.0000711	1.61	COPS6	COP9 signalosome subunit 6	Upregulated
A_24_P343	0.000995	0.000079	1.61	ATP5J2	ATP synthase, H+ transporting, mitochondrial Fo complex, subunit F2	Upregulated
A_23_P110	0.001062	0.0000911	1.61	CHIC2	cysteine-rich hydrophobic domain 2	Upregulated
A_24_P137	0.001068	0.000093	1.61	ZNF34	zinc finger protein 34	Upregulated
A_23_P880	0.001068	0.0000932	1.61	TBC1D4	TBC1 domain family, member 4	Upregulated
A_32_P171	0.001075	0.0000942	1.61	NBPF10	neuroblastoma breakpoint family, member 10	Upregulated
A_23_P315	0.001087	0.0000963	1.61	KIAA0930	KIAA0930	Upregulated
A_23_P350	0.001098	0.0000985	1.61	REEP5	receptor accessory protein 5	Upregulated
A_23_P133	0.001121	0.000104	1.61	HARS	histidyl-tRNA synthetase	Upregulated
A_23_P930	0.001124	0.000104	1.61	NUS1	nuclear undecaprenyl pyrophosphate synthase 1 homolog (S. cerevisia	Upregulated
A_21_P001	0.001222	0.000123	1.61	lnc-TAF9-2	lnc-TAF9-2:1	Upregulated
A_33_P322	0.00124	0.000126	1.61	DOK3	docking protein 3	Upregulated
A_23_P103	0.001235	0.000126	1.61	KTI12	KTI12 homolog, chromatin associated (S. cerevisiae)	Upregulated
A_23_P395	0.001356	0.000149	1.61	UBE2F	ubiquitin-conjugating enzyme E2F (putative)	Upregulated
A_33_P334	0.001824	0.000255	1.61	LOC1001313	uncharacterized LOC100131373	Upregulated
A_23_P140	0.002078	0.000322	1.61	CYFIP1	cytoplasmic FMR1 interacting protein 1	Upregulated
A_33_P332	0.002618	0.000461	1.61	FUBP3	far upstream element (FUSE) binding protein 3	Upregulated

A_23_P383	0.003651	0.000764	1.61	NFKBID	nuclear factor of kappa light polypeptide gene enhancer in B-cells inhibi	Upregulated
A_23_P152	0.004508	0.00104	1.61	SLC6A4	solute carrier family 6 (neurotransmitter transporter), member 4	Upregulated
A_24_P201	0.008442	0.00255	1.61	PPP1R3B	protein phosphatase 1, regulatory subunit 3B	Upregulated
A_33_P332	0.01123	0.00371	1.61	lnc-PAX9-2	lnc-PAX9-2:1	Upregulated
A_23_P429	0.021351	0.00842	1.61	ZNF232	zinc finger protein 232	Upregulated
A_21_P001	0.066164	0.0335	1.61	BMS1P20	BMS1 pseudogene 20	Upregulated
A_23_P145	0.000995	0.0000778	1.6	MED23	mediator complex subunit 23	Upregulated
A_23_P458	0.000995	0.0000791	1.6	CHD1L	chromodomain helicase DNA binding protein 1-like	Upregulated
A_21_P001	0.001029	0.000085	1.6	XLOC_l2_010013		Upregulated
A_22_P000	0.001065	0.0000919	1.6	lnc-SRGAP3-	lnc-SRGAP3-1:21	Upregulated
A_22_P000	0.001133	0.000106	1.6	lnc-CTBP2-1	lnc-CTBP2-1:1	Upregulated
A_23_P129	0.001173	0.000113	1.6	GOLGA2P7	golgin A2 pseudogene 7	Upregulated
A_33_P331	0.001215	0.000122	1.6	ZNRF2	zinc and ring finger 2, E3 ubiquitin protein ligase	Upregulated
A_24_P345	0.001949	0.000286	1.6	XRCC5	X-ray repair complementing defective repair in Chinese hamster cells 5 (	Upregulated
A_23_P277	0.001967	0.000292	1.6	FBXO46	F-box protein 46	Upregulated
A_24_P521	0.003176	0.000622	1.6	KLHL24	kelch-like family member 24	Upregulated
A_23_P725	0.004084	0.000898	1.6	KLHL2	kelch-like family member 2	Upregulated
A_33_P330	0.0044	0.00101	1.6	GPX4	glutathione peroxidase 4	Upregulated
A_23_P371	0.004588	0.00107	1.6	DTD2	D-tyrosyl-tRNA deacylase 2 (putative)	Upregulated
A_23_P160	0.006067	0.00161	1.6	PPAN	peter pan homolog (Drosophila)	Upregulated
A_21_P000	0.006553	0.0018	1.6	LINC00882	long intergenic non-protein coding RNA 882	Upregulated
A_23_P693	0.009096	0.00282	1.6	HYAL1	hyaluronoglucosaminidase 1	Upregulated
A_24_P235	0.012945	0.00446	1.6	MTHFD1L	methylenetetrahydrofolate dehydrogenase (NADP+ dependent) 1-like	Upregulated
A_22_P000	0.014197	0.00503	1.6	lnc-TREX1-2	lnc-TREX1-2:1	Upregulated
A_33_P335	0.017622	0.00665	1.6	DUSP8	dual specificity phosphatase 8	Upregulated
A_21_P001	0.000979	0.0000756	1.59	XLOC_l2_015478		Upregulated
A_24_P351	0.000995	0.0000782	1.59	IMMT	inner membrane protein, mitochondrial	Upregulated
A_23_P369	0.001062	0.0000908	1.59	FAM98C	family with sequence similarity 98, member C	Upregulated
A_24_P137	0.001066	0.0000924	1.59	IFRD1	interferon-related developmental regulator 1	Upregulated
A_23_P211	0.001087	0.0000964	1.59	NUP50	nucleoporin 50kDa	Upregulated
A_23_P885	0.001219	0.000123	1.59	ARID3B	AT rich interactive domain 3B (BRIGHT-like)	Upregulated
A_32_P273	0.001341	0.000146	1.59	TCTN3	tectonic family member 3	Upregulated
A_23_P124	0.001393	0.000156	1.59	CHKA	choline kinase alpha	Upregulated
A_33_P323	0.001985	0.000296	1.59	F11R	F11 receptor	Upregulated
A_23_P588	0.002046	0.000314	1.59	GSTM2P1	glutathione S-transferase mu 2 (muscle) pseudogene 1	Upregulated
A_24_P144	0.002232	0.000362	1.59	DHRS4-AS1	DHRS4 antisense RNA 1	Upregulated
A_21_P000	0.002528	0.000437	1.59	lnc-PRKCQ-2	lnc-PRKCQ-2:1	Upregulated

A_33_P335	0.003543	0.000732	1.59	POGZ	pogo transposable element with ZNF domain	Upregulated
A_33_P326	0.005964	0.00157	1.59	MPDU1	mannose-P-dolichol utilization defect 1	Upregulated
A_23_P105	0.009292	0.0029	1.59	TMEM116	transmembrane protein 116	Upregulated
A_21_P000	0.014899	0.00537	1.59	LOC1019293	uncharacterized LOC101929382	Upregulated
A_22_P000	0.017875	0.00676	1.59	lnc-PPM1D-1	lnc-PPM1D-1:1	Upregulated
A_24_P210	0.019849	0.0077	1.59	NME4	NME/NM23 nucleoside diphosphate kinase 4	Upregulated
A_33_P329	0.023681	0.00959	1.59	TCOF1	Treacher Collins-Franceschetti syndrome 1	Upregulated
A_23_P116	0.02952	0.0127	1.59	ATG101	autophagy related 101	Upregulated
A_33_P331	0.040833	0.0188	1.59	AP2A2	adaptor-related protein complex 2, alpha 2 subunit	Upregulated
A_23_P469	0.000995	0.0000779	1.58	HIF1AN	hypoxia inducible factor 1, alpha subunit inhibitor	Upregulated
A_21_P000	0.001004	0.0000805	1.58	LINC01128	long intergenic non-protein coding RNA 1128	Upregulated
A_33_P337	0.001007	0.0000812	1.58	MFAP3	microfibrillar-associated protein 3	Upregulated
A_24_P349	0.001046	0.0000879	1.58	POM121	POM121 transmembrane nucleoporin	Upregulated
A_23_P560	0.001361	0.00015	1.58	DOK1	docking protein 1, 62kDa (downstream of tyrosine kinase 1)	Upregulated
A_33_P322	0.00161	0.000204	1.58	HIST1H2BF	histone cluster 1, H2bf	Upregulated
A_33_P340	0.001854	0.000263	1.58	YWHAE	tyrosine 3-monooxygenase/tryptophan 5-monooxygenase activation prc	Upregulated
A_19_P008	0.001859	0.000264	1.58	APTR	Alu-mediated CDKN1A/p21 transcriptional regulator (non-protein coding	Upregulated
A_22_P000	0.002132	0.000338	1.58	lnc-RNF125-3	lnc-RNF125-3:1	Upregulated
A_33_P324	0.003094	0.000596	1.58	KCNE4	potassium channel, voltage gated subfamily E regulatory beta subunit 4	Upregulated
A_23_P271	0.003535	0.000729	1.58	NSRP1	nuclear speckle splicing regulatory protein 1	Upregulated
A_24_P471	0.004579	0.00107	1.58	VCL	vinculin	Upregulated
A_33_P338	0.005554	0.00142	1.58	ANKRD20A1	ankyrin repeat domain 20 family, member A11, pseudogene	Upregulated
A_23_P129	0.005554	0.00142	1.58	EFCAB11	EF-hand calcium binding domain 11	Upregulated
A_22_P000	0.005554	0.00142	1.58	STARD13	StAR-related lipid transfer (START) domain containing 13	Upregulated
A_33_P324	0.02016	0.00785	1.58	DGKK	diacylglycerol kinase, kappa	Upregulated
A_32_P165	0.025419	0.0105	1.58	IQCF2	IQ motif containing F2	Upregulated
A_33_P322	0.031069	0.0135	1.58	RREB1	ras responsive element binding protein 1	Upregulated
A_33_P330	0.000991	0.0000772	1.57	TAPT1	transmembrane anterior posterior transformation 1	Upregulated
A_23_P143	0.001022	0.0000837	1.57	ARHGEF3	Rho guanine nucleotide exchange factor (GEF) 3	Upregulated
A_23_P104	0.001069	0.0000935	1.57	DNAJC9	DnaJ (Hsp40) homolog, subfamily C, member 9	Upregulated
A_21_P001	0.001254	0.00013	1.57	XLOC_l2_001448		Upregulated
A_23_P141	0.00129	0.000136	1.57	SUPT6H	suppressor of Ty 6 homolog (S. cerevisiae)	Upregulated
A_24_P940	0.001453	0.00017	1.57	STK4	serine/threonine kinase 4	Upregulated
A_22_P000	0.001454	0.000171	1.57	lnc-FAM196B	lnc-FAM196B-1:1	Upregulated
A_23_P839	0.001563	0.000194	1.57	KDM2A	lysine (K)-specific demethylase 2A	Upregulated
A_23_P605	0.001581	0.000198	1.57	PRPF4	pre-mRNA processing factor 4	Upregulated
A_23_P214	0.001803	0.000249	1.57	MTRF1L	mitochondrial translational release factor 1-like	Upregulated

A_33_P324	0.005095	0.00125	1.57	VARS	valyl-tRNA synthetase	Upregulated
A_22_P000	0.00608	0.00161	1.57	lnc-PLOD2-2	lnc-PLOD2-2:1	Upregulated
A_23_P326	0.010867	0.00355	1.57	U2AF1L4	U2 small nuclear RNA auxiliary factor 1-like 4	Upregulated
A_23_P108	0.001199	0.000119	1.56	MREG	melanoregulin	Upregulated
A_24_P105	0.001328	0.000144	1.56	AP1S3	adaptor-related protein complex 1, sigma 3 subunit	Upregulated
A_23_P142	0.001528	0.000186	1.56	LILRA2	leukocyte immunoglobulin-like receptor, subfamily A (with TM domain), m	Upregulated
A_24_P223	0.001857	0.000263	1.56	FNDC3B	fibronectin type III domain containing 3B	Upregulated
A_22_P000	0.001912	0.000276	1.56	lnc-SLC12A7·	lnc-SLC12A7-1:16	Upregulated
A_23_P114	0.002012	0.000304	1.56	MORF4L2	mortality factor 4 like 2	Upregulated
A_24_P811	0.002258	0.00037	1.56	PPFIBP1	PTPRF interacting protein, binding protein 1 (liprin beta 1)	Upregulated
A_33_P332	0.002659	0.000471	1.56	PHACTR4	phosphatase and actin regulator 4	Upregulated
A_23_P407	0.002951	0.000553	1.56	COMMD2	COMM domain containing 2	Upregulated
A_21_P001	0.003094	0.000595	1.56	XLOC_l2_007967		Upregulated
A_23_P372	0.003877	0.000835	1.56	S100A13	S100 calcium binding protein A13	Upregulated
A_23_P876	0.005765	0.00149	1.56	SART3	squamous cell carcinoma antigen recognized by T cells 3	Upregulated
A_22_P000	0.006367	0.00172	1.56	LINC00460	long intergenic non-protein coding RNA 460	Upregulated
A_33_P327	0.015162	0.00549	1.56	CLTC	clathrin, heavy chain (Hc)	Upregulated
A_33_P339	0.016157	0.00596	1.56	P4HA2	prolyl 4-hydroxylase, alpha polypeptide II	Upregulated
A_33_P322	0.021531	0.0085	1.56	VWA8	von Willebrand factor A domain containing 8	Upregulated
A_24_P187	0.028225	0.012	1.56	BID	BH3 interacting domain death agonist	Upregulated
A_23_P332	0.032256	0.0141	1.56	ARHGEF19	Rho guanine nucleotide exchange factor (GEF) 19	Upregulated
A_24_P256	0.032256	0.0141	1.56	LOC1001282	uncharacterized LOC100128288	Upregulated
A_22_P000	0.001108	0.000101	1.55	CECR7	cat eye syndrome chromosome region, candidate 7 (non-protein coding	Upregulated
A_23_P164	0.001171	0.000113	1.55	ZBTB45	zinc finger and BTB domain containing 45	Upregulated
A_24_P190	0.001297	0.000138	1.55	TMEM97	transmembrane protein 97	Upregulated
A_23_P102	0.001331	0.000144	1.55	BCL2L13	BCL2-like 13 (apoptosis facilitator)	Upregulated
A_33_P352	0.001412	0.000161	1.55	GRPEL1	GrpE-like 1, mitochondrial (E. coli)	Upregulated
A_23_P583	0.001438	0.000166	1.55	C4orf32	chromosome 4 open reading frame 32	Upregulated
A_33_P333	0.002078	0.000322	1.55	DEFB109P1	defensin, beta 109, pseudogene 1	Upregulated
A_23_P954	0.002572	0.00045	1.55	NRXN1	neurexin 1	Upregulated
A_23_P539	0.002625	0.000463	1.55	TP53I3	tumor protein p53 inducible protein 3	Upregulated
A_22_P000	0.003684	0.000774	1.55	C21orf91-OT·	C21orf91 overlapping transcript 1	Upregulated
A_33_P341	0.007348	0.0021	1.55	HP1BP3	heterochromatin protein 1, binding protein 3	Upregulated
A_33_P321	0.010348	0.00334	1.55	SMARCAL1	SWI/SNF related, matrix associated, actin dependent regulator of chrom	Upregulated
A_33_P335	0.011193	0.00369	1.55	RNASEH2C	ribonuclease H2, subunit C	Upregulated
A_33_P377	0.011221	0.00371	1.55	GK5	glycerol kinase 5 (putative)	Upregulated
A_33_P326	0.011221	0.00371	1.55	lnc-ELF2-2	lnc-ELF2-2:3	Upregulated

A_33_P339	0.014228	0.00505	1.55	VSIG10L	V-set and immunoglobulin domain containing 10 like	Upregulated
A_22_P000	0.019522	0.00755	1.55	LINC00635	long intergenic non-protein coding RNA 635	Upregulated
A_33_P330	0.030749	0.0133	1.55	PLEKHB1	pleckstrin homology domain containing, family B (evectins) member 1	Upregulated
A_32_P230	0.001049	0.0000883	1.54	GAS5	growth arrest-specific 5 (non-protein coding)	Upregulated
A_32_P149	0.001198	0.000118	1.54	DNAJC18	DnaJ (Hsp40) homolog, subfamily C, member 18	Upregulated
A_23_P244	0.001245	0.000128	1.54	TMEM5	transmembrane protein 5	Upregulated
A_24_P325	0.001429	0.000164	1.54	FAM160B1	family with sequence similarity 160, member B1	Upregulated
A_23_P214	0.001443	0.000167	1.54	MTHFD1L	methylenetetrahydrofolate dehydrogenase (NADP+ dependent) 1-like	Upregulated
A_33_P332	0.001451	0.000169	1.54	RAB3IP	RAB3A interacting protein	Upregulated
A_33_P328	0.001469	0.000174	1.54	MLLT1	myeloid/lymphoid or mixed-lineage leukemia (trithorax homolog, Drosoph	Upregulated
A_33_P364	0.001753	0.000237	1.54	PDCD4-AS1	PDCD4 antisense RNA 1	Upregulated
A_21_P001	0.001809	0.000251	1.54	LOC729815	uncharacterized LOC729815	Upregulated
A_22_P000	0.001812	0.000252	1.54	lnc-WNT11-2	lnc-WNT11-2:1	Upregulated
A_24_P908	0.001881	0.000269	1.54	THUMPD1	THUMP domain containing 1	Upregulated
A_23_P884	0.002163	0.000344	1.54	INTS9	integrator complex subunit 9	Upregulated
A_23_P906	0.002172	0.000347	1.54	MCM6	minichromosome maintenance complex component 6	Upregulated
A_23_P139	0.004971	0.0012	1.54	RSF1	remodeling and spacing factor 1	Upregulated
A_23_P334	0.007108	0.00201	1.54	FAM126B	family with sequence similarity 126, member B	Upregulated
A_23_P200	0.011035	0.00362	1.54	MAGOH	mago-nashi homolog, proliferation-associated (Drosophila)	Upregulated
A_33_P321	0.013879	0.00488	1.54	ANKRD36	ankyrin repeat domain 36	Upregulated
A_32_P154	0.015315	0.00556	1.54	RNF185	ring finger protein 185	Upregulated
A_33_P338	0.024963	0.0103	1.54	TBC1D3B	TBC1 domain family, member 3B	Upregulated
A_32_P103	0.033635	0.0148	1.54	MCM2	minichromosome maintenance complex component 2	Upregulated
A_21_P001	0.040788	0.0188	1.54	GS1-259H13.	transmembrane protein 225-like	Upregulated
A_23_P337	0.059984	0.0298	1.54	TOM1L2	target of myb1-like 2 (chicken)	Upregulated
A_33_P339	0.001092	0.0000974	1.53	ATL2	atlastin GTPase 2	Upregulated
A_24_P113	0.001105	0.0001	1.53	SLC35E2	solute carrier family 35, member E2	Upregulated
A_21_P000	0.001201	0.000119	1.53	SNHG4	small nucleolar RNA host gene 4 (non-protein coding)	Upregulated
A_33_P326	0.001243	0.000128	1.53	CSNK1G1	casein kinase 1, gamma 1	Upregulated
A_23_P208	0.00129	0.000136	1.53	MAP2K2	mitogen-activated protein kinase kinase 2	Upregulated
A_23_P581	0.001415	0.000162	1.53	EXOSC7	exosome component 7	Upregulated
A_23_P123	0.001476	0.000175	1.53	EXOSC3	exosome component 3	Upregulated
A_22_P000	0.00261	0.000459	1.53	lnc-SH3RF2-1	lnc-SH3RF2-1:1	Upregulated
A_33_P324	0.002696	0.000482	1.53	SLC7A11	solute carrier family 7 (anionic amino acid transporter light chain, xc- syst	Upregulated
A_33_P353	0.002967	0.000558	1.53	C1QTNF9B-A	C1QTNF9B antisense RNA 1	Upregulated
A_24_P350	0.00343	0.000697	1.53	TNIK	TRAF2 and NCK interacting kinase	Upregulated
A_32_P163	0.003648	0.000763	1.53	VDAC1	voltage-dependent anion channel 1	Upregulated

A_33_P333	0.004156	0.000923	1.53	METTL10	methyltransferase like 10	Upregulated
A_24_P982	0.005207	0.00129	1.53	VPS13B	vacuolar protein sorting 13 homolog B (yeast)	Upregulated
A_33_P322	0.005457	0.00138	1.53	MIER2	mesoderm induction early response 1, family member 2	Upregulated
A_21_P000	0.005502	0.0014	1.53	lnc-ZNF486-1	lnc-ZNF486-1:1	Upregulated
A_33_P336	0.007778	0.00228	1.53	TMEM194B	transmembrane protein 194B	Upregulated
A_22_P000	0.007958	0.00235	1.53	lnc-IRX2-5	lnc-IRX2-5:1	Upregulated
A_33_P334	0.015738	0.00576	1.53	RC3H2	ring finger and CCCH-type domains 2	Upregulated
A_23_P410	0.023814	0.00967	1.53	TEX264	testis expressed 264	Upregulated
A_23_P168	0.02435	0.00995	1.53	FBXO16	F-box protein 16	Upregulated
A_32_P790	0.029856	0.0128	1.53	KATNAL2	katanin p60 subunit A-like 2	Upregulated
A_23_P211	0.047957	0.0228	1.53	TGFBR2	transforming growth factor, beta receptor II (70/80kDa)	Upregulated
A_23_P162	0.059037	0.0293	1.53	SERPINE3	serpin peptidase inhibitor, clade E (nexin, plasminogen activator inhibito	Upregulated
A_23_P133	0.001062	0.0000909	1.52	TCERG1	transcription elongation regulator 1	Upregulated
A_33_P323	0.00114	0.000107	1.52	HIF1A	hypoxia inducible factor 1, alpha subunit (basic helix-loop-helix transcrip	Upregulated
A_24_P305	0.00115	0.000109	1.52	LEMD2	LEM domain containing 2	Upregulated
A_22_P000	0.001154	0.00011	1.52	lnc-RP11-392	lnc-RP11-392O18.1.1-2:1	Upregulated
A_33_P341	0.001197	0.000118	1.52	RLIM	ring finger protein, LIM domain interacting	Upregulated
A_23_P256	0.001253	0.00013	1.52	TRAPPC12	trafficking protein particle complex 12	Upregulated
A_23_P222	0.001394	0.000157	1.52	EIF4EBP1	eukaryotic translation initiation factor 4E binding protein 1	Upregulated
A_23_P329	0.001409	0.000161	1.52	ILF3	interleukin enhancer binding factor 3, 90kDa	Upregulated
A_22_P000	0.001498	0.00018	1.52	lnc-GRID2IP-1	lnc-GRID2IP-1:1	Upregulated
A_23_P314	0.001668	0.000217	1.52	LEO1	Leo1, Paf1/RNA polymerase II complex component, homolog (S. cerevis	Upregulated
A_33_P329	0.001751	0.000236	1.52	SMC1A	structural maintenance of chromosomes 1A	Upregulated
A_22_P000	0.001954	0.000288	1.52	TUG1	taurine up-regulated 1 (non-protein coding)	Upregulated
A_33_P331	0.003287	0.000655	1.52	NUS1	nuclear undecaprenyl pyrophosphate synthase 1 homolog (S. cerevisia	Upregulated
A_23_P416	0.004301	0.000973	1.52	TAS2R31	taste receptor, type 2, member 31	Upregulated
A_23_P145	0.00479	0.00114	1.52	ZNF318	zinc finger protein 318	Upregulated
A_23_P253	0.006568	0.0018	1.52	FAM175A	family with sequence similarity 175, member A	Upregulated
A_33_P339	0.00733	0.0021	1.52	ZNF441	zinc finger protein 441	Upregulated
A_24_P303	0.007614	0.00221	1.52	CXCL10	chemokine (C-X-C motif) ligand 10	Upregulated
A_22_P000	0.013997	0.00493	1.52	LOC1019277	uncharacterized LOC101927793	Upregulated
A_19_P003	0.015108	0.00547	1.52	LOC1005059	uncharacterized LOC100505920	Upregulated
A_33_P338	0.015108	0.00547	1.52	PLB1	phospholipase B1	Upregulated
A_22_P000	0.029471	0.0126	1.52	lnc-TYMS-2	lnc-TYMS-2:1	Upregulated
A_33_P335	0.031902	0.0139	1.52	SUV420H2	suppressor of variegation 4-20 homolog 2 (Drosophila)	Upregulated
A_22_P000	0.039388	0.018	1.52	lnc-ZNF790-1	lnc-ZNF790-1:1	Upregulated
A_22_P000	0.040403	0.0186	1.52	LOC1019291	uncharacterized LOC101929172	Upregulated

A_21_P000	0.00104	0.0000865	1.51	lnc-ZFP36L1-	lnc-ZFP36L1-1:1	Upregulated
A_23_P215	0.001047	0.0000881	1.51	ATP5J2	ATP synthase, H+ transporting, mitochondrial Fo complex, subunit F2	Upregulated
A_23_P339	0.001052	0.0000889	1.51	HAT1	histone acetyltransferase 1	Upregulated
A_32_P950	0.001055	0.0000897	1.51	AK4	adenylate kinase 4	Upregulated
A_24_P161	0.001105	0.0001	1.51	ZFAS1	ZNFX1 antisense RNA 1	Upregulated
A_33_P334	0.001107	0.000101	1.51	OPA1	optic atrophy 1 (autosomal dominant)	Upregulated
A_23_P341	0.001134	0.000106	1.51	KLHL21	kelch-like family member 21	Upregulated
A_24_P928	0.001203	0.00012	1.51	LMAN2	lectin, mannose-binding 2	Upregulated
A_23_P976	0.00124	0.000127	1.51	RRAGC	Ras-related GTP binding C	Upregulated
A_23_P735	0.001458	0.000172	1.51	ARAF	A-Raf proto-oncogene, serine/threonine kinase	Upregulated
A_23_P468	0.002167	0.000346	1.51	BTRC	beta-transducin repeat containing E3 ubiquitin protein ligase	Upregulated
A_23_P742	0.003316	0.000665	1.51	SRM	spermidine synthase	Upregulated
A_33_P334	0.003631	0.000758	1.51	ZBTB38	zinc finger and BTB domain containing 38	Upregulated
A_23_P782	0.003706	0.000781	1.51	FAM26F	family with sequence similarity 26, member F	Upregulated
A_33_P330	0.00419	0.000935	1.51	KIAA1841	KIAA1841	Upregulated
A_22_P000	0.004196	0.000937	1.51	lnc-TMED5-1	lnc-TMED5-1:27	Upregulated
A_32_P730	0.004748	0.00113	1.51	CMAS	cytidine monophosphate N-acetylneuraminic acid synthetase	Upregulated
A_32_P589	0.00479	0.00114	1.51	MBTD1	mbt domain containing 1	Upregulated
A_33_P341	0.004841	0.00116	1.51	C7orf60	chromosome 7 open reading frame 60	Upregulated
A_21_P000	0.010047	0.00321	1.51	lnc-SOX6-1	lnc-SOX6-1:1	Upregulated
A_33_P342	0.011268	0.00373	1.51	RSU1	Ras suppressor protein 1	Upregulated
A_23_P941	0.036858	0.0166	1.51	RAD54B	RAD54 homolog B (S. cerevisiae)	Upregulated
A_21_P001	0.041579	0.0192	1.51	XLOC_l2_010348		Upregulated
A_22_P000	0.051415	0.0248	1.51	lnc-CNOT7-1	lnc-CNOT7-1:1	Upregulated
A_22_P000	0.001115	0.000102	1.5	LOC1019271	uncharacterized LOC101927104	Upregulated
A_33_P321	0.001171	0.000113	1.5	RCC2	regulator of chromosome condensation 2	Upregulated
A_23_P201	0.001336	0.000145	1.5	CREM	cAMP responsive element modulator	Upregulated
A_21_P000	0.00142	0.000163	1.5	USP12-AS2	USP12 antisense RNA 2 (head to head)	Upregulated
A_22_P000	0.001476	0.000175	1.5	FAM155A-IT1	FAM155A intronic transcript 1 (non-protein coding)	Upregulated
A_19_P008	0.00168	0.00022	1.5	lnc-OSTC-2	lnc-OSTC-2:1	Upregulated
A_33_P366	0.00168	0.00022	1.5	LOC284669	uncharacterized LOC284669	Upregulated
A_24_P277	0.002096	0.000325	1.5	CNPY3	canopy FGF signaling regulator 3	Upregulated
A_23_P251	0.0021	0.000326	1.5	NSUN5	NOP2/Sun domain family, member 5	Upregulated
A_22_P000	0.00293	0.000546	1.5	lnc-MTHFR-2	lnc-MTHFR-2:1	Upregulated
A_22_P000	0.002941	0.000549	1.5	lnc-INPPL1-1	lnc-INPPL1-1:1	Upregulated
A_24_P231	0.003889	0.000839	1.5	BOD1L1	biorientation of chromosomes in cell division 1-like 1	Upregulated
A_24_P261	0.006914	0.00193	1.5	VPRBP	Vpr (HIV-1) binding protein	Upregulated

A_23_P324	0.01058	0.00343	1.5	DISP2	dispatched homolog 2 (Drosophila)	Upregulated
A_24_P385	0.019404	0.0075	1.5	NOL6	nucleolar protein 6 (RNA-associated)	Upregulated
A_23_P430	0.023487	0.00949	1.5	INTS8	integrator complex subunit 8	Upregulated
A_21_P000	0.023986	0.00976	1.5	lnc-RASA1-3	lnc-RASA1-3:1	Upregulated
A_23_P140	0.031297	0.0136	1.5	MYO5C	myosin VC	Upregulated
A_32_P212	0.031758	0.0138	1.5	CEP19	centrosomal protein 19kDa	Upregulated
A_21_P000	0.0352	0.0157	1.5	NKAPP1	NFKB activating protein pseudogene 1	Upregulated
A_33_P326	0.044346	0.0208	1.5	TDRD10	tudor domain containing 10	Upregulated
A_23_P215	0.00126	0.000131	1.49	AHR	aryl hydrocarbon receptor	Upregulated
A_23_P102	0.001333	0.000145	1.49	SSFA2	sperm specific antigen 2	Upregulated
A_23_P168	0.001443	0.000168	1.49	HERPUD2	HERPUD family member 2	Upregulated
A_33_P329	0.001478	0.000176	1.49	DCAF16	DDB1 and CUL4 associated factor 16	Upregulated
A_32_P749	0.001548	0.00019	1.49	EIF1AD	eukaryotic translation initiation factor 1A domain containing	Upregulated
A_21_P001	0.0017	0.000226	1.49	XLOC_l2_001967		Upregulated
A_33_P331	0.002258	0.00037	1.49	RBMS1	RNA binding motif, single stranded interacting protein 1	Upregulated
A_32_P687	0.00376	0.000798	1.49	SMU1	smu-1 suppressor of mec-8 and unc-52 homolog (C. elegans)	Upregulated
A_19_P008	0.004094	0.000902	1.49	lnc-HIST1H1A	lnc-HIST1H1A-1:2	Upregulated
A_22_P000	0.0056	0.00143	1.49	LOC1019289	uncharacterized LOC101928989	Upregulated
A_22_P000	0.007045	0.00199	1.49	lnc-C9orf163-	lnc-C9orf163-1:1	Upregulated
A_23_P207	0.009601	0.00302	1.49	RNFT1	ring finger protein, transmembrane 1	Upregulated
A_21_P000	0.012808	0.0044	1.49	lnc-CHL1-1	lnc-CHL1-1:3	Upregulated
A_22_P000	0.048098	0.0229	1.49	LOC1001313	uncharacterized LOC100131315	Upregulated
A_23_P551	0.001076	0.0000945	1.48	EFTUD2	elongation factor Tu GTP binding domain containing 2	Upregulated
A_23_P562	0.001243	0.000128	1.48	GMIP	GEM interacting protein	Upregulated
A_23_P113	0.00142	0.000163	1.48	ATP6AP2	ATPase, H+ transporting, lysosomal accessory protein 2	Upregulated
A_23_P311	0.001821	0.000255	1.48	MITD1	MIT, microtubule interacting and transport, domain containing 1	Upregulated
A_23_P828	0.001827	0.000257	1.48	PINX1	PIN2/TERF1 interacting, telomerase inhibitor 1	Upregulated
A_32_P233	0.002078	0.000322	1.48	FAM133B	family with sequence similarity 133, member B	Upregulated
A_33_P336	0.003155	0.000615	1.48	PAM	peptidylglycine alpha-amidating monooxygenase	Upregulated
A_23_P215	0.003751	0.000796	1.48	TYW1	tRNA-yW synthesizing protein 1 homolog (S. cerevisiae)	Upregulated
A_23_P376	0.003909	0.000845	1.48	CLYBL	citrate lyase beta like	Upregulated
A_33_P322	0.006617	0.00182	1.48	HERC2P10	hect domain and RLD 2 pseudogene 10	Upregulated
A_23_P110	0.006606	0.00182	1.48	HERC5	HECT and RLD domain containing E3 ubiquitin protein ligase 5	Upregulated
A_33_P337	0.011063	0.00364	1.48	TNPO3	transportin 3	Upregulated
A_23_P677	0.016609	0.00618	1.48	SPAG16	sperm associated antigen 16	Upregulated
A_23_P303	0.025744	0.0107	1.48	CRYBG3	beta-gamma crystallin domain containing 3	Upregulated
A_33_P340	0.001376	0.000153	1.47	PPM1B	protein phosphatase, Mg2+/Mn2+ dependent, 1B	Upregulated

A_23_P205	0.001795	0.000247	1.47	MAP4K5	mitogen-activated protein kinase kinase kinase kinase 5	Upregulated
A_23_P146	0.002696	0.000482	1.47	BAG1	BCL2-associated athanogene	Upregulated
A_19_P008	0.002984	0.000563	1.47	lnc-GXYLT1-2	lnc-GXYLT1-2:1	Upregulated
A_32_P234	0.004162	0.000926	1.47	ARMC1	armadillo repeat containing 1	Upregulated
A_22_P000	0.00605	0.0016	1.47	LOC1019275	uncharacterized LOC101927518	Upregulated
A_23_P205	0.007407	0.00213	1.47	NR2E3	nuclear receptor subfamily 2, group E, member 3	Upregulated
A_32_P201	0.008875	0.00272	1.47	HECTD2	HECT domain containing E3 ubiquitin protein ligase 2	Upregulated
A_33_P321	0.009576	0.00301	1.47	TYW1	tRNA-yW synthesizing protein 1 homolog (S. cerevisiae)	Upregulated
A_24_P127	0.011428	0.0038	1.47	CLASP2	cytoplasmic linker associated protein 2	Upregulated
A_22_P000	0.012965	0.00447	1.47	lnc-PHKB-5	lnc-PHKB-5:1	Upregulated
A_22_P000	0.016831	0.00629	1.47	LINC00443	long intergenic non-protein coding RNA 443	Upregulated
A_33_P369	0.01825	0.00695	1.47	LHFPL3-AS2	LHFPL3 antisense RNA 2	Upregulated
A_23_P977	0.036455	0.0164	1.47	TXNIP	thioredoxin interacting protein	Upregulated
A_22_P000	0.039243	0.0179	1.47	lnc-YWHAZ-1	lnc-YWHAZ-1:1	Upregulated
A_33_P325	0.001108	0.000101	1.46	C16orf91	chromosome 16 open reading frame 91	Upregulated
A_23_P405	0.001215	0.000122	1.46	CDC14A	cell division cycle 14A	Upregulated
A_23_P686	0.001354	0.000149	1.46	ADRM1	adhesion regulating molecule 1	Upregulated
A_33_P338	0.001394	0.000156	1.46	AKT2	v-akt murine thymoma viral oncogene homolog 2	Upregulated
A_23_P147	0.001721	0.000231	1.46	ADPGK	ADP-dependent glucokinase	Upregulated
A_32_P788	0.001763	0.00024	1.46	PSPH	phosphoserine phosphatase	Upregulated
A_23_P164	0.00188	0.000269	1.46	MFAP4	microfibrillar-associated protein 4	Upregulated
A_33_P339	0.002067	0.000318	1.46	ZNF420	zinc finger protein 420	Upregulated
A_22_P000	0.002351	0.000391	1.46	lnc-NMNAT1-3	lnc-NMNAT1-3:1	Upregulated
A_21_P001	0.002361	0.000394	1.46	XLOC_l2_012870		Upregulated
A_33_P337	0.002667	0.000474	1.46	MITD1	MIT, microtubule interacting and transport, domain containing 1	Upregulated
A_24_P294	0.003323	0.000667	1.46	TMEM14B	transmembrane protein 14B	Upregulated
A_21_P000	0.003878	0.000836	1.46	SNORD49A	small nucleolar RNA, C/D box 49A	Upregulated
A_21_P001	0.005496	0.00139	1.46	THEGL	theg spermatid protein-like	Upregulated
A_33_P332	0.006945	0.00195	1.46	ZNF615	zinc finger protein 615	Upregulated
A_23_P203	0.007867	0.00231	1.46	SCARB1	scavenger receptor class B, member 1	Upregulated
A_22_P000	0.009276	0.00289	1.46	lnc-TNN-1	lnc-TNN-1:1	Upregulated
A_23_P412	0.016749	0.00625	1.46	MFSD10	major facilitator superfamily domain containing 10	Upregulated
A_23_P582	0.001117	0.000103	1.45	GAR1	GAR1 ribonucleoprotein	Upregulated
A_22_P000	0.001135	0.000107	1.45	lnc-KLLN-1	lnc-KLLN-1:1	Upregulated
A_33_P337	0.001135	0.000107	1.45	MLLT6	myeloid/lymphoid or mixed-lineage leukemia (trithorax homolog, Drosoph	Upregulated
A_33_P321	0.001187	0.000116	1.45	INSIG1	insulin induced gene 1	Upregulated
A_33_P335	0.00125	0.000129	1.45	NUSAP1	nucleolar and spindle associated protein 1	Upregulated

A_23_P391	0.001365	0.000151	1.45	IVNS1ABP	influenza virus NS1A binding protein	Upregulated
A_23_P255	0.001386	0.000155	1.45	DAPP1	dual adaptor of phosphotyrosine and 3-phosphoinositides	Upregulated
A_33_P338	0.001396	0.000157	1.45	SNX30	sorting nexin family member 30	Upregulated
A_33_P335	0.001415	0.000162	1.45	SLAMF1	signaling lymphocytic activation molecule family member 1	Upregulated
A_22_P000	0.001522	0.000185	1.45	BTG1	B-cell translocation gene 1, anti-proliferative	Upregulated
A_33_P325	0.001594	0.000199	1.45	SLC36A1	solute carrier family 36 (proton/amino acid symporter), member 1	Upregulated
A_32_P191	0.00165	0.000213	1.45	SETD8	SET domain containing (lysine methyltransferase) 8	Upregulated
A_23_P132	0.001848	0.000261	1.45	TPST2	tyrosylprotein sulfotransferase 2	Upregulated
A_22_P000	0.003109	0.000602	1.45	lnc-FAM129B	lnc-FAM129B-1:1	Upregulated
A_33_P339	0.007045	0.00199	1.45	BRWD1	bromodomain and WD repeat domain containing 1	Upregulated
A_24_P383	0.007257	0.00207	1.45	KRTAP13-1	keratin associated protein 13-1	Upregulated
A_33_P328	0.017536	0.00661	1.45	ZNF778	zinc finger protein 778	Upregulated
A_23_P866	0.001173	0.000113	1.44	SRGN	serglycin	Upregulated
A_33_P331	0.001187	0.000116	1.44	BTNL9	butyrophilin-like 9	Upregulated
A_23_P142	0.001187	0.000116	1.44	GRWD1	glutamate-rich WD repeat containing 1	Upregulated
A_33_P327	0.001263	0.000132	1.44	ZNF701	zinc finger protein 701	Upregulated
A_22_P000	0.001341	0.000146	1.44	lnc-OIT3-2	lnc-OIT3-2:1	Upregulated
A_33_P325	0.001616	0.000205	1.44	SEC22C	SEC22 vesicle trafficking protein homolog C (S. cerevisiae)	Upregulated
A_23_P167	0.001697	0.000225	1.44	RNF4	ring finger protein 4	Upregulated
A_33_P321	0.001784	0.000245	1.44	FAM3A	family with sequence similarity 3, member A	Upregulated
A_23_P408	0.001785	0.000245	1.44	MXD1	MAX dimerization protein 1	Upregulated
A_22_P000	0.001861	0.000264	1.44	TAF10	TAF10 RNA polymerase II, TATA box binding protein (TBP)-associated f	Upregulated
A_23_P132	0.001959	0.00029	1.44	PES1	pescadillo ribosomal biogenesis factor 1	Upregulated
A_23_P857	0.002021	0.000306	1.44	GNPAT	glyceronephosphate O-acyltransferase	Upregulated
A_23_P134	0.002579	0.000452	1.44	CDYL	chromodomain protein, Y-like	Upregulated
A_23_P138	0.002687	0.000479	1.44	NOC2L	nucleolar complex associated 2 homolog (S. cerevisiae)	Upregulated
A_24_P101	0.002794	0.000508	1.44	NOP56	NOP56 ribonucleoprotein	Upregulated
A_23_P266	0.002832	0.000519	1.44	NMRAL1	NmrA-like family domain containing 1	Upregulated
A_23_P302	0.003036	0.000579	1.44	IPO11	importin 11	Upregulated
A_23_P521	0.003595	0.000748	1.44	ACBD6	acyl-CoA binding domain containing 6	Upregulated
A_23_P208	0.004582	0.00107	1.44	SEMA6B	sema domain, transmembrane domain (TM), and cytoplasmic domain, (s	Upregulated
A_23_P316	0.00479	0.00114	1.44	ZBED6CL	ZBED6 C-terminal like	Upregulated
A_23_P543	0.00812	0.00241	1.44	MEGF11	multiple EGF-like-domains 11	Upregulated
A_33_P331	0.014594	0.00522	1.44	ZDHHC8	zinc finger, DHHC-type containing 8	Upregulated
A_33_P324	0.019729	0.00764	1.44	EMR4P	egf-like module containing, mucin-like, hormone receptor-like 4 pseudog	Upregulated
A_21_P001	0.029345	0.0125	1.44	LOC1019283	uncharacterized LOC101928381	Upregulated
A_33_P330	0.037574	0.017	1.44	PDPR	pyruvate dehydrogenase phosphatase regulatory subunit	Upregulated

A_19_P008	0.037658	0.0171	1.44	NASP	nuclear autoantigenic sperm protein (histone-binding)	Upregulated
A_21_P000	0.049868	0.0239	1.44	lnc-FAM53A-2	lnc-FAM53A-2:1	Upregulated
A_23_P387	0.061638	0.0308	1.44	CDH2	cadherin 2, type 1, N-cadherin (neuronal)	Upregulated
A_32_P108	0.001173	0.000114	1.43	MIR155HG	MIR155 host gene (non-protein coding)	Upregulated
A_23_P215	0.001226	0.000124	1.43	POLR2J3	polymerase (RNA) II (DNA directed) polypeptide J3	Upregulated
A_23_P494	0.001322	0.000142	1.43	ST6GALNAC	ST6 (alpha-N-acetyl-neuraminyl-2,3-beta-galactosyl-1,3)-N-acetylgalacto	Upregulated
A_23_P205	0.001344	0.000147	1.43	GABPB1	GA binding protein transcription factor, beta subunit 1	Upregulated
A_23_P851	0.001359	0.00015	1.43	ARMCX5	armadillo repeat containing, X-linked 5	Upregulated
A_23_P206	0.001398	0.000158	1.43	DNAJA4	DnaJ (Hsp40) homolog, subfamily A, member 4	Upregulated
A_24_P723	0.001404	0.00016	1.43	RBAK	RB-associated KRAB zinc finger	Upregulated
A_33_P324	0.001458	0.000171	1.43	PANK3	pantothenate kinase 3	Upregulated
A_21_P000	0.001499	0.00018	1.43	SNORA5B	small nucleolar RNA, H/ACA box 5B	Upregulated
A_23_P140	0.001522	0.000185	1.43	FBXL3	F-box and leucine-rich repeat protein 3	Upregulated
A_32_P685	0.002113	0.000331	1.43	ZDBF2	zinc finger, DBF-type containing 2	Upregulated
A_33_P333	0.002184	0.00035	1.43	LOC1019278	uncharacterized LOC101927838	Upregulated
A_23_P709	0.003175	0.000622	1.43	UMAD1	UBAP1-MVB12-associated (UMA) domain containing 1	Upregulated
A_33_P339	0.003461	0.000706	1.43	C2orf50	chromosome 2 open reading frame 50	Upregulated
A_21_P001	0.005389	0.00136	1.43	lnc-ABHD12B	lnc-ABHD12B-5:1	Upregulated
A_33_P333	0.008161	0.00243	1.43	PDE1C	phosphodiesterase 1C, calmodulin-dependent 70kDa	Upregulated
A_33_P326	0.008344	0.00251	1.43	RHEBL1	Ras homolog enriched in brain like 1	Upregulated
A_33_P331	0.012521	0.00427	1.43	FBXL17	F-box and leucine-rich repeat protein 17	Upregulated
A_23_P214	0.015734	0.00575	1.43	CNR1	cannabinoid receptor 1 (brain)	Upregulated
A_23_P494	0.015734	0.00575	1.43	LOC81691	exonuclease NEF-sp	Upregulated
A_22_P000	0.066456	0.0337	1.43	lnc-COX17-1	lnc-COX17-1:1	Upregulated
A_21_P000	0.001157	0.00011	1.42	lnc-CADM2-5	lnc-CADM2-5:1	Upregulated
A_23_P412	0.001157	0.00011	1.42	TGM4	transglutaminase 4	Upregulated
A_24_P332	0.001211	0.000121	1.42	RPL7L1	ribosomal protein L7-like 1	Upregulated
A_33_P335	0.001227	0.000124	1.42	PPAPDC1B	phosphatidic acid phosphatase type 2 domain containing 1B	Upregulated
A_23_P256	0.001263	0.000132	1.42	GOLGA4	golgin A4	Upregulated
A_33_P321	0.001479	0.000176	1.42	ZNF777	zinc finger protein 777	Upregulated
A_24_P282	0.001541	0.000189	1.42	SSR2	signal sequence receptor, beta (translocon-associated protein beta)	Upregulated
A_23_P202	0.001604	0.000202	1.42	MAP2K1	mitogen-activated protein kinase kinase 1	Upregulated
A_23_P320	0.001626	0.000208	1.42	BUB3	BUB3 mitotic checkpoint protein	Upregulated
A_33_P328	0.00188	0.000269	1.42	SMCO1	single-pass membrane protein with coiled-coil domains 1	Upregulated
A_24_P185	0.002179	0.000349	1.42	DMD	dystrophin	Upregulated
A_33_P335	0.002535	0.000439	1.42	HSPB1	heat shock 27kDa protein 1	Upregulated
A_23_P141	0.00264	0.000467	1.42	C17orf49	chromosome 17 open reading frame 49	Upregulated

A_23_P423	0.003001	0.000568	1.42	CREB3	cAMP responsive element binding protein 3	Upregulated
A_33_P336	0.003474	0.000709	1.42	AGPAT2	1-acylglycerol-3-phosphate O-acyltransferase 2	Upregulated
A_22_P000	0.004746	0.00113	1.42	OXCT1-AS1	OXCT1 antisense RNA 1	Upregulated
A_23_P218	0.005135	0.00126	1.42	POLQ	polymerase (DNA directed), theta	Upregulated
A_33_P329	0.006015	0.00159	1.42	ZNF550	zinc finger protein 550	Upregulated
A_23_P126	0.006861	0.00191	1.42	TNFSF4	tumor necrosis factor (ligand) superfamily, member 4	Upregulated
A_23_P891	0.008774	0.00268	1.42	EXOC7	exocyst complex component 7	Upregulated
A_23_P357	0.008875	0.00272	1.42	RPS6KA4	ribosomal protein S6 kinase, 90kDa, polypeptide 4	Upregulated
A_24_P701	0.009801	0.00311	1.42	BVES-AS1	BVES antisense RNA 1	Upregulated
A_33_P322	0.015865	0.00581	1.42	PRH2	proline-rich protein HaeIII subfamily 2	Upregulated
A_21_P001	0.016021	0.00589	1.42	LINC00539	long intergenic non-protein coding RNA 539	Upregulated
A_24_P332	0.022982	0.00923	1.42	JAKMIP3	Janus kinase and microtubule interacting protein 3	Upregulated
A_21_P000	0.025809	0.0107	1.42	LOC1019287	uncharacterized LOC101928739	Upregulated
A_33_P324	0.027568	0.0116	1.42	PIBF1	progesterone immunomodulatory binding factor 1	Upregulated
A_21_P001	0.034251	0.0152	1.42	XLOC_l2_004843		Upregulated
A_19_P003	0.034839	0.0155	1.42	LINC00963	long intergenic non-protein coding RNA 963	Upregulated
A_33_P324	0.042922	0.02	1.42	NRK	Nik related kinase	Upregulated
A_33_P337	0.050416	0.0242	1.42	TDRD1	tudor domain containing 1	Upregulated
A_33_P344	0.00118	0.000115	1.41	PPP6R3	protein phosphatase 6, regulatory subunit 3	Upregulated
A_23_P117	0.001203	0.00012	1.41	PRDM4	PR domain containing 4	Upregulated
A_23_P209	0.00121	0.000121	1.41	CD22	CD22 molecule	Upregulated
A_21_P000	0.001242	0.000127	1.41	SNORA5A	small nucleolar RNA, H/ACA box 5A	Upregulated
A_22_P000	0.00129	0.000136	1.41	lnc-SLC25A3	lnc-SLC25A37-1:1	Upregulated
A_33_P326	0.001342	0.000147	1.41	PGAM1	phosphoglycerate mutase 1 (brain)	Upregulated
A_23_P121	0.001398	0.000158	1.41	GBE1	glucan (1,4-alpha-), branching enzyme 1	Upregulated
A_19_P008	0.001448	0.000169	1.41	PPA1	pyrophosphatase (inorganic) 1	Upregulated
A_33_P334	0.0015	0.000181	1.41	EDRF1	erythroid differentiation regulatory factor 1	Upregulated
A_23_P105	0.001693	0.000223	1.41	CHPT1	choline phosphotransferase 1	Upregulated
A_32_P179	0.001779	0.000244	1.41	TOB2	transducer of ERBB2, 2	Upregulated
A_33_P329	0.002005	0.000301	1.41	DEGS1	delta(4)-desaturase, sphingolipid 1	Upregulated
A_33_P356	0.002112	0.00033	1.41	MAD1L1	MAD1 mitotic arrest deficient-like 1 (yeast)	Upregulated
A_23_P485	0.002113	0.000331	1.41	C14orf93	chromosome 14 open reading frame 93	Upregulated
A_33_P328	0.002243	0.000366	1.41	POLL	polymerase (DNA directed), lambda	Upregulated
A_33_P321	0.002559	0.000445	1.41	KLK3	kallikrein-related peptidase 3	Upregulated
A_33_P329	0.005753	0.00149	1.41	DDRGK1	DDRGK domain containing 1	Upregulated
A_22_P000	0.014369	0.00511	1.41	lnc-AVEN-1	lnc-AVEN-1:1	Upregulated
A_33_P333	0.01486	0.00535	1.41	BTN2A1	butyrophilin, subfamily 2, member A1	Upregulated

A_23_P205	0.021173	0.00833	1.41	DDHD1	DDHD domain containing 1	Upregulated
A_23_P122	0.023982	0.00975	1.41	C5orf30	chromosome 5 open reading frame 30	Upregulated
A_32_P159	0.033974	0.015	1.41	ASIC2	acid sensing (proton gated) ion channel 2	Upregulated
A_24_P400	0.001208	0.00012	1.4	GET4	golgi to ER traffic protein 4 homolog (S. cerevisiae)	Upregulated
A_24_P365	0.001211	0.000121	1.4	TSPAN33	tetraspanin 33	Upregulated
A_23_P768	0.00124	0.000126	1.4	NGDN	neuroguidin, EIF4E binding protein	Upregulated
A_33_P340	0.001591	0.000199	1.4	GM2A	GM2 ganglioside activator	Upregulated
A_33_P335	0.001635	0.00021	1.4	USP37	ubiquitin specific peptidase 37	Upregulated
A_24_P285	0.001772	0.000242	1.4	DGUOK	deoxyguanosine kinase	Upregulated
A_22_P000	0.001776	0.000243	1.4	TMPO-AS1	TMPO antisense RNA 1	Upregulated
A_23_P584	0.00183	0.000257	1.4	MGAT1	mannosyl (alpha-1,3-)-glycoprotein beta-1,2-N-acetylglucosaminyltransfe	Upregulated
A_33_P337	0.002203	0.000355	1.4	SGTB	small glutamine-rich tetratricopeptide repeat (TPR)-containing, beta	Upregulated
A_23_P664	0.00239	0.000401	1.4	MED9	mediator complex subunit 9	Upregulated
A_23_P214	0.002597	0.000456	1.4	COL10A1	collagen, type X, alpha 1	Upregulated
A_33_P335	0.002707	0.000485	1.4	IGBP1	immunoglobulin (CD79A) binding protein 1	Upregulated
A_23_P352	0.004554	0.00106	1.4	RCAN3	RCAN family member 3	Upregulated
A_21_P000	0.005261	0.00131	1.4	lnc-NUDT7-1	lnc-NUDT7-1:1	Upregulated
A_33_P369	0.006321	0.00171	1.4	LOC283485	uncharacterized LOC283485	Upregulated
A_24_P532	0.006348	0.00172	1.4	CPD	carboxypeptidase D	Upregulated
A_22_P000	0.006888	0.00192	1.4	PTOV1-AS1	PTOV1 antisense RNA 1	Upregulated
A_22_P000	0.010139	0.00326	1.4	lnc-API5-1	lnc-API5-1:1	Upregulated
A_33_P333	0.014014	0.00494	1.4	ARMCX6	armadillo repeat containing, X-linked 6	Upregulated
A_23_P302	0.017396	0.00655	1.4	SRD5A1	steroid-5-alpha-reductase, alpha polypeptide 1 (3-oxo-5 alpha-steroid d	Upregulated
A_32_P150	0.021642	0.00856	1.4	C17orf100	chromosome 17 open reading frame 100	Upregulated
A_22_P000	0.024408	0.00999	1.4	lnc-CNR1-1	lnc-CNR1-1:2	Upregulated
A_33_P327	0.029372	0.0126	1.4	PHLPP2	PH domain and leucine rich repeat protein phosphatase 2	Upregulated
A_23_P127	0.054093	0.0264	1.4	HYLS1	hydrolethalus syndrome 1	Upregulated
A_21_P000	0.090554	0.0492	1.4	lnc-CD83-2	lnc-CD83-2:1	Upregulated
A_23_P136	0.001232	0.000125	1.39	IMPAD1	inositol monophosphatase domain containing 1	Upregulated
A_32_P204	0.001347	0.000147	1.39	FABP5	fatty acid binding protein 5 (psoriasis-associated)	Upregulated
A_23_P117	0.001405	0.00016	1.39	KIAA0101	KIAA0101	Upregulated
A_33_P330	0.001569	0.000195	1.39	C1orf52	chromosome 1 open reading frame 52	Upregulated
A_24_P590	0.001753	0.000237	1.39	RRN3	RRN3 RNA polymerase I transcription factor homolog (S. cerevisiae)	Upregulated
A_23_P944	0.001934	0.000282	1.39	PDCD1LG2	programmed cell death 1 ligand 2	Upregulated
A_23_P314	0.002097	0.000326	1.39	BMI1	BMI1 proto-oncogene, polycomb ring finger	Upregulated
A_23_P208	0.002211	0.000356	1.39	CLEC4M	C-type lectin domain family 4, member M	Upregulated
A_23_P302	0.00299	0.000565	1.39	HMHB1	histocompatibility (minor) HB-1	Upregulated

A_33_P333	0.003458	0.000705	1.39	SP1	Sp1 transcription factor	Upregulated
A_22_P000	0.006239	0.00168	1.39	lnc-ENAH-1	lnc-ENAH-1:1	Upregulated
A_22_P000	0.010952	0.00358	1.39	LOC1019277	uncharacterized LOC101927740	Upregulated
A_22_P000	0.013454	0.00469	1.39	SLC50A1	solute carrier family 50 (sugar efflux transporter), member 1	Upregulated
A_33_P322	0.015611	0.0057	1.39	CCR10	chemokine (C-C motif) receptor 10	Upregulated
A_21_P000	0.072667	0.0375	1.39	LINC00485	long intergenic non-protein coding RNA 485	Upregulated
A_33_P340	0.001242	0.000127	1.38	TMEM120B	transmembrane protein 120B	Upregulated
A_23_P773	0.001435	0.000165	1.38	CETN3	centrin, EF-hand protein, 3	Upregulated
A_23_P257	0.001435	0.000166	1.38	TMEM117	transmembrane protein 117	Upregulated
A_23_P101	0.001583	0.000198	1.38	EML2	echinoderm microtubule associated protein like 2	Upregulated
A_23_P330	0.001594	0.0002	1.38	MAF1	MAF1 homolog (S. cerevisiae)	Upregulated
A_33_P333	0.001749	0.000236	1.38	HUS1	HUS1 checkpoint homolog (S. pombe)	Upregulated
A_24_P102	0.00176	0.000239	1.38	DNAJB2	DnaJ (Hsp40) homolog, subfamily B, member 2	Upregulated
A_24_P880	0.001839	0.000259	1.38	C8orf33	chromosome 8 open reading frame 33	Upregulated
A_23_P100	0.002072	0.00032	1.38	VKORC1	vitamin K epoxide reductase complex, subunit 1	Upregulated
A_22_P000	0.002117	0.000332	1.38	lnc-CTDSPL2	lnc-CTDSPL2-2:1	Upregulated
A_23_P118	0.002603	0.000458	1.38	C17orf62	chromosome 17 open reading frame 62	Upregulated
A_19_P003	0.002992	0.000566	1.38	LOC145474	uncharacterized LOC145474	Upregulated
A_33_P320	0.003548	0.000733	1.38	IARS	isoleucyl-tRNA synthetase	Upregulated
A_21_P000	0.005415	0.00137	1.38	lnc-HPDL-1	lnc-HPDL-1:1	Upregulated
A_22_P000	0.006474	0.00177	1.38	lnc-STAT1-2	lnc-STAT1-2:1	Upregulated
A_23_P156	0.008574	0.0026	1.38	STX11	syntaxin 11	Upregulated
A_22_P000	0.009631	0.00304	1.38	lnc-ZNF667-1	lnc-ZNF667-1:1	Upregulated
A_23_P128	0.010107	0.00324	1.38	RNF34	ring finger protein 34, E3 ubiquitin protein ligase	Upregulated
A_22_P000	0.010598	0.00344	1.38	lnc-SPAG1-4	lnc-SPAG1-4:2	Upregulated
A_33_P321	0.012467	0.00425	1.38	METTL6	methyltransferase like 6	Upregulated
A_21_P001	0.01384	0.00486	1.38	LINC01503	long intergenic non-protein coding RNA 1503	Upregulated
A_33_P337	0.021475	0.00848	1.38	LOC1001312	uncharacterized LOC100131242	Upregulated
A_21_P001	0.025049	0.0103	1.38	MUC19	mucin 19, oligomeric	Upregulated
A_23_P379	0.028169	0.0119	1.38	MAPK3	mitogen-activated protein kinase 3	Upregulated
A_22_P000	0.037267	0.0168	1.38	lnc-TSPYL6-1	lnc-TSPYL6-1:1	Upregulated
A_33_P331	0.045877	0.0217	1.38	OR5C1	olfactory receptor, family 5, subfamily C, member 1	Upregulated
A_23_P700	0.001222	0.000123	1.37	PFDN1	prefoldin subunit 1	Upregulated
A_23_P385	0.001241	0.000127	1.37	ARL8B	ADP-ribosylation factor-like 8B	Upregulated
A_24_P353	0.001398	0.000158	1.37	SLAMF7	SLAM family member 7	Upregulated
A_33_P340	0.001453	0.000171	1.37	GTF3C4	general transcription factor IIIC, polypeptide 4, 90kDa	Upregulated
A_24_P661	0.001474	0.000174	1.37	STAG2	stromal antigen 2	Upregulated

A_23_P247	0.001537	0.000188	1.37	OSBP	oxysterol binding protein	Upregulated
A_23_P108	0.001611	0.000204	1.37	YPEL5	yippee-like 5 (Drosophila)	Upregulated
A_33_P321	0.0017	0.000226	1.37	PANK3	pantothenate kinase 3	Upregulated
A_32_P193	0.001754	0.000237	1.37	FMNL2	formin-like 2	Upregulated
A_23_P672	0.001831	0.000257	1.37	PKN1	protein kinase N1	Upregulated
A_33_P332	0.001925	0.00028	1.37	ATXN7	ataxin 7	Upregulated
A_33_P324	0.002337	0.000387	1.37	MUC19	mucin 19, oligomeric	Upregulated
A_23_P258	0.00248	0.000424	1.37	MANBA	mannosidase, beta A, lysosomal	Upregulated
A_33_P321	0.00264	0.000467	1.37	IMPDH2	IMP (inosine 5'-monophosphate) dehydrogenase 2	Upregulated
A_24_P509	0.006118	0.00163	1.37	TRIM11	tripartite motif containing 11	Upregulated
A_32_P198	0.018743	0.00717	1.37	lnc-EPHA1-1	lnc-EPHA1-1:1	Upregulated
A_22_P000	0.018801	0.0072	1.37	lnc-CLGN-2	lnc-CLGN-2:1	Upregulated
A_23_P121	0.026279	0.011	1.37	PRSS12	protease, serine, 12 (neurotrypsin, motopsin)	Upregulated
A_21_P001	0.026279	0.011	1.37	ZDHHC11	zinc finger, DHHC-type containing 11	Upregulated
A_24_P348	0.044464	0.0208	1.37	LRRC8E	leucine rich repeat containing 8 family, member E	Upregulated
A_24_P935	0.04772	0.0227	1.37	ADCY9	adenylate cyclase 9	Upregulated
A_23_P433	0.051162	0.0247	1.37	SPG7	spastic paraplegia 7 (pure and complicated autosomal recessive)	Upregulated
A_21_P000	0.064134	0.0323	1.37	lnc-NPAT-1	lnc-NPAT-1:1	Upregulated
A_33_P328	0.001243	0.000128	1.36	RMND5A	required for meiotic nuclear division 5 homolog A (S. cerevisiae)	Upregulated
A_24_P196	0.001298	0.000138	1.36	KMT2E	lysine (K)-specific methyltransferase 2E	Upregulated
A_23_P157	0.001373	0.000152	1.36	MRPS33	mitochondrial ribosomal protein S33	Upregulated
A_33_P332	0.001398	0.000158	1.36	GAS5	growth arrest-specific 5 (non-protein coding)	Upregulated
A_24_P823	0.001478	0.000176	1.36	HSP90AB1	heat shock protein 90kDa alpha (cytosolic), class B member 1	Upregulated
A_23_P205	0.001484	0.000178	1.36	PSEN1	presenilin 1	Upregulated
A_23_P163	0.001563	0.000194	1.36	MTHFS	5,10-methenyltetrahydrofolate synthetase (5-formyltetrahydrofolate cyclo	Upregulated
A_33_P325	0.001884	0.000269	1.36	ZNF451	zinc finger protein 451	Upregulated
A_24_P276	0.002464	0.000419	1.36	PPT1	palmitoyl-protein thioesterase 1	Upregulated
A_32_P169	0.00275	0.000497	1.36	ALG1	ALG1, chitobiosyldiphosphodolichol beta-mannosyltransferase	Upregulated
A_23_P632	0.003291	0.000656	1.36	POGZ	pogo transposable element with ZNF domain	Upregulated
A_23_P327	0.007634	0.00222	1.36	KIAA0232	KIAA0232	Upregulated
A_24_P305	0.00852	0.00258	1.36	SMS	spermine synthase	Upregulated
A_23_P401	0.009741	0.00308	1.36	COL9A3	collagen, type IX, alpha 3	Upregulated
A_33_P324	0.010112	0.00325	1.36	DIAPH1	diaphanous-related formin 1	Upregulated
A_24_P283	0.011042	0.00363	1.36	NABP2	nucleic acid binding protein 2	Upregulated
A_32_P137	0.011404	0.00379	1.36	TRIM27	tripartite motif containing 27	Upregulated
A_24_P989	0.012763	0.00438	1.36	PFKM	phosphofructokinase, muscle	Upregulated
A_33_P338	0.001298	0.000138	1.35	EEF2KMT	eukaryotic elongation factor 2 lysine methyltransferase	Upregulated

A_23_P145	0.001347	0.000148	1.35	HSP90AB1	heat shock protein 90kDa alpha (cytosolic), class B member 1	Upregulated
A_33_P330	0.001397	0.000157	1.35	CPSF7	cleavage and polyadenylation specific factor 7, 59kDa	Upregulated
A_33_P341	0.00141	0.000161	1.35	SLC24A1	solute carrier family 24 (sodium/potassium/calcium exchanger), member	Upregulated
A_33_P321	0.001479	0.000176	1.35	POLR2A	polymerase (RNA) II (DNA directed) polypeptide A, 220kDa	Upregulated
A_32_P698	0.001517	0.000184	1.35	BEX5	brain expressed, X-linked 5	Upregulated
A_22_P000	0.00163	0.000209	1.35	lnc-PHYHD1-1	lnc-PHYHD1-1:1	Upregulated
A_22_P000	0.001808	0.00025	1.35	lnc-SLC12A7-	lnc-SLC12A7-1:2	Upregulated
A_32_P126	0.001944	0.000284	1.35	E2F6	E2F transcription factor 6	Upregulated
A_24_P309	0.00196	0.00029	1.35	SNN	stannin	Upregulated
A_19_P003	0.002571	0.000449	1.35	LOC1027251	uncharacterized LOC102725166	Upregulated
A_33_P332	0.002662	0.000472	1.35	RRM2B	ribonucleotide reductase M2 B (TP53 inducible)	Upregulated
A_33_P330	0.004565	0.00106	1.35	ZFAND4	zinc finger, AN1-type domain 4	Upregulated
A_33_P321	0.011063	0.00364	1.35	ATAD2	ATPase family, AAA domain containing 2	Upregulated
A_33_P321	0.011582	0.00388	1.35	ZC3H12A	zinc finger CCCH-type containing 12A	Upregulated
A_22_P000	0.012101	0.00409	1.35	CYB5B	cytochrome b5 type B (outer mitochondrial membrane)	Upregulated
A_33_P325	0.019729	0.00764	1.35	PAN3	PAN3 poly(A) specific ribonuclease subunit	Upregulated
A_23_P250	0.034507	0.0153	1.35	BRPF1	bromodomain and PHD finger containing, 1	Upregulated
A_33_P337	0.042114	0.0195	1.35	FAM98B	family with sequence similarity 98, member B	Upregulated
A_23_P184	0.001295	0.000137	1.34	TIMMDC1	translocase of inner mitochondrial membrane domain containing 1	Upregulated
A_32_P683	0.001337	0.000145	1.34	C5orf51	chromosome 5 open reading frame 51	Upregulated
A_23_P395	0.001375	0.000153	1.34	PWP1	PWP1 homolog (S. cerevisiae)	Upregulated
A_23_P191	0.001376	0.000153	1.34	TTC1	tetratricopeptide repeat domain 1	Upregulated
A_33_P324	0.001386	0.000155	1.34	PIGX	phosphatidylinositol glycan anchor biosynthesis, class X	Upregulated
A_32_P148	0.001469	0.000174	1.34	SNRPD1	small nuclear ribonucleoprotein D1 polypeptide 16kDa	Upregulated
A_23_P302	0.001479	0.000176	1.34	FAM174A	family with sequence similarity 174, member A	Upregulated
A_21_P001	0.001481	0.000177	1.34	XLOC_l2_015213		Upregulated
A_33_P325	0.001531	0.000187	1.34	FAM21C	family with sequence similarity 21, member C	Upregulated
A_33_P321	0.001605	0.000203	1.34	IL18R1	interleukin 18 receptor 1	Upregulated
A_24_P990	0.001854	0.000263	1.34	IPO5	importin 5	Upregulated
A_22_P000	0.002544	0.000442	1.34	lnc-S100A13-	lnc-S100A13-1:1	Upregulated
A_23_P362	0.002882	0.000534	1.34	SLC25A33	solute carrier family 25 (pyrimidine nucleotide carrier), member 33	Upregulated
A_22_P000	0.003054	0.000583	1.34	lnc-USP8-1	lnc-USP8-1:1	Upregulated
A_23_P170	0.003974	0.000865	1.34	CHCHD7	coiled-coil-helix-coiled-coil-helix domain containing 7	Upregulated
A_23_P977	0.004221	0.000948	1.34	ACBD5	acyl-CoA binding domain containing 5	Upregulated
A_19_P008	0.004467	0.00103	1.34	BMPR1A	bone morphogenetic protein receptor, type IA	Upregulated
A_23_P521	0.006304	0.0017	1.34	TBCE	tubulin folding cofactor E	Upregulated
A_21_P000	0.009878	0.00314	1.34	lnc-CTAGE5-1	lnc-CTAGE5-1:1	Upregulated

A_33_P326	0.011099	0.00365	1.34	ZNF546	zinc finger protein 546	Upregulated
A_22_P000	0.011787	0.00396	1.34	LOC1001290	uncharacterized LOC100129083	Upregulated
A_33_P328	0.014447	0.00515	1.34	ANKRD20A9I	ankyrin repeat domain 20 family, member A9, pseudogene	Upregulated
A_32_P352	0.02519	0.0104	1.34	LOC650293	seven transmembrane helix receptor	Upregulated
A_22_P000	0.027701	0.0117	1.34	USP12-AS1	USP12 antisense RNA 1	Upregulated
A_33_P324	0.031087	0.0135	1.34	LOC1005057	uncharacterized LOC100505739	Upregulated
A_23_P696	0.033521	0.0148	1.34	SEC24B	SEC24 family member B	Upregulated
A_22_P000	0.040455	0.0186	1.34	lnc-ACOT9-1	lnc-ACOT9-1:1	Upregulated
A_21_P001	0.048807	0.0233	1.34	LOC1005060	uncharacterized LOC100506023	Upregulated
A_21_P000	0.050765	0.0244	1.34	BEND7	BEN domain containing 7	Upregulated
A_33_P335	0.063294	0.0318	1.34	KIF22	kinesin family member 22	Upregulated
A_33_P377	0.001316	0.000142	1.33	SERAC1	serine active site containing 1	Upregulated
A_33_P334	0.001386	0.000155	1.33	HSPH1	heat shock 105kDa/110kDa protein 1	Upregulated
A_23_P110	0.001469	0.000174	1.33	SKIV2L2	superkiller viralicidic activity 2-like 2 (S. cerevisiae)	Upregulated
A_33_P322	0.002113	0.00033	1.33	UBE2E3	ubiquitin-conjugating enzyme E2E 3	Upregulated
A_33_P321	0.002241	0.000365	1.33	PAG1	phosphoprotein membrane anchor with glycosphingolipid microdomains	Upregulated
A_23_P492	0.002951	0.000553	1.33	C16orf87	chromosome 16 open reading frame 87	Upregulated
A_33_P359	0.003355	0.000675	1.33	PAPPA	pregnancy-associated plasma protein A, pappalysin 1	Upregulated
A_24_P202	0.004389	0.001	1.33	ITPKC	inositol-trisphosphate 3-kinase C	Upregulated
A_33_P335	0.004443	0.00102	1.33	ZNF416	zinc finger protein 416	Upregulated
A_23_P808	0.004454	0.00103	1.33	FYTTD1	forty-two-three domain containing 1	Upregulated
A_22_P000	0.005038	0.00123	1.33	lnc-ADAMTS1	lnc-ADAMTS18-6:1	Upregulated
A_23_P131	0.006324	0.00171	1.33	ACSBG2	acyl-CoA synthetase bubblegum family member 2	Upregulated
A_23_P214	0.008186	0.00244	1.33	PPARD	peroxisome proliferator-activated receptor delta	Upregulated
A_33_P325	0.016121	0.00594	1.33	ZNF134	zinc finger protein 134	Upregulated
A_21_P001	0.016713	0.00623	1.33	LOC441124	uncharacterized LOC441124	Upregulated
A_23_P501	0.019666	0.00761	1.33	ASB4	ankyrin repeat and SOCS box containing 4	Upregulated
A_23_P132	0.031217	0.0136	1.33	GLT8D1	glycosyltransferase 8 domain containing 1	Upregulated
A_21_P000	0.031596	0.0137	1.33	LOC1027235	uncharacterized LOC102723525	Upregulated
A_32_P853	0.039434	0.0181	1.33	ST20-AS1	ST20 antisense RNA 1	Upregulated
A_22_P000	0.04551	0.0215	1.33	lnc-KIAA1598	lnc-KIAA1598-1:1	Upregulated
A_23_P149	0.053536	0.026	1.33	PANK4	pantothenate kinase 4	Upregulated
A_21_P001	0.076749	0.04	1.33	lnc-CD93-1	lnc-CD93-1:1	Upregulated
A_23_P604	0.001314	0.000141	1.32	ZNF462	zinc finger protein 462	Upregulated
A_33_P338	0.001369	0.000152	1.32	FAM27B	family with sequence similarity 27, member B	Upregulated
A_23_P824	0.001621	0.000206	1.32	PUS7	pseudouridylate synthase 7 (putative)	Upregulated
A_33_P334	0.001657	0.000215	1.32	TTC5	tetratricopeptide repeat domain 5	Upregulated

A_23_P130	0.001694	0.000224	1.32	AURKB	aurora kinase B	Upregulated
A_23_P109	0.001753	0.000237	1.32	PSMD6	proteasome (prosome, macropain) 26S subunit, non-ATPase, 6	Upregulated
A_23_P604	0.001784	0.000245	1.32	UBAP2	ubiquitin associated protein 2	Upregulated
A_33_P330	0.001833	0.000258	1.32	LOC1001293	uncharacterized LOC100129363	Upregulated
A_33_P334	0.001908	0.000276	1.32	AKT1S1	AKT1 substrate 1 (proline-rich)	Upregulated
A_23_P502	0.001908	0.000276	1.32	PHF2	PHD finger protein 2	Upregulated
A_23_P359	0.00195	0.000286	1.32	EPC2	enhancer of polycomb homolog 2 (Drosophila)	Upregulated
A_21_P001	0.002062	0.000317	1.32	lnc-NFATC2-1	lnc-NFATC2-1:1	Upregulated
A_23_P249	0.002232	0.000362	1.32	CDK4	cyclin-dependent kinase 4	Upregulated
A_24_P417	0.002347	0.00039	1.32	FRG1B	FSHD region gene 1 family, member B	Upregulated
A_24_P241	0.002454	0.000416	1.32	DCAF4	DDB1 and CUL4 associated factor 4	Upregulated
A_24_P597	0.00247	0.000421	1.32	CTD-2151A2.	uncharacterized LOC102724855	Upregulated
A_33_P331	0.003343	0.000672	1.32	FANCC	Fanconi anemia, complementation group C	Upregulated
A_33_P327	0.003667	0.000769	1.32	ZIK1	zinc finger protein interacting with K protein 1	Upregulated
A_21_P001	0.005054	0.00123	1.32	XLOC_l2_004594		Upregulated
A_33_P336	0.005296	0.00132	1.32	BRMS1	breast cancer metastasis suppressor 1	Upregulated
A_23_P936	0.006372	0.00173	1.32	ASIC3	acid sensing (proton gated) ion channel 3	Upregulated
A_23_P148	0.008614	0.00262	1.32	PTGER4	prostaglandin E receptor 4 (subtype EP4)	Upregulated
A_21_P001	0.009666	0.00305	1.32	PHF21B	PHD finger protein 21B	Upregulated
A_33_P342	0.023957	0.00974	1.32	OR4D6	olfactory receptor, family 4, subfamily D, member 6	Upregulated
A_22_P000	0.025963	0.0108	1.32	lnc-C16orf88-	lnc-C16orf88-1:3	Upregulated
A_21_P000	0.043671	0.0204	1.32	lnc-SGPP1-1	lnc-SGPP1-1:1	Upregulated
A_23_P598	0.054346	0.0265	1.32	METTL2B	methyltransferase like 2B	Upregulated
A_23_P426	0.001385	0.000155	1.31	POLR2J	polymerase (RNA) II (DNA directed) polypeptide J, 13.3kDa	Upregulated
A_32_P430	0.001394	0.000157	1.31	FRG1	FSHD region gene 1	Upregulated
A_23_P384	0.001398	0.000158	1.31	TAOK1	TAO kinase 1	Upregulated
A_23_P310	0.001444	0.000168	1.31	RANBP3	RAN binding protein 3	Upregulated
A_33_P340	0.001522	0.000185	1.31	C6orf226	chromosome 6 open reading frame 226	Upregulated
A_33_P326	0.001537	0.000188	1.31	LOC1001311	uncharacterized LOC100131132	Upregulated
A_22_P000	0.00163	0.000209	1.31	lnc-TLCD2-2	lnc-TLCD2-2:1	Upregulated
A_24_P400	0.001894	0.000272	1.31	TMC8	transmembrane channel-like 8	Upregulated
A_33_P337	0.00191	0.000276	1.31	PLEKHO1	pleckstrin homology domain containing, family O member 1	Upregulated
A_23_P616	0.001991	0.000298	1.31	CLK4	CDC-like kinase 4	Upregulated
A_24_P166	0.002027	0.000308	1.31	CDK6	cyclin-dependent kinase 6	Upregulated
A_19_P003	0.002721	0.000489	1.31	ZNF83	zinc finger protein 83	Upregulated
A_23_P167	0.00302	0.000574	1.31	PRPF40A	PRP40 pre-mRNA processing factor 40 homolog A (S. cerevisiae)	Upregulated
A_32_P353	0.003438	0.000699	1.31	PTDSS2	phosphatidylserine synthase 2	Upregulated

A_21_P001	0.004356	0.000994	1.31	DUX4	double homeobox 4	Upregulated
A_24_P353	0.004466	0.00103	1.31	GUCD1	guanylyl cyclase domain containing 1	Upregulated
A_24_P943	0.005753	0.00149	1.31	ARL5B	ADP-ribosylation factor-like 5B	Upregulated
A_33_P341	0.007033	0.00198	1.31	lnc-HJURP-1	lnc-HJURP-1:1	Upregulated
A_33_P335	0.00708	0.002	1.31	SMG7	SMG7 nonsense mediated mRNA decay factor	Upregulated
A_33_P340	0.008114	0.00241	1.31	EPHA6	EPH receptor A6	Upregulated
A_24_P218	0.013137	0.00455	1.31	MED17	mediator complex subunit 17	Upregulated
A_33_P333	0.031837	0.0139	1.31	ABCB4	ATP-binding cassette, sub-family B (MDR/TAP), member 4	Upregulated
A_23_P112	0.048955	0.0234	1.31	GSDMD	gasdermin D	Upregulated
A_24_P159	0.001559	0.000193	1.3	PDCD7	programmed cell death 7	Upregulated
A_23_P365	0.001626	0.000208	1.3	PPP1R14B	protein phosphatase 1, regulatory (inhibitor) subunit 14B	Upregulated
A_23_P159	0.001691	0.000223	1.3	TAF15	TAF15 RNA polymerase II, TATA box binding protein (TBP)-associated f	Upregulated
A_33_P339	0.001793	0.000247	1.3	LOC727721	uncharacterized LOC727721	Upregulated
A_23_P419	0.001879	0.000268	1.3	CCDC127	coiled-coil domain containing 127	Upregulated
A_23_P413	0.002002	0.000301	1.3	ABCE1	ATP-binding cassette, sub-family E (OABP), member 1	Upregulated
A_21_P000	0.002229	0.000362	1.3	LARP4	La ribonucleoprotein domain family, member 4	Upregulated
A_23_P148	0.002428	0.00041	1.3	CSTF2	cleavage stimulation factor, 3' pre-RNA, subunit 2, 64kDa	Upregulated
A_23_P111	0.002495	0.000428	1.3	TBC1D7	TBC1 domain family, member 7	Upregulated
A_23_P132	0.002528	0.000437	1.3	EIF4ENIF1	eukaryotic translation initiation factor 4E nuclear import factor 1	Upregulated
A_23_P453	0.002952	0.000554	1.3	HTATSF1	HIV-1 Tat specific factor 1	Upregulated
A_22_P000	0.002984	0.000562	1.3	lnc-CES1-3	lnc-CES1-3:1	Upregulated
A_24_P632	0.003293	0.000657	1.3	RPN1	ribophorin I	Upregulated
A_21_P000	0.004207	0.000941	1.3	LOC1009963	uncharacterized LOC100996324	Upregulated
A_23_P212	0.004498	0.00104	1.3	TTC14	tetratricopeptide repeat domain 14	Upregulated
A_23_P165	0.008932	0.00275	1.3	EIF4E2	eukaryotic translation initiation factor 4E family member 2	Upregulated
A_22_P000	0.009837	0.00312	1.3	lnc-PKLR-2	lnc-PKLR-2:1	Upregulated
A_33_P333	0.02036	0.00794	1.3	ABCB8	ATP-binding cassette, sub-family B (MDR/TAP), member 8	Upregulated
A_21_P000	0.022763	0.00912	1.3	PGM3	phosphoglucomutase 3	Upregulated
A_21_P001	0.023746	0.00963	1.3	XLOC_l2_007829		Upregulated
A_24_P110	0.028148	0.0119	1.3	CSAG1	chondrosarcoma associated gene 1	Upregulated
A_23_P695	0.07597	0.0396	1.3	GUCY1A3	guanylate cyclase 1, soluble, alpha 3	Upregulated
A_33_P340	0.001443	0.000168	1.29	IL4I1	interleukin 4 induced 1	Upregulated
A_33_P321	0.001483	0.000177	1.29	SLC35F2	solute carrier family 35, member F2	Upregulated
A_23_P218	0.001597	0.000201	1.29	XPNPEP3	X-prolyl aminopeptidase (aminopeptidase P) 3, putative	Upregulated
A_23_P347	0.001603	0.000202	1.29	SP3	Sp3 transcription factor	Upregulated
A_23_P706	0.001612	0.000204	1.29	CD83	CD83 molecule	Upregulated
A_33_P336	0.001626	0.000208	1.29	HIBCH	3-hydroxyisobutyryl-CoA hydrolase	Upregulated

A_21_P001	0.001919	0.000279	1.29	LINC00158	long intergenic non-protein coding RNA 158	Upregulated
A_23_P128	0.001949	0.000286	1.29	RAB21	RAB21, member RAS oncogene family	Upregulated
A_23_P358	0.001958	0.000289	1.29	POPDC3	popeye domain containing 3	Upregulated
A_22_P000	0.002511	0.000433	1.29	LOC1019287	uncharacterized LOC101928737	Upregulated
A_24_P302	0.002728	0.000491	1.29	FCHSD2	FCH and double SH3 domains 2	Upregulated
A_23_P102	0.002834	0.00052	1.29	COQ10B	coenzyme Q10 homolog B (S. cerevisiae)	Upregulated
A_24_P642	0.002872	0.00053	1.29	ALDH1B1	aldehyde dehydrogenase 1 family, member B1	Upregulated
A_23_P658	0.003163	0.000618	1.29	FBXO22	F-box protein 22	Upregulated
A_24_P187	0.003199	0.00063	1.29	CNOT4	CCR4-NOT transcription complex, subunit 4	Upregulated
A_24_P391	0.003224	0.000636	1.29	PTTG1IP	pituitary tumor-transforming 1 interacting protein	Upregulated
A_21_P001	0.00452	0.00105	1.29	XLOC_l2_015438		Upregulated
A_33_P341	0.006535	0.00179	1.29	ZNF891	zinc finger protein 891	Upregulated
A_24_P377	0.008344	0.00251	1.29	OSBPL3	oxysterol binding protein-like 3	Upregulated
A_33_P335	0.008765	0.00268	1.29	LOC403323	uncharacterized LOC403323	Upregulated
A_23_P378	0.011229	0.00371	1.29	TMEM64	transmembrane protein 64	Upregulated
A_33_P342	0.015816	0.00579	1.29	ZNF710	zinc finger protein 710	Upregulated
A_22_P000	0.01767	0.00667	1.29	lnc-CTSL2-2	lnc-CTSL2-2:1	Upregulated
A_21_P001	0.01767	0.00667	1.29	LOC646588	uncharacterized LOC646588	Upregulated
A_33_P384	0.018047	0.00685	1.29	LINC01281	long intergenic non-protein coding RNA 1281	Upregulated
A_22_P000	0.025564	0.0106	1.29	lnc-GJA4-1	lnc-GJA4-1:1	Upregulated
A_23_P124	0.026603	0.0111	1.29	CLCN3	chloride channel, voltage-sensitive 3	Upregulated
A_33_P339	0.030769	0.0133	1.29	LRP1	low density lipoprotein receptor-related protein 1	Upregulated
A_19_P003	0.03725	0.0168	1.29	LINC01013	long intergenic non-protein coding RNA 1013	Upregulated
A_33_P327	0.03725	0.0168	1.29	NMT2	N-myristoyltransferase 2	Upregulated
A_21_P001	0.059747	0.0297	1.29	XLOC_l2_004385		Upregulated
A_23_P106	0.001475	0.000175	1.28	NFKBIA	nuclear factor of kappa light polypeptide gene enhancer in B-cells inhibi	Upregulated
A_23_P415	0.001543	0.000189	1.28	HIST1H4E	histone cluster 1, H4e	Upregulated
A_24_P700	0.001699	0.000226	1.28	LATS2	large tumor suppressor kinase 2	Upregulated
A_33_P337	0.001812	0.000252	1.28	NAMPT	nicotinamide phosphoribosyltransferase	Upregulated
A_23_P656	0.001819	0.000254	1.28	GTF2A1	general transcription factor IIA, 1, 19/37kDa	Upregulated
A_23_P409	0.001854	0.000263	1.28	USP13	ubiquitin specific peptidase 13 (isopeptidase T-3)	Upregulated
A_23_P602	0.002179	0.000349	1.28	EIF4B	eukaryotic translation initiation factor 4B	Upregulated
A_24_P299	0.002768	0.000502	1.28	FAM101B	family with sequence similarity 101, member B	Upregulated
A_23_P134	0.002944	0.00055	1.28	BTBD10	BTB (POZ) domain containing 10	Upregulated
A_23_P156	0.003795	0.000808	1.28	C17orf75	chromosome 17 open reading frame 75	Upregulated
A_22_P000	0.003997	0.000872	1.28	LINC01252	long intergenic non-protein coding RNA 1252	Upregulated
A_23_P113	0.004324	0.000982	1.28	KATNA1	katanin p60 (ATPase containing) subunit A 1	Upregulated

A_23_P251	0.004678	0.0011	1.28	NF2	neurofibromin 2 (merlin)	Upregulated
A_32_P703	0.005244	0.0013	1.28	LOC646626	uncharacterized LOC646626	Upregulated
A_22_P000	0.007062	0.002	1.28	lnc-PRKACG-	lnc-PRKACG-3:1	Upregulated
A_24_P172	0.008584	0.0026	1.28	GMFB	glia maturation factor, beta	Upregulated
A_22_P000	0.018659	0.00713	1.28	lnc-MRAP-1	lnc-MRAP-1:1	Upregulated
A_24_P223	0.026736	0.0112	1.28	NAF1	nuclear assembly factor 1 ribonucleoprotein	Upregulated
A_22_P000	0.038392	0.0175	1.28	lnc-KCNMB1-:	lnc-KCNMB1-3:2	Upregulated
A_22_P000	0.042136	0.0196	1.28	lnc-THBS4-3	lnc-THBS4-3:1	Upregulated
A_33_P331	0.042471	0.0197	1.28	UHRF1BP1	UHRF1 binding protein 1	Upregulated
A_21_P000	0.067234	0.0342	1.28	lnc-SH3GL3-1	lnc-SH3GL3-1:1	Upregulated
A_24_P433	0.001443	0.000167	1.27	RNF38	ring finger protein 38	Upregulated
A_23_P130	0.001481	0.000177	1.27	ROCK1	Rho-associated, coiled-coil containing protein kinase 1	Upregulated
A_23_P140	0.001731	0.000232	1.27	NDUFB1	NADH dehydrogenase (ubiquinone) 1 beta subcomplex, 1, 7kDa	Upregulated
A_23_P411	0.001825	0.000256	1.27	OSTC	oligosaccharyltransferase complex subunit (non-catalytic)	Upregulated
A_24_P298	0.002075	0.00032	1.27	CBX1	chromobox homolog 1	Upregulated
A_33_P370	0.002124	0.000335	1.27	lnc-ZNF717-1	lnc-ZNF717-1:1	Upregulated
A_23_P363	0.002748	0.000496	1.27	SLC38A1	solute carrier family 38, member 1	Upregulated
A_33_P336	0.002951	0.000553	1.27	YIF1A	Yip1 interacting factor homolog A (S. cerevisiae)	Upregulated
A_23_P152	0.003041	0.00058	1.27	ENGASE	endo-beta-N-acetylglucosaminidase	Upregulated
A_22_P000	0.003311	0.000662	1.27	STX6	syntaxin 6	Upregulated
A_23_P432	0.004321	0.000981	1.27	PMM2	phosphomannomutase 2	Upregulated
A_21_P001	0.004882	0.00117	1.27	LOC1027233	uncharacterized LOC102723382	Upregulated
A_23_P122	0.006012	0.00159	1.27	MSH3	mutS homolog 3	Upregulated
A_33_P333	0.006219	0.00167	1.27	TMEM53	transmembrane protein 53	Upregulated
A_23_P108	0.011539	0.00385	1.27	STK16	serine/threonine kinase 16	Upregulated
A_23_P158	0.020623	0.00807	1.27	BRIP1	BRCA1 interacting protein C-terminal helicase 1	Upregulated
A_21_P000	0.035323	0.0158	1.27	LOC1005065	uncharacterized LOC100506548	Upregulated
A_33_P326	0.037112	0.0168	1.27	RAP1GAP	RAP1 GTPase activating protein	Upregulated
A_33_P334	0.051781	0.025	1.27	LOC284788	uncharacterized LOC284788	Upregulated
A_22_P000	0.075898	0.0395	1.27	lnc-CDKAL1-1	lnc-CDKAL1-1:1	Upregulated
A_22_P000	0.075898	0.0395	1.27	RORB-AS1	RORB antisense RNA 1	Upregulated
A_33_P322	0.001453	0.00017	1.26	NCF1	neutrophil cytosolic factor 1	Upregulated
A_22_P000	0.001481	0.000177	1.26	lnc-FAM184B	lnc-FAM184B-1:1	Upregulated
A_32_P519	0.001552	0.000191	1.26	KIAA1191	KIAA1191	Upregulated
A_33_P340	0.001595	0.0002	1.26	BOD1L1	biorientation of chromosomes in cell division 1-like 1	Upregulated
A_21_P001	0.001634	0.00021	1.26	LOC374443	C-type lectin domain family 2, member D pseudogene	Upregulated
A_23_P150	0.001661	0.000216	1.26	C11orf1	chromosome 11 open reading frame 1	Upregulated

A_23_P211	0.001663	0.000217	1.26	TCTEX1D2	Tctex1 domain containing 2	Upregulated
A_33_P325	0.001682	0.00022	1.26	TRIM33	tripartite motif containing 33	Upregulated
A_23_P859	0.001693	0.000223	1.26	ZNF326	zinc finger protein 326	Upregulated
A_33_P335	0.001695	0.000224	1.26	MYH9	myosin, heavy chain 9, non-muscle	Upregulated
A_24_P335	0.001859	0.000264	1.26	ZNF266	zinc finger protein 266	Upregulated
A_24_P127	0.00191	0.000276	1.26	ST13	suppression of tumorigenicity 13 (colon carcinoma) (Hsp70 interacting pı	Upregulated
A_22_P000	0.001986	0.000297	1.26	lnc-ARL6IP5-	lnc-ARL6IP5-1:1	Upregulated
A_24_P193	0.001996	0.000299	1.26	PIGN	phosphatidylinositol glycan anchor biosynthesis, class N	Upregulated
A_24_P743	0.002028	0.000308	1.26	CTSA	cathepsin A	Upregulated
A_23_P825	0.002034	0.00031	1.26	ABCB1	ATP-binding cassette, sub-family B (MDR/TAP), member 1	Upregulated
A_24_P134	0.002179	0.000349	1.26	SPATA13	spermatogenesis associated 13	Upregulated
A_19_P003	0.002283	0.000375	1.26	UBA6-AS1	UBA6 antisense RNA 1 (head to head)	Upregulated
A_23_P641	0.00241	0.000406	1.26	CARD16	caspase recruitment domain family, member 16	Upregulated
A_33_P338	0.002482	0.000424	1.26	GTSF1	gametocyte specific factor 1	Upregulated
A_32_P896	0.002765	0.000501	1.26	ALG10B	ALG10B, alpha-1,2-glucosyltransferase	Upregulated
A_23_P775	0.003584	0.000746	1.26	SSTR5	somatostatin receptor 5	Upregulated
A_23_P516	0.005013	0.00122	1.26	ATP13A1	ATPase type 13A1	Upregulated
A_19_P003	0.007111	0.00202	1.26	lnc-UBLCP1-4	lnc-UBLCP1-4:1	Upregulated
A_22_P000	0.007339	0.0021	1.26	lnc-SPATA9-1	lnc-SPATA9-1:1	Upregulated
A_33_P330	0.007505	0.00217	1.26	GPT	glutamic-pyruvate transaminase (alanine aminotransferase)	Upregulated
A_24_P845	0.017312	0.00651	1.26	SPAG9	sperm associated antigen 9	Upregulated
A_33_P324	0.021031	0.00826	1.26	FAIM2	Fas apoptotic inhibitory molecule 2	Upregulated
A_33_P333	0.030025	0.0129	1.26	SLC26A11	solute carrier family 26 (anion exchanger), member 11	Upregulated
A_23_P800	0.036295	0.0163	1.26	FER1L4	fer-1-like family member 4, pseudogene (functional)	Upregulated
A_33_P326	0.036295	0.0163	1.26	NR6A1	nuclear receptor subfamily 6, group A, member 1	Upregulated
A_21_P000	0.052813	0.0256	1.26	lnc-LRP8-1	lnc-LRP8-1:1	Upregulated
A_24_P344	0.00151	0.000182	1.25	AGPAT3	1-acylglycerol-3-phosphate O-acyltransferase 3	Upregulated
A_32_P846	0.001644	0.000212	1.25	RPS23	ribosomal protein S23	Upregulated
A_32_P199	0.001771	0.000242	1.25	HSP90AA1	heat shock protein 90kDa alpha (cytosolic), class A member 1	Upregulated
A_32_P179	0.002037	0.000311	1.25	LOC1019296	uncharacterized LOC101929687	Upregulated
A_33_P322	0.002087	0.000324	1.25	EIF1AX	eukaryotic translation initiation factor 1A, X-linked	Upregulated
A_24_P790	0.002097	0.000326	1.25	TGFB1	transforming growth factor, beta 1	Upregulated
A_33_P322	0.002251	0.000368	1.25	DKFZp434J0	uncharacterized LOC93429	Upregulated
A_23_P159	0.002452	0.000415	1.25	VEGFB	vascular endothelial growth factor B	Upregulated
A_33_P329	0.002734	0.000492	1.25	BAG1	BCL2-associated athanogene	Upregulated
A_33_P321	0.002775	0.000504	1.25	AP5Z1	adaptor-related protein complex 5, zeta 1 subunit	Upregulated
A_33_P332	0.002901	0.000539	1.25	NRSN2	neurensin 2	Upregulated

A_33_P380	0.002952	0.000554	1.25	SPATS2	spermatogenesis associated, serine-rich 2	Upregulated
A_23_P699	0.003231	0.000638	1.25	RARS	arginyl-tRNA synthetase	Upregulated
A_24_P690	0.003515	0.000721	1.25	SEC23B	Sec23 homolog B (S. cerevisiae)	Upregulated
A_22_P000	0.003914	0.000847	1.25	lnc-FBN2-6	lnc-FBN2-6:1	Upregulated
A_23_P204	0.004106	0.000906	1.25	MPHOSPH8	M-phase phosphoprotein 8	Upregulated
A_21_P000	0.004154	0.000922	1.25	lnc-PFKP-9	lnc-PFKP-9:1	Upregulated
A_32_P662	0.006163	0.00164	1.25	ANKRD20A2	ankyrin repeat domain 20 family, member A2	Upregulated
A_33_P338	0.006791	0.00189	1.25	STXBP2	syntaxin binding protein 2	Upregulated
A_23_P872	0.007225	0.00206	1.25	MRPL17	mitochondrial ribosomal protein L17	Upregulated
A_33_P323	0.009552	0.003	1.25	TET2	tet methylcytosine dioxygenase 2	Upregulated
A_33_P339	0.0141	0.00498	1.25	SPATA25	spermatogenesis associated 25	Upregulated
A_33_P329	0.014691	0.00527	1.25	ALG2	ALG2, alpha-1,3/1,6-mannosyltransferase	Upregulated
A_21_P000	0.019729	0.00764	1.25	lnc-ZRANB1-2	lnc-ZRANB1-2:1	Upregulated
A_24_P522	0.01975	0.00765	1.25	ERICH3	glutamate-rich 3	Upregulated
A_23_P200	0.020694	0.0081	1.25	LRP8	low density lipoprotein receptor-related protein 8, apolipoprotein e recep	Upregulated
A_33_P336	0.023746	0.00963	1.25	SCARNA5	small Cajal body-specific RNA 5	Upregulated
A_23_P629	0.030032	0.0129	1.25	PBX1	pre-B-cell leukemia homeobox 1	Upregulated
A_23_P435	0.0414	0.0191	1.25	GPC4	glypican 4	Upregulated
A_32_P225	0.001497	0.00018	1.24	UTS2B	urotensin 2B	Upregulated
A_23_P137	0.001503	0.000181	1.24	ENO1	enolase 1, (alpha)	Upregulated
A_23_P105	0.001522	0.000185	1.24	ATL3	atlastin GTPase 3	Upregulated
A_23_P752	0.001523	0.000185	1.24	SLC25A28	solute carrier family 25 (mitochondrial iron transporter), member 28	Upregulated
A_24_P927	0.001552	0.000191	1.24	OXNAD1	oxidoreductase NAD-binding domain containing 1	Upregulated
A_32_P114	0.001606	0.000203	1.24	CACYBP	calcyclin binding protein	Upregulated
A_23_P293	0.001661	0.000216	1.24	RYBP	RING1 and YY1 binding protein	Upregulated
A_24_P340	0.001804	0.00025	1.24	NDUFAF4P1	NADH dehydrogenase (ubiquinone) complex I, assembly factor 4 pseud	Upregulated
A_19_P008	0.001908	0.000275	1.24	FTL	ferritin, light polypeptide	Upregulated
A_23_P125	0.002025	0.000307	1.24	PGK1	phosphoglycerate kinase 1	Upregulated
A_23_P160	0.002034	0.00031	1.24	SLC19A2	solute carrier family 19 (thiamine transporter), member 2	Upregulated
A_32_P100	0.0021	0.000326	1.24	FLJ37453	uncharacterized LOC729614	Upregulated
A_24_P209	0.002455	0.000416	1.24	MRPS10	mitochondrial ribosomal protein S10	Upregulated
A_24_P286	0.002682	0.000478	1.24	PURB	purine-rich element binding protein B	Upregulated
A_23_P131	0.006395	0.00174	1.24	VPS54	vacuolar protein sorting 54 homolog (S. cerevisiae)	Upregulated
A_23_P116	0.006443	0.00176	1.24	IFT46	intraflagellar transport 46	Upregulated
A_22_P000	0.006628	0.00183	1.24	LOC1019270	uncharacterized LOC101927043	Upregulated
A_23_P358	0.006753	0.00187	1.24	PML	promyelocytic leukemia	Upregulated
A_23_P144	0.011081	0.00364	1.24	CCRN4L	CCR4 carbon catabolite repression 4-like (S. cerevisiae)	Upregulated

A_23_P125	0.014474	0.00517	1.24	SLC26A11	solute carrier family 26 (anion exchanger), member 11	Upregulated
A_23_P969	0.021235	0.00836	1.24	SYNC	syncoilin, intermediate filament protein	Upregulated
A_21_P000	0.022183	0.00883	1.24	ZBED6	zinc finger, BED-type containing 6	Upregulated
A_23_P259	0.025418	0.0105	1.24	KCND2	potassium channel, voltage gated Shal related subfamily D, member 2	Upregulated
A_23_P139	0.025552	0.0106	1.24	GALNT18	polypeptide N-acetylgalactosaminyltransferase 18	Upregulated
A_23_P484	0.030335	0.0131	1.24	CCNA1	cyclin A1	Upregulated
A_21_P000	0.032013	0.014	1.24	LOC1019277	uncharacterized LOC101927780	Upregulated
A_21_P001	0.037423	0.0169	1.24	FOXP4-AS1	FOXP4 antisense RNA 1	Upregulated
A_33_P321	0.040083	0.0184	1.24	HIRIP3	HIRA interacting protein 3	Upregulated
A_22_P000	0.05525	0.0271	1.24	lnc-AKIRIN1-1	lnc-AKIRIN1-1:1	Upregulated
A_22_P000	0.057401	0.0283	1.24	lnc-TAF15-1	lnc-TAF15-1:1	Upregulated
A_22_P000	0.058736	0.0291	1.24	CREB1	cAMP responsive element binding protein 1	Upregulated
A_23_P420	0.070072	0.0359	1.24	SLC22A16	solute carrier family 22 (organic cation/carnitine transporter), member 16	Upregulated
A_32_P800	0.001889	0.000271	1.23	CLEC2D	C-type lectin domain family 2, member D	Upregulated
A_32_P743	0.001908	0.000276	1.23	VCPIP1	valosin containing protein (p97)/p47 complex interacting protein 1	Upregulated
A_33_P340	0.002006	0.000302	1.23	ANP32E	acidic (leucine-rich) nuclear phosphoprotein 32 family, member E	Upregulated
A_33_P333	0.002024	0.000307	1.23	HSD17B12	hydroxysteroid (17-beta) dehydrogenase 12	Upregulated
A_33_P334	0.002132	0.000338	1.23	CHEK1	checkpoint kinase 1	Upregulated
A_23_P205	0.002163	0.000344	1.23	GMFB	glia maturation factor, beta	Upregulated
A_23_P217	0.002968	0.000559	1.23	RGS16	regulator of G-protein signaling 16	Upregulated
A_33_P335	0.003003	0.000569	1.23	POU3F1	POU class 3 homeobox 1	Upregulated
A_23_P344	0.004051	0.000889	1.23	TMEM39B	transmembrane protein 39B	Upregulated
A_33_P339	0.004084	0.000898	1.23	LINC01160	long intergenic non-protein coding RNA 1160	Upregulated
A_23_P201	0.004491	0.00104	1.23	DESI2	desumoylating isopeptidase 2	Upregulated
A_23_P128	0.004763	0.00113	1.23	FKBP4	FK506 binding protein 4, 59kDa	Upregulated
A_23_P407	0.005058	0.00123	1.23	PARVB	parvin, beta	Upregulated
A_23_P661	0.006203	0.00166	1.23	KNOP1	lysine-rich nucleolar protein 1	Upregulated
A_23_P204	0.011684	0.00392	1.23	SLC41A2	solute carrier family 41 (magnesium transporter), member 2	Upregulated
A_33_P342	0.013296	0.00462	1.23	lnc-TMED5-1	lnc-TMED5-1:28	Upregulated
A_21_P001	0.015312	0.00556	1.23	TPTE2	transmembrane phosphoinositide 3-phosphatase and tensin homolog 2	Upregulated
A_32_P326	0.021987	0.00874	1.23	SENP5	SUMO1/sentrin specific peptidase 5	Upregulated
A_33_P326	0.03179	0.0138	1.23	B3GALT5-AS	B3GALT5 antisense RNA 1	Upregulated
A_21_P001	0.033281	0.0146	1.23	XLOC_l2_012366		Upregulated
A_23_P253	0.042047	0.0195	1.23	UNC13B	unc-13 homolog B (C. elegans)	Upregulated
A_23_P629	0.001532	0.000187	1.22	ATF6	activating transcription factor 6	Upregulated
A_23_P654	0.001603	0.000202	1.22	RAB2B	RAB2B, member RAS oncogene family	Upregulated
A_22_P000	0.001606	0.000203	1.22	LRRC75A-AS	LRRC75A antisense RNA 1	Upregulated

A_24_P831	0.00183	0.000257	1.22	AFF4	AF4/FMR2 family, member 4	Upregulated
A_23_P255	0.001848	0.000261	1.22	RNF14	ring finger protein 14	Upregulated
A_33_P326	0.001965	0.000292	1.22	FAM120AOS	family with sequence similarity 120A opposite strand	Upregulated
A_23_P119	0.0021	0.000326	1.22	CNNM4	cyclin and CBS domain divalent metal cation transport mediator 4	Upregulated
A_24_P370	0.002124	0.000334	1.22	ZMYM6NB	ZMYM6 neighbor	Upregulated
A_23_P258	0.002241	0.000365	1.22	PSMD10	proteasome (prosome, macropain) 26S subunit, non-ATPase, 10	Upregulated
A_33_P322	0.002361	0.000394	1.22	LOC1027248	uncharacterized LOC102724804	Upregulated
A_33_P341	0.002579	0.000452	1.22	RNF220	ring finger protein 220	Upregulated
A_23_P155	0.003178	0.000623	1.22	GFM1	G elongation factor, mitochondrial 1	Upregulated
A_19_P008	0.003184	0.000625	1.22	lnc-FBXW4-1	lnc-FBXW4-1:1	Upregulated
A_33_P332	0.003668	0.00077	1.22	LOC389906	zinc finger protein 839 pseudogene	Upregulated
A_24_P543	0.003779	0.000803	1.22	RASGRP3	RAS guanyl releasing protein 3 (calcium and DAG-regulated)	Upregulated
A_21_P001	0.004983	0.00121	1.22	DGCR5	DiGeorge syndrome critical region gene 5 (non-protein coding)	Upregulated
A_21_P001	0.005238	0.0013	1.22	LINC00454	long intergenic non-protein coding RNA 454	Upregulated
A_23_P678	0.005382	0.00135	1.22	ADCY3	adenylate cyclase 3	Upregulated
A_21_P001	0.005847	0.00152	1.22	ZNF807	zinc finger protein 807	Upregulated
A_22_P000	0.006753	0.00187	1.22	lnc-PEA15-1	lnc-PEA15-1:1	Upregulated
A_33_P328	0.010886	0.00356	1.22	SP6	Sp6 transcription factor	Upregulated
A_21_P001	0.032067	0.014	1.22	C1orf61	chromosome 1 open reading frame 61	Upregulated
A_33_P334	0.03211	0.014	1.22	LSM14B	LSM14B, SCD6 homolog B (S. cerevisiae)	Upregulated
A_33_P332	0.056476	0.0278	1.22	PRR26	proline rich 26	Upregulated
A_23_P215	0.078611	0.0412	1.22	HBP1	HMG-box transcription factor 1	Upregulated
A_24_P586	0.001617	0.000206	1.21	LOC1001333	uncharacterized LOC100133331	Upregulated
A_24_P154	0.001687	0.000221	1.21	ECE1	endothelin converting enzyme 1	Upregulated
A_24_P319	0.001687	0.000221	1.21	SUMO1	small ubiquitin-like modifier 1	Upregulated
A_24_P924	0.001972	0.000293	1.21	VEZF1	vascular endothelial zinc finger 1	Upregulated
A_24_P402	0.002034	0.00031	1.21	MBP	myelin basic protein	Upregulated
A_33_P331	0.002083	0.000323	1.21	PRKAA1	protein kinase, AMP-activated, alpha 1 catalytic subunit	Upregulated
A_33_P331	0.002166	0.000345	1.21	USF2	upstream transcription factor 2, c-fos interacting	Upregulated
A_23_P259	0.002309	0.000381	1.21	TKTL1	transketolase-like 1	Upregulated
A_23_P192	0.003949	0.000858	1.21	SDHAF2	succinate dehydrogenase complex assembly factor 2	Upregulated
A_21_P001	0.005296	0.00132	1.21	lnc-MMRN1-2	lnc-MMRN1-2:3	Upregulated
A_23_P593	0.005417	0.00137	1.21	CEP57L1	centrosomal protein 57kDa-like 1	Upregulated
A_23_P141	0.00612	0.00163	1.21	ZNF688	zinc finger protein 688	Upregulated
A_23_P994	0.009925	0.00316	1.21	BRCA2	breast cancer 2, early onset	Upregulated
A_21_P000	0.009925	0.00316	1.21	lnc-TNFSF11-	lnc-TNFSF11-1:1	Upregulated
A_24_P321	0.011487	0.00383	1.21	PRKRIR	protein-kinase, interferon-inducible double stranded RNA dependent inh	Upregulated

A_33_P321	0.01209	0.00409	1.21	BMPR1A	bone morphogenetic protein receptor, type IA	Upregulated
A_22_P000	0.012113	0.0041	1.21	LOC1019276	uncharacterized LOC101927637	Upregulated
A_22_P000	0.013468	0.00469	1.21	lnc-ZWINT-5	lnc-ZWINT-5:1	Upregulated
A_22_P000	0.035165	0.0157	1.21	lnc-AC00603	lnc-AC006035.2-3:1	Upregulated
A_23_P121	0.044655	0.0209	1.21	TRAPPC13	trafficking protein particle complex 13	Upregulated
A_22_P000	0.060624	0.0302	1.21	lnc-GATAD1-	lnc-GATAD1-2:1	Upregulated
A_22_P000	0.073261	0.0379	1.21	KCNJ10	potassium channel, inwardly rectifying subfamily J, member 10	Upregulated
A_33_P329	0.001808	0.000251	1.2	DPP9	dipeptidyl-peptidase 9	Upregulated
A_23_P103	0.001913	0.000277	1.2	MFNG	MFNG O-fucosylpeptide 3-beta-N-acetylglucosaminyltransferase	Upregulated
A_24_P337	0.002043	0.000313	1.2	RABGEF1	RAB guanine nucleotide exchange factor (GEF) 1	Upregulated
A_23_P120	0.002117	0.000333	1.2	MTHFD2	methylenetetrahydrofolate dehydrogenase (NADP+ dependent) 2, meth	Upregulated
A_23_P200	0.002128	0.000336	1.2	NEXN	nexilin (F actin binding protein)	Upregulated
A_24_P105	0.002124	0.000336	1.2	PRKAB2	protein kinase, AMP-activated, beta 2 non-catalytic subunit	Upregulated
A_23_P503	0.002227	0.000361	1.2	EDARADD	EDAR-associated death domain	Upregulated
A_19_P003	0.002353	0.000391	1.2	FAM200B	family with sequence similarity 200, member B	Upregulated
A_24_P136	0.002423	0.000409	1.2	TOPORS-AS	TOPORS antisense RNA 1	Upregulated
A_24_P356	0.002452	0.000415	1.2	GABARAPL2	GABA(A) receptor-associated protein-like 2	Upregulated
A_23_P470	0.002499	0.000429	1.2	WDR37	WD repeat domain 37	Upregulated
A_23_P372	0.002596	0.000455	1.2	GAPVD1	GTPase activating protein and VPS9 domains 1	Upregulated
A_23_P204	0.00263	0.000464	1.2	H2AFJ	H2A histone family, member J	Upregulated
A_33_P336	0.002907	0.000541	1.2	NAP1L4	nucleosome assembly protein 1-like 4	Upregulated
A_33_P324	0.00604	0.0016	1.2	SPDL1	spindle apparatus coiled-coil protein 1	Upregulated
A_23_P468	0.006588	0.00181	1.2	OBFC1	oligonucleotide/oligosaccharide-binding fold containing 1	Upregulated
A_32_P206	0.007166	0.00204	1.2	ZNF322	zinc finger protein 322	Upregulated
A_22_P000	0.011991	0.00405	1.2	lnc-C10orf11-	lnc-C10orf11-3:1	Upregulated
A_32_P191	0.011991	0.00405	1.2	LOC644662	uncharacterized LOC644662	Upregulated
A_23_P328	0.013274	0.00461	1.2	LCOR	ligand dependent nuclear receptor corepressor	Upregulated
A_23_P140	0.014406	0.00513	1.2	TMEM8A	transmembrane protein 8A	Upregulated
A_23_P565	0.017727	0.0067	1.2	C1D	C1D nuclear receptor corepressor	Upregulated
A_23_P112	0.018945	0.00727	1.2	NUDT2	nudix (nucleoside diphosphate linked moiety X)-type motif 2	Upregulated
A_24_P965	0.033475	0.0147	1.2	EVI5	ecotropic viral integration site 5	Upregulated
A_22_P000	0.035267	0.0158	1.2	LOC1005070	uncharacterized LOC100507071	Upregulated
A_22_P000	0.03885	0.0177	1.2	LOC1001290	uncharacterized LOC100129046	Upregulated
A_33_P335	0.063131	0.0317	1.2	GAS6-AS1	GAS6 antisense RNA 1	Upregulated
A_33_P330	0.08413	0.0448	1.2	MTRR	5-methyltetrahydrofolate-homocysteine methyltransferase reductase	Upregulated
A_33_P322	0.001635	0.00021	1.19	CDK11A	cyclin-dependent kinase 11A	Upregulated
A_23_P557	0.001667	0.000217	1.19	RELB	v-rel avian reticuloendotheliosis viral oncogene homolog B	Upregulated

A_23_P368	0.001684	0.000221	1.19	EME1	essential meiotic structure-specific endonuclease 1	Upregulated
A_24_P565	0.001709	0.000228	1.19	ALG1	ALG1, chitobiosyldiphosphodolichol beta-mannosyltransferase	Upregulated
A_23_P646	0.001709	0.000229	1.19	ZCCHC8	zinc finger, CCHC domain containing 8	Upregulated
A_23_P519	0.00174	0.000234	1.19	PFDN2	prefoldin subunit 2	Upregulated
A_23_P257	0.001805	0.00025	1.19	USP16	ubiquitin specific peptidase 16	Upregulated
A_23_P431	0.001981	0.000295	1.19	YIPF6	Yip1 domain family, member 6	Upregulated
A_23_P171	0.002012	0.000304	1.19	GPN1	GPN-loop GTPase 1	Upregulated
A_22_P000	0.002037	0.000311	1.19	lnc-RUNDC3A	lnc-RUNDC3A-1:1	Upregulated
A_22_P000	0.002722	0.000489	1.19	lnc-UBE2Z-1	lnc-UBE2Z-1:1	Upregulated
A_23_P111	0.00274	0.000494	1.19	YIPF3	Yip1 domain family, member 3	Upregulated
A_23_P425	0.00284	0.000522	1.19	TRIO	trio Rho guanine nucleotide exchange factor	Upregulated
A_21_P001	0.003394	0.000687	1.19	FRG1	FSHD region gene 1	Upregulated
A_22_P000	0.004088	0.0009	1.19	lnc-AQP8-1	lnc-AQP8-1:1	Upregulated
A_32_P558	0.004502	0.00104	1.19	SKA2	spindle and kinetochore associated complex subunit 2	Upregulated
A_33_P335	0.005252	0.00131	1.19	MFSD12	major facilitator superfamily domain containing 12	Upregulated
A_21_P001	0.005296	0.00132	1.19	XLOC_l2_009613		Upregulated
A_33_P338	0.0066	0.00182	1.19	WHSC1	Wolf-Hirschhorn syndrome candidate 1	Upregulated
A_23_P510	0.0111	0.00365	1.19	SPC25	SPC25, NDC80 kinetochore complex component	Upregulated
A_21_P001	0.014099	0.00498	1.19	lnc-RGL4-4	lnc-RGL4-4:4	Upregulated
A_22_P000	0.025957	0.0108	1.19	LINCR-0002	uncharacterized LincR-0002	Upregulated
A_22_P000	0.025957	0.0108	1.19	lnc-TSPAN32	lnc-TSPAN32-1:1	Upregulated
A_21_P000	0.052165	0.0252	1.19	LINC01304	long intergenic non-protein coding RNA 1304	Upregulated
A_33_P337	0.001643	0.000212	1.18	SAT1	spermidine/spermine N1-acetyltransferase 1	Upregulated
A_23_P171	0.001651	0.000214	1.18	IGBP1	immunoglobulin (CD79A) binding protein 1	Upregulated
A_23_P717	0.001695	0.000224	1.18	CKS2	CDC28 protein kinase regulatory subunit 2	Upregulated
A_33_P331	0.00176	0.000239	1.18	FAM46C	family with sequence similarity 46, member C	Upregulated
A_19_P008	0.001837	0.000259	1.18	lnc-SAA2-2	lnc-SAA2-2:1	Upregulated
A_33_P321	0.001936	0.000282	1.18	ETV6	ets variant 6	Upregulated
A_23_P695	0.001991	0.000298	1.18	CCNI	cyclin I	Upregulated
A_24_P743	0.002248	0.000368	1.18	ZNF493	zinc finger protein 493	Upregulated
A_22_P000	0.002314	0.000382	1.18	lnc-GATAD2B	lnc-GATAD2B-1:2	Upregulated
A_24_P277	0.002361	0.000394	1.18	HIST1H4G	histone cluster 1, H4g	Upregulated
A_32_P584	0.002468	0.00042	1.18	PHF13	PHD finger protein 13	Upregulated
A_33_P327	0.002572	0.00045	1.18	ZNF525	zinc finger protein 525	Upregulated
A_33_P336	0.002825	0.000517	1.18	TAF8	TAF8 RNA polymerase II, TATA box binding protein (TBP)-associated fa	Upregulated
A_23_P897	0.003387	0.000683	1.18	PHLPP1	PH domain and leucine rich repeat protein phosphatase 1	Upregulated
A_23_P353	0.003468	0.000707	1.18	LARP1B	La ribonucleoprotein domain family, member 1B	Upregulated

A_19_P003	0.003611	0.000753	1.18	LOC220729	succinate dehydrogenase complex, subunit A, flavoprotein (Fp) pseudo	Upregulated
A_33_P328	0.003728	0.000789	1.18	ZNF775	zinc finger protein 775	Upregulated
A_23_P171	0.003985	0.000868	1.18	WDR44	WD repeat domain 44	Upregulated
A_22_P000	0.00422	0.000947	1.18	lnc-SLC24A5-	lnc-SLC24A5-3:1	Upregulated
A_33_P341	0.005195	0.00128	1.18	ACER2	alkaline ceramidase 2	Upregulated
A_24_P148	0.005869	0.00153	1.18	UBE2E3	ubiquitin-conjugating enzyme E2E 3	Upregulated
A_33_P321	0.008048	0.00238	1.18	LZTS1	leucine zipper, putative tumor suppressor 1	Upregulated
A_22_P000	0.009619	0.00303	1.18	CKMT2-AS1	CKMT2 antisense RNA 1	Upregulated
A_33_P339	0.010598	0.00344	1.18	FAM73A	family with sequence similarity 73, member A	Upregulated
A_21_P000	0.011718	0.00393	1.18	LOC1019292	uncharacterized LOC101929295	Upregulated
A_24_P287	0.012163	0.00412	1.18	TOLLIP	toll interacting protein	Upregulated
A_23_P145	0.02598	0.0108	1.18	SUGCT	succinyl-CoA:glutarate-CoA transferase	Upregulated
A_33_P335	0.031056	0.0135	1.18	DDC	dopa decarboxylase (aromatic L-amino acid decarboxylase)	Upregulated
A_33_P330	0.036625	0.0165	1.18	LOC1001300	uncharacterized LOC100130078	Upregulated
A_33_P335	0.049558	0.0238	1.18	ETV1	ets variant 1	Upregulated
A_21_P000	0.050136	0.0241	1.18	lnc-USP12-2	lnc-USP12-2:1	Upregulated
A_19_P003	0.053537	0.0261	1.18	LINC00870	long intergenic non-protein coding RNA 870	Upregulated
A_22_P000	0.0017	0.000226	1.17	lnc-CEP152-1	lnc-CEP152-1:1	Upregulated
A_33_P333	0.001815	0.000253	1.17	GINM1	glycoprotein integral membrane 1	Upregulated
A_22_P000	0.001863	0.000265	1.17	LOC1009964	uncharacterized LOC100996425	Upregulated
A_24_P407	0.001922	0.000279	1.17	GRB2	growth factor receptor-bound protein 2	Upregulated
A_23_P202	0.001958	0.000289	1.17	PCGF5	polycomb group ring finger 5	Upregulated
A_22_P000	0.002022	0.000306	1.17	LOC1019277	uncharacterized LOC101927730	Upregulated
A_22_P000	0.002034	0.00031	1.17	lnc-AC11494	lnc-AC114947.1.1-1:2	Upregulated
A_22_P000	0.002112	0.00033	1.17	lnc-ACTR6-1	lnc-ACTR6-1:1	Upregulated
A_24_P912	0.002112	0.00033	1.17	MGC50722	uncharacterized MGC50722	Upregulated
A_32_P457	0.00218	0.000349	1.17	PGAM1	phosphoglycerate mutase 1 (brain)	Upregulated
A_23_P698	0.002459	0.000417	1.17	DHX15	DEAH (Asp-Glu-Ala-His) box helicase 15	Upregulated
A_23_P211	0.00247	0.00042	1.17	DGCR8	DGCR8 microprocessor complex subunit	Upregulated
A_33_P334	0.002524	0.000436	1.17	AP2S1	adaptor-related protein complex 2, sigma 1 subunit	Upregulated
A_24_P286	0.002572	0.000449	1.17	AHCTF1	AT hook containing transcription factor 1	Upregulated
A_23_P158	0.002661	0.000472	1.17	ESRRA	estrogen-related receptor alpha	Upregulated
A_33_P335	0.002874	0.000531	1.17	AKIRIN1	akirin 1	Upregulated
A_33_P327	0.003113	0.000603	1.17	FKBP8	FK506 binding protein 8, 38kDa	Upregulated
A_22_P000	0.003389	0.000685	1.17	lnc-FAM168A	lnc-FAM168A-1:1	Upregulated
A_24_P158	0.004647	0.00109	1.17	IRAK4	interleukin-1 receptor-associated kinase 4	Upregulated
A_23_P212	0.006202	0.00166	1.17	PTPN23	protein tyrosine phosphatase, non-receptor type 23	Upregulated

A_22_P000	0.006764	0.00188	1.17	lnc-B4GALNT	lnc-B4GALNT3-1:1	Upregulated
A_23_P353	0.010026	0.0032	1.17	IGFBP7	insulin-like growth factor binding protein 7	Upregulated
A_22_P000	0.010209	0.00329	1.17	lnc-TMC7-1	lnc-TMC7-1:1	Upregulated
A_33_P325	0.011464	0.00382	1.17	PPP1R12B	protein phosphatase 1, regulatory subunit 12B	Upregulated
A_21_P001	0.013974	0.00492	1.17	LOC1019302	uncharacterized LOC101930282	Upregulated
A_24_P139	0.020379	0.00795	1.17	USP21	ubiquitin specific peptidase 21	Upregulated
A_23_P367	0.023201	0.00934	1.17	EPOR	erythropoietin receptor	Upregulated
A_23_P516	0.030538	0.0132	1.17	PLK3	polo-like kinase 3	Upregulated
A_23_P366	0.036454	0.0164	1.17	TDGF1	teratocarcinoma-derived growth factor 1	Upregulated
A_21_P001	0.044618	0.0209	1.17	XLOC_l2_011309		Upregulated
A_33_P330	0.06701	0.0341	1.17	GGA1	golgi-associated, gamma adaptin ear containing, ARF binding protein 1	Upregulated
A_33_P353	0.001732	0.000233	1.16	DARS	aspartyl-tRNA synthetase	Upregulated
A_24_P100	0.001837	0.000259	1.16	SH3RF1	SH3 domain containing ring finger 1	Upregulated
A_23_P404	0.001953	0.000287	1.16	KIAA1147	KIAA1147	Upregulated
A_33_P341	0.002034	0.00031	1.16	FAM96A	family with sequence similarity 96, member A	Upregulated
A_33_P334	0.002219	0.000358	1.16	TRAF3IP2	TRAF3 interacting protein 2	Upregulated
A_23_P600	0.002369	0.000396	1.16	ANGPT2	angiopoietin 2	Upregulated
A_33_P342	0.002492	0.000427	1.16	IKZF5	IKAROS family zinc finger 5 (Pegasus)	Upregulated
A_32_P221	0.003483	0.000712	1.16	MGC70870	C-terminal binding protein 2 pseudogene	Upregulated
A_32_P168	0.003532	0.000727	1.16	ANAPC13	anaphase promoting complex subunit 13	Upregulated
A_33_P338	0.006489	0.00177	1.16	FBXO5	F-box protein 5	Upregulated
A_33_P334	0.010643	0.00346	1.16	RHOBTB1	Rho-related BTB domain containing 1	Upregulated
A_23_P333	0.011562	0.00387	1.16	ZNF547	zinc finger protein 547	Upregulated
A_22_P000	0.012254	0.00416	1.16	lnc-OTUD3-1	lnc-OTUD3-1:1	Upregulated
A_22_P000	0.013997	0.00493	1.16	RASAL2	RAS protein activator like 2	Upregulated
A_23_P119	0.027185	0.0114	1.16	FXYD7	FXYD domain containing ion transport regulator 7	Upregulated
A_33_P337	0.02899	0.0124	1.16	OPA3	optic atrophy 3 (autosomal recessive, with chorea and spastic paraplegi	Upregulated
A_33_P330	0.02899	0.0124	1.16	SLC16A10	solute carrier family 16 (aromatic amino acid transporter), member 10	Upregulated
A_22_P000	0.032792	0.0144	1.16	lnc-AC09018(	lnc-AC090186.1-3:1	Upregulated
A_21_P000	0.039618	0.0182	1.16	lnc-UBLCP1-8	lnc-UBLCP1-8:2	Upregulated
A_21_P000	0.052196	0.0253	1.16	lnc-PRR16-1	lnc-PRR16-1:2	Upregulated
A_23_P352	0.001751	0.000236	1.15	POLR2C	polymerase (RNA) II (DNA directed) polypeptide C, 33kDa	Upregulated
A_24_P216	0.001808	0.000251	1.15	LSM10	LSM10, U7 small nuclear RNA associated	Upregulated
A_24_P920	0.001863	0.000265	1.15	ASB7	ankyrin repeat and SOCS box containing 7	Upregulated
A_33_P328	0.001962	0.000291	1.15	KAT6A	K(lysine) acetyltransferase 6A	Upregulated
A_24_P418	0.002027	0.000308	1.15	RPS17	ribosomal protein S17	Upregulated
A_24_P260	0.002124	0.000335	1.15	LRPPRC	leucine-rich pentatricopeptide repeat containing	Upregulated

A_23_P102	0.002154	0.000342	1.15	CCT4	chaperonin containing TCP1, subunit 4 (delta)	Upregulated
A_24_P854	0.002271	0.000372	1.15	ARIH1	ariadne RBR E3 ubiquitin protein ligase 1	Upregulated
A_24_P388	0.00228	0.000374	1.15	SRP19	signal recognition particle 19kDa	Upregulated
A_23_P133	0.002286	0.000376	1.15	PJA2	praja ring finger 2, E3 ubiquitin protein ligase	Upregulated
A_23_P784	0.0025	0.00043	1.15	ELP2	elongator acetyltransferase complex subunit 2	Upregulated
A_33_P327	0.002532	0.000438	1.15	SLC25A16	solute carrier family 25 (mitochondrial carrier), member 16	Upregulated
A_32_P226	0.00301	0.000571	1.15	FOXP4-AS1	FOXP4 antisense RNA 1	Upregulated
A_23_P255	0.003658	0.000766	1.15	DUS1L	dihydrouridine synthase 1-like (S. cerevisiae)	Upregulated
A_23_P128	0.004595	0.00107	1.15	HELLS	helicase, lymphoid-specific	Upregulated
A_33_P328	0.004613	0.00108	1.15	IVNS1ABP	influenza virus NS1A binding protein	Upregulated
A_33_P336	0.005041	0.00123	1.15	RBBP9	retinoblastoma binding protein 9	Upregulated
A_23_P310	0.005219	0.00129	1.15	PRSS3P2	protease, serine, 3 pseudogene 2	Upregulated
A_21_P000	0.005539	0.00141	1.15	lnc-AC06925	lnc-AC069257.9.1-5:1	Upregulated
A_23_P266	0.005854	0.00153	1.15	TMEM186	transmembrane protein 186	Upregulated
A_24_P942	0.006994	0.00197	1.15	PGP	phosphoglycolate phosphatase	Upregulated
A_23_P208	0.007101	0.00201	1.15	WDR34	WD repeat domain 34	Upregulated
A_33_P333	0.009013	0.00278	1.15	PPCS	phosphopantothenoylcysteine synthetase	Upregulated
A_32_P434	0.010871	0.00355	1.15	ZNF182	zinc finger protein 182	Upregulated
A_23_P214	0.016636	0.00619	1.15	AIF1	allograft inflammatory factor 1	Upregulated
A_19_P003	0.017213	0.00647	1.15	LOC1019271	uncharacterized LOC101927151	Upregulated
A_22_P000	0.025457	0.0105	1.15	lnc-PAX8-1	lnc-PAX8-1:2	Upregulated
A_23_P210	0.025963	0.0108	1.15	SIRPA	signal-regulatory protein alpha	Upregulated
A_22_P000	0.035067	0.0156	1.15	lnc-STOM-1	lnc-STOM-1:1	Upregulated
A_33_P332	0.041447	0.0192	1.15	ZDHHC3	zinc finger, DHHC-type containing 3	Upregulated
A_22_P000	0.059444	0.0295	1.15	lnc-RHD-1	lnc-RHD-1:1	Upregulated
A_22_P000	0.001782	0.000244	1.14	lnc-EFR3B-4	lnc-EFR3B-4:1	Upregulated
A_32_P384	0.001823	0.000255	1.14	SNHG8	small nucleolar RNA host gene 8 (non-protein coding)	Upregulated
A_24_P899	0.001884	0.000269	1.14	SURF4	surfeit 4	Upregulated
A_22_P000	0.001893	0.000272	1.14	LOC1027233	uncharacterized LOC102723366	Upregulated
A_24_P874	0.001892	0.000272	1.14	TVP23C	trans-golgi network vesicle protein 23 homolog C (S. cerevisiae)	Upregulated
A_23_P618	0.001948	0.000285	1.14	RAB24	RAB24, member RAS oncogene family	Upregulated
A_33_P323	0.001958	0.000289	1.14	DENND4B	DENN/MADD domain containing 4B	Upregulated
A_23_P509	0.002053	0.000315	1.14	ITGAV	integrin, alpha V	Upregulated
A_23_P204	0.002124	0.000335	1.14	TDG	thymine-DNA glycosylase	Upregulated
A_23_P212	0.002241	0.000365	1.14	ELP6	elongator acetyltransferase complex subunit 6	Upregulated
A_32_P150	0.002374	0.000397	1.14	LOC407835	mitogen-activated protein kinase kinase 2 pseudogene	Upregulated
A_23_P487	0.002527	0.000437	1.14	UCK2	uridine-cytidine kinase 2	Upregulated

A_33_P322	0.002597	0.000456	1.14	PSIP1	PC4 and SFRS1 interacting protein 1	Upregulated
A_23_P364	0.002655	0.000471	1.14	SELK	selenoprotein K	Upregulated
A_33_P329	0.002714	0.000487	1.14	PPM1B	protein phosphatase, Mg2+/Mn2+ dependent, 1B	Upregulated
A_24_P155	0.002932	0.000547	1.14	ASB7	ankyrin repeat and SOCS box containing 7	Upregulated
A_21_P001	0.004221	0.000948	1.14	LOC388692	uncharacterized LOC388692	Upregulated
A_23_P144	0.004303	0.000974	1.14	ANKRD32	ankyrin repeat domain 32	Upregulated
A_23_P375	0.005628	0.00145	1.14	AAGAB	alpha- and gamma-adaptin binding protein	Upregulated
A_23_P502	0.00702	0.00198	1.14	CBFA2T2	core-binding factor, runt domain, alpha subunit 2; translocated to, 2	Upregulated
A_23_P218	0.009165	0.00284	1.14	TPCN1	two pore segment channel 1	Upregulated
A_23_P160	0.010976	0.00359	1.14	MYOG	myogenin (myogenic factor 4)	Upregulated
A_23_P457	0.021337	0.00842	1.14	ORC1	origin recognition complex, subunit 1	Upregulated
A_33_P337	0.026634	0.0111	1.14	AGAP11	ankyrin repeat and GTPase domain Arf GTPase activating protein 11	Upregulated
A_21_P000	0.031962	0.0139	1.14	lnc-NFE2L3-2	lnc-NFE2L3-2:1	Upregulated
A_22_P000	0.072699	0.0375	1.14	lnc-HAS2-1	lnc-HAS2-1:1	Upregulated
A_23_P833	0.00179	0.000246	1.13	MRPL18	mitochondrial ribosomal protein L18	Upregulated
A_23_P157	0.001861	0.000264	1.13	INTS10	integrator complex subunit 10	Upregulated
A_21_P000	0.001938	0.000283	1.13	SNORD46	small nucleolar RNA, C/D box 46	Upregulated
A_33_P341	0.002114	0.000331	1.13	NUDT21	nudix (nucleoside diphosphate linked moiety X)-type motif 21	Upregulated
A_33_P324	0.00234	0.000388	1.13	XIAP	X-linked inhibitor of apoptosis, E3 ubiquitin protein ligase	Upregulated
A_24_P406	0.002416	0.000407	1.13	SLC43A3	solute carrier family 43, member 3	Upregulated
A_33_P332	0.002469	0.00042	1.13	RSPRY1	ring finger and SPRY domain containing 1	Upregulated
A_23_P435	0.002483	0.000425	1.13	WSB1	WD repeat and SOCS box containing 1	Upregulated
A_23_P356	0.002644	0.000468	1.13	MAPK8	mitogen-activated protein kinase 8	Upregulated
A_33_P330	0.002785	0.000506	1.13	EXOSC4	exosome component 4	Upregulated
A_21_P000	0.003149	0.000612	1.13	lnc-COPS4-1	lnc-COPS4-1:2	Upregulated
A_21_P001	0.003465	0.000707	1.13	ANKRD20A9I	ankyrin repeat domain 20 family, member A9, pseudogene	Upregulated
A_23_P614	0.003721	0.000787	1.13	ETFDH	electron-transferring-flavoprotein dehydrogenase	Upregulated
A_23_P743	0.004269	0.000964	1.13	TADA1	transcriptional adaptor 1	Upregulated
A_23_P166	0.004313	0.000978	1.13	TRMT10C	tRNA methyltransferase 10 homolog C (S. cerevisiae)	Upregulated
A_33_P331	0.004725	0.00112	1.13	ZNF215	zinc finger protein 215	Upregulated
A_21_P000	0.005231	0.0013	1.13	lnc-C5orf43-2	lnc-C5orf43-2:1	Upregulated
A_33_P326	0.005399	0.00136	1.13	MAZ	MYC-associated zinc finger protein (purine-binding transcription factor)	Upregulated
A_23_P346	0.006116	0.00163	1.13	MCU	mitochondrial calcium uniporter	Upregulated
A_33_P324	0.008137	0.00242	1.13	GOLGA6L4	golgin A6 family-like 4	Upregulated
A_23_P163	0.008331	0.0025	1.13	ECI1	enoyl-CoA delta isomerase 1	Upregulated
A_23_P376	0.008796	0.00269	1.13	BEND7	BEN domain containing 7	Upregulated
A_24_P372	0.008796	0.00269	1.13	PEX5	peroxisomal biogenesis factor 5	Upregulated

A_33_P338	0.009543	0.003	1.13	KIF3A	kinesin family member 3A	Upregulated
A_22_P000	0.020606	0.00806	1.13	LOC1019273	uncharacterized LOC101927326	Upregulated
A_33_P336	0.022884	0.00918	1.13	GYPA	glycophorin A (MNS blood group)	Upregulated
A_33_P361	0.026664	0.0112	1.13	POLR1A	polymerase (RNA) I polypeptide A, 194kDa	Upregulated
A_33_P324	0.052806	0.0256	1.13	ZNF814	zinc finger protein 814	Upregulated
A_22_P000	0.077329	0.0404	1.13	UBXN10-AS1	UBXN10 antisense RNA 1	Upregulated
A_21_P000	0.001838	0.000259	1.12	LINC01490	long intergenic non-protein coding RNA 1490	Upregulated
A_21_P000	0.001838	0.000259	1.12	lnc-ATP6AP2	lnc-ATP6AP2-5:1	Upregulated
A_33_P341	0.001949	0.000286	1.12	ATMIN	ATM interactor	Upregulated
A_32_P644	0.002027	0.000308	1.12	SDE2	SDE2 telomere maintenance homolog (S. pombe)	Upregulated
A_23_P942	0.00249	0.000426	1.12	LY96	lymphocyte antigen 96	Upregulated
A_23_P400	0.002572	0.00045	1.12	ETV3	ets variant 3	Upregulated
A_33_P335	0.002631	0.000465	1.12	TRIM78P	tripartite motif containing 78, pseudogene	Upregulated
A_33_P321	0.002889	0.000536	1.12	IBA57	IBA57, iron-sulfur cluster assembly homolog (S. cerevisiae)	Upregulated
A_21_P000	0.003491	0.000714	1.12	LINC01012	long intergenic non-protein coding RNA 1012	Upregulated
A_32_P946	0.004113	0.000908	1.12	LRRC37B	leucine rich repeat containing 37B	Upregulated
A_23_P278	0.004813	0.00115	1.12	ZNF607	zinc finger protein 607	Upregulated
A_33_P384	0.005278	0.00132	1.12	IKZF2	IKAROS family zinc finger 2 (Helios)	Upregulated
A_33_P332	0.005389	0.00136	1.12	THRB-AS1	THRB antisense RNA 1	Upregulated
A_23_P424	0.006917	0.00194	1.12	RHOV	ras homolog family member V	Upregulated
A_21_P000	0.007685	0.00224	1.12	LOC1019278	uncharacterized LOC101927815	Upregulated
A_21_P000	0.009226	0.00287	1.12	VTRNA1-3	vault RNA 1-3	Upregulated
A_24_P400	0.010993	0.0036	1.12	SLC25A44	solute carrier family 25, member 44	Upregulated
A_33_P340	0.01156	0.00386	1.12	TMEM38B	transmembrane protein 38B	Upregulated
A_22_P000	0.024466	0.01	1.12	MCM3AP	minichromosome maintenance complex component 3 associated proteir	Upregulated
A_32_P150	0.024864	0.0102	1.12	LOC1019283	uncharacterized LOC101928370	Upregulated
A_23_P171	0.025552	0.0106	1.12	CCNB3	cyclin B3	Upregulated
A_22_P000	0.031752	0.0138	1.12	LOC1019274	uncharacterized LOC101927472	Upregulated
A_23_P320	0.045156	0.0213	1.12	DEFB125	defensin, beta 125	Upregulated
A_21_P000	0.045156	0.0213	1.12	lnc-SBF2-2	lnc-SBF2-2:1	Upregulated
A_21_P000	0.048094	0.0229	1.12	LOC1027252	uncharacterized LOC102725283	Upregulated
A_24_P343	0.048094	0.0229	1.12	NTRK2	neurotrophic tyrosine kinase, receptor, type 2	Upregulated
A_23_P873	0.054634	0.0267	1.12	COMMD9	COMM domain containing 9	Upregulated
A_21_P000	0.054634	0.0267	1.12	LOC1005057	uncharacterized LOC100505715	Upregulated
A_22_P000	0.054634	0.0267	1.12	LOC1005060	uncharacterized LOC100506085	Upregulated
A_21_P001	0.066933	0.034	1.12	LOC1002895	uncharacterized LOC100289580	Upregulated
A_21_P000	0.076004	0.0396	1.12	PABPC1L2B-	PABPC1L2B antisense RNA 1 (head to head)	Upregulated

A_23_P408	0.001865	0.000266	1.11	CCT6B	chaperonin containing TCP1, subunit 6B (zeta 2)	Upregulated
A_21_P001	0.001991	0.000298	1.11	LOC1027254	uncharacterized LOC102725415	Upregulated
A_24_P766	0.002117	0.000333	1.11	CSNK2A1	casein kinase 2, alpha 1 polypeptide	Upregulated
A_23_P306	0.002132	0.000338	1.11	PPTC7	PTC7 protein phosphatase homolog (S. cerevisiae)	Upregulated
A_23_P708	0.002154	0.000342	1.11	BPGM	2,3-bisphosphoglycerate mutase	Upregulated
A_24_P417	0.002361	0.000394	1.11	TBL1X	transducin (beta)-like 1X-linked	Upregulated
A_24_P722	0.002483	0.000425	1.11	DHTKD1	dehydrogenase E1 and transketolase domain containing 1	Upregulated
A_23_P120	0.002677	0.000476	1.11	TRIT1	tRNA isopentenyltransferase 1	Upregulated
A_21_P001	0.003393	0.000687	1.11	SYNGR2	synaptogyrin 2	Upregulated
A_33_P339	0.003567	0.00074	1.11	ZC3HAV1	zinc finger CCCH-type, antiviral 1	Upregulated
A_32_P213	0.004341	0.000989	1.11	C20orf202	chromosome 20 open reading frame 202	Upregulated
A_24_P207	0.004341	0.000989	1.11	LCE2B	late cornified envelope 2B	Upregulated
A_19_P003	0.004341	0.000989	1.11	TBXAS1	thromboxane A synthase 1 (platelet)	Upregulated
A_24_P154	0.004843	0.00116	1.11	ZBTB49	zinc finger and BTB domain containing 49	Upregulated
A_23_P152	0.006493	0.00177	1.11	EARS2	glutamyl-tRNA synthetase 2, mitochondrial	Upregulated
A_23_P334	0.006885	0.00192	1.11	SENP8	SUMO/sentrin specific peptidase family member 8	Upregulated
A_33_P321	0.007518	0.00217	1.11	CCZ1	CCZ1 vacuolar protein trafficking and biogenesis associated homolog (S	Upregulated
A_33_P332	0.008143	0.00242	1.11	GOLGA2P6	golgin A2 pseudogene 6	Upregulated
A_33_P334	0.008884	0.00273	1.11	UBAP2	ubiquitin associated protein 2	Upregulated
A_23_P131	0.011812	0.00397	1.11	THUMPD2	THUMP domain containing 2	Upregulated
A_21_P001	0.015092	0.00546	1.11	SEPT7P2	septin 7 pseudogene 2	Upregulated
A_23_P561	0.023072	0.00927	1.11	SMG9	SMG9 nonsense mediated mRNA decay factor	Upregulated
A_23_P316	0.033316	0.0146	1.11	HOXB3	homeobox B3	Upregulated
A_21_P000	0.03335	0.0147	1.11	lnc-RMI1-3	lnc-RMI1-3:1	Upregulated
A_33_P337	0.073354	0.0379	1.11	FAM229B	family with sequence similarity 229, member B	Upregulated
A_24_P258	0.001949	0.000286	1.1	MASTL	microtubule associated serine/threonine kinase-like	Upregulated
A_24_P235	0.00198	0.000295	1.1	VDAC3	voltage-dependent anion channel 3	Upregulated
A_23_P166	0.001986	0.000297	1.1	ABCG1	ATP-binding cassette, sub-family G (WHITE), member 1	Upregulated
A_23_P631	0.002124	0.000335	1.1	DDX20	DEAD (Asp-Glu-Ala-Asp) box polypeptide 20	Upregulated
A_33_P334	0.002254	0.000369	1.1	RRNAD1	ribosomal RNA adenine dimethylase domain containing 1	Upregulated
A_33_P338	0.002284	0.000375	1.1	ANKRD40	ankyrin repeat domain 40	Upregulated
A_33_P339	0.00233	0.000385	1.1	CCDC6	coiled-coil domain containing 6	Upregulated
A_24_P233	0.002726	0.00049	1.1	SDHC	succinate dehydrogenase complex, subunit C, integral membrane prote	Upregulated
A_23_P317	0.002881	0.000533	1.1	C8orf76	chromosome 8 open reading frame 76	Upregulated
A_24_P368	0.003019	0.000574	1.1	WDR26	WD repeat domain 26	Upregulated
A_23_P735	0.003184	0.000625	1.1	GRIPAP1	GRIP1 associated protein 1	Upregulated
A_24_P294	0.003572	0.000742	1.1	PIK3R1	phosphoinositide-3-kinase, regulatory subunit 1 (alpha)	Upregulated

A_19_P003	0.003689	0.000776	1.1	LOC1019276	uncharacterized LOC101927686	Upregulated
A_23_P169	0.004227	0.00095	1.1	SDC1	syndecan 1	Upregulated
A_23_P151	0.004447	0.00102	1.1	IFNG	interferon, gamma	Upregulated
A_21_P001	0.005796	0.00151	1.1	LOC1019282	uncharacterized LOC101928228	Upregulated
A_23_P361	0.005854	0.00153	1.1	MS4A6A	membrane-spanning 4-domains, subfamily A, member 6A	Upregulated
A_21_P000	0.006058	0.0016	1.1	lnc-RABGAP1	lnc-RABGAP1-1:1	Upregulated
A_33_P325	0.006203	0.00166	1.1	TCF7	transcription factor 7 (T-cell specific, HMG-box)	Upregulated
A_23_P209	0.009437	0.00295	1.1	ATG12	autophagy related 12	Upregulated
A_33_P342	0.014864	0.00536	1.1	OR4D9	olfactory receptor, family 4, subfamily D, member 9	Upregulated
A_23_P362	0.014864	0.00536	1.1	OR52B2	olfactory receptor, family 52, subfamily B, member 2	Upregulated
A_23_P205	0.01536	0.00558	1.1	CDC37L1	cell division cycle 37-like 1	Upregulated
A_23_P216	0.016641	0.0062	1.1	CDC14B	cell division cycle 14B	Upregulated
A_23_P502	0.022057	0.00877	1.1	DGKH	diacylglycerol kinase, eta	Upregulated
A_23_P139	0.022275	0.00887	1.1	OASL	2'-5'-oligoadenylate synthetase-like	Upregulated
A_33_P338	0.022415	0.00894	1.1	TCP11X2	t-complex 11 family, X-linked 2	Upregulated
A_23_P507	0.029562	0.0127	1.1	ZNF181	zinc finger protein 181	Upregulated
A_33_P327	0.030088	0.013	1.1	UCN2	urocortin 2	Upregulated
A_21_P000	0.036707	0.0165	1.1	lnc-ZNF296-1	lnc-ZNF296-1:1	Upregulated
A_33_P335	0.042467	0.0197	1.1	PTGFR	prostaglandin F receptor (FP)	Upregulated
A_22_P000	0.055792	0.0274	1.1	LINC00642	long intergenic non-protein coding RNA 642	Upregulated
A_21_P000	0.056334	0.0277	1.1	lnc-LY86-1	lnc-LY86-1:1	Upregulated
A_33_P331	0.087386	0.0471	1.1	XLOC_l2_005197		Upregulated
A_23_P165	0.001925	0.00028	1.09	PSMD14	proteasome (prosome, macropain) 26S subunit, non-ATPase, 14	Upregulated
A_23_P478	0.001962	0.000291	1.09	DDX55	DEAD (Asp-Glu-Ala-Asp) box polypeptide 55	Upregulated
A_24_P377	0.002112	0.00033	1.09	HSPA4	heat shock 70kDa protein 4	Upregulated
A_23_P252	0.002124	0.000335	1.09	YBX3	Y box binding protein 3	Upregulated
A_23_P569	0.002174	0.000348	1.09	HSPE1	heat shock 10kDa protein 1	Upregulated
A_23_P154	0.002464	0.000419	1.09	NOP58	NOP58 ribonucleoprotein	Upregulated
A_23_P218	0.002471	0.000421	1.09	EIF5B	eukaryotic translation initiation factor 5B	Upregulated
A_33_P336	0.002476	0.000422	1.09	LY9	lymphocyte antigen 9	Upregulated
A_24_P250	0.00301	0.000571	1.09	CBWD5	COBW domain containing 5	Upregulated
A_33_P321	0.003056	0.000584	1.09	ZNF808	zinc finger protein 808	Upregulated
A_22_P000	0.003139	0.00061	1.09	FAM204A	family with sequence similarity 204, member A	Upregulated
A_23_P256	0.003239	0.000641	1.09	TLR6	toll-like receptor 6	Upregulated
A_23_P106	0.003456	0.000704	1.09	DDX28	DEAD (Asp-Glu-Ala-Asp) box polypeptide 28	Upregulated
A_21_P001	0.004102	0.000905	1.09	LOC1002880	uncharacterized LOC100288069	Upregulated
A_33_P342	0.004626	0.00108	1.09	C8orf59	chromosome 8 open reading frame 59	Upregulated

A_22_P000	0.004856	0.00116	1.09	LOC1019277	uncharacterized LOC101927734	Upregulated
A_32_P414	0.005095	0.00124	1.09	THOC3	THO complex 3	Upregulated
A_22_P000	0.005732	0.00148	1.09	lnc-PPP2R2D	lnc-PPP2R2D-3:1	Upregulated
A_21_P000	0.007258	0.00207	1.09	lnc-C1orf195-	lnc-C1orf195-3:2	Upregulated
A_23_P129	0.008618	0.00262	1.09	SREBF1	sterol regulatory element binding transcription factor 1	Upregulated
A_23_P789	0.009825	0.00312	1.09	DUSP22	dual specificity phosphatase 22	Upregulated
A_22_P000	0.024792	0.0102	1.09	LINC01405	long intergenic non-protein coding RNA 1405	Upregulated
A_23_P203	0.025133	0.0104	1.09	PSMD9	proteasome (prosome, macropain) 26S subunit, non-ATPase, 9	Upregulated
A_33_P332	0.032347	0.0142	1.09	ZNF320	zinc finger protein 320	Upregulated
A_33_P336	0.034497	0.0153	1.09	ARAF	A-Raf proto-oncogene, serine/threonine kinase	Upregulated
A_21_P000	0.044049	0.0206	1.09	SNORA25	small nucleolar RNA, H/ACA box 25	Upregulated
A_23_P928	0.001986	0.000296	1.08	SAR1B	secretion associated, Ras related GTPase 1B	Upregulated
A_33_P329	0.002026	0.000307	1.08	TCOF1	Treacher Collins-Franceschetti syndrome 1	Upregulated
A_23_P163	0.002113	0.000331	1.08	EHD4	EH-domain containing 4	Upregulated
A_22_P000	0.002166	0.000345	1.08	lnc-MRPL39-2	lnc-MRPL39-2:1	Upregulated
A_33_P332	0.002186	0.000351	1.08	RGP1	RGP1 retrograde golgi transport homolog (S. cerevisiae)	Upregulated
A_24_P351	0.002191	0.000352	1.08	MREG	melanoregulin	Upregulated
A_33_P324	0.002243	0.000366	1.08	ADIPOR1	adiponectin receptor 1	Upregulated
A_32_P620	0.002243	0.000366	1.08	RPL10A	ribosomal protein L10a	Upregulated
A_23_P202	0.002439	0.000412	1.08	ZBTB44	zinc finger and BTB domain containing 44	Upregulated
A_33_P331	0.002479	0.000423	1.08	PACSIN2	protein kinase C and casein kinase substrate in neurons 2	Upregulated
A_33_P339	0.002589	0.000454	1.08	USP32	ubiquitin specific peptidase 32	Upregulated
A_23_P164	0.002667	0.000474	1.08	MMD	monocyte to macrophage differentiation-associated	Upregulated
A_32_P311	0.002835	0.00052	1.08	RPL7	ribosomal protein L7	Upregulated
A_23_P572	0.002888	0.000535	1.08	URB1	URB1 ribosome biogenesis 1 homolog (S. cerevisiae)	Upregulated
A_23_P134	0.003929	0.000851	1.08	C7orf50	chromosome 7 open reading frame 50	Upregulated
A_23_P418	0.004176	0.00093	1.08	TNKS1BP1	tankyrase 1 binding protein 1, 182kDa	Upregulated
A_23_P400	0.004913	0.00118	1.08	TRMT10B	tRNA methyltransferase 10 homolog B (S. cerevisiae)	Upregulated
A_24_P156	0.005496	0.00139	1.08	LYSMD3	LysM, putative peptidoglycan-binding, domain containing 3	Upregulated
A_33_P326	0.00581	0.00151	1.08	CBFA2T2	core-binding factor, runt domain, alpha subunit 2; translocated to, 2	Upregulated
A_22_P000	0.010756	0.0035	1.08	LOC1019270	uncharacterized LOC101927027	Upregulated
A_33_P324	0.011371	0.00378	1.08	PC	pyruvate carboxylase	Upregulated
A_21_P001	0.012852	0.00442	1.08	LINC01057	long intergenic non-protein coding RNA 1057	Upregulated
A_33_P336	0.012907	0.00444	1.08	KLHL36	kelch-like family member 36	Upregulated
A_33_P335	0.01307	0.00452	1.08	TDG	thymine-DNA glycosylase	Upregulated
A_32_P104	0.014406	0.00514	1.08	DCUN1D3	DCN1, defective in cullin neddylation 1, domain containing 3	Upregulated
A_23_P428	0.014743	0.0053	1.08	TTC21A	tetratricopeptide repeat domain 21A	Upregulated

A_21_P001	0.019849	0.0077	1.08	LOC1010605	uncharacterized LOC101060542	Upregulated
A_24_P265	0.020111	0.00783	1.08	NTRK1	neurotrophic tyrosine kinase, receptor, type 1	Upregulated
A_22_P000	0.026781	0.0112	1.08	PSMD5-AS1	PSMD5 antisense RNA 1 (head to head)	Upregulated
A_22_P000	0.029356	0.0126	1.08	lnc-SPP2-1	lnc-SPP2-1:1	Upregulated
A_33_P329	0.033413	0.0147	1.08	TBX5-AS1	TBX5 antisense RNA 1	Upregulated
A_24_P111	0.033779	0.0149	1.08	POMT2	protein-O-mannosyltransferase 2	Upregulated
A_23_P106	0.037257	0.0168	1.08	LCMT2	leucine carboxyl methyltransferase 2	Upregulated
A_22_P000	0.038334	0.0174	1.08	lnc-TSPAN12	lnc-TSPAN12-1:1	Upregulated
A_21_P000	0.047102	0.0223	1.08	lnc-PHF3-4	lnc-PHF3-4:1	Upregulated
A_23_P219	0.048025	0.0229	1.08	ZNF3	zinc finger protein 3	Upregulated
A_21_P000	0.054726	0.0268	1.08	SNORA41	small nucleolar RNA, H/ACA box 41	Upregulated
A_22_P000	0.059292	0.0294	1.08	lnc-HIATL1-5	lnc-HIATL1-5:1	Upregulated
A_33_P338	0.061982	0.0311	1.08	FANCF	Fanconi anemia, complementation group F	Upregulated
A_22_P000	0.071841	0.037	1.08	lnc-PRPF19-1	lnc-PRPF19-1:1	Upregulated
A_22_P000	0.084486	0.0451	1.08	lnc-COQ9-1	lnc-COQ9-1:1	Upregulated
A_23_P329	0.085404	0.0458	1.08	GREB1	growth regulation by estrogen in breast cancer 1	Upregulated
A_24_P673	0.002046	0.000313	1.07	FABP5	fatty acid binding protein 5 (psoriasis-associated)	Upregulated
A_33_P332	0.002043	0.000313	1.07	FCGR2B	Fc fragment of IgG, low affinity IIb, receptor (CD32)	Upregulated
A_23_P212	0.002253	0.000369	1.07	RAB5A	RAB5A, member RAS oncogene family	Upregulated
A_23_P998	0.00247	0.000421	1.07	FAM214A	family with sequence similarity 214, member A	Upregulated
A_33_P342	0.002494	0.000428	1.07	ZNF770	zinc finger protein 770	Upregulated
A_23_P214	0.002693	0.000481	1.07	PERP	PERP, TP53 apoptosis effector	Upregulated
A_23_P117	0.002724	0.00049	1.07	AHSA1	AHA1, activator of heat shock 90kDa protein ATPase homolog 1 (yeast	Upregulated
A_33_P327	0.002761	0.0005	1.07	PPP1R14B	protein phosphatase 1, regulatory (inhibitor) subunit 14B	Upregulated
A_23_P115	0.003248	0.000643	1.07	SZRD1	SUZ RNA binding domain containing 1	Upregulated
A_23_P147	0.003401	0.000689	1.07	TSEN54	TSEN54 tRNA splicing endonuclease subunit	Upregulated
A_33_P327	0.003527	0.000725	1.07	RNF126	ring finger protein 126	Upregulated
A_23_P117	0.003703	0.00078	1.07	BAHD1	bromo adjacent homology domain containing 1	Upregulated
A_24_P113	0.004238	0.000953	1.07	ZNHIT6	zinc finger, HIT-type containing 6	Upregulated
A_23_P341	0.004262	0.00096	1.07	IDH3G	isocitrate dehydrogenase 3 (NAD+) gamma	Upregulated
A_21_P001	0.005008	0.00122	1.07	XLOC_l2_012802		Upregulated
A_23_P214	0.005327	0.00133	1.07	EXOC2	exocyst complex component 2	Upregulated
A_33_P325	0.006386	0.00173	1.07	RNF38	ring finger protein 38	Upregulated
A_24_P325	0.006513	0.00178	1.07	RNF214	ring finger protein 214	Upregulated
A_33_P327	0.006811	0.00189	1.07	PHF20L1	PHD finger protein 20-like 1	Upregulated
A_33_P333	0.007028	0.00198	1.07	GLIS2	GLIS family zinc finger 2	Upregulated
A_33_P338	0.008115	0.00241	1.07	SIKE1	suppressor of IKBKE 1	Upregulated

A_22_P000	0.008157	0.00243	1.07	lnc-EMX2-3	lnc-EMX2-3:1	Upregulated
A_23_P559	0.009803	0.00311	1.07	NAPA	N-ethylmaleimide-sensitive factor attachment protein, alpha	Upregulated
A_24_P162	0.009878	0.00314	1.07	ERC1	ELKS/RAB6-interacting/CAST family member 1	Upregulated
A_33_P338	0.010424	0.00337	1.07	TAT-AS1	TAT antisense RNA 1	Upregulated
A_24_P322	0.012389	0.00422	1.07	DIDO1	death inducer-obliterator 1	Upregulated
A_23_P933	0.012465	0.00425	1.07	NUDT3	nudix (nucleoside diphosphate linked moiety X)-type motif 3	Upregulated
A_23_P169	0.018847	0.00722	1.07	ORM1	orosomucoid 1	Upregulated
A_32_P578	0.020454	0.00799	1.07	RNF157	ring finger protein 157	Upregulated
A_33_P324	0.027772	0.0117	1.07	CSMD2	CUB and Sushi multiple domains 2	Upregulated
A_33_P324	0.041151	0.019	1.07	RBM12B	RNA binding motif protein 12B	Upregulated
A_22_P000	0.048413	0.0231	1.07	lnc-TLCD1-1	lnc-TLCD1-1:1	Upregulated
A_24_P205	0.065493	0.0331	1.07	SHMT1	serine hydroxymethyltransferase 1 (soluble)	Upregulated
A_21_P000	0.069437	0.0355	1.07	LOC1027247	uncharacterized LOC102724732	Upregulated
A_21_P001	0.089753	0.0486	1.07	XLOC_l2_014830		Upregulated
A_22_P000	0.002062	0.000317	1.06	VASH1	vasohibin 1	Upregulated
A_33_P329	0.002238	0.000364	1.06	NAA35	N(alpha)-acetyltransferase 35, NatC auxiliary subunit	Upregulated
A_23_P118	0.002347	0.00039	1.06	SUMO2	small ubiquitin-like modifier 2	Upregulated
A_33_P335	0.002395	0.000403	1.06	ARCN1	archain 1	Upregulated
A_33_P380	0.002471	0.000421	1.06	DNAJA1P5	DnaJ (Hsp40) homolog, subfamily A, member 1 pseudogene 5	Upregulated
A_23_P354	0.002624	0.000463	1.06	LYST	lysosomal trafficking regulator	Upregulated
A_23_P881	0.002657	0.000471	1.06	HSPH1	heat shock 105kDa/110kDa protein 1	Upregulated
A_33_P335	0.002872	0.00053	1.06	MTA1	metastasis associated 1	Upregulated
A_23_P121	0.002963	0.000557	1.06	THG1L	tRNA-histidine guanylyltransferase 1-like (S. cerevisiae)	Upregulated
A_33_P323	0.003008	0.00057	1.06	MAP2K3	mitogen-activated protein kinase kinase 3	Upregulated
A_23_P648	0.003428	0.000696	1.06	SELPLG	selectin P ligand	Upregulated
A_23_P387	0.00411	0.000907	1.06	MICB	MHC class I polypeptide-related sequence B	Upregulated
A_24_P398	0.004951	0.00119	1.06	TRIM34	tripartite motif containing 34	Upregulated
A_24_P115	0.005608	0.00144	1.06	DAGLA	diacylglycerol lipase, alpha	Upregulated
A_33_P339	0.005608	0.00144	1.06	PCDHGA5	protocadherin gamma subfamily A, 5	Upregulated
A_23_P771	0.007727	0.00226	1.06	RNF111	ring finger protein 111	Upregulated
A_23_P102	0.01557	0.00568	1.06	CHD6	chromodomain helicase DNA binding protein 6	Upregulated
A_22_P000	0.017477	0.00658	1.06	LINC00648	long intergenic non-protein coding RNA 648	Upregulated
A_33_P331	0.017999	0.00682	1.06	ANO7	anoctamin 7	Upregulated
A_23_P906	0.022678	0.00907	1.06	STRADB	STE20-related kinase adaptor beta	Upregulated
A_22_P000	0.030594	0.0132	1.06	lnc-INPP5F-1	lnc-INPP5F-1:1	Upregulated
A_33_P331	0.035881	0.0161	1.06	METTL4	methyltransferase like 4	Upregulated
A_23_P933	0.052653	0.0255	1.06	DDR1	discoidin domain receptor tyrosine kinase 1	Upregulated

A_23_P107	0.002078	0.000322	1.05	RPA1	replication protein A1, 70kDa	Upregulated
A_23_P252	0.002144	0.00034	1.05	MRPS30	mitochondrial ribosomal protein S30	Upregulated
A_23_P725	0.00215	0.000341	1.05	AIFM1	apoptosis-inducing factor, mitochondrion-associated, 1	Upregulated
A_32_P830	0.002196	0.000353	1.05	EFR3B	EFR3 homolog B (S. cerevisiae)	Upregulated
A_22_P000	0.002224	0.00036	1.05	LOC1027240	uncharacterized LOC102724096	Upregulated
A_22_P000	0.002337	0.000387	1.05	LINC00944	long intergenic non-protein coding RNA 944	Upregulated
A_23_P370	0.002491	0.000427	1.05	SMIM4	small integral membrane protein 4	Upregulated
A_22_P000	0.002757	0.000499	1.05	lnc-LEO1-4	lnc-LEO1-4:1	Upregulated
A_21_P001	0.002813	0.000514	1.05	LOC729218	uncharacterized LOC729218	Upregulated
A_24_P641	0.002921	0.000544	1.05	CPNE3	copine III	Upregulated
A_33_P338	0.003054	0.000584	1.05	NOP14	NOP14 nucleolar protein	Upregulated
A_32_P180	0.003057	0.000585	1.05	LOC728323	uncharacterized LOC728323	Upregulated
A_33_P341	0.003084	0.000593	1.05	OXTR	oxytocin receptor	Upregulated
A_21_P000	0.004247	0.000956	1.05	lnc-VKORC1L	lnc-VKORC1L1-1:1	Upregulated
A_33_P329	0.00429	0.00097	1.05	PARP1	poly (ADP-ribose) polymerase 1	Upregulated
A_23_P201	0.004843	0.00116	1.05	ARPC1B	actin related protein 2/3 complex, subunit 1B, 41kDa	Upregulated
A_23_P257	0.004939	0.00119	1.05	HCCS	holocytochrome c synthase	Upregulated
A_22_P000	0.005194	0.00128	1.05	lnc-ZRANB1-2	lnc-ZRANB1-2:1	Upregulated
A_33_P326	0.005191	0.00128	1.05	SAMD9L	sterile alpha motif domain containing 9-like	Upregulated
A_33_P338	0.005253	0.00131	1.05	FAM222B	family with sequence similarity 222, member B	Upregulated
A_33_P346	0.005653	0.00145	1.05	LOC286154	uncharacterized LOC286154	Upregulated
A_24_P341	0.006994	0.00197	1.05	TMEM75	transmembrane protein 75	Upregulated
A_23_P315	0.007563	0.00219	1.05	EMX1	empty spiracles homeobox 1	Upregulated
A_33_P334	0.0083	0.00249	1.05	SYNGR2	synaptogyrin 2	Upregulated
A_21_P000	0.009143	0.00284	1.05	lnc-CRISP2-1	lnc-CRISP2-1:1	Upregulated
A_21_P000	0.010096	0.00323	1.05	lnc-SYNDIG1-	lnc-SYNDIG1-4:1	Upregulated
A_33_P332	0.012926	0.00445	1.05	PLXNA4	plexin A4	Upregulated
A_21_P000	0.014243	0.00506	1.05	lnc-ADCY2-1	lnc-ADCY2-1:1	Upregulated
A_33_P333	0.015437	0.00562	1.05	RTCA	RNA 3'-terminal phosphate cyclase	Upregulated
A_22_P000	0.016738	0.00624	1.05	RFESD	Rieske (Fe-S) domain containing	Upregulated
A_23_P432	0.021482	0.00848	1.05	CCDC117	coiled-coil domain containing 117	Upregulated
A_33_P325	0.027925	0.0118	1.05	GRIP1	glutamate receptor interacting protein 1	Upregulated
A_33_P327	0.036788	0.0166	1.05	SPATA21	spermatogenesis associated 21	Upregulated
A_33_P338	0.045923	0.0217	1.05	ZNF511	zinc finger protein 511	Upregulated
A_33_P325	0.080564	0.0425	1.05	PLAC9	placenta-specific 9	Upregulated
A_32_P191	0.080627	0.0426	1.05	SCGB2B2	secretoglobin, family 2B, member 2	Upregulated
A_22_P000	0.089781	0.0487	1.05	lnc-GPN2-1	lnc-GPN2-1:1	Upregulated

A_33_P324	0.002439	0.000412	1.04	CD69	CD69 molecule	Upregulated
A_19_P003	0.00248	0.000424	1.04	SNHG5	small nucleolar RNA host gene 5 (non-protein coding)	Upregulated
A_21_P000	0.002503	0.000431	1.04	lnc-AL669831	lnc-AL669831.1-4:1	Upregulated
A_23_P351	0.002649	0.000469	1.04	TAF13	TAF13 RNA polymerase II, TATA box binding protein (TBP)-associated f	Upregulated
A_23_P887	0.00284	0.000522	1.04	RAD51	RAD51 recombinase	Upregulated
A_23_P156	0.002967	0.000558	1.04	LNPEP	leucyl/cystinyl aminopeptidase	Upregulated
A_24_P230	0.003022	0.000575	1.04	CCDC137	coiled-coil domain containing 137	Upregulated
A_23_P312	0.003057	0.000585	1.04	TUBB4B	tubulin, beta 4B class IVb	Upregulated
A_24_P144	0.003398	0.000688	1.04	RNF6	ring finger protein (C3H2C3 type) 6	Upregulated
A_23_P149	0.003516	0.000722	1.04	SDHB	succinate dehydrogenase complex, subunit B, iron sulfur (Ip)	Upregulated
A_21_P000	0.003564	0.000738	1.04	lnc-KIF20B-5	lnc-KIF20B-5:1	Upregulated
A_23_P169	0.003574	0.000743	1.04	CDK9	cyclin-dependent kinase 9	Upregulated
A_24_P488	0.003904	0.000843	1.04	SNAP29	synaptosomal-associated protein, 29kDa	Upregulated
A_33_P322	0.004183	0.000932	1.04	ZNF284	zinc finger protein 284	Upregulated
A_32_P149	0.00422	0.000947	1.04	LINC00515	long intergenic non-protein coding RNA 515	Upregulated
A_24_P241	0.004294	0.000971	1.04	EXOSC8	exosome component 8	Upregulated
A_24_P767	0.004641	0.00109	1.04	CC2D1B	coiled-coil and C2 domain containing 1B	Upregulated
A_32_P199	0.004648	0.00109	1.04	TFDP1	transcription factor Dp-1	Upregulated
A_19_P003	0.005773	0.0015	1.04	ZSWIM6	zinc finger, SWIM-type containing 6	Upregulated
A_32_P144	0.006725	0.00186	1.04	ZNF518B	zinc finger protein 518B	Upregulated
A_21_P000	0.009922	0.00316	1.04	lnc-CTAGE5-1	lnc-CTAGE5-1:5	Upregulated
A_23_P347	0.016148	0.00595	1.04	MOB1B	MOB kinase activator 1B	Upregulated
A_21_P000	0.017109	0.00641	1.04	lnc-RP11-597	lnc-RP11-597K23.2.1-2:1	Upregulated
A_24_P199	0.020609	0.00806	1.04	VPS54	vacuolar protein sorting 54 homolog (S. cerevisiae)	Upregulated
A_21_P000	0.024291	0.00993	1.04	lnc-SREK1-1	lnc-SREK1-1:4	Upregulated
A_23_P467	0.032859	0.0144	1.04	EPC1	enhancer of polycomb homolog 1 (Drosophila)	Upregulated
A_21_P000	0.041057	0.0189	1.04	HCG14	HLA complex group 14 (non-protein coding)	Upregulated
A_23_P250	0.044142	0.0207	1.04	IGF2BP2	insulin-like growth factor 2 mRNA binding protein 2	Upregulated
A_22_P000	0.045941	0.0217	1.04	lnc-TRPM2-1	lnc-TRPM2-1:1	Upregulated
A_23_P383	0.048442	0.0231	1.04	SNAPC5	small nuclear RNA activating complex, polypeptide 5, 19kDa	Upregulated
A_21_P000	0.051415	0.0248	1.04	lnc-CCND1-1	lnc-CCND1-1:1	Upregulated
A_19_P003	0.085527	0.0459	1.04	APCDD1L-AS	APCDD1L antisense RNA 1 (head to head)	Upregulated
A_23_P475	0.002197	0.000353	1.03	LDHA	lactate dehydrogenase A	Upregulated
A_21_P000	0.00226	0.00037	1.03	ATP1A1-AS1	ATP1A1 antisense RNA 1	Upregulated
A_33_P320	0.00232	0.000383	1.03	CD58	CD58 molecule	Upregulated
A_23_P115	0.00238	0.000398	1.03	HPS6	Hermansky-Pudlak syndrome 6	Upregulated
A_23_P134	0.002478	0.000423	1.03	MAP3K5	mitogen-activated protein kinase kinase kinase 5	Upregulated

A_33_P336	0.002508	0.000432	1.03	NAMPT	nicotinamide phosphoribosyltransferase	Upregulated
A_33_P330	0.00255	0.000443	1.03	SRSF10	serine/arginine-rich splicing factor 10	Upregulated
A_19_P003	0.002592	0.000454	1.03	SNHG5	small nucleolar RNA host gene 5 (non-protein coding)	Upregulated
A_23_P245	0.002644	0.000468	1.03	C11orf57	chromosome 11 open reading frame 57	Upregulated
A_21_P000	0.002734	0.000492	1.03	lnc-PMFBP1-2	lnc-PMFBP1-2:1	Upregulated
A_33_P324	0.002888	0.000535	1.03	SLC4A5	solute carrier family 4 (sodium bicarbonate cotransporter), member 5	Upregulated
A_23_P828	0.003738	0.000792	1.03	IFNGR1	interferon gamma receptor 1	Upregulated
A_33_P325	0.004106	0.000906	1.03	MFHAS1	malignant fibrous histiocytoma amplified sequence 1	Upregulated
A_23_P395	0.004313	0.000978	1.03	FBXO31	F-box protein 31	Upregulated
A_33_P341	0.004786	0.00114	1.03	SNX8	sorting nexin 8	Upregulated
A_23_P500	0.005029	0.00122	1.03	B3GNT2	UDP-GlcNAc:betaGal beta-1,3-N-acetylglucosaminyltransferase 2	Upregulated
A_23_P309	0.005241	0.0013	1.03	HIST2H2AA4	histone cluster 2, H2aa4	Upregulated
A_23_P251	0.005278	0.00132	1.03	CHRNA2	cholinergic receptor, nicotinic, alpha 2 (neuronal)	Upregulated
A_24_P247	0.005925	0.00155	1.03	ZNF589	zinc finger protein 589	Upregulated
A_22_P000	0.006513	0.00178	1.03	lnc-DHRS7B-3	lnc-DHRS7B-3:1	Upregulated
A_24_P861	0.007422	0.00213	1.03	BRWD1	bromodomain and WD repeat domain containing 1	Upregulated
A_33_P377	0.008914	0.00274	1.03	KRT8P12	keratin 8 pseudogene 12	Upregulated
A_22_P000	0.009695	0.00306	1.03	ANKRD44-IT1	ANKRD44 intronic transcript 1 (non-protein coding)	Upregulated
A_23_P713	0.00982	0.00312	1.03	CCDC25	coiled-coil domain containing 25	Upregulated
A_21_P000	0.010993	0.0036	1.03	SNORA68	small nucleolar RNA, H/ACA box 68	Upregulated
A_23_P148	0.014554	0.0052	1.03	FTHL17	ferritin, heavy polypeptide-like 17	Upregulated
A_23_P413	0.015515	0.00566	1.03	HAUS1	HAUS augmin-like complex, subunit 1	Upregulated
A_33_P325	0.016087	0.00592	1.03	MEG3	maternally expressed 3 (non-protein coding)	Upregulated
A_32_P103	0.016795	0.00627	1.03	B4GALT4	UDP-Gal:betaGlcNAc beta 1,4- galactosyltransferase, polypeptide 4	Upregulated
A_22_P000	0.027449	0.0116	1.03	lnc-PABPC4-1	lnc-PABPC4-1:1	Upregulated
A_22_P000	0.031596	0.0137	1.03	ABO	ABO blood group (transferase A, alpha 1-3-N-acetylgalactosaminyltrans	Upregulated
A_21_P000	0.031596	0.0137	1.03	lnc-C17orf58-	lnc-C17orf58-5:1	Upregulated
A_24_P226	0.031962	0.0139	1.03	FGFBP2	fibroblast growth factor binding protein 2	Upregulated
A_22_P000	0.031962	0.0139	1.03	lnc-HEPH-2	lnc-HEPH-2:1	Upregulated
A_21_P000	0.031962	0.0139	1.03	lnc-OR5H15-1	lnc-OR5H15-1:1	Upregulated
A_23_P336	0.037331	0.0169	1.03	GEMIN5	gem (nuclear organelle) associated protein 5	Upregulated
A_24_P626	0.086889	0.0468	1.03	MUC3	intestinal mucin-like	Upregulated
A_33_P332	0.002311	0.000381	1.02	HSPE1	heat shock 10kDa protein 1	Upregulated
A_23_P116	0.002369	0.000396	1.02	LGALS9	lectin, galactoside-binding, soluble, 9	Upregulated
A_23_P359	0.002515	0.000434	1.02	NFAT5	nuclear factor of activated T-cells 5, tonicity-responsive	Upregulated
A_33_P335	0.002561	0.000446	1.02	COQ10B	coenzyme Q10 homolog B (S. cerevisiae)	Upregulated
A_33_P326	0.002618	0.000461	1.02	WIBG	within bgcn homolog (Drosophila)	Upregulated

A_33_P336	0.002676	0.000476	1.02	EIF1AX	eukaryotic translation initiation factor 1A, X-linked	Upregulated
A_21_P000	0.002876	0.000532	1.02	lnc-LHX2-1	lnc-LHX2-1:1	Upregulated
A_23_P321	0.00293	0.000546	1.02	BCL2A1	BCL2-related protein A1	Upregulated
A_23_P304	0.003096	0.000596	1.02	CHRNE	cholinergic receptor, nicotinic, epsilon (muscle)	Upregulated
A_23_P354	0.003125	0.000606	1.02	KCTD11	potassium channel tetramerization domain containing 11	Upregulated
A_24_P101	0.003335	0.000669	1.02	PDIA3	protein disulfide isomerase family A, member 3	Upregulated
A_33_P323	0.003392	0.000686	1.02	SBDS	Shwachman-Bodian-Diamond syndrome	Upregulated
A_22_P000	0.003638	0.00076	1.02	lnc-CCDC7-4	lnc-CCDC7-4:1	Upregulated
A_23_P611	0.003798	0.00081	1.02	SEC23B	Sec23 homolog B (S. cerevisiae)	Upregulated
A_23_P251	0.004094	0.000902	1.02	FKBP11	FK506 binding protein 11, 19 kDa	Upregulated
A_23_P194	0.004524	0.00105	1.02	DDAH2	dimethylarginine dimethylaminohydrolase 2	Upregulated
A_33_P331	0.004589	0.00107	1.02	RHOG	ras homolog family member G	Upregulated
A_23_P945	0.004744	0.00112	1.02	POLR1E	polymerase (RNA) I polypeptide E, 53kDa	Upregulated
A_33_P323	0.005113	0.00125	1.02	ALG11	ALG11, alpha-1,2-mannosyltransferase	Upregulated
A_33_P333	0.005207	0.00129	1.02	SEC24A	SEC24 family member A	Upregulated
A_22_P000	0.00576	0.00149	1.02	lnc-ARHGAP1	lnc-ARHGAP17-1:1	Upregulated
A_33_P333	0.005832	0.00152	1.02	MSTO1	misato 1, mitochondrial distribution and morphology regulator	Upregulated
A_19_P003	0.00608	0.00161	1.02	TDRG1	testis development related 1 (non-protein coding)	Upregulated
A_23_P436	0.008013	0.00237	1.02	RBM34	RNA binding motif protein 34	Upregulated
A_21_P000	0.009032	0.00279	1.02	LOC646938	TBC1 domain family, member 2B pseudogene	Upregulated
A_21_P000	0.009869	0.00314	1.02	lnc-BATF3-2	lnc-BATF3-2:7	Upregulated
A_33_P325	0.010129	0.00325	1.02	PPIL4	peptidylprolyl isomerase (cyclophilin)-like 4	Upregulated
A_33_P326	0.012589	0.0043	1.02	CYP26C1	cytochrome P450, family 26, subfamily C, polypeptide 1	Upregulated
A_24_P296	0.014395	0.00513	1.02	ZMIZ2	zinc finger, MIZ-type containing 2	Upregulated
A_23_P206	0.018666	0.00714	1.02	AEN	apoptosis enhancing nuclease	Upregulated
A_23_P748	0.022067	0.00877	1.02	GJA9	gap junction protein, alpha 9, 59kDa	Upregulated
A_33_P322	0.031643	0.0138	1.02	PCNXL4	pecanex-like 4 (Drosophila)	Upregulated
A_23_P170	0.033989	0.015	1.02	MRPL12	mitochondrial ribosomal protein L12	Upregulated
A_22_P000	0.069042	0.0353	1.02	lnc-ADRB1-2	lnc-ADRB1-2:1	Upregulated
A_21_P000	0.071036	0.0365	1.02	lnc-FBXO32-1	lnc-FBXO32-1:1	Upregulated
A_22_P000	0.0822	0.0435	1.02	LINC00316	long intergenic non-protein coding RNA 316	Upregulated
A_21_P000	0.089984	0.0488	1.02	lnc-C7orf41-1	lnc-C7orf41-1:1	Upregulated
A_33_P330	0.002353	0.000391	1.01	RPS5	ribosomal protein S5	Upregulated
A_22_P000	0.002357	0.000392	1.01	lnc-HLA-DMA-	lnc-HLA-DMA-1:1	Upregulated
A_23_P350	0.002504	0.000431	1.01	TET2	tet methylcytosine dioxygenase 2	Upregulated
A_23_P920	0.002517	0.000434	1.01	SETD5	SET domain containing 5	Upregulated
A_23_P102	0.002528	0.000437	1.01	CTSE	cathepsin E	Upregulated

A_33_P321	0.002746	0.000496	1.01	NCF1	neutrophil cytosolic factor 1	Upregulated
A_33_P342	0.00275	0.000497	1.01	TP53INP1	tumor protein p53 inducible nuclear protein 1	Upregulated
A_32_P326	0.00281	0.000513	1.01	KRR1	KRR1, small subunit (SSU) processome component, homolog (yeast)	Upregulated
A_33_P323	0.003106	0.0006	1.01	ZNF497	zinc finger protein 497	Upregulated
A_23_P209	0.003415	0.000693	1.01	ATL2	atlastin GTPase 2	Upregulated
A_24_P272	0.003497	0.000716	1.01	SCIMP	SLP adaptor and CSK interacting membrane protein	Upregulated
A_33_P322	0.00532	0.00133	1.01	AMZ2	archaelysin family metallopeptidase 2	Upregulated
A_23_P318	0.006955	0.00195	1.01	PLEKHA8	pleckstrin homology domain containing, family A (phosphoinositide bindi	Upregulated
A_23_P166	0.00901	0.00278	1.01	OSM	oncostatin M	Upregulated
A_33_P335	0.011555	0.00386	1.01	LOC1009962	uncharacterized LOC100996273	Upregulated
A_22_P000	0.011641	0.0039	1.01	LOC1027245	uncharacterized LOC102724596	Upregulated
A_23_P388	0.015066	0.00545	1.01	DMAP1	DNA methyltransferase 1 associated protein 1	Upregulated
A_21_P000	0.015066	0.00545	1.01	lnc-SBDS-8	lnc-SBDS-8:1	Upregulated
A_33_P326	0.01536	0.00558	1.01	RFX3	regulatory factor X, 3 (influences HLA class II expression)	Upregulated
A_21_P000	0.017465	0.00658	1.01	lnc-FAM153C	lnc-FAM153C-3:1	Upregulated
A_33_P332	0.017976	0.00681	1.01	DENND3	DENN/MADD domain containing 3	Upregulated
A_33_P339	0.025963	0.0108	1.01	LOC1001297	uncharacterized LOC100129785	Upregulated
A_22_P000	0.034547	0.0153	1.01	lnc-ACOX3-3	lnc-ACOX3-3:1	Upregulated
A_21_P001	0.04387	0.0205	1.01	ANKRD20A5I	ankyrin repeat domain 20 family, member A5, pseudogene	Upregulated
A_21_P001	0.063582	0.032	1.01	ANKRD20A2	ankyrin repeat domain 20 family, member A2	Upregulated
A_21_P000	0.064668	0.0326	1.01	lnc-ZNF487P-	lnc-ZNF487P-1:1	Upregulated
A_22_P000	0.072556	0.0374	1.01	lnc-QPCT-3	lnc-QPCT-3:4	Upregulated
A_22_P000	0.002356	0.000392	1	DNAJC27-AS	DNAJC27 antisense RNA 1	Upregulated
A_21_P001	0.002445	0.000414	1	EIF4A1	eukaryotic translation initiation factor 4A1	Upregulated
A_32_P447	0.002495	0.000428	1	C9orf85	chromosome 9 open reading frame 85	Upregulated
A_24_P295	0.002853	0.000525	1	BLOC1S2	biogenesis of lysosomal organelles complex-1, subunit 2	Upregulated
A_33_P330	0.002853	0.000525	1	RWDD1	RWD domain containing 1	Upregulated
A_23_P206	0.002872	0.000531	1	WWP2	WW domain containing E3 ubiquitin protein ligase 2	Upregulated
A_24_P942	0.002881	0.000533	1	KDM6B	lysine (K)-specific demethylase 6B	Upregulated
A_33_P333	0.002882	0.000534	1	POLR1C	polymerase (RNA) I polypeptide C, 30kDa	Upregulated
A_23_P387	0.002989	0.000565	1	TUBB	tubulin, beta class I	Upregulated
A_21_P000	0.003081	0.000592	1	RPS3A	ribosomal protein S3A	Upregulated
A_33_P321	0.003175	0.000622	1	ZWINT	ZW10 interacting kinetochore protein	Upregulated
A_23_P123	0.004455	0.00103	1	LRSAM1	leucine rich repeat and sterile alpha motif containing 1	Upregulated
A_22_P000	0.005537	0.00141	1	lnc-HDDC2-5	lnc-HDDC2-5:3	Upregulated
A_23_P426	0.006757	0.00187	1	SH3BP5L	SH3-binding domain protein 5-like	Upregulated
A_24_P125	0.009895	0.00315	1	BCCIP	BRCA2 and CDKN1A interacting protein	Upregulated

A_33_P660	0.010112	0.00324	1	YTHDF3	YTH N(6)-methyladenosine RNA binding protein 3	Upregulated
A_22_P000	0.011014	0.00361	1	INTS6-AS1	INTS6 antisense RNA 1	Upregulated
A_23_P343	0.013026	0.0045	1	PARP11	poly (ADP-ribose) polymerase family, member 11	Upregulated
A_32_P132	0.01568	0.00573	1	FAM86JP	family with sequence similarity 86, member A pseudogene	Upregulated
A_22_P000	0.016357	0.00606	1	LOC115110	uncharacterized LOC115110	Upregulated
A_33_P328	0.020165	0.00785	1	LRFN4	leucine rich repeat and fibronectin type III domain containing 4	Upregulated
A_33_P331	0.023452	0.00947	1	LONP1	lon peptidase 1, mitochondrial	Upregulated
A_23_P321	0.051156	0.0247	1	DEF6	differentially expressed in FDCP 6 homolog (mouse)	Upregulated
A_23_P211	0.002309	0.000381	-1	UBP1	upstream binding protein 1 (LBP-1a)	Downregulated
A_19_P003	0.00249	0.000427	-1	LOC1004227	uncharacterized LOC100422737	Downregulated
A_33_P332	0.002511	0.000433	-1	CIITA	class II, major histocompatibility complex, transactivator	Downregulated
A_22_P000	0.002572	0.00045	-1	lnc-TMEM105	lnc-TMEM105-1:1	Downregulated
A_23_P949	0.002798	0.000509	-1	MMADHC	methylmalonic aciduria (cobalamin deficiency) cblD type, with homocystir	Downregulated
A_23_P310	0.003012	0.000572	-1	NUDT16	nudix (nucleoside diphosphate linked moiety X)-type motif 16	Downregulated
A_33_P341	0.003033	0.000578	-1	MLH1	mutL homolog 1	Downregulated
A_33_P331	0.003277	0.000652	-1	FLJ44674	FLJ44674 protein	Downregulated
A_23_P674	0.003794	0.000808	-1	PSMD8	proteasome (prosome, macropain) 26S subunit, non-ATPase, 8	Downregulated
A_33_P339	0.003806	0.000813	-1	GUK1	guanylate kinase 1	Downregulated
A_33_P327	0.004147	0.000919	-1	TXNDC8	thioredoxin domain containing 8 (spermatozoa)	Downregulated
A_33_P333	0.004928	0.00119	-1	MECP2	methyl CpG binding protein 2	Downregulated
A_22_P000	0.005491	0.00139	-1	LMO7	LIM domain 7	Downregulated
A_21_P000	0.005537	0.00141	-1	lnc-PARN-6	lnc-PARN-6:3	Downregulated
A_24_P287	0.005537	0.00141	-1	PSMC3IP	PSMC3 interacting protein	Downregulated
A_23_P239	0.005697	0.00147	-1	FAM188A	family with sequence similarity 188, member A	Downregulated
A_32_P925	0.006983	0.00196	-1	LCLAT1	lysocardiolipin acyltransferase 1	Downregulated
A_23_P736	0.007458	0.00215	-1	NR0B1	nuclear receptor subfamily 0, group B, member 1	Downregulated
A_33_P370	0.007458	0.00215	-1	SNORA28	small nucleolar RNA, H/ACA box 28	Downregulated
A_22_P000	0.007512	0.00217	-1	lnc-RIMS3-2	lnc-RIMS3-2:1	Downregulated
A_24_P801	0.008107	0.00241	-1	PDCL3	phosducin-like 3	Downregulated
A_23_P133	0.008875	0.00272	-1	PPIL1	peptidylprolyl isomerase (cyclophilin)-like 1	Downregulated
A_24_P403	0.008915	0.00274	-1	ZNF385A	zinc finger protein 385A	Downregulated
A_22_P000	0.009491	0.00297	-1	lnc-DOCK8-1	lnc-DOCK8-1:1	Downregulated
A_21_P000	0.009491	0.00297	-1	lnc-PIK3CG-2	lnc-PIK3CG-2:3	Downregulated
A_24_P924	0.013026	0.0045	-1	CFI	complement factor I	Downregulated
A_23_P115	0.014197	0.00503	-1	IPO13	importin 13	Downregulated
A_22_P000	0.014724	0.00529	-1	C7orf13	chromosome 7 open reading frame 13	Downregulated
A_23_P367	0.016265	0.00601	-1	C2CD5	C2 calcium-dependent domain containing 5	Downregulated

A_23_P286	0.017092	0.00641	-1	CPSF3	cleavage and polyadenylation specific factor 3, 73kDa	Downregulated
A_33_P322	0.017554	0.00662	-1	ZNF117	zinc finger protein 117	Downregulated
A_33_P328	0.022678	0.00908	-1	CCDC136	coiled-coil domain containing 136	Downregulated
A_24_P124	0.0342	0.0151	-1	NCOA7	nuclear receptor coactivator 7	Downregulated
A_22_P000	0.03469	0.0154	-1	lnc-ATP6AP2	lnc-ATP6AP2-3:1	Downregulated
A_33_P337	0.03469	0.0154	-1	RGS7BP	regulator of G-protein signaling 7 binding protein	Downregulated
A_21_P000	0.04017	0.0184	-1	lnc-DLGAP2-4	lnc-DLGAP2-4:1	Downregulated
A_21_P001	0.043763	0.0204	-1	XLOC_l2_000423		Downregulated
A_23_P360	0.046842	0.0222	-1	LRWD1	leucine-rich repeats and WD repeat domain containing 1	Downregulated
A_33_P647	0.059444	0.0295	-1	SHANK2-AS1	SHANK2 antisense RNA 1	Downregulated
A_32_P194	0.075725	0.0394	-1	SDK2	sidekick cell adhesion molecule 2	Downregulated
A_21_P000	0.078838	0.0414	-1	lnc-KIDINS22	lnc-KIDINS220-6:7	Downregulated
A_23_P112	0.082675	0.0438	-1	CST1	cystatin SN	Downregulated
A_23_P325	0.002339	0.000388	-1.01	XPO6	exportin 6	Downregulated
A_33_P337	0.00237	0.000396	-1.01	TRAFD1	TRAF-type zinc finger domain containing 1	Downregulated
A_22_P000	0.002521	0.000435	-1.01	lnc-C1orf124-	lnc-C1orf124-2:1	Downregulated
A_23_P456	0.00255	0.000443	-1.01	FUBP1	far upstream element (FUSE) binding protein 1	Downregulated
A_23_P629	0.002707	0.000485	-1.01	BTG2	BTG family, member 2	Downregulated
A_23_P377	0.002859	0.000527	-1.01	SRSF5	serine/arginine-rich splicing factor 5	Downregulated
A_23_P167	0.003012	0.000572	-1.01	IGJ	immunoglobulin J polypeptide, linker protein for immunoglobulin alpha a	Downregulated
A_23_P622	0.003068	0.000588	-1.01	KDM5C	lysine (K)-specific demethylase 5C	Downregulated
A_24_P139	0.003096	0.000597	-1.01	ITCH	itchy E3 ubiquitin protein ligase	Downregulated
A_32_P994	0.003208	0.000632	-1.01	TRAPPC5	trafficking protein particle complex 5	Downregulated
A_22_P000	0.003262	0.000647	-1.01	lnc-ASH2L-1	lnc-ASH2L-1:1	Downregulated
A_22_P000	0.003338	0.000671	-1.01	lnc-TIMM9-2	lnc-TIMM9-2:12	Downregulated
A_23_P105	0.003878	0.000836	-1.01	EIF2B1	eukaryotic translation initiation factor 2B, subunit 1 alpha, 26kDa	Downregulated
A_33_P336	0.004079	0.000896	-1.01	FLNA	filamin A, alpha	Downregulated
A_23_P404	0.004987	0.00121	-1.01	PCTP	phosphatidylcholine transfer protein	Downregulated
A_33_P327	0.006955	0.00195	-1.01	FILIP1	filamin A interacting protein 1	Downregulated
A_33_P333	0.006955	0.00195	-1.01	LINC00113	long intergenic non-protein coding RNA 113	Downregulated
A_33_P331	0.007183	0.00204	-1.01	OR51B6	olfactory receptor, family 51, subfamily B, member 6	Downregulated
A_33_P335	0.00914	0.00283	-1.01	KCMF1	potassium channel modulatory factor 1	Downregulated
A_33_P325	0.012505	0.00427	-1.01	FCHO1	FCH domain only 1	Downregulated
A_19_P003	0.048739	0.0233	-1.01	lnc-ERP44-3	lnc-ERP44-3:3	Downregulated
A_32_P155	0.048739	0.0233	-1.01	PRIMA1	proline rich membrane anchor 1	Downregulated
A_33_P381	0.057747	0.0285	-1.01	LOC344967	acyl-CoA thioesterase 7 pseudogene	Downregulated
A_23_P125	0.068341	0.0348	-1.01	GABRQ	gamma-aminobutyric acid (GABA) A receptor, theta	Downregulated

A_21_P000	0.069528	0.0356	-1.01	ZSCAN12	zinc finger and SCAN domain containing 12	Downregulated
A_24_P142	0.002248	0.000368	-1.02	CHMP4A	charged multivesicular body protein 4A	Downregulated
A_23_P978	0.002277	0.000374	-1.02	LIPA	lipase A, lysosomal acid, cholesterol esterase	Downregulated
A_23_P980	0.00228	0.000375	-1.02	PTEN	phosphatase and tensin homolog	Downregulated
A_24_P239	0.002505	0.000431	-1.02	RNF187	ring finger protein 187	Downregulated
A_23_P211	0.002562	0.000446	-1.02	DYRK1A	dual-specificity tyrosine-(Y)-phosphorylation regulated kinase 1A	Downregulated
A_23_P165	0.002775	0.000504	-1.02	BIN1	bridging integrator 1	Downregulated
A_23_P429	0.003132	0.000608	-1.02	GNPNAT1	glucosamine-phosphate N-acetyltransferase 1	Downregulated
A_23_P119	0.003173	0.000621	-1.02	RETN	resistin	Downregulated
A_22_P000	0.003663	0.000768	-1.02	lnc-DGKE-2	lnc-DGKE-2:1	Downregulated
A_21_P000	0.003826	0.000819	-1.02	AHSA2	AHA1, activator of heat shock 90kDa protein ATPase homolog 2 (yeast	Downregulated
A_33_P333	0.003847	0.000826	-1.02	ZNF428	zinc finger protein 428	Downregulated
A_23_P972	0.004739	0.00112	-1.02	GON4L	gon-4-like (C. elegans)	Downregulated
A_24_P335	0.005623	0.00144	-1.02	SLC7A5	solute carrier family 7 (amino acid transporter light chain, L system), men	Downregulated
A_32_P191	0.006724	0.00186	-1.02	ATAD2B	ATPase family, AAA domain containing 2B	Downregulated
A_23_P139	0.010841	0.00354	-1.02	C11orf73	chromosome 11 open reading frame 73	Downregulated
A_23_P122	0.013064	0.00451	-1.02	DDX41	DEAD (Asp-Glu-Ala-Asp) box polypeptide 41	Downregulated
A_23_P350	0.013248	0.00459	-1.02	ZC3H18	zinc finger CCCH-type containing 18	Downregulated
A_33_P336	0.013308	0.00462	-1.02	NPPB	natriuretic peptide B	Downregulated
A_24_P898	0.016311	0.00604	-1.02	ZNF189	zinc finger protein 189	Downregulated
A_19_P008	0.021061	0.00828	-1.02	lnc-RLIM-3	lnc-RLIM-3:1	Downregulated
A_33_P331	0.021835	0.00866	-1.02	TOR1AIP1	torsin A interacting protein 1	Downregulated
A_33_P348	0.031087	0.0135	-1.02	SPI1	Spi-1 proto-oncogene	Downregulated
A_23_P153	0.034667	0.0154	-1.02	YIF1B	Yip1 interacting factor homolog B (S. cerevisiae)	Downregulated
A_21_P001	0.035247	0.0157	-1.02	XLOC_l2_006745		Downregulated
A_23_P118	0.053877	0.0263	-1.02	TVP23B	trans-golgi network vesicle protein 23 homolog B (S. cerevisiae)	Downregulated
A_23_P346	0.06886	0.0352	-1.02	AVPR2	arginine vasopressin receptor 2	Downregulated
A_22_P000	0.070111	0.0359	-1.02	CASC18	cancer susceptibility candidate 18 (non-protein coding)	Downregulated
A_19_P003	0.07829	0.041	-1.02	LOC1019270	uncharacterized LOC101927043	Downregulated
A_24_P305	0.002219	0.000358	-1.03	PITPNB	phosphatidylinositol transfer protein, beta	Downregulated
A_33_P331	0.002221	0.000359	-1.03	PELI1	pellino E3 ubiquitin protein ligase 1	Downregulated
A_33_P331	0.002249	0.000368	-1.03	PDPR	pyruvate dehydrogenase phosphatase regulatory subunit	Downregulated
A_21_P000	0.002365	0.000395	-1.03	SCARNA18	small Cajal body-specific RNA 18	Downregulated
A_24_P398	0.002381	0.000399	-1.03	USP6NL	USP6 N-terminal like	Downregulated
A_22_P000	0.002423	0.000409	-1.03	CERS6-AS1	CERS6 antisense RNA 1	Downregulated
A_33_P330	0.002533	0.000439	-1.03	KLHL36	kelch-like family member 36	Downregulated
A_33_P333	0.002558	0.000445	-1.03	FBXL20	F-box and leucine-rich repeat protein 20	Downregulated

A_24_P271	0.002559	0.000445	-1.03	SLC2A10	solute carrier family 2 (facilitated glucose transporter), member 10	Downregulated
A_33_P331	0.002874	0.000532	-1.03	TMEM154	transmembrane protein 154	Downregulated
A_23_P255	0.0029	0.000539	-1.03	PLGRKT	plasminogen receptor, C-terminal lysine transmembrane protein	Downregulated
A_22_P000	0.002967	0.000558	-1.03	LOC1005061	uncharacterized LOC100506100	Downregulated
A_24_P172	0.002968	0.000559	-1.03	AARS	alanyl-tRNA synthetase	Downregulated
A_22_P000	0.002995	0.000567	-1.03	lnc-MLH3-1	lnc-MLH3-1:1	Downregulated
A_23_P549	0.003096	0.000597	-1.03	MRPL38	mitochondrial ribosomal protein L38	Downregulated
A_21_P000	0.003109	0.000601	-1.03	SNORD25	small nucleolar RNA, C/D box 25	Downregulated
A_23_P161	0.003795	0.000809	-1.03	PDSS1	prenyl (decaprenyl) diphosphate synthase, subunit 1	Downregulated
A_24_P352	0.003822	0.000818	-1.03	MRPL42	mitochondrial ribosomal protein L42	Downregulated
A_23_P171	0.004113	0.000908	-1.03	EBP	emopamil binding protein (sterol isomerase)	Downregulated
A_23_P786	0.004499	0.00104	-1.03	FARSA	phenylalanyl-tRNA synthetase, alpha subunit	Downregulated
A_23_P215	0.004859	0.00117	-1.03	CDK13	cyclin-dependent kinase 13	Downregulated
A_23_P534	0.004979	0.0012	-1.03	IKBIP	IKBKB interacting protein	Downregulated
A_33_P321	0.00511	0.00125	-1.03	SIKE1	suppressor of IKBKE 1	Downregulated
A_33_P322	0.005864	0.00153	-1.03	ADAMTS10	ADAM metallopeptidase with thrombospondin type 1 motif, 10	Downregulated
A_23_P604	0.007149	0.00203	-1.03	PPP2R4	protein phosphatase 2A activator, regulatory subunit 4	Downregulated
A_32_P101	0.007716	0.00225	-1.03	TMEM106A	transmembrane protein 106A	Downregulated
A_33_P332	0.012991	0.00448	-1.03	MED25	mediator complex subunit 25	Downregulated
A_33_P322	0.019344	0.00746	-1.03	GOLGA6L6	golgin A6 family-like 6	Downregulated
A_21_P000	0.02316	0.00932	-1.03	lnc-GLRX5-1	lnc-GLRX5-1:5	Downregulated
A_23_P111	0.02316	0.00932	-1.03	PPIL6	peptidylprolyl isomerase (cyclophilin)-like 6	Downregulated
A_23_P123	0.031596	0.0137	-1.03	PSMG3-AS1	PSMG3 antisense RNA 1 (head to head)	Downregulated
A_21_P001	0.031596	0.0137	-1.03	ZNF726	zinc finger protein 726	Downregulated
A_23_P184	0.047422	0.0225	-1.03	MAEA	macrophage erythroblast attacher	Downregulated
A_33_P335	0.05406	0.0264	-1.03	HMGA2	high mobility group AT-hook 2	Downregulated
A_21_P000	0.075293	0.0391	-1.03	FLJ20021	uncharacterized LOC90024	Downregulated
A_33_P332	0.091663	0.0499	-1.03	TTN	titin	Downregulated
A_23_P134	0.002369	0.000396	-1.04	CSGALNACT	chondroitin sulfate N-acetylgalactosaminyltransferase 1	Downregulated
A_23_P350	0.002394	0.000402	-1.04	GLTSCR1L	GLTSCR1-like	Downregulated
A_33_P337	0.002472	0.000421	-1.04	RBM14-RBM4	RBM14-RBM4 readthrough	Downregulated
A_24_P119	0.00268	0.000477	-1.04	PHACTR4	phosphatase and actin regulator 4	Downregulated
A_33_P344	0.002912	0.000542	-1.04	OXR1	oxidation resistance 1	Downregulated
A_24_P247	0.003279	0.000653	-1.04	AK3	adenylate kinase 3	Downregulated
A_22_P000	0.003408	0.000691	-1.04	lnc-NT5E-1	lnc-NT5E-1:1	Downregulated
A_24_P885	0.003504	0.000718	-1.04	PEX11B	peroxisomal biogenesis factor 11 beta	Downregulated
A_33_P329	0.003719	0.000786	-1.04	WEE1	WEE1 G2 checkpoint kinase	Downregulated

A_23_P343	0.003983	0.000868	-1.04	ANGEL2	angel homolog 2 (Drosophila)	Downregulated
A_21_P001	0.004088	0.000899	-1.04	GK3P	glycerol kinase 3 pseudogene	Downregulated
A_23_P282	0.004106	0.000906	-1.04	CTDSP1	CTD (carboxy-terminal domain, RNA polymerase II, polypeptide A) small	Downregulated
A_22_P000	0.004114	0.000909	-1.04	lnc-PITPNC1-	lnc-PITPNC1-1:1	Downregulated
A_24_P250	0.004669	0.0011	-1.04	SNRPA	small nuclear ribonucleoprotein polypeptide A	Downregulated
A_33_P330	0.005091	0.00124	-1.04	LRRC39	leucine rich repeat containing 39	Downregulated
A_23_P490	0.005154	0.00127	-1.04	TMEM62	transmembrane protein 62	Downregulated
A_23_P881	0.005735	0.00148	-1.04	CINP	cyclin-dependent kinase 2 interacting protein	Downregulated
A_22_P000	0.005796	0.00151	-1.04	LOC1019279	uncharacterized LOC101927963	Downregulated
A_23_P117	0.005917	0.00155	-1.04	MTFMT	mitochondrial methionyl-tRNA formyltransferase	Downregulated
A_23_P487	0.006603	0.00182	-1.04	C14orf159	chromosome 14 open reading frame 159	Downregulated
A_33_P327	0.006715	0.00186	-1.04	TXNDC5	thioredoxin domain containing 5 (endoplasmic reticulum)	Downregulated
A_24_P390	0.006906	0.00193	-1.04	TRAPPC6A	trafficking protein particle complex 6A	Downregulated
A_21_P000	0.011269	0.00373	-1.04	LOC1027247	uncharacterized LOC102724758	Downregulated
A_22_P000	0.011812	0.00397	-1.04	lnc-C6orf221-	lnc-C6orf221-2:3	Downregulated
A_24_P194	0.014614	0.00523	-1.04	EHBP1	EH domain binding protein 1	Downregulated
A_21_P000	0.01848	0.00705	-1.04	lnc-BANP-1	lnc-BANP-1:4	Downregulated
A_22_P000	0.022463	0.00897	-1.04	LOC284009	uncharacterized LOC284009	Downregulated
A_33_P383	0.023436	0.00946	-1.04	HAUS2	HAUS augmin-like complex, subunit 2	Downregulated
A_32_P321	0.030488	0.0132	-1.04	WDFY4	WDFY family member 4	Downregulated
A_33_P335	0.031609	0.0138	-1.04	NHSL2	NHS-like 2	Downregulated
A_22_P000	0.036406	0.0164	-1.04	LOC1005068	uncharacterized LOC100506801	Downregulated
A_21_P001	0.046867	0.0222	-1.04	XLOC_l2_002033		Downregulated
A_21_P000	0.057717	0.0285	-1.04	LINC00908	long intergenic non-protein coding RNA 908	Downregulated
A_23_P541	0.060451	0.0301	-1.04	NIF3L1	NIF3 NGG1 interacting factor 3-like 1 (S. cerevisiae)	Downregulated
A_22_P000	0.060593	0.0302	-1.04	lnc-SETD1B-1	lnc-SETD1B-1:1	Downregulated
A_22_P000	0.066548	0.0338	-1.04	lnc-OCIAD2-1	lnc-OCIAD2-1:1	Downregulated
A_21_P000	0.072528	0.0374	-1.04	LOC645434	uncharacterized LOC645434	Downregulated
A_33_P341	0.002131	0.000337	-1.05	NUDT8	nudix (nucleoside diphosphate linked moiety X)-type motif 8	Downregulated
A_33_P339	0.002227	0.000361	-1.05	TNFSF9	tumor necrosis factor (ligand) superfamily, member 9	Downregulated
A_24_P673	0.002321	0.000383	-1.05	PIP4K2A	phosphatidylinositol-5-phosphate 4-kinase, type II, alpha	Downregulated
A_23_P201	0.002433	0.000411	-1.05	GUK1	guanylate kinase 1	Downregulated
A_24_P453	0.00255	0.000443	-1.05	NIPAL3	NIPA-like domain containing 3	Downregulated
A_23_P368	0.002563	0.000446	-1.05	AGPS	alkylglycerone phosphate synthase	Downregulated
A_23_P259	0.002712	0.000487	-1.05	EIF2B2	eukaryotic translation initiation factor 2B, subunit 2 beta, 39kDa	Downregulated
A_23_P117	0.003094	0.000596	-1.05	UBL7	ubiquitin-like 7	Downregulated
A_23_P133	0.003855	0.000829	-1.05	LSP1	lymphocyte-specific protein 1	Downregulated

A_33_P325	0.004778	0.00114	-1.05	SPTBN1	spectrin, beta, non-erythrocytic 1	Downregulated
A_21_P000	0.006003	0.00158	-1.05	SNORA71D	small nucleolar RNA, H/ACA box 71D	Downregulated
A_23_P136	0.006349	0.00172	-1.05	MAGEA6	melanoma antigen family A, 6	Downregulated
A_33_P322	0.006477	0.00177	-1.05	C16orf54	chromosome 16 open reading frame 54	Downregulated
A_24_P131	0.007614	0.00221	-1.05	ATP13A2	ATPase type 13A2	Downregulated
A_23_P422	0.008365	0.00252	-1.05	WDR63	WD repeat domain 63	Downregulated
A_22_P000	0.010093	0.00323	-1.05	IFNG-AS1	IFNG antisense RNA 1	Downregulated
A_23_P152	0.012697	0.00435	-1.05	B9D1	B9 protein domain 1	Downregulated
A_24_P410	0.012697	0.00435	-1.05	ROR1	receptor tyrosine kinase-like orphan receptor 1	Downregulated
A_21_P000	0.016548	0.00615	-1.05	lnc-ZNF680-1	lnc-ZNF680-1:1	Downregulated
A_23_P503	0.049204	0.0235	-1.05	NAT14	N-acetyltransferase 14 (GCN5-related, putative)	Downregulated
A_21_P001	0.055566	0.0272	-1.05	XLOC_l2_000138		Downregulated
A_22_P000	0.071529	0.0368	-1.05	LINC00670	long intergenic non-protein coding RNA 670	Downregulated
A_24_P386	0.002087	0.000324	-1.06	PPP1CC	protein phosphatase 1, catalytic subunit, gamma isozyme	Downregulated
A_23_P145	0.002124	0.000334	-1.06	S100A11	S100 calcium binding protein A11	Downregulated
A_21_P001	0.002124	0.000335	-1.06	LINC01347	long intergenic non-protein coding RNA 1347	Downregulated
A_23_P256	0.002241	0.000365	-1.06	RPA3	replication protein A3, 14kDa	Downregulated
A_32_P209	0.002423	0.000409	-1.06	MRPL46	mitochondrial ribosomal protein L46	Downregulated
A_21_P000	0.002427	0.00041	-1.06	lnc-DENND1A	lnc-DENND1A-1:1	Downregulated
A_33_P324	0.002454	0.000416	-1.06	VWA9	von Willebrand factor A domain containing 9	Downregulated
A_22_P000	0.002516	0.000434	-1.06	lnc-PDHX-1	lnc-PDHX-1:1	Downregulated
A_23_P806	0.002611	0.00046	-1.06	SETMAR	SET domain and mariner transposase fusion gene	Downregulated
A_21_P000	0.002669	0.000474	-1.06	lnc-CHL1-1	lnc-CHL1-1:1	Downregulated
A_23_P602	0.002719	0.000488	-1.06	GRHPR	glyoxylate reductase/hydroxypyruvate reductase	Downregulated
A_33_P323	0.002873	0.000531	-1.06	MIA3	melanoma inhibitory activity family, member 3	Downregulated
A_32_P155	0.003033	0.000578	-1.06	CD2AP	CD2-associated protein	Downregulated
A_23_P113	0.003094	0.000596	-1.06	C10orf11	chromosome 10 open reading frame 11	Downregulated
A_33_P323	0.003159	0.000617	-1.06	PYHIN1	pyrin and HIN domain family, member 1	Downregulated
A_21_P000	0.003283	0.000654	-1.06	lnc-SETD6-8	lnc-SETD6-8:1	Downregulated
A_33_P341	0.003529	0.000726	-1.06	MAP3K2	mitogen-activated protein kinase kinase kinase 2	Downregulated
A_23_P376	0.003535	0.000728	-1.06	PAPOLA	poly(A) polymerase alpha	Downregulated
A_33_P328	0.003893	0.000841	-1.06	DNM1L	dynamin 1-like	Downregulated
A_33_P332	0.004674	0.0011	-1.06	POLL	polymerase (DNA directed), lambda	Downregulated
A_22_P000	0.004658	0.0011	-1.06	TMEM231	transmembrane protein 231	Downregulated
A_33_P337	0.00501	0.00122	-1.06	SCIMP	SLP adaptor and CSK interacting membrane protein	Downregulated
A_33_P322	0.005296	0.00132	-1.06	LINC00656	long intergenic non-protein coding RNA 656	Downregulated
A_24_P225	0.007006	0.00197	-1.06	IRS1	insulin receptor substrate 1	Downregulated

A_33_P333	0.007949	0.00234	-1.06	ZXDB	zinc finger, X-linked, duplicated B	Downregulated
A_23_P140	0.009322	0.00291	-1.06	LMAN1L	lectin, mannose-binding, 1 like	Downregulated
A_33_P324	0.009413	0.00294	-1.06	RAD52	RAD52 homolog (S. cerevisiae)	Downregulated
A_23_P382	0.010757	0.00351	-1.06	BCKDK	branched chain ketoacid dehydrogenase kinase	Downregulated
A_33_P337	0.014378	0.00512	-1.06	SNAPC2	small nuclear RNA activating complex, polypeptide 2, 45kDa	Downregulated
A_22_P000	0.014597	0.00523	-1.06	CASC11	cancer susceptibility candidate 11 (non-protein coding)	Downregulated
A_33_P329	0.014597	0.00523	-1.06	FAM127C	family with sequence similarity 127, member C	Downregulated
A_22_P000	0.014597	0.00523	-1.06	lnc-AKAP10-1	lnc-AKAP10-1:1	Downregulated
A_21_P000	0.016221	0.00599	-1.06	lnc-ZNF608-5	lnc-ZNF608-5:1	Downregulated
A_23_P144	0.017139	0.00643	-1.06	RAD1	RAD1 checkpoint DNA exonuclease	Downregulated
A_23_P832	0.021971	0.00873	-1.06	AK8	adenylate kinase 8	Downregulated
A_22_P000	0.021998	0.00874	-1.06	lnc-GCM1-1	lnc-GCM1-1:1	Downregulated
A_21_P000	0.022121	0.0088	-1.06	LINC01586	long intergenic non-protein coding RNA 1586	Downregulated
A_33_P336	0.024852	0.0102	-1.06	SYT8	synaptotagmin VIII	Downregulated
A_21_P001	0.038507	0.0175	-1.06	lnc-FGF10-3	lnc-FGF10-3:3	Downregulated
A_23_P372	0.039011	0.0178	-1.06	CGRRF1	cell growth regulator with ring finger domain 1	Downregulated
A_23_P365	0.041105	0.019	-1.06	KCNG4	potassium channel, voltage gated modifier subfamily G, member 4	Downregulated
A_23_P903	0.002119	0.000333	-1.07	TMEM259	transmembrane protein 259	Downregulated
A_24_P583	0.002128	0.000337	-1.07	DCAKD	dephospho-CoA kinase domain containing	Downregulated
A_23_P243	0.002197	0.000353	-1.07	CCDC88B	coiled-coil domain containing 88B	Downregulated
A_24_P134	0.002219	0.000358	-1.07	MYADM	myeloid-associated differentiation marker	Downregulated
A_23_P309	0.002339	0.000388	-1.07	CYB5R4	cytochrome b5 reductase 4	Downregulated
A_21_P001	0.002995	0.000567	-1.07	XLOC_l2_015565		Downregulated
A_21_P001	0.003155	0.000615	-1.07	LOC1019286	uncharacterized LOC101928670	Downregulated
A_24_P153	0.003259	0.000647	-1.07	FGD3	FYVE, RhoGEF and PH domain containing 3	Downregulated
A_33_P329	0.003381	0.000682	-1.07	TET1	tet methylcytosine dioxygenase 1	Downregulated
A_23_P422	0.003648	0.000763	-1.07	CUL4B	cullin 4B	Downregulated
A_21_P001	0.003957	0.00086	-1.07	XLOC_l2_005874		Downregulated
A_23_P914	0.004131	0.000914	-1.07	RWDD2B	RWD domain containing 2B	Downregulated
A_24_P678	0.00431	0.000976	-1.07	MGEA5	meningioma expressed antigen 5 (hyaluronidase)	Downregulated
A_32_P148	0.004708	0.00111	-1.07	ANXA2	annexin A2	Downregulated
A_23_P314	0.004901	0.00118	-1.07	CHKB	choline kinase beta	Downregulated
A_23_P703	0.005095	0.00125	-1.07	ENPP4	ectonucleotide pyrophosphatase/phosphodiesterase 4 (putative)	Downregulated
A_33_P325	0.005114	0.00125	-1.07	TAB3	TGF-beta activated kinase 1/MAP3K7 binding protein 3	Downregulated
A_23_P170	0.005516	0.0014	-1.07	PACRG	PARK2 co-regulated	Downregulated
A_32_P226	0.005508	0.0014	-1.07	YWHAZ	tyrosine 3-monooxygenase/tryptophan 5-monooxygenase activation pro	Downregulated
A_21_P000	0.006094	0.00162	-1.07	DNAJC3-AS1	DNAJC3 antisense RNA 1 (head to head)	Downregulated

A_33_P338	0.006203	0.00166	-1.07	NUDT8	nudix (nucleoside diphosphate linked moiety X)-type motif 8	Downregulated
A_24_P345	0.006262	0.00168	-1.07	ANTXR2	anthrax toxin receptor 2	Downregulated
A_22_P000	0.007028	0.00198	-1.07	lnc-ADCK3-1	lnc-ADCK3-1:1	Downregulated
A_24_P608	0.007136	0.00203	-1.07	CHCHD10	coiled-coil-helix-coiled-coil-helix domain containing 10	Downregulated
A_21_P000	0.008574	0.0026	-1.07	lnc-C1QTNF1	lnc-C1QTNF1-1:1	Downregulated
A_23_P221	0.009285	0.00289	-1.07	PLEC	plectin	Downregulated
A_22_P000	0.012583	0.0043	-1.07	lnc-TEAD4-2	lnc-TEAD4-2:1	Downregulated
A_33_P328	0.016168	0.00596	-1.07	AAK1	AP2 associated kinase 1	Downregulated
A_23_P103	0.017467	0.00658	-1.07	OCLM	oculomedin	Downregulated
A_23_P371	0.053461	0.026	-1.07	PRKACB	protein kinase, cAMP-dependent, catalytic, beta	Downregulated
A_33_P329	0.057647	0.0285	-1.07	RAC3	ras-related C3 botulinum toxin substrate 3 (rho family, small GTP binding	Downregulated
A_22_P000	0.075234	0.0391	-1.07	lnc-PRL-2	lnc-PRL-2:1	Downregulated
A_24_P413	0.001974	0.000294	-1.08	TGOLN2	trans-golgi network protein 2	Downregulated
A_23_P143	0.002016	0.000305	-1.08	TIPARP	TCDD-inducible poly(ADP-ribose) polymerase	Downregulated
A_23_P156	0.002053	0.000315	-1.08	GOLPH3	golgi phosphoprotein 3 (coat-protein)	Downregulated
A_24_P934	0.002072	0.00032	-1.08	SYNGAP1	synaptic Ras GTPase activating protein 1	Downregulated
A_33_P335	0.002077	0.000321	-1.08	RIC1	RAB6A GEF complex partner 1	Downregulated
A_23_P164	0.0021	0.000326	-1.08	RFFL	ring finger and FYVE-like domain containing E3 ubiquitin protein ligase	Downregulated
A_22_P000	0.002114	0.000331	-1.08	lnc-GTPBP1-1	lnc-GTPBP1-1:1	Downregulated
A_23_P535	0.002128	0.000337	-1.08	CCDC53	coiled-coil domain containing 53	Downregulated
A_33_P335	0.002167	0.000346	-1.08	LRRFIP1	leucine rich repeat (in FLII) interacting protein 1	Downregulated
A_24_P801	0.002229	0.000361	-1.08	PTPN18	protein tyrosine phosphatase, non-receptor type 18 (brain-derived)	Downregulated
A_33_P341	0.002471	0.000421	-1.08	VEZT	vezatin, adherens junctions transmembrane protein	Downregulated
A_22_P000	0.002673	0.000475	-1.08	LOC1019274	uncharacterized LOC101927450	Downregulated
A_33_P339	0.003195	0.000628	-1.08	SYS1	Sys1 golgi trafficking protein	Downregulated
A_23_P688	0.003312	0.000663	-1.08	TXN2	thioredoxin 2	Downregulated
A_23_P137	0.0045	0.00104	-1.08	NENF	neudesin neurotrophic factor	Downregulated
A_23_P100	0.005121	0.00126	-1.08	STAT3	signal transducer and activator of transcription 3 (acute-phase response	Downregulated
A_33_P327	0.005568	0.00142	-1.08	ZC3H11A	zinc finger CCCH-type containing 11A	Downregulated
A_24_P345	0.006688	0.00185	-1.08	ING5	inhibitor of growth family, member 5	Downregulated
A_33_P325	0.007492	0.00216	-1.08	TP53TG1	TP53 target 1 (non-protein coding)	Downregulated
A_22_P000	0.00906	0.0028	-1.08	lnc-PLCL2-1	lnc-PLCL2-1:1	Downregulated
A_33_P327	0.00906	0.0028	-1.08	TMEM236	transmembrane protein 236	Downregulated
A_24_P148	0.009631	0.00304	-1.08	RUVBL1	RuvB-like AAA ATPase 1	Downregulated
A_22_P000	0.010291	0.00332	-1.08	lnc-KIN-1	lnc-KIN-1:1	Downregulated
A_33_P360	0.013178	0.00456	-1.08	ILF3-AS1	ILF3 antisense RNA 1 (head to head)	Downregulated
A_33_P335	0.015069	0.00545	-1.08	FAM198A	family with sequence similarity 198, member A	Downregulated

A_33_P339	0.023121	0.0093	-1.08	LINC01296	long intergenic non-protein coding RNA 1296	Downregulated
A_32_P855	0.029515	0.0126	-1.08	HCFC2	host cell factor C2	Downregulated
A_33_P336	0.038334	0.0174	-1.08	FAM205BP	transmembrane protein C9orf144B pseudogene	Downregulated
A_33_P323	0.038334	0.0174	-1.08	KCNA2	potassium channel, voltage gated shaker related subfamily A, member 2	Downregulated
A_22_P000	0.038334	0.0174	-1.08	LOC285889	uncharacterized LOC285889	Downregulated
A_24_P321	0.044736	0.021	-1.08	SLC38A4	solute carrier family 38, member 4	Downregulated
A_23_P726	0.045917	0.0217	-1.08	GPIHBP1	glycosylphosphatidylinositol anchored high density lipoprotein binding p	Downregulated
A_33_P329	0.062694	0.0315	-1.08	LMOD1	leiomodin 1 (smooth muscle)	Downregulated
A_22_P000	0.062694	0.0315	-1.08	lnc-UBFD1-1	lnc-UBFD1-1:1	Downregulated
A_21_P000	0.06874	0.0351	-1.08	LOC1019276	uncharacterized LOC101927664	Downregulated
A_21_P000	0.069545	0.0356	-1.08	lnc-STIL-3	lnc-STIL-3:1	Downregulated
A_24_P185	0.001942	0.000284	-1.09	GSK3A	glycogen synthase kinase 3 alpha	Downregulated
A_23_P254	0.002021	0.000306	-1.09	RANGRF	RAN guanine nucleotide release factor	Downregulated
A_24_P671	0.002046	0.000314	-1.09	SON	SON DNA binding protein	Downregulated
A_33_P330	0.002166	0.000345	-1.09	ATF6B	activating transcription factor 6 beta	Downregulated
A_23_P754	0.002638	0.000466	-1.09	NUDT22	nudix (nucleoside diphosphate linked moiety X)-type motif 22	Downregulated
A_24_P934	0.002949	0.000552	-1.09	MTCH2	mitochondrial carrier 2	Downregulated
A_24_P497	0.00301	0.000571	-1.09	IRF2BP2	interferon regulatory factor 2 binding protein 2	Downregulated
A_33_P333	0.003041	0.000581	-1.09	XLOC_l2_015508		Downregulated
A_23_P172	0.003177	0.000623	-1.09	IAH1	isoamyl acetate-hydrolyzing esterase 1 homolog (S. cerevisiae)	Downregulated
A_22_P000	0.003287	0.000655	-1.09	SLC25A29	solute carrier family 25 (mitochondrial carnitine/acylcarnitine carrier), men	Downregulated
A_21_P001	0.004632	0.00109	-1.09	LOC1005055	uncharacterized LOC100505501	Downregulated
A_24_P211	0.005114	0.00125	-1.09	ICA1L	islet cell autoantigen 1,69kDa-like	Downregulated
A_21_P000	0.005184	0.00128	-1.09	SNORD71	small nucleolar RNA, C/D box 71	Downregulated
A_32_P286	0.005221	0.00129	-1.09	SNRPA1	small nuclear ribonucleoprotein polypeptide A'	Downregulated
A_22_P000	0.005706	0.00147	-1.09	LINC01402	long intergenic non-protein coding RNA 1402	Downregulated
A_33_P384	0.006428	0.00175	-1.09	IKZF1	IKAROS family zinc finger 1 (Ikaros)	Downregulated
A_23_P149	0.013177	0.00456	-1.09	EFCAB2	EF-hand calcium binding domain 2	Downregulated
A_21_P001	0.013653	0.00477	-1.09	LINC00665	long intergenic non-protein coding RNA 665	Downregulated
A_33_P348	0.013647	0.00477	-1.09	PTPN20B	protein tyrosine phosphatase, non-receptor type 20B	Downregulated
A_21_P000	0.014643	0.00525	-1.09	lnc-GINS2-3	lnc-GINS2-3:1	Downregulated
A_23_P218	0.024384	0.00997	-1.09	RBM26	RNA binding motif protein 26	Downregulated
A_33_P321	0.031749	0.0138	-1.09	GPATCH4	G patch domain containing 4	Downregulated
A_33_P341	0.041211	0.019	-1.09	ZRSR2	zinc finger (CCCH type), RNA-binding motif and serine/arginine rich 2	Downregulated
A_23_P139	0.045974	0.0217	-1.09	UBAC2	UBA domain containing 2	Downregulated
A_22_P000	0.04728	0.0224	-1.09	lnc-PRR15L-4	lnc-PRR15L-4:1	Downregulated
A_23_P474	0.084486	0.0451	-1.09	ACAD8	acyl-CoA dehydrogenase family, member 8	Downregulated

A_33_P326	0.084486	0.0451	-1.09	GOLGA6L2	golgin A6 family-like 2	Downregulated
A_23_P196	0.084486	0.0451	-1.09	LRP11	low density lipoprotein receptor-related protein 11	Downregulated
A_21_P001	0.001986	0.000297	-1.1	XLOC_l2_009539		Downregulated
A_24_P361	0.002022	0.000306	-1.1	NDUFA9	NADH dehydrogenase (ubiquinone) 1 alpha subcomplex, 9, 39kDa	Downregulated
A_24_P183	0.002124	0.000335	-1.1	PLAC8	placenta-specific 8	Downregulated
A_22_P000	0.00216	0.000344	-1.1	lnc-SETDB1-1	lnc-SETDB1-1:1	Downregulated
A_23_P208	0.002572	0.00045	-1.1	SEC11C	SEC11 homolog C (S. cerevisiae)	Downregulated
A_21_P001	0.002689	0.00048	-1.1	LINC00339	long intergenic non-protein coding RNA 339	Downregulated
A_23_P166	0.002712	0.000486	-1.1	DCP1A	decapping mRNA 1A	Downregulated
A_33_P328	0.003796	0.000809	-1.1	LINC01534	long intergenic non-protein coding RNA 1534	Downregulated
A_24_P316	0.004182	0.000932	-1.1	NDUFA10	NADH dehydrogenase (ubiquinone) 1 alpha subcomplex, 10, 42kDa	Downregulated
A_21_P000	0.004318	0.00098	-1.1	C2orf48	chromosome 2 open reading frame 48	Downregulated
A_22_P000	0.004324	0.000982	-1.1	lnc-NPAT-2	lnc-NPAT-2:1	Downregulated
A_23_P414	0.004432	0.00102	-1.1	DDX60	DEAD (Asp-Glu-Ala-Asp) box polypeptide 60	Downregulated
A_33_P329	0.004864	0.00117	-1.1	ARID1B	AT rich interactive domain 1B (SWI1-like)	Downregulated
A_33_P321	0.005004	0.00121	-1.1	TM9SF1	transmembrane 9 superfamily member 1	Downregulated
A_21_P001	0.005562	0.00142	-1.1	MIR4435-1HC	MIR4435-1 host gene (non-protein coding)	Downregulated
A_23_P160	0.006528	0.00179	-1.1	UAP1	UDP-N-acetylglucosamine pyrophosphorylase 1	Downregulated
A_21_P001	0.007287	0.00208	-1.1	XLOC_l2_005438		Downregulated
A_33_P342	0.009679	0.00306	-1.1	FXYD5	FXYD domain containing ion transport regulator 5	Downregulated
A_23_P649	0.011268	0.00373	-1.1	TNRC6B	trinucleotide repeat containing 6B	Downregulated
A_24_P194	0.011813	0.00397	-1.1	SMEK2	SMEK homolog 2, suppressor of mek1 (Dictyostelium)	Downregulated
A_24_P139	0.017407	0.00655	-1.1	USP25	ubiquitin specific peptidase 25	Downregulated
A_21_P000	0.019522	0.00755	-1.1	lnc-KATNAL2	lnc-KATNAL2-4:1	Downregulated
A_23_P256	0.027577	0.0116	-1.1	RDH11	retinol dehydrogenase 11 (all-trans/9-cis/11-cis)	Downregulated
A_23_P161	0.040057	0.0184	-1.1	HPX	hemopexin	Downregulated
A_32_P922	0.071673	0.0369	-1.1	PMS2P9	postmeiotic segregation increased 2 pseudogene 9	Downregulated
A_23_P526	0.001866	0.000266	-1.11	COX8A	cytochrome c oxidase subunit VIIIA (ubiquitous)	Downregulated
A_23_P420	0.001933	0.000282	-1.11	BRK1	BRICK1, SCAR/WAVE actin-nucleating complex subunit	Downregulated
A_22_P000	0.002145	0.00034	-1.11	lnc-VN1R2-1	lnc-VN1R2-1:4	Downregulated
A_23_P371	0.002326	0.000385	-1.11	ICOS	inducible T-cell co-stimulator	Downregulated
A_24_P174	0.002375	0.000397	-1.11	FBXW7	F-box and WD repeat domain containing 7, E3 ubiquitin protein ligase	Downregulated
A_33_P339	0.002429	0.00041	-1.11	DIXDC1	DIX domain containing 1	Downregulated
A_24_P241	0.002457	0.000417	-1.11	JUNB	jun B proto-oncogene	Downregulated
A_33_P329	0.002517	0.000434	-1.11	PRKCB	protein kinase C, beta	Downregulated
A_23_P213	0.003001	0.000568	-1.11	SMARCAD1	SWI/SNF-related, matrix-associated actin-dependent regulator of chroma	Downregulated
A_33_P322	0.003033	0.000578	-1.11	SCIMP	SLP adaptor and CSK interacting membrane protein	Downregulated

A_33_P333	0.003423	0.000695	-1.11	PLEKHA2	pleckstrin homology domain containing, family A (phosphoinositide bindi	Downregulated
A_33_P341	0.003929	0.000851	-1.11	GLIPR1	GLI pathogenesis-related 1	Downregulated
A_21_P001	0.004341	0.000989	-1.11	XLOC_l2_007167		Downregulated
A_22_P000	0.005709	0.00147	-1.11	lnc-SLC39A1(	lnc-SLC39A10-3:1	Downregulated
A_32_P167	0.006586	0.00181	-1.11	MED14OS	MED14 opposite strand	Downregulated
A_24_P107	0.006885	0.00192	-1.11	USP2	ubiquitin specific peptidase 2	Downregulated
A_33_P338	0.006988	0.00197	-1.11	C5orf56	chromosome 5 open reading frame 56	Downregulated
A_33_P337	0.008925	0.00274	-1.11	lnc-FCGR3B-2	lnc-FCGR3B-2:1	Downregulated
A_21_P000	0.015117	0.00547	-1.11	lnc-KCND3-1	lnc-KCND3-1:1	Downregulated
A_23_P138	0.01924	0.00741	-1.11	KAT5	K(lysine) acetyltransferase 5	Downregulated
A_33_P326	0.023072	0.00927	-1.11	PCK2	phosphoenolpyruvate carboxykinase 2 (mitochondrial)	Downregulated
A_22_P000	0.031142	0.0135	-1.11	LOC1027245	uncharacterized LOC102724598	Downregulated
A_21_P000	0.033859	0.015	-1.11	lnc-RAMP3-2	lnc-RAMP3-2:1	Downregulated
A_23_P302	0.038195	0.0174	-1.11	PCDHB16	protocadherin beta 16	Downregulated
A_24_P883	0.048785	0.0233	-1.11	C2orf49	chromosome 2 open reading frame 49	Downregulated
A_23_P380	0.065461	0.0331	-1.11	NAGPA	N-acetylglucosamine-1-phosphodiester alpha-N-acetylglucosaminidase	Downregulated
A_21_P000	0.070008	0.0359	-1.11	LOC1027249	uncharacterized LOC102724943	Downregulated
A_23_P106	0.072703	0.0375	-1.11	PSEN1	presenilin 1	Downregulated
A_24_P418	0.089223	0.0483	-1.11	ARHGAP6	Rho GTPase activating protein 6	Downregulated
A_33_P343	0.002006	0.000302	-1.12	ZC3H14	zinc finger CCCH-type containing 14	Downregulated
A_23_P124	0.002154	0.000342	-1.12	MED24	mediator complex subunit 24	Downregulated
A_24_P166	0.002397	0.000403	-1.12	TMEM60	transmembrane protein 60	Downregulated
A_23_P159	0.002515	0.000434	-1.12	PHKA2	phosphorylase kinase, alpha 2 (liver)	Downregulated
A_23_P168	0.002823	0.000516	-1.12	TXNDC5	thioredoxin domain containing 5 (endoplasmic reticulum)	Downregulated
A_19_P003	0.005578	0.00143	-1.12	H19	H19, imprinted maternally expressed transcript (non-protein coding)	Downregulated
A_23_P361	0.00588	0.00154	-1.12	ATF7	activating transcription factor 7	Downregulated
A_33_P324	0.005942	0.00156	-1.12	LTB	lymphotoxin beta (TNF superfamily, member 3)	Downregulated
A_24_P289	0.006203	0.00166	-1.12	TFF3	trefoil factor 3 (intestinal)	Downregulated
A_22_P000	0.006435	0.00175	-1.12	lnc-AIPL1-2	lnc-AIPL1-2:3	Downregulated
A_33_P363	0.007013	0.00198	-1.12	UBE2D4	ubiquitin-conjugating enzyme E2D 4 (putative)	Downregulated
A_22_P000	0.008876	0.00272	-1.12	lnc-DLX2-2	lnc-DLX2-2:1	Downregulated
A_22_P000	0.009998	0.00319	-1.12	lnc-ALPK3-1	lnc-ALPK3-1:1	Downregulated
A_21_P000	0.014945	0.00539	-1.12	SNORA19	small nucleolar RNA, H/ACA box 19	Downregulated
A_33_P324	0.016327	0.00605	-1.12	NEFM	neurofilament, medium polypeptide	Downregulated
A_24_P362	0.016327	0.00605	-1.12	PAX3	paired box 3	Downregulated
A_23_P257	0.021938	0.00872	-1.12	NUP214	nucleoporin 214kDa	Downregulated
A_33_P337	0.023875	0.0097	-1.12	TMEM106B	transmembrane protein 106B	Downregulated

A_24_P202	0.025367	0.0105	-1.12	METTL9	methyltransferase like 9	Downregulated
A_21_P001	0.02671	0.0112	-1.12	XLOC_l2_011627		Downregulated
A_22_P000	0.027602	0.0116	-1.12	lnc-EIF2AK3-	lnc-EIF2AK3-4:2	Downregulated
A_21_P001	0.029687	0.0127	-1.12	LINC-PINT	long intergenic non-protein coding RNA, p53 induced transcript	Downregulated
A_33_P321	0.030226	0.013	-1.12	ELF2	E74-like factor 2 (ets domain transcription factor)	Downregulated
A_21_P000	0.034154	0.0151	-1.12	lnc-RGMA-9	lnc-RGMA-9:2	Downregulated
A_22_P000	0.035468	0.0159	-1.12	DISC1-IT1	DISC1 intronic transcript 1 (non-protein coding)	Downregulated
A_22_P000	0.036418	0.0164	-1.12	LOC1027247	uncharacterized LOC102724776	Downregulated
A_32_P967	0.045156	0.0213	-1.12	SHCBP1	SHC SH2-domain binding protein 1	Downregulated
A_22_P000	0.050103	0.0241	-1.12	LINC01021	long intergenic non-protein coding RNA 1021	Downregulated
A_24_P217	0.050382	0.0242	-1.12	RAD9A	RAD9 homolog A (S. pombe)	Downregulated
A_22_P000	0.069343	0.0354	-1.12	lnc-ATP6V1E	lnc-ATP6V1E2-3:1	Downregulated
A_23_P134	0.074516	0.0386	-1.12	COL26A1	collagen, type XXVI, alpha 1	Downregulated
A_22_P000	0.079443	0.0418	-1.12	lnc-RTN3-1	lnc-RTN3-1:1	Downregulated
A_32_P952	0.001821	0.000255	-1.13	FDPSP2	farnesyl diphosphate synthase pseudogene 2	Downregulated
A_23_P325	0.001845	0.000261	-1.13	SKI	SKI proto-oncogene	Downregulated
A_22_P000	0.001901	0.000274	-1.13	TMEM258	transmembrane protein 258	Downregulated
A_33_P340	0.001958	0.000289	-1.13	VAMP2	vesicle-associated membrane protein 2 (synaptobrevin 2)	Downregulated
A_33_P341	0.002203	0.000355	-1.13	DNAJC5	DnaJ (Hsp40) homolog, subfamily C, member 5	Downregulated
A_23_P346	0.002241	0.000365	-1.13	MTOR	mechanistic target of rapamycin (serine/threonine kinase)	Downregulated
A_33_P341	0.002336	0.000387	-1.13	LOC645645	uncharacterized LOC645645	Downregulated
A_23_P156	0.002764	0.000501	-1.13	UQCC2	ubiquinol-cytochrome c reductase complex assembly factor 2	Downregulated
A_33_P324	0.002803	0.000511	-1.13	MAP3K8	mitogen-activated protein kinase kinase kinase 8	Downregulated
A_33_P385	0.002832	0.000519	-1.13	KIAA0319L	KIAA0319-like	Downregulated
A_23_P102	0.003511	0.00072	-1.13	PDE6D	phosphodiesterase 6D, cGMP-specific, rod, delta	Downregulated
A_21_P000	0.003533	0.000728	-1.13	lnc-SGCG-5	lnc-SGCG-5:3	Downregulated
A_24_P142	0.003721	0.000786	-1.13	AKAP13	A kinase (PRKA) anchor protein 13	Downregulated
A_21_P000	0.003789	0.000806	-1.13	lnc-AC09955	lnc-AC099552.4.1-1:2	Downregulated
A_22_P000	0.004411	0.00101	-1.13	lnc-TMEM85-	lnc-TMEM85-1:1	Downregulated
A_23_P118	0.005231	0.0013	-1.13	SLFN12	schlafen family member 12	Downregulated
A_23_P490	0.005268	0.00131	-1.13	WDR61	WD repeat domain 61	Downregulated
A_23_P397	0.006108	0.00162	-1.13	INIP	INTS3 and NABP interacting protein	Downregulated
A_23_P942	0.006365	0.00172	-1.13	LONRF1	LON peptidase N-terminal domain and ring finger 1	Downregulated
A_23_P551	0.007225	0.00206	-1.13	RANGRF	RAN guanine nucleotide release factor	Downregulated
A_22_P000	0.007332	0.0021	-1.13	LOC1001310	uncharacterized LOC100131047	Downregulated
A_23_P383	0.007698	0.00224	-1.13	CD300LG	CD300 molecule-like family member g	Downregulated
A_22_P000	0.008796	0.00269	-1.13	lnc-TMX3-1	lnc-TMX3-1:1	Downregulated

A_23_P409	0.008796	0.00269	-1.13	PPFIBP2	PTPRF interacting protein, binding protein 2 (liprin beta 2)	Downregulated
A_32_P957	0.009012	0.00278	-1.13	FANCI	Fanconi anemia, complementation group I	Downregulated
A_23_P206	0.014984	0.00541	-1.13	NDE1	nudE neurodevelopment protein 1	Downregulated
A_33_P339	0.0158	0.00578	-1.13	C10orf99	chromosome 10 open reading frame 99	Downregulated
A_23_P377	0.016556	0.00615	-1.13	FHOD1	formin homology 2 domain containing 1	Downregulated
A_21_P000	0.024386	0.00997	-1.13	lnc-C16orf72-	lnc-C16orf72-5:1	Downregulated
A_23_P129	0.026911	0.0113	-1.13	ATF7IP2	activating transcription factor 7 interacting protein 2	Downregulated
A_22_P000	0.030866	0.0134	-1.13	lnc-ALDH9A1	lnc-ALDH9A1-1:1	Downregulated
A_24_P131	0.030866	0.0134	-1.13	MYL3	myosin, light chain 3, alkali; ventricular, skeletal, slow	Downregulated
A_23_P423	0.037331	0.0169	-1.13	PHC2	polyhomeotic homolog 2 (Drosophila)	Downregulated
A_23_P113	0.045493	0.0214	-1.13	P4HTM	prolyl 4-hydroxylase, transmembrane (endoplasmic reticulum)	Downregulated
A_22_P000	0.060062	0.0299	-1.13	lnc-TMCO3-2	lnc-TMCO3-2:1	Downregulated
A_21_P000	0.063206	0.0318	-1.13	lnc-MAT2B-3	lnc-MAT2B-3:15	Downregulated
A_24_P237	0.088541	0.0478	-1.13	NCOR1	nuclear receptor corepressor 1	Downregulated
A_21_P000	0.091508	0.0498	-1.13	lnc-CACNG3-	lnc-CACNG3-1:4	Downregulated
A_23_P255	0.001962	0.000291	-1.14	GPR183	G protein-coupled receptor 183	Downregulated
A_32_P402	0.001972	0.000293	-1.14	TMEM200A	transmembrane protein 200A	Downregulated
A_24_P350	0.00206	0.000317	-1.14	PRRC2B	proline-rich coiled-coil 2B	Downregulated
A_23_P133	0.002167	0.000346	-1.14	CCDC167	coiled-coil domain containing 167	Downregulated
A_19_P003	0.002241	0.000365	-1.14	ATP2C1	ATPase, Ca++ transporting, type 2C, member 1	Downregulated
A_23_P475	0.002243	0.000366	-1.14	TMEM258	transmembrane protein 258	Downregulated
A_33_P329	0.002476	0.000422	-1.14	FAM204A	family with sequence similarity 204, member A	Downregulated
A_33_P329	0.002503	0.000431	-1.14	MVP	major vault protein	Downregulated
A_22_P000	0.002503	0.000431	-1.14	PDE10A	phosphodiesterase 10A	Downregulated
A_23_P303	0.002952	0.000553	-1.14	TAS2R45	taste receptor, type 2, member 45	Downregulated
A_21_P001	0.003132	0.000608	-1.14	XLOC_l2_006789		Downregulated
A_23_P253	0.003189	0.000626	-1.14	CD99L2	CD99 molecule-like 2	Downregulated
A_23_P591	0.003363	0.000678	-1.14	POU5F1	POU class 5 homeobox 1	Downregulated
A_21_P000	0.005126	0.00126	-1.14	lnc-ZBTB17-2	lnc-ZBTB17-2:1	Downregulated
A_23_P118	0.005191	0.00128	-1.14	EXTL2	exostosin-like glycosyltransferase 2	Downregulated
A_21_P001	0.005196	0.00128	-1.14	LOC1001301	uncharacterized LOC100130156	Downregulated
A_23_P879	0.006336	0.00171	-1.14	DYRK4	dual-specificity tyrosine-(Y)-phosphorylation regulated kinase 4	Downregulated
A_22_P000	0.009281	0.00289	-1.14	LOC1019288	uncharacterized LOC101928812	Downregulated
A_33_P388	0.009391	0.00293	-1.14	POT1	protection of telomeres 1	Downregulated
A_22_P000	0.009912	0.00316	-1.14	lnc-CLEC3A-4	lnc-CLEC3A-4:1	Downregulated
A_23_P107	0.010132	0.00326	-1.14	RAB5C	RAB5C, member RAS oncogene family	Downregulated
A_23_P413	0.015334	0.00557	-1.14	P2RX5	purinergic receptor P2X, ligand gated ion channel, 5	Downregulated

A_23_P146	0.020326	0.00792	-1.14	CORO2A	coronin, actin binding protein, 2A	Downregulated
A_33_P331	0.021337	0.00842	-1.14	TBCB	tubulin folding cofactor B	Downregulated
A_33_P336	0.022942	0.00921	-1.14	NFYC	nuclear transcription factor Y, gamma	Downregulated
A_21_P000	0.028614	0.0122	-1.14	LINC01229	long intergenic non-protein coding RNA 1229	Downregulated
A_23_P129	0.045011	0.0212	-1.14	VPS9D1	VPS9 domain containing 1	Downregulated
A_24_P608	0.045613	0.0215	-1.14	ACHE	acetylcholinesterase (Yt blood group)	Downregulated
A_23_P212	0.060616	0.0302	-1.14	VPRBP	Vpr (HIV-1) binding protein	Downregulated
A_23_P211	0.083962	0.0447	-1.14	LINC01547	long intergenic non-protein coding RNA 1547	Downregulated
A_33_P342	0.001815	0.000253	-1.15	SON	SON DNA binding protein	Downregulated
A_22_P000	0.001858	0.000264	-1.15	lnc-YIF1A-1	lnc-YIF1A-1:2	Downregulated
A_33_P341	0.001903	0.000274	-1.15	ACLY	ATP citrate lyase	Downregulated
A_23_P204	0.001923	0.000279	-1.15	CDKN1B	cyclin-dependent kinase inhibitor 1B (p27, Kip1)	Downregulated
A_24_P394	0.001956	0.000289	-1.15	SHISA5	shisa family member 5	Downregulated
A_21_P000	0.001962	0.000291	-1.15	lnc-TRAK1-1	lnc-TRAK1-1:1	Downregulated
A_32_P129	0.002142	0.000339	-1.15	RAB1B	RAB1B, member RAS oncogene family	Downregulated
A_24_P926	0.00216	0.000344	-1.15	UBE2D3	ubiquitin-conjugating enzyme E2D 3	Downregulated
A_33_P325	0.002309	0.000381	-1.15	SNX17	sorting nexin 17	Downregulated
A_23_P331	0.002476	0.000423	-1.15	HIATL1	hippocampus abundant transcript-like 1	Downregulated
A_21_P001	0.002645	0.000468	-1.15	ZNF767P	zinc finger family member 767, pseudogene	Downregulated
A_24_P328	0.002989	0.000564	-1.15	YIPF5	Yip1 domain family, member 5	Downregulated
A_23_P264	0.00308	0.000591	-1.15	RHBDL1	rhomboid, veinlet-like 1 (Drosophila)	Downregulated
A_22_P000	0.003286	0.000655	-1.15	RPARP-AS1	RPARP antisense RNA 1	Downregulated
A_23_P382	0.00348	0.000712	-1.15	OR1F2P	olfactory receptor, family 1, subfamily F, member 2	Downregulated
A_23_P403	0.00379	0.000806	-1.15	DZANK1	double zinc ribbon and ankyrin repeat domains 1	Downregulated
A_23_P229	0.004003	0.000874	-1.15	SLC30A7	solute carrier family 30 (zinc transporter), member 7	Downregulated
A_24_P287	0.004119	0.00091	-1.15	RSAD2	radical S-adenosyl methionine domain containing 2	Downregulated
A_32_P959	0.005191	0.00128	-1.15	CLEC2D	C-type lectin domain family 2, member D	Downregulated
A_33_P328	0.005238	0.0013	-1.15	PYGO2	pygopus family PHD finger 2	Downregulated
A_19_P003	0.00537	0.00135	-1.15	lnc-GGCT-1	lnc-GGCT-1:14	Downregulated
A_22_P000	0.009013	0.00278	-1.15	LOC1005062	uncharacterized LOC100506274	Downregulated
A_24_P372	0.010267	0.00331	-1.15	EDRF1	erythroid differentiation regulatory factor 1	Downregulated
A_23_P105	0.017082	0.0064	-1.15	MDM1	Mdm1 nuclear protein homolog (mouse)	Downregulated
A_33_P324	0.018994	0.00729	-1.15	ZNF680	zinc finger protein 680	Downregulated
A_21_P001	0.026876	0.0113	-1.15	TRAK1	trafficking protein, kinesin binding 1	Downregulated
A_33_P334	0.027505	0.0116	-1.15	ZNF568	zinc finger protein 568	Downregulated
A_23_P807	0.028781	0.0123	-1.15	PLCD1	phospholipase C, delta 1	Downregulated
A_21_P001	0.029432	0.0126	-1.15	LOC1019282	uncharacterized LOC101928203	Downregulated

A_21_P000	0.029561	0.0127	-1.15	lnc-TSSC4-2	lnc-TSSC4-2:1	Downregulated
A_33_P341	0.031218	0.0136	-1.15	CHAF1A	chromatin assembly factor 1, subunit A (p150)	Downregulated
A_22_P000	0.036218	0.0163	-1.15	lnc-CETP-1	lnc-CETP-1:1	Downregulated
A_23_P862	0.042305	0.0196	-1.15	PIGC	phosphatidylinositol glycan anchor biosynthesis, class C	Downregulated
A_33_P326	0.049406	0.0237	-1.15	CPTP	ceramide-1-phosphate transfer protein	Downregulated
A_21_P001	0.050068	0.024	-1.15	ZNF37BP	zinc finger protein 37B, pseudogene	Downregulated
A_33_P340	0.073132	0.0378	-1.15	DEFB108B	defensin, beta 108B	Downregulated
A_24_P120	0.075523	0.0393	-1.15	GADD45G	growth arrest and DNA-damage-inducible, gamma	Downregulated
A_23_P118	0.082713	0.0439	-1.15	ATP2B4	ATPase, Ca++ transporting, plasma membrane 4	Downregulated
A_33_P340	0.001959	0.00029	-1.16	SELL	selectin L	Downregulated
A_33_P337	0.002053	0.000315	-1.16	LYPLA2	lysophospholipase II	Downregulated
A_23_P869	0.00219	0.000352	-1.16	FADD	Fas (TNFRSF6)-associated via death domain	Downregulated
A_21_P000	0.00241	0.000406	-1.16	SNORD22	small nucleolar RNA, C/D box 22	Downregulated
A_22_P000	0.002572	0.00045	-1.16	LOC1005070	uncharacterized LOC100507006	Downregulated
A_22_P000	0.002921	0.000544	-1.16	lnc-CCDC68-2	lnc-CCDC68-2:1	Downregulated
A_23_P115	0.003103	0.0006	-1.16	FUCA1	fucosidase, alpha-L- 1, tissue	Downregulated
A_33_P326	0.004804	0.00114	-1.16	PCBP4	poly(rC) binding protein 4	Downregulated
A_23_P406	0.008025	0.00237	-1.16	CAMSAP2	calmodulin regulated spectrin-associated protein family, member 2	Downregulated
A_23_P347	0.011448	0.00381	-1.16	TFCP2	transcription factor CP2	Downregulated
A_32_P175	0.01785	0.00675	-1.16	RCN2	reticulocalbin 2, EF-hand calcium binding domain	Downregulated
A_21_P001	0.021111	0.0083	-1.16	XLOC_l2_009139		Downregulated
A_33_P327	0.02899	0.0124	-1.16	NUP35	nucleoporin 35kDa	Downregulated
A_21_P000	0.045082	0.0212	-1.16	IL10RB-AS1	IL10RB antisense RNA 1 (head to head)	Downregulated
A_23_P154	0.051891	0.0251	-1.16	AOX1	aldehyde oxidase 1	Downregulated
A_21_P001	0.055447	0.0272	-1.16	XLOC_l2_012806		Downregulated
A_21_P000	0.057473	0.0284	-1.16	RNASEH1-AS	RNASEH1 antisense RNA 1	Downregulated
A_23_P102	0.061663	0.0309	-1.16	TXNRD2	thioredoxin reductase 2	Downregulated
A_32_P389	0.089753	0.0486	-1.16	HEATR5B	HEAT repeat containing 5B	Downregulated
A_33_P339	0.001684	0.000221	-1.17	MBNL2	muscleblind-like splicing regulator 2	Downregulated
A_33_P333	0.001753	0.000237	-1.17	ARID1B	AT rich interactive domain 1B (SWI1-like)	Downregulated
A_23_P208	0.001753	0.000237	-1.17	NDUFA3	NADH dehydrogenase (ubiquinone) 1 alpha subcomplex, 3, 9kDa	Downregulated
A_33_P322	0.00178	0.000244	-1.17	NCF1B	neutrophil cytosolic factor 1B pseudogene	Downregulated
A_19_P008	0.001791	0.000247	-1.17	BRK1	BRICK1, SCAR/WAVE actin-nucleating complex subunit	Downregulated
A_23_P170	0.001805	0.00025	-1.17	ANTXR2	anthrax toxin receptor 2	Downregulated
A_33_P330	0.001824	0.000255	-1.17	LOC1001291	uncharacterized LOC100129129	Downregulated
A_33_P328	0.001849	0.000262	-1.17	POMGNT1	protein O-linked mannose N-acetylglucosaminyltransferase 1 (beta 1,2-)	Downregulated
A_22_P000	0.001876	0.000268	-1.17	NAPSB	napsin B aspartic peptidase, pseudogene	Downregulated

A_23_P930	0.001916	0.000277	-1.17	SRP19	signal recognition particle 19kDa	Downregulated
A_33_P332	0.001992	0.000298	-1.17	ATP7A	ATPase, Cu++ transporting, alpha polypeptide	Downregulated
A_23_P214	0.001995	0.000299	-1.17	FLOT1	flotillin 1	Downregulated
A_24_P997	0.00204	0.000312	-1.17	ISOC2	isochorismatase domain containing 2	Downregulated
A_22_P000	0.00206	0.000317	-1.17	lnc-CNPY3-1	lnc-CNPY3-1:1	Downregulated
A_23_P372	0.002303	0.000379	-1.17	AHSA2	AHA1, activator of heat shock 90kDa protein ATPase homolog 2 (yeast	Downregulated
A_23_P162	0.002625	0.000463	-1.17	CNOT2	CCR4-NOT transcription complex, subunit 2	Downregulated
A_22_P000	0.002659	0.000472	-1.17	lnc-VN1R2-1	lnc-VN1R2-1:1	Downregulated
A_23_P409	0.002775	0.000504	-1.17	HNRNPUL2	heterogeneous nuclear ribonucleoprotein U-like 2	Downregulated
A_23_P349	0.003046	0.000582	-1.17	ATF3	activating transcription factor 3	Downregulated
A_33_P329	0.003964	0.000862	-1.17	LMO4	LIM domain only 4	Downregulated
A_23_P823	0.004301	0.000973	-1.17	SLC25A13	solute carrier family 25 (aspartate/glutamate carrier), member 13	Downregulated
A_24_P801	0.004333	0.000985	-1.17	TMEM127	transmembrane protein 127	Downregulated
A_33_P330	0.004801	0.00114	-1.17	TRIM10	tripartite motif containing 10	Downregulated
A_19_P003	0.005098	0.00125	-1.17	HCG18	HLA complex group 18 (non-protein coding)	Downregulated
A_21_P001	0.006202	0.00166	-1.17	XLOC_l2_005276		Downregulated
A_23_P830	0.006645	0.00183	-1.17	HIATL1	hippocampus abundant transcript-like 1	Downregulated
A_33_P325	0.009508	0.00298	-1.17	P3H4	prolyl 3-hydroxylase family member 4 (non-enzymatic)	Downregulated
A_22_P000	0.011562	0.00387	-1.17	lnc-DALRD3-1	lnc-DALRD3-1:1	Downregulated
A_22_P000	0.011812	0.00397	-1.17	lnc-DLX2-5	lnc-DLX2-5:2	Downregulated
A_21_P000	0.01988	0.00772	-1.17	OSTM1-AS1	OSTM1 antisense RNA 1	Downregulated
A_24_P201	0.026043	0.0108	-1.17	FGFBP3	fibroblast growth factor binding protein 3	Downregulated
A_21_P000	0.034076	0.0151	-1.17	ZRANB2-AS1	ZRANB2 antisense RNA 1	Downregulated
A_22_P000	0.037888	0.0172	-1.17	LINC00877	long intergenic non-protein coding RNA 877	Downregulated
A_33_P324	0.042428	0.0197	-1.17	LUC7L3	LUC7-like 3 (S. cerevisiae)	Downregulated
A_33_P340	0.044274	0.0207	-1.17	KIF2A	kinesin heavy chain member 2A	Downregulated
A_22_P000	0.063964	0.0322	-1.17	lnc-CCNE2-1	lnc-CCNE2-1:1	Downregulated
A_22_P000	0.065575	0.0332	-1.17	lnc-APOBEC4	lnc-APOBEC4-1:1	Downregulated
A_33_P339	0.073875	0.0382	-1.17	GLTSCR1	glioma tumor suppressor candidate region gene 1	Downregulated
A_23_P255	0.001698	0.000225	-1.18	SMPD4	sphingomyelin phosphodiesterase 4, neutral membrane (neutral sphing	Downregulated
A_33_P333	0.001715	0.00023	-1.18	PTMS	parathymosin	Downregulated
A_23_P247	0.001827	0.000257	-1.18	COPS7A	COP9 signalosome subunit 7A	Downregulated
A_19_P003	0.001978	0.000294	-1.18	NEAT1	nuclear paraspeckle assembly transcript 1 (non-protein coding)	Downregulated
A_32_P182	0.001977	0.000294	-1.18	TALDO1	transaldolase 1	Downregulated
A_33_P323	0.002027	0.000308	-1.18	IL24	interleukin 24	Downregulated
A_23_P103	0.0021	0.000326	-1.18	YWHAH	tyrosine 3-monooxygenase/tryptophan 5-monooxygenase activation pro	Downregulated
A_23_P147	0.002112	0.00033	-1.18	ATXN2L	ataxin 2-like	Downregulated

A_24_P355	0.002132	0.000338	-1.18	FLI1	Fli-1 proto-oncogene, ETS transcription factor	Downregulated
A_33_P340	0.00218	0.000349	-1.18	TMEM19	transmembrane protein 19	Downregulated
A_23_P368	0.00226	0.00037	-1.18	FCHSD2	FCH and double SH3 domains 2	Downregulated
A_23_P647	0.003041	0.000581	-1.18	AAAS	achalasia, adrenocortical insufficiency, alacrimia	Downregulated
A_32_P117	0.003232	0.000639	-1.18	LYSMD4	LysM, putative peptidoglycan-binding, domain containing 4	Downregulated
A_23_P515	0.003365	0.000678	-1.18	DUSP12	dual specificity phosphatase 12	Downregulated
A_33_P323	0.003983	0.000867	-1.18	BOLA2B	bolA family member 2B	Downregulated
A_23_P117	0.004406	0.00101	-1.18	GZMB	granzyme B (granzyme 2, cytotoxic T-lymphocyte-associated serine este	Downregulated
A_23_P202	0.004628	0.00109	-1.18	ZNF22	zinc finger protein 22	Downregulated
A_23_P252	0.004859	0.00116	-1.18	RECQL	RecQ helicase-like	Downregulated
A_23_P348	0.005553	0.00141	-1.18	MKL1	megakaryoblastic leukemia (translocation) 1	Downregulated
A_21_P000	0.007949	0.00234	-1.18	lnc-CLN8-1	lnc-CLN8-1:1	Downregulated
A_22_P000	0.008048	0.00238	-1.18	lnc-TRAPPC8	lnc-TRAPPC8-2:1	Downregulated
A_21_P001	0.009184	0.00285	-1.18	lnc-FOXA2-1	lnc-FOXA2-1:3	Downregulated
A_33_P336	0.019522	0.00755	-1.18	BLNK	B-cell linker	Downregulated
A_22_P000	0.022447	0.00896	-1.18	LOC1027233	uncharacterized LOC102723373	Downregulated
A_19_P003	0.025432	0.0105	-1.18	LOC389602	uncharacterized LOC389602	Downregulated
A_24_P193	0.026142	0.0109	-1.18	CCNF	cyclin F	Downregulated
A_21_P000	0.029405	0.0126	-1.18	lnc-CYB5D2-1	lnc-CYB5D2-1:2	Downregulated
A_23_P111	0.044789	0.021	-1.18	ZBTB12	zinc finger and BTB domain containing 12	Downregulated
A_33_P333	0.063022	0.0317	-1.18	TAF4B	TAF4b RNA polymerase II, TATA box binding protein (TBP)-associated f	Downregulated
A_23_P111	0.072689	0.0375	-1.18	ZSCAN21	zinc finger and SCAN domain containing 21	Downregulated
A_23_P165	0.001787	0.000246	-1.19	STRN	striatin, calmodulin binding protein	Downregulated
A_23_P257	0.001805	0.00025	-1.19	HERC1	HECT and RLD domain containing E3 ubiquitin protein ligase family mer	Downregulated
A_23_P104	0.001956	0.000288	-1.19	BPNT1	3'(2'), 5'-bisphosphate nucleotidase 1	Downregulated
A_23_P350	0.001965	0.000292	-1.19	PRDM1	PR domain containing 1, with ZNF domain	Downregulated
A_33_P337	0.00198	0.000295	-1.19	HMGXB3	HMG box domain containing 3	Downregulated
A_23_P399	0.002006	0.000302	-1.19	TMEM256	transmembrane protein 256	Downregulated
A_32_P101	0.002023	0.000307	-1.19	FAM3C	family with sequence similarity 3, member C	Downregulated
A_33_P321	0.002033	0.00031	-1.19	SMNDC1	survival motor neuron domain containing 1	Downregulated
A_33_P336	0.002099	0.000326	-1.19	lnc-TBC1D29-	lnc-TBC1D29-1:1	Downregulated
A_23_P377	0.002124	0.000335	-1.19	ACTR2	ARP2 actin-related protein 2 homolog (yeast)	Downregulated
A_33_P340	0.002389	0.0004	-1.19	LOC1001293	uncharacterized LOC100129399	Downregulated
A_21_P001	0.002575	0.000451	-1.19	XLOC_l2_008140		Downregulated
A_23_P104	0.003223	0.000636	-1.19	DDIT4	DNA-damage-inducible transcript 4	Downregulated
A_33_P337	0.003527	0.000725	-1.19	ALDH1A1	aldehyde dehydrogenase 1 family, member A1	Downregulated
A_33_P323	0.004182	0.000932	-1.19	DDX18	DEAD (Asp-Glu-Ala-Asp) box polypeptide 18	Downregulated

A_24_P210	0.004205	0.00094	-1.19	BTBD7	BTB (POZ) domain containing 7	Downregulated
A_19_P008	0.004269	0.000963	-1.19	HIGD1A	HIG1 hypoxia inducible domain family, member 1A	Downregulated
A_22_P000	0.00431	0.000977	-1.19	lnc-AC10502(	lnc-AC105020.1-1:1	Downregulated
A_23_P309	0.004347	0.000991	-1.19	HECTD4	HECT domain containing E3 ubiquitin protein ligase 4	Downregulated
A_23_P141	0.005457	0.00138	-1.19	DRG2	developmentally regulated GTP binding protein 2	Downregulated
A_33_P337	0.005554	0.00142	-1.19	PYROXD1	pyridine nucleotide-disulphide oxidoreductase domain 1	Downregulated
A_23_P307	0.006841	0.0019	-1.19	CEP95	centrosomal protein 95kDa	Downregulated
A_23_P142	0.007455	0.00215	-1.19	LMAN2L	lectin, mannose-binding 2-like	Downregulated
A_22_P000	0.007941	0.00234	-1.19	lnc-HRH4-9	lnc-HRH4-9:1	Downregulated
A_33_P331	0.012389	0.00422	-1.19	ZNF419	zinc finger protein 419	Downregulated
A_33_P336	0.013004	0.00449	-1.19	PDHB	pyruvate dehydrogenase (lipoamide) beta	Downregulated
A_21_P000	0.013768	0.00483	-1.19	lnc-SUMF2-1	lnc-SUMF2-1:1	Downregulated
A_23_P851	0.016281	0.00602	-1.19	EMD	emerin	Downregulated
A_23_P319	0.016575	0.00616	-1.19	UHRF1BP1L	UHRF1 binding protein 1-like	Downregulated
A_33_P729	0.017296	0.0065	-1.19	LOC1019269	uncharacterized LOC101926943	Downregulated
A_23_P118	0.025191	0.0104	-1.19	LRRC36	leucine rich repeat containing 36	Downregulated
A_21_P001	0.029816	0.0128	-1.19	XLOC_l2_009790		Downregulated
A_23_P378	0.034214	0.0152	-1.19	IKZF4	IKAROS family zinc finger 4 (Eos)	Downregulated
A_32_P148	0.035227	0.0157	-1.19	UBXN2B	UBX domain protein 2B	Downregulated
A_33_P333	0.036536	0.0164	-1.19	USP17L7	ubiquitin specific peptidase 17-like family member 7	Downregulated
A_21_P000	0.044157	0.0207	-1.19	PSMD6-AS2	PSMD6 antisense RNA 2	Downregulated
A_24_P810	0.048872	0.0234	-1.19	ANO9	anoctamin 9	Downregulated
A_33_P321	0.076165	0.0397	-1.19	ATP7A	ATPase, Cu++ transporting, alpha polypeptide	Downregulated
A_33_P366	0.084486	0.045	-1.19	LINC00954	long intergenic non-protein coding RNA 954	Downregulated
A_33_P323	0.001594	0.0002	-1.2	CAPZB	capping protein (actin filament) muscle Z-line, beta	Downregulated
A_23_P380	0.001626	0.000208	-1.2	LMO4	LIM domain only 4	Downregulated
A_32_P534	0.001657	0.000215	-1.2	BOLA2B	bolA family member 2B	Downregulated
A_23_P156	0.001683	0.00022	-1.2	ANKS1A	ankyrin repeat and sterile alpha motif domain containing 1A	Downregulated
A_24_P194	0.001744	0.000235	-1.2	SAE1	SUMO1 activating enzyme subunit 1	Downregulated
A_23_P143	0.001808	0.000251	-1.2	ACIN1	apoptotic chromatin condensation inducer 1	Downregulated
A_24_P123	0.001958	0.000289	-1.2	PPAT	phosphoribosyl pyrophosphate amidotransferase	Downregulated
A_33_P325	0.001986	0.000297	-1.2	WDR55	WD repeat domain 55	Downregulated
A_24_P252	0.002027	0.000308	-1.2	BTN3A2	butyrophilin, subfamily 3, member A2	Downregulated
A_33_P323	0.002171	0.000347	-1.2	CBR4	carbonyl reductase 4	Downregulated
A_23_P454	0.002382	0.000399	-1.2	GDI1	GDP dissociation inhibitor 1	Downregulated
A_23_P371	0.002493	0.000427	-1.2	TAS2R43	taste receptor, type 2, member 43	Downregulated
A_23_P424	0.002746	0.000496	-1.2	POU2F1	POU class 2 homeobox 1	Downregulated

A_23_P211	0.002811	0.000514	-1.2	LAMTOR1	late endosomal/lysosomal adaptor, MAPK and MTOR activator 1	Downregulated
A_23_P121	0.002825	0.000517	-1.2	HACL1	2-hydroxyacyl-CoA lyase 1	Downregulated
A_22_P000	0.002951	0.000553	-1.2	lnc-CHRNA5-1	lnc-CHRNA5-1:1	Downregulated
A_21_P000	0.003335	0.00067	-1.2	GCFC2	GC-rich sequence DNA-binding factor 2	Downregulated
A_23_P895	0.004102	0.000905	-1.2	NLRP1	NLR family, pyrin domain containing 1	Downregulated
A_21_P000	0.005358	0.00135	-1.2	LOC1005066	hepatocellular carcinoma-associated antigen HCA25a	Downregulated
A_23_P329	0.008735	0.00267	-1.2	COG7	component of oligomeric golgi complex 7	Downregulated
A_23_P819	0.010546	0.00342	-1.2	HSD17B8	hydroxysteroid (17-beta) dehydrogenase 8	Downregulated
A_33_P323	0.011808	0.00397	-1.2	MPRIP	myosin phosphatase Rho interacting protein	Downregulated
A_24_P267	0.01239	0.00422	-1.2	SAMHD1	SAM domain and HD domain 1	Downregulated
A_23_P149	0.013312	0.00463	-1.2	MBTPS1	membrane-bound transcription factor peptidase, site 1	Downregulated
A_23_P135	0.017199	0.00646	-1.2	TLE1	transducin-like enhancer of split 1 (E(sp1) homolog, Drosophila)	Downregulated
A_33_P326	0.018895	0.00725	-1.2	NAA16	N(alpha)-acetyltransferase 16, NatA auxiliary subunit	Downregulated
A_33_P321	0.018895	0.00725	-1.2	NMNAT1	nicotinamide nucleotide adenylyltransferase 1	Downregulated
A_19_P003	0.023836	0.00968	-1.2	L3MBTL1	l(3)mbt-like 1 (Drosophila)	Downregulated
A_21_P000	0.024412	0.00999	-1.2	lnc-RP11-100	lnc-RP11-1007G5.2.1-2:1	Downregulated
A_23_P205	0.024412	0.00999	-1.2	PPIP5K1	diphosphoinositol pentakisphosphate kinase 1	Downregulated
A_21_P001	0.029966	0.0129	-1.2	XLOC_l2_001592		Downregulated
A_33_P324	0.05004	0.024	-1.2	OBP2B	odorant binding protein 2B	Downregulated
A_23_P700	0.001597	0.000201	-1.21	CD74	CD74 molecule, major histocompatibility complex, class II invariant chain	Downregulated
A_24_P124	0.001606	0.000203	-1.21	PSMA4	proteasome (prosome, macropain) subunit, alpha type, 4	Downregulated
A_23_P978	0.001663	0.000216	-1.21	LDB1	LIM domain binding 1	Downregulated
A_33_P322	0.001808	0.000251	-1.21	XPO5	exportin 5	Downregulated
A_23_P209	0.00193	0.000281	-1.21	PER2	period circadian clock 2	Downregulated
A_23_P251	0.001941	0.000283	-1.21	CPEB4	cytoplasmic polyadenylation element binding protein 4	Downregulated
A_33_P321	0.001978	0.000295	-1.21	DICER1	dicer 1, ribonuclease type III	Downregulated
A_22_P000	0.002114	0.000331	-1.21	PLAC4	placenta-specific 4	Downregulated
A_23_P605	0.002124	0.000335	-1.21	TOR2A	torsin family 2, member A	Downregulated
A_19_P003	0.002158	0.000343	-1.21	ZNF254	zinc finger protein 254	Downregulated
A_23_P138	0.002235	0.000363	-1.21	MLF2	myeloid leukemia factor 2	Downregulated
A_33_P333	0.002303	0.000379	-1.21	ARSD	arylsulfatase D	Downregulated
A_23_P115	0.002343	0.000389	-1.21	HNRNPH3	heterogeneous nuclear ribonucleoprotein H3 (2H9)	Downregulated
A_22_P000	0.0025	0.00043	-1.21	lnc-FAM78A-3	lnc-FAM78A-3:1	Downregulated
A_24_P176	0.002681	0.000477	-1.21	ATMIN	ATM interactor	Downregulated
A_23_P251	0.002707	0.000485	-1.21	ABCF1	ATP-binding cassette, sub-family F (GCN20), member 1	Downregulated
A_24_P331	0.003421	0.000694	-1.21	COMMD4	COMM domain containing 4	Downregulated
A_33_P371	0.003542	0.000731	-1.21	LOC283737	uncharacterized LOC283737	Downregulated

A_22_P000	0.005296	0.00132	-1.21	lnc-RGMA-1	lnc-RGMA-1:1	Downregulated
A_33_P380	0.005296	0.00132	-1.21	RYR3	ryanodine receptor 3	Downregulated
A_23_P877	0.005589	0.00143	-1.21	PLBD1	phospholipase B domain containing 1	Downregulated
A_24_P103	0.006176	0.00165	-1.21	IDI1	isopentenyl-diphosphate delta isomerase 1	Downregulated
A_32_P439	0.006315	0.0017	-1.21	DNAH17	dynein, axonemal, heavy chain 17	Downregulated
A_22_P000	0.009618	0.00303	-1.21	USP12-AS1	USP12 antisense RNA 1	Downregulated
A_22_P000	0.012113	0.0041	-1.21	ZNRD1-AS1	ZNRD1 antisense RNA 1	Downregulated
A_32_P129	0.012964	0.00447	-1.21	NHLRC3	NHL repeat containing 3	Downregulated
A_21_P000	0.014122	0.005	-1.21	LOC1027253	uncharacterized LOC102725333	Downregulated
A_22_P000	0.017124	0.00642	-1.21	LINC00927	long intergenic non-protein coding RNA 927	Downregulated
A_23_P312	0.020523	0.00802	-1.21	DNAJC4	DnaJ (Hsp40) homolog, subfamily C, member 4	Downregulated
A_23_P205	0.021012	0.00825	-1.21	WDR20	WD repeat domain 20	Downregulated
A_21_P001	0.035165	0.0157	-1.21	XLOC_l2_006794		Downregulated
A_24_P185	0.035408	0.0158	-1.21	TP53I13	tumor protein p53 inducible protein 13	Downregulated
A_23_P373	0.038	0.0173	-1.21	CCDC85C	coiled-coil domain containing 85C	Downregulated
A_23_P104	0.05204	0.0252	-1.21	MYPN	myopalladin	Downregulated
A_21_P000	0.06947	0.0355	-1.21	SNORD18C	small nucleolar RNA, C/D box 18C	Downregulated
A_23_P209	0.00163	0.000209	-1.22	ROCK2	Rho-associated, coiled-coil containing protein kinase 2	Downregulated
A_22_P000	0.001754	0.000238	-1.22	lnc-PMM2-6	lnc-PMM2-6:1	Downregulated
A_33_P366	0.001826	0.000256	-1.22	LOC644656	uncharacterized LOC644656	Downregulated
A_33_P326	0.001934	0.000282	-1.22	TCEANC2	transcription elongation factor A (SII) N-terminal and central domain con	Downregulated
A_33_P331	0.001979	0.000295	-1.22	KDM4B	lysine (K)-specific demethylase 4B	Downregulated
A_22_P000	0.002018	0.000305	-1.22	lnc-C11orf1-1	lnc-C11orf1-1:1	Downregulated
A_33_P339	0.002033	0.00031	-1.22	KMT2A	lysine (K)-specific methyltransferase 2A	Downregulated
A_23_P480	0.002062	0.000317	-1.22	NUP37	nucleoporin 37kDa	Downregulated
A_24_P371	0.002062	0.000318	-1.22	MRPL36	mitochondrial ribosomal protein L36	Downregulated
A_23_P210	0.002117	0.000332	-1.22	PTPN18	protein tyrosine phosphatase, non-receptor type 18 (brain-derived)	Downregulated
A_32_P577	0.002479	0.000423	-1.22	PMS2P1	postmeiotic segregation increased 2 pseudogene 1	Downregulated
A_33_P333	0.002719	0.000488	-1.22	DUSP11	dual specificity phosphatase 11 (RNA/RNP complex 1-interacting)	Downregulated
A_21_P000	0.002718	0.000488	-1.22	lnc-LYZL1-2	lnc-LYZL1-2:1	Downregulated
A_24_P122	0.003003	0.000569	-1.22	PSMF1	proteasome (prosome, macropain) inhibitor subunit 1 (PI31)	Downregulated
A_33_P321	0.003733	0.00079	-1.22	NBPF9	neuroblastoma breakpoint family, member 9	Downregulated
A_24_P919	0.004147	0.000919	-1.22	ATPAF1	ATP synthase mitochondrial F1 complex assembly factor 1	Downregulated
A_22_P000	0.004744	0.00112	-1.22	SLC25A25-A:	SLC25A25 antisense RNA 1	Downregulated
A_32_P130	0.00603	0.00159	-1.22	SLC16A7	solute carrier family 16 (monocarboxylate transporter), member 7	Downregulated
A_24_P596	0.007158	0.00203	-1.22	JAK3	Janus kinase 3	Downregulated
A_23_P469	0.007949	0.00234	-1.22	MMS19	MMS19 nucleotide excision repair homolog (S. cerevisiae)	Downregulated

A_24_P909	0.008594	0.00261	-1.22	HNRNPDL	heterogeneous nuclear ribonucleoprotein D-like	Downregulated
A_21_P000	0.009992	0.00319	-1.22	LOC1019276	uncharacterized LOC101927694	Downregulated
A_32_P910	0.013354	0.00465	-1.22	LOC1001290	uncharacterized LOC100129034	Downregulated
A_24_P940	0.015403	0.0056	-1.22	CCDC57	coiled-coil domain containing 57	Downregulated
A_32_P416	0.02096	0.00822	-1.22	NLRC5	NLR family, CARD domain containing 5	Downregulated
A_21_P000	0.026644	0.0111	-1.22	LRRC34	leucine rich repeat containing 34	Downregulated
A_33_P333	0.031215	0.0135	-1.22	SH3YL1	SH3 and SYLF domain containing 1	Downregulated
A_21_P000	0.075743	0.0394	-1.22	lnc-IRF2BPL-:	lnc-IRF2BPL-2:1	Downregulated
A_32_P357	0.078204	0.0409	-1.22	PPHLN1	periphilin 1	Downregulated
A_22_P000	0.087386	0.0471	-1.22	lnc-FAM63B-1	lnc-FAM63B-1:1	Downregulated
A_24_P798	0.001576	0.000197	-1.23	PBXIP1	pre-B-cell leukemia homeobox interacting protein 1	Downregulated
A_23_P345	0.002046	0.000313	-1.23	ZNF655	zinc finger protein 655	Downregulated
A_23_P968	0.002086	0.000323	-1.23	FAF1	Fas (TNFRSF6) associated factor 1	Downregulated
A_23_P349	0.002173	0.000348	-1.23	JTB	jumping translocation breakpoint	Downregulated
A_23_P139	0.002184	0.00035	-1.23	ERGIC2	ERGIC and golgi 2	Downregulated
A_33_P324	0.002365	0.000395	-1.23	SLC41A3	solute carrier family 41, member 3	Downregulated
A_23_P390	0.002503	0.000431	-1.23	RRAS	related RAS viral (r-ras) oncogene homolog	Downregulated
A_23_P320	0.003199	0.00063	-1.23	ZNF827	zinc finger protein 827	Downregulated
A_23_P217	0.003265	0.000648	-1.23	AK6	adenylate kinase 6	Downregulated
A_23_P245	0.006147	0.00164	-1.23	ACAT1	acetyl-CoA acetyltransferase 1	Downregulated
A_33_P325	0.00706	0.00199	-1.23	MFSD1	major facilitator superfamily domain containing 1	Downregulated
A_23_P127	0.00751	0.00217	-1.23	PPRC1	peroxisome proliferator-activated receptor gamma, coactivator-related 1	Downregulated
A_33_P341	0.011128	0.00366	-1.23	BTBD7	BTB (POZ) domain containing 7	Downregulated
A_22_P000	0.011362	0.00377	-1.23	LOC284648	uncharacterized LOC284648	Downregulated
A_33_P326	0.01517	0.0055	-1.23	LOC1027247	tricarboxylate transport protein, mitochondrial-like	Downregulated
A_22_P000	0.016063	0.00591	-1.23	lnc-ADAMTS7	lnc-ADAMTS7-1:3	Downregulated
A_23_P244	0.023072	0.00927	-1.23	CTSF	cathepsin F	Downregulated
A_23_P258	0.024838	0.0102	-1.23	ENOX2	ecto-NOX disulfide-thiol exchanger 2	Downregulated
A_21_P000	0.024825	0.0102	-1.23	lnc-PDE4D-1	lnc-PDE4D-1:1	Downregulated
A_33_P321	0.02969	0.0127	-1.23	AGPAT5	1-acylglycerol-3-phosphate O-acyltransferase 5	Downregulated
A_21_P000	0.03179	0.0138	-1.23	lnc-WRNIP1-2	lnc-WRNIP1-2:20	Downregulated
A_23_P106	0.044848	0.0211	-1.23	CHST6	carbohydrate (N-acetylglucosamine 6-O) sulfotransferase 6	Downregulated
A_24_P931	0.057031	0.0281	-1.23	LOC1001288	ACAH3104	Downregulated
A_21_P001	0.064522	0.0325	-1.23	LOC388692	uncharacterized LOC388692	Downregulated
A_23_P333	0.07196	0.0371	-1.23	SH3D19	SH3 domain containing 19	Downregulated
A_23_P425	0.072642	0.0375	-1.23	SPDYE1	speedy/RINGO cell cycle regulator family member E1	Downregulated
A_21_P000	0.001606	0.000203	-1.24	SNORA70E	small nucleolar RNA, H/ACA box 70E	Downregulated

A_23_P901	0.001611	0.000204	-1.24	PPP1R15A	protein phosphatase 1, regulatory subunit 15A	Downregulated
A_23_P546	0.001629	0.000209	-1.24	ATP6V0D1	ATPase, H+ transporting, lysosomal 38kDa, V0 subunit d1	Downregulated
A_33_P338	0.001633	0.00021	-1.24	CTDNEP1	CTD nuclear envelope phosphatase 1	Downregulated
A_23_P795	0.001635	0.00021	-1.24	SUCLG1	succinate-CoA ligase, alpha subunit	Downregulated
A_23_P206	0.001754	0.000238	-1.24	ITGAL	integrin, alpha L (antigen CD11A (p180), lymphocyte function-associate	Downregulated
A_24_P361	0.001772	0.000242	-1.24	CHD8	chromodomain helicase DNA binding protein 8	Downregulated
A_23_P100	0.00186	0.000264	-1.24	ZNF598	zinc finger protein 598	Downregulated
A_33_P333	0.001903	0.000274	-1.24	FCRL5	Fc receptor-like 5	Downregulated
A_33_P326	0.00193	0.000281	-1.24	NCBP1	nuclear cap binding protein subunit 1, 80kDa	Downregulated
A_23_P122	0.001973	0.000293	-1.24	HDAC2	histone deacetylase 2	Downregulated
A_23_P140	0.002006	0.000302	-1.24	SUPT20H	suppressor of Ty 20 homolog (S. cerevisiae)	Downregulated
A_23_P866	0.002125	0.000336	-1.24	HNRNPA3	heterogeneous nuclear ribonucleoprotein A3	Downregulated
A_24_P924	0.002558	0.000445	-1.24	PCNXL3	pecanex-like 3 (Drosophila)	Downregulated
A_23_P132	0.002572	0.00045	-1.24	SPCS3	signal peptidase complex subunit 3 homolog (S. cerevisiae)	Downregulated
A_23_P101	0.002818	0.000515	-1.24	TMEM38A	transmembrane protein 38A	Downregulated
A_23_P211	0.002853	0.000525	-1.24	NDUFV3	NADH dehydrogenase (ubiquinone) flavoprotein 3, 10kDa	Downregulated
A_23_P942	0.003338	0.000671	-1.24	OXR1	oxidation resistance 1	Downregulated
A_33_P342	0.005267	0.00131	-1.24	LOC1027251	uncharacterized LOC102725171	Downregulated
A_24_P942	0.005372	0.00135	-1.24	TRAPPC6B	trafficking protein particle complex 6B	Downregulated
A_23_P140	0.00638	0.00173	-1.24	DDX19B	DEAD (Asp-Glu-Ala-Asp) box polypeptide 19B	Downregulated
A_33_P360	0.006443	0.00176	-1.24	C12orf80	chromosome 12 open reading frame 80	Downregulated
A_33_P322	0.006923	0.00194	-1.24	LOC1720	dihydrofolate reductase pseudogene	Downregulated
A_22_P000	0.007511	0.00217	-1.24	lnc-POU5F1B	lnc-POU5F1B-3:3	Downregulated
A_33_P332	0.008765	0.00268	-1.24	FAM69A	family with sequence similarity 69, member A	Downregulated
A_21_P000	0.00896	0.00276	-1.24	PDCL3	phosducin-like 3	Downregulated
A_22_P000	0.015038	0.00544	-1.24	lnc-SNURF-1	lnc-SNURF-1:9	Downregulated
A_21_P000	0.015038	0.00544	-1.24	SNORD42B	small nucleolar RNA, C/D box 42B	Downregulated
A_24_P337	0.025764	0.0107	-1.24	ANKRA2	ankyrin repeat, family A (RFXANK-like), 2	Downregulated
A_21_P001	0.033792	0.0149	-1.24	DNAH17	dynein, axonemal, heavy chain 17	Downregulated
A_22_P000	0.038334	0.0174	-1.24	lnc-TPPP2-1	lnc-TPPP2-1:2	Downregulated
A_21_P000	0.075523	0.0393	-1.24	lnc-BLID-1	lnc-BLID-1:18	Downregulated
A_24_P276	0.001481	0.000177	-1.25	FCRLA	Fc receptor-like A	Downregulated
A_23_P215	0.001643	0.000212	-1.25	ECHDC1	ethylmalonyl-CoA decarboxylase 1	Downregulated
A_24_P126	0.001656	0.000215	-1.25	NR2C2AP	nuclear receptor 2C2-associated protein	Downregulated
A_23_P603	0.001663	0.000217	-1.25	C9orf64	chromosome 9 open reading frame 64	Downregulated
A_22_P000	0.001763	0.00024	-1.25	NUP50-AS1	NUP50 antisense RNA 1 (head to head)	Downregulated
A_33_P330	0.001772	0.000242	-1.25	MAP3K11	mitogen-activated protein kinase kinase kinase 11	Downregulated

A_24_P728	0.00193	0.000281	-1.25	XLOC_l2_011874		Downregulated
A_22_P000	0.001949	0.000286	-1.25	lnc-LRRC14-1	lnc-LRRC14-1:1	Downregulated
A_23_P212	0.002041	0.000312	-1.25	THUMPD3	THUMP domain containing 3	Downregulated
A_33_P322	0.002072	0.00032	-1.25	C9orf64	chromosome 9 open reading frame 64	Downregulated
A_23_P703	0.002168	0.000346	-1.25	PHF3	PHD finger protein 3	Downregulated
A_23_P152	0.002391	0.000402	-1.25	C17orf59	chromosome 17 open reading frame 59	Downregulated
A_23_P946	0.002538	0.00044	-1.25	TBC1D13	TBC1 domain family, member 13	Downregulated
A_23_P155	0.003358	0.000676	-1.25	XYLT2	xylosyltransferase II	Downregulated
A_22_P000	0.003469	0.000708	-1.25	CRTC3-AS1	CRTC3 antisense RNA 1	Downregulated
A_24_P381	0.004156	0.000923	-1.25	SYVN1	synovial apoptosis inhibitor 1, synoviolin	Downregulated
A_21_P000	0.004321	0.000981	-1.25	SNORD36A	small nucleolar RNA, C/D box 36A	Downregulated
A_33_P322	0.006058	0.0016	-1.25	C17orf50	chromosome 17 open reading frame 50	Downregulated
A_33_P324	0.006593	0.00181	-1.25	CEP350	centrosomal protein 350kDa	Downregulated
A_33_P364	0.006791	0.00189	-1.25	LOC1002893	uncharacterized LOC100289333	Downregulated
A_23_P133	0.006797	0.00189	-1.25	TBCK	TBC1 domain containing kinase	Downregulated
A_33_P329	0.007192	0.00205	-1.25	LRRC37A3	leucine rich repeat containing 37, member A3	Downregulated
A_22_P000	0.009487	0.00297	-1.25	lnc-SEBOX-1	lnc-SEBOX-1:1	Downregulated
A_24_P132	0.009487	0.00297	-1.25	RNF14	ring finger protein 14	Downregulated
A_21_P000	0.015464	0.00563	-1.25	lnc-KATNAL2	lnc-KATNAL2-1:1	Downregulated
A_21_P001	0.048785	0.0233	-1.25	LINC00993	long intergenic non-protein coding RNA 993	Downregulated
A_21_P000	0.060547	0.0302	-1.25	lnc-FUT8-2	lnc-FUT8-2:2	Downregulated
A_24_P144	0.077079	0.0402	-1.25	WFDC2	WAP four-disulfide core domain 2	Downregulated
A_23_P330	0.001453	0.00017	-1.26	DERL1	derlin 1	Downregulated
A_24_P914	0.001626	0.000208	-1.26	MARK2	MAP/microtubule affinity-regulating kinase 2	Downregulated
A_23_P127	0.001754	0.000238	-1.26	CTR9	CTR9, Paf1/RNA polymerase II complex component	Downregulated
A_32_P170	0.001797	0.000248	-1.26	SUB1	SUB1 homolog (S. cerevisiae)	Downregulated
A_33_P325	0.001808	0.000251	-1.26	LINC01561	long intergenic non-protein coding RNA 1561	Downregulated
A_33_P333	0.00196	0.00029	-1.26	TMEM208	transmembrane protein 208	Downregulated
A_23_P312	0.002016	0.000305	-1.26	PNPLA8	patatin-like phospholipase domain containing 8	Downregulated
A_23_P253	0.002227	0.000361	-1.26	PNOC	prepronociceptin	Downregulated
A_22_P000	0.002235	0.000363	-1.26	lnc-ATP11B-2	lnc-ATP11B-2:1	Downregulated
A_21_P001	0.002499	0.000429	-1.26	FOXN3	forkhead box N3	Downregulated
A_23_P310	0.002682	0.000478	-1.26	NUP43	nucleoporin 43kDa	Downregulated
A_24_P138	0.003331	0.000668	-1.26	FAM120A	family with sequence similarity 120A	Downregulated
A_24_P848	0.003747	0.000794	-1.26	LINC00032	long intergenic non-protein coding RNA 32	Downregulated
A_24_P945	0.003747	0.000794	-1.26	RBM15B	RNA binding motif protein 15B	Downregulated
A_23_P376	0.004843	0.00116	-1.26	TICAM1	toll-like receptor adaptor molecule 1	Downregulated

A_33_P342	0.005354	0.00134	-1.26	WHSC1	Wolf-Hirschhorn syndrome candidate 1	Downregulated
A_22_P000	0.00847	0.00256	-1.26	lnc-LPGAT1-2	lnc-LPGAT1-2:1	Downregulated
A_22_P000	0.008877	0.00272	-1.26	lnc-IGFALS-1	lnc-IGFALS-1:1	Downregulated
A_32_P146	0.009981	0.00318	-1.26	PDLIM5	PDZ and LIM domain 5	Downregulated
A_22_P000	0.010188	0.00328	-1.26	lnc-C11orf44-	lnc-C11orf44-2:1	Downregulated
A_33_P338	0.012906	0.00444	-1.26	AKAP9	A kinase (PRKA) anchor protein 9	Downregulated
A_23_P109	0.015033	0.00543	-1.26	SLC26A6	solute carrier family 26 (anion exchanger), member 6	Downregulated
A_23_P321	0.018829	0.00721	-1.26	SAAL1	serum amyloid A-like 1	Downregulated
A_21_P000	0.019078	0.00734	-1.26	IQCH-AS1	IQCH antisense RNA 1	Downregulated
A_24_P102	0.023517	0.00951	-1.26	DAPK2	death-associated protein kinase 2	Downregulated
A_33_P357	0.02857	0.0121	-1.26	NCR1	natural cytotoxicity triggering receptor 1	Downregulated
A_21_P001	0.028955	0.0123	-1.26	lnc-SIK1-4	lnc-SIK1-4:9	Downregulated
A_33_P341	0.032982	0.0145	-1.26	ARHGAP44	Rho GTPase activating protein 44	Downregulated
A_33_P322	0.03335	0.0147	-1.26	LIMS3-LOC44	LIMS3-LOC440895 readthrough	Downregulated
A_23_P160	0.053925	0.0263	-1.26	GALE	UDP-galactose-4-epimerase	Downregulated
A_21_P000	0.070917	0.0364	-1.26	lnc-DHX37-5	lnc-DHX37-5:2	Downregulated
A_33_P378	0.001563	0.000194	-1.27	PAK1	p21 protein (Cdc42/Rac)-activated kinase 1	Downregulated
A_33_P326	0.001604	0.000202	-1.27	DCTN1	dynactin 1	Downregulated
A_23_P434	0.001683	0.00022	-1.27	CAST	calpastatin	Downregulated
A_23_P877	0.001854	0.000263	-1.27	PARPBP	PARP1 binding protein	Downregulated
A_23_P126	0.001949	0.000286	-1.27	S100A11	S100 calcium binding protein A11	Downregulated
A_24_P115	0.002043	0.000313	-1.27	COX15	cytochrome c oxidase assembly homolog 15 (yeast)	Downregulated
A_21_P000	0.002078	0.000322	-1.27	lnc-HES1-2	lnc-HES1-2:6	Downregulated
A_33_P330	0.002356	0.000392	-1.27	UQCC1	ubiquinol-cytochrome c reductase complex assembly factor 1	Downregulated
A_23_P214	0.003232	0.000639	-1.27	PHF19	PHD finger protein 19	Downregulated
A_33_P330	0.004882	0.00117	-1.27	TTC23L	tetratricopeptide repeat domain 23-like	Downregulated
A_33_P340	0.004977	0.0012	-1.27	PHC3	polyhomeotic homolog 3 (Drosophila)	Downregulated
A_33_P334	0.005095	0.00124	-1.27	LOC1019276	ERV-FRD provirus ancestral Env polyprotein-like	Downregulated
A_33_P328	0.008919	0.00274	-1.27	UHRF1BP1L	UHRF1 binding protein 1-like	Downregulated
A_23_P426	0.00959	0.00302	-1.27	ZYG11B	zyg-11 family member B, cell cycle regulator	Downregulated
A_23_P217	0.011865	0.004	-1.27	EHMT1	euchromatic histone-lysine N-methyltransferase 1	Downregulated
A_23_P386	0.028176	0.0119	-1.27	GTF2H3	general transcription factor IIH, polypeptide 3, 34kDa	Downregulated
A_24_P363	0.037512	0.017	-1.27	FTSJ2	FtsJ RNA methyltransferase homolog 2 (E. coli)	Downregulated
A_33_P324	0.037512	0.017	-1.27	MZF1	myeloid zinc finger 1	Downregulated
A_19_P003	0.037512	0.017	-1.27	XLOC_l2_015561		Downregulated
A_24_P392	0.053369	0.0259	-1.27	MYPOP	Myb-related transcription factor, partner of profilin	Downregulated
A_24_P299	0.001403	0.00016	-1.28	ARPC5L	actin related protein 2/3 complex, subunit 5-like	Downregulated

A_23_P203	0.001422	0.000163	-1.28	DDX11	DEAD/H (Asp-Glu-Ala-Asp/His) box helicase 11	Downregulated
A_23_P170	0.001433	0.000165	-1.28	PSMB2	proteasome (prosome, macropain) subunit, beta type, 2	Downregulated
A_23_P945	0.001575	0.000196	-1.28	ANXA1	annexin A1	Downregulated
A_33_P333	0.001618	0.000206	-1.28	WIPF1	WAS/WASL interacting protein family, member 1	Downregulated
A_23_P451	0.001766	0.000241	-1.28	PROSC	proline synthetase co-transcribed homolog (bacterial)	Downregulated
A_23_P945	0.001908	0.000275	-1.28	TMEM141	transmembrane protein 141	Downregulated
A_23_P106	0.00206	0.000317	-1.28	NOB1	NIN1/RPN12 binding protein 1 homolog (S. cerevisiae)	Downregulated
A_22_P000	0.002189	0.000351	-1.28	lnc-SH2D7-3	lnc-SH2D7-3:1	Downregulated
A_23_P144	0.002361	0.000394	-1.28	GALNT7	polypeptide N-acetylgalactosaminyltransferase 7	Downregulated
A_33_P333	0.002989	0.000564	-1.28	WASH5P	WAS protein family homolog 5 pseudogene	Downregulated
A_33_P681	0.005462	0.00138	-1.28	LOC1005068	uncharacterized LOC100506844	Downregulated
A_23_P796	0.007222	0.00206	-1.28	CCDC93	coiled-coil domain containing 93	Downregulated
A_22_P000	0.008431	0.00254	-1.28	lnc-LINC0034	lnc-LINC00346-2:1	Downregulated
A_22_P000	0.018659	0.00713	-1.28	ZFPM2-AS1	ZFPM2 antisense RNA 1	Downregulated
A_24_P399	0.020052	0.0078	-1.28	TXNL4B	thioredoxin-like 4B	Downregulated
A_21_P001	0.04072	0.0187	-1.28	NUTM2A-AS1	NUTM2A antisense RNA 1	Downregulated
A_22_P000	0.044301	0.0207	-1.28	lnc-ARMC6-1	lnc-ARMC6-1:1	Downregulated
A_21_P001	0.049305	0.0236	-1.28	lnc-C22orf26-	lnc-C22orf26-2:10	Downregulated
A_22_P000	0.056721	0.0279	-1.28	lnc-ZMYM2-4	lnc-ZMYM2-4:1	Downregulated
A_23_P334	0.001433	0.000165	-1.29	ITFG1	integrin alpha FG-GAP repeat containing 1	Downregulated
A_23_P165	0.00162	0.000206	-1.29	RFXANK	regulatory factor X-associated ankyrin-containing protein	Downregulated
A_24_P189	0.001635	0.00021	-1.29	LMF1	lipase maturation factor 1	Downregulated
A_23_P691	0.001719	0.00023	-1.29	DYNC1LI1	dynein, cytoplasmic 1, light intermediate chain 1	Downregulated
A_33_P323	0.001785	0.000245	-1.29	UTRN	utrophin	Downregulated
A_33_P321	0.001798	0.000248	-1.29	CS	citrate synthase	Downregulated
A_21_P000	0.001842	0.00026	-1.29	SNORA70B	small nucleolar RNA, H/ACA box 70B	Downregulated
A_23_P209	0.001871	0.000267	-1.29	CYP1B1	cytochrome P450, family 1, subfamily B, polypeptide 1	Downregulated
A_24_P940	0.001889	0.000271	-1.29	CTBS	chitobiase, di-N-acetyl-	Downregulated
A_23_P425	0.001951	0.000287	-1.29	TRIM14	tripartite motif containing 14	Downregulated
A_23_P129	0.002568	0.000447	-1.29	C11orf24	chromosome 11 open reading frame 24	Downregulated
A_23_P607	0.002805	0.000512	-1.29	ASMTL-AS1	ASMTL antisense RNA 1	Downregulated
A_23_P545	0.003392	0.000686	-1.29	MKL2	MKL/myocardin-like 2	Downregulated
A_33_P322	0.003446	0.000701	-1.29	ALKBH3	alkB, alkylation repair homolog 3 (E. coli)	Downregulated
A_22_P000	0.004079	0.000896	-1.29	RPARP-AS1	RPARP antisense RNA 1	Downregulated
A_33_P340	0.00487	0.00117	-1.29	GGTLC2	gamma-glutamyltransferase light chain 2	Downregulated
A_23_P141	0.006915	0.00194	-1.29	TYK2	tyrosine kinase 2	Downregulated
A_23_P137	0.007306	0.00209	-1.29	TBX19	T-box 19	Downregulated

A_21_P000	0.007491	0.00216	-1.29	lnc-OIP5-1	lnc-OIP5-1:1	Downregulated
A_21_P001	0.008324	0.0025	-1.29	lnc-TEX261-2	lnc-TEX261-2:1	Downregulated
A_24_P397	0.008765	0.00268	-1.29	GCNT2	glucosaminyl (N-acetyl) transferase 2, I-branching enzyme (I blood grou	Downregulated
A_23_P565	0.009793	0.0031	-1.29	IL37	interleukin 37	Downregulated
A_21_P001	0.012945	0.00446	-1.29	XLOC_l2_009159		Downregulated
A_23_P918	0.01767	0.00667	-1.29	DCBLD2	discoidin, CUB and LCCL domain containing 2	Downregulated
A_21_P000	0.030769	0.0133	-1.29	SCARNA14	small Cajal body-specific RNA 14	Downregulated
A_23_P420	0.042335	0.0197	-1.29	CIT	citron rho-interacting serine/threonine kinase	Downregulated
A_21_P000	0.045135	0.0212	-1.29	lnc-FTH1-1	lnc-FTH1-1:1	Downregulated
A_22_P000	0.045135	0.0212	-1.29	lnc-SLC6A12-	lnc-SLC6A12-4:1	Downregulated
A_23_P687	0.047581	0.0226	-1.29	DNAJC28	DnaJ (Hsp40) homolog, subfamily C, member 28	Downregulated
A_33_P332	0.075672	0.0394	-1.29	CAGE1	cancer antigen 1	Downregulated
A_24_P328	0.085795	0.0461	-1.29	SOCS5	suppressor of cytokine signaling 5	Downregulated
A_23_P145	0.001421	0.000163	-1.3	FUCA2	fucosidase, alpha-L- 2, plasma	Downregulated
A_24_P316	0.001429	0.000164	-1.3	MLLT10	myeloid/lymphoid or mixed-lineage leukemia (trithorax homolog, Drosopl	Downregulated
A_23_P256	0.001802	0.000249	-1.3	LAS1L	LAS1-like (S. cerevisiae)	Downregulated
A_22_P000	0.001889	0.000271	-1.3	lnc-PAX9-4	lnc-PAX9-4:1	Downregulated
A_24_P100	0.001906	0.000275	-1.3	EMC4	ER membrane protein complex subunit 4	Downregulated
A_23_P255	0.002101	0.000327	-1.3	DCAF12	DDB1 and CUL4 associated factor 12	Downregulated
A_23_P317	0.002156	0.000343	-1.3	UIMC1	ubiquitin interaction motif containing 1	Downregulated
A_22_P000	0.002472	0.000421	-1.3	lnc-RPL36AL-	lnc-RPL36AL-1:1	Downregulated
A_33_P384	0.002984	0.000562	-1.3	FHL1	four and a half LIM domains 1	Downregulated
A_22_P000	0.002984	0.000562	-1.3	lnc-GPD2-1	lnc-GPD2-1:1	Downregulated
A_24_P290	0.003219	0.000635	-1.3	OGDH	oxoglutarate (alpha-ketoglutarate) dehydrogenase (lipoamide)	Downregulated
A_23_P952	0.003228	0.000638	-1.3	RBKS	ribokinase	Downregulated
A_33_P333	0.003401	0.000689	-1.3	UBE2Q2	ubiquitin-conjugating enzyme E2Q family member 2	Downregulated
A_33_P383	0.00366	0.000767	-1.3	C19orf24	chromosome 19 open reading frame 24	Downregulated
A_33_P348	0.003663	0.000768	-1.3	SNORA19	small nucleolar RNA, H/ACA box 19	Downregulated
A_33_P323	0.003791	0.000807	-1.3	ITPR2	inositol 1,4,5-trisphosphate receptor, type 2	Downregulated
A_21_P000	0.004257	0.000959	-1.3	SNORD1B	small nucleolar RNA, C/D box 1B	Downregulated
A_24_P124	0.005096	0.00125	-1.3	NDNL2	necdin-like 2	Downregulated
A_23_P499	0.005683	0.00146	-1.3	KRT10	keratin 10, type I	Downregulated
A_33_P345	0.006372	0.00173	-1.3	LINC01573	long intergenic non-protein coding RNA 1573	Downregulated
A_33_P333	0.006506	0.00178	-1.3	ACACB	acetyl-CoA carboxylase beta	Downregulated
A_33_P646	0.007927	0.00233	-1.3	SNHG7	small nucleolar RNA host gene 7 (non-protein coding)	Downregulated
A_22_P000	0.013405	0.00467	-1.3	lnc-C1orf31-4	lnc-C1orf31-4:1	Downregulated
A_22_P000	0.01422	0.00505	-1.3	lnc-CCNB1IP	lnc-CCNB1IP1-2:1	Downregulated

A_33_P334	0.015271	0.00555	-1.3	ZC3H18	zinc finger CCCH-type containing 18	Downregulated
A_21_P001	0.016739	0.00624	-1.3	lnc-PRC1-1	lnc-PRC1-1:1	Downregulated
A_21_P000	0.052859	0.0256	-1.3	lnc-DMRTA1-5	lnc-DMRTA1-5:2	Downregulated
A_33_P333	0.065215	0.033	-1.3	ANKK1	ankyrin repeat and kinase domain containing 1	Downregulated
A_24_P204	0.001333	0.000145	-1.31	ANXA2P1	annexin A2 pseudogene 1	Downregulated
A_32_P220	0.001421	0.000163	-1.31	ZFAND6	zinc finger, AN1-type domain 6	Downregulated
A_23_P104	0.001443	0.000167	-1.31	ITGB1	integrin, beta 1 (fibronectin receptor, beta polypeptide, antigen CD29 in	Downregulated
A_33_P342	0.001808	0.000251	-1.31	CADM1	cell adhesion molecule 1	Downregulated
A_23_P329	0.001936	0.000282	-1.31	RHBDF2	rhomboid 5 homolog 2 (Drosophila)	Downregulated
A_33_P338	0.001948	0.000285	-1.31	ATP6V1D	ATPase, H+ transporting, lysosomal 34kDa, V1 subunit D	Downregulated
A_33_P333	0.002114	0.000331	-1.31	INSR	insulin receptor	Downregulated
A_21_P000	0.002568	0.000448	-1.31	lnc-AC00310	lnc-AC003101.1.1-1:1	Downregulated
A_23_P591	0.003119	0.000604	-1.31	LSM2	LSM2 homolog, U6 small nuclear RNA associated (S. cerevisiae)	Downregulated
A_33_P325	0.00321	0.000633	-1.31	RBM48	RNA binding motif protein 48	Downregulated
A_23_P124	0.003315	0.000664	-1.31	QSOX1	quiescin Q6 sulfhydryl oxidase 1	Downregulated
A_22_P000	0.003535	0.000729	-1.31	lnc-WNT1-2	lnc-WNT1-2:1	Downregulated
A_23_P141	0.004184	0.000933	-1.31	FBXL20	F-box and leucine-rich repeat protein 20	Downregulated
A_23_P131	0.004203	0.000939	-1.31	PRADC1	protease-associated domain containing 1	Downregulated
A_33_P337	0.004529	0.00105	-1.31	TLE1	transducin-like enhancer of split 1 (E(sp1) homolog, Drosophila)	Downregulated
A_23_P505	0.004627	0.00108	-1.31	DMPK	dystrophia myotonica-protein kinase	Downregulated
A_22_P000	0.004969	0.0012	-1.31	LOC1001316	uncharacterized LOC100131655	Downregulated
A_33_P362	0.005253	0.00131	-1.31	CDRT8	CMT1A duplicated region transcript 8	Downregulated
A_24_P879	0.006235	0.00167	-1.31	WAC-AS1	WAC antisense RNA 1 (head to head)	Downregulated
A_24_P367	0.006645	0.00183	-1.31	SOCS7	suppressor of cytokine signaling 7	Downregulated
A_33_P340	0.006846	0.00191	-1.31	SEH1L	SEH1-like (S. cerevisiae)	Downregulated
A_23_P477	0.00743	0.00214	-1.31	FOLR2	folate receptor 2 (fetal)	Downregulated
A_33_P328	0.01771	0.00669	-1.31	CLDN3	claudin 3	Downregulated
A_32_P180	0.022311	0.00889	-1.31	PCYOX1	prenylcysteine oxidase 1	Downregulated
A_33_P336	0.022662	0.00907	-1.31	ERCC1	excision repair cross-complementation group 1	Downregulated
A_21_P001	0.025587	0.0106	-1.31	lnc-CSTB-1	lnc-CSTB-1:5	Downregulated
A_32_P528	0.034462	0.0153	-1.31	TM7SF3	transmembrane 7 superfamily member 3	Downregulated
A_33_P328	0.042642	0.0198	-1.31	SNHG7	small nucleolar RNA host gene 7 (non-protein coding)	Downregulated
A_21_P000	0.048955	0.0234	-1.31	lnc-PELI2-6	lnc-PELI2-6:1	Downregulated
A_23_P348	0.058355	0.0289	-1.31	CDHR3	cadherin-related family member 3	Downregulated
A_22_P000	0.06469	0.0327	-1.31	lnc-ADAM21-2	lnc-ADAM21-2:1	Downregulated
A_33_P326	0.090474	0.0491	-1.31	FAAH	fatty acid amide hydrolase	Downregulated
A_19_P003	0.001359	0.00015	-1.32	UBE2E3	ubiquitin-conjugating enzyme E2E 3	Downregulated

A_24_P899	0.001376	0.000153	-1.32	NAIF1	nuclear apoptosis inducing factor 1	Downregulated
A_23_P680	0.001398	0.000158	-1.32	STAT4	signal transducer and activator of transcription 4	Downregulated
A_33_P376	0.00151	0.000182	-1.32	DCAF4L2	DDB1 and CUL4 associated factor 4-like 2	Downregulated
A_23_P102	0.00151	0.000182	-1.32	NFS1	NFS1 cysteine desulfurase	Downregulated
A_33_P322	0.00178	0.000244	-1.32	UTY	ubiquitously transcribed tetratricopeptide repeat containing, Y-linked	Downregulated
A_21_P000	0.001812	0.000252	-1.32	lnc-C15orf2-2	lnc-C15orf2-2:27	Downregulated
A_23_P146	0.001904	0.000275	-1.32	ZFAND1	zinc finger, AN1-type domain 1	Downregulated
A_23_P110	0.002012	0.000303	-1.32	DUSP1	dual specificity phosphatase 1	Downregulated
A_24_P263	0.002229	0.000362	-1.32	DOCK2	dedicator of cytokinesis 2	Downregulated
A_32_P563	0.002389	0.0004	-1.32	RBMX	RNA binding motif protein, X-linked	Downregulated
A_21_P001	0.00247	0.000421	-1.32	XLOC_l2_013328		Downregulated
A_23_P117	0.002738	0.000493	-1.32	CHRM5	cholinergic receptor, muscarinic 5	Downregulated
A_22_P000	0.002909	0.000541	-1.32	lnc-CBR1-1	lnc-CBR1-1:1	Downregulated
A_33_P323	0.003447	0.000702	-1.32	FAM122B	family with sequence similarity 122B	Downregulated
A_24_P693	0.003531	0.000727	-1.32	LOC1001909	uncharacterized LOC100190986	Downregulated
A_21_P001	0.003572	0.000742	-1.32	GBP3	guanylate binding protein 3	Downregulated
A_22_P000	0.004402	0.00101	-1.32	lnc-ZMYM3-1	lnc-ZMYM3-1:1	Downregulated
A_33_P321	0.006254	0.00168	-1.32	HDDC2	HD domain containing 2	Downregulated
A_21_P000	0.007778	0.00228	-1.32	SNORA70D	small nucleolar RNA, H/ACA box 70D	Downregulated
A_32_P228	0.01158	0.00388	-1.32	PRDM10	PR domain containing 10	Downregulated
A_33_P333	0.01221	0.00414	-1.32	LOC1001282	uncharacterized LOC100128219	Downregulated
A_23_P411	0.012756	0.00438	-1.32	B3GALNT1	beta-1,3-N-acetylgalactosaminyltransferase 1 (globoside blood group)	Downregulated
A_33_P333	0.01675	0.00625	-1.32	ATXN7L1	ataxin 7-like 1	Downregulated
A_33_P339	0.030972	0.0134	-1.32	SDHA	succinate dehydrogenase complex, subunit A, flavoprotein (Fp)	Downregulated
A_23_P202	0.039243	0.0179	-1.32	CYP2R1	cytochrome P450, family 2, subfamily R, polypeptide 1	Downregulated
A_21_P000	0.049381	0.0237	-1.32	lnc-SLC2A13-	lnc-SLC2A13-3:2	Downregulated
A_22_P000	0.069855	0.0358	-1.32	lnc-SLC7A6-1	lnc-SLC7A6-1:1	Downregulated
A_24_P171	0.001403	0.000159	-1.33	RASSF5	Ras association (RalGDS/AF-6) domain family member 5	Downregulated
A_23_P129	0.001548	0.00019	-1.33	PLEKHO2	pleckstrin homology domain containing, family O member 2	Downregulated
A_21_P000	0.001551	0.000191	-1.33	SNORA70C	small nucleolar RNA, H/ACA box 70C	Downregulated
A_19_P003	0.001595	0.0002	-1.33	FLJ32255	uncharacterized LOC643977	Downregulated
A_23_P391	0.001698	0.000225	-1.33	LPHN1	latrophilin 1	Downregulated
A_23_P212	0.001802	0.000249	-1.33	NKTR	natural killer cell triggering receptor	Downregulated
A_22_P000	0.002124	0.000335	-1.33	LOC1001297	uncharacterized LOC100129781	Downregulated
A_23_P432	0.002183	0.00035	-1.33	RTN4RL1	reticulon 4 receptor-like 1	Downregulated
A_23_P847	0.002221	0.00036	-1.33	PLRG1	pleiotropic regulator 1	Downregulated
A_33_P327	0.002849	0.000524	-1.33	RHOQ	ras homolog family member Q	Downregulated

A_33_P369	0.002892	0.000537	-1.33	SEMA4D	sema domain, immunoglobulin domain (Ig), transmembrane domain (TM	Downregulated
A_21_P000	0.002931	0.000547	-1.33	SNORA70	small nucleolar RNA, H/ACA box 70	Downregulated
A_33_P330	0.003158	0.000617	-1.33	KIAA1549L	KIAA1549-like	Downregulated
A_23_P329	0.004315	0.000979	-1.33	ARMC10	armadillo repeat containing 10	Downregulated
A_23_P103	0.0059	0.00154	-1.33	AKR7A3	aldo-keto reductase family 7, member A3 (aflatoxin aldehyde reductase)	Downregulated
A_21_P001	0.006544	0.00179	-1.33	LOC1002880	uncharacterized LOC100288069	Downregulated
A_23_P855	0.00863	0.00262	-1.33	RNF2	ring finger protein 2	Downregulated
A_33_P384	0.010475	0.00339	-1.33	MAGI1-IT1	MAGI1 intronic transcript 1 (non-protein coding)	Downregulated
A_22_P000	0.011167	0.00368	-1.33	RNF219-AS1	RNF219 antisense RNA 1	Downregulated
A_32_P133	0.011499	0.00383	-1.33	SPON1	spondin 1, extracellular matrix protein	Downregulated
A_22_P000	0.016121	0.00594	-1.33	lnc-TM9SF2-2	lnc-TM9SF2-2:1	Downregulated
A_33_P330	0.02602	0.0108	-1.33	TPP2	tripeptidyl peptidase II	Downregulated
A_21_P000	0.029173	0.0125	-1.33	lnc-NTRK2-3	lnc-NTRK2-3:1	Downregulated
A_21_P000	0.035596	0.0159	-1.33	SNORA11	small nucleolar RNA, H/ACA box 11	Downregulated
A_22_P000	0.046324	0.0219	-1.33	lnc-PIK3R1-3	lnc-PIK3R1-3:1	Downregulated
A_21_P000	0.051724	0.025	-1.33	lnc-RPL24-2	lnc-RPL24-2:4	Downregulated
A_24_P151	0.076749	0.04	-1.33	MYL4	myosin, light chain 4, alkali; atrial, embryonic	Downregulated
A_33_P323	0.001286	0.000136	-1.34	TSC22D3	TSC22 domain family, member 3	Downregulated
A_24_P944	0.001299	0.000139	-1.34	HP1BP3	heterochromatin protein 1, binding protein 3	Downregulated
A_24_P837	0.001324	0.000143	-1.34	SPNS2	spinster homolog 2 (Drosophila)	Downregulated
A_33_P327	0.001383	0.000154	-1.34	DNMT3A	DNA (cytosine-5-)-methyltransferase 3 alpha	Downregulated
A_33_P332	0.001404	0.00016	-1.34	RAB8A	RAB8A, member RAS oncogene family	Downregulated
A_23_P362	0.001504	0.000181	-1.34	UBR2	ubiquitin protein ligase E3 component n-recognin 2	Downregulated
A_23_P207	0.00152	0.000184	-1.34	BRCA1	breast cancer 1, early onset	Downregulated
A_24_P194	0.001556	0.000192	-1.34	FXYD5	FXYD domain containing ion transport regulator 5	Downregulated
A_24_P285	0.001696	0.000224	-1.34	IDS	iduronate 2-sulfatase	Downregulated
A_21_P000	0.001958	0.000289	-1.34	SNORD5	small nucleolar RNA, C/D box 5	Downregulated
A_21_P000	0.002	0.0003	-1.34	lnc-AC06925[illegible]	lnc-AC069257.9.1-5:2	Downregulated
A_24_P406	0.002719	0.000488	-1.34	MTMR2	myotubularin related protein 2	Downregulated
A_24_P520	0.002886	0.000534	-1.34	PDS5A	PDS5 cohesin associated factor A	Downregulated
A_24_P272	0.002889	0.000536	-1.34	AVL9	AVL9 homolog (S. cerevisiase)	Downregulated
A_24_P631	0.003155	0.000615	-1.34	P2RY13	purinergic receptor P2Y, G-protein coupled, 13	Downregulated
A_23_P111	0.003155	0.000615	-1.34	RSPO3	R-spondin 3	Downregulated
A_24_P370	0.003203	0.000631	-1.34	MAN2B1	mannosidase, alpha, class 2B, member 1	Downregulated
A_23_P462	0.005346	0.00134	-1.34	CELA2A	chymotrypsin-like elastase family, member 2A	Downregulated
A_32_P723	0.005869	0.00153	-1.34	TRIM59	tripartite motif containing 59	Downregulated
A_32_P226	0.00616	0.00164	-1.34	ZFHX2	zinc finger homeobox 2	Downregulated

A_33_P325	0.007979	0.00236	-1.34	SLC35B4	solute carrier family 35 (UDP-xylose/UDP-N-acetylglucosamine transporte	Downregulated
A_33_P384	0.009157	0.00284	-1.34	FRYL	FRY-like	Downregulated
A_33_P333	0.010538	0.00342	-1.34	SCARNA13	small Cajal body-specific RNA 13	Downregulated
A_23_P229	0.012416	0.00423	-1.34	NFYC	nuclear transcription factor Y, gamma	Downregulated
A_24_P373	0.051456	0.0248	-1.34	C19orf25	chromosome 19 open reading frame 25	Downregulated
A_23_P319	0.001265	0.000132	-1.35	VPS28	vacuolar protein sorting 28 homolog (S. cerevisiae)	Downregulated
A_22_P000	0.001301	0.000139	-1.35	LINC01258	long intergenic non-protein coding RNA 1258	Downregulated
A_23_P405	0.001322	0.000143	-1.35	WIZ	widely interspaced zinc finger motifs	Downregulated
A_21_P001	0.001376	0.000153	-1.35	CS	citrate synthase	Downregulated
A_32_P181	0.001398	0.000158	-1.35	DOCK8	dedicator of cytokinesis 8	Downregulated
A_22_P000	0.001474	0.000174	-1.35	lnc-SETDB1-1	lnc-SETDB1-1:2	Downregulated
A_32_P900	0.001478	0.000176	-1.35	C4orf48	chromosome 4 open reading frame 48	Downregulated
A_23_P676	0.001492	0.000179	-1.35	CAPNS1	calpain, small subunit 1	Downregulated
A_23_P270	0.001636	0.000211	-1.35	UBE2G1	ubiquitin-conjugating enzyme E2G 1	Downregulated
A_23_P108	0.001706	0.000228	-1.35	TLR1	toll-like receptor 1	Downregulated
A_33_P325	0.001916	0.000278	-1.35	SNORD3B-1	small nucleolar RNA, C/D box 3B-1	Downregulated
A_33_P325	0.002013	0.000304	-1.35	MYLIP	myosin regulatory light chain interacting protein	Downregulated
A_32_P207	0.002034	0.00031	-1.35	ARL16	ADP-ribosylation factor-like 16	Downregulated
A_23_P375	0.002078	0.000322	-1.35	COL19A1	collagen, type XIX, alpha 1	Downregulated
A_23_P254	0.002117	0.000332	-1.35	CENPU	centromere protein U	Downregulated
A_33_P322	0.002391	0.000402	-1.35	CYFIP2	cytoplasmic FMR1 interacting protein 2	Downregulated
A_22_P000	0.002411	0.000406	-1.35	lnc-NFYB-2	lnc-NFYB-2:1	Downregulated
A_21_P000	0.002836	0.00052	-1.35	lnc-AMELY-4	lnc-AMELY-4:1	Downregulated
A_21_P000	0.003022	0.000575	-1.35	SYS1	Sys1 golgi trafficking protein	Downregulated
A_24_P199	0.004441	0.00102	-1.35	RNF2	ring finger protein 2	Downregulated
A_33_P321	0.004694	0.00111	-1.35	FCHSD2	FCH and double SH3 domains 2	Downregulated
A_24_P734	0.005017	0.00122	-1.35	CCNYL1	cyclin Y-like 1	Downregulated
A_23_P317	0.006006	0.00158	-1.35	ANAPC4	anaphase promoting complex subunit 4	Downregulated
A_24_P244	0.00628	0.00169	-1.35	ANAPC15	anaphase promoting complex subunit 15	Downregulated
A_23_P999	0.006837	0.0019	-1.35	MAGEL2	MAGE-like 2	Downregulated
A_23_P170	0.00999	0.00319	-1.35	TRAIP	TRAF interacting protein	Downregulated
A_23_P159	0.021596	0.00854	-1.35	TOPBP1	topoisomerase (DNA) II binding protein 1	Downregulated
A_22_P000	0.021708	0.00859	-1.35	EIF4ENIF1	eukaryotic translation initiation factor 4E nuclear import factor 1	Downregulated
A_24_P797	0.028393	0.0121	-1.35	COX20	COX20 cytochrome c oxidase assembly factor	Downregulated
A_23_P316	0.029195	0.0125	-1.35	GLIS1	GLIS family zinc finger 1	Downregulated
A_22_P000	0.033609	0.0148	-1.35	lnc-NUB1-1	lnc-NUB1-1:4	Downregulated
A_23_P143	0.04558	0.0215	-1.35	ATP6V1E2	ATPase, H+ transporting, lysosomal 31kDa, V1 subunit E2	Downregulated

A_23_P215	0.00124	0.000126	-1.36	IGFBP3	insulin-like growth factor binding protein 3	Downregulated
A_21_P000	0.001375	0.000153	-1.36	SNORD43	small nucleolar RNA, C/D box 43	Downregulated
A_21_P001	0.001391	0.000156	-1.36	LOC1019273	uncharacterized LOC101927372	Downregulated
A_24_P242	0.00151	0.000183	-1.36	ZRANB2	zinc finger, RAN-binding domain containing 2	Downregulated
A_33_P324	0.001626	0.000208	-1.36	MPP5	membrane protein, palmitoylated 5 (MAGUK p55 subfamily member 5)	Downregulated
A_22_P000	0.001772	0.000242	-1.36	COQ9	coenzyme Q9	Downregulated
A_33_P336	0.002016	0.000305	-1.36	IFNAR2	interferon (alpha, beta and omega) receptor 2	Downregulated
A_23_P925	0.002243	0.000366	-1.36	SCFD2	sec1 family domain containing 2	Downregulated
A_24_P385	0.002327	0.000385	-1.36	SP100	SP100 nuclear antigen	Downregulated
A_33_P367	0.002752	0.000498	-1.36	VPS36	vacuolar protein sorting 36 homolog (S. cerevisiae)	Downregulated
A_33_P339	0.003826	0.000819	-1.36	TP53I3	tumor protein p53 inducible protein 3	Downregulated
A_33_P321	0.005855	0.00153	-1.36	ERN2	endoplasmic reticulum to nucleus signaling 2	Downregulated
A_24_P210	0.006362	0.00172	-1.36	C11orf71	chromosome 11 open reading frame 71	Downregulated
A_22_P000	0.00714	0.00203	-1.36	lnc-MRPL40-2	lnc-MRPL40-2:1	Downregulated
A_22_P000	0.010862	0.00355	-1.36	lnc-POLR1E-2	lnc-POLR1E-2:1	Downregulated
A_23_P391	0.012359	0.00421	-1.36	RUVBL2	RuvB-like AAA ATPase 2	Downregulated
A_24_P123	0.026335	0.011	-1.36	HNRNPD	heterogeneous nuclear ribonucleoprotein D (AU-rich element RNA bindi	Downregulated
A_33_P323	0.063842	0.0322	-1.36	RMND5A	required for meiotic nuclear division 5 homolog A (S. cerevisiae)	Downregulated
A_23_P314	0.00124	0.000127	-1.37	PRKAG2	protein kinase, AMP-activated, gamma 2 non-catalytic subunit	Downregulated
A_33_P333	0.001245	0.000128	-1.37	RPA2	replication protein A2, 32kDa	Downregulated
A_24_P217	0.001304	0.00014	-1.37	SLC3A1	solute carrier family 3 (amino acid transporter heavy chain), member 1	Downregulated
A_23_P385	0.001314	0.000141	-1.37	STAMBPL1	STAM binding protein-like 1	Downregulated
A_33_P339	0.001342	0.000147	-1.37	TLK2	tousled-like kinase 2	Downregulated
A_23_P269	0.001376	0.000153	-1.37	POLG2	polymerase (DNA directed), gamma 2, accessory subunit	Downregulated
A_32_P989	0.001544	0.00019	-1.37	C15orf57	chromosome 15 open reading frame 57	Downregulated
A_33_P338	0.001767	0.000241	-1.37	CXCR3	chemokine (C-X-C motif) receptor 3	Downregulated
A_33_P682	0.002006	0.000302	-1.37	NFATC3	nuclear factor of activated T-cells, cytoplasmic, calcineurin-dependent 3	Downregulated
A_32_P117	0.002006	0.000302	-1.37	PMS2P4	postmeiotic segregation increased 2 pseudogene 4	Downregulated
A_22_P000	0.002607	0.000459	-1.37	ERN1	endoplasmic reticulum to nucleus signaling 1	Downregulated
A_24_P188	0.003064	0.000587	-1.37	VPS18	vacuolar protein sorting 18 homolog (S. cerevisiae)	Downregulated
A_33_P349	0.003158	0.000616	-1.37	TMEM65	transmembrane protein 65	Downregulated
A_24_P268	0.004312	0.000977	-1.37	DRAM2	DNA-damage regulated autophagy modulator 2	Downregulated
A_21_P000	0.005097	0.00125	-1.37	lnc-VN1R2-1	lnc-VN1R2-1:2	Downregulated
A_23_P545	0.005204	0.00129	-1.37	TYRO3	TYRO3 protein tyrosine kinase	Downregulated
A_33_P323	0.006202	0.00166	-1.37	TGFB1I1	transforming growth factor beta 1 induced transcript 1	Downregulated
A_24_P576	0.006513	0.00178	-1.37	SPRN	shadow of prion protein homolog (zebrafish)	Downregulated
A_21_P001	0.006967	0.00196	-1.37	XLOC_l2_004854		Downregulated

A_21_P000	0.008371	0.00252	-1.37	LOC1019287	uncharacterized LOC101928767	Downregulated
A_33_P321	0.008877	0.00272	-1.37	RAP1GDS1	RAP1, GTP-GDP dissociation stimulator 1	Downregulated
A_23_P250	0.011589	0.00388	-1.37	RAB1A	RAB1A, member RAS oncogene family	Downregulated
A_22_P000	0.020456	0.00799	-1.37	CASP8AP2	caspase 8 associated protein 2	Downregulated
A_23_P344	0.026279	0.011	-1.37	OGG1	8-oxoguanine DNA glycosylase	Downregulated
A_33_P330	0.042003	0.0195	-1.37	PDIK1L	PDLIM1 interacting kinase 1 like	Downregulated
A_23_P219	0.001214	0.000122	-1.38	ABCC10	ATP-binding cassette, sub-family C (CFTR/MRP), member 10	Downregulated
A_21_P000	0.001222	0.000123	-1.38	LINC00629	long intergenic non-protein coding RNA 629	Downregulated
A_23_P128	0.001244	0.000128	-1.38	ZFYVE1	zinc finger, FYVE domain containing 1	Downregulated
A_23_P888	0.001258	0.000131	-1.38	CMTM3	CKLF-like MARVEL transmembrane domain containing 3	Downregulated
A_23_P170	0.001268	0.000133	-1.38	PMCHL1	pro-melanin-concentrating hormone-like 1, pseudogene	Downregulated
A_24_P350	0.001324	0.000143	-1.38	KIAA0907	KIAA0907	Downregulated
A_19_P008	0.001394	0.000157	-1.38	LINC01215	long intergenic non-protein coding RNA 1215	Downregulated
A_33_P322	0.001433	0.000165	-1.38	SCPEP1	serine carboxypeptidase 1	Downregulated
A_33_P334	0.001576	0.000197	-1.38	C16orf13	chromosome 16 open reading frame 13	Downregulated
A_24_P852	0.001603	0.000202	-1.38	ELMSAN1	ELM2 and Myb/SANT-like domain containing 1	Downregulated
A_23_P363	0.001611	0.000204	-1.38	CGGBP1	CGG triplet repeat binding protein 1	Downregulated
A_23_P148	0.001802	0.000249	-1.38	CUL4B	cullin 4B	Downregulated
A_22_P000	0.0018	0.000249	-1.38	lnc-FBXO25-2	lnc-FBXO25-2:1	Downregulated
A_21_P000	0.002243	0.000366	-1.38	PDCD6IP	programmed cell death 6 interacting protein	Downregulated
A_23_P251	0.002243	0.000366	-1.38	TEX261	testis expressed 261	Downregulated
A_32_P144	0.002872	0.00053	-1.38	PARP4	poly (ADP-ribose) polymerase family, member 4	Downregulated
A_22_P000	0.003109	0.000602	-1.38	C21orf2	chromosome 21 open reading frame 2	Downregulated
A_21_P000	0.008213	0.00245	-1.38	lnc-ZSWIM2-4	lnc-ZSWIM2-4:1	Downregulated
A_33_P336	0.01315	0.00455	-1.38	BAGE	B melanoma antigen	Downregulated
A_22_P000	0.013209	0.00458	-1.38	lnc-OLR1-2	lnc-OLR1-2:1	Downregulated
A_21_P001	0.014311	0.00509	-1.38	XLOC_l2_004180		Downregulated
A_33_P666	0.018713	0.00716	-1.38	PRIM2	primase, DNA, polypeptide 2 (58kDa)	Downregulated
A_23_P771	0.025006	0.0103	-1.38	TM6SF1	transmembrane 6 superfamily member 1	Downregulated
A_24_P405	0.028067	0.0119	-1.38	PDIK1L	PDLIM1 interacting kinase 1 like	Downregulated
A_23_P687	0.029266	0.0125	-1.38	PDXK	pyridoxal (pyridoxine, vitamin B6) kinase	Downregulated
A_22_P000	0.053737	0.0262	-1.38	lnc-ACCSL-1	lnc-ACCSL-1:1	Downregulated
A_22_P000	0.063259	0.0318	-1.38	lnc-MIER3-2	lnc-MIER3-2:1	Downregulated
A_21_P001	0.00121	0.000121	-1.39	PRDX6	peroxiredoxin 6	Downregulated
A_24_P418	0.001215	0.000122	-1.39	MACF1	microtubule-actin crosslinking factor 1	Downregulated
A_33_P337	0.001324	0.000143	-1.39	CHAC1	ChaC glutathione-specific gamma-glutamylcyclotransferase 1	Downregulated
A_23_P129	0.001534	0.000187	-1.39	USP8	ubiquitin specific peptidase 8	Downregulated

A_33_P339	0.001688	0.000222	-1.39	ACOXL	acyl-CoA oxidase-like	Downregulated
A_23_P103	0.001726	0.000231	-1.39	PSEN2	presenilin 2	Downregulated
A_22_P000	0.002211	0.000356	-1.39	lnc-CTU2-2	lnc-CTU2-2:1	Downregulated
A_23_P144	0.002564	0.000447	-1.39	NAP1L5	nucleosome assembly protein 1-like 5	Downregulated
A_24_P294	0.003518	0.000723	-1.39	ATXN1	ataxin 1	Downregulated
A_33_P323	0.006124	0.00163	-1.39	CDC14B	cell division cycle 14B	Downregulated
A_24_P108	0.0097	0.00307	-1.39	IMPACT	impact RWD domain protein	Downregulated
A_23_P171	0.021169	0.00833	-1.39	MORC4	MORC family CW-type zinc finger 4	Downregulated
A_21_P001	0.034462	0.0153	-1.39	FAM220A	family with sequence similarity 220, member A	Downregulated
A_23_P162	0.048135	0.0229	-1.39	IRAK3	interleukin-1 receptor-associated kinase 3	Downregulated
A_23_P962	0.052171	0.0252	-1.39	REEP4	receptor accessory protein 4	Downregulated
A_23_P252	0.052391	0.0254	-1.39	MTRR	5-methyltetrahydrofolate-homocysteine methyltransferase reductase	Downregulated
A_33_P339	0.055883	0.0274	-1.39	UBXN11	UBX domain protein 11	Downregulated
A_33_P333	0.001492	0.000179	-1.4	OPTN	optineurin	Downregulated
A_23_P216	0.001544	0.00019	-1.4	SUSD1	sushi domain containing 1	Downregulated
A_32_P193	0.001566	0.000195	-1.4	RICTOR	RPTOR independent companion of MTOR, complex 2	Downregulated
A_23_P356	0.001601	0.000201	-1.4	DESI1	desumoylating isopeptidase 1	Downregulated
A_33_P326	0.001711	0.000229	-1.4	CCDC167	coiled-coil domain containing 167	Downregulated
A_33_P353	0.001815	0.000253	-1.4	LARS	leucyl-tRNA synthetase	Downregulated
A_23_P654	0.001813	0.000253	-1.4	ZBTB17	zinc finger and BTB domain containing 17	Downregulated
A_24_P244	0.001888	0.000271	-1.4	MCTP2	multiple C2 domains, transmembrane 2	Downregulated
A_33_P329	0.00189	0.000271	-1.4	TRA2B	transformer 2 beta homolog (Drosophila)	Downregulated
A_33_P321	0.002006	0.000302	-1.4	ADAMTS7	ADAM metallopeptidase with thrombospondin type 1 motif, 7	Downregulated
A_32_P185	0.002107	0.000329	-1.4	YBEY	ybeY metallopeptidase (putative)	Downregulated
A_32_P237	0.002124	0.000336	-1.4	XLOC_l2_010916		Downregulated
A_23_P913	0.002785	0.000506	-1.4	GPCPD1	glycerophosphocholine phosphodiesterase GDE1 homolog (S. cerevisia	Downregulated
A_23_P165	0.002889	0.000535	-1.4	ILVBL	ilvB (bacterial acetolactate synthase)-like	Downregulated
A_23_P117	0.005348	0.00134	-1.4	LETMD1	LETM1 domain containing 1	Downregulated
A_23_P151	0.006398	0.00174	-1.4	FOXM1	forkhead box M1	Downregulated
A_33_P324	0.006768	0.00188	-1.4	EBF1	early B-cell factor 1	Downregulated
A_23_P208	0.011043	0.00363	-1.4	AKT2	v-akt murine thymoma viral oncogene homolog 2	Downregulated
A_23_P314	0.012756	0.00438	-1.4	ZDHHC19	zinc finger, DHHC-type containing 19	Downregulated
A_19_P008	0.013059	0.00451	-1.4	lnc-ITSN1-2	lnc-ITSN1-2:5	Downregulated
A_24_P937	0.013516	0.00472	-1.4	LOC645984	uncharacterized LOC645984	Downregulated
A_23_P422	0.013516	0.00472	-1.4	SLC35F3	solute carrier family 35, member F3	Downregulated
A_22_P000	0.016638	0.00619	-1.4	TTN-AS1	TTN antisense RNA 1	Downregulated
A_33_P382	0.020419	0.00797	-1.4	CCDC112	coiled-coil domain containing 112	Downregulated

A_23_P308	0.028784	0.0123	-1.4	TTC39C	tetratricopeptide repeat domain 39C	Downregulated
A_24_P340	0.031758	0.0138	-1.4	SERHL2	serine hydrolase-like 2	Downregulated
A_33_P331	0.03786	0.0172	-1.4	lnc-LIN28B-1	lnc-LIN28B-1:4	Downregulated
A_24_P336	0.053672	0.0261	-1.4	C22orf23	chromosome 22 open reading frame 23	Downregulated
A_22_P000	0.053672	0.0261	-1.4	LOC1001322	uncharacterized LOC100132215	Downregulated
A_23_P270	0.064702	0.0327	-1.4	LPAR6	lysophosphatidic acid receptor 6	Downregulated
A_23_P367	0.079233	0.0416	-1.4	ALDH2	aldehyde dehydrogenase 2 family (mitochondrial)	Downregulated
A_22_P000	0.001178	0.000114	-1.41	lnc-COLQ-1	lnc-COLQ-1:1	Downregulated
A_23_P399	0.001198	0.000118	-1.41	IL12RB1	interleukin 12 receptor, beta 1	Downregulated
A_23_P134	0.00146	0.000172	-1.41	COG5	component of oligomeric golgi complex 5	Downregulated
A_23_P408	0.001581	0.000198	-1.41	CHST2	carbohydrate (N-acetylglucosamine-6-O) sulfotransferase 2	Downregulated
A_33_P321	0.001711	0.000229	-1.41	RUNX1	runt-related transcription factor 1	Downregulated
A_24_P409	0.001859	0.000264	-1.41	NPEPPS	aminopeptidase puromycin sensitive	Downregulated
A_24_P252	0.002034	0.00031	-1.41	NHLRC2	NHL repeat containing 2	Downregulated
A_23_P253	0.002459	0.000417	-1.41	CENPE	centromere protein E, 312kDa	Downregulated
A_32_P228	0.002597	0.000456	-1.41	GAPVD1	GTPase activating protein and VPS9 domains 1	Downregulated
A_33_P337	0.003439	0.0007	-1.41	FBXO32	F-box protein 32	Downregulated
A_33_P324	0.00391	0.000846	-1.41	AGO3	argonaute RISC catalytic component 3	Downregulated
A_24_P212	0.003973	0.000864	-1.41	ZNF33A	zinc finger protein 33A	Downregulated
A_23_P374	0.004031	0.000882	-1.41	SH3KBP1	SH3-domain kinase binding protein 1	Downregulated
A_33_P335	0.005304	0.00133	-1.41	ZNF639	zinc finger protein 639	Downregulated
A_21_P001	0.006445	0.00176	-1.41	LOC1019297	uncharacterized LOC101929787	Downregulated
A_21_P001	0.007457	0.00215	-1.41	lnc-C21orf63-	lnc-C21orf63-2:2	Downregulated
A_33_P325	0.007707	0.00225	-1.41	ANKS1B	ankyrin repeat and sterile alpha motif domain containing 1B	Downregulated
A_19_P003	0.008533	0.00258	-1.41	LINC01278	long intergenic non-protein coding RNA 1278	Downregulated
A_24_P193	0.014153	0.00501	-1.41	RAB15	RAB15, member RAS oncogene family	Downregulated
A_33_P337	0.014169	0.00502	-1.41	CLIC4	chloride intracellular channel 4	Downregulated
A_22_P000	0.015189	0.00551	-1.41	lnc-SHISA9-1	lnc-SHISA9-1:1	Downregulated
A_24_P225	0.017171	0.00644	-1.41	DPH3	diphthamide biosynthesis 3	Downregulated
A_22_P000	0.038453	0.0175	-1.41	TM4SF19-AS	TM4SF19 antisense RNA 1	Downregulated
A_22_P000	0.065863	0.0334	-1.41	lnc-BLID-3	lnc-BLID-3:2	Downregulated
A_23_P321	0.001349	0.000148	-1.42	CLECL1	C-type lectin-like 1	Downregulated
A_22_P000	0.001445	0.000168	-1.42	lnc-LPIN2-1	lnc-LPIN2-1:2	Downregulated
A_33_P320	0.001503	0.000181	-1.42	WDFY4	WDFY family member 4	Downregulated
A_23_P204	0.001548	0.000191	-1.42	AKAP11	A kinase (PRKA) anchor protein 11	Downregulated
A_33_P332	0.001611	0.000204	-1.42	CAPZA2	capping protein (actin filament) muscle Z-line, alpha 2	Downregulated
A_23_P112	0.001815	0.000253	-1.42	ATF7IP	activating transcription factor 7 interacting protein	Downregulated

A_23_P129	0.002243	0.000366	-1.42	PRDX5	peroxiredoxin 5	Downregulated
A_33_P328	0.004602	0.00108	-1.42	AAK1	AP2 associated kinase 1	Downregulated
A_24_P398	0.006867	0.00191	-1.42	EIF5	eukaryotic translation initiation factor 5	Downregulated
A_33_P339	0.008555	0.00259	-1.42	SPIN4	spindlin family, member 4	Downregulated
A_23_P152	0.008584	0.0026	-1.42	SLC16A6	solute carrier family 16, member 6	Downregulated
A_23_P126	0.011921	0.00402	-1.42	CLSPN	claspin	Downregulated
A_21_P000	0.01794	0.0068	-1.42	LOC440028	uncharacterized LOC440028	Downregulated
A_24_P392	0.020558	0.00804	-1.42	GLTPD2	glycolipid transfer protein domain containing 2	Downregulated
A_33_P322	0.034839	0.0155	-1.42	ZNF705B	zinc finger protein 705B	Downregulated
A_21_P000	0.037459	0.017	-1.42	LOC1025462	uncharacterized LOC102546298	Downregulated
A_33_P323	0.050072	0.024	-1.42	UROS	uroporphyrinogen III synthase	Downregulated
A_33_P332	0.056347	0.0277	-1.42	MAST2	microtubule associated serine/threonine kinase 2	Downregulated
A_23_P121	0.066933	0.034	-1.42	CAMK1	calcium/calmodulin-dependent protein kinase I	Downregulated
A_24_P985	0.001226	0.000124	-1.43	PPP3CB	protein phosphatase 3, catalytic subunit, beta isozyme	Downregulated
A_23_P308	0.001254	0.00013	-1.43	RRP36	ribosomal RNA processing 36 homolog (S. cerevisiae)	Downregulated
A_23_P164	0.001307	0.00014	-1.43	PSMC5	proteasome (prosome, macropain) 26S subunit, ATPase, 5	Downregulated
A_23_P191	0.001316	0.000142	-1.43	GSPT2	G1 to S phase transition 2	Downregulated
A_23_P162	0.001333	0.000144	-1.43	CCDC90B	coiled-coil domain containing 90B	Downregulated
A_21_P001	0.001328	0.000144	-1.43	INTS4	integrator complex subunit 4	Downregulated
A_33_P326	0.001533	0.000187	-1.43	MSL1	male-specific lethal 1 homolog (Drosophila)	Downregulated
A_23_P455	0.001785	0.000245	-1.43	SETBP1	SET binding protein 1	Downregulated
A_22_P000	0.00207	0.000319	-1.43	LOC1001309	uncharacterized LOC100130950	Downregulated
A_22_P000	0.002597	0.000456	-1.43	KCNMA1-AS2	KCNMA1 antisense RNA 2	Downregulated
A_23_P109	0.002597	0.000456	-1.43	TADA3	transcriptional adaptor 3	Downregulated
A_22_P000	0.00435	0.000992	-1.43	lnc-DYNLRB1	lnc-DYNLRB1-1:1	Downregulated
A_22_P000	0.00435	0.000992	-1.43	lnc-LPPR5.1-	lnc-LPPR5.1-1:1	Downregulated
A_23_P124	0.00435	0.000992	-1.43	NSD1	nuclear receptor binding SET domain protein 1	Downregulated
A_21_P001	0.004701	0.00111	-1.43	XLOC_l2_009469		Downregulated
A_22_P000	0.005023	0.00122	-1.43	lnc-ARF6-2	lnc-ARF6-2:1	Downregulated
A_21_P000	0.006446	0.00176	-1.43	LINC00662	long intergenic non-protein coding RNA 662	Downregulated
A_23_P229	0.007088	0.002	-1.43	HELB	helicase (DNA) B	Downregulated
A_33_P337	0.008871	0.00272	-1.43	MIER3	mesoderm induction early response 1, family member 3	Downregulated
A_23_P380	0.012265	0.00417	-1.43	R3HDM1	R3H domain containing 1	Downregulated
A_32_P900	0.017341	0.00652	-1.43	ARMC10	armadillo repeat containing 10	Downregulated
A_22_P000	0.02943	0.0126	-1.43	LINC00523	long intergenic non-protein coding RNA 523	Downregulated
A_33_P328	0.001134	0.000106	-1.44	MBIP	MAP3K12 binding inhibitory protein 1	Downregulated
A_32_P162	0.001187	0.000116	-1.44	ARHGAP18	Rho GTPase activating protein 18	Downregulated

A_23_P201	0.001186	0.000116	-1.44	FCRL5	Fc receptor-like 5	Downregulated
A_23_P111	0.00121	0.000121	-1.44	ATG5	autophagy related 5	Downregulated
A_33_P377	0.00122	0.000123	-1.44	PRKACB	protein kinase, cAMP-dependent, catalytic, beta	Downregulated
A_23_P384	0.001227	0.000124	-1.44	GYG1	glycogenin 1	Downregulated
A_33_P324	0.001245	0.000128	-1.44	LAMTOR2	late endosomal/lysosomal adaptor, MAPK and MTOR activator 2	Downregulated
A_33_P322	0.001445	0.000168	-1.44	HIPK2	homeodomain interacting protein kinase 2	Downregulated
A_23_P904	0.001548	0.00019	-1.44	RBM42	RNA binding motif protein 42	Downregulated
A_23_P112	0.001683	0.00022	-1.44	KIAA0196	KIAA0196	Downregulated
A_22_P000	0.001687	0.000221	-1.44	lnc-PPP1R1A	lnc-PPP1R1A-2:1	Downregulated
A_22_P000	0.001702	0.000227	-1.44	lnc-TUBB-7	lnc-TUBB-7:1	Downregulated
A_32_P140	0.001935	0.000282	-1.44	PMS2	PMS2 postmeiotic segregation increased 2 (S. cerevisiae)	Downregulated
A_24_P160	0.002374	0.000397	-1.44	MRPL55	mitochondrial ribosomal protein L55	Downregulated
A_23_P101	0.002538	0.00044	-1.44	BCAT2	branched chain amino-acid transaminase 2, mitochondrial	Downregulated
A_23_P551	0.003747	0.000794	-1.44	G6PC3	glucose 6 phosphatase, catalytic, 3	Downregulated
A_22_P000	0.004159	0.000924	-1.44	VIPR1-AS1	VIPR1 antisense RNA 1	Downregulated
A_23_P207	0.004658	0.0011	-1.44	CARD14	caspase recruitment domain family, member 14	Downregulated
A_21_P000	0.007914	0.00233	-1.44	RBM4	RNA binding motif protein 4	Downregulated
A_33_P336	0.00812	0.00241	-1.44	CRNDE	colorectal neoplasia differentially expressed (non-protein coding)	Downregulated
A_21_P001	0.015267	0.00554	-1.44	XLOC_l2_015536		Downregulated
A_33_P329	0.023179	0.00933	-1.44	TMEM175	transmembrane protein 175	Downregulated
A_23_P850	0.037403	0.0169	-1.44	RHOXF1	Rhox homeobox family, member 1	Downregulated
A_24_P659	0.046528	0.022	-1.44	UQCC1	ubiquinol-cytochrome c reductase complex assembly factor 1	Downregulated
A_24_P484	0.04934	0.0236	-1.44	CIDECP	cell death-inducing DFFA-like effector c pseudogene	Downregulated
A_33_P336	0.001108	0.000101	-1.45	PPP1R13B	protein phosphatase 1, regulatory subunit 13B	Downregulated
A_23_P494	0.001117	0.000103	-1.45	NUDT16L1	nudix (nucleoside diphosphate linked moiety X)-type motif 16-like 1	Downregulated
A_23_P522	0.001149	0.000109	-1.45	IFIT1	interferon-induced protein with tetratricopeptide repeats 1	Downregulated
A_33_P681	0.00124	0.000127	-1.45	LOC1005068	uncharacterized LOC100506844	Downregulated
A_33_P335	0.001243	0.000128	-1.45	ING3	inhibitor of growth family, member 3	Downregulated
A_24_P329	0.00125	0.000129	-1.45	BTN3A1	butyrophilin, subfamily 3, member A1	Downregulated
A_23_P165	0.001302	0.000139	-1.45	LOC1001299	lectin, galactoside-binding, soluble, 14 pseudogene	Downregulated
A_33_P321	0.001369	0.000151	-1.45	ZNF684	zinc finger protein 684	Downregulated
A_24_P541	0.001375	0.000152	-1.45	TNFRSF1B	tumor necrosis factor receptor superfamily, member 1B	Downregulated
A_24_P242	0.001453	0.00017	-1.45	TSN	translin	Downregulated
A_22_P000	0.001492	0.000179	-1.45	MED6	mediator complex subunit 6	Downregulated
A_33_P335	0.001565	0.000195	-1.45	CCDC69	coiled-coil domain containing 69	Downregulated
A_23_P395	0.001672	0.000218	-1.45	RSC1A1	regulatory solute carrier protein, family 1, member 1	Downregulated
A_33_P328	0.001922	0.000279	-1.45	COPS8	COP9 signalosome subunit 8	Downregulated

A_33_P329	0.001992	0.000298	-1.45	SLC12A8	solute carrier family 12, member 8	Downregulated
A_21_P000	0.00289	0.000536	-1.45	lnc-RAB11FIF	lnc-RAB11FIP2-2:3	Downregulated
A_24_P329	0.003154	0.000614	-1.45	UBQLN1	ubiquilin 1	Downregulated
A_33_P338	0.003189	0.000626	-1.45	POMGNT1	protein O-linked mannose N-acetylglucosaminyltransferase 1 (beta 1,2-)	Downregulated
A_23_P540	0.003316	0.000664	-1.45	THTPA	thiamine triphosphatase	Downregulated
A_23_P893	0.003559	0.000736	-1.45	EPN2	epsin 2	Downregulated
A_23_P319	0.003579	0.000744	-1.45	NR1I3	nuclear receptor subfamily 1, group I, member 3	Downregulated
A_33_P328	0.003791	0.000807	-1.45	LOC1006531	golgin subfamily A member 6-like protein 1-like	Downregulated
A_22_P000	0.00399	0.000871	-1.45	MED4-AS1	MED4 antisense RNA 1	Downregulated
A_22_P000	0.004058	0.000891	-1.45	lnc-C9orf66-3	lnc-C9orf66-3:1	Downregulated
A_23_P142	0.005696	0.00147	-1.45	PRPF31	pre-mRNA processing factor 31	Downregulated
A_23_P115	0.009857	0.00313	-1.45	FAM204A	family with sequence similarity 204, member A	Downregulated
A_33_P332	0.010989	0.0036	-1.45	TTN	titin	Downregulated
A_33_P339	0.016381	0.00607	-1.45	BCL2L11	BCL2-like 11 (apoptosis facilitator)	Downregulated
A_21_P000	0.023517	0.00951	-1.45	lnc-MFSD8-2	lnc-MFSD8-2:1	Downregulated
A_21_P000	0.026793	0.0112	-1.45	LOC1027237	uncharacterized LOC102723765	Downregulated
A_33_P324	0.039717	0.0182	-1.45	MAVS	mitochondrial antiviral signaling protein	Downregulated
A_22_P000	0.039717	0.0182	-1.45	NRSN2-AS1	NRSN2 antisense RNA 1	Downregulated
A_33_P327	0.041752	0.0193	-1.45	RAD1	RAD1 checkpoint DNA exonuclease	Downregulated
A_23_P281	0.001181	0.000115	-1.46	ARL6IP6	ADP-ribosylation factor-like 6 interacting protein 6	Downregulated
A_24_P943	0.001543	0.000189	-1.46	NR1D2	nuclear receptor subfamily 1, group D, member 2	Downregulated
A_22_P000	0.001691	0.000223	-1.46	LOC1019272	uncharacterized LOC101927272	Downregulated
A_23_P259	0.001706	0.000228	-1.46	THNSL2	threonine synthase-like 2 (S. cerevisiae)	Downregulated
A_24_P917	0.001749	0.000236	-1.46	LOC284912	uncharacterized LOC284912	Downregulated
A_32_P216	0.002072	0.00032	-1.46	SPDYE3	speedy/RINGO cell cycle regulator family member E3	Downregulated
A_23_P151	0.002302	0.000379	-1.46	UBFD1	ubiquitin family domain containing 1	Downregulated
A_33_P322	0.0025	0.00043	-1.46	MED15P9	mediator complex subunit 15 pseudogene 9	Downregulated
A_23_P208	0.003074	0.000589	-1.46	MBOAT7	membrane bound O-acyltransferase domain containing 7	Downregulated
A_22_P000	0.003267	0.000649	-1.46	lnc-NID1-3	lnc-NID1-3:1	Downregulated
A_23_P376	0.006339	0.00171	-1.46	GOLGA8A	golgin A8 family, member A	Downregulated
A_23_P131	0.008293	0.00249	-1.46	C2orf81	chromosome 2 open reading frame 81	Downregulated
A_23_P753	0.00968	0.00306	-1.46	ARHGAP22	Rho GTPase activating protein 22	Downregulated
A_23_P129	0.033467	0.0147	-1.46	SDR42E1	short chain dehydrogenase/reductase family 42E, member 1	Downregulated
A_24_P410	0.043926	0.0205	-1.46	BCL2L13	BCL2-like 13 (apoptosis facilitator)	Downregulated
A_23_P120	0.001117	0.000103	-1.47	SP110	SP110 nuclear body protein	Downregulated
A_23_P870	0.001187	0.000116	-1.47	TAGLN	transgelin	Downregulated
A_23_P428	0.001193	0.000117	-1.47	SND1	staphylococcal nuclease and tudor domain containing 1	Downregulated

A_24_P410	0.001341	0.000146	-1.47	RITA1	RBPJ interacting and tubulin associated 1	Downregulated
A_32_P438	0.001394	0.000157	-1.47	DCUN1D4	DCN1, defective in cullin neddylation 1, domain containing 4	Downregulated
A_33_P336	0.001571	0.000196	-1.47	PTPRC	protein tyrosine phosphatase, receptor type, C	Downregulated
A_23_P108	0.001626	0.000208	-1.47	CREB3L3	cAMP responsive element binding protein 3-like 3	Downregulated
A_23_P502	0.001697	0.000225	-1.47	MRPL47	mitochondrial ribosomal protein L47	Downregulated
A_23_P705	0.001778	0.000243	-1.47	HSPA1L	heat shock 70kDa protein 1-like	Downregulated
A_23_P382	0.001955	0.000288	-1.47	PRPF8	pre-mRNA processing factor 8	Downregulated
A_23_P114	0.002238	0.000364	-1.47	MTPAP	mitochondrial poly(A) polymerase	Downregulated
A_33_P338	0.002748	0.000496	-1.47	SHARPIN	SHANK-associated RH domain interactor	Downregulated
A_23_P137	0.002984	0.000563	-1.47	CHI3L1	chitinase 3-like 1 (cartilage glycoprotein-39)	Downregulated
A_23_P418	0.00314	0.00061	-1.47	CARD6	caspase recruitment domain family, member 6	Downregulated
A_33_P326	0.003157	0.000616	-1.47	ANKRD36B	ankyrin repeat domain 36B	Downregulated
A_33_P332	0.003529	0.000726	-1.47	IRF3	interferon regulatory factor 3	Downregulated
A_23_P134	0.005519	0.0014	-1.47	PTCD1	pentatricopeptide repeat domain 1	Downregulated
A_21_P001	0.007314	0.00209	-1.47	lnc-NBPF3-4	lnc-NBPF3-4:1	Downregulated
A_33_P324	0.009198	0.00286	-1.47	DNAJC11	DnaJ (Hsp40) homolog, subfamily C, member 11	Downregulated
A_33_P321	0.022876	0.00918	-1.47	ANAPC16	anaphase promoting complex subunit 16	Downregulated
A_22_P000	0.022876	0.00918	-1.47	lnc-CCNJL-1	lnc-CCNJL-1:2	Downregulated
A_33_P335	0.02543	0.0105	-1.47	ZBP1	Z-DNA binding protein 1	Downregulated
A_33_P334	0.053098	0.0258	-1.47	ZNF578	zinc finger protein 578	Downregulated
A_33_P340	0.084425	0.045	-1.47	SORBS2	sorbin and SH3 domain containing 2	Downregulated
A_23_P157	0.001095	0.0000979	-1.48	PSMB6	proteasome (prosome, macropain) subunit, beta type, 6	Downregulated
A_21_P000	0.001379	0.000154	-1.48	lnc-MFAP4-4	lnc-MFAP4-4:1	Downregulated
A_23_P111	0.001474	0.000175	-1.48	PARP12	poly (ADP-ribose) polymerase family, member 12	Downregulated
A_24_P332	0.001816	0.000253	-1.48	GUSBP1	glucuronidase, beta pseudogene 1	Downregulated
A_33_P323	0.001841	0.00026	-1.48	MIEF1	mitochondrial elongation factor 1	Downregulated
A_21_P000	0.002943	0.00055	-1.48	lnc-SGCG-5	lnc-SGCG-5:1	Downregulated
A_32_P219	0.00376	0.000798	-1.48	CENPJ	centromere protein J	Downregulated
A_33_P329	0.004843	0.00116	-1.48	NTM	neurotrimin	Downregulated
A_33_P326	0.006501	0.00178	-1.48	DUS1L	dihydrouridine synthase 1-like (S. cerevisiae)	Downregulated
A_24_P914	0.008489	0.00256	-1.48	MEF2BNB	MEF2B neighbor	Downregulated
A_23_P206	0.008651	0.00263	-1.48	SEC14L1	SEC14-like 1 (S. cerevisiae)	Downregulated
A_33_P322	0.009425	0.00295	-1.48	RRBP1	ribosome binding protein 1	Downregulated
A_22_P000	0.015522	0.00566	-1.48	LOC1001287	uncharacterized LOC100128714	Downregulated
A_22_P000	0.015957	0.00586	-1.48	lnc-LEPRE1-1	lnc-LEPRE1-1:1	Downregulated
A_23_P855	0.046873	0.0222	-1.48	GPR52	G protein-coupled receptor 52	Downregulated
A_23_P775	0.048094	0.0229	-1.48	VAC14	Vac14 homolog (S. cerevisiae)	Downregulated

A_33_P331	0.001098	0.0000987	-1.49	UBAP2L	ubiquitin associated protein 2-like	Downregulated
A_23_P126	0.001146	0.000108	-1.49	SH3BGRL3	SH3 domain binding glutamate-rich protein like 3	Downregulated
A_33_P655	0.001222	0.000123	-1.49	ZNF518A	zinc finger protein 518A	Downregulated
A_23_P769	0.001316	0.000142	-1.49	SIPA1L1	signal-induced proliferation-associated 1 like 1	Downregulated
A_23_P623	0.001369	0.000151	-1.49	TAZ	tafazzin	Downregulated
A_23_P161	0.001479	0.000176	-1.49	ELK4	ELK4, ETS-domain protein (SRF accessory protein 1)	Downregulated
A_22_P000	0.001763	0.00024	-1.49	ANKRD36BP2	ankyrin repeat domain 36B pseudogene 2	Downregulated
A_33_P382	0.002117	0.000332	-1.49	LINC00857	long intergenic non-protein coding RNA 857	Downregulated
A_23_P108	0.002338	0.000387	-1.49	CNOT11	CCR4-NOT transcription complex, subunit 11	Downregulated
A_22_P000	0.003346	0.000673	-1.49	lnc-COL9A1-1	lnc-COL9A1-1:1	Downregulated
A_24_P219	0.00339	0.000685	-1.49	PTOV1	prostate tumor overexpressed 1	Downregulated
A_33_P329	0.004588	0.00107	-1.49	NFS1	NFS1 cysteine desulfurase	Downregulated
A_23_P257	0.005067	0.00124	-1.49	MAPK1	mitogen-activated protein kinase 1	Downregulated
A_33_P330	0.006173	0.00165	-1.49	PIDD1	p53-induced death domain protein 1	Downregulated
A_23_P139	0.006941	0.00195	-1.49	CHST11	carbohydrate (chondroitin 4) sulfotransferase 11	Downregulated
A_23_P671	0.007077	0.002	-1.49	PIN1	peptidylprolyl cis/trans isomerase, NIMA-interacting 1	Downregulated
A_23_P909	0.010039	0.00321	-1.49	IL36B	interleukin 36, beta	Downregulated
A_24_P207	0.012808	0.0044	-1.49	UBE3A	ubiquitin protein ligase E3A	Downregulated
A_22_P000	0.015153	0.00549	-1.49	LINC00461	long intergenic non-protein coding RNA 461	Downregulated
A_21_P000	0.02252	0.009	-1.49	lnc-ARID2-3	lnc-ARID2-3:1	Downregulated
A_23_P165	0.02549	0.0105	-1.49	MPV17L2	MPV17 mitochondrial membrane protein-like 2	Downregulated
A_33_P321	0.041895	0.0194	-1.49	JPH4	junctophilin 4	Downregulated
A_24_P114	0.046941	0.0223	-1.49	MBOAT2	membrane bound O-acyltransferase domain containing 2	Downregulated
A_33_P334	0.05145	0.0248	-1.49	EXOSC2	exosome component 2	Downregulated
A_33_P337	0.001065	0.0000919	-1.5	SH3YL1	SH3 and SYLF domain containing 1	Downregulated
A_22_P000	0.00109	0.0000971	-1.5	lnc-NBPF3-4	lnc-NBPF3-4:1	Downregulated
A_33_P338	0.001145	0.000108	-1.5	C19orf54	chromosome 19 open reading frame 54	Downregulated
A_24_P245	0.001165	0.000112	-1.5	ATP5A1	ATP synthase, H+ transporting, mitochondrial F1 complex, alpha subuni	Downregulated
A_24_P245	0.001266	0.000133	-1.5	USP7	ubiquitin specific peptidase 7 (herpes virus-associated)	Downregulated
A_23_P128	0.001393	0.000156	-1.5	PSTK	phosphoseryl-tRNA kinase	Downregulated
A_23_P130	0.001626	0.000208	-1.5	MOB3A	MOB kinase activator 3A	Downregulated
A_21_P000	0.001817	0.000254	-1.5	lnc-GABRB3-	lnc-GABRB3-1:1	Downregulated
A_22_P000	0.002137	0.000339	-1.5	lnc-GLS-2	lnc-GLS-2:2	Downregulated
A_33_P338	0.004759	0.00113	-1.5	PXN	paxillin	Downregulated
A_22_P000	0.006123	0.00163	-1.5	lnc-MAPK6-9	lnc-MAPK6-9:1	Downregulated
A_23_P123	0.006795	0.00189	-1.5	APH1A	APH1A gamma secretase subunit	Downregulated
A_23_P150	0.006955	0.00195	-1.5	SEPHS1	selenophosphate synthetase 1	Downregulated

A_21_P001	0.027632	0.0117	-1.5	XLOC_l2_009811		Downregulated
A_33_P339	0.037331	0.0169	-1.5	RIT1	Ras-like without CAAX 1	Downregulated
A_33_P341	0.058429	0.0289	-1.5	ABI3BP	ABI family, member 3 (NESH) binding protein	Downregulated
A_23_P209	0.0011	0.0000992	-1.51	PSMD1	proteasome (prosome, macropain) 26S subunit, non-ATPase, 1	Downregulated
A_22_P000	0.001122	0.000104	-1.51	lnc-GPR126-1	lnc-GPR126-1:1	Downregulated
A_33_P327	0.001382	0.000154	-1.51	IFNAR1	interferon (alpha, beta and omega) receptor 1	Downregulated
A_23_P214	0.001475	0.000175	-1.51	PBX2	pre-B-cell leukemia homeobox 2	Downregulated
A_33_P335	0.001477	0.000175	-1.51	PVRIG	poliovirus receptor related immunoglobulin domain containing	Downregulated
A_24_P398	0.001653	0.000214	-1.51	RGP1	RGP1 retrograde golgi transport homolog (S. cerevisiae)	Downregulated
A_22_P000	0.001678	0.000219	-1.51	lnc-FAM211A	lnc-FAM211A-2:1	Downregulated
A_21_P000	0.001815	0.000253	-1.51	ZNF726	zinc finger protein 726	Downregulated
A_24_P345	0.002426	0.000409	-1.51	CYBRD1	cytochrome b reductase 1	Downregulated
A_23_P346	0.003631	0.000758	-1.51	CACNA2D2	calcium channel, voltage-dependent, alpha 2/delta subunit 2	Downregulated
A_23_P376	0.004602	0.00108	-1.51	NIT1	nitrilase 1	Downregulated
A_23_P389	0.007273	0.00207	-1.51	VAV1	vav 1 guanine nucleotide exchange factor	Downregulated
A_23_P943	0.007332	0.0021	-1.51	TSTA3	tissue specific transplantation antigen P35B	Downregulated
A_23_P705	0.008165	0.00243	-1.51	SDAD1	SDA1 domain containing 1	Downregulated
A_23_P155	0.015831	0.0058	-1.51	NAAA	N-acylethanolamine acid amidase	Downregulated
A_22_P000	0.016042	0.0059	-1.51	lnc-BARHL2-3	lnc-BARHL2-3:1	Downregulated
A_23_P501	0.0168	0.00627	-1.51	CSRP2BP	CSRP2 binding protein	Downregulated
A_21_P000	0.03265	0.0143	-1.51	lnc-PFKP-8	lnc-PFKP-8:1	Downregulated
A_23_P144	0.001054	0.0000892	-1.52	CAMK2D	calcium/calmodulin-dependent protein kinase II delta	Downregulated
A_23_P486	0.001086	0.0000961	-1.52	TMEM55B	transmembrane protein 55B	Downregulated
A_23_P693	0.001117	0.000103	-1.52	PARP9	poly (ADP-ribose) polymerase family, member 9	Downregulated
A_23_P206	0.001258	0.000131	-1.52	NIPSNAP3A	nipsnap homolog 3A (C. elegans)	Downregulated
A_24_P384	0.001291	0.000137	-1.52	EWSR1	EWS RNA-binding protein 1	Downregulated
A_23_P834	0.001405	0.00016	-1.52	SMARCC1	SWI/SNF related, matrix associated, actin dependent regulator of chrom	Downregulated
A_23_P533	0.001916	0.000278	-1.52	XRCC6BP1	XRCC6 binding protein 1	Downregulated
A_23_P428	0.002237	0.000363	-1.52	PDIA4	protein disulfide isomerase family A, member 4	Downregulated
A_21_P000	0.00284	0.000522	-1.52	lnc-MKI67IP-1	lnc-MKI67IP-1:3	Downregulated
A_23_P124	0.002907	0.000541	-1.52	DEAF1	DEAF1 transcription factor	Downregulated
A_23_P743	0.003187	0.000626	-1.52	OPN3	opsin 3	Downregulated
A_22_P000	0.00353	0.000726	-1.52	MANEA-AS1	MANEA antisense RNA 1 (head to head)	Downregulated
A_33_P331	0.003646	0.000762	-1.52	IMMT	inner membrane protein, mitochondrial	Downregulated
A_24_P174	0.003936	0.000853	-1.52	NNT	nicotinamide nucleotide transhydrogenase	Downregulated
A_24_P184	0.004114	0.000909	-1.52	PXN	paxillin	Downregulated
A_23_P122	0.004206	0.000941	-1.52	ELMO1	engulfment and cell motility 1	Downregulated

A_21_P001	0.006614	0.00182	-1.52	AMZ2P1	archaelysin family metallopeptidase 2 pseudogene 1	Downregulated
A_23_P345	0.00833	0.0025	-1.52	TICRR	TOPBP1-interacting checkpoint and replication regulator	Downregulated
A_33_P323	0.00833	0.0025	-1.52	TRIM45	tripartite motif containing 45	Downregulated
A_23_P132	0.008661	0.00264	-1.52	C22orf46	chromosome 22 open reading frame 46	Downregulated
A_21_P001	0.015108	0.00547	-1.52	XLOC_l2_005020		Downregulated
A_22_P000	0.018211	0.00693	-1.52	lnc-ATF7IP2-2	lnc-ATF7IP2-2:1	Downregulated
A_21_P000	0.021294	0.00839	-1.52	HDHD1	haloacid dehalogenase-like hydrolase domain containing 1	Downregulated
A_21_P000	0.024515	0.01	-1.52	SNORA60	small nucleolar RNA, H/ACA box 60	Downregulated
A_19_P003	0.044371	0.0208	-1.52	LOC1019280	uncharacterized LOC101928004	Downregulated
A_33_P323	0.058458	0.0289	-1.52	ATXN3L	ataxin 3-like	Downregulated
A_24_P253	0.001067	0.0000928	-1.53	EMG1	EMG1 N1-specific pseudouridine methyltransferase	Downregulated
A_23_P256	0.001097	0.0000983	-1.53	TSSC1	tumor suppressing subtransferable candidate 1	Downregulated
A_23_P159	0.0011	0.000099	-1.53	COX7B	cytochrome c oxidase subunit VIIb	Downregulated
A_33_P332	0.001295	0.000138	-1.53	CDK13	cyclin-dependent kinase 13	Downregulated
A_33_P326	0.001443	0.000167	-1.53	RASA3	RAS p21 protein activator 3	Downregulated
A_23_P488	0.001492	0.000179	-1.53	KIF23	kinesin family member 23	Downregulated
A_24_P149	0.001791	0.000246	-1.53	ZBTB43	zinc finger and BTB domain containing 43	Downregulated
A_21_P001	0.001863	0.000265	-1.53	LINC00665	long intergenic non-protein coding RNA 665	Downregulated
A_22_P000	0.002029	0.000309	-1.53	lnc-ABCC3-1	lnc-ABCC3-1:1	Downregulated
A_23_P205	0.002364	0.000394	-1.53	ANKRD10	ankyrin repeat domain 10	Downregulated
A_23_P215	0.002794	0.000508	-1.53	NUB1	negative regulator of ubiquitin-like proteins 1	Downregulated
A_23_P283	0.003171	0.000621	-1.53	GMCL1	germ cell-less, spermatogenesis associated 1	Downregulated
A_22_P000	0.003193	0.000628	-1.53	lnc-RP11-728	lnc-RP11-728F11.4.1-1:3	Downregulated
A_23_P333	0.004094	0.000902	-1.53	NEK3	NIMA-related kinase 3	Downregulated
A_19_P008	0.005715	0.00148	-1.53	XLOC_l2_010511		Downregulated
A_24_P184	0.006166	0.00165	-1.53	LRTOMT	leucine rich transmembrane and O-methyltransferase domain containing	Downregulated
A_23_P103	0.006203	0.00166	-1.53	AGMAT	agmatine ureohydrolase (agmatinase)	Downregulated
A_23_P957	0.006202	0.00166	-1.53	ITLN1	intelectin 1 (galactofuranose binding)	Downregulated
A_22_P000	0.00833	0.0025	-1.53	lnc-WDR45L-2	lnc-WDR45L-2:1	Downregulated
A_33_P329	0.008991	0.00277	-1.53	lnc-MLL3-1	lnc-MLL3-1:1	Downregulated
A_24_P178	0.011221	0.00371	-1.53	PHLDB2	pleckstrin homology-like domain, family B, member 2	Downregulated
A_33_P336	0.019258	0.00743	-1.53	LBH	limb bud and heart development	Downregulated
A_22_P000	0.021061	0.00828	-1.53	NEURL4	neuralized E3 ubiquitin protein ligase 4	Downregulated
A_21_P000	0.021697	0.00859	-1.53	LINC01482	long intergenic non-protein coding RNA 1482	Downregulated
A_23_P115	0.03678	0.0166	-1.53	FCRL4	Fc receptor-like 4	Downregulated
A_33_P334	0.001014	0.0000822	-1.54	ATG2B	autophagy related 2B	Downregulated
A_22_P000	0.001092	0.0000973	-1.54	lnc-RAB30-1	lnc-RAB30-1:1	Downregulated

A_33_P335	0.001098	0.0000985	-1.54	BTLA	B and T lymphocyte associated	Downregulated
A_33_P329	0.001443	0.000168	-1.54	LUC7L3	LUC7-like 3 (S. cerevisiae)	Downregulated
A_23_P336	0.001458	0.000172	-1.54	GLCCI1	glucocorticoid induced transcript 1	Downregulated
A_21_P000	0.001817	0.000254	-1.54	lnc-LRP1B-4	lnc-LRP1B-4:1	Downregulated
A_22_P000	0.001899	0.000273	-1.54	lnc-SUPT6H-1	lnc-SUPT6H-1:1	Downregulated
A_19_P003	0.001953	0.000287	-1.54	PVT1	Pvt1 oncogene (non-protein coding)	Downregulated
A_23_P363	0.002112	0.00033	-1.54	ATG16L2	autophagy related 16-like 2 (S. cerevisiae)	Downregulated
A_23_P385	0.003497	0.000716	-1.54	KRT16	keratin 16, type I	Downregulated
A_33_P341	0.009988	0.00319	-1.54	ZNF407	zinc finger protein 407	Downregulated
A_33_P326	0.02063	0.00807	-1.54	GAPVD1	GTPase activating protein and VPS9 domains 1	Downregulated
A_21_P001	0.0282	0.012	-1.54	XLOC_l2_001086		Downregulated
A_22_P000	0.047843	0.0228	-1.54	RGS5	regulator of G-protein signaling 5	Downregulated
A_21_P001	0.051179	0.0247	-1.54	LOC1019306	uncharacterized LOC101930634	Downregulated
A_23_P334	0.001021	0.0000834	-1.55	CRELD2	cysteine-rich with EGF-like domains 2	Downregulated
A_23_P413	0.001306	0.00014	-1.55	PREX1	phosphatidylinositol-3,4,5-trisphosphate-dependent Rac exchange facto	Downregulated
A_23_P128	0.001465	0.000173	-1.55	ARG2	arginase 2	Downregulated
A_33_P340	0.001478	0.000176	-1.55	TIMM23	translocase of inner mitochondrial membrane 23 homolog (yeast)	Downregulated
A_24_P419	0.001486	0.000178	-1.55	BICD2	bicaudal D homolog 2 (Drosophila)	Downregulated
A_32_P221	0.001486	0.000178	-1.55	HIST1H2AM	histone cluster 1, H2am	Downregulated
A_23_P121	0.001808	0.000251	-1.55	TMEM43	transmembrane protein 43	Downregulated
A_33_P327	0.001848	0.000261	-1.55	RIPK1	receptor (TNFRSF)-interacting serine-threonine kinase 1	Downregulated
A_22_P000	0.002008	0.000303	-1.55	lnc-STRA8-1	lnc-STRA8-1:1	Downregulated
A_23_P951	0.002163	0.000344	-1.55	SLC37A3	solute carrier family 37, member 3	Downregulated
A_33_P321	0.002476	0.000422	-1.55	EMR1	egf-like module containing, mucin-like, hormone receptor-like 1	Downregulated
A_22_P000	0.002572	0.00045	-1.55	LGALS17A	Charcot-Leyden crystal protein pseudogene	Downregulated
A_23_P149	0.003536	0.000729	-1.55	ABHD12	abhydrolase domain containing 12	Downregulated
A_23_P141	0.004135	0.000916	-1.55	CXXC1	CXXC finger protein 1	Downregulated
A_23_P129	0.009311	0.0029	-1.55	PALB2	partner and localizer of BRCA2	Downregulated
A_22_P000	0.009571	0.00301	-1.55	lnc-GGCT-1	lnc-GGCT-1:3	Downregulated
A_24_P425	0.014665	0.00526	-1.55	ACOT9	acyl-CoA thioesterase 9	Downregulated
A_22_P000	0.016175	0.00597	-1.55	lnc-RP11-43D	lnc-RP11-43D2.2.1-4:1	Downregulated
A_33_P330	0.02566	0.0106	-1.55	ZGLP1	zinc finger, GATA-like protein 1	Downregulated
A_23_P603	0.000995	0.0000781	-1.56	UBAC1	UBA domain containing 1	Downregulated
A_22_P000	0.001157	0.00011	-1.56	PLCG2	phospholipase C, gamma 2 (phosphatidylinositol-specific)	Downregulated
A_24_P148	0.001295	0.000137	-1.56	MST1	macrophage stimulating 1 (hepatocyte growth factor-like)	Downregulated
A_33_P322	0.001445	0.000168	-1.56	PTRH1	peptidyl-tRNA hydrolase 1 homolog (S. cerevisiae)	Downregulated
A_21_P000	0.001771	0.000242	-1.56	lnc-ADAMTS7	lnc-ADAMTS7-1:1	Downregulated

A_21_P001	0.002077	0.000321	-1.56	LOC1005074	uncharacterized LOC100507487	Downregulated
A_23_P714	0.002207	0.000355	-1.56	UBE2W	ubiquitin-conjugating enzyme E2W (putative)	Downregulated
A_33_P330	0.00247	0.00042	-1.56	VWA5A	von Willebrand factor A domain containing 5A	Downregulated
A_33_P330	0.002651	0.00047	-1.56	KMT2A	lysine (K)-specific methyltransferase 2A	Downregulated
A_23_P146	0.003126	0.000606	-1.56	STX8	syntaxin 8	Downregulated
A_23_P381	0.003164	0.000618	-1.56	CCDC74B	coiled-coil domain containing 74B	Downregulated
A_23_P125	0.003686	0.000775	-1.56	CASP7	caspase 7, apoptosis-related cysteine peptidase	Downregulated
A_21_P000	0.004039	0.000885	-1.56	GOLGA2P5	golgin A2 pseudogene 5	Downregulated
A_33_P333	0.004356	0.000995	-1.56	LOC1027252	chondroitin sulfate proteoglycan 4-like	Downregulated
A_23_P319	0.005643	0.00145	-1.56	EYA2	EYA transcriptional coactivator and phosphatase 2	Downregulated
A_23_P115	0.005735	0.00148	-1.56	PCGF6	polycomb group ring finger 6	Downregulated
A_23_P150	0.007125	0.00202	-1.56	SUOX	sulfite oxidase	Downregulated
A_33_P321	0.007348	0.0021	-1.56	LOC149373	uncharacterized LOC149373	Downregulated
A_24_P143	0.01223	0.00415	-1.56	FAM109A	family with sequence similarity 109, member A	Downregulated
A_22_P000	0.023558	0.00953	-1.56	lnc-MPHOSPI	lnc-MPHOSPH8-3:1	Downregulated
A_23_P200	0.029816	0.0128	-1.56	NTPCR	nucleoside-triphosphatase, cancer-related	Downregulated
A_23_P442	0.03225	0.0141	-1.56	FLII	flightless I homolog (Drosophila)	Downregulated
A_24_P749	0.000987	0.0000765	-1.57	PLP2	proteolipid protein 2 (colonic epithelium-enriched)	Downregulated
A_23_P553	0.001078	0.0000947	-1.57	KANSL1	KAT8 regulatory NSL complex subunit 1	Downregulated
A_23_P388	0.001088	0.0000968	-1.57	RABAC1	Rab acceptor 1 (prenylated)	Downregulated
A_24_P392	0.001092	0.0000975	-1.57	TRPM3	transient receptor potential cation channel, subfamily M, member 3	Downregulated
A_21_P001	0.001097	0.0000983	-1.57	STAU2	staufen double-stranded RNA binding protein 2	Downregulated
A_33_P330	0.001107	0.000101	-1.57	GOLGA2	golgin A2	Downregulated
A_23_P151	0.001154	0.00011	-1.57	MYCBP2	MYC binding protein 2, E3 ubiquitin protein ligase	Downregulated
A_22_P000	0.00126	0.000131	-1.57	lnc-WDR67-1	lnc-WDR67-1:1	Downregulated
A_33_P323	0.001275	0.000134	-1.57	UBL4A	ubiquitin-like 4A	Downregulated
A_33_P340	0.001404	0.00016	-1.57	STPG1	sperm-tail PG-rich repeat containing 1	Downregulated
A_24_P769	0.001597	0.000201	-1.57	C12orf73	chromosome 12 open reading frame 73	Downregulated
A_24_P324	0.001694	0.000224	-1.57	KCTD2	potassium channel tetramerization domain containing 2	Downregulated
A_24_P454	0.001821	0.000255	-1.57	SLAMF6	SLAM family member 6	Downregulated
A_23_P143	0.00189	0.000271	-1.57	PISD	phosphatidylserine decarboxylase	Downregulated
A_23_P802	0.00239	0.000401	-1.57	SFI1	Sfi1 homolog, spindle assembly associated (yeast)	Downregulated
A_23_P522	0.003012	0.000572	-1.57	ERLIN1	ER lipid raft associated 1	Downregulated
A_24_P753	0.004163	0.000927	-1.57	GAS2L1P2	growth arrest-specific 2 like 1 pseudogene 2	Downregulated
A_24_P978	0.004862	0.00117	-1.57	CCDC69	coiled-coil domain containing 69	Downregulated
A_23_P312	0.00758	0.0022	-1.57	GAL3ST4	galactose-3-O-sulfotransferase 4	Downregulated
A_33_P332	0.009447	0.00296	-1.57	ADCY2	adenylate cyclase 2 (brain)	Downregulated

A_23_P206	0.014296	0.00508	-1.57	NQO1	NAD(P)H dehydrogenase, quinone 1	Downregulated
A_22_P000	0.019849	0.0077	-1.57	lnc-LBR-1	lnc-LBR-1:3	Downregulated
A_22_P000	0.029816	0.0128	-1.57	EIF1AX-AS1	EIF1AX antisense RNA 1	Downregulated
A_22_P000	0.029816	0.0128	-1.57	LINC01265	long intergenic non-protein coding RNA 1265	Downregulated
A_24_P484	0.000991	0.0000773	-1.58	YES1	YES proto-oncogene 1, Src family tyrosine kinase	Downregulated
A_33_P340	0.001049	0.0000885	-1.58	MROH1	maestro heat-like repeat family member 1	Downregulated
A_23_P151	0.001066	0.0000922	-1.58	ARHGDIB	Rho GDP dissociation inhibitor (GDI) beta	Downregulated
A_22_P000	0.001181	0.000115	-1.58	LOC1027247	uncharacterized LOC102724715	Downregulated
A_21_P000	0.00121	0.000121	-1.58	LOC1019295	uncharacterized LOC101929531	Downregulated
A_23_P103	0.001448	0.000169	-1.58	FGR	FGR proto-oncogene, Src family tyrosine kinase	Downregulated
A_33_P326	0.001464	0.000173	-1.58	ANKRD52	ankyrin repeat domain 52	Downregulated
A_23_P123	0.001481	0.000177	-1.58	IDNK	idnK, gluconokinase homolog (E. coli)	Downregulated
A_24_P981	0.001977	0.000294	-1.58	SNX10	sorting nexin 10	Downregulated
A_33_P330	0.002341	0.000388	-1.58	TMEM110	transmembrane protein 110	Downregulated
A_24_P605	0.002517	0.000434	-1.58	PGGT1B	protein geranylgeranyltransferase type I, beta subunit	Downregulated
A_22_P000	0.002989	0.000564	-1.58	lnc-CCT4-1	lnc-CCT4-1:1	Downregulated
A_32_P216	0.003094	0.000596	-1.58	LDLRAP1	low density lipoprotein receptor adaptor protein 1	Downregulated
A_23_P103	0.003875	0.000835	-1.58	FCRL3	Fc receptor-like 3	Downregulated
A_23_P115	0.005162	0.00127	-1.58	ZZZ3	zinc finger, ZZ-type containing 3	Downregulated
A_33_P322	0.005837	0.00152	-1.58	TRAPPC13	trafficking protein particle complex 13	Downregulated
A_22_P000	0.035323	0.0158	-1.58	LOC1019294	uncharacterized LOC101929425	Downregulated
A_32_P189	0.036007	0.0161	-1.58	USHBP1	Usher syndrome 1C binding protein 1	Downregulated
A_33_P324	0.042851	0.0199	-1.58	SLMAP	sarcolemma associated protein	Downregulated
A_33_P332	0.001134	0.000106	-1.59	TBC1D10C	TBC1 domain family, member 10C	Downregulated
A_24_P363	0.001237	0.000126	-1.59	C5orf45	chromosome 5 open reading frame 45	Downregulated
A_21_P000	0.001596	0.0002	-1.59	lnc-DHRS2-3	lnc-DHRS2-3:1	Downregulated
A_33_P339	0.001606	0.000203	-1.59	USP39	ubiquitin specific peptidase 39	Downregulated
A_24_P356	0.002027	0.000308	-1.59	HEXIM1	hexamethylene bis-acetamide inducible 1	Downregulated
A_22_P000	0.002041	0.000312	-1.59	lnc-C17orf66-	lnc-C17orf66-1:1	Downregulated
A_33_P321	0.002544	0.000441	-1.59	ENTPD1	ectonucleoside triphosphate diphosphohydrolase 1	Downregulated
A_24_P557	0.003009	0.000571	-1.59	XAF1	XIAP associated factor 1	Downregulated
A_24_P954	0.005795	0.00151	-1.59	CARS	cysteinyl-tRNA synthetase	Downregulated
A_22_P000	0.008069	0.00239	-1.59	LOC1019276	uncharacterized LOC101927620	Downregulated
A_21_P000	0.013155	0.00455	-1.59	lnc-BOD1-2	lnc-BOD1-2:9	Downregulated
A_22_P000	0.016097	0.00593	-1.59	lnc-LRRC40-1	lnc-LRRC40-1:1	Downregulated
A_23_P395	0.028344	0.012	-1.59	ZFP42	ZFP42 zinc finger protein	Downregulated
A_23_P939	0.035934	0.0161	-1.59	ARHGEF5	Rho guanine nucleotide exchange factor (GEF) 5	Downregulated

A_24_P203	0.038648	0.0176	-1.59	SLC1A7	solute carrier family 1 (glutamate transporter), member 7	Downregulated
A_22_P000	0.000979	0.0000755	-1.6	TUG1	taurine up-regulated 1 (non-protein coding)	Downregulated
A_23_P478	0.001054	0.0000894	-1.6	TM7SF3	transmembrane 7 superfamily member 3	Downregulated
A_22_P000	0.001067	0.0000927	-1.6	SASH3	SAM and SH3 domain containing 3	Downregulated
A_33_P328	0.001179	0.000115	-1.6	S1PR4	sphingosine-1-phosphate receptor 4	Downregulated
A_21_P001	0.001193	0.000117	-1.6	FCRL5	Fc receptor-like 5	Downregulated
A_33_P340	0.001196	0.000118	-1.6	FCGR2C	Fc fragment of IgG, low affinity IIc, receptor for (CD32) (gene/pseudoger	Downregulated
A_33_P347	0.001479	0.000176	-1.6	PPP1R11	protein phosphatase 1, regulatory (inhibitor) subunit 11	Downregulated
A_23_P138	0.001821	0.000254	-1.6	FAIM3	Fas apoptotic inhibitory molecule 3	Downregulated
A_32_P162	0.002102	0.000328	-1.6	TAB3	TGF-beta activated kinase 1/MAP3K7 binding protein 3	Downregulated
A_23_P212	0.002291	0.000377	-1.6	SHISA5	shisa family member 5	Downregulated
A_23_P144	0.002469	0.00042	-1.6	GNPDA2	glucosamine-6-phosphate deaminase 2	Downregulated
A_22_P000	0.002944	0.00055	-1.6	lnc-TSPYL4-1	lnc-TSPYL4-1:1	Downregulated
A_33_P329	0.004951	0.00119	-1.6	FCRLB	Fc receptor-like B	Downregulated
A_24_P414	0.005502	0.0014	-1.6	PPP3CA	protein phosphatase 3, catalytic subunit, alpha isozyme	Downregulated
A_23_P105	0.006921	0.00194	-1.6	METRNL	meteorin, glial cell differentiation regulator-like	Downregulated
A_24_P179	0.008581	0.0026	-1.6	WDR66	WD repeat domain 66	Downregulated
A_33_P327	0.010612	0.00345	-1.6	ELOVL5	ELOVL fatty acid elongase 5	Downregulated
A_21_P001	0.020467	0.008	-1.6	LOC1005074	uncharacterized LOC100507487	Downregulated
A_23_P252	0.038752	0.0177	-1.6	COMMD10	COMM domain containing 10	Downregulated
A_23_P357	0.00096	0.0000717	-1.61	MBNL1	muscleblind-like splicing regulator 1	Downregulated
A_23_P423	0.001005	0.0000807	-1.61	HLA-DMA	major histocompatibility complex, class II, DM alpha	Downregulated
A_33_P329	0.001065	0.0000918	-1.61	IL2RG	interleukin 2 receptor, gamma	Downregulated
A_23_P210	0.001134	0.000106	-1.61	PCIF1	PDX1 C-terminal inhibiting factor 1	Downregulated
A_33_P680	0.001135	0.000107	-1.61	LOC1005068	uncharacterized LOC100506844	Downregulated
A_23_P377	0.001175	0.000114	-1.61	TNRC18	trinucleotide repeat containing 18	Downregulated
A_23_P373	0.001267	0.000133	-1.61	FUZ	fuzzy planar cell polarity protein	Downregulated
A_22_P000	0.001298	0.000138	-1.61	lnc-CT47A1-1	lnc-CT47A1-1:1	Downregulated
A_22_P000	0.001542	0.000189	-1.61	LINC00506	long intergenic non-protein coding RNA 506	Downregulated
A_24_P371	0.001633	0.00021	-1.61	C3orf58	chromosome 3 open reading frame 58	Downregulated
A_23_P105	0.001647	0.000213	-1.61	CAT	catalase	Downregulated
A_23_P861	0.001947	0.000285	-1.61	RPA2	replication protein A2, 32kDa	Downregulated
A_22_P000	0.002683	0.000478	-1.61	LOC255654	uncharacterized LOC255654	Downregulated
A_32_P236	0.003439	0.0007	-1.61	ABCB10	ATP-binding cassette, sub-family B (MDR/TAP), member 10	Downregulated
A_33_P332	0.006203	0.00166	-1.61	BLOC1S3	biogenesis of lysosomal organelles complex-1, subunit 3	Downregulated
A_24_P945	0.006753	0.00187	-1.61	RABEP1	rabaptin, RAB GTPase binding effector protein 1	Downregulated
A_23_P500	0.007149	0.00203	-1.61	SYNE1	spectrin repeat containing, nuclear envelope 1	Downregulated

A_23_P375	0.015845	0.00581	-1.61	FUNDC1	FUN14 domain containing 1	Downregulated
A_23_P288	0.020806	0.00816	-1.61	PIGH	phosphatidylinositol glycan anchor biosynthesis, class H	Downregulated
A_23_P365	0.030776	0.0133	-1.61	UBE3D	ubiquitin protein ligase E3D	Downregulated
A_23_P204	0.00097	0.0000738	-1.62	RB1	retinoblastoma 1	Downregulated
A_23_P411	0.000975	0.0000749	-1.62	PYGO2	pygopus family PHD finger 2	Downregulated
A_23_P111	0.001055	0.0000896	-1.62	AGAP3	ArfGAP with GTPase domain, ankyrin repeat and PH domain 3	Downregulated
A_33_P322	0.001078	0.0000947	-1.62	OFD1	oral-facial-digital syndrome 1	Downregulated
A_24_P160	0.001086	0.0000962	-1.62	PTRHD1	peptidyl-tRNA hydrolase domain containing 1	Downregulated
A_23_P148	0.0011	0.0000993	-1.62	RLIM	ring finger protein, LIM domain interacting	Downregulated
A_33_P335	0.001199	0.000119	-1.62	AGAP2	ArfGAP with GTPase domain, ankyrin repeat and PH domain 2	Downregulated
A_23_P127	0.001372	0.000152	-1.62	FAM35A	family with sequence similarity 35, member A	Downregulated
A_33_P339	0.001552	0.000191	-1.62	RHOB	ras homolog family member B	Downregulated
A_23_P392	0.001595	0.0002	-1.62	ZFP36	ZFP36 ring finger protein	Downregulated
A_23_P423	0.001709	0.000228	-1.62	FANCE	Fanconi anemia, complementation group E	Downregulated
A_23_P478	0.001753	0.000237	-1.62	DIABLO	diablo, IAP-binding mitochondrial protein	Downregulated
A_32_P768	0.001754	0.000238	-1.62	GOLGA8R	golgin A8 family, member R	Downregulated
A_32_P407	0.001916	0.000278	-1.62	LOC1019270	uncharacterized LOC101927085	Downregulated
A_33_P326	0.002253	0.000369	-1.62	ACTR3C	ARP3 actin-related protein 3 homolog C (yeast)	Downregulated
A_22_P000	0.003535	0.000729	-1.62	lnc-WDFY4-1	lnc-WDFY4-1:1	Downregulated
A_22_P000	0.003795	0.000808	-1.62	lnc-FSCB-4	lnc-FSCB-4:1	Downregulated
A_23_P200	0.007984	0.00236	-1.62	TGFBR3	transforming growth factor, beta receptor III	Downregulated
A_32_P479	0.014341	0.0051	-1.62	CAMK2D	calcium/calmodulin-dependent protein kinase II delta	Downregulated
A_23_P215	0.018633	0.00712	-1.62	KLHL7	kelch-like family member 7	Downregulated
A_21_P000	0.042431	0.0197	-1.62	lnc-NFRKB-1	lnc-NFRKB-1:1	Downregulated
A_33_P335	0.000946	0.0000693	-1.63	GPD2	glycerol-3-phosphate dehydrogenase 2 (mitochondrial)	Downregulated
A_23_P100	0.000964	0.0000731	-1.63	SKAP1	src kinase associated phosphoprotein 1	Downregulated
A_33_P326	0.001066	0.0000924	-1.63	RNVU1-18	RNA, variant U1 small nuclear 18	Downregulated
A_23_P122	0.001134	0.000106	-1.63	NELFE	negative elongation factor complex member E	Downregulated
A_23_P857	0.001142	0.000108	-1.63	FCGR2A	Fc fragment of IgG, low affinity IIa, receptor (CD32)	Downregulated
A_23_P326	0.001224	0.000124	-1.63	RBBP4	retinoblastoma binding protein 4	Downregulated
A_23_P226	0.001295	0.000138	-1.63	CYSLTR1	cysteinyl leukotriene receptor 1	Downregulated
A_23_P699	0.001826	0.000256	-1.63	SOX30	SRY (sex determining region Y)-box 30	Downregulated
A_19_P003	0.001826	0.000256	-1.63	TUG1	taurine up-regulated 1 (non-protein coding)	Downregulated
A_23_P366	0.002124	0.000335	-1.63	AQP5	aquaporin 5	Downregulated
A_24_P174	0.002414	0.000407	-1.63	PPP1R2	protein phosphatase 1, regulatory (inhibitor) subunit 2	Downregulated
A_21_P000	0.002709	0.000486	-1.63	RBM19	RNA binding motif protein 19	Downregulated
A_33_P331	0.003027	0.000576	-1.63	PARD6G-AS1	PARD6G antisense RNA 1	Downregulated

A_23_P371	0.006716	0.00186	-1.63	ALKBH8	alkB, alkylation repair homolog 8 (E. coli)	Downregulated
A_22_P000	0.01975	0.00765	-1.63	lnc-ACP1-1	lnc-ACP1-1:2	Downregulated
A_23_P225	0.02385	0.00969	-1.63	CHM	choroideremia (Rab escort protein 1)	Downregulated
A_24_P216	0.024364	0.00996	-1.63	SOAT1	sterol O-acyltransferase 1	Downregulated
A_24_P145	0.000938	0.0000671	-1.64	TRA2B	transformer 2 beta homolog (Drosophila)	Downregulated
A_24_P273	0.000954	0.0000702	-1.64	LINC00152	long intergenic non-protein coding RNA 152	Downregulated
A_23_P398	0.000954	0.0000704	-1.64	MIEN1	migration and invasion enhancer 1	Downregulated
A_24_P253	0.000973	0.0000746	-1.64	AP2B1	adaptor-related protein complex 2, beta 1 subunit	Downregulated
A_23_P132	0.000991	0.0000771	-1.64	UQCRC1	ubiquinol-cytochrome c reductase core protein I	Downregulated
A_33_P338	0.001069	0.0000934	-1.64	CAPG	capping protein (actin filament), gelsolin-like	Downregulated
A_23_P435	0.001142	0.000108	-1.64	ST8SIA4	ST8 alpha-N-acetyl-neuraminide alpha-2,8-sialyltransferase 4	Downregulated
A_23_P770	0.001203	0.00012	-1.64	SPPL2A	signal peptide peptidase like 2A	Downregulated
A_24_P196	0.001245	0.000128	-1.64	GTF2H5	general transcription factor IIH, polypeptide 5	Downregulated
A_23_P810	0.001443	0.000168	-1.64	STIM2	stromal interaction molecule 2	Downregulated
A_22_P000	0.001607	0.000203	-1.64	lnc-RP11-257	lnc-RP11-257K9.7.1-2:1	Downregulated
A_33_P327	0.001925	0.00028	-1.64	UBE2J1	ubiquitin-conjugating enzyme E2, J1	Downregulated
A_33_P333	0.00212	0.000333	-1.64	ZBED5-AS1	ZBED5 antisense RNA 1	Downregulated
A_21_P000	0.003716	0.000784	-1.64	TM4SF19	transmembrane 4 L six family member 19	Downregulated
A_23_P752	0.005695	0.00147	-1.64	NSMCE4A	non-SMC element 4 homolog A (S. cerevisiae)	Downregulated
A_22_P000	0.009842	0.00313	-1.64	ZEB2-AS1	ZEB2 antisense RNA 1	Downregulated
A_23_P357	0.014594	0.00522	-1.64	NAA40	N(alpha)-acetyltransferase 40, NatD catalytic subunit	Downregulated
A_33_P328	0.015428	0.00562	-1.64	LOC1001322	uncharacterized LOC100132249	Downregulated
A_23_P157	0.019778	0.00766	-1.64	POLR2K	polymerase (RNA) II (DNA directed) polypeptide K, 7.0kDa	Downregulated
A_33_P337	0.042497	0.0198	-1.64	IMPA1	inositol(myo)-1(or 4)-monophosphatase 1	Downregulated
A_22_P000	0.000919	0.0000645	-1.65	lnc-PAPSS2-2	lnc-PAPSS2-2:1	Downregulated
A_23_P420	0.000949	0.0000698	-1.65	PRKCB	protein kinase C, beta	Downregulated
A_23_P502	0.001187	0.000116	-1.65	ANGPTL6	angiopoietin-like 6	Downregulated
A_21_P001	0.00119	0.000117	-1.65	XLOC_l2_004771		Downregulated
A_23_P152	0.001314	0.000141	-1.65	ICAM2	intercellular adhesion molecule 2	Downregulated
A_23_P397	0.001612	0.000205	-1.65	SAMD3	sterile alpha motif domain containing 3	Downregulated
A_23_P215	0.001884	0.00027	-1.65	ST7	suppression of tumorigenicity 7	Downregulated
A_33_P339	0.001996	0.000299	-1.65	PI4KA	phosphatidylinositol 4-kinase, catalytic, alpha	Downregulated
A_33_P335	0.003126	0.000607	-1.65	NFXL1	nuclear transcription factor, X-box binding-like 1	Downregulated
A_33_P331	0.003445	0.000701	-1.65	ZZZ3	zinc finger, ZZ-type containing 3	Downregulated
A_33_P339	0.01153	0.00385	-1.65	CCDC134	coiled-coil domain containing 134	Downregulated
A_23_P256	0.015485	0.00564	-1.65	SLC10A1	solute carrier family 10 (sodium/bile acid cotransporter), member 1	Downregulated
A_24_P577	0.015919	0.00584	-1.65	MRPL52	mitochondrial ribosomal protein L52	Downregulated

A_22_P000	0.000995	0.0000787	-1.66	lnc-VAMP1-1	lnc-VAMP1-1:7	Downregulated
A_22_P000	0.00102	0.000083	-1.66	lnc-CTU2-2	lnc-CTU2-2:1	Downregulated
A_23_P154	0.001029	0.0000849	-1.66	SAP130	Sin3A-associated protein, 130kDa	Downregulated
A_23_P163	0.001121	0.000104	-1.66	PARP6	poly (ADP-ribose) polymerase family, member 6	Downregulated
A_23_P435	0.001156	0.00011	-1.66	NDUFA8	NADH dehydrogenase (ubiquinone) 1 alpha subcomplex, 8, 19kDa	Downregulated
A_21_P001	0.001353	0.000149	-1.66	XLOC_l2_013462		Downregulated
A_33_P328	0.00162	0.000206	-1.66	FUBP1	far upstream element (FUSE) binding protein 1	Downregulated
A_23_P169	0.00179	0.000246	-1.66	MRPS28	mitochondrial ribosomal protein S28	Downregulated
A_23_P407	0.001844	0.00026	-1.66	KIRREL3	kin of IRRE like 3 (Drosophila)	Downregulated
A_33_P334	0.00232	0.000383	-1.66	SUPT3H	suppressor of Ty 3 homolog (S. cerevisiae)	Downregulated
A_23_P621	0.002674	0.000475	-1.66	TIMP1	TIMP metallopeptidase inhibitor 1	Downregulated
A_23_P660	0.002685	0.000479	-1.66	B3GNT9	UDP-GlcNAc:betaGal beta-1,3-N-acetylglucosaminyltransferase 9	Downregulated
A_23_P353	0.002685	0.000479	-1.66	RMI2	RecQ mediated genome instability 2	Downregulated
A_33_P324	0.002701	0.000484	-1.66	SLC35E2B	solute carrier family 35, member E2B	Downregulated
A_32_P348	0.005993	0.00158	-1.66	WDR93	WD repeat domain 93	Downregulated
A_22_P000	0.007155	0.00203	-1.66	LOC1026064	uncharterized LOC102606465	Downregulated
A_33_P337	0.010925	0.00357	-1.66	ABCB9	ATP-binding cassette, sub-family B (MDR/TAP), member 9	Downregulated
A_22_P000	0.012258	0.00416	-1.66	MEG3	maternally expressed 3 (non-protein coding)	Downregulated
A_22_P000	0.014395	0.00513	-1.66	MRPL46	mitochondrial ribosomal protein L46	Downregulated
A_21_P000	0.01597	0.00587	-1.66	LOC1019275	uncharacterized LOC101927584	Downregulated
A_33_P339	0.029475	0.0126	-1.66	GYLTL1B	glycosyltransferase-like 1B	Downregulated
A_23_P311	0.000918	0.0000643	-1.67	GINM1	glycoprotein integral membrane 1	Downregulated
A_33_P325	0.000928	0.0000657	-1.67	ARHGAP40	Rho GTPase activating protein 40	Downregulated
A_33_P323	0.000945	0.0000688	-1.67	RLTPR	RGD motif, leucine rich repeats, tropomodulin domain and proline-rich co	Downregulated
A_23_P726	0.000963	0.0000727	-1.67	HGS	hepatocyte growth factor-regulated tyrosine kinase substrate	Downregulated
A_23_P394	0.001052	0.0000889	-1.67	BST2	bone marrow stromal cell antigen 2	Downregulated
A_23_P155	0.001142	0.000108	-1.67	CRELD1	cysteine-rich with EGF-like domains 1	Downregulated
A_24_P416	0.001181	0.000115	-1.67	RBL2	retinoblastoma-like 2	Downregulated
A_22_P000	0.001369	0.000152	-1.67	lnc-CTDSPL2	lnc-CTDSPL2-2:1	Downregulated
A_23_P162	0.001397	0.000158	-1.67	PTPN6	protein tyrosine phosphatase, non-receptor type 6	Downregulated
A_23_P161	0.001498	0.00018	-1.67	SNAPIN	SNAP-associated protein	Downregulated
A_19_P008	0.001653	0.000214	-1.67	LOC1005068	uncharacterized LOC100506804	Downregulated
A_23_P374	0.002912	0.000542	-1.67	SECISBP2L	SECIS binding protein 2-like	Downregulated
A_22_P000	0.003096	0.000597	-1.67	LOC1027253	uncharacterized LOC102725300	Downregulated
A_23_P135	0.004362	0.000996	-1.67	CKAP5	cytoskeleton associated protein 5	Downregulated
A_21_P000	0.005292	0.00132	-1.67	lnc-MDGA2-2	lnc-MDGA2-2:1	Downregulated
A_23_P145	0.006123	0.00163	-1.67	MTO1	mitochondrial tRNA translation optimization 1	Downregulated

A_24_P937	0.006315	0.0017	-1.67	EHMT1-IT1	EHMT1 intronic transcript 1	Downregulated
A_33_P339	0.007941	0.00234	-1.67	ATG4C	autophagy related 4C, cysteine peptidase	Downregulated
A_19_P003	0.009601	0.00302	-1.67	MCF2L	MCF.2 cell line derived transforming sequence-like	Downregulated
A_22_P000	0.014634	0.00525	-1.67	lnc-MPHOSPI	lnc-MPHOSPH8-3:1	Downregulated
A_24_P236	0.021828	0.00866	-1.67	CD2BP2	CD2 (cytoplasmic tail) binding protein 2	Downregulated
A_23_P729	0.02308	0.00928	-1.67	PRPS1	phosphoribosyl pyrophosphate synthetase 1	Downregulated
A_24_P270	0.023092	0.00928	-1.67	SCN4A	sodium channel, voltage gated, type IV alpha subunit	Downregulated
A_24_P416	0.000904	0.0000619	-1.68	GGA2	golgi-associated, gamma adaptin ear containing, ARF binding protein 2	Downregulated
A_33_P326	0.000938	0.0000677	-1.68	NEDD1	neural precursor cell expressed, developmentally down-regulated 1	Downregulated
A_24_P282	0.000959	0.0000711	-1.68	TLK1	tousled-like kinase 1	Downregulated
A_33_P327	0.001043	0.0000871	-1.68	FKBP2	FK506 binding protein 2, 13kDa	Downregulated
A_23_P798	0.001118	0.000103	-1.68	OSER1	oxidative stress responsive serine-rich 1	Downregulated
A_32_P386	0.001135	0.000106	-1.68	KRBA1	KRAB-A domain containing 1	Downregulated
A_23_P531	0.001296	0.000138	-1.68	LMO2	LIM domain only 2 (rhombotin-like 1)	Downregulated
A_33_P321	0.001694	0.000224	-1.68	KDM5C	lysine (K)-specific demethylase 5C	Downregulated
A_23_P107	0.001753	0.000237	-1.68	RAB27B	RAB27B, member RAS oncogene family	Downregulated
A_21_P000	0.002118	0.000333	-1.68	lnc-GPR65-1	lnc-GPR65-1:1	Downregulated
A_22_P000	0.002221	0.000359	-1.68	lnc-RIIAD1-1	lnc-RIIAD1-1:1	Downregulated
A_23_P307	0.002408	0.000405	-1.68	LSM12	LSM12 homolog (S. cerevisiae)	Downregulated
A_24_P212	0.003714	0.000784	-1.68	ZNF33A	zinc finger protein 33A	Downregulated
A_21_P000	0.005951	0.00156	-1.68	SNORD42A	small nucleolar RNA, C/D box 42A	Downregulated
A_23_P327	0.007949	0.00234	-1.68	GATS	GATS, stromal antigen 3 opposite strand	Downregulated
A_33_P324	0.008307	0.00249	-1.68	XLOC_l2_000001		Downregulated
A_23_P943	0.013786	0.00484	-1.68	ADCK5	aarF domain containing kinase 5	Downregulated
A_24_P182	0.016408	0.00609	-1.68	GCSAM	germinal center-associated, signaling and motility	Downregulated
A_19_P008	0.018008	0.00683	-1.68	XLOC_l2_008221		Downregulated
A_33_P340	0.019244	0.00742	-1.68	EXOG	endo/exonuclease (5'-3'), endonuclease G-like	Downregulated
A_33_P331	0.019709	0.00763	-1.68	LOC1005061	uncharacterized LOC100506191	Downregulated
A_24_P937	0.029816	0.0128	-1.68	TMEM198B	transmembrane protein 198B, pseudogene	Downregulated
A_22_P000	0.000908	0.0000626	-1.69	lnc-EMILIN2-1	lnc-EMILIN2-1:2	Downregulated
A_23_P419	0.000966	0.0000734	-1.69	BLCAP	bladder cancer associated protein	Downregulated
A_32_P309	0.001054	0.0000894	-1.69	WDFY4	WDFY family member 4	Downregulated
A_21_P000	0.001057	0.0000901	-1.69	lnc-CHST12-1	lnc-CHST12-1:6	Downregulated
A_23_P303	0.001443	0.000167	-1.69	STX7	syntaxin 7	Downregulated
A_24_P581	0.001557	0.000192	-1.69	C14orf159	chromosome 14 open reading frame 159	Downregulated
A_22_P000	0.002889	0.000536	-1.69	lnc-SIVA1-1	lnc-SIVA1-1:1	Downregulated
A_23_P559	0.00324	0.000641	-1.69	SLC1A5	solute carrier family 1 (neutral amino acid transporter), member 5	Downregulated

A_22_P000	0.003633	0.000759	-1.69	lnc-COL1A1-2	lnc-COL1A1-2:1	Downregulated
A_33_P322	0.00701	0.00197	-1.69	FBXO43	F-box protein 43	Downregulated
A_24_P116	0.008498	0.00257	-1.69	WWOX	WW domain containing oxidoreductase	Downregulated
A_33_P334	0.009072	0.00281	-1.69	ARGFXP2	arginine-fifty homeobox pseudogene 2	Downregulated
A_23_P307	0.009186	0.00285	-1.69	ABT1	activator of basal transcription 1	Downregulated
A_32_P940	0.023473	0.00948	-1.69	FAM92A1P2	family with sequence similarity 92, member A3	Downregulated
A_24_P237	0.025844	0.0107	-1.69	EIF1AX	eukaryotic translation initiation factor 1A, X-linked	Downregulated
A_21_P001	0.03725	0.0168	-1.69	HHIP	hedgehog interacting protein	Downregulated
A_33_P346	0.000913	0.0000639	-1.7	GTPBP10	GTP-binding protein 10 (putative)	Downregulated
A_33_P339	0.000925	0.0000652	-1.7	PTGR1	prostaglandin reductase 1	Downregulated
A_22_P000	0.000938	0.0000675	-1.7	DUS2	dihydrouridine synthase 2	Downregulated
A_24_P320	0.000998	0.0000798	-1.7	C6orf106	chromosome 6 open reading frame 106	Downregulated
A_24_P323	0.001023	0.0000839	-1.7	GNRH2	gonadotropin-releasing hormone 2	Downregulated
A_22_P000	0.001095	0.0000978	-1.7	lnc-ITM2C-2	lnc-ITM2C-2:1	Downregulated
A_33_P325	0.001453	0.000171	-1.7	RMND5B	required for meiotic nuclear division 5 homolog B (S. cerevisiae)	Downregulated
A_23_P341	0.001536	0.000188	-1.7	MNT	MAX network transcriptional repressor	Downregulated
A_33_P335	0.001691	0.000223	-1.7	LOC388692	uncharacterized LOC388692	Downregulated
A_23_P119	0.002488	0.000426	-1.7	TIMM44	translocase of inner mitochondrial membrane 44 homolog (yeast)	Downregulated
A_23_P208	0.003521	0.000724	-1.7	EMC10	ER membrane protein complex subunit 10	Downregulated
A_23_P315	0.005093	0.00124	-1.7	ABHD11	abhydrolase domain containing 11	Downregulated
A_23_P125	0.007111	0.00202	-1.7	TMEM255A	transmembrane protein 255A	Downregulated
A_23_P996	0.007969	0.00235	-1.7	RNF31	ring finger protein 31	Downregulated
A_24_P322	0.010101	0.00324	-1.7	ELMO2	engulfment and cell motility 2	Downregulated
A_33_P324	0.011206	0.0037	-1.7	MAVS	mitochondrial antiviral signaling protein	Downregulated
A_33_P325	0.012979	0.00448	-1.7	OGFR	opioid growth factor receptor	Downregulated
A_33_P329	0.027436	0.0116	-1.7	DENND4C	DENN/MADD domain containing 4C	Downregulated
A_32_P206	0.028104	0.0119	-1.7	DNAH2	dynein, axonemal, heavy chain 2	Downregulated
A_22_P000	0.000899	0.0000611	-1.71	lnc-PLEKHA3	lnc-PLEKHA3-3:1	Downregulated
A_23_P537	0.000924	0.0000649	-1.71	FBXO21	F-box protein 21	Downregulated
A_33_P330	0.000949	0.0000698	-1.71	C21orf58	chromosome 21 open reading frame 58	Downregulated
A_23_P361	0.000954	0.0000705	-1.71	SLC35F5	solute carrier family 35, member F5	Downregulated
A_33_P324	0.000971	0.0000743	-1.71	HBS1L	HBS1-like translational GTPase	Downregulated
A_22_P000	0.001022	0.0000836	-1.71	LINC01429	long intergenic non-protein coding RNA 1429	Downregulated
A_33_P330	0.001062	0.0000907	-1.71	RANBP2	RAN binding protein 2	Downregulated
A_33_P365	0.001086	0.000096	-1.71	CXorf49B	chromosome X open reading frame 49B	Downregulated
A_33_P324	0.001991	0.000298	-1.71	ATAD3C	ATPase family, AAA domain containing 3C	Downregulated
A_21_P000	0.002488	0.000426	-1.71	LOC1002893	uncharacterized LOC100289361	Downregulated

A_22_P000	0.006688	0.00185	-1.71	lnc-UPK3B-1	lnc-UPK3B-1:3	Downregulated
A_33_P324	0.026919	0.0113	-1.71	RGS5	regulator of G-protein signaling 5	Downregulated
A_23_P483	0.041342	0.0191	-1.71	PCCA	propionyl CoA carboxylase, alpha polypeptide	Downregulated
A_23_P212	0.001015	0.0000824	-1.72	NAT6	N-acetyltransferase 6 (GCN5-related)	Downregulated
A_23_P209	0.001029	0.0000848	-1.72	TRAK2	trafficking protein, kinesin binding 2	Downregulated
A_22_P000	0.0011	0.0000992	-1.72	lnc-ADAMTS1	lnc-ADAMTS18-1:1	Downregulated
A_23_P578	0.001187	0.000116	-1.72	ACY1	aminoacylase 1	Downregulated
A_23_P798	0.001386	0.000155	-1.72	SERINC3	serine incorporator 3	Downregulated
A_22_P000	0.001444	0.000168	-1.72	lnc-SLC22A1	lnc-SLC22A18-1:1	Downregulated
A_22_P000	0.00151	0.000182	-1.72	lnc-LIFR-1	lnc-LIFR-1:1	Downregulated
A_33_P331	0.001604	0.000202	-1.72	CCDC14	coiled-coil domain containing 14	Downregulated
A_33_P331	0.001738	0.000234	-1.72	PHIP	pleckstrin homology domain interacting protein	Downregulated
A_23_P196	0.001742	0.000235	-1.72	RARRES3	retinoic acid receptor responder (tazarotene induced) 3	Downregulated
A_21_P000	0.001744	0.000235	-1.72	SNORA80E	small nucleolar RNA, H/ACA box 80E	Downregulated
A_21_P000	0.001808	0.000251	-1.72	METTL23	methyltransferase like 23	Downregulated
A_24_P134	0.001893	0.000272	-1.72	BTBD3	BTB (POZ) domain containing 3	Downregulated
A_23_P209	0.001962	0.000291	-1.72	CLIP4	CAP-GLY domain containing linker protein family, member 4	Downregulated
A_23_P520	0.002022	0.000306	-1.72	GRHL3	grainyhead-like 3 (Drosophila)	Downregulated
A_23_P498	0.002621	0.000462	-1.72	ADAP2	ArfGAP with dual PH domains 2	Downregulated
A_23_P396	0.003847	0.000827	-1.72	ORC2	origin recognition complex, subunit 2	Downregulated
A_23_P252	0.003875	0.000835	-1.72	RIPK2	receptor-interacting serine-threonine kinase 2	Downregulated
A_33_P321	0.004961	0.0012	-1.72	ENTPD1	ectonucleoside triphosphate diphosphohydrolase 1	Downregulated
A_21_P000	0.006305	0.0017	-1.72	lnc-ZNF37A-3	lnc-ZNF37A-3:2	Downregulated
A_21_P001	0.00635	0.00172	-1.72	XLOC_l2_007586		Downregulated
A_23_P741	0.006955	0.00195	-1.72	RAD54L	RAD54-like (S. cerevisiae)	Downregulated
A_23_P432	0.030987	0.0134	-1.72	CHMP4C	charged multivesicular body protein 4C	Downregulated
A_33_P330	0.000877	0.0000583	-1.73	PARP4	poly (ADP-ribose) polymerase family, member 4	Downregulated
A_23_P131	0.000909	0.000063	-1.73	HDLBP	high density lipoprotein binding protein	Downregulated
A_24_P412	0.000915	0.0000641	-1.73	TMEM143	transmembrane protein 143	Downregulated
A_24_P216	0.001022	0.0000836	-1.73	ERGIC3	ERGIC and golgi 3	Downregulated
A_23_P118	0.001036	0.000086	-1.73	SRP68	signal recognition particle 68kDa	Downregulated
A_33_P338	0.00124	0.000126	-1.73	MDM4	MDM4, p53 regulator	Downregulated
A_23_P622	0.001279	0.000135	-1.73	NKAP	NFKB activating protein	Downregulated
A_23_P142	0.001492	0.000179	-1.73	GIGYF2	GRB10 interacting GYF protein 2	Downregulated
A_33_P334	0.002027	0.000308	-1.73	SLC25A37	solute carrier family 25 (mitochondrial iron transporter), member 37	Downregulated
A_33_P322	0.002054	0.000315	-1.73	KNSTRN	kinetochore-localized astrin/SPAG5 binding protein	Downregulated
A_33_P334	0.002219	0.000358	-1.73	ZNF280D	zinc finger protein 280D	Downregulated

A_33_P336	0.002358	0.000393	-1.73	TRAPPC2	trafficking protein particle complex 2	Downregulated
A_23_P203	0.002596	0.000455	-1.73	GAB2	GRB2-associated binding protein 2	Downregulated
A_23_P140	0.003567	0.000739	-1.73	NDRG4	NDRG family member 4	Downregulated
A_33_P335	0.004971	0.0012	-1.73	TAF4B	TAF4b RNA polymerase II, TATA box binding protein (TBP)-associated f	Downregulated
A_21_P000	0.007266	0.00207	-1.73	LOC1027239	uncharacterized LOC102723932	Downregulated
A_21_P000	0.01353	0.00472	-1.73	NIFK-AS1	NIFK antisense RNA 1	Downregulated
A_21_P000	0.022388	0.00893	-1.73	SNORD35A	small nucleolar RNA, C/D box 35A	Downregulated
A_33_P324	0.025262	0.0104	-1.73	IL1RN	interleukin 1 receptor antagonist	Downregulated
A_33_P326	0.030274	0.0131	-1.73	LOC1002891	uncharacterized LOC100289120	Downregulated
A_23_P147	0.000866	0.0000569	-1.74	RPS27L	ribosomal protein S27-like	Downregulated
A_23_P137	0.000931	0.0000663	-1.74	KDM5D	lysine (K)-specific demethylase 5D	Downregulated
A_22_P000	0.000938	0.0000676	-1.74	lnc-C14orf57-	lnc-C14orf57-1:1	Downregulated
A_24_P277	0.000991	0.0000772	-1.74	FIS1	fission 1 (mitochondrial outer membrane) homolog (S. cerevisiae)	Downregulated
A_22_P000	0.00114	0.000108	-1.74	LINC00116	long intergenic non-protein coding RNA 116	Downregulated
A_22_P000	0.001393	0.000156	-1.74	lnc-MAPK6-8	lnc-MAPK6-8:1	Downregulated
A_33_P329	0.001763	0.00024	-1.74	FAM159A	family with sequence similarity 159, member A	Downregulated
A_23_P148	0.002243	0.000366	-1.74	TSPAN3	tetraspanin 3	Downregulated
A_24_P303	0.002301	0.000378	-1.74	ARL15	ADP-ribosylation factor-like 15	Downregulated
A_24_P234	0.002794	0.000508	-1.74	CSNK1G3	casein kinase 1, gamma 3	Downregulated
A_23_P837	0.005191	0.00128	-1.74	C11orf63	chromosome 11 open reading frame 63	Downregulated
A_33_P342	0.008303	0.00249	-1.74	SP2	Sp2 transcription factor	Downregulated
A_23_P163	0.000853	0.000055	-1.75	ACYP1	acylphosphatase 1, erythrocyte (common) type	Downregulated
A_23_P359	0.000903	0.0000616	-1.75	ALKBH3	alkB, alkylation repair homolog 3 (E. coli)	Downregulated
A_33_P331	0.000923	0.0000648	-1.75	IMMT	inner membrane protein, mitochondrial	Downregulated
A_33_P339	0.000991	0.0000772	-1.75	PROSER1	proline and serine rich 1	Downregulated
A_23_P152	0.001065	0.0000919	-1.75	CSK	c-src tyrosine kinase	Downregulated
A_24_P219	0.001546	0.00019	-1.75	RBM23	RNA binding motif protein 23	Downregulated
A_23_P231	0.002421	0.000408	-1.75	PINK1	PTEN induced putative kinase 1	Downregulated
A_33_P333	0.002503	0.000431	-1.75	XLOC_l2_015203		Downregulated
A_21_P000	0.002568	0.000448	-1.75	lnc-CSMD1-9	lnc-CSMD1-9:1	Downregulated
A_21_P000	0.002803	0.000511	-1.75	SNORD62A	small nucleolar RNA, C/D box 62A	Downregulated
A_33_P330	0.003637	0.00076	-1.75	DDX31	DEAD (Asp-Glu-Ala-Asp) box polypeptide 31	Downregulated
A_33_P326	0.003878	0.000836	-1.75	PITRM1	pitrilysin metallopeptidase 1	Downregulated
A_33_P335	0.004745	0.00113	-1.75	ATHL1	ATH1, acid trehalase-like 1 (yeast)	Downregulated
A_21_P000	0.004979	0.0012	-1.75	RAB30-AS1	RAB30 antisense RNA 1 (head to head)	Downregulated
A_23_P201	0.005327	0.00133	-1.75	PTPN7	protein tyrosine phosphatase, non-receptor type 7	Downregulated
A_23_P125	0.005417	0.00137	-1.75	SLC25A43	solute carrier family 25, member 43	Downregulated

A_19_P003	0.016688	0.00622	-1.75	AATBC	uncharacterized LOC284837	Downregulated
A_23_P122	0.00086	0.0000563	-1.76	LTV1	LTV1 ribosome biogenesis factor	Downregulated
A_24_P189	0.000952	0.0000701	-1.76	ENDOD1	endonuclease domain containing 1	Downregulated
A_24_P416	0.000959	0.0000711	-1.76	ADCY7	adenylate cyclase 7	Downregulated
A_24_P917	0.000995	0.0000788	-1.76	TMED10	transmembrane emp24-like trafficking protein 10 (yeast)	Downregulated
A_33_P332	0.000998	0.0000796	-1.76	NLK	nemo-like kinase	Downregulated
A_21_P001	0.001013	0.000082	-1.76	lnc-HSP90AA	lnc-HSP90AA1-5:1	Downregulated
A_33_P328	0.00123	0.000125	-1.76	FAM204A	family with sequence similarity 204, member A	Downregulated
A_23_P398	0.001763	0.00024	-1.76	C15orf57	chromosome 15 open reading frame 57	Downregulated
A_23_P205	0.001826	0.000256	-1.76	GNB5	guanine nucleotide binding protein (G protein), beta 5	Downregulated
A_23_P604	0.00183	0.000257	-1.76	ODF2	outer dense fiber of sperm tails 2	Downregulated
A_33_P336	0.002101	0.000327	-1.76	RRN3	RRN3 RNA polymerase I transcription factor homolog (S. cerevisiae)	Downregulated
A_21_P000	0.002832	0.000519	-1.76	ITGB2-AS1	ITGB2 antisense RNA 1	Downregulated
A_24_P399	0.004012	0.000877	-1.76	ZCCHC3	zinc finger, CCHC domain containing 3	Downregulated
A_33_P375	0.005623	0.00144	-1.76	M1AP	meiosis 1 associated protein	Downregulated
A_22_P000	0.009143	0.00284	-1.76	ZBED1	zinc finger, BED-type containing 1	Downregulated
A_23_P370	0.009526	0.00299	-1.76	SEPN1	selenoprotein N, 1	Downregulated
A_33_P361	0.009939	0.00317	-1.76	LOC283516	uncharacterized LOC283516	Downregulated
A_33_P330	0.010871	0.00355	-1.76	ZPBP	zona pellucida binding protein	Downregulated
A_33_P332	0.011625	0.00389	-1.76	DAZAP2	DAZ associated protein 2	Downregulated
A_22_P000	0.016015	0.00588	-1.76	LOC1019281	uncharacterized LOC101928156	Downregulated
A_21_P000	0.024466	0.01	-1.76	CMAHP	cytidine monophospho-N-acetylneuraminic acid hydroxylase, pseudoger	Downregulated
A_24_P844	0.000898	0.0000609	-1.77	VAV2	vav 2 guanine nucleotide exchange factor	Downregulated
A_22_P000	0.000938	0.0000674	-1.77	CCDC175	coiled-coil domain containing 175	Downregulated
A_24_P655	0.000945	0.0000688	-1.77	STRA13	stimulated by retinoic acid 13	Downregulated
A_23_P243	0.000995	0.0000789	-1.77	OTUB1	OTU deubiquitinase, ubiquitin aldehyde binding 1	Downregulated
A_23_P120	0.000999	0.0000799	-1.77	APOBEC3C	apolipoprotein B mRNA editing enzyme, catalytic polypeptide-like 3C	Downregulated
A_23_P341	0.001333	0.000145	-1.77	WWC3	WWC family member 3	Downregulated
A_23_P750	0.001638	0.000211	-1.77	ZMYND11	zinc finger, MYND-type containing 11	Downregulated
A_23_P771	0.001702	0.000227	-1.77	RAB11A	RAB11A, member RAS oncogene family	Downregulated
A_22_P000	0.001808	0.00025	-1.77	lnc-ARL16-1	lnc-ARL16-1:1	Downregulated
A_23_P334	0.002241	0.000365	-1.77	FAM167A	family with sequence similarity 167, member A	Downregulated
A_22_P000	0.002995	0.000567	-1.77	lnc-FANCI-1	lnc-FANCI-1:1	Downregulated
A_23_P115	0.003568	0.00074	-1.77	CELF2	CUGBP, Elav-like family member 2	Downregulated
A_33_P322	0.013974	0.00492	-1.77	TRMT13	tRNA methyltransferase 13 homolog (S. cerevisiae)	Downregulated
A_23_P141	0.023836	0.00968	-1.77	DHRS7B	dehydrogenase/reductase (SDR family) member 7B	Downregulated
A_23_P204	0.026881	0.0113	-1.77	ZNF384	zinc finger protein 384	Downregulated

A_33_P337	0.000987	0.0000764	-1.78	ABR	active BCR-related	Downregulated
A_23_P400	0.000998	0.0000798	-1.78	GTF3C6	general transcription factor IIIC, polypeptide 6, alpha 35kDa	Downregulated
A_23_P858	0.001007	0.0000812	-1.78	CD52	CD52 molecule	Downregulated
A_21_P001	0.001133	0.000106	-1.78	lnc-PPDPF-1	lnc-PPDPF-1:2	Downregulated
A_23_P697	0.001149	0.000109	-1.78	AP1AR	adaptor-related protein complex 1 associated regulatory protein	Downregulated
A_24_P829	0.001203	0.00012	-1.78	RNF114	ring finger protein 114	Downregulated
A_22_P000	0.00151	0.000182	-1.78	lnc-SPTBN5-2	lnc-SPTBN5-2:1	Downregulated
A_33_P320	0.001836	0.000258	-1.78	CTNND1	catenin (cadherin-associated protein), delta 1	Downregulated
A_32_P933	0.002067	0.000319	-1.78	NUDT19	nudix (nucleoside diphosphate linked moiety X)-type motif 19	Downregulated
A_23_P831	0.0024	0.000404	-1.78	PTPLAD2	protein tyrosine phosphatase-like A domain containing 2	Downregulated
A_24_P274	0.003316	0.000665	-1.78	GTF2H2C_2	GTF2H2 family member C, copy 2	Downregulated
A_33_P324	0.003599	0.00075	-1.78	DMKN	dermokine	Downregulated
A_24_P630	0.005643	0.00145	-1.78	SFXN5	sideroflexin 5	Downregulated
A_23_P409	0.010468	0.00339	-1.78	PPM1A	protein phosphatase, Mg2+/Mn2+ dependent, 1A	Downregulated
A_23_P402	0.011468	0.00382	-1.78	LNX2	ligand of numb-protein X 2	Downregulated
A_23_P200	0.01384	0.00486	-1.78	NOTCH2	notch 2	Downregulated
A_33_P338	0.015382	0.00559	-1.78	LOC1019271	uncharacterized LOC101927100	Downregulated
A_33_P323	0.023408	0.00945	-1.78	CLIP4	CAP-GLY domain containing linker protein family, member 4	Downregulated
A_23_P536	0.028042	0.0119	-1.78	PAWR	PRKC, apoptosis, WT1, regulator	Downregulated
A_23_P326	0.035407	0.0158	-1.78	CSMD3	CUB and Sushi multiple domains 3	Downregulated
A_22_P000	0.000807	0.0000487	-1.79	lnc-PLCD3-1	lnc-PLCD3-1:1	Downregulated
A_23_P688	0.000906	0.0000623	-1.79	TUG1	taurine up-regulated 1 (non-protein coding)	Downregulated
A_24_P253	0.000961	0.000072	-1.79	CIRH1A	cirrhosis, autosomal recessive 1A (cirhin)	Downregulated
A_23_P126	0.000995	0.0000779	-1.79	PGD	phosphogluconate dehydrogenase	Downregulated
A_21_P000	0.001022	0.0000836	-1.79	SNORD58C	small nucleolar RNA, C/D box 58C	Downregulated
A_32_P177	0.001731	0.000232	-1.79	SPDYE5	speedy/RINGO cell cycle regulator family member E5	Downregulated
A_33_P336	0.002022	0.000306	-1.79	SYT2	synaptotagmin II	Downregulated
A_22_P000	0.002309	0.00038	-1.79	lnc-CD53-1	lnc-CD53-1:1	Downregulated
A_33_P339	0.003003	0.000569	-1.79	C19orf84	chromosome 19 open reading frame 84	Downregulated
A_22_P000	0.003308	0.000661	-1.79	STRIP1	striatin interacting protein 1	Downregulated
A_23_P535	0.004344	0.00099	-1.79	MTERF2	mitochondrial transcription termination factor 2	Downregulated
A_32_P355	0.004528	0.00105	-1.79	VPS35	vacuolar protein sorting 35 homolog (S. cerevisiae)	Downregulated
A_22_P000	0.004677	0.0011	-1.79	lnc-EID1-3	lnc-EID1-3:1	Downregulated
A_23_P158	0.009825	0.00312	-1.79	C9orf16	chromosome 9 open reading frame 16	Downregulated
A_23_P141	0.019109	0.00735	-1.79	RASL11A	RAS-like, family 11, member A	Downregulated
A_23_P361	0.033723	0.0149	-1.79	ATXN3L	ataxin 3-like	Downregulated
A_21_P000	0.000904	0.0000619	-1.8	LOC1019277	uncharacterized LOC101927712	Downregulated

A_24_P389	0.001107	0.000101	-1.8	RELT	RELT tumor necrosis factor receptor	Downregulated
A_23_P123	0.001325	0.000143	-1.8	PRMT6	protein arginine methyltransferase 6	Downregulated
A_24_P336	0.001409	0.000161	-1.8	CABIN1	calcineurin binding protein 1	Downregulated
A_23_P215	0.001443	0.000167	-1.8	POM121	POM121 transmembrane nucleoporin	Downregulated
A_23_P385	0.001512	0.000183	-1.8	DNAJB6	DnaJ (Hsp40) homolog, subfamily B, member 6	Downregulated
A_22_P000	0.006544	0.00179	-1.8	lnc-SMG6-3	lnc-SMG6-3:1	Downregulated
A_33_P340	0.01812	0.00689	-1.8	CEP85L	centrosomal protein 85kDa-like	Downregulated
A_33_P354	0.02876	0.0122	-1.8	OR5AK4P	olfactory receptor, family 5, subfamily AK, member 4 pseudogene	Downregulated
A_23_P300	0.000805	0.0000483	-1.81	C6orf136	chromosome 6 open reading frame 136	Downregulated
A_23_P239	0.000814	0.0000497	-1.81	CAPN2	calpain 2, (m/II) large subunit	Downregulated
A_24_P186	0.000901	0.0000614	-1.81	PDS5A	PDS5 cohesin associated factor A	Downregulated
A_33_P339	0.00093	0.000066	-1.81	MBD1	methyl-CpG binding domain protein 1	Downregulated
A_21_P000	0.000939	0.0000679	-1.81	PARGP1	poly (ADP-ribose) glycohydrolase pseudogene 1	Downregulated
A_21_P000	0.001471	0.000174	-1.81	lnc-CDC7-1	lnc-CDC7-1:1	Downregulated
A_21_P001	0.001611	0.000204	-1.81	LINC00342	long intergenic non-protein coding RNA 342	Downregulated
A_22_P000	0.001919	0.000278	-1.81	lnc-PAK2-1	lnc-PAK2-1:1	Downregulated
A_33_P333	0.003816	0.000815	-1.81	ZNF746	zinc finger protein 746	Downregulated
A_24_P235	0.003932	0.000852	-1.81	THAP5	THAP domain containing 5	Downregulated
A_21_P000	0.006961	0.00195	-1.81	RNASEH1-AS	RNASEH1 antisense RNA 1	Downregulated
A_22_P000	0.007438	0.00214	-1.81	lnc-AL020996	lnc-AL020996.1-1:2	Downregulated
A_23_P381	0.013653	0.00478	-1.81	GGA3	golgi-associated, gamma adaptin ear containing, ARF binding protein 3	Downregulated
A_33_P330	0.000793	0.0000467	-1.82	RHOB	ras homolog family member B	Downregulated
A_33_P325	0.000814	0.0000498	-1.82	DPM3	dolichyl-phosphate mannosyltransferase polypeptide 3	Downregulated
A_32_P101	0.00082	0.0000507	-1.82	lnc-THNSL1-2	lnc-THNSL1-2:1	Downregulated
A_23_P734	0.000883	0.000059	-1.82	RUFY1	RUN and FYVE domain containing 1	Downregulated
A_22_P000	0.000922	0.0000647	-1.82	SLC25A30-AS	SLC25A30 antisense RNA 1	Downregulated
A_23_P393	0.001065	0.000092	-1.82	HCST	hematopoietic cell signal transducer	Downregulated
A_24_P358	0.001312	0.000141	-1.82	GTPBP6	GTP binding protein 6 (putative)	Downregulated
A_33_P326	0.001657	0.000215	-1.82	KRTAP5-6	keratin associated protein 5-6	Downregulated
A_24_P409	0.001763	0.00024	-1.82	BCL2L13	BCL2-like 13 (apoptosis facilitator)	Downregulated
A_24_P315	0.002575	0.000451	-1.82	POM121L8P	POM121 transmembrane nucleoporin-like 8 pseudogene	Downregulated
A_22_P000	0.004157	0.000924	-1.82	lnc-RP4-604K	lnc-RP4-604K5.1.1-1:1	Downregulated
A_24_P203	0.005562	0.00142	-1.82	ACAT1	acetyl-CoA acetyltransferase 1	Downregulated
A_24_P416	0.005764	0.00149	-1.82	HOXB4	homeobox B4	Downregulated
A_24_P321	0.007357	0.00211	-1.82	TTLL5	tubulin tyrosine ligase-like family member 5	Downregulated
A_24_P156	0.011666	0.00391	-1.82	MPL	MPL proto-oncogene, thrombopoietin receptor	Downregulated
A_22_P000	0.016458	0.00611	-1.82	lnc-NARF-1	lnc-NARF-1:1	Downregulated

A_21_P000	0.024008	0.00977	-1.82	lnc-CEP68-1	lnc-CEP68-1:2	Downregulated
A_21_P000	0.000795	0.0000469	-1.83	SLITRK5	SLIT and NTRK-like family, member 5	Downregulated
A_23_P240	0.000875	0.0000581	-1.83	IFIT2	interferon-induced protein with tetratricopeptide repeats 2	Downregulated
A_23_P116	0.001065	0.0000919	-1.83	HMGB4	high mobility group box 4	Downregulated
A_23_P141	0.001171	0.000113	-1.83	ZNF18	zinc finger protein 18	Downregulated
A_22_P000	0.001564	0.000194	-1.83	lnc-CDK12-2	lnc-CDK12-2:1	Downregulated
A_21_P000	0.001693	0.000223	-1.83	lnc-DCTD-1	lnc-DCTD-1:1	Downregulated
A_23_P250	0.001826	0.000256	-1.83	CLIP1	CAP-GLY domain containing linker protein 1	Downregulated
A_23_P167	0.002112	0.00033	-1.83	HIST1H2AC	histone cluster 1, H2ac	Downregulated
A_32_P197	0.00293	0.000546	-1.83	ZNF697	zinc finger protein 697	Downregulated
A_32_P230	0.003359	0.000677	-1.83	DOCK7	dedicator of cytokinesis 7	Downregulated
A_33_P329	0.007446	0.00214	-1.83	DISP1	dispatched homolog 1 (Drosophila)	Downregulated
A_24_P126	0.021799	0.00864	-1.83	TMEM194A	transmembrane protein 194A	Downregulated
A_33_P332	0.000792	0.0000463	-1.84	ANKLE2	ankyrin repeat and LEM domain containing 2	Downregulated
A_24_P117	0.000792	0.0000464	-1.84	LDLR	low density lipoprotein receptor	Downregulated
A_23_P161	0.000982	0.0000759	-1.84	ZDHHC6	zinc finger, DHHC-type containing 6	Downregulated
A_24_P135	0.001134	0.000106	-1.84	PTPLB	protein tyrosine phosphatase-like (proline instead of catalytic arginine), r	Downregulated
A_23_P350	0.001171	0.000113	-1.84	TRIM56	tripartite motif containing 56	Downregulated
A_24_P232	0.002012	0.000304	-1.84	SMARCD1	SWI/SNF related, matrix associated, actin dependent regulator of chrom	Downregulated
A_23_P160	0.003056	0.000584	-1.84	NR0B2	nuclear receptor subfamily 0, group B, member 2	Downregulated
A_23_P298	0.003119	0.000604	-1.84	CBLB	Cbl proto-oncogene B, E3 ubiquitin protein ligase	Downregulated
A_23_P302	0.003542	0.000731	-1.84	ERAP2	endoplasmic reticulum aminopeptidase 2	Downregulated
A_23_P153	0.006575	0.00181	-1.84	GTDC1	glycosyltransferase-like domain containing 1	Downregulated
A_23_P253	0.013477	0.0047	-1.84	GRAMD1C	GRAM domain containing 1C	Downregulated
A_23_P342	0.035237	0.0157	-1.84	FBXW4	F-box and WD repeat domain containing 4	Downregulated
A_24_P472	0.000808	0.0000489	-1.85	ARF6	ADP-ribosylation factor 6	Downregulated
A_33_P334	0.001085	0.0000959	-1.85	MKLN1	muskelin 1, intracellular mediator containing kelch motifs	Downregulated
A_23_P365	0.001153	0.00011	-1.85	EID2	EP300 interacting inhibitor of differentiation 2	Downregulated
A_22_P000	0.001157	0.00011	-1.85	LOC1019282	uncharacterized LOC101928273	Downregulated
A_23_P615	0.001399	0.000159	-1.85	CD2BP2	CD2 (cytoplasmic tail) binding protein 2	Downregulated
A_23_P646	0.001424	0.000163	-1.85	ARHGAP9	Rho GTPase activating protein 9	Downregulated
A_23_P314	0.001427	0.000164	-1.85	ERVW-1	endogenous retrovirus group W, member 1	Downregulated
A_23_P386	0.001537	0.000188	-1.85	TWISTNB	TWIST neighbor	Downregulated
A_23_P390	0.001558	0.000193	-1.85	WDR83	WD repeat domain 83	Downregulated
A_21_P000	0.002016	0.000305	-1.85	lnc-SLC2A9-1	lnc-SLC2A9-1:1	Downregulated
A_24_P248	0.003749	0.000795	-1.85	ACSL3	acyl-CoA synthetase long-chain family member 3	Downregulated
A_33_P336	0.006501	0.00178	-1.85	CDC27	cell division cycle 27	Downregulated

A_33_P324	0.007142	0.00203	-1.85	PKN2	protein kinase N2	Downregulated
A_22_P000	0.014655	0.00526	-1.85	lnc-PAPLN-1	lnc-PAPLN-1:1	Downregulated
A_23_P200	0.01581	0.00579	-1.85	HYI	hydroxypyruvate isomerase (putative)	Downregulated
A_23_P116	0.019336	0.00746	-1.85	SPRR2D	small proline-rich protein 2D	Downregulated
A_23_P833	0.000776	0.0000439	-1.86	ENG	endoglin	Downregulated
A_23_P258	0.000853	0.0000548	-1.86	MRPS17	mitochondrial ribosomal protein S17	Downregulated
A_23_P169	0.0009	0.0000612	-1.86	ANXA4	annexin A4	Downregulated
A_24_P329	0.000913	0.0000638	-1.86	TSC1	tuberous sclerosis 1	Downregulated
A_23_P102	0.000947	0.0000693	-1.86	CXCR4	chemokine (C-X-C motif) receptor 4	Downregulated
A_23_P378	0.001215	0.000122	-1.86	STUB1	STIP1 homology and U-box containing protein 1, E3 ubiquitin protein lig	Downregulated
A_33_P324	0.001453	0.000171	-1.86	SNORA23	small nucleolar RNA, H/ACA box 23	Downregulated
A_33_P334	0.001839	0.000259	-1.86	lnc-WWP2-1	lnc-WWP2-1:1	Downregulated
A_23_P455	0.001916	0.000278	-1.86	PPP2R3B	protein phosphatase 2, regulatory subunit B'', beta	Downregulated
A_33_P320	0.002033	0.00031	-1.86	RASGRP2	RAS guanyl releasing protein 2 (calcium and DAG-regulated)	Downregulated
A_23_P153	0.002242	0.000365	-1.86	PPP5C	protein phosphatase 5, catalytic subunit	Downregulated
A_23_P232	0.00261	0.000459	-1.86	BLZF1	basic leucine zipper nuclear factor 1	Downregulated
A_21_P001	0.006435	0.00175	-1.86	LINC01474	long intergenic non-protein coding RNA 1474	Downregulated
A_23_P174	0.02294	0.00921	-1.86	TMEM230	transmembrane protein 230	Downregulated
A_33_P324	0.000786	0.0000453	-1.87	ZNF346	zinc finger protein 346	Downregulated
A_23_P372	0.001007	0.0000813	-1.87	CIPC	CLOCK-interacting pacemaker	Downregulated
A_33_P323	0.00174	0.000234	-1.87	ANKRD54	ankyrin repeat domain 54	Downregulated
A_23_P418	0.0018	0.000248	-1.87	C11orf65	chromosome 11 open reading frame 65	Downregulated
A_23_P359	0.001947	0.000285	-1.87	DNAH2	dynein, axonemal, heavy chain 2	Downregulated
A_21_P000	0.002166	0.000345	-1.87	SAP30L-AS1	SAP30L antisense RNA 1 (head to head)	Downregulated
A_23_P310	0.002855	0.000526	-1.87	C19orf52	chromosome 19 open reading frame 52	Downregulated
A_24_P568	0.003011	0.000571	-1.87	CRLF3	cytokine receptor-like factor 3	Downregulated
A_23_P211	0.003846	0.000826	-1.87	ABHD6	abhydrolase domain containing 6	Downregulated
A_33_P328	0.005623	0.00144	-1.87	PLCL1	phospholipase C-like 1	Downregulated
A_21_P000	0.00872	0.00266	-1.87	CNPY2	canopy FGF signaling regulator 2	Downregulated
A_22_P000	0.010142	0.00326	-1.87	lnc-TMEM14E	lnc-TMEM14E-2:1	Downregulated
A_22_P000	0.015057	0.00545	-1.87	lnc-PHC3-1	lnc-PHC3-1:1	Downregulated
A_21_P001	0.000801	0.0000478	-1.88	PLA2G10	phospholipase A2, group X	Downregulated
A_23_P323	0.000819	0.0000505	-1.88	PPP1CA	protein phosphatase 1, catalytic subunit, alpha isozyme	Downregulated
A_23_P351	0.000839	0.000053	-1.88	ALG6	ALG6, alpha-1,3-glucosyltransferase	Downregulated
A_33_P337	0.000977	0.0000753	-1.88	KLHDC4	kelch domain containing 4	Downregulated
A_33_P333	0.001112	0.000102	-1.88	GOLGA2P5	golgin A2 pseudogene 5	Downregulated
A_23_P830	0.001171	0.000113	-1.88	TLE4	transducin-like enhancer of split 4	Downregulated

A_23_P323	0.001211	0.000121	-1.88	LYRM4	LYR motif containing 4	Downregulated
A_33_P339	0.001227	0.000124	-1.88	INO80E	INO80 complex subunit E	Downregulated
A_24_P192	0.001688	0.000222	-1.88	FADS1	fatty acid desaturase 1	Downregulated
A_21_P000	0.001816	0.000254	-1.88	PITPNA-AS1	PITPNA antisense RNA 1	Downregulated
A_24_P651	0.002219	0.000358	-1.88	CDK10	cyclin-dependent kinase 10	Downregulated
A_32_P141	0.00302	0.000574	-1.88	COMMD7	COMM domain containing 7	Downregulated
A_33_P339	0.003103	0.000599	-1.88	FAM168B	family with sequence similarity 168, member B	Downregulated
A_22_P000	0.003421	0.000694	-1.88	lnc-PKN2-1	lnc-PKN2-1:1	Downregulated
A_24_P712	0.00383	0.000821	-1.88	PIK3CD	phosphatidylinositol-4,5-bisphosphate 3-kinase, catalytic subunit delta	Downregulated
A_33_P329	0.005674	0.00146	-1.88	C11orf49	chromosome 11 open reading frame 49	Downregulated
A_21_P000	0.009704	0.00307	-1.88	MAPKAPK5-A	MAPKAPK5 antisense RNA 1	Downregulated
A_23_P159	0.012122	0.0041	-1.88	CD8B	CD8b molecule	Downregulated
A_23_P831	0.016087	0.00592	-1.88	CDK5RAP2	CDK5 regulatory subunit associated protein 2	Downregulated
A_33_P324	0.000752	0.0000408	-1.89	SLC39A4	solute carrier family 39 (zinc transporter), member 4	Downregulated
A_33_P322	0.000763	0.000042	-1.89	SNORD3B-1	small nucleolar RNA, C/D box 3B-1	Downregulated
A_22_P000	0.000799	0.0000474	-1.89	lnc-CENPBD1	lnc-CENPBD1-1:1	Downregulated
A_23_P570	0.000905	0.0000621	-1.89	STAU1	staufen double-stranded RNA binding protein 1	Downregulated
A_23_P383	0.000995	0.0000783	-1.89	DHX58	DEXH (Asp-Glu-X-His) box polypeptide 58	Downregulated
A_23_P322	0.001026	0.0000843	-1.89	EFNA4	ephrin-A4	Downregulated
A_23_P415	0.001067	0.0000927	-1.89	PRMT9	protein arginine methyltransferase 9	Downregulated
A_33_P323	0.001359	0.00015	-1.89	EDEM3	ER degradation enhancer, mannosidase alpha-like 3	Downregulated
A_21_P001	0.001563	0.000194	-1.89	LOC285638	uncharacterized LOC285638	Downregulated
A_23_P425	0.002871	0.00053	-1.89	MED11	mediator complex subunit 11	Downregulated
A_22_P000	0.004984	0.00121	-1.89	CDK3	cyclin-dependent kinase 3	Downregulated
A_32_P221	0.005249	0.0013	-1.89	RRP15	ribosomal RNA processing 15 homolog (S. cerevisiae)	Downregulated
A_33_P321	0.006496	0.00177	-1.89	RFC3	replication factor C (activator 1) 3, 38kDa	Downregulated
A_23_P103	0.025445	0.0105	-1.89	TTC13	tetratricopeptide repeat domain 13	Downregulated
A_33_P329	0.026117	0.0109	-1.89	PRRC2B	proline-rich coiled-coil 2B	Downregulated
A_23_P118	0.000919	0.0000645	-1.9	FAHD1	fumarylacetoacetate hydrolase domain containing 1	Downregulated
A_24_P380	0.000949	0.0000696	-1.9	SMAP1	small ArfGAP 1	Downregulated
A_24_P262	0.001062	0.0000912	-1.9	DDA1	DET1 and DDB1 associated 1	Downregulated
A_24_P445	0.00121	0.000121	-1.9	MON1B	MON1 secretory trafficking family member B	Downregulated
A_23_P131	0.002149	0.000341	-1.9	MANBAL	mannosidase, beta A, lysosomal-like	Downregulated
A_24_P394	0.002786	0.000506	-1.9	SFXN1	sideroflexin 1	Downregulated
A_23_P300	0.002804	0.000511	-1.9	CCNY	cyclin Y	Downregulated
A_33_P337	0.004435	0.00102	-1.9	NR2C2	nuclear receptor subfamily 2, group C, member 2	Downregulated
A_23_P621	0.027476	0.0116	-1.9	ZC4H2	zinc finger, C4H2 domain containing	Downregulated

A_23_P119	0.000758	0.0000414	-1.91	EMP3	epithelial membrane protein 3	Downregulated
A_33_P339	0.00076	0.0000416	-1.91	POLR2G	polymerase (RNA) II (DNA directed) polypeptide G	Downregulated
A_22_P000	0.000764	0.0000422	-1.91	lnc-SYNC-2	lnc-SYNC-2:1	Downregulated
A_23_P259	0.000788	0.0000457	-1.91	ZBP1	Z-DNA binding protein 1	Downregulated
A_23_P745	0.000788	0.0000458	-1.91	CD53	CD53 molecule	Downregulated
A_23_P318	0.000838	0.0000528	-1.91	ZAK	sterile alpha motif and leucine zipper containing kinase AZK	Downregulated
A_23_P253	0.000853	0.0000549	-1.91	AUP1	ancient ubiquitous protein 1	Downregulated
A_23_P205	0.000875	0.0000582	-1.91	ABHD4	abhydrolase domain containing 4	Downregulated
A_22_P000	0.000909	0.0000631	-1.91	DPYS	dihydropyrimidinase	Downregulated
A_32_P184	0.000961	0.000072	-1.91	ARID5B	AT rich interactive domain 5B (MRF1-like)	Downregulated
A_23_P127	0.000964	0.0000728	-1.91	TUBGCP2	tubulin, gamma complex associated protein 2	Downregulated
A_24_P205	0.001079	0.0000949	-1.91	ACOT7	acyl-CoA thioesterase 7	Downregulated
A_33_P386	0.001187	0.000116	-1.91	TRABD	TraB domain containing	Downregulated
A_32_P358	0.001243	0.000128	-1.91	ICE1	interactor of little elongation complex ELL subunit 1	Downregulated
A_32_P163	0.001851	0.000262	-1.91	SPDYE3	speedy/RINGO cell cycle regulator family member E3	Downregulated
A_33_P339	0.001987	0.000297	-1.91	SMG1	SMG1 phosphatidylinositol 3-kinase-related kinase	Downregulated
A_22_P000	0.002956	0.000555	-1.91	ZRANB1	zinc finger, RAN-binding domain containing 1	Downregulated
A_24_P387	0.003163	0.000618	-1.91	PKN2	protein kinase N2	Downregulated
A_24_P282	0.049228	0.0236	-1.91	ABL1	ABL proto-oncogene 1, non-receptor tyrosine kinase	Downregulated
A_33_P328	0.000739	0.0000392	-1.92	IGFL1	IGF-like family member 1	Downregulated
A_33_P325	0.001087	0.0000963	-1.92	MAPKAP1	mitogen-activated protein kinase associated protein 1	Downregulated
A_33_P323	0.001376	0.000153	-1.92	TET2	tet methylcytosine dioxygenase 2	Downregulated
A_24_P368	0.001756	0.000238	-1.92	ARL10	ADP-ribosylation factor-like 10	Downregulated
A_32_P112	0.003657	0.000766	-1.92	CHTF8	CTF8, chromosome transmission fidelity factor 8 homolog (S. cerevisiae)	Downregulated
A_32_P100	0.004771	0.00113	-1.92	LOC1019279	uncharacterized LOC101927974	Downregulated
A_33_P334	0.006202	0.00166	-1.92	MGME1	mitochondrial genome maintenance exonuclease 1	Downregulated
A_23_P567	0.006349	0.00172	-1.92	HNMT	histamine N-methyltransferase	Downregulated
A_33_P322	0.012104	0.0041	-1.92	NAT8B	N-acetyltransferase 8B (GCN5-related, putative, gene/pseudogene)	Downregulated
A_22_P000	0.01765	0.00666	-1.92	ZBTB20-AS1	ZBTB20 antisense RNA 1	Downregulated
A_22_P000	0.000807	0.0000487	-1.93	lnc-SLC15A1-	lnc-SLC15A1-1:1	Downregulated
A_33_P328	0.000821	0.0000509	-1.93	RASA2	RAS p21 protein activator 2	Downregulated
A_23_P153	0.001069	0.0000936	-1.93	RALBP1	ralA binding protein 1	Downregulated
A_23_P989	0.001108	0.000101	-1.93	LRMP	lymphoid-restricted membrane protein	Downregulated
A_33_P383	0.001333	0.000145	-1.93	POU2F2	POU class 2 homeobox 2	Downregulated
A_23_P888	0.001543	0.000189	-1.93	DEF8	differentially expressed in FDCP 8 homolog (mouse)	Downregulated
A_22_P000	0.001688	0.000222	-1.93	lnc-C17orf48-	lnc-C17orf48-1:1	Downregulated
A_33_P323	0.002908	0.000541	-1.93	FAM216A	family with sequence similarity 216, member A	Downregulated

A_23_P105	0.003609	0.000752	-1.93	APPL2	adaptor protein, phosphotyrosine interaction, PH domain and leucine zi	Downregulated
A_22_P000	0.003609	0.000752	-1.93	lnc-DBN1-1	lnc-DBN1-1:1	Downregulated
A_33_P335	0.004373	0.001	-1.93	CCSAP	centriole, cilia and spindle-associated protein	Downregulated
A_21_P001	0.004493	0.00104	-1.93	LOC285626	uncharacterized LOC285626	Downregulated
A_23_P530	0.0046	0.00107	-1.93	ZNF215	zinc finger protein 215	Downregulated
A_23_P145	0.005651	0.00145	-1.93	TPK1	thiamin pyrophosphokinase 1	Downregulated
A_22_P000	0.006986	0.00196	-1.93	lnc-DDI1-1	lnc-DDI1-1:1	Downregulated
A_22_P000	0.01198	0.00404	-1.93	LOC1019285	uncharacterized LOC101928573	Downregulated
A_33_P322	0.025791	0.0107	-1.93	ERCC1	excision repair cross-complementation group 1	Downregulated
A_21_P001	0.026743	0.0112	-1.93	XLOC_l2_014814		Downregulated
A_23_P666	0.000837	0.0000528	-1.94	EVI2B	ecotropic viral integration site 2B	Downregulated
A_33_P334	0.000864	0.0000568	-1.94	APTX	aprataxin	Downregulated
A_24_P237	0.000887	0.0000596	-1.94	SASH3	SAM and SH3 domain containing 3	Downregulated
A_21_P000	0.000915	0.0000641	-1.94	lnc-TMCC1-4	lnc-TMCC1-4:1	Downregulated
A_33_P328	0.000971	0.000074	-1.94	TRAF3IP3	TRAF3 interacting protein 3	Downregulated
A_23_P700	0.000998	0.0000797	-1.94	PPAP2A	phosphatidic acid phosphatase type 2A	Downregulated
A_33_P329	0.001297	0.000138	-1.94	PLK1	polo-like kinase 1	Downregulated
A_23_P417	0.00165	0.000213	-1.94	IRF1	interferon regulatory factor 1	Downregulated
A_32_P201	0.001706	0.000228	-1.94	AMMECR1	Alport syndrome, mental retardation, midface hypoplasia and elliptocyto	Downregulated
A_33_P333	0.003798	0.00081	-1.94	LOC1001320	uncharacterized LOC100132077	Downregulated
A_33_P337	0.004648	0.00109	-1.94	DYNC1LI2	dynein, cytoplasmic 1, light intermediate chain 2	Downregulated
A_23_P195	0.004644	0.00109	-1.94	TMEM223	transmembrane protein 223	Downregulated
A_22_P000	0.005219	0.00129	-1.94	lnc-PCYOX1L	lnc-PCYOX1L-1:1	Downregulated
A_23_P138	0.006706	0.00185	-1.94	GPN2	GPN-loop GTPase 2	Downregulated
A_23_P765	0.000712	0.0000358	-1.95	ITGB7	integrin, beta 7	Downregulated
A_24_P100	0.000801	0.0000476	-1.95	BCAS3	breast carcinoma amplified sequence 3	Downregulated
A_23_P373	0.000805	0.0000484	-1.95	CIDEB	cell death-inducing DFFA-like effector b	Downregulated
A_23_P409	0.000808	0.0000489	-1.95	FAM83C	family with sequence similarity 83, member C	Downregulated
A_21_P001	0.000814	0.0000501	-1.95	EBLN3	endogenous Bornavirus-like nucleoprotein 3	Downregulated
A_23_P167	0.001055	0.0000898	-1.95	CENPC	centromere protein C	Downregulated
A_22_P000	0.001086	0.0000962	-1.95	lnc-NPDC1-1	lnc-NPDC1-1:1	Downregulated
A_23_P630	0.001398	0.000158	-1.95	CERS2	ceramide synthase 2	Downregulated
A_24_P119	0.001453	0.000171	-1.95	MYH15	myosin, heavy chain 15	Downregulated
A_23_P147	0.001563	0.000194	-1.95	RAB33A	RAB33A, member RAS oncogene family	Downregulated
A_23_P242	0.001626	0.000208	-1.95	MAGOHB	mago-nashi homolog B (Drosophila)	Downregulated
A_21_P000	0.001812	0.000252	-1.95	C5orf56	chromosome 5 open reading frame 56	Downregulated
A_33_P330	0.002361	0.000394	-1.95	ANKRD30BL	ankyrin repeat domain 30B-like	Downregulated

A_32_P269	0.003101	0.000599	-1.95	ERICH1	glutamate-rich 1	Downregulated
A_33_P334	0.006483	0.00177	-1.95	CEP83	centrosomal protein 83kDa	Downregulated
A_33_P359	0.007304	0.00209	-1.95	HABP4	hyaluronan binding protein 4	Downregulated
A_23_P170	0.00796	0.00235	-1.95	ZNF692	zinc finger protein 692	Downregulated
A_32_P178	0.013227	0.00458	-1.95	C16orf86	chromosome 16 open reading frame 86	Downregulated
A_22_P000	0.01384	0.00486	-1.95	lnc-RP11-43D	lnc-RP11-43D2.2.1-3:1	Downregulated
A_21_P000	0.01384	0.00486	-1.95	SNORD119	small nucleolar RNA, C/D box 119	Downregulated
A_33_P329	0.021484	0.00848	-1.95	ASB14	ankyrin repeat and SOCS box containing 14	Downregulated
A_23_P324	0.000963	0.0000726	-1.96	KIAA0355	KIAA0355	Downregulated
A_21_P000	0.001107	0.000101	-1.96	FLJ42351	uncharacterized LOC400999	Downregulated
A_23_P333	0.001274	0.000134	-1.96	ERGIC1	endoplasmic reticulum-golgi intermediate compartment (ERGIC) 1	Downregulated
A_21_P001	0.001395	0.000157	-1.96	LOC1001281	uncharacterized LOC100128108	Downregulated
A_24_P108	0.001693	0.000223	-1.96	KCTD17	potassium channel tetramerization domain containing 17	Downregulated
A_22_P000	0.001918	0.000278	-1.96	lnc-DLL1-3	lnc-DLL1-3:1	Downregulated
A_21_P000	0.003228	0.000637	-1.96	lnc-C12orf74-	lnc-C12orf74-1:1	Downregulated
A_33_P341	0.004718	0.00112	-1.96	PLXNA3	plexin A3	Downregulated
A_33_P322	0.009494	0.00298	-1.96	AMER1	APC membrane recruitment protein 1	Downregulated
A_33_P329	0.009651	0.00305	-1.96	PTPRCAP	protein tyrosine phosphatase, receptor type, C-associated protein	Downregulated
A_22_P000	0.013095	0.00453	-1.96	lnc-NPIPL2-2	lnc-NPIPL2-2:1	Downregulated
A_22_P000	0.000707	0.0000342	-1.97	lnc-TSPYL2-2	lnc-TSPYL2-2:1	Downregulated
A_23_P975	0.000718	0.0000368	-1.97	VAMP3	vesicle-associated membrane protein 3	Downregulated
A_22_P000	0.000758	0.0000414	-1.97	LOC1027245	uncharacterized LOC102724508	Downregulated
A_33_P324	0.001015	0.0000824	-1.97	CAMK2N2	calcium/calmodulin-dependent protein kinase II inhibitor 2	Downregulated
A_22_P000	0.00115	0.000109	-1.97	lnc-LUZP1-1	lnc-LUZP1-1:1	Downregulated
A_23_P211	0.001262	0.000132	-1.97	PAXBP1	PAX3 and PAX7 binding protein 1	Downregulated
A_33_P334	0.001562	0.000194	-1.97	TBL1X	transducin (beta)-like 1X-linked	Downregulated
A_24_P402	0.001647	0.000213	-1.97	ITM2C	integral membrane protein 2C	Downregulated
A_32_P231	0.003469	0.000708	-1.97	GOLGA6L9	golgin A6 family-like 9	Downregulated
A_33_P337	0.004031	0.000882	-1.97	LOC1002708	uncharacterized LOC100270804	Downregulated
A_32_P170	0.005106	0.00125	-1.97	POLA1	polymerase (DNA directed), alpha 1, catalytic subunit	Downregulated
A_32_P721	0.014802	0.00533	-1.97	LINC00669	long intergenic non-protein coding RNA 669	Downregulated
A_23_P209	0.016063	0.00591	-1.97	COLGALT1	collagen beta(1-O)galactosyltransferase 1	Downregulated
A_22_P000	0.01721	0.00646	-1.97	lnc-CCDC69-1	lnc-CCDC69-1:1	Downregulated
A_23_P108	0.022843	0.00916	-1.97	TJP3	tight junction protein 3	Downregulated
A_21_P000	0.038546	0.0176	-1.97	lnc-RPS7-1	lnc-RPS7-1:6	Downregulated
A_24_P342	0.000709	0.000035	-1.98	RERE	arginine-glutamic acid dipeptide (RE) repeats	Downregulated
A_33_P326	0.000752	0.0000407	-1.98	CECR1	cat eye syndrome chromosome region, candidate 1	Downregulated

A_24_P322	0.000766	0.0000427	-1.98	IL10RB	interleukin 10 receptor, beta	Downregulated
A_33_P321	0.001006	0.0000808	-1.98	SEC13	SEC13 homolog (S. cerevisiae)	Downregulated
A_24_P108	0.001068	0.0000932	-1.98	RTTN	rotatin	Downregulated
A_21_P000	0.001235	0.000126	-1.98	lnc-RP11-736	lnc-RP11-736N17.6.1-1:1	Downregulated
A_23_P430	0.001313	0.000141	-1.98	ELP3	elongator acetyltransferase complex subunit 3	Downregulated
A_24_P233	0.001342	0.000147	-1.98	CEPT1	choline/ethanolamine phosphotransferase 1	Downregulated
A_23_P639	0.001913	0.000277	-1.98	WDR11	WD repeat domain 11	Downregulated
A_24_P244	0.002199	0.000354	-1.98	BSCL2	Berardinelli-Seip congenital lipodystrophy 2 (seipin)	Downregulated
A_33_P341	0.00608	0.00161	-1.98	ADAL	adenosine deaminase-like	Downregulated
A_22_P000	0.010112	0.00325	-1.98	lnc-SZT2-2	lnc-SZT2-2:1	Downregulated
A_33_P335	0.000707	0.000034	-1.99	ITGA4	integrin, alpha 4 (antigen CD49D, alpha 4 subunit of VLA-4 receptor)	Downregulated
A_23_P357	0.000737	0.0000388	-1.99	APOBEC3F	apolipoprotein B mRNA editing enzyme, catalytic polypeptide-like 3F	Downregulated
A_23_P273	0.000931	0.0000664	-1.99	SMAD4	SMAD family member 4	Downregulated
A_19_P003	0.000945	0.000069	-1.99	WDFY4	WDFY family member 4	Downregulated
A_22_P000	0.000995	0.0000788	-1.99	lnc-MED10-5	lnc-MED10-5:1	Downregulated
A_24_P636	0.001085	0.0000959	-1.99	STK17B	serine/threonine kinase 17b	Downregulated
A_21_P000	0.001395	0.000157	-1.99	lnc-SELV.1-1	lnc-SELV.1-1:1	Downregulated
A_21_P000	0.002068	0.000319	-1.99	lnc-SHH-1	lnc-SHH-1:1	Downregulated
A_33_P384	0.002566	0.000447	-1.99	SLC25A15	solute carrier family 25 (mitochondrial carrier; ornithine transporter) meml	Downregulated
A_33_P329	0.002995	0.000567	-1.99	LINC00894	long intergenic non-protein coding RNA 894	Downregulated
A_21_P001	0.00452	0.00105	-1.99	C19orf68	chromosome 19 open reading frame 68	Downregulated
A_33_P335	0.005261	0.00131	-1.99	GPATCH2	G patch domain containing 2	Downregulated
A_33_P322	0.011775	0.00396	-1.99	CD8A	CD8a molecule	Downregulated
A_23_P255	0.00082	0.0000508	-2	CCDC109B	coiled-coil domain containing 109B	Downregulated
A_23_P133	0.000848	0.0000544	-2	MRPS27	mitochondrial ribosomal protein S27	Downregulated
A_23_P356	0.000912	0.0000636	-2	FBXO11	F-box protein 11	Downregulated
A_19_P003	0.000995	0.000078	-2	SCARNA16	small Cajal body-specific RNA 16	Downregulated
A_22_P000	0.001041	0.0000868	-2	lnc-PINK1-2	lnc-PINK1-2:1	Downregulated
A_33_P326	0.001098	0.0000988	-2	ABHD17B	abhydrolase domain containing 17B	Downregulated
A_33_P339	0.00112	0.000103	-2	RPS27	ribosomal protein S27	Downregulated
A_22_P000	0.001172	0.000113	-2	lnc-NOS2-2	lnc-NOS2-2:1	Downregulated
A_23_P213	0.001178	0.000114	-2	PALLD	palladin, cytoskeletal associated protein	Downregulated
A_24_P475	0.001469	0.000174	-2	OPA3	optic atrophy 3 (autosomal recessive, with chorea and spastic paraplegi	Downregulated
A_23_P244	0.001695	0.000224	-2	DHCR7	7-dehydrocholesterol reductase	Downregulated
A_33_P336	0.001946	0.000285	-2	QKI	QKI, KH domain containing, RNA binding	Downregulated
A_22_P000	0.004668	0.0011	-2	lnc-AGMAT-1	lnc-AGMAT-1:1	Downregulated
A_22_P000	0.010871	0.00355	-2	LOC1019296	uncharacterized LOC101929641	Downregulated

A_22_P000	0.015182	0.0055	-2	lnc-TRIM63-1	lnc-TRIM63-1:1	Downregulated
A_33_P326	0.016602	0.00618	-2	OR2D3	olfactory receptor, family 2, subfamily D, member 3	Downregulated
A_23_P351	0.017088	0.0064	-2	NPHP4	nephronophthisis 4	Downregulated
A_23_P799	0.021538	0.00851	-2	PSMF1	proteasome (prosome, macropain) inhibitor subunit 1 (PI31)	Downregulated
A_23_P122	0.022882	0.00918	-2	TAF6	TAF6 RNA polymerase II, TATA box binding protein (TBP)-associated fa	Downregulated
A_24_P182	0.024104	0.00982	-2	KCNAB1	potassium channel, voltage gated subfamily A regulatory beta subunit 1	Downregulated
A_23_P320	0.000707	0.0000341	-2.01	MEF2C	myocyte enhancer factor 2C	Downregulated
A_23_P125	0.00072	0.000037	-2.01	TRAPPC1	trafficking protein particle complex 1	Downregulated
A_33_P334	0.000814	0.00005	-2.01	POMC	proopiomelanocortin	Downregulated
A_23_P131	0.000883	0.0000592	-2.01	PIGF	phosphatidylinositol glycan anchor biosynthesis, class F	Downregulated
A_33_P331	0.000909	0.000063	-2.01	PXYLP1	2-phosphoxylose phosphatase 1	Downregulated
A_23_P403	0.001035	0.0000858	-2.01	MAPRE1	microtubule-associated protein, RP/EB family, member 1	Downregulated
A_23_P217	0.001265	0.000132	-2.01	IDS	iduronate 2-sulfatase	Downregulated
A_24_P717	0.001453	0.00017	-2.01	DIP2A	DIP2 disco-interacting protein 2 homolog A (Drosophila)	Downregulated
A_23_P119	0.001453	0.00017	-2.01	TTC32	tetratricopeptide repeat domain 32	Downregulated
A_23_P133	0.00174	0.000234	-2.01	PREP	prolyl endopeptidase	Downregulated
A_24_P298	0.002112	0.00033	-2.01	PRMT5	protein arginine methyltransferase 5	Downregulated
A_33_P334	0.017831	0.00674	-2.01	TLCD2	TLC domain containing 2	Downregulated
A_23_P396	0.000792	0.0000465	-2.02	TBC1D2B	TBC1 domain family, member 2B	Downregulated
A_33_P321	0.000799	0.0000473	-2.02	DGKQ	diacylglycerol kinase, theta 110kDa	Downregulated
A_23_P414	0.000883	0.000059	-2.02	TRPC3	transient receptor potential cation channel, subfamily C, member 3	Downregulated
A_24_P376	0.000888	0.0000599	-2.02	MBD1	methyl-CpG binding domain protein 1	Downregulated
A_23_P207	0.000959	0.0000711	-2.02	PPY	pancreatic polypeptide	Downregulated
A_23_P603	0.001115	0.000102	-2.02	SMARCA2	SWI/SNF related, matrix associated, actin dependent regulator of chrom	Downregulated
A_23_P204	0.001299	0.000139	-2.02	PPP1CC	protein phosphatase 1, catalytic subunit, gamma isozyme	Downregulated
A_23_P164	0.001403	0.000159	-2.02	FKRP	fukutin related protein	Downregulated
A_23_P254	0.005232	0.0013	-2.02	TUBB6	tubulin, beta 6 class V	Downregulated
A_32_P761	0.000697	0.0000321	-2.03	RWDD4	RWD domain containing 4	Downregulated
A_23_P652	0.000707	0.0000345	-2.03	TMTC4	transmembrane and tetratricopeptide repeat containing 4	Downregulated
A_33_P331	0.000712	0.0000359	-2.03	INTS6	integrator complex subunit 6	Downregulated
A_21_P001	0.000718	0.0000368	-2.03	XLOC_l2_011987		Downregulated
A_23_P145	0.000744	0.00004	-2.03	BAK1	BCL2-antagonist/killer 1	Downregulated
A_21_P000	0.000959	0.0000712	-2.03	lnc-HMBOX1-	lnc-HMBOX1-1:1	Downregulated
A_24_P940	0.000964	0.0000729	-2.03	IPCEF1	interaction protein for cytohesin exchange factors 1	Downregulated
A_32_P466	0.000995	0.000079	-2.03	VWA3B	von Willebrand factor A domain containing 3B	Downregulated
A_23_P139	0.001321	0.000142	-2.03	ARFIP2	ADP-ribosylation factor interacting protein 2	Downregulated
A_24_P397	0.001382	0.000154	-2.03	ZC2HC1A	zinc finger, C2HC-type containing 1A	Downregulated

A_21_P001	0.001968	0.000292	-2.03	LINC01347	long intergenic non-protein coding RNA 1347	Downregulated
A_23_P147	0.002117	0.000332	-2.03	ZNF280D	zinc finger protein 280D	Downregulated
A_22_P000	0.002311	0.000381	-2.03	lnc-OSBPL9-2	lnc-OSBPL9-2:1	Downregulated
A_21_P001	0.00348	0.000712	-2.03	LINC00869	long intergenic non-protein coding RNA 869	Downregulated
A_33_P326	0.005233	0.0013	-2.03	ZDHHC2	zinc finger, DHHC-type containing 2	Downregulated
A_22_P000	0.006753	0.00187	-2.03	LOC153684	uncharacterized LOC153684	Downregulated
A_33_P323	0.00819	0.00244	-2.03	MAP3K7CL	MAP3K7 C-terminal like	Downregulated
A_23_P925	0.014847	0.00535	-2.03	SYK	spleen tyrosine kinase	Downregulated
A_23_P463	0.029806	0.0128	-2.03	TNFAIP8L2	tumor necrosis factor, alpha-induced protein 8-like 2	Downregulated
A_33_P338	0.000691	0.0000306	-2.04	SKAP2	src kinase associated phosphoprotein 2	Downregulated
A_33_P358	0.000699	0.0000323	-2.04	GNAI2	guanine nucleotide binding protein (G protein), alpha inhibiting activity p	Downregulated
A_32_P101	0.000764	0.0000422	-2.04	USP39	ubiquitin specific peptidase 39	Downregulated
A_23_P638	0.000967	0.0000735	-2.04	GOT1	glutamic-oxaloacetic transaminase 1, soluble	Downregulated
A_32_P881	0.001021	0.0000832	-2.04	YPEL1	yippee-like 1 (Drosophila)	Downregulated
A_23_P436	0.00124	0.000127	-2.04	MLLT4	myeloid/lymphoid or mixed-lineage leukemia (trithorax homolog, Drosoph	Downregulated
A_21_P001	0.001443	0.000168	-2.04	XLOC_l2_009134		Downregulated
A_22_P000	0.001847	0.000261	-2.04	lnc-ME3-1	lnc-ME3-1:1	Downregulated
A_33_P342	0.00235	0.00039	-2.04	GOLGA7	golgin A7	Downregulated
A_22_P000	0.003458	0.000705	-2.04	lnc-TFDP2-1	lnc-TFDP2-1:1	Downregulated
A_24_P142	0.010357	0.00335	-2.04	PI4KA	phosphatidylinositol 4-kinase, catalytic, alpha	Downregulated
A_22_P000	0.014118	0.00499	-2.04	ZBTB20	zinc finger and BTB domain containing 20	Downregulated
A_24_P153	0.017086	0.0064	-2.04	OSBPL8	oxysterol binding protein-like 8	Downregulated
A_33_P335	0.000696	0.0000317	-2.05	GPD2	glycerol-3-phosphate dehydrogenase 2 (mitochondrial)	Downregulated
A_33_P325	0.000697	0.0000321	-2.05	RPS23	ribosomal protein S23	Downregulated
A_33_P341	0.000781	0.0000446	-2.05	ADTRP	androgen-dependent TFPI-regulating protein	Downregulated
A_21_P000	0.000928	0.0000657	-2.05	SNORA11C	small nucleolar RNA, H/ACA box 11C	Downregulated
A_23_P205	0.000938	0.0000676	-2.05	BCL11B	B-cell CLL/lymphoma 11B (zinc finger protein)	Downregulated
A_21_P000	0.001043	0.0000873	-2.05	USP3-AS1	USP3 antisense RNA 1	Downregulated
A_32_P498	0.00139	0.000156	-2.05	RHOQ	ras homolog family member Q	Downregulated
A_24_P898	0.001699	0.000226	-2.05	CYHR1	cysteine/histidine-rich 1	Downregulated
A_23_P325	0.001699	0.000226	-2.05	SCAI	suppressor of cancer cell invasion	Downregulated
A_22_P000	0.00203	0.000309	-2.05	GAS5-AS1	GAS5 antisense RNA 1	Downregulated
A_33_P388	0.002705	0.000484	-2.05	PPA2	pyrophosphatase (inorganic) 2	Downregulated
A_33_P321	0.006067	0.00161	-2.05	CHIC1	cysteine-rich hydrophobic domain 1	Downregulated
A_33_P321	0.006352	0.00172	-2.05	RCOR1	REST corepressor 1	Downregulated
A_24_P276	0.000718	0.0000368	-2.06	RCOR3	REST corepressor 3	Downregulated
A_23_P809	0.000752	0.0000408	-2.06	RNF5	ring finger protein 5, E3 ubiquitin protein ligase	Downregulated

A_22_P000	0.000764	0.0000422	-2.06	SMARCC2	SWI/SNF related, matrix associated, actin dependent regulator of chrom	Downregulated
A_23_P593	0.000814	0.0000498	-2.06	HECA	headcase homolog (Drosophila)	Downregulated
A_22_P000	0.002246	0.000367	-2.06	RNF139-AS1	RNF139 antisense RNA 1 (head to head)	Downregulated
A_22_P000	0.002853	0.000525	-2.06	lnc-C6orf225-	lnc-C6orf225-1:1	Downregulated
A_24_P288	0.003525	0.000724	-2.06	SRRD	SRR1 domain containing	Downregulated
A_33_P681	0.00689	0.00193	-2.06	lnc-GGCT-1	lnc-GGCT-1:6	Downregulated
A_23_P601	0.008676	0.00264	-2.06	ABL1	ABL proto-oncogene 1, non-receptor tyrosine kinase	Downregulated
A_33_P332	0.011297	0.00374	-2.06	BDNF-AS	BDNF antisense RNA	Downregulated
A_33_P336	0.000743	0.0000397	-2.07	PTPRC	protein tyrosine phosphatase, receptor type, C	Downregulated
A_23_P426	0.000765	0.0000425	-2.07	GGCT	gamma-glutamylcyclotransferase	Downregulated
A_33_P332	0.000869	0.0000576	-2.07	CDK19	cyclin-dependent kinase 19	Downregulated
A_23_P345	0.000977	0.0000752	-2.07	RPAP1	RNA polymerase II associated protein 1	Downregulated
A_33_P349	0.000987	0.0000765	-2.07	lnc-AC01674	lnc-AC016745.1-2:1	Downregulated
A_23_P562	0.00104	0.0000867	-2.07	POLR2I	polymerase (RNA) II (DNA directed) polypeptide I, 14.5kDa	Downregulated
A_24_P493	0.001042	0.000087	-2.07	AAMDC	adipogenesis associated, Mth938 domain containing	Downregulated
A_33_P336	0.001611	0.000204	-2.07	KLF6	Kruppel-like factor 6	Downregulated
A_32_P173	0.001881	0.000269	-2.07	TMEM41B	transmembrane protein 41B	Downregulated
A_33_P321	0.002319	0.000383	-2.07	FAM230B	family with sequence similarity 230, member B (non-protein coding)	Downregulated
A_23_P369	0.003304	0.000661	-2.07	MSI2	musashi RNA-binding protein 2	Downregulated
A_22_P000	0.003649	0.000764	-2.07	lnc-COL9A2-1	lnc-COL9A2-1:1	Downregulated
A_33_P339	0.004219	0.000946	-2.07	IPCEF1	interaction protein for cytohesin exchange factors 1	Downregulated
A_23_P407	0.004288	0.000969	-2.07	DNM2	dynamin 2	Downregulated
A_24_P351	0.004894	0.00118	-2.07	GATAD1	GATA zinc finger domain containing 1	Downregulated
A_33_P326	0.000709	0.0000348	-2.08	NCK2	NCK adaptor protein 2	Downregulated
A_23_P154	0.000775	0.0000437	-2.08	NAGK	N-acetylglucosamine kinase	Downregulated
A_23_P122	0.000995	0.0000787	-2.08	DAXX	death-domain associated protein	Downregulated
A_21_P000	0.001095	0.0000979	-2.08	lnc-ZNF169-1	lnc-ZNF169-1:1	Downregulated
A_33_P336	0.001097	0.0000983	-2.08	KCTD5	potassium channel tetramerization domain containing 5	Downregulated
A_33_P325	0.002816	0.000515	-2.08	SH2D3A	SH2 domain containing 3A	Downregulated
A_33_P339	0.003175	0.000622	-2.08	FAM178A	family with sequence similarity 178, member A	Downregulated
A_24_P314	0.008684	0.00265	-2.08	GOLGA2P2Y	golgin A2 pseudogene 2, Y-linked	Downregulated
A_23_P743	0.014083	0.00497	-2.08	NUF2	NUF2, NDC80 kinetochore complex component	Downregulated
A_33_P337	0.019239	0.00741	-2.08	FGD5P1	FYVE, RhoGEF and PH domain containing 5 pseudogene 1	Downregulated
A_21_P000	0.000669	0.0000285	-2.09	RNU11	RNA, U11 small nuclear	Downregulated
A_33_P336	0.000706	0.0000335	-2.09	BSDC1	BSD domain containing 1	Downregulated
A_33_P337	0.000726	0.0000377	-2.09	TXLNA	taxilin alpha	Downregulated
A_23_P480	0.00074	0.0000394	-2.09	CD27	CD27 molecule	Downregulated

A_21_P001	0.000844	0.0000536	-2.09	LOC1019284	uncharacterized LOC101928464	Downregulated
A_22_P000	0.000998	0.0000798	-2.09	lnc-PRR15L-2	lnc-PRR15L-2:2	Downregulated
A_24_P274	0.00112	0.000103	-2.09	STAT1	signal transducer and activator of transcription 1, 91kDa	Downregulated
A_21_P000	0.001678	0.000219	-2.09	lnc-CCDC111	lnc-CCDC111-1:1	Downregulated
A_21_P000	0.002947	0.000551	-2.09	lnc-AMZ2-1	lnc-AMZ2-1:1	Downregulated
A_24_P912	0.003176	0.000623	-2.09	KIAA0754	KIAA0754	Downregulated
A_33_P335	0.004396	0.00101	-2.09	VAC14-AS1	VAC14 antisense RNA 1	Downregulated
A_23_P897	0.005822	0.00152	-2.09	CTDP1	CTD (carboxy-terminal domain, RNA polymerase II, polypeptide A) phos	Downregulated
A_23_P544	0.013794	0.00484	-2.09	ACSBG1	acyl-CoA synthetase bubblegum family member 1	Downregulated
A_21_P000	0.016316	0.00604	-2.09	lnc-MIB2-1	lnc-MIB2-1:5	Downregulated
A_24_P112	0.021843	0.00867	-2.09	ENTPD7	ectonucleoside triphosphate diphosphohydrolase 7	Downregulated
A_33_P728	0.000707	0.0000343	-2.1	LOC401320	uncharacterized LOC401320	Downregulated
A_23_P106	0.000715	0.0000363	-2.1	HSPBP1	HSPA (heat shock 70kDa) binding protein, cytoplasmic cochaperone 1	Downregulated
A_33_P340	0.000721	0.0000373	-2.1	HAS3	hyaluronan synthase 3	Downregulated
A_23_P104	0.000739	0.0000392	-2.1	CPT1A	carnitine palmitoyltransferase 1A (liver)	Downregulated
A_32_P121	0.000864	0.0000568	-2.1	DOK3	docking protein 3	Downregulated
A_21_P000	0.000866	0.0000572	-2.1	SNORA4	small nucleolar RNA, H/ACA box 4	Downregulated
A_23_P357	0.001043	0.0000873	-2.1	ANXA6	annexin A6	Downregulated
A_33_P322	0.00105	0.0000886	-2.1	CD151	CD151 molecule (Raph blood group)	Downregulated
A_23_P776	0.00112	0.000103	-2.1	MAP1LC3B	microtubule-associated protein 1 light chain 3 beta	Downregulated
A_23_P322	0.001138	0.000107	-2.1	KLF4	Kruppel-like factor 4 (gut)	Downregulated
A_23_P384	0.001424	0.000163	-2.1	CCDC14	coiled-coil domain containing 14	Downregulated
A_33_P334	0.002055	0.000316	-2.1	JMJD7	jumonji domain containing 7	Downregulated
A_23_P200	0.002237	0.000363	-2.1	NADK	NAD kinase	Downregulated
A_23_P394	0.002243	0.000366	-2.1	ALKBH6	alkB, alkylation repair homolog 6 (E. coli)	Downregulated
A_21_P001	0.003023	0.000575	-2.1	XLOC_l2_013857		Downregulated
A_23_P518	0.003278	0.000652	-2.1	PUSL1	pseudouridylate synthase-like 1	Downregulated
A_33_P328	0.006575	0.00181	-2.1	CTNS	cystinosin, lysosomal cystine transporter	Downregulated
A_24_P353	0.008507	0.00257	-2.1	LIMK2	LIM domain kinase 2	Downregulated
A_24_P694	0.000709	0.0000351	-2.11	SLC25A32	solute carrier family 25 (mitochondrial folate carrier), member 32	Downregulated
A_21_P000	0.00075	0.0000404	-2.11	LOC1019273	uncharacterized LOC101927334	Downregulated
A_24_P410	0.000813	0.0000496	-2.11	VGLL4	vestigial-like family member 4	Downregulated
A_22_P000	0.00094	0.0000682	-2.11	lnc-HIST1H3F	lnc-HIST1H3F-1:1	Downregulated
A_33_P336	0.001131	0.000105	-2.11	BLVRB	biliverdin reductase B	Downregulated
A_23_P159	0.001196	0.000118	-2.11	SNRNP35	small nuclear ribonucleoprotein 35kDa (U11/U12)	Downregulated
A_23_P138	0.001215	0.000122	-2.11	C10orf95	chromosome 10 open reading frame 95	Downregulated
A_23_P120	0.001264	0.000132	-2.11	MTX2	metaxin 2	Downregulated

A_21_P000	0.002744	0.000495	-2.11	SNORD34	small nucleolar RNA, C/D box 34	Downregulated
A_33_P350	0.00433	0.000985	-2.11	CASP10	caspase 10, apoptosis-related cysteine peptidase	Downregulated
A_21_P000	0.004816	0.00115	-2.11	lnc-AC07409	lnc-AC074091.13.1-1:1	Downregulated
A_23_P189	0.005872	0.00153	-2.11	MIER3	mesoderm induction early response 1, family member 3	Downregulated
A_21_P001	0.008161	0.00243	-2.11	LOC1005074	uncharacterized LOC100507460	Downregulated
A_33_P330	0.011279	0.00374	-2.11	RBM20	RNA binding motif protein 20	Downregulated
A_24_P153	0.011931	0.00402	-2.11	TRIM37	tripartite motif containing 37	Downregulated
A_24_P920	0.023878	0.0097	-2.11	COMMD6	COMM domain containing 6	Downregulated
A_23_P250	0.000848	0.0000543	-2.12	ST3GAL6	ST3 beta-galactoside alpha-2,3-sialyltransferase 6	Downregulated
A_24_P295	0.000855	0.0000555	-2.12	RASSF4	Ras association (RalGDS/AF-6) domain family member 4	Downregulated
A_21_P000	0.000857	0.0000558	-2.12	HMGA1P7	high mobility group AT-hook 1 pseudogene 7	Downregulated
A_24_P381	0.000959	0.0000714	-2.12	ITM2B	integral membrane protein 2B	Downregulated
A_21_P000	0.001163	0.000112	-2.12	LINC01138	long intergenic non-protein coding RNA 1138	Downregulated
A_32_P165	0.00203	0.000309	-2.12	WDR92	WD repeat domain 92	Downregulated
A_21_P000	0.004687	0.00111	-2.12	lnc-H3F3C-1	lnc-H3F3C-1:1	Downregulated
A_21_P001	0.01356	0.00474	-2.12	XLOC_l2_003647		Downregulated
A_21_P000	0.015921	0.00584	-2.12	lnc-AC01231	lnc-AC012313.1-1:4	Downregulated
A_21_P000	0.000647	0.0000267	-2.13	BTN2A1	butyrophilin, subfamily 2, member A1	Downregulated
A_23_P612	0.000693	0.0000309	-2.13	FAM207A	family with sequence similarity 207, member A	Downregulated
A_23_P201	0.000792	0.0000465	-2.13	LAMC1	laminin, gamma 1 (formerly LAMB2)	Downregulated
A_23_P282	0.000808	0.0000489	-2.13	ACTR1B	ARP1 actin-related protein 1 homolog B, centractin beta (yeast)	Downregulated
A_22_P000	0.00112	0.000103	-2.13	lnc-IL15RA-1	lnc-IL15RA-1:1	Downregulated
A_21_P000	0.002101	0.000327	-2.13	lnc-GOLGA8	lnc-GOLGA8J-1:1	Downregulated
A_32_P416	0.003175	0.000622	-2.13	F5	coagulation factor V (proaccelerin, labile factor)	Downregulated
A_33_P337	0.005033	0.00122	-2.13	PDE5A	phosphodiesterase 5A, cGMP-specific	Downregulated
A_23_P112	0.005093	0.00124	-2.13	DNAJB5	DnaJ (Hsp40) homolog, subfamily B, member 5	Downregulated
A_33_P323	0.017742	0.0067	-2.13	AGFG1	ArfGAP with FG repeats 1	Downregulated
A_33_P322	0.000693	0.0000309	-2.14	DAPK3	death-associated protein kinase 3	Downregulated
A_32_P209	0.000699	0.0000323	-2.14	CIITA	class II, major histocompatibility complex, transactivator	Downregulated
A_23_P428	0.000739	0.0000392	-2.14	CHCHD4	coiled-coil-helix-coiled-coil-helix domain containing 4	Downregulated
A_24_P273	0.000754	0.000041	-2.14	EML4	echinoderm microtubule associated protein like 4	Downregulated
A_23_P156	0.000888	0.0000599	-2.14	CNOT4	CCR4-NOT transcription complex, subunit 4	Downregulated
A_24_P274	0.000963	0.0000726	-2.14	ARRDC3	arrestin domain containing 3	Downregulated
A_23_P237	0.001085	0.0000959	-2.14	VPS45	vacuolar protein sorting 45 homolog (S. cerevisiae)	Downregulated
A_33_P338	0.001092	0.0000975	-2.14	HSPA6	heat shock 70kDa protein 6 (HSP70B')	Downregulated
A_21_P001	0.001265	0.000132	-2.14	XLOC_l2_005490		Downregulated
A_21_P001	0.001386	0.000155	-2.14	C9orf3	chromosome 9 open reading frame 3	Downregulated

A_24_P971	0.002219	0.000358	-2.14	CTDSP1	CTD (carboxy-terminal domain, RNA polymerase II, polypeptide A) small	Downregulated
A_23_P218	0.00239	0.000401	-2.14	CAPN3	calpain 3, (p94)	Downregulated
A_23_P153	0.002773	0.000503	-2.14	CERS4	ceramide synthase 4	Downregulated
A_24_P137	0.00299	0.000565	-2.14	USP53	ubiquitin specific peptidase 53	Downregulated
A_23_P142	0.004315	0.000979	-2.14	C2orf69	chromosome 2 open reading frame 69	Downregulated
A_24_P296	0.00615	0.00164	-2.14	COG3	component of oligomeric golgi complex 3	Downregulated
A_33_P336	0.008528	0.00258	-2.14	MAP3K1	mitogen-activated protein kinase kinase kinase 1, E3 ubiquitin protein li	Downregulated
A_33_P336	0.019992	0.00777	-2.14	NEDD9	neural precursor cell expressed, developmentally down-regulated 9	Downregulated
A_24_P346	0.000848	0.0000544	-2.15	TMEM248	transmembrane protein 248	Downregulated
A_23_P213	0.001056	0.00009	-2.15	GFM2	G elongation factor, mitochondrial 2	Downregulated
A_23_P217	0.001141	0.000108	-2.15	KLF8	Kruppel-like factor 8	Downregulated
A_23_P215	0.003406	0.00069	-2.15	KIAA1429	KIAA1429	Downregulated
A_22_P000	0.006185	0.00165	-2.15	lnc-GTDC1-5	lnc-GTDC1-5:2	Downregulated
A_32_P191	0.006847	0.00191	-2.15	LINC00265	long intergenic non-protein coding RNA 265	Downregulated
A_23_P301	0.014292	0.00508	-2.15	PAIP2B	poly(A) binding protein interacting protein 2B	Downregulated
A_21_P001	0.000642	0.0000262	-2.16	PPP2R5C	protein phosphatase 2, regulatory subunit B', gamma	Downregulated
A_23_P367	0.00065	0.000027	-2.16	SNX29	sorting nexin 29	Downregulated
A_23_P127	0.000738	0.0000389	-2.16	POLD4	polymerase (DNA-directed), delta 4, accessory subunit	Downregulated
A_23_P232	0.000971	0.0000741	-2.16	RCSD1	RCSD domain containing 1	Downregulated
A_22_P000	0.001129	0.000105	-2.16	KDM4A-AS1	KDM4A antisense RNA 1	Downregulated
A_33_P334	0.001337	0.000145	-2.16	LOC1001322	uncharacterized LOC100132249	Downregulated
A_24_P562	0.001494	0.000179	-2.16	DYRK2	dual-specificity tyrosine-(Y)-phosphorylation regulated kinase 2	Downregulated
A_33_P321	0.00163	0.000209	-2.16	RBM33	RNA binding motif protein 33	Downregulated
A_23_P654	0.001702	0.000227	-2.16	TEP1	telomerase-associated protein 1	Downregulated
A_22_P000	0.001889	0.000271	-2.16	LOC1005072	uncharacterized LOC100507250	Downregulated
A_23_P207	0.00308	0.000591	-2.16	ATP2A3	ATPase, Ca++ transporting, ubiquitous	Downregulated
A_33_P323	0.003875	0.000835	-2.16	MAP2K7	mitogen-activated protein kinase kinase 7	Downregulated
A_33_P332	0.003985	0.000868	-2.16	TNFRSF1B	tumor necrosis factor receptor superfamily, member 1B	Downregulated
A_24_P193	0.005861	0.00153	-2.16	KLRK1	killer cell lectin-like receptor subfamily K, member 1	Downregulated
A_22_P000	0.006363	0.00172	-2.16	JARID2-AS1	JARID2 antisense RNA 1	Downregulated
A_32_P194	0.008234	0.00246	-2.16	TECPR1	tectonin beta-propeller repeat containing 1	Downregulated
A_33_P331	0.000805	0.0000483	-2.17	WDR91	WD repeat domain 91	Downregulated
A_23_P200	0.00198	0.000295	-2.17	SHC1	SHC (Src homology 2 domain containing) transforming protein 1	Downregulated
A_33_P342	0.002243	0.000366	-2.17	GRHL1	grainyhead-like 1 (Drosophila)	Downregulated
A_33_P324	0.002279	0.000374	-2.17	CALHM3	calcium homeostasis modulator 3	Downregulated
A_22_P000	0.002528	0.000437	-2.17	lnc-PRMT8-2	lnc-PRMT8-2:1	Downregulated
A_32_P117	0.003389	0.000685	-2.17	TRIQK	triple QxxK/R motif containing	Downregulated

A_33_P330	0.004269	0.000963	-2.17	STRA6	stimulated by retinoic acid 6	Downregulated
A_33_P335	0.006863	0.00191	-2.17	C1orf194	chromosome 1 open reading frame 194	Downregulated
A_23_P395	0.007703	0.00225	-2.17	C2orf76	chromosome 2 open reading frame 76	Downregulated
A_33_P323	0.009012	0.00278	-2.17	RPP30	ribonuclease P/MRP 30kDa subunit	Downregulated
A_23_P212	0.011519	0.00384	-2.17	TRIM37	tripartite motif containing 37	Downregulated
A_23_P102	0.028637	0.0122	-2.17	CEP250	centrosomal protein 250kDa	Downregulated
A_23_P770	0.000626	0.0000247	-2.18	SNRPN	small nuclear ribonucleoprotein polypeptide N	Downregulated
A_33_P322	0.00063	0.000025	-2.18	NDOR1	NADPH dependent diflavin oxidoreductase 1	Downregulated
A_23_P385	0.000701	0.0000327	-2.18	PAOX	polyamine oxidase (exo-N4-amino)	Downregulated
A_23_P134	0.000712	0.0000358	-2.18	RBM12B-AS1	RBM12B antisense RNA 1	Downregulated
A_33_P370	0.000746	0.0000402	-2.18	DPY19L3	dpy-19-like 3 (C. elegans)	Downregulated
A_23_P627	0.000824	0.0000511	-2.18	CCDC28B	coiled-coil domain containing 28B	Downregulated
A_22_P000	0.000858	0.0000561	-2.18	lnc-RP11-148	lnc-RP11-148O21.2.1-2:1	Downregulated
A_23_P217	0.000938	0.0000677	-2.18	BRCC3	BRCA1/BRCA2-containing complex, subunit 3	Downregulated
A_33_P335	0.000971	0.0000743	-2.18	SLFN14	schlafen family member 14	Downregulated
A_22_P000	0.001341	0.000146	-2.18	lnc-SHANK2-[illegible]	lnc-SHANK2-5:2	Downregulated
A_24_P832	0.001381	0.000154	-2.18	FAM120AOS	family with sequence similarity 120A opposite strand	Downregulated
A_24_P184	0.001808	0.000251	-2.18	COCH	cochlin	Downregulated
A_23_P615	0.014995	0.00542	-2.18	NSUN6	NOP2/Sun domain family, member 6	Downregulated
A_33_P333	0.000722	0.0000374	-2.19	IFNAR2	interferon (alpha, beta and omega) receptor 2	Downregulated
A_33_P323	0.000755	0.0000411	-2.19	UTRN	utrophin	Downregulated
A_23_P214	0.000937	0.0000669	-2.19	REV3L	REV3-like, polymerase (DNA directed), zeta, catalytic subunit	Downregulated
A_24_P299	0.000942	0.0000685	-2.19	ZBTB18	zinc finger and BTB domain containing 18	Downregulated
A_33_P380	0.001126	0.000105	-2.19	TRAF2	TNF receptor-associated factor 2	Downregulated
A_21_P000	0.001369	0.000152	-2.19	SNORA6	small nucleolar RNA, H/ACA box 6	Downregulated
A_24_P400	0.003125	0.000606	-2.19	SMCHD1	structural maintenance of chromosomes flexible hinge domain containin	Downregulated
A_24_P622	0.003543	0.000731	-2.19	CERK	ceramide kinase	Downregulated
A_23_P251	0.005608	0.00144	-2.19	SNTG2	syntrophin, gamma 2	Downregulated
A_22_P000	0.009491	0.00297	-2.19	lnc-RMND5A-[illegible]	lnc-RMND5A-1:2	Downregulated
A_23_P413	0.020076	0.00781	-2.19	POC5	POC5 centriolar protein	Downregulated
A_23_P117	0.000626	0.0000247	-2.2	CLN6	ceroid-lipofuscinosis, neuronal 6, late infantile, variant	Downregulated
A_21_P001	0.000705	0.0000332	-2.2	C6orf62	chromosome 6 open reading frame 62	Downregulated
A_23_P786	0.000739	0.0000391	-2.2	DENND1C	DENN/MADD domain containing 1C	Downregulated
A_33_P324	0.000858	0.000056	-2.2	RALGAPB	Ral GTPase activating protein, beta subunit (non-catalytic)	Downregulated
A_24_P678	0.001654	0.000214	-2.2	STMN3	stathmin-like 3	Downregulated
A_33_P336	0.002352	0.000391	-2.2	SLC35B2	solute carrier family 35 (adenosine 3'-phospho 5'-phosphosulfate transp	Downregulated
A_24_P655	0.002544	0.000442	-2.2	OIP5-AS1	OIP5 antisense RNA 1	Downregulated

A_23_P119	0.00284	0.000522	-2.2	TMEM185B	transmembrane protein 185B	Downregulated
A_21_P000	0.006029	0.00159	-2.2	LINC01364	long intergenic non-protein coding RNA 1364	Downregulated
A_21_P000	0.006384	0.00173	-2.2	LOC1005076	uncharacterized LOC100507651	Downregulated
A_33_P322	0.007361	0.00211	-2.2	ITGA4	integrin, alpha 4 (antigen CD49D, alpha 4 subunit of VLA-4 receptor)	Downregulated
A_22_P000	0.014764	0.00531	-2.2	lnc-C5orf35-1	lnc-C5orf35-1:1	Downregulated
A_23_P502	0.018525	0.00707	-2.2	RASAL2	RAS protein activator like 2	Downregulated
A_24_P206	0.00077	0.0000431	-2.21	SNX15	sorting nexin 15	Downregulated
A_21_P000	0.003085	0.000593	-2.21	lnc-AC02456:	lnc-AC024563.1-2:1	Downregulated
A_22_P000	0.003687	0.000776	-2.21	lnc-CCDC83-1	lnc-CCDC83-1:1	Downregulated
A_24_P251	0.004003	0.000874	-2.21	ABCF3	ATP-binding cassette, sub-family F (GCN20), member 3	Downregulated
A_21_P001	0.01064	0.00346	-2.21	XLOC_l2_013873		Downregulated
A_33_P338	0.000614	0.0000235	-2.22	COL5A1	collagen, type V, alpha 1	Downregulated
A_33_P325	0.000663	0.000028	-2.22	RNF7	ring finger protein 7	Downregulated
A_23_P218	0.000682	0.0000298	-2.22	ZGPAT	zinc finger, CCCH-type with G patch domain	Downregulated
A_23_P661	0.000701	0.0000328	-2.22	ITFG3	integrin alpha FG-GAP repeat containing 3	Downregulated
A_23_P759	0.000829	0.0000518	-2.22	CLPB	ClpB caseinolytic peptidase B homolog (E. coli)	Downregulated
A_33_P336	0.000856	0.0000556	-2.22	BOLA3	bolA family member 3	Downregulated
A_21_P001	0.001034	0.0000856	-2.22	XLOC_l2_004595		Downregulated
A_23_P297	0.00112	0.000103	-2.22	WWTR1	WW domain containing transcription regulator 1	Downregulated
A_33_P341	0.002006	0.000302	-2.22	AREG	amphiregulin	Downregulated
A_33_P335	0.002076	0.000321	-2.22	UBLCP1	ubiquitin-like domain containing CTD phosphatase 1	Downregulated
A_22_P000	0.003516	0.000722	-2.22	UBXN8	UBX domain protein 8	Downregulated
A_32_P676	0.000709	0.0000351	-2.23	FAM120C	family with sequence similarity 120C	Downregulated
A_33_P385	0.00277	0.000502	-2.23	CIRBP-AS1	CIRBP antisense RNA 1	Downregulated
A_33_P386	0.0044	0.00101	-2.23	lnc-FOXD4L6	lnc-FOXD4L6-3:1	Downregulated
A_33_P333	0.007276	0.00208	-2.23	MUC1	mucin 1, cell surface associated	Downregulated
A_22_P000	0.00895	0.00275	-2.23	lnc-CAPN13-1	lnc-CAPN13-1:1	Downregulated
A_23_P781	0.010163	0.00327	-2.23	MYO18A	myosin XVIIIA	Downregulated
A_23_P316	0.000596	0.0000211	-2.24	ACOX3	acyl-CoA oxidase 3, pristanoyl	Downregulated
A_24_P115	0.000603	0.0000221	-2.24	RIC8A	RIC8 guanine nucleotide exchange factor A	Downregulated
A_24_P230	0.000646	0.0000266	-2.24	ZNFX1	zinc finger, NFX1-type containing 1	Downregulated
A_24_P208	0.000783	0.0000449	-2.24	EDEM3	ER degradation enhancer, mannosidase alpha-like 3	Downregulated
A_23_P717	0.001147	0.000109	-2.24	TMEM206	transmembrane protein 206	Downregulated
A_33_P336	0.001171	0.000113	-2.24	FCRL3	Fc receptor-like 3	Downregulated
A_23_P115	0.001226	0.000124	-2.24	ECD	ecdysoneless homolog (Drosophila)	Downregulated
A_23_P686	0.001763	0.00024	-2.24	TMEM115	transmembrane protein 115	Downregulated
A_23_P130	0.001948	0.000285	-2.24	ZDHHC13	zinc finger, DHHC-type containing 13	Downregulated

A_23_P557	0.003453	0.000703	-2.24	CIC	capicua transcriptional repressor	Downregulated
A_21_P000	0.008039	0.00238	-2.24	lnc-ANO10-1	lnc-ANO10-1:1	Downregulated
A_23_P783	0.000702	0.000033	-2.25	C18orf8	chromosome 18 open reading frame 8	Downregulated
A_23_P309	0.000773	0.0000434	-2.25	HENMT1	HEN1 methyltransferase homolog 1 (Arabidopsis)	Downregulated
A_23_P368	0.000783	0.0000448	-2.25	GIMAP2	GTPase, IMAP family member 2	Downregulated
A_33_P333	0.000964	0.0000728	-2.25	FAM86FP	family with sequence similarity 86, member A pseudogene	Downregulated
A_33_P330	0.001032	0.0000854	-2.25	PLEKHG2	pleckstrin homology domain containing, family G (with RhoGef domain) r	Downregulated
A_23_P138	0.001137	0.000107	-2.25	ARL8A	ADP-ribosylation factor-like 8A	Downregulated
A_32_P220	0.004151	0.000921	-2.25	ZNF658	zinc finger protein 658	Downregulated
A_23_P952	0.005354	0.00134	-2.25	CASC1	cancer susceptibility candidate 1	Downregulated
A_23_P648	0.005582	0.00143	-2.25	OAS1	2'-5'-oligoadenylate synthetase 1, 40/46kDa	Downregulated
A_21_P000	0.007547	0.00219	-2.25	LOC1019271	uncharacterized LOC101927156	Downregulated
A_23_P366	0.008108	0.00241	-2.25	LATS1	large tumor suppressor kinase 1	Downregulated
A_23_P168	0.011241	0.00372	-2.25	WASF1	WAS protein family, member 1	Downregulated
A_33_P334	0.000611	0.0000232	-2.26	UQCRBP1	ubiquinol-cytochrome c reductase binding protein pseudogene 1	Downregulated
A_23_P137	0.000619	0.0000238	-2.26	MRPL24	mitochondrial ribosomal protein L24	Downregulated
A_23_P120	0.000746	0.0000402	-2.26	ORMDL1	ORMDL sphingolipid biosynthesis regulator 1	Downregulated
A_22_P000	0.000854	0.0000551	-2.26	lnc-GRHL2-1	lnc-GRHL2-1:1	Downregulated
A_23_P366	0.000911	0.0000633	-2.26	SCRN1	secernin 1	Downregulated
A_24_P388	0.001448	0.000169	-2.26	TPP1	tripeptidyl peptidase I	Downregulated
A_33_P366	0.00265	0.000469	-2.26	RHOT1	ras homolog family member T1	Downregulated
A_23_P250	0.005353	0.00134	-2.26	HACE1	HECT domain and ankyrin repeat containing E3 ubiquitin protein ligase	Downregulated
A_23_P355	0.000647	0.0000267	-2.27	EXOSC1	exosome component 1	Downregulated
A_23_P611	0.000669	0.0000286	-2.27	INPP5D	inositol polyphosphate-5-phosphatase, 145kDa	Downregulated
A_23_P856	0.000781	0.0000446	-2.27	GBP2	guanylate binding protein 2, interferon-inducible	Downregulated
A_33_P321	0.000866	0.0000571	-2.27	CREB3L2	cAMP responsive element binding protein 3-like 2	Downregulated
A_23_P137	0.001885	0.00027	-2.27	WRAP73	WD repeat containing, antisense to TP73	Downregulated
A_21_P001	0.002196	0.000353	-2.27	TXLNGY	taxilin gamma pseudogene, Y-linked	Downregulated
A_33_P371	0.002768	0.000502	-2.27	ARHGAP42	Rho GTPase activating protein 42	Downregulated
A_23_P148	0.003473	0.000709	-2.27	EHBP1L1	EH domain binding protein 1-like 1	Downregulated
A_21_P001	0.005906	0.00155	-2.27	FAM185A	family with sequence similarity 185, member A	Downregulated
A_21_P001	0.008618	0.00262	-2.27	XLOC_l2_006166		Downregulated
A_24_P202	0.014929	0.00538	-2.27	SIPA1L3	signal-induced proliferation-associated 1 like 3	Downregulated
A_23_P202	0.000596	0.0000211	-2.28	C11orf54	chromosome 11 open reading frame 54	Downregulated
A_21_P000	0.000654	0.0000273	-2.28	lnc-RPGRIP1-	lnc-RPGRIP1-2:1	Downregulated
A_23_P108	0.000788	0.0000456	-2.28	PPAP2C	phosphatidic acid phosphatase type 2C	Downregulated
A_33_P321	0.000795	0.0000469	-2.28	MED1	mediator complex subunit 1	Downregulated

A_24_P289	0.000901	0.0000614	-2.28	RNASET2	ribonuclease T2	Downregulated
A_33_P329	0.001134	0.000106	-2.28	CNTLN	centlein, centrosomal protein	Downregulated
A_23_P923	0.001134	0.000106	-2.28	TMEM156	transmembrane protein 156	Downregulated
A_33_P386	0.001405	0.00016	-2.28	MTMR10	myotubularin related protein 10	Downregulated
A_21_P000	0.001453	0.00017	-2.28	lnc-ABCA13-2	lnc-ABCA13-2:1	Downregulated
A_21_P001	0.003527	0.000725	-2.28	LOC1019301	uncharacterized LOC101930100	Downregulated
A_21_P000	0.0077	0.00225	-2.28	SNORD21	small nucleolar RNA, C/D box 21	Downregulated
A_24_P226	0.012265	0.00417	-2.28	TOX	thymocyte selection-associated high mobility group box	Downregulated
A_22_P000	0.000585	0.0000201	-2.29	lnc-GK-3	lnc-GK-3:1	Downregulated
A_33_P324	0.000594	0.0000208	-2.29	ZEB2	zinc finger E-box binding homeobox 2	Downregulated
A_21_P000	0.000612	0.0000233	-2.29	MKRN1	makorin ring finger protein 1	Downregulated
A_33_P322	0.000636	0.0000254	-2.29	MRPL11	mitochondrial ribosomal protein L11	Downregulated
A_22_P000	0.000691	0.0000305	-2.29	lnc-UBLCP1-7	lnc-UBLCP1-7:1	Downregulated
A_23_P387	0.000853	0.0000551	-2.29	SMCHD1	structural maintenance of chromosomes flexible hinge domain containin	Downregulated
A_23_P159	0.000938	0.0000672	-2.29	ADAM15	ADAM metallopeptidase domain 15	Downregulated
A_33_P333	0.000963	0.0000723	-2.29	TMEM59	transmembrane protein 59	Downregulated
A_21_P000	0.001322	0.000143	-2.29	LOC1001315	uncharacterized LOC100131564	Downregulated
A_24_P753	0.001641	0.000212	-2.29	BMPR2	bone morphogenetic protein receptor, type II (serine/threonine kinase)	Downregulated
A_22_P000	0.001769	0.000242	-2.29	LOC1019273	uncharacterized LOC101927365	Downregulated
A_22_P000	0.002965	0.000557	-2.29	lnc-AC112512	lnc-AC112512.1-1:1	Downregulated
A_23_P477	0.005063	0.00123	-2.29	METTL1	methyltransferase like 1	Downregulated
A_21_P000	0.005417	0.00137	-2.29	VTRNA2-1	vault RNA 2-1	Downregulated
A_24_P263	0.001066	0.0000924	-2.3	ATXN7L3B	ataxin 7-like 3B	Downregulated
A_24_P132	0.001237	0.000126	-2.3	IKBKB	inhibitor of kappa light polypeptide gene enhancer in B-cells, kinase bet	Downregulated
A_33_P328	0.001492	0.000179	-2.3	LMO7	LIM domain 7	Downregulated
A_23_P991	0.001775	0.000243	-2.3	UBE3B	ubiquitin protein ligase E3B	Downregulated
A_21_P001	0.006541	0.00179	-2.3	ANKRD26P3	ankyrin repeat domain 26 pseudogene 3	Downregulated
A_21_P000	0.007979	0.00236	-2.3	ZDHHC11	zinc finger, DHHC-type containing 11	Downregulated
A_23_P103	0.000707	0.0000343	-2.31	DTL	denticleless E3 ubiquitin protein ligase homolog (Drosophila)	Downregulated
A_33_P332	0.000797	0.0000471	-2.31	NDEL1	nudE neurodevelopment protein 1-like 1	Downregulated
A_23_P799	0.000855	0.0000555	-2.31	NOP56	NOP56 ribonucleoprotein	Downregulated
A_33_P321	0.000866	0.0000572	-2.31	RFX7	regulatory factor X, 7	Downregulated
A_23_P209	0.001394	0.000157	-2.31	PPM1G	protein phosphatase, Mg2+/Mn2+ dependent, 1G	Downregulated
A_33_P335	0.001949	0.000286	-2.31	FAM122C	family with sequence similarity 122C	Downregulated
A_33_P339	0.00308	0.000591	-2.31	GNG12	guanine nucleotide binding protein (G protein), gamma 12	Downregulated
A_23_P208	0.003529	0.000726	-2.31	LENG1	leukocyte receptor cluster (LRC) member 1	Downregulated
A_22_P000	0.005296	0.00132	-2.31	lnc-FITM2-1	lnc-FITM2-1:1	Downregulated

A_23_P280	0.00685	0.00191	-2.31	ZNF558	zinc finger protein 558	Downregulated
A_22_P000	0.007273	0.00207	-2.31	lnc-RTKN2-1	lnc-RTKN2-1:1	Downregulated
A_33_P323	0.014406	0.00514	-2.31	LOC1001285	uncharacterized LOC100128517	Downregulated
A_33_P342	0.000578	0.0000189	-2.32	TRAF3IP3	TRAF3 interacting protein 3	Downregulated
A_33_P337	0.00062	0.000024	-2.32	ABCC10	ATP-binding cassette, sub-family C (CFTR/MRP), member 10	Downregulated
A_23_P159	0.000693	0.0000309	-2.32	SSNA1	Sjogren syndrome nuclear autoantigen 1	Downregulated
A_23_P252	0.000848	0.0000542	-2.32	STK25	serine/threonine kinase 25	Downregulated
A_33_P341	0.000945	0.000069	-2.32	RABGAP1	RAB GTPase activating protein 1	Downregulated
A_23_P371	0.000963	0.0000725	-2.32	CDYL2	chromodomain protein, Y-like 2	Downregulated
A_24_P241	0.002145	0.00034	-2.32	ZC3H14	zinc finger CCCH-type containing 14	Downregulated
A_23_P214	0.002991	0.000565	-2.32	GPX5	glutathione peroxidase 5	Downregulated
A_24_P942	0.003041	0.00058	-2.32	ECT2L	epithelial cell transforming 2 like	Downregulated
A_21_P000	0.015034	0.00543	-2.32	LOC1009967	uncharacterized LOC100996741	Downregulated
A_33_P328	0.024503	0.01	-2.32	TRIM51HP	tripartite motif-containing 51H, pseudogene	Downregulated
A_23_P177	0.000578	0.0000192	-2.33	DPAGT1	dolichyl-phosphate (UDP-N-acetylglucosamine) N-acetylglucosaminepho	Downregulated
A_23_P120	0.000709	0.0000351	-2.33	LBH	limb bud and heart development	Downregulated
A_23_P201	0.000711	0.0000357	-2.33	MAPKAPK2	mitogen-activated protein kinase-activated protein kinase 2	Downregulated
A_33_P326	0.000855	0.0000555	-2.33	CD160	CD160 molecule	Downregulated
A_23_P114	0.001029	0.0000846	-2.33	HDAC1	histone deacetylase 1	Downregulated
A_23_P150	0.001214	0.000122	-2.33	MRE11A	MRE11 meiotic recombination 11 homolog A (S. cerevisiae)	Downregulated
A_23_P649	0.001267	0.000133	-2.33	RIC8B	RIC8 guanine nucleotide exchange factor B	Downregulated
A_32_P225	0.001403	0.000159	-2.33	FAM187B	family with sequence similarity 187, member B	Downregulated
A_24_P100	0.001557	0.000192	-2.33	CYB5R3	cytochrome b5 reductase 3	Downregulated
A_23_P129	0.005096	0.00125	-2.33	TARSL2	threonyl-tRNA synthetase-like 2	Downregulated
A_23_P102	0.025103	0.0103	-2.33	CHST10	carbohydrate sulfotransferase 10	Downregulated
A_33_P334	0.000578	0.0000194	-2.34	RAB28	RAB28, member RAS oncogene family	Downregulated
A_23_P124	0.00061	0.0000231	-2.34	ZCCHC7	zinc finger, CCHC domain containing 7	Downregulated
A_23_P143	0.00075	0.0000405	-2.34	ARID5A	AT rich interactive domain 5A (MRF1-like)	Downregulated
A_33_P337	0.000971	0.0000741	-2.34	MED25	mediator complex subunit 25	Downregulated
A_33_P340	0.001121	0.000104	-2.34	PLEKHM1	pleckstrin homology domain containing, family M (with RUN domain) mer	Downregulated
A_23_P226	0.001691	0.000223	-2.34	SLC9A6	solute carrier family 9, subfamily A (NHE6, cation proton antiporter 6), m	Downregulated
A_33_P325	0.000597	0.0000213	-2.35	CRIP1	cysteine-rich protein 1 (intestinal)	Downregulated
A_23_P396	0.000801	0.0000478	-2.35	NIN	ninein (GSK3B interacting protein)	Downregulated
A_24_P167	0.001386	0.000155	-2.35	VPS39	vacuolar protein sorting 39 homolog (S. cerevisiae)	Downregulated
A_33_P328	0.004021	0.000879	-2.35	TMEM254	transmembrane protein 254	Downregulated
A_23_P595	0.004433	0.00102	-2.35	ACN9	ACN9 homolog (S. cerevisiae)	Downregulated
A_21_P001	0.005135	0.00126	-2.35	XLOC_l2_003992		Downregulated

A_22_P000	0.007457	0.00215	-2.35	lnc-SNURF-3	lnc-SNURF-3:21	Downregulated
A_22_P000	0.007547	0.00219	-2.35	DIAPH2-AS1	DIAPH2 antisense RNA 1	Downregulated
A_33_P338	0.009895	0.00315	-2.35	FPGT	fucose-1-phosphate guanylyltransferase	Downregulated
A_21_P001	0.000603	0.0000224	-2.36	LOC1027248	uncharacterized LOC102724889	Downregulated
A_24_P787	0.001197	0.000118	-2.36	YPEL2	yippee-like 2 (Drosophila)	Downregulated
A_23_P142	0.001705	0.000228	-2.36	ZNF513	zinc finger protein 513	Downregulated
A_23_P433	0.002887	0.000535	-2.36	MLANA	melan-A	Downregulated
A_23_P831	0.00856	0.00259	-2.36	ABHD17B	abhydrolase domain containing 17B	Downregulated
A_33_P338	0.008606	0.00261	-2.36	RPPH1	ribonuclease P RNA component H1	Downregulated
A_21_P001	0.008661	0.00264	-2.36	TRANK1	tetratricopeptide repeat and ankyrin repeat containing 1	Downregulated
A_33_P337	0.000577	0.0000184	-2.37	TPPP	tubulin polymerization promoting protein	Downregulated
A_33_P339	0.000605	0.0000228	-2.37	SLC2A6	solute carrier family 2 (facilitated glucose transporter), member 6	Downregulated
A_32_P486	0.000788	0.0000456	-2.37	IGSF22	immunoglobulin superfamily, member 22	Downregulated
A_23_P883	0.000812	0.0000494	-2.37	EPHX2	epoxide hydrolase 2, cytoplasmic	Downregulated
A_23_P399	0.004499	0.00104	-2.37	ACTG2	actin, gamma 2, smooth muscle, enteric	Downregulated
A_24_P305	0.005211	0.00129	-2.37	GATAD2A	GATA zinc finger domain containing 2A	Downregulated
A_23_P418	0.008825	0.0027	-2.37	ATPAF2	ATP synthase mitochondrial F1 complex assembly factor 2	Downregulated
A_23_P212	0.011673	0.00391	-2.37	TBL1XR1	transducin (beta)-like 1 X-linked receptor 1	Downregulated
A_23_P140	0.00057	0.0000172	-2.38	IFI27L2	interferon, alpha-inducible protein 27-like 2	Downregulated
A_23_P202	0.000612	0.0000233	-2.38	APLP2	amyloid beta (A4) precursor-like protein 2	Downregulated
A_33_P328	0.000622	0.0000244	-2.38	METTL15	methyltransferase like 15	Downregulated
A_23_P166	0.000626	0.0000247	-2.38	RASSF2	Ras association (RalGDS/AF-6) domain family member 2	Downregulated
A_23_P118	0.001405	0.00016	-2.38	BIRC5	baculoviral IAP repeat containing 5	Downregulated
A_23_P393	0.002033	0.00031	-2.38	IGHMBP2	immunoglobulin mu binding protein 2	Downregulated
A_23_P388	0.002738	0.000493	-2.38	C4orf3	chromosome 4 open reading frame 3	Downregulated
A_33_P342	0.002897	0.000538	-2.38	PALLD	palladin, cytoskeletal associated protein	Downregulated
A_23_P974	0.003293	0.000657	-2.38	UBE2Q1	ubiquitin-conjugating enzyme E2Q family member 1	Downregulated
A_33_P333	0.003825	0.000818	-2.38	ALDH3A2	aldehyde dehydrogenase 3 family, member A2	Downregulated
A_23_P404	0.006699	0.00185	-2.38	CREBRF	CREB3 regulatory factor	Downregulated
A_23_P747	0.007611	0.00221	-2.38	EYA3	EYA transcriptional coactivator and phosphatase 3	Downregulated
A_33_P321	0.008008	0.00237	-2.38	C6orf89	chromosome 6 open reading frame 89	Downregulated
A_22_P000	0.015796	0.00578	-2.38	lnc-NUCB2-1	lnc-NUCB2-1:1	Downregulated
A_23_P123	0.000605	0.0000228	-2.39	GLDC	glycine dehydrogenase (decarboxylating)	Downregulated
A_24_P182	0.001125	0.000104	-2.39	CELSR2	cadherin, EGF LAG seven-pass G-type receptor 2	Downregulated
A_21_P000	0.001202	0.000119	-2.39	FAM174B	family with sequence similarity 174, member B	Downregulated
A_24_P253	0.002901	0.000539	-2.39	DBF4B	DBF4 zinc finger B	Downregulated
A_22_P000	0.009588	0.00302	-2.39	lnc-CLCN6-1	lnc-CLCN6-1:2	Downregulated

A_32_P151	0.000577	0.0000179	-2.4	FAM72D	family with sequence similarity 72, member D	Downregulated
A_21_P000	0.000673	0.0000291	-2.4	SNORD82	small nucleolar RNA, C/D box 82	Downregulated
A_22_P000	0.000706	0.0000334	-2.4	lnc-MAP3K12	lnc-MAP3K12-1:1	Downregulated
A_22_P000	0.000742	0.0000396	-2.4	lnc-GLIPR1-3	lnc-GLIPR1-3:1	Downregulated
A_23_P121	0.000991	0.0000773	-2.4	TSHB	thyroid stimulating hormone, beta	Downregulated
A_23_P371	0.001029	0.0000846	-2.4	ZNF227	zinc finger protein 227	Downregulated
A_22_P000	0.001295	0.000137	-2.4	LOC1005068	uncharacterized LOC100506844	Downregulated
A_23_P157	0.005544	0.00141	-2.4	INVS	inversin	Downregulated
A_33_P328	0.005673	0.00146	-2.4	CTNNBIP1	catenin, beta interacting protein 1	Downregulated
A_19_P003	0.008172	0.00244	-2.4	LOC1010599	uncharacterized LOC101059954	Downregulated
A_23_P110	0.009995	0.00319	-2.4	APBB3	amyloid beta (A4) precursor protein-binding, family B, member 3	Downregulated
A_21_P000	0.00056	0.0000165	-2.41	BZRAP1-AS1	BZRAP1 antisense RNA 1	Downregulated
A_23_P106	0.000618	0.0000237	-2.41	FOS	FBJ murine osteosarcoma viral oncogene homolog	Downregulated
A_23_P659	0.00062	0.0000241	-2.41	ZFYVE19	zinc finger, FYVE domain containing 19	Downregulated
A_19_P003	0.00064	0.0000259	-2.41	TSTD3	thiosulfate sulfurtransferase (rhodanese)-like domain containing 3	Downregulated
A_23_P148	0.000669	0.0000285	-2.41	GNPTG	N-acetylglucosamine-1-phosphate transferase, gamma subunit	Downregulated
A_23_P818	0.000695	0.0000316	-2.41	TJAP1	tight junction associated protein 1 (peripheral)	Downregulated
A_24_P134	0.000696	0.0000318	-2.41	RHBDD1	rhomboid domain containing 1	Downregulated
A_23_P500	0.001015	0.0000824	-2.41	KANK1	KN motif and ankyrin repeat domains 1	Downregulated
A_23_P742	0.001268	0.000133	-2.41	GBP5	guanylate binding protein 5	Downregulated
A_24_P227	0.00189	0.000271	-2.41	KLC2	kinesin light chain 2	Downregulated
A_22_P000	0.004328	0.000984	-2.41	lnc-CDNF-1	lnc-CDNF-1:1	Downregulated
A_21_P000	0.000596	0.0000211	-2.42	lnc-APBA2-2	lnc-APBA2-2:1	Downregulated
A_33_P322	0.000603	0.0000224	-2.42	SLC12A9	solute carrier family 12, member 9	Downregulated
A_23_P117	0.000639	0.0000256	-2.42	WASH1	WAS protein family homolog 1	Downregulated
A_23_P388	0.000695	0.0000312	-2.42	ELAVL1	ELAV like RNA binding protein 1	Downregulated
A_33_P336	0.000792	0.0000463	-2.42	MPP2	membrane protein, palmitoylated 2 (MAGUK p55 subfamily member 2)	Downregulated
A_23_P106	0.000801	0.0000478	-2.42	RAB11FIP3	RAB11 family interacting protein 3 (class II)	Downregulated
A_23_P283	0.001065	0.0000918	-2.42	NDUFAF7	NADH dehydrogenase (ubiquinone) complex I, assembly factor 7	Downregulated
A_33_P326	0.002006	0.000302	-2.42	UVRAG	UV radiation resistance associated	Downregulated
A_23_P370	0.00238	0.000398	-2.42	C12orf66	chromosome 12 open reading frame 66	Downregulated
A_23_P893	0.016218	0.00599	-2.42	ZNF286A	zinc finger protein 286A	Downregulated
A_33_P323	0.000568	0.0000172	-2.43	DGKZ	diacylglycerol kinase, zeta	Downregulated
A_22_P000	0.000578	0.0000193	-2.43	FGF12-AS1	FGF12 antisense RNA 1	Downregulated
A_33_P381	0.000647	0.0000268	-2.43	GABARAPL1	GABA(A) receptor-associated protein like 1	Downregulated
A_33_P339	0.001062	0.0000913	-2.43	TP53RK	TP53 regulating kinase	Downregulated
A_23_P139	0.001258	0.000131	-2.43	GNG3	guanine nucleotide binding protein (G protein), gamma 3	Downregulated

A_33_P334	0.002219	0.000358	-2.43	CPTP	ceramide-1-phosphate transfer protein	Downregulated
A_24_P132	0.003557	0.000735	-2.43	NAP1L4	nucleosome assembly protein 1-like 4	Downregulated
A_22_P000	0.004306	0.000975	-2.43	THAP9-AS1	THAP9 antisense RNA 1	Downregulated
A_24_P509	0.006045	0.0016	-2.43	GOLGA6L6	golgin A6 family-like 6	Downregulated
A_23_P360	0.00899	0.00277	-2.43	CPNE5	copine V	Downregulated
A_24_P185	0.009601	0.00302	-2.43	FAM134C	family with sequence similarity 134, member C	Downregulated
A_23_P217	0.012702	0.00435	-2.43	TIMM8A	translocase of inner mitochondrial membrane 8 homolog A (yeast)	Downregulated
A_22_P000	0.000602	0.0000219	-2.44	lnc-CCNE2-1	lnc-CCNE2-1:3	Downregulated
A_23_P126	0.000605	0.0000227	-2.44	EIF4G3	eukaryotic translation initiation factor 4 gamma, 3	Downregulated
A_24_P502	0.00067	0.0000288	-2.44	HLA-DMA	major histocompatibility complex, class II, DM alpha	Downregulated
A_21_P000	0.000793	0.0000466	-2.44	lnc-CCDC140	lnc-CCDC140-2:1	Downregulated
A_23_P502	0.000871	0.0000578	-2.44	FYN	FYN proto-oncogene, Src family tyrosine kinase	Downregulated
A_22_P000	0.000883	0.0000591	-2.44	LOC1009965	uncharacterized LOC100996579	Downregulated
A_23_P705	0.001062	0.0000907	-2.44	SLC39A7	solute carrier family 39 (zinc transporter), member 7	Downregulated
A_22_P000	0.001714	0.000229	-2.44	lnc-APOC3-6	lnc-APOC3-6:1	Downregulated
A_24_P544	0.007028	0.00198	-2.44	SSR1	signal sequence receptor, alpha	Downregulated
A_33_P324	0.007298	0.00208	-2.44	FAM72A	family with sequence similarity 72, member A	Downregulated
A_33_P325	0.000565	0.000017	-2.45	N6AMT1	N-6 adenine-specific DNA methyltransferase 1 (putative)	Downregulated
A_33_P346	0.000819	0.0000505	-2.45	lnc-NMNAT2-2	lnc-NMNAT2-2:1	Downregulated
A_21_P000	0.000995	0.0000786	-2.45	SNORD32A	small nucleolar RNA, C/D box 32A	Downregulated
A_21_P000	0.001057	0.0000901	-2.45	lnc-KLHL25-9	lnc-KLHL25-9:1	Downregulated
A_24_P706	0.001455	0.000171	-2.45	HNRNPA3	heterogeneous nuclear ribonucleoprotein A3	Downregulated
A_23_P132	0.001504	0.000181	-2.45	MPST	mercaptopyruvate sulfurtransferase	Downregulated
A_23_P411	0.002951	0.000553	-2.45	WDR90	WD repeat domain 90	Downregulated
A_23_P132	0.000568	0.0000172	-2.46	MSL2	male-specific lethal 2 homolog (Drosophila)	Downregulated
A_33_P323	0.000577	0.0000181	-2.46	BOLA2B	bolA family member 2B	Downregulated
A_23_P329	0.000821	0.0000509	-2.46	JAK3	Janus kinase 3	Downregulated
A_33_P333	0.000905	0.0000622	-2.46	DTNBP1	dystrobrevin binding protein 1	Downregulated
A_33_P328	0.000995	0.0000789	-2.46	TMEM254	transmembrane protein 254	Downregulated
A_23_P121	0.001528	0.000186	-2.46	CSRNP1	cysteine-serine-rich nuclear protein 1	Downregulated
A_22_P000	0.001676	0.000219	-2.46	lnc-SCAPER-	lnc-SCAPER-1:1	Downregulated
A_24_P628	0.001937	0.000283	-2.46	STAM2	signal transducing adaptor molecule (SH3 domain and ITAM motif) 2	Downregulated
A_23_P200	0.001943	0.000284	-2.46	TXNDC12	thioredoxin domain containing 12 (endoplasmic reticulum)	Downregulated
A_33_P354	0.001956	0.000289	-2.46	IL17RA	interleukin 17 receptor A	Downregulated
A_23_P312	0.002009	0.000303	-2.46	ALMS1	Alstrom syndrome 1	Downregulated
A_22_P000	0.003779	0.000803	-2.46	lnc-GGH-1	lnc-GGH-1:1	Downregulated
A_23_P148	0.005795	0.0015	-2.46	ZDHHC9	zinc finger, DHHC-type containing 9	Downregulated

A_23_P869	0.010923	0.00357	-2.46	B4GAT1	beta-1,4-glucuronyltransferase 1	Downregulated
A_33_P327	0.000805	0.0000484	-2.47	ARFGAP2	ADP-ribosylation factor GTPase activating protein 2	Downregulated
A_23_P415	0.001899	0.000273	-2.47	KLF9	Kruppel-like factor 9	Downregulated
A_22_P000	0.003936	0.000854	-2.47	lnc-SMG1-2	lnc-SMG1-2:1	Downregulated
A_33_P322	0.004843	0.00116	-2.47	NPHP3	nephronophthisis 3 (adolescent)	Downregulated
A_23_P121	0.007132	0.00202	-2.47	TBCCD1	TBCC domain containing 1	Downregulated
A_21_P001	0.000648	0.0000268	-2.48	NNT-AS1	NNT antisense RNA 1	Downregulated
A_23_P105	0.000689	0.0000303	-2.48	FRY	furry homolog (Drosophila)	Downregulated
A_22_P000	0.000766	0.0000428	-2.48	DCP1A	decapping mRNA 1A	Downregulated
A_23_P121	0.002427	0.00041	-2.48	CYB561D2	cytochrome b561 family, member D2	Downregulated
A_23_P210	0.003191	0.000627	-2.48	RTFDC1	replication termination factor 2 domain containing 1	Downregulated
A_23_P749	0.003966	0.000863	-2.48	MR1	major histocompatibility complex, class I-related	Downregulated
A_23_P202	0.008622	0.00262	-2.48	MCMBP	minichromosome maintenance complex binding protein	Downregulated
A_23_P107	0.010485	0.0034	-2.48	TMEM11	transmembrane protein 11	Downregulated
A_23_P257	0.000603	0.000022	-2.49	SRA1	steroid receptor RNA activator 1	Downregulated
A_23_P442	0.001054	0.0000893	-2.49	CLASP2	cytoplasmic linker associated protein 2	Downregulated
A_33_P330	0.001194	0.000117	-2.49	METTL15	methyltransferase like 15	Downregulated
A_22_P000	0.001263	0.000132	-2.49	PHKA2-AS1	PHKA2 antisense RNA 1	Downregulated
A_23_P236	0.001492	0.000179	-2.49	USF1	upstream transcription factor 1	Downregulated
A_22_P000	0.001839	0.000259	-2.49	LOC1019270	uncharacterized LOC101927027	Downregulated
A_19_P003	0.000537	0.0000141	-2.5	ZNF678	zinc finger protein 678	Downregulated
A_33_P335	0.000571	0.0000173	-2.5	SOS1	son of sevenless homolog 1 (Drosophila)	Downregulated
A_24_P945	0.00062	0.0000241	-2.5	SKA2	spindle and kinetochore associated complex subunit 2	Downregulated
A_23_P201	0.000695	0.0000317	-2.5	S100A6	S100 calcium binding protein A6	Downregulated
A_19_P003	0.000803	0.0000481	-2.5	lnc-SPAG1-3	lnc-SPAG1-3:3	Downregulated
A_21_P000	0.000964	0.0000729	-2.5	SNORD32B	small nucleolar RNA, C/D box 32B	Downregulated
A_23_P847	0.002688	0.00048	-2.5	TNFRSF13B	tumor necrosis factor receptor superfamily, member 13B	Downregulated
A_33_P333	0.006915	0.00194	-2.5	SDCCAG3	serologically defined colon cancer antigen 3	Downregulated
A_33_P330	0.007099	0.00201	-2.5	EID1	EP300 interacting inhibitor of differentiation 1	Downregulated
A_21_P001	0.009497	0.00298	-2.5	LOC1019285	uncharacterized LOC101928595	Downregulated
A_33_P387	0.000577	0.0000186	-2.51	TRIM39-RPP2	TRIM39-RPP21 readthrough	Downregulated
A_32_P466	0.000601	0.0000217	-2.51	IRF2BPL	interferon regulatory factor 2 binding protein-like	Downregulated
A_33_P327	0.000724	0.0000375	-2.51	TARDBP	TAR DNA binding protein	Downregulated
A_22_P000	0.002018	0.000305	-2.51	RNASEH2B-A	RNASEH2B antisense RNA 1	Downregulated
A_23_P216	0.004355	0.000994	-2.51	SLA	Src-like-adaptor	Downregulated
A_23_P163	0.005444	0.00138	-2.51	RBM4B	RNA binding motif protein 4B	Downregulated
A_24_P392	0.009995	0.00319	-2.51	PSG8	pregnancy specific beta-1-glycoprotein 8	Downregulated

A_23_P368	0.000537	0.0000139	-2.52	SNX12	sorting nexin 12	Downregulated
A_23_P411	0.000763	0.0000419	-2.52	CEBPB	CCAAT/enhancer binding protein (C/EBP), beta	Downregulated
A_24_P116	0.000925	0.0000653	-2.52	GCOM1	GRINL1A complex locus 1	Downregulated
A_22_P000	0.001014	0.0000822	-2.52	PAQR9-AS1	PAQR9 antisense RNA 1	Downregulated
A_23_P301	0.001254	0.00013	-2.52	IP6K2	inositol hexakisphosphate kinase 2	Downregulated
A_23_P106	0.001349	0.000148	-2.52	GALNS	galactosamine (N-acetyl)-6-sulfatase	Downregulated
A_23_P118	0.002323	0.000384	-2.52	BCL7C	B-cell CLL/lymphoma 7C	Downregulated
A_23_P769	0.003074	0.000589	-2.52	SIX1	SIX homeobox 1	Downregulated
A_23_P125	0.000577	0.0000183	-2.53	IRF2	interferon regulatory factor 2	Downregulated
A_33_P325	0.000707	0.0000341	-2.53	VIMP	VCP-interacting membrane protein	Downregulated
A_32_P822	0.001349	0.000148	-2.53	GRM8	glutamate receptor, metabotropic 8	Downregulated
A_24_P136	0.001918	0.000278	-2.53	CA5BP1	carbonic anhydrase VB pseudogene 1	Downregulated
A_33_P335	0.003068	0.000588	-2.53	TSC1	tuberous sclerosis 1	Downregulated
A_24_P680	0.008254	0.00247	-2.53	CCDC73	coiled-coil domain containing 73	Downregulated
A_23_P293	0.000808	0.0000489	-2.54	SAMM50	SAMM50 sorting and assembly machinery component	Downregulated
A_33_P324	0.000887	0.0000597	-2.54	LOC1001298	uncharacterized LOC100129888	Downregulated
A_33_P327	0.002237	0.000363	-2.54	YIPF2	Yip1 domain family, member 2	Downregulated
A_21_P000	0.003986	0.000869	-2.54	lnc-SRSF2-1	lnc-SRSF2-1:1	Downregulated
A_22_P000	0.008623	0.00262	-2.54	LOC1019291	uncharacterized LOC101929162	Downregulated
A_23_P141	0.000534	0.0000136	-2.55	GPR18	G protein-coupled receptor 18	Downregulated
A_23_P338	0.000719	0.0000369	-2.55	KDM1B	lysine (K)-specific demethylase 1B	Downregulated
A_23_P218	0.000863	0.0000566	-2.55	B9D2	B9 protein domain 2	Downregulated
A_23_P390	0.000938	0.000067	-2.55	SMARCA4	SWI/SNF related, matrix associated, actin dependent regulator of chrom	Downregulated
A_33_P338	0.00152	0.000184	-2.55	ERCC5	excision repair cross-complementation group 5	Downregulated
A_33_P337	0.001727	0.000232	-2.55	FAM117B	family with sequence similarity 117, member B	Downregulated
A_23_P466	0.001919	0.000278	-2.55	LPGAT1	lysophosphatidylglycerol acyltransferase 1	Downregulated
A_23_P164	0.002113	0.00033	-2.55	WDR7	WD repeat domain 7	Downregulated
A_23_P360	0.003287	0.000655	-2.55	NUDT7	nudix (nucleoside diphosphate linked moiety X)-type motif 7	Downregulated
A_23_P220	0.000713	0.0000361	-2.56	TAOK3	TAO kinase 3	Downregulated
A_23_P128	0.000715	0.0000363	-2.56	FBXO18	F-box protein, helicase, 18	Downregulated
A_21_P000	0.000727	0.0000378	-2.56	RAB30-AS1	RAB30 antisense RNA 1 (head to head)	Downregulated
A_33_P329	0.000728	0.0000381	-2.56	COPS7B	COP9 signalosome subunit 7B	Downregulated
A_23_P852	0.000791	0.0000461	-2.56	TTN	titin	Downregulated
A_23_P164	0.001635	0.00021	-2.56	RCE1	Ras converting CAAX endopeptidase 1	Downregulated
A_23_P845	0.001799	0.000248	-2.56	MZB1	marginal zone B and B1 cell-specific protein	Downregulated
A_33_P324	0.002101	0.000327	-2.56	IKBKB	inhibitor of kappa light polypeptide gene enhancer in B-cells, kinase bet	Downregulated
A_33_P337	0.003804	0.000812	-2.56	DDX51	DEAD (Asp-Glu-Ala-Asp) box polypeptide 51	Downregulated

A_23_P759	0.006531	0.00179	-2.56	RNF121	ring finger protein 121	Downregulated
A_24_P601	0.008888	0.00273	-2.56	C2orf74	chromosome 2 open reading frame 74	Downregulated
A_21_P000	0.000578	0.0000188	-2.57	lnc-DDX51-4	lnc-DDX51-4:1	Downregulated
A_23_P141	0.000578	0.0000194	-2.57	SERTAD3	SERTA domain containing 3	Downregulated
A_23_P146	0.000739	0.0000392	-2.57	ASAP1-IT1	ASAP1 intronic transcript 1 (non-protein coding)	Downregulated
A_23_P134	0.000784	0.0000451	-2.57	NUDT1	nudix (nucleoside diphosphate linked moiety X)-type motif 1	Downregulated
A_21_P000	0.000794	0.0000467	-2.57	SNORA14B	small nucleolar RNA, H/ACA box 14B	Downregulated
A_21_P001	0.000905	0.0000622	-2.57	LOC1019284	uncharacterized LOC101928461	Downregulated
A_22_P000	0.001047	0.0000881	-2.57	SVIL-AS1	SVIL antisense RNA 1	Downregulated
A_23_P682	0.001062	0.0000909	-2.57	TTN	titin	Downregulated
A_22_P000	0.001697	0.000225	-2.57	lnc-USP50-1	lnc-USP50-1:1	Downregulated
A_33_P325	0.001914	0.000277	-2.57	AIDA	axin interactor, dorsalization associated	Downregulated
A_22_P000	0.002351	0.000391	-2.57	NEURL4	neuralized E3 ubiquitin protein ligase 4	Downregulated
A_23_P152	0.003586	0.000746	-2.57	E2F4	E2F transcription factor 4, p107/p130-binding	Downregulated
A_23_P287	0.000577	0.0000181	-2.58	DBNDD2	dysbindin (dystrobrevin binding protein 1) domain containing 2	Downregulated
A_23_P141	0.000707	0.0000344	-2.58	RDM1	RAD52 motif containing 1	Downregulated
A_23_P398	0.00098	0.0000758	-2.58	TTC31	tetratricopeptide repeat domain 31	Downregulated
A_23_P101	0.001018	0.0000827	-2.58	ZFP36L2	ZFP36 ring finger protein-like 2	Downregulated
A_22_P000	0.004602	0.00108	-2.58	lnc-SP140-2	lnc-SP140-2:1	Downregulated
A_23_P162	0.00536	0.00135	-2.58	WNT10B	wingless-type MMTV integration site family, member 10B	Downregulated
A_24_P192	0.001952	0.000287	-2.59	MLLT3	myeloid/lymphoid or mixed-lineage leukemia (trithorax homolog, Drosopł	Downregulated
A_33_P322	0.001964	0.000291	-2.59	lnc-DLX2-4	lnc-DLX2-4:3	Downregulated
A_33_P339	0.00206	0.000317	-2.59	MRPL4	mitochondrial ribosomal protein L4	Downregulated
A_21_P000	0.002163	0.000344	-2.59	lnc-BOD1-1	lnc-BOD1-1:7	Downregulated
A_33_P330	0.002901	0.000539	-2.59	ALOX12-AS1	ALOX12 antisense RNA 1	Downregulated
A_23_P416	0.003667	0.000769	-2.59	PIF1	PIF1 5'-to-3' DNA helicase	Downregulated
A_33_P334	0.010001	0.00319	-2.59	HIP1	huntingtin interacting protein 1	Downregulated
A_23_P347	0.011519	0.00384	-2.59	MTSS1	metastasis suppressor 1	Downregulated
A_22_P000	0.001257	0.00013	-2.6	lnc-SLC25A1!	lnc-SLC25A19-1:1	Downregulated
A_21_P000	0.00141	0.000161	-2.6	LOC1001315	uncharacterized LOC100131564	Downregulated
A_21_P000	0.00165	0.000213	-2.6	lnc-DLEU1-1	lnc-DLEU1-1:1	Downregulated
A_33_P336	0.002968	0.000559	-2.6	DNAJC15	DnaJ (Hsp40) homolog, subfamily C, member 15	Downregulated
A_24_P166	0.008188	0.00244	-2.6	IMPAD1	inositol monophosphatase domain containing 1	Downregulated
A_23_P446	0.000526	0.0000124	-2.61	CRIP1	cysteine-rich protein 1 (intestinal)	Downregulated
A_21_P001	0.00055	0.0000151	-2.61	XLOC_l2_001066		Downregulated
A_23_P149	0.000575	0.0000176	-2.61	PDLIM1	PDZ and LIM domain 1	Downregulated
A_23_P202	0.000805	0.0000482	-2.61	COMMD5	COMM domain containing 5	Downregulated

A_33_P327	0.000954	0.0000704	-2.61	NADK	NAD kinase	Downregulated
A_33_P328	0.001397	0.000157	-2.61	SSX2IP	synovial sarcoma, X breakpoint 2 interacting protein	Downregulated
A_21_P000	0.002062	0.000318	-2.61	TSFM	Ts translation elongation factor, mitochondrial	Downregulated
A_23_P145	0.002081	0.000322	-2.61	GPR65	G protein-coupled receptor 65	Downregulated
A_23_P302	0.002571	0.000449	-2.61	PCYOX1L	prenylcysteine oxidase 1 like	Downregulated
A_23_P565	0.003543	0.000731	-2.61	ITGA4	integrin, alpha 4 (antigen CD49D, alpha 4 subunit of VLA-4 receptor)	Downregulated
A_24_P379	0.0038	0.00081	-2.61	RPS14	ribosomal protein S14	Downregulated
A_33_P367	0.003815	0.000815	-2.61	USP28	ubiquitin specific peptidase 28	Downregulated
A_23_P115	0.000597	0.0000214	-2.62	VPS72	vacuolar protein sorting 72 homolog (S. cerevisiae)	Downregulated
A_19_P008	0.000792	0.0000463	-2.62	LOC1019293	uncharacterized LOC101929378	Downregulated
A_21_P000	0.00124	0.000127	-2.62	lnc-PCF11-1	lnc-PCF11-1:12	Downregulated
A_23_P155	0.002693	0.000481	-2.62	CYTH4	cytohesin 4	Downregulated
A_21_P001	0.006965	0.00196	-2.62	FTX	FTX transcript, XIST regulator (non-protein coding)	Downregulated
A_24_P203	0.00067	0.0000288	-2.63	IL2RB	interleukin 2 receptor, beta	Downregulated
A_21_P000	0.00071	0.0000354	-2.63	SNORD20	small nucleolar RNA, C/D box 20	Downregulated
A_33_P324	0.000719	0.0000369	-2.63	CTSD	cathepsin D	Downregulated
A_24_P371	0.000819	0.0000505	-2.63	SLC35G2	solute carrier family 35, member G2	Downregulated
A_21_P000	0.000959	0.0000714	-2.63	lnc-VEGFC-1	lnc-VEGFC-1:1	Downregulated
A_23_P902	0.000959	0.0000714	-2.63	ZNF585B	zinc finger protein 585B	Downregulated
A_33_P376	0.001129	0.000105	-2.63	PIGU	phosphatidylinositol glycan anchor biosynthesis, class U	Downregulated
A_22_P000	0.00773	0.00226	-2.63	lnc-S1PR1-1	lnc-S1PR1-1:1	Downregulated
A_21_P000	0.011169	0.00368	-2.63	SNORD26	small nucleolar RNA, C/D box 26	Downregulated
A_23_P135	0.000526	0.0000128	-2.64	PLAA	phospholipase A2-activating protein	Downregulated
A_24_P231	0.00056	0.000016	-2.64	DNPEP	aspartyl aminopeptidase	Downregulated
A_23_P102	0.000707	0.0000342	-2.64	BANK1	B-cell scaffold protein with ankyrin repeats 1	Downregulated
A_33_P324	0.000928	0.0000657	-2.64	CNOT6L	CCR4-NOT transcription complex, subunit 6-like	Downregulated
A_24_P159	0.000998	0.0000798	-2.64	PAK6	p21 protein (Cdc42/Rac)-activated kinase 6	Downregulated
A_23_P976	0.001008	0.0000813	-2.64	CNTROB	centrobin, centrosomal BRCA2 interacting protein	Downregulated
A_23_P154	0.001171	0.000113	-2.64	BACE2	beta-site APP-cleaving enzyme 2	Downregulated
A_22_P000	0.001266	0.000133	-2.64	lnc-NSUN6-1	lnc-NSUN6-1:1	Downregulated
A_23_P596	0.002101	0.000327	-2.64	NPVF	neuropeptide VF precursor	Downregulated
A_33_P321	0.002276	0.000373	-2.64	AAK1	AP2 associated kinase 1	Downregulated
A_23_P358	0.00412	0.000911	-2.64	PRIMPOL	primase and polymerase (DNA-directed)	Downregulated
A_21_P000	0.007766	0.00227	-2.64	lnc-HMG20A-5	lnc-HMG20A-5:1	Downregulated
A_33_P322	0.00067	0.0000289	-2.65	MTMR10	myotubularin related protein 10	Downregulated
A_22_P000	0.000765	0.0000424	-2.65	lnc-ZC3H12D-	lnc-ZC3H12D-2:1	Downregulated
A_33_P322	0.000866	0.0000571	-2.65	KIF21B	kinesin family member 21B	Downregulated

A_33_P329	0.000942	0.0000684	-2.65	UBIAD1	UbiA prenyltransferase domain containing 1	Downregulated
A_24_P942	0.001163	0.000112	-2.65	TMX4	thioredoxin-related transmembrane protein 4	Downregulated
A_22_P000	0.002572	0.000449	-2.65	IFNG-AS1	IFNG antisense RNA 1	Downregulated
A_23_P577	0.006659	0.00184	-2.65	DRD3	dopamine receptor D3	Downregulated
A_33_P324	0.000563	0.0000168	-2.66	NSUN4	NOP2/Sun domain family, member 4	Downregulated
A_23_P122	0.00065	0.000027	-2.66	CCNB1	cyclin B1	Downregulated
A_23_P153	0.000673	0.000029	-2.66	XRCC1	X-ray repair complementing defective repair in Chinese hamster cells 1	Downregulated
A_33_P323	0.000707	0.0000336	-2.66	KIAA1217	KIAA1217	Downregulated
A_23_P147	0.000721	0.0000373	-2.66	KIF13B	kinesin family member 13B	Downregulated
A_33_P323	0.001066	0.0000923	-2.66	MTMR3	myotubularin related protein 3	Downregulated
A_22_P000	0.00129	0.000136	-2.66	lnc-DPCD-1	lnc-DPCD-1:1	Downregulated
A_33_P321	0.001692	0.000223	-2.66	RNASEH2B	ribonuclease H2, subunit B	Downregulated
A_23_P354	0.002909	0.000541	-2.66	MYOF	myoferlin	Downregulated
A_33_P331	0.006257	0.00168	-2.66	FOXK2	forkhead box K2	Downregulated
A_23_P119	0.013974	0.00492	-2.66	GUCA2A	guanylate cyclase activator 2A (guanylin)	Downregulated
A_33_P337	0.000526	0.0000119	-2.67	CYBA	cytochrome b-245, alpha polypeptide	Downregulated
A_24_P273	0.00056	0.0000163	-2.67	MED20	mediator complex subunit 20	Downregulated
A_33_P330	0.000578	0.000019	-2.67	COL19A1	collagen, type XIX, alpha 1	Downregulated
A_33_P340	0.000906	0.0000624	-2.67	PECAM1	platelet/endothelial cell adhesion molecule 1	Downregulated
A_23_P121	0.000925	0.0000653	-2.67	PARP8	poly (ADP-ribose) polymerase family, member 8	Downregulated
A_23_P141	0.001103	0.0000999	-2.67	TPGS2	tubulin polyglutamylase complex subunit 2	Downregulated
A_21_P001	0.001603	0.000202	-2.67	CD99P1	CD99 molecule pseudogene 1	Downregulated
A_33_P328	0.002153	0.000342	-2.67	CTSB	cathepsin B	Downregulated
A_21_P000	0.003674	0.000771	-2.67	SNORD54	small nucleolar RNA, C/D box 54	Downregulated
A_23_P554	0.00478	0.00114	-2.67	CBX8	chromobox homolog 8	Downregulated
A_24_P348	0.000711	0.0000357	-2.68	TTTY15	testis-specific transcript, Y-linked 15 (non-protein coding)	Downregulated
A_32_P110	0.000831	0.000052	-2.68	GGACT	gamma-glutamylamine cyclotransferase	Downregulated
A_33_P326	0.001563	0.000194	-2.68	IVD	isovaleryl-CoA dehydrogenase	Downregulated
A_33_P331	0.003893	0.000841	-2.68	LINC01184	long intergenic non-protein coding RNA 1184	Downregulated
A_21_P000	0.009843	0.00313	-2.68	lnc-C1orf124-	lnc-C1orf124-1:1	Downregulated
A_33_P325	0.000525	0.0000111	-2.69	BCL2L11	BCL2-like 11 (apoptosis facilitator)	Downregulated
A_32_P298	0.000532	0.0000132	-2.69	CRADD	CASP2 and RIPK1 domain containing adaptor with death domain	Downregulated
A_23_P611	0.000597	0.0000213	-2.69	APOO	apolipoprotein O	Downregulated
A_23_P128	0.000773	0.0000434	-2.69	GZMH	granzyme H (cathepsin G-like 2, protein h-CCPX)	Downregulated
A_24_P383	0.00093	0.0000661	-2.69	DPYSL2	dihydropyrimidinase-like 2	Downregulated
A_23_P611	0.002627	0.000464	-2.69	PLCXD1	phosphatidylinositol-specific phospholipase C, X domain containing 1	Downregulated
A_33_P338	0.000709	0.0000351	-2.7	TBPL1	TBP-like 1	Downregulated

A_23_P610	0.001427	0.000164	-2.7	MLKL	mixed lineage kinase domain-like	Downregulated
A_33_P339	0.001445	0.000168	-2.7	PARP10	poly (ADP-ribose) polymerase family, member 10	Downregulated
A_24_P544	0.001537	0.000188	-2.7	CCDC115	coiled-coil domain containing 115	Downregulated
A_23_P949	0.001695	0.000224	-2.7	ZNF283	zinc finger protein 283	Downregulated
A_23_P193	0.00248	0.000424	-2.7	CUL7	cullin 7	Downregulated
A_24_P152	0.003568	0.00074	-2.7	C10orf76	chromosome 10 open reading frame 76	Downregulated
A_22_P000	0.00056	0.0000162	-2.71	lnc-RWDD2B-	lnc-RWDD2B-1:1	Downregulated
A_24_P354	0.000759	0.0000415	-2.71	MTMR12	myotubularin related protein 12	Downregulated
A_23_P329	0.000603	0.0000224	-2.72	VPS4A	vacuolar protein sorting 4 homolog A (S. cerevisiae)	Downregulated
A_22_P000	0.000766	0.0000427	-2.72	lnc-ZNF8-1	lnc-ZNF8-1:1	Downregulated
A_22_P000	0.000776	0.0000438	-2.72	lnc-CDIPT-1	lnc-CDIPT-1:7	Downregulated
A_23_P682	0.000995	0.000079	-2.72	GPR75	G protein-coupled receptor 75	Downregulated
A_21_P000	0.001804	0.000249	-2.72	lnc-NDFIP2-8	lnc-NDFIP2-8:1	Downregulated
A_23_P216	0.000525	0.0000115	-2.73	ENTPD4	ectonucleoside triphosphate diphosphohydrolase 4	Downregulated
A_24_P709	0.000526	0.0000128	-2.73	CD99	CD99 molecule	Downregulated
A_24_P912	0.000695	0.0000317	-2.73	AKNA	AT-hook transcription factor	Downregulated
A_23_P203	0.000925	0.0000653	-2.73	INTS4	integrator complex subunit 4	Downregulated
A_32_P584	0.000961	0.0000721	-2.73	KCND3	potassium channel, voltage gated Shal related subfamily D, member 3	Downregulated
A_33_P333	0.001203	0.000119	-2.73	FBRS	fibrosin	Downregulated
A_23_P152	0.002456	0.000416	-2.73	RSAD1	radical S-adenosyl methionine domain containing 1	Downregulated
A_33_P331	0.004645	0.00109	-2.73	ZNF749	zinc finger protein 749	Downregulated
A_24_P380	0.000589	0.0000204	-2.74	PANK3	pantothenate kinase 3	Downregulated
A_33_P327	0.000621	0.0000242	-2.74	UHRF2	ubiquitin-like with PHD and ring finger domains 2, E3 ubiquitin protein liç	Downregulated
A_24_P398	0.001062	0.0000913	-2.74	IFT43	intraflagellar transport 43	Downregulated
A_24_P244	0.001569	0.000195	-2.74	NUDT15	nudix (nucleoside diphosphate linked moiety X)-type motif 15	Downregulated
A_23_P208	0.001569	0.000195	-2.74	SIRT6	sirtuin 6	Downregulated
A_23_P492	0.001919	0.000278	-2.74	C19orf68	chromosome 19 open reading frame 68	Downregulated
A_24_P942	0.00069	0.0000304	-2.75	FRMD4A	FERM domain containing 4A	Downregulated
A_23_P252	0.000769	0.000043	-2.75	STRBP	spermatid perinuclear RNA binding protein	Downregulated
A_24_P132	0.000783	0.0000449	-2.75	GIMAP8	GTPase, IMAP family member 8	Downregulated
A_33_P323	0.0017	0.000226	-2.75	MYO18A	myosin XVIIIA	Downregulated
A_21_P001	0.002117	0.000332	-2.75	XLOC_l2_009804		Downregulated
A_23_P255	0.000525	0.0000112	-2.76	MANEA	mannosidase, endo-alpha	Downregulated
A_21_P001	0.001581	0.000197	-2.76	PACRGL	PARK2 co-regulated-like	Downregulated
A_24_P272	0.003909	0.000845	-2.76	MUSTN1	musculoskeletal, embryonic nuclear protein 1	Downregulated
A_24_P371	0.006209	0.00166	-2.76	FAM208A	family with sequence similarity 208, member A	Downregulated
A_23_P413	0.006309	0.0017	-2.76	MDM1	Mdm1 nuclear protein homolog (mouse)	Downregulated

A_23_P114	0.011083	0.00365	-2.76	MBTPS2	membrane-bound transcription factor peptidase, site 2	Downregulated
A_24_P946	0.000792	0.0000462	-2.77	ARIH2	ariadne RBR E3 ubiquitin protein ligase 2	Downregulated
A_23_P157	0.000914	0.0000639	-2.77	DNAJC30	DnaJ (Hsp40) homolog, subfamily C, member 30	Downregulated
A_23_P205	0.00109	0.0000969	-2.77	UTP14A	UTP14, U3 small nucleolar ribonucleoprotein, homolog A (yeast)	Downregulated
A_23_P882	0.001211	0.000121	-2.77	RBM23	RNA binding motif protein 23	Downregulated
A_33_P323	0.004387	0.001	-2.77	RIMS3	regulating synaptic membrane exocytosis 3	Downregulated
A_21_P000	0.000647	0.0000267	-2.78	C5orf56	chromosome 5 open reading frame 56	Downregulated
A_22_P000	0.000728	0.0000381	-2.78	LOC1019278	uncharacterized LOC101927841	Downregulated
A_33_P344	0.000728	0.0000381	-2.78	LOC286071	uncharacterized LOC286071	Downregulated
A_33_P324	0.002117	0.000333	-2.78	DCAF8	DDB1 and CUL4 associated factor 8	Downregulated
A_23_P400	0.004131	0.000914	-2.78	AMMECR1L	AMMECR1-like	Downregulated
A_33_P328	0.004483	0.00104	-2.78	GCNT7	glucosaminyl (N-acetyl) transferase family member 7	Downregulated
A_23_P141	0.006046	0.0016	-2.78	BCAS3	breast carcinoma amplified sequence 3	Downregulated
A_21_P000	0.000855	0.0000554	-2.79	lnc-SLC1A4-1	lnc-SLC1A4-1:1	Downregulated
A_23_P157	0.00108	0.000095	-2.79	KRT9	keratin 9, type I	Downregulated
A_21_P000	0.0011	0.0000994	-2.79	lnc-C15orf2-9	lnc-C15orf2-9:10	Downregulated
A_23_P100	0.001277	0.000134	-2.79	MYO19	myosin XIX	Downregulated
A_33_P323	0.001433	0.000165	-2.79	ZNF3	zinc finger protein 3	Downregulated
A_24_P932	0.001435	0.000166	-2.79	TMEM14E	transmembrane protein 14E	Downregulated
A_21_P001	0.003544	0.000732	-2.79	XLOC_l2_004595		Downregulated
A_23_P386	0.016097	0.00593	-2.79	DIRAS1	DIRAS family, GTP-binding RAS-like 1	Downregulated
A_21_P001	0.000537	0.000014	-2.8	lnc-AF16513ε	lnc-AF165138.7.1-3:2	Downregulated
A_23_P153	0.000717	0.0000365	-2.8	ACVR2A	activin A receptor, type IIA	Downregulated
A_33_P332	0.000893	0.0000604	-2.8	ZNF19	zinc finger protein 19	Downregulated
A_33_P324	0.001341	0.000146	-2.8	AFF1	AF4/FMR2 family, member 1	Downregulated
A_21_P000	0.001353	0.000149	-2.8	LOC1005057	uncharacterized LOC100505771	Downregulated
A_22_P000	0.001699	0.000226	-2.8	PRKXP1	protein kinase, X-linked, pseudogene 1	Downregulated
A_22_P000	0.002221	0.000359	-2.8	IGFBP7	insulin-like growth factor binding protein 7	Downregulated
A_23_P155	0.002219	0.000359	-2.8	NEK11	NIMA-related kinase 11	Downregulated
A_22_P000	0.002409	0.000405	-2.8	lnc-ZBTB38-1	lnc-ZBTB38-1:1	Downregulated
A_23_P113	0.000526	0.0000127	-2.81	USP19	ubiquitin specific peptidase 19	Downregulated
A_23_P415	0.000537	0.0000142	-2.81	METTL7A	methyltransferase like 7A	Downregulated
A_21_P001	0.000545	0.0000147	-2.81	XLOC_l2_006026		Downregulated
A_33_P341	0.000717	0.0000365	-2.81	DDX58	DEAD (Asp-Glu-Ala-Asp) box polypeptide 58	Downregulated
A_23_P383	0.001324	0.000143	-2.81	ZSWIM5	zinc finger, SWIM-type containing 5	Downregulated
A_23_P134	0.002302	0.000379	-2.81	THAP1	THAP domain containing, apoptosis associated protein 1	Downregulated
A_24_P279	0.007565	0.00219	-2.81	MRI1	methylthioribose-1-phosphate isomerase 1	Downregulated

A_22_P000	0.016069	0.00591	-2.81	lnc-PGBD5-1	lnc-PGBD5-1:1	Downregulated
A_23_P131	0.001612	0.000205	-2.82	EXT2	exostosin glycosyltransferase 2	Downregulated
A_33_P342	0.002832	0.000519	-2.82	WDPCP	WD repeat containing planar cell polarity effector	Downregulated
A_23_P706	0.000525	0.0000113	-2.83	LY86	lymphocyte antigen 86	Downregulated
A_22_P000	0.000612	0.0000234	-2.83	FLJ32255	uncharacterized LOC643977	Downregulated
A_22_P000	0.00071	0.0000353	-2.83	lnc-C10orf31-	lnc-C10orf31-2:1	Downregulated
A_22_P000	0.000896	0.0000607	-2.83	lnc-CD63-1	lnc-CD63-1:1	Downregulated
A_23_P320	0.001266	0.000133	-2.83	CEP128	centrosomal protein 128kDa	Downregulated
A_21_P000	0.00281	0.000513	-2.83	lnc-SMARCAL	lnc-SMARCAL1-2:5	Downregulated
A_22_P000	0.004513	0.00105	-2.83	LOC1005068	uncharacterized LOC100506844	Downregulated
A_21_P000	0.006568	0.0018	-2.83	lnc-GCNT1-2	lnc-GCNT1-2:1	Downregulated
A_23_P380	0.000577	0.0000183	-2.84	ANKRD13B	ankyrin repeat domain 13B	Downregulated
A_21_P000	0.000642	0.0000262	-2.84	SNORD100	small nucleolar RNA, C/D box 100	Downregulated
A_33_P330	0.001797	0.000248	-2.84	DBT	dihydrolipoamide branched chain transacylase E2	Downregulated
A_33_P325	0.002585	0.000453	-2.84	PLCB1	phospholipase C, beta 1 (phosphoinositide-specific)	Downregulated
A_21_P001	0.003024	0.000575	-2.84	XLOC_l2_015441		Downregulated
A_23_P771	0.000709	0.0000349	-2.85	DPH6	diphthamine biosynthesis 6	Downregulated
A_24_P751	0.000894	0.0000605	-2.85	HBD	hemoglobin, delta	Downregulated
A_33_P338	0.001397	0.000157	-2.85	DUSP28	dual specificity phosphatase 28	Downregulated
A_23_P317	0.001443	0.000168	-2.85	E2F5	E2F transcription factor 5, p130-binding	Downregulated
A_22_P000	0.002356	0.000392	-2.85	lnc-CCDC7-3	lnc-CCDC7-3:1	Downregulated
A_22_P000	0.004251	0.000957	-2.85	lnc-CDC25C-1	lnc-CDC25C-1:1	Downregulated
A_24_P330	0.004632	0.00109	-2.85	CALCOCO2	calcium binding and coiled-coil domain 2	Downregulated
A_33_P328	0.000515	9.85E-06	-2.86	NUP133	nucleoporin 133kDa	Downregulated
A_22_P000	0.000526	0.0000126	-2.86	lnc-EIF2AK4-3	lnc-EIF2AK4-3:1	Downregulated
A_23_P139	0.000577	0.000018	-2.86	GOLGA2P5	golgin A2 pseudogene 5	Downregulated
A_23_P400	0.000715	0.0000362	-2.86	PMS1	PMS1 postmeiotic segregation increased 1 (S. cerevisiae)	Downregulated
A_33_P333	0.001158	0.000111	-2.86	SHROOM3	shroom family member 3	Downregulated
A_24_P314	0.001365	0.000151	-2.86	SPC24	SPC24, NDC80 kinetochore complex component	Downregulated
A_22_P000	0.005238	0.0013	-2.86	lnc-ST7L-1	lnc-ST7L-1:1	Downregulated
A_21_P000	0.005271	0.00131	-2.86	MAPKAPK5-A	MAPKAPK5 antisense RNA 1	Downregulated
A_24_P108	0.005534	0.00141	-2.86	GOSR2	golgi SNAP receptor complex member 2	Downregulated
A_23_P949	0.007026	0.00198	-2.86	LETM1	leucine zipper-EF-hand containing transmembrane protein 1	Downregulated
A_23_P251	0.000526	0.0000127	-2.87	DCTN4	dynactin 4 (p62)	Downregulated
A_33_P328	0.000545	0.0000147	-2.87	KAT6B	K(lysine) acetyltransferase 6B	Downregulated
A_23_P361	0.000707	0.0000341	-2.87	SESN3	sestrin 3	Downregulated
A_24_P370	0.00124	0.000127	-2.87	WBSCR16	Williams-Beuren syndrome chromosome region 16	Downregulated

A_23_P911	0.003389	0.000685	-2.87	PECR	peroxisomal trans-2-enoyl-CoA reductase	Downregulated
A_33_P326	0.008796	0.00269	-2.87	TUBE1	tubulin, epsilon 1	Downregulated
A_23_P107	0.000543	0.0000146	-2.88	ETFB	electron-transfer-flavoprotein, beta polypeptide	Downregulated
A_22_P000	0.000776	0.0000438	-2.88	ZNF154	zinc finger protein 154	Downregulated
A_33_P325	0.001279	0.000135	-2.88	SETDB1	SET domain, bifurcated 1	Downregulated
A_33_P328	0.001522	0.000185	-2.88	CDK11A	cyclin-dependent kinase 11A	Downregulated
A_19_P008	0.001893	0.000272	-2.88	RNF213	ring finger protein 213	Downregulated
A_33_P321	0.002927	0.000545	-2.88	TTBK2	tau tubulin kinase 2	Downregulated
A_23_P166	0.003699	0.000779	-2.88	ITGB5	integrin, beta 5	Downregulated
A_19_P003	0.005192	0.00128	-2.88	HCG18	HLA complex group 18 (non-protein coding)	Downregulated
A_24_P392	0.006904	0.00193	-2.88	SIMC1	SUMO-interacting motifs containing 1	Downregulated
A_33_P682	0.000701	0.0000325	-2.89	LOC1005068	uncharacterized LOC100506844	Downregulated
A_33_P331	0.001443	0.000168	-2.89	CYB5R4	cytochrome b5 reductase 4	Downregulated
A_23_P416	0.001453	0.00017	-2.89	LRRC70	leucine rich repeat containing 70	Downregulated
A_23_P167	0.001511	0.000183	-2.89	FAM134B	family with sequence similarity 134, member B	Downregulated
A_22_P000	0.003171	0.000621	-2.89	lnc-FDXACB1	lnc-FDXACB1-1:1	Downregulated
A_23_P127	0.000603	0.0000224	-2.9	STK33	serine/threonine kinase 33	Downregulated
A_33_P336	0.000639	0.0000258	-2.9	MICA	MHC class I polypeptide-related sequence A	Downregulated
A_23_P117	0.0008	0.0000475	-2.9	ZFAND4	zinc finger, AN1-type domain 4	Downregulated
A_33_P324	0.001802	0.000249	-2.9	SMIM5	small integral membrane protein 5	Downregulated
A_33_P333	0.001959	0.00029	-2.9	SYNE1	spectrin repeat containing, nuclear envelope 1	Downregulated
A_33_P323	0.00542	0.00137	-2.9	FLJ16734	uncharacterized LOC641928	Downregulated
A_23_P553	0.000521	0.0000103	-2.91	FLOT2	flotillin 2	Downregulated
A_32_P191	0.001343	0.000147	-2.91	KCTD5	potassium channel tetramerization domain containing 5	Downregulated
A_23_P566	0.002279	0.000374	-2.91	MCEE	methylmalonyl CoA epimerase	Downregulated
A_22_P000	0.000515	9.18E-06	-2.92	lnc-SDCCAG8	lnc-SDCCAG8-1:1	Downregulated
A_23_P951	0.00056	0.0000164	-2.92	SEMA4B	sema domain, immunoglobulin domain (Ig), transmembrane domain (TM	Downregulated
A_32_P196	0.000837	0.0000527	-2.92	DPY19L4	dpy-19-like 4 (C. elegans)	Downregulated
A_23_P211	0.000837	0.0000527	-2.92	ZNF35	zinc finger protein 35	Downregulated
A_33_P336	0.001043	0.0000875	-2.92	UGGT1	UDP-glucose glycoprotein glucosyltransferase 1	Downregulated
A_33_P336	0.001054	0.0000893	-2.92	TRPS1	trichorhinophalangeal syndrome I	Downregulated
A_33_P342	0.001524	0.000185	-2.92	ARPC5	actin related protein 2/3 complex, subunit 5, 16kDa	Downregulated
A_33_P340	0.002221	0.000359	-2.92	LOC283788	FSHD region gene 1 pseudogene	Downregulated
A_21_P001	0.006241	0.00168	-2.92	LOC1005070	uncharacterized LOC100507006	Downregulated
A_23_P202	0.007422	0.00213	-2.92	SLC35C1	solute carrier family 35 (GDP-fucose transporter), member C1	Downregulated
A_33_P327	0.000502	8.36E-06	-2.93	REC8	REC8 meiotic recombination protein	Downregulated
A_22_P000	0.00058	0.0000196	-2.93	lnc-SNX11-2	lnc-SNX11-2:1	Downregulated

A_23_P917	0.00065	0.000027	-2.93	TNFRSF13C	tumor necrosis factor receptor superfamily, member 13C	Downregulated
A_33_P375	0.002207	0.000355	-2.93	TOM1	target of myb1 (chicken)	Downregulated
A_32_P539	0.000527	0.000013	-2.94	LINC01410	long intergenic non-protein coding RNA 1410	Downregulated
A_22_P000	0.00062	0.0000239	-2.94	lnc-SRPR-1	lnc-SRPR-1:1	Downregulated
A_23_P331	0.00069	0.0000304	-2.94	TTYH3	tweety family member 3	Downregulated
A_21_P000	0.000995	0.0000785	-2.94	MCPH1	microcephalin 1	Downregulated
A_23_P252	0.001203	0.000119	-2.94	EAF2	ELL associated factor 2	Downregulated
A_23_P390	0.001815	0.000253	-2.94	PSKH1	protein serine kinase H1	Downregulated
A_33_P342	0.003232	0.000639	-2.94	TRIM14	tripartite motif containing 14	Downregulated
A_23_P164	0.006105	0.00162	-2.94	FCER2	Fc fragment of IgE, low affinity II, receptor for (CD23)	Downregulated
A_22_P000	0.006604	0.00182	-2.94	lnc-CHADL-2	lnc-CHADL-2:1	Downregulated
A_22_P000	0.00774	0.00226	-2.94	lnc-DHX38-5	lnc-DHX38-5:1	Downregulated
A_33_P325	0.000499	8.01E-06	-2.95	CNTRL	centriolin	Downregulated
A_33_P327	0.0005	8.07E-06	-2.95	METTL16	methyltransferase like 16	Downregulated
A_32_P198	0.000713	0.000036	-2.95	SREK1IP1	SREK1-interacting protein 1	Downregulated
A_33_P323	0.000814	0.00005	-2.95	RNFT2	ring finger protein, transmembrane 2	Downregulated
A_23_P422	0.001493	0.000179	-2.95	ARHGAP20	Rho GTPase activating protein 20	Downregulated
A_23_P302	0.001662	0.000216	-2.95	PLK2	polo-like kinase 2	Downregulated
A_23_P156	0.001997	0.0003	-2.95	ECI2	enoyl-CoA delta isomerase 2	Downregulated
A_21_P000	0.002376	0.000398	-2.95	LINC01419	long intergenic non-protein coding RNA 1419	Downregulated
A_23_P132	0.00596	0.00157	-2.95	ZMAT5	zinc finger, matrin-type 5	Downregulated
A_23_P142	0.007099	0.00201	-2.95	GNA11	guanine nucleotide binding protein (G protein), alpha 11 (Gq class)	Downregulated
A_21_P001	0.00995	0.00317	-2.95	XLOC_l2_009184		Downregulated
A_22_P000	0.017144	0.00643	-2.95	lnc-HLA-DQA	lnc-HLA-DQA1-5:1	Downregulated
A_33_P340	0.000578	0.0000193	-2.96	UQCC3	ubiquinol-cytochrome c reductase complex assembly factor 3	Downregulated
A_33_P321	0.001478	0.000176	-2.96	ZCCHC6	zinc finger, CCHC domain containing 6	Downregulated
A_23_P254	0.001501	0.000181	-2.96	C12orf4	chromosome 12 open reading frame 4	Downregulated
A_33_P340	0.001626	0.000207	-2.96	SEC14L1P1	SEC14-like 1 pseudogene 1	Downregulated
A_23_P103	0.00255	0.000443	-2.96	B4GALT3	UDP-Gal:betaGlcNAc beta 1,4- galactosyltransferase, polypeptide 3	Downregulated
A_33_P336	0.000498	0.0000077	-2.97	RTN3	reticulon 3	Downregulated
A_23_P215	0.000513	8.94E-06	-2.97	BAZ1B	bromodomain adjacent to zinc finger domain, 1B	Downregulated
A_33_P322	0.000614	0.0000235	-2.97	COL4A4	collagen, type IV, alpha 4	Downregulated
A_23_P233	0.000857	0.0000558	-2.97	MLLT11	myeloid/lymphoid or mixed-lineage leukemia (trithorax homolog, Drosopl	Downregulated
A_22_P000	0.001055	0.0000897	-2.97	GSN	gelsolin	Downregulated
A_24_P406	0.001369	0.000151	-2.97	MLLT10	myeloid/lymphoid or mixed-lineage leukemia (trithorax homolog, Drosopl	Downregulated
A_22_P000	0.00166	0.000216	-2.97	LOC340512	uncharacterized LOC340512	Downregulated
A_23_P398	0.001763	0.00024	-2.97	HIP1R	huntingtin interacting protein 1 related	Downregulated

A_24_P586	0.006035	0.0016	-2.97	GRAMD1B	GRAM domain containing 1B	Downregulated
A_23_P692	0.000532	0.0000134	-2.98	SUMF1	sulfatase modifying factor 1	Downregulated
A_23_P838	0.000658	0.0000276	-2.98	CA8	carbonic anhydrase VIII	Downregulated
A_24_P571	0.001824	0.000256	-2.98	PROSER3	proline and serine rich 3	Downregulated
A_24_P365	0.0027	0.000483	-2.98	NKAPP1	NFKB activating protein pseudogene 1	Downregulated
A_23_P100	0.007415	0.00213	-2.98	ZCCHC14	zinc finger, CCHC domain containing 14	Downregulated
A_22_P000	0.008429	0.00254	-2.98	lnc-ATP4B-1	lnc-ATP4B-1:1	Downregulated
A_33_P331	0.000577	0.0000185	-2.99	MTERF4	mitochondrial transcription termination factor 4	Downregulated
A_24_P111	0.000693	0.0000308	-2.99	FAM172A	family with sequence similarity 172, member A	Downregulated
A_23_P754	0.000736	0.0000387	-2.99	MEN1	multiple endocrine neoplasia I	Downregulated
A_21_P000	0.00075	0.0000405	-2.99	SNORD75	small nucleolar RNA, C/D box 75	Downregulated
A_24_P320	0.000869	0.0000576	-2.99	PRKRIP1	PRKR interacting protein 1 (IL11 inducible)	Downregulated
A_33_P327	0.002757	0.000499	-2.99	HLA-DPB1	major histocompatibility complex, class II, DP beta 1	Downregulated
A_23_P446	0.00411	0.000907	-2.99	ADAMTS12	ADAM metallopeptidase with thrombospondin type 1 motif, 12	Downregulated
A_24_P364	0.004211	0.000943	-2.99	NDUFC2	NADH dehydrogenase (ubiquinone) 1, subcomplex unknown, 2, 14.5kD	Downregulated
A_24_P344	0.000584	0.0000199	-3	RNF167	ring finger protein 167	Downregulated
A_22_P000	0.000683	0.0000299	-3	lnc-KLHL25-3	lnc-KLHL25-3:1	Downregulated
A_23_P148	0.00383	0.00082	-3	ADI1	acireductone dioxygenase 1	Downregulated
A_23_P207	0.000711	0.0000356	-3.01	RAD51C	RAD51 paralog C	Downregulated
A_23_P100	0.00124	0.000127	-3.01	ORC6	origin recognition complex, subunit 6	Downregulated
A_22_P000	0.001291	0.000137	-3.01	LRRC8B	leucine rich repeat containing 8 family, member B	Downregulated
A_23_P390	0.001314	0.000141	-3.01	ZNF630	zinc finger protein 630	Downregulated
A_21_P000	0.001991	0.000298	-3.01	SARS	seryl-tRNA synthetase	Downregulated
A_33_P341	0.00362	0.000756	-3.01	LINC00886	long intergenic non-protein coding RNA 886	Downregulated
A_33_P334	0.004325	0.000983	-3.01	RABL2A	RAB, member of RAS oncogene family-like 2A	Downregulated
A_33_P332	0.005877	0.00154	-3.01	SBF1	SET binding factor 1	Downregulated
A_24_P252	0.000498	7.85E-06	-3.02	TRIB1	tribbles pseudokinase 1	Downregulated
A_33_P357	0.000504	8.58E-06	-3.02	AP3D1	adaptor-related protein complex 3, delta 1 subunit	Downregulated
A_33_P341	0.000964	0.000073	-3.02	PURA	purine-rich element binding protein A	Downregulated
A_23_P128	0.000516	0.00001	-3.03	CARS2	cysteinyl-tRNA synthetase 2, mitochondrial (putative)	Downregulated
A_23_P502	0.000603	0.0000222	-3.03	CYB5R3	cytochrome b5 reductase 3	Downregulated
A_33_P328	0.00125	0.000129	-3.03	NSF	N-ethylmaleimide-sensitive factor	Downregulated
A_23_P852	0.002591	0.000454	-3.03	TMEM106B	transmembrane protein 106B	Downregulated
A_23_P414	0.007089	0.00201	-3.03	TBC1D9	TBC1 domain family, member 9 (with GRAM domain)	Downregulated
A_33_P326	0.000489	7.32E-06	-3.04	SLC25A23	solute carrier family 25 (mitochondrial carrier; phosphate carrier), membe	Downregulated
A_22_P000	0.000525	0.000011	-3.04	LOC1019289	uncharacterized LOC101928954	Downregulated
A_23_P446	0.000601	0.0000216	-3.04	ECT2	epithelial cell transforming 2	Downregulated

A_22_P000	0.001117	0.000103	-3.04	lnc-EFR3B-3	lnc-EFR3B-3:1	Downregulated
A_33_P337	0.001134	0.000106	-3.04	RBM14	RNA binding motif protein 14	Downregulated
A_33_P321	0.001836	0.000258	-3.04	TRIQK	triple QxxK/R motif containing	Downregulated
A_21_P000	0.00275	0.000497	-3.04	RNF103	ring finger protein 103	Downregulated
A_33_P327	0.004032	0.000883	-3.04	GALM	galactose mutarotase (aldose 1-epimerase)	Downregulated
A_33_P336	0.007627	0.00222	-3.04	EIF4ENIF1	eukaryotic translation initiation factor 4E nuclear import factor 1	Downregulated
A_33_P341	0.000526	0.0000125	-3.05	HNRNPU-AS1	HNRNPU antisense RNA 1	Downregulated
A_33_P333	0.000577	0.0000181	-3.05	HKDC1	hexokinase domain containing 1	Downregulated
A_23_P128	0.001115	0.000102	-3.05	FANCF	Fanconi anemia, complementation group F	Downregulated
A_21_P000	0.001952	0.000287	-3.05	SNORD44	small nucleolar RNA, C/D box 44	Downregulated
A_33_P327	0.005428	0.00137	-3.05	CELF2-AS1	CELF2 antisense RNA 1	Downregulated
A_24_P481	0.005933	0.00156	-3.05	LOC1001315	uncharacterized LOC100131564	Downregulated
A_22_P000	0.000504	8.56E-06	-3.06	lnc-CTU2-1	lnc-CTU2-1:1	Downregulated
A_23_P217	0.000592	0.0000207	-3.06	DPM2	dolichyl-phosphate mannosyltransferase polypeptide 2, regulatory subu	Downregulated
A_23_P144	0.000645	0.0000264	-3.06	SSUH2	ssu-2 homolog (C. elegans)	Downregulated
A_22_P000	0.000662	0.0000279	-3.06	lnc-FAM184B	lnc-FAM184B-1:1	Downregulated
A_33_P327	0.000739	0.0000391	-3.06	NR1D2	nuclear receptor subfamily 1, group D, member 2	Downregulated
A_23_P119	0.000802	0.000048	-3.06	CYTH2	cytohesin 2	Downregulated
A_23_P150	0.001132	0.000105	-3.06	C2CD3	C2 calcium-dependent domain containing 3	Downregulated
A_23_P215	0.001626	0.000208	-3.06	KIAA0408	KIAA0408	Downregulated
A_23_P446	0.003709	0.000782	-3.06	ANAPC7	anaphase promoting complex subunit 7	Downregulated
A_33_P327	0.00056	0.0000157	-3.07	lnc-KLHL36-1	lnc-KLHL36-1:1	Downregulated
A_24_P157	0.00067	0.0000288	-3.07	IL17RB	interleukin 17 receptor B	Downregulated
A_22_P000	0.000697	0.0000319	-3.07	SPAG5-AS1	SPAG5 antisense RNA 1	Downregulated
A_23_P216	0.002078	0.000322	-3.07	EXOSC2	exosome component 2	Downregulated
A_24_P185	0.002757	0.000499	-3.07	NDRG3	NDRG family member 3	Downregulated
A_24_P331	0.003057	0.000585	-3.07	GUCD1	guanylyl cyclase domain containing 1	Downregulated
A_23_P890	0.000756	0.0000413	-3.08	CLUHP3	clustered mitochondria (cluA/CLU1) homolog pseudogene 3	Downregulated
A_33_P342	0.001042	0.000087	-3.08	MRPS34	mitochondrial ribosomal protein S34	Downregulated
A_23_P372	0.001066	0.0000922	-3.08	ITPKB	inositol-trisphosphate 3-kinase B	Downregulated
A_33_P329	0.001863	0.000265	-3.08	NAAA	N-acylethanolamine acid amidase	Downregulated
A_33_P334	0.002553	0.000444	-3.08	LOC1001304	uncharacterized LOC100130458	Downregulated
A_22_P000	0.004207	0.000942	-3.08	lnc-DBT-2	lnc-DBT-2:1	Downregulated
A_21_P000	0.000718	0.0000367	-3.09	VTRNA1-1	vault RNA 1-1	Downregulated
A_33_P338	0.00084	0.0000532	-3.09	AGO1	argonaute RISC catalytic component 1	Downregulated
A_23_P273	0.004588	0.00107	-3.09	TSHZ1	teashirt zinc finger homeobox 1	Downregulated
A_22_P000	0.005951	0.00156	-3.09	HDAC9	histone deacetylase 9	Downregulated

A_33_P321	0.006385	0.00173	-3.09	FECH	ferrochelatase	Downregulated
A_23_P361	0.000502	8.47E-06	-3.1	METTL6	methyltransferase like 6	Downregulated
A_21_P001	0.000664	0.0000283	-3.1	lnc-PRR11-1	lnc-PRR11-1:1	Downregulated
A_24_P361	0.000664	0.0000283	-3.1	ZNF839	zinc finger protein 839	Downregulated
A_24_P919	0.000701	0.0000327	-3.1	WDR17	WD repeat domain 17	Downregulated
A_22_P000	0.000718	0.0000368	-3.1	LINC01585	long intergenic non-protein coding RNA 1585	Downregulated
A_33_P341	0.00074	0.0000393	-3.1	DNAJA2	DnaJ (Hsp40) homolog, subfamily A, member 2	Downregulated
A_24_P116	0.000839	0.0000532	-3.1	PSMD9	proteasome (prosome, macropain) 26S subunit, non-ATPase, 9	Downregulated
A_33_P341	0.001054	0.0000895	-3.1	SHROOM3	shroom family member 3	Downregulated
A_33_P329	0.001187	0.000116	-3.1	CCDC125	coiled-coil domain containing 125	Downregulated
A_23_P391	0.000486	0.0000071	-3.11	RCAN3	RCAN family member 3	Downregulated
A_24_P920	0.000559	0.0000155	-3.11	PPIA	peptidylprolyl isomerase A (cyclophilin A)	Downregulated
A_23_P337	0.000717	0.0000365	-3.11	SERBP1	SERPINE1 mRNA binding protein 1	Downregulated
A_32_P100	0.000855	0.0000555	-3.11	MTURN	maturin, neural progenitor differentiation regulator homolog (Xenopus)	Downregulated
A_21_P000	0.001175	0.000114	-3.11	LOC1005076	uncharacterized LOC100507616	Downregulated
A_21_P001	0.004458	0.00103	-3.11	LOC1002894	uncharacterized LOC100289455	Downregulated
A_24_P401	0.000701	0.0000327	-3.12	KIAA0556	KIAA0556	Downregulated
A_23_P168	0.001036	0.000086	-3.12	TP53INP1	tumor protein p53 inducible nuclear protein 1	Downregulated
A_21_P001	0.00166	0.000216	-3.12	FTO-IT1	FTO intronic transcript 1 (non-protein coding)	Downregulated
A_22_P000	0.002696	0.000482	-3.12	lnc-NUMB-2	lnc-NUMB-2:1	Downregulated
A_23_P112	0.004355	0.000994	-3.12	CNOT10	CCR4-NOT transcription complex, subunit 10	Downregulated
A_33_P336	0.005953	0.00157	-3.12	RPS6	ribosomal protein S6	Downregulated
A_21_P001	0.000501	8.17E-06	-3.13	XLOC_l2_003992		Downregulated
A_22_P000	0.000765	0.0000426	-3.13	SLC7A11-AS	SLC7A11 antisense RNA 1	Downregulated
A_23_P934	0.001031	0.0000851	-3.13	BCKDHB	branched chain keto acid dehydrogenase E1, beta polypeptide	Downregulated
A_23_P969	0.001481	0.000177	-3.13	NVL	nuclear VCP-like	Downregulated
A_33_P334	0.002498	0.000429	-3.13	NAIP	NLR family, apoptosis inhibitory protein	Downregulated
A_21_P001	0.004031	0.000883	-3.13	XLOC_l2_011911		Downregulated
A_33_P341	0.006535	0.00179	-3.13	HPS1	Hermansky-Pudlak syndrome 1	Downregulated
A_23_P593	0.002804	0.000511	-3.14	RSPH3	radial spoke 3 homolog (Chlamydomonas)	Downregulated
A_19_P003	0.005245	0.0013	-3.14	XLOC_l2_015760		Downregulated
A_23_P367	0.000502	8.39E-06	-3.15	PCBD1	pterin-4 alpha-carbinolamine dehydratase/dimerization cofactor of hepat	Downregulated
A_23_P963	0.000526	0.0000117	-3.15	PRAF2	PRA1 domain family, member 2	Downregulated
A_22_P000	0.000743	0.0000399	-3.15	PRR34-AS1	PRR34 antisense RNA 1	Downregulated
A_33_P335	0.000896	0.0000607	-3.15	HMG20B	high mobility group 20B	Downregulated
A_23_P981	0.001085	0.0000959	-3.15	HRAS	Harvey rat sarcoma viral oncogene homolog	Downregulated
A_23_P300	0.001213	0.000121	-3.15	LMBR1	limb development membrane protein 1	Downregulated

A_33_P322	0.001403	0.000159	-3.15	SNORA74B	small nucleolar RNA, H/ACA box 74B	Downregulated
A_33_P332	0.001524	0.000185	-3.15	ZNF605	zinc finger protein 605	Downregulated
A_23_P250	0.000642	0.0000262	-3.16	ZDHHC14	zinc finger, DHHC-type containing 14	Downregulated
A_24_P403	0.000752	0.0000408	-3.16	PHF20L1	PHD finger protein 20-like 1	Downregulated
A_22_P000	0.000883	0.000059	-3.16	lnc-TSPAN5-2	lnc-TSPAN5-2:2	Downregulated
A_33_P335	0.005377	0.00135	-3.16	MTSS1	metastasis suppressor 1	Downregulated
A_23_P200	0.005531	0.00141	-3.16	PCNXL2	pecanex-like 2 (Drosophila)	Downregulated
A_33_P336	0.000526	0.0000124	-3.17	CSGALNACT	chondroitin sulfate N-acetylgalactosaminyltransferase 1	Downregulated
A_33_P323	0.000665	0.0000283	-3.17	TRIM47	tripartite motif containing 47	Downregulated
A_24_P277	0.000839	0.000053	-3.17	RAB43	RAB43, member RAS oncogene family	Downregulated
A_33_P376	0.000846	0.0000538	-3.17	SIRT5	sirtuin 5	Downregulated
A_33_P321	0.000931	0.0000664	-3.17	RNF43	ring finger protein 43	Downregulated
A_23_P207	0.000977	0.0000753	-3.17	MAP3K14	mitogen-activated protein kinase kinase kinase 14	Downregulated
A_23_P156	0.002468	0.00042	-3.17	SOBP	sine oculis binding protein homolog (Drosophila)	Downregulated
A_23_P215	0.000525	0.0000115	-3.18	PODXL	podocalyxin-like	Downregulated
A_22_P000	0.000526	0.000012	-3.18	LOC1027246	uncharacterized LOC102724601	Downregulated
A_23_P966	0.00056	0.000016	-3.18	PRPS2	phosphoribosyl pyrophosphate synthetase 2	Downregulated
A_23_P841	0.000563	0.0000168	-3.18	PITPNC1	phosphatidylinositol transfer protein, cytoplasmic 1	Downregulated
A_33_P327	0.001157	0.00011	-3.18	PHF20L1	PHD finger protein 20-like 1	Downregulated
A_23_P129	0.001341	0.000146	-3.18	IDH2	isocitrate dehydrogenase 2 (NADP+), mitochondrial	Downregulated
A_23_P257	0.001576	0.000197	-3.18	CD180	CD180 molecule	Downregulated
A_23_P217	0.00766	0.00223	-3.18	SLC31A2	solute carrier family 31 (copper transporter), member 2	Downregulated
A_23_P132	0.000483	0.0000064	-3.19	SIK1	salt-inducible kinase 1	Downregulated
A_33_P336	0.000585	0.0000201	-3.19	ABCG8	ATP-binding cassette, sub-family G (WHITE), member 8	Downregulated
A_24_P253	0.000707	0.0000337	-3.19	FLOT2	flotillin 2	Downregulated
A_23_P479	0.001179	0.000115	-3.19	MED13L	mediator complex subunit 13-like	Downregulated
A_23_P139	0.002054	0.000315	-3.19	TRIM44	tripartite motif containing 44	Downregulated
A_23_P358	0.004639	0.00109	-3.19	GOLGA6L4	golgin A6 family-like 4	Downregulated
A_33_P339	0.000793	0.0000466	-3.2	LINC00494	long intergenic non-protein coding RNA 494	Downregulated
A_23_P168	0.000896	0.0000608	-3.2	CCDC126	coiled-coil domain containing 126	Downregulated
A_23_P365	0.002874	0.000532	-3.2	MDN1	MDN1, midasin homolog (yeast)	Downregulated
A_22_P000	0.005105	0.00125	-3.2	KIAA0040	KIAA0040	Downregulated
A_24_P229	0.001115	0.000102	-3.21	SMIM22	small integral membrane protein 22	Downregulated
A_23_P443	0.001604	0.000202	-3.21	CASKIN2	CASK interacting protein 2	Downregulated
A_22_P000	0.000701	0.0000326	-3.22	LUZP1	leucine zipper protein 1	Downregulated
A_23_P898	0.000967	0.0000735	-3.22	TMEM241	transmembrane protein 241	Downregulated
A_23_P335	0.001179	0.000115	-3.22	HIBADH	3-hydroxyisobutyrate dehydrogenase	Downregulated

A_33_P324	0.001885	0.00027	-3.22	C17orf59	chromosome 17 open reading frame 59	Downregulated
A_23_P550	0.000483	5.72E-06	-3.23	SLC38A10	solute carrier family 38, member 10	Downregulated
A_24_P303	0.000483	6.04E-06	-3.23	BMI1	BMI1 proto-oncogene, polycomb ring finger	Downregulated
A_22_P000	0.000501	8.13E-06	-3.23	lnc-TMEM30B	lnc-TMEM30B-1:3	Downregulated
A_21_P001	0.000515	9.85E-06	-3.23	XLOC_l2_005179		Downregulated
A_33_P323	0.000601	0.0000216	-3.23	ANG	angiogenin, ribonuclease, RNase A family, 5	Downregulated
A_24_P168	0.000601	0.0000218	-3.24	GNAS	GNAS complex locus	Downregulated
A_24_P109	0.000771	0.0000432	-3.24	NBEAL1	neurobeachin-like 1	Downregulated
A_23_P175	0.000863	0.0000566	-3.24	KIF16B	kinesin family member 16B	Downregulated
A_21_P000	0.004288	0.000969	-3.24	lnc-CBWD3-1	lnc-CBWD3-1:1	Downregulated
A_22_P000	0.000486	6.98E-06	-3.25	lnc-SERAC1-2	lnc-SERAC1-2:1	Downregulated
A_22_P000	0.000831	0.000052	-3.25	KCNQ5-AS1	KCNQ5 antisense RNA 1	Downregulated
A_22_P000	0.002459	0.000417	-3.25	PSMA3-AS1	PSMA3 antisense RNA 1	Downregulated
A_23_P636	0.000483	5.48E-06	-3.26	IDE	insulin-degrading enzyme	Downregulated
A_24_P398	0.000665	0.0000284	-3.26	UNC45A	unc-45 homolog A (C. elegans)	Downregulated
A_24_P443	0.004371	0.000999	-3.26	UGGT2	UDP-glucose glycoprotein glucosyltransferase 2	Downregulated
A_23_P151	0.000525	0.0000113	-3.27	TCHP	trichoplein, keratin filament binding	Downregulated
A_23_P156	0.000695	0.0000314	-3.27	ADTRP	androgen-dependent TFPI-regulating protein	Downregulated
A_23_P523	0.000801	0.0000479	-3.27	NDST2	N-deacetylase/N-sulfotransferase (heparan glucosaminyl) 2	Downregulated
A_33_P339	0.000855	0.0000555	-3.27	EXOC7	exocyst complex component 7	Downregulated
A_24_P114	0.000521	0.0000104	-3.28	CHMP2B	charged multivesicular body protein 2B	Downregulated
A_33_P325	0.000669	0.0000286	-3.28	LOC1001292	beta-defensin 131-like	Downregulated
A_23_P339	0.003119	0.000604	-3.28	NUP62CL	nucleoporin 62kDa C-terminal like	Downregulated
A_23_P411	0.000515	9.49E-06	-3.29	PER2	period circadian clock 2	Downregulated
A_24_P319	0.000639	0.0000256	-3.29	NEK2	NIMA-related kinase 2	Downregulated
A_24_P298	0.002241	0.000364	-3.29	LOC1001307	uncharacterized LOC100130744	Downregulated
A_23_P153	0.002709	0.000486	-3.29	GNG7	guanine nucleotide binding protein (G protein), gamma 7	Downregulated
A_32_P438	0.001098	0.0000987	-3.3	NEUROG2	neurogenin 2	Downregulated
A_24_P940	0.00126	0.000131	-3.3	SETX	senataxin	Downregulated
A_24_P145	0.001555	0.000192	-3.3	DTNBP1	dystrobrevin binding protein 1	Downregulated
A_22_P000	0.003027	0.000576	-3.3	lnc-RPL7L1-1	lnc-RPL7L1-1:1	Downregulated
A_23_P411	0.003535	0.000728	-3.3	LRRC34	leucine rich repeat containing 34	Downregulated
A_23_P135	0.000483	5.83E-06	-3.31	MRPS2	mitochondrial ribosomal protein S2	Downregulated
A_33_P334	0.000526	0.0000121	-3.31	EHBP1	EH domain binding protein 1	Downregulated
A_21_P001	0.00056	0.0000156	-3.31	LOC1019284	serine/threonine-protein kinase tousled-like 2-like	Downregulated
A_24_P791	0.000786	0.0000453	-3.31	NUFIP2	nuclear fragile X mental retardation protein interacting protein 2	Downregulated
A_21_P000	0.001108	0.000101	-3.31	SNORA55	small nucleolar RNA, H/ACA box 55	Downregulated

A_33_P330	0.000483	5.49E-06	-3.32	CR2	complement component (3d/Epstein Barr virus) receptor 2	Downregulated
A_33_P336	0.000498	7.69E-06	-3.32	EIF4ENIF1	eukaryotic translation initiation factor 4E nuclear import factor 1	Downregulated
A_23_P640	0.00055	0.0000151	-3.32	FERMT3	fermitin family member 3	Downregulated
A_24_P329	0.004738	0.00112	-3.32	SIK3	SIK family kinase 3	Downregulated
A_23_P118	0.000778	0.0000441	-3.33	C1orf112	chromosome 1 open reading frame 112	Downregulated
A_23_P940	0.000948	0.0000695	-3.33	TRRAP	transformation/transcription domain-associated protein	Downregulated
A_23_P122	0.000995	0.0000789	-3.33	AVL9	AVL9 homolog (S. cerevisiase)	Downregulated
A_24_P181	0.001062	0.000091	-3.33	TMEM135	transmembrane protein 135	Downregulated
A_33_P334	0.001912	0.000276	-3.33	DHRS12	dehydrogenase/reductase (SDR family) member 12	Downregulated
A_33_P340	0.002053	0.000315	-3.33	PPM1M	protein phosphatase, Mg2+/Mn2+ dependent, 1M	Downregulated
A_24_P244	0.003134	0.000609	-3.33	EXOG	endo/exonuclease (5'-3'), endonuclease G-like	Downregulated
A_22_P000	0.000556	0.0000153	-3.34	lnc-RP11-279	lnc-RP11-279O9.4.1-2:1	Downregulated
A_23_P194	0.000575	0.0000176	-3.34	MDC1	mediator of DNA-damage checkpoint 1	Downregulated
A_33_P342	0.000579	0.0000195	-3.34	EIF3D	eukaryotic translation initiation factor 3, subunit D	Downregulated
A_21_P001	0.00062	0.000024	-3.34	XLOC_l2_012210		Downregulated
A_32_P118	0.001124	0.000104	-3.34	DENND6A	DENN/MADD domain containing 6A	Downregulated
A_24_P218	0.001297	0.000138	-3.34	CDCA3	cell division cycle associated 3	Downregulated
A_24_P126	0.001754	0.000238	-3.34	SLC12A9	solute carrier family 12, member 9	Downregulated
A_24_P413	0.004648	0.00109	-3.34	CENPA	centromere protein A	Downregulated
A_23_P210	0.000483	5.11E-06	-3.35	CDC25B	cell division cycle 25B	Downregulated
A_23_P368	0.000521	0.0000105	-3.35	LRRC56	leucine rich repeat containing 56	Downregulated
A_23_P201	0.000603	0.000022	-3.35	PPP1R12B	protein phosphatase 1, regulatory subunit 12B	Downregulated
A_21_P000	0.001081	0.0000952	-3.35	LOC1019276	uncharacterized LOC101927686	Downregulated
A_24_P535	0.001399	0.000159	-3.35	CHAF1A	chromatin assembly factor 1, subunit A (p150)	Downregulated
A_33_P325	0.001626	0.000208	-3.35	KDM7A	lysine (K)-specific demethylase 7A	Downregulated
A_33_P327	0.001949	0.000286	-3.35	LOC90784	uncharacterized LOC90784	Downregulated
A_33_P331	0.000486	7.19E-06	-3.36	MGAT1	mannosyl (alpha-1,3-)-glycoprotein beta-1,2-N-acetylglucosaminyltransfe	Downregulated
A_23_P414	0.000526	0.0000127	-3.36	PLXNC1	plexin C1	Downregulated
A_21_P000	0.000814	0.0000499	-3.36	lnc-PRKAB2-1	lnc-PRKAB2-1:1	Downregulated
A_23_P345	0.001228	0.000125	-3.36	SCLY	selenocysteine lyase	Downregulated
A_33_P362	0.00056	0.0000165	-3.37	PKI55	DKFZp434H1419	Downregulated
A_33_P378	0.000795	0.0000469	-3.37	SCAMP1-AS1	SCAMP1 antisense RNA 1	Downregulated
A_22_P000	0.000828	0.0000517	-3.37	lnc-RP11-33N	lnc-RP11-33N16.1.1-1:1	Downregulated
A_23_P320	0.001197	0.000118	-3.37	SLC35D2	solute carrier family 35 (UDP-GlcNAc/UDP-glucose transporter), member	Downregulated
A_21_P000	0.005093	0.00124	-3.37	lnc-ZSCAN2-2	lnc-ZSCAN2-2:1	Downregulated
A_23_P971	0.002568	0.000448	-3.38	MTFR1L	mitochondrial fission regulator 1-like	Downregulated
A_24_P380	0.002756	0.000499	-3.38	ARHGAP24	Rho GTPase activating protein 24	Downregulated

A_23_P694	0.000483	5.13E-06	-3.39	GPR15	G protein-coupled receptor 15	Downregulated
A_23_P948	0.000486	6.83E-06	-3.39	S100A4	S100 calcium binding protein A4	Downregulated
A_22_P000	0.000515	9.66E-06	-3.39	lnc-HNRNPA1	lnc-HNRNPA1-1:1	Downregulated
A_24_P641	0.000577	0.0000182	-3.39	SLC25A37	solute carrier family 25 (mitochondrial iron transporter), member 37	Downregulated
A_33_P380	0.000578	0.0000188	-3.39	LOC286437	uncharacterized LOC286437	Downregulated
A_23_P140	0.000763	0.000042	-3.39	DNAJC17	DnaJ (Hsp40) homolog, subfamily C, member 17	Downregulated
A_23_P679	0.001198	0.000118	-3.39	C1D	C1D nuclear receptor corepressor	Downregulated
A_21_P000	0.001426	0.000164	-3.39	SNORD59A	small nucleolar RNA, C/D box 59A	Downregulated
A_23_P294	0.001548	0.000191	-3.39	GYG1	glycogenin 1	Downregulated
A_19_P003	0.001885	0.00027	-3.39	PVT1	Pvt1 oncogene (non-protein coding)	Downregulated
A_22_P000	0.005619	0.00144	-3.39	lnc-AC13505(	lnc-AC135050.1-1:1	Downregulated
A_24_P237	0.001069	0.0000936	-3.4	FAM102B	family with sequence similarity 102, member B	Downregulated
A_23_P132	0.001134	0.000106	-3.4	VGLL4	vestigial-like family member 4	Downregulated
A_33_P334	0.000483	0.0000063	-3.41	PRDM1	PR domain containing 1, with ZNF domain	Downregulated
A_23_P270	0.000486	6.74E-06	-3.41	TMEM98	transmembrane protein 98	Downregulated
A_33_P344	0.000711	0.0000357	-3.41	FRMD4B	FERM domain containing 4B	Downregulated
A_23_P152	0.000961	0.0000719	-3.41	EFTUD1	elongation factor Tu GTP binding domain containing 1	Downregulated
A_24_P177	0.001211	0.000121	-3.41	TBC1D9B	TBC1 domain family, member 9B (with GRAM domain)	Downregulated
A_21_P001	0.000483	4.84E-06	-3.42	XLOC_l2_005781		Downregulated
A_33_P365	0.000483	5.05E-06	-3.42	ERCC8	excision repair cross-complementation group 8	Downregulated
A_33_P321	0.000537	0.0000141	-3.42	C15orf61	chromosome 15 open reading frame 61	Downregulated
A_33_P333	0.00055	0.000015	-3.42	B4GALT3	UDP-Gal:betaGlcNAc beta 1,4- galactosyltransferase, polypeptide 3	Downregulated
A_22_P000	0.000565	0.000017	-3.42	ZRANB2-AS1	ZRANB2 antisense RNA 1	Downregulated
A_23_P363	0.00179	0.000246	-3.42	FBXO3	F-box protein 3	Downregulated
A_32_P122	0.000501	8.09E-06	-3.43	LOC441455	makorin ring finger protein 1 pseudogene	Downregulated
A_22_P000	0.000521	0.0000102	-3.43	lnc-PDZD7-1	lnc-PDZD7-1:1	Downregulated
A_33_P332	0.000582	0.0000198	-3.43	PDE4DIP	phosphodiesterase 4D interacting protein	Downregulated
A_22_P000	0.001398	0.000158	-3.43	HOTAIRM1	HOXA transcript antisense RNA, myeloid-specific 1	Downregulated
A_21_P001	0.000483	4.85E-06	-3.44	LINC01420	long intergenic non-protein coding RNA 1420	Downregulated
A_21_P000	0.000483	5.16E-06	-3.44	lnc-FARP1-1	lnc-FARP1-1:2	Downregulated
A_23_P135	0.000558	0.0000154	-3.44	EIF2AK3	eukaryotic translation initiation factor 2-alpha kinase 3	Downregulated
A_33_P325	0.000577	0.0000182	-3.44	CNTRL	centriolin	Downregulated
A_33_P340	0.000883	0.0000591	-3.44	FLYWCH1	FLYWCH-type zinc finger 1	Downregulated
A_22_P000	0.000959	0.0000712	-3.44	lnc-APOM-3	lnc-APOM-3:1	Downregulated
A_23_P327	0.00307	0.000588	-3.44	C3orf38	chromosome 3 open reading frame 38	Downregulated
A_22_P000	0.003205	0.000632	-3.44	SCAMP1	secretory carrier membrane protein 1	Downregulated
A_33_P681	0.000578	0.0000191	-3.45	NDUFA6-AS1	NDUFA6 antisense RNA 1 (head to head)	Downregulated

A_24_P248	0.00076	0.0000417	-3.45	SYT11	synaptotagmin XI	Downregulated
A_24_P442	0.000971	0.0000744	-3.45	FBXW8	F-box and WD repeat domain containing 8	Downregulated
A_23_P349	0.00123	0.000125	-3.45	EVI5	ecotropic viral integration site 5	Downregulated
A_33_P335	0.001349	0.000148	-3.45	CTBP1	C-terminal binding protein 1	Downregulated
A_23_P210	0.002109	0.000329	-3.45	NSFL1C	NSFL1 (p97) cofactor (p47)	Downregulated
A_24_P193	0.000715	0.0000363	-3.46	DEF8	differentially expressed in FDCP 8 homolog (mouse)	Downregulated
A_33_P332	0.001688	0.000222	-3.46	NMS	neuromedin S	Downregulated
A_33_P353	0.002053	0.000315	-3.46	PRO2852	uncharacterized protein PRO2852	Downregulated
A_24_P405	0.002627	0.000464	-3.46	HOMEZ	homeobox and leucine zipper encoding	Downregulated
A_23_P877	0.002623	0.000462	-3.47	IFFO1	intermediate filament family orphan 1	Downregulated
A_33_P328	0.004142	0.000918	-3.47	MIER1	mesoderm induction early response 1, transcriptional regulator	Downregulated
A_24_P627	0.000534	0.0000136	-3.48	EPM2AIP1	EPM2A (laforin) interacting protein 1	Downregulated
A_23_P858	0.001563	0.000194	-3.48	GLMP	glycosylated lysosomal membrane protein	Downregulated
A_33_P341	0.004152	0.000921	-3.48	ZNF268	zinc finger protein 268	Downregulated
A_23_P377	0.000502	8.43E-06	-3.49	TNFRSF17	tumor necrosis factor receptor superfamily, member 17	Downregulated
A_24_P116	0.000563	0.0000168	-3.49	KLHDC2	kelch domain containing 2	Downregulated
A_33_P340	0.000938	0.0000675	-3.49	UBE3A	ubiquitin protein ligase E3A	Downregulated
A_19_P003	0.000942	0.0000683	-3.49	CA5BP1	carbonic anhydrase VB pseudogene 1	Downregulated
A_33_P324	0.000945	0.000069	-3.49	TMEM87A	transmembrane protein 87A	Downregulated
A_23_P145	0.002124	0.000335	-3.49	FIG4	FIG4 phosphoinositide 5-phosphatase	Downregulated
A_23_P106	0.000489	7.37E-06	-3.5	ORAI3	ORAI calcium release-activated calcium modulator 3	Downregulated
A_33_P322	0.000498	7.83E-06	-3.5	CSRNP1	cysteine-serine-rich nuclear protein 1	Downregulated
A_23_P116	0.000526	0.0000126	-3.5	PLA2G4A	phospholipase A2, group IVA (cytosolic, calcium-dependent)	Downregulated
A_33_P326	0.00056	0.0000156	-3.5	FAM167A	family with sequence similarity 167, member A	Downregulated
A_23_P445	0.002701	0.000483	-3.5	DFFB	DNA fragmentation factor, 40kDa, beta polypeptide (caspase-activated	Downregulated
A_33_P340	0.000592	0.0000208	-3.51	AKT2	v-akt murine thymoma viral oncogene homolog 2	Downregulated
A_22_P000	0.000622	0.0000243	-3.51	ARHGEF38	Rho guanine nucleotide exchange factor (GEF) 38	Downregulated
A_23_P409	0.000701	0.0000327	-3.51	NR2C2	nuclear receptor subfamily 2, group C, member 2	Downregulated
A_33_P332	0.001363	0.00015	-3.51	FANCL	Fanconi anemia, complementation group L	Downregulated
A_24_P141	0.001943	0.000284	-3.51	METAP2	methionyl aminopeptidase 2	Downregulated
A_22_P000	0.002832	0.000519	-3.51	lnc-SPTBN5-1	lnc-SPTBN5-1:1	Downregulated
A_22_P000	0.000578	0.0000189	-3.52	LOC1019276	uncharacterized LOC101927609	Downregulated
A_21_P000	0.000912	0.0000635	-3.52	SNORD92	small nucleolar RNA, C/D box 92	Downregulated
A_21_P000	0.00234	0.000388	-3.52	lnc-ANKRD27	lnc-ANKRD27-2:3	Downregulated
A_21_P001	0.002968	0.000559	-3.52	MMP25-AS1	MMP25 antisense RNA 1	Downregulated
A_22_P000	0.000483	6.01E-06	-3.53	TRG-AS1	T cell receptor gamma locus antisense RNA 1	Downregulated
A_23_P399	0.000995	0.0000783	-3.53	COA5	cytochrome c oxidase assembly factor 5	Downregulated

A_23_P137	0.001043	0.0000874	-3.53	BTK	Bruton agammaglobulinemia tyrosine kinase	Downregulated
A_33_P334	0.004301	0.000973	-3.53	KMT2D	lysine (K)-specific methyltransferase 2D	Downregulated
A_33_P332	0.000483	4.28E-06	-3.54	RHOT1	ras homolog family member T1	Downregulated
A_24_P356	0.000577	0.0000184	-3.54	MAP2K5	mitogen-activated protein kinase kinase 5	Downregulated
A_21_P000	0.000603	0.0000223	-3.54	lnc-HERPUD2	lnc-HERPUD2-1:1	Downregulated
A_21_P001	0.002168	0.000346	-3.54	XLOC_l2_002790		Downregulated
A_23_P139	0.001499	0.00018	-3.55	DHX37	DEAH (Asp-Glu-Ala-His) box polypeptide 37	Downregulated
A_22_P000	0.003909	0.000845	-3.55	LOC1027245	uncharacterized LOC102724532	Downregulated
A_21_P000	0.000905	0.0000619	-3.56	lnc-RNFT2-1	lnc-RNFT2-1:1	Downregulated
A_23_P303	0.000494	7.51E-06	-3.57	CHMP7	charged multivesicular body protein 7	Downregulated
A_33_P383	0.00056	0.000016	-3.57	RNU4ATAC	RNA, U4atac small nuclear (U12-dependent splicing)	Downregulated
A_23_P359	0.0015	0.000181	-3.57	AKAP2	A kinase (PRKA) anchor protein 2	Downregulated
A_33_P341	0.003266	0.000649	-3.57	ANKRD36B	ankyrin repeat domain 36B	Downregulated
A_23_P410	0.003509	0.00072	-3.57	RASSF1	Ras association (RalGDS/AF-6) domain family member 1	Downregulated
A_32_P112	0.000707	0.0000339	-3.58	LINC00999	long intergenic non-protein coding RNA 999	Downregulated
A_33_P324	0.00072	0.000037	-3.58	TMCO3	transmembrane and coiled-coil domains 3	Downregulated
A_22_P000	0.000483	6.35E-06	-3.59	LYST	lysosomal trafficking regulator	Downregulated
A_21_P000	0.000483	6.42E-06	-3.59	LOC1019297	uncharacterized LOC101929719	Downregulated
A_23_P778	0.000945	0.0000689	-3.59	FN3KRP	fructosamine 3 kinase related protein	Downregulated
A_23_P427	0.001178	0.000114	-3.59	LOC646652	integral membrane glycoprotein-like	Downregulated
A_23_P203	0.001443	0.000168	-3.59	DPYS	dihydropyrimidinase	Downregulated
A_22_P000	0.001925	0.00028	-3.59	lnc-PRR25-2	lnc-PRR25-2:1	Downregulated
A_23_P300	0.000642	0.0000262	-3.6	DDX60L	DEAD (Asp-Glu-Ala-Asp) box polypeptide 60-like	Downregulated
A_23_P169	0.000776	0.0000438	-3.6	EXOC6	exocyst complex component 6	Downregulated
A_24_P300	0.000788	0.0000457	-3.6	SCN9A	sodium channel, voltage gated, type IX alpha subunit	Downregulated
A_24_P745	0.001612	0.000205	-3.6	CYTH4	cytohesin 4	Downregulated
A_23_P205	0.000605	0.0000227	-3.61	DHRS12	dehydrogenase/reductase (SDR family) member 12	Downregulated
A_33_P365	0.000642	0.0000262	-3.61	SNORA26	small nucleolar RNA, H/ACA box 26	Downregulated
A_23_P935	0.000707	0.0000337	-3.61	SAMD3	sterile alpha motif domain containing 3	Downregulated
A_21_P000	0.000866	0.000057	-3.61	lnc-PHACTR1	lnc-PHACTR1-1:1	Downregulated
A_33_P341	0.001287	0.000136	-3.61	lnc-VIM-1	lnc-VIM-1:2	Downregulated
A_23_P404	0.001559	0.000193	-3.61	ARHGAP33	Rho GTPase activating protein 33	Downregulated
A_23_P368	0.003646	0.000762	-3.61	TMEM131	transmembrane protein 131	Downregulated
A_23_P131	0.004198	0.000937	-3.61	SNAI1	snail family zinc finger 1	Downregulated
A_19_P003	0.000812	0.0000494	-3.62	LINC01094	long intergenic non-protein coding RNA 1094	Downregulated
A_21_P001	0.001597	0.000201	-3.62	XLOC_l2_006173		Downregulated
A_23_P105	0.005153	0.00127	-3.62	POC1B	POC1 centriolar protein B	Downregulated

A_24_P235	0.00055	0.000015	-3.63	ZNF706	zinc finger protein 706	Downregulated
A_24_P382	0.00056	0.0000158	-3.63	RPRD1A	regulation of nuclear pre-mRNA domain containing 1A	Downregulated
A_22_P000	0.000603	0.0000221	-3.63	LOC1027238	uncharacterized LOC102723885	Downregulated
A_23_P161	0.000849	0.0000545	-3.63	TMEM134	transmembrane protein 134	Downregulated
A_23_P258	0.000925	0.0000652	-3.63	PPM1M	protein phosphatase, Mg2+/Mn2+ dependent, 1M	Downregulated
A_33_P335	0.0011	0.0000992	-3.63	LOC1001309	uncharacterized LOC100130913	Downregulated
A_33_P328	0.001117	0.000103	-3.63	CRAT	carnitine O-acetyltransferase	Downregulated
A_24_P379	0.000483	0.0000046	-3.64	CHCHD5	coiled-coil-helix-coiled-coil-helix domain containing 5	Downregulated
A_22_P000	0.001064	0.0000915	-3.64	ERICH2	glutamate-rich 2	Downregulated
A_23_P110	0.000483	4.09E-06	-3.65	WDR53	WD repeat domain 53	Downregulated
A_21_P000	0.000537	0.0000142	-3.65	SNORD63	small nucleolar RNA, C/D box 63	Downregulated
A_23_P615	0.000715	0.0000363	-3.65	CCDC71	coiled-coil domain containing 71	Downregulated
A_23_P884	0.002389	0.000401	-3.65	FOXN3	forkhead box N3	Downregulated
A_22_P000	0.000483	0.000004	-3.66	lnc-WRAP73-:	lnc-WRAP73-2:1	Downregulated
A_23_P420	0.000544	0.0000147	-3.66	BPHL	biphenyl hydrolase-like (serine hydrolase)	Downregulated
A_23_P136	0.000663	0.000028	-3.66	ZBTB39	zinc finger and BTB domain containing 39	Downregulated
A_33_P329	0.000695	0.0000316	-3.66	RPL28	ribosomal protein L28	Downregulated
A_33_P339	0.000775	0.0000436	-3.66	AKAP5	A kinase (PRKA) anchor protein 5	Downregulated
A_33_P359	0.001758	0.000239	-3.66	FAM91A1	family with sequence similarity 91, member A1	Downregulated
A_24_P818	0.000483	4.23E-06	-3.67	CDKN1B	cyclin-dependent kinase inhibitor 1B (p27, Kip1)	Downregulated
A_32_P140	0.00088	0.0000586	-3.67	F13A1	coagulation factor XIII, A1 polypeptide	Downregulated
A_22_P000	0.001009	0.0000815	-3.67	lnc-MIER3-2	lnc-MIER3-2:1	Downregulated
A_23_P252	0.000483	4.79E-06	-3.68	WBP1	WW domain binding protein 1	Downregulated
A_33_P325	0.000521	0.0000105	-3.68	ZNF551	zinc finger protein 551	Downregulated
A_23_P204	0.001427	0.000164	-3.68	ITFG2	integrin alpha FG-GAP repeat containing 2	Downregulated
A_21_P000	0.001998	0.0003	-3.68	lnc-RBM10-1	lnc-RBM10-1:1	Downregulated
A_23_P597	0.000483	6.36E-06	-3.69	SRI	sorcin	Downregulated
A_21_P000	0.001006	0.0000809	-3.69	LINC01578	long intergenic non-protein coding RNA 1578	Downregulated
A_33_P339	0.001035	0.0000857	-3.69	LINC01420	long intergenic non-protein coding RNA 1420	Downregulated
A_33_P336	0.00111	0.000101	-3.69	FRS2	fibroblast growth factor receptor substrate 2	Downregulated
A_22_P000	0.001956	0.000288	-3.69	LOC1019274	uncharacterized LOC101927418	Downregulated
A_33_P338	0.003669	0.00077	-3.69	TEX9	testis expressed 9	Downregulated
A_21_P001	0.005534	0.00141	-3.7	XLOC_l2_009448		Downregulated
A_23_P364	0.000483	0.0000044	-3.71	TXLNGY	taxilin gamma pseudogene, Y-linked	Downregulated
A_21_P001	0.000697	0.000032	-3.71	UBE2Q2P1	ubiquitin-conjugating enzyme E2Q family member 2 pseudogene 1	Downregulated
A_24_P927	0.001086	0.0000961	-3.71	SLED1	proteoglycan 3 pseudogene	Downregulated
A_33_P342	0.00056	0.0000163	-3.73	UVSSA	UV-stimulated scaffold protein A	Downregulated

A_33_P323	0.000532	0.0000134	-3.74	GOLGA6L9	golgin A6 family-like 9	Downregulated
A_23_P205	0.00104	0.0000864	-3.74	PDS5B	PDS5 cohesin associated factor B	Downregulated
A_23_P250	0.002326	0.000385	-3.74	CAND2	cullin-associated and neddylation-dissociated 2 (putative)	Downregulated
A_33_P328	0.000515	0.0000093	-3.75	ARF3	ADP-ribosylation factor 3	Downregulated
A_23_P541	0.00072	0.0000372	-3.75	MED6	mediator complex subunit 6	Downregulated
A_21_P000	0.001108	0.000101	-3.75	lnc-USP8-2	lnc-USP8-2:8	Downregulated
A_23_P259	0.001432	0.000165	-3.75	NCOA6	nuclear receptor coactivator 6	Downregulated
A_22_P000	0.001755	0.000238	-3.75	lnc-CHAC1-4	lnc-CHAC1-4:1	Downregulated
A_21_P000	0.001021	0.0000832	-3.76	lnc-DIRAS2-1	lnc-DIRAS2-1:4	Downregulated
A_22_P000	0.004207	0.000941	-3.76	lnc-SUSD1-2	lnc-SUSD1-2:1	Downregulated
A_21_P001	0.004217	0.000945	-3.76	XLOC_l2_010511		Downregulated
A_33_P338	0.000483	4.45E-06	-3.77	USP9X	ubiquitin specific peptidase 9, X-linked	Downregulated
A_33_P328	0.000707	0.0000346	-3.77	AP1S3	adaptor-related protein complex 1, sigma 3 subunit	Downregulated
A_21_P000	0.000806	0.0000485	-3.77	LOC1005057	uncharacterized LOC100505716	Downregulated
A_23_P110	0.001064	0.0000916	-3.77	METTL14	methyltransferase like 14	Downregulated
A_23_P217	0.000483	3.89E-06	-3.78	VPS13A	vacuolar protein sorting 13 homolog A (S. cerevisiae)	Downregulated
A_33_P331	0.000938	0.0000672	-3.78	BBIP1	BBSome interacting protein 1	Downregulated
A_21_P000	0.001161	0.000111	-3.78	lnc-APITD1-1	lnc-APITD1-1:1	Downregulated
A_23_P432	0.001154	0.00011	-3.8	CCDC125	coiled-coil domain containing 125	Downregulated
A_23_P183	0.001385	0.000154	-3.8	ARMC8	armadillo repeat containing 8	Downregulated
A_24_P449	0.000527	0.0000129	-3.81	MPND	MPN domain containing	Downregulated
A_23_P411	0.000706	0.0000334	-3.81	APEH	acylaminoacyl-peptide hydrolase	Downregulated
A_22_P000	0.000575	0.0000175	-3.82	CNPY3	canopy FGF signaling regulator 3	Downregulated
A_23_P363	0.001845	0.000261	-3.82	INPPL1	inositol polyphosphate phosphatase-like 1	Downregulated
A_23_P157	0.001863	0.000265	-3.82	BZW2	basic leucine zipper and W2 domains 2	Downregulated
A_23_P250	0.00275	0.000497	-3.82	HDAC6	histone deacetylase 6	Downregulated
A_22_P000	0.000697	0.0000318	-3.83	lnc-ZNF131-2	lnc-ZNF131-2:2	Downregulated
A_21_P000	0.000534	0.0000136	-3.84	SNORA45B	small nucleolar RNA, H/ACA box 45B	Downregulated
A_21_P000	0.000589	0.0000205	-3.84	SNORD36C	small nucleolar RNA, C/D box 36C	Downregulated
A_21_P000	0.000839	0.0000531	-3.84	lnc-SEL1L3-1	lnc-SEL1L3-1:3	Downregulated
A_33_P335	0.00119	0.000117	-3.84	OSBPL7	oxysterol binding protein-like 7	Downregulated
A_23_P134	0.001375	0.000153	-3.84	USP45	ubiquitin specific peptidase 45	Downregulated
A_33_P341	0.002393	0.000402	-3.84	EGOT	eosinophil granule ontogeny transcript (non-protein coding)	Downregulated
A_21_P001	0.000689	0.0000303	-3.85	lnc-TPTE2-2	lnc-TPTE2-2:1	Downregulated
A_21_P000	0.000842	0.0000534	-3.85	lnc-MEF2D-1	lnc-MEF2D-1:1	Downregulated
A_33_P327	0.002055	0.000316	-3.85	DIS3L2	DIS3 like 3'-5' exoribonuclease 2	Downregulated
A_23_P958	0.000483	3.63E-06	-3.86	TUFM	Tu translation elongation factor, mitochondrial	Downregulated

A_22_P000	0.000483	5.13E-06	-3.86	lnc-PMEL-1	lnc-PMEL-1:1	Downregulated
A_33_P337	0.000799	0.0000474	-3.86	CACUL1	CDK2-associated, cullin domain 1	Downregulated
A_23_P202	0.000483	4.74E-06	-3.87	SFXN3	sideroflexin 3	Downregulated
A_22_P000	0.000515	0.0000096	-3.87	lnc-OPN4-1	lnc-OPN4-1:2	Downregulated
A_24_P359	0.00071	0.0000353	-3.87	GPATCH8	G patch domain containing 8	Downregulated
A_22_P000	0.001101	0.0000996	-3.87	BLCAP	bladder cancer associated protein	Downregulated
A_23_P251	0.00121	0.000121	-3.87	RFESD	Rieske (Fe-S) domain containing	Downregulated
A_33_P329	0.000486	6.58E-06	-3.89	SART1	squamous cell carcinoma antigen recognized by T cells	Downregulated
A_24_P106	0.000525	0.0000114	-3.89	SLC44A2	solute carrier family 44 (choline transporter), member 2	Downregulated
A_21_P000	0.000515	9.64E-06	-3.9	SNORD59B	small nucleolar RNA, C/D box 59B	Downregulated
A_33_P330	0.00056	0.0000163	-3.9	PTPRS	protein tyrosine phosphatase, receptor type, S	Downregulated
A_24_P191	0.000592	0.0000207	-3.9	SAP30L	SAP30-like	Downregulated
A_23_P145	0.003854	0.000829	-3.9	ACOT4	acyl-CoA thioesterase 4	Downregulated
A_24_P130	0.000521	0.0000107	-3.91	NSUN4	NOP2/Sun domain family, member 4	Downregulated
A_23_P140	0.000549	0.0000149	-3.91	AKAP3	A kinase (PRKA) anchor protein 3	Downregulated
A_23_P119	0.00056	0.000016	-3.91	SLC6A9	solute carrier family 6 (neurotransmitter transporter, glycine), member 9	Downregulated
A_23_P203	0.000597	0.0000213	-3.91	CREBZF	CREB/ATF bZIP transcription factor	Downregulated
A_24_P174	0.000812	0.0000493	-3.91	SLC22A5	solute carrier family 22 (organic cation/carnitine transporter), member 5	Downregulated
A_23_P215	0.000537	0.0000139	-3.92	FKBP14	FK506 binding protein 14, 22 kDa	Downregulated
A_33_P335	0.000543	0.0000146	-3.92	FBXO22-AS1	FBXO22 antisense RNA 1	Downregulated
A_23_P290	0.00072	0.0000372	-3.92	CBR1	carbonyl reductase 1	Downregulated
A_33_P379	0.002333	0.000386	-3.92	LOC1027236	uncharacterized LOC102723652	Downregulated
A_21_P001	0.000839	0.000053	-3.93	XLOC_l2_014331		Downregulated
A_32_P284	0.000483	0.0000041	-3.94	LRRC37BP1	leucine rich repeat containing 37B pseudogene 1	Downregulated
A_33_P330	0.000483	4.48E-06	-3.94	TLE4	transducin-like enhancer of split 4	Downregulated
A_22_P000	0.001653	0.000214	-3.94	lnc-GTDC1-3	lnc-GTDC1-3:1	Downregulated
A_23_P436	0.000525	0.0000115	-3.95	SLC25A42	solute carrier family 25, member 42	Downregulated
A_33_P338	0.002951	0.000553	-3.95	LCK	LCK proto-oncogene, Src family tyrosine kinase	Downregulated
A_33_P333	0.00058	0.0000196	-3.96	NUDT17	nudix (nucleoside diphosphate linked moiety X)-type motif 17	Downregulated
A_23_P146	0.00124	0.000127	-3.96	NIPAL2	NIPA-like domain containing 2	Downregulated
A_21_P000	0.000483	4.32E-06	-3.97	SNORD12C	small nucleolar RNA, C/D box 12C	Downregulated
A_21_P001	0.000731	0.0000383	-3.97	LOC1019277	uncharacterized LOC101927752	Downregulated
A_23_P417	0.001049	0.0000885	-3.97	TSPYL5	TSPY-like 5	Downregulated
A_23_P162	0.001076	0.0000943	-3.97	HECTD4	HECT domain containing E3 ubiquitin protein ligase 4	Downregulated
A_19_P003	0.002196	0.000353	-3.97	CDYL2	chromodomain protein, Y-like 2	Downregulated
A_33_P340	0.000526	0.0000117	-3.98	POTEM	POTE ankyrin domain family, member M	Downregulated
A_23_P215	0.000526	0.0000128	-3.98	DUS4L	dihydrouridine synthase 4-like (S. cerevisiae)	Downregulated

A_23_P149	0.000534	0.0000136	-3.98	HIST2H2BE	histone cluster 2, H2be	Downregulated
A_22_P000	0.000577	0.0000179	-3.98	lnc-ZNF382-1	lnc-ZNF382-1:1	Downregulated
A_23_P354	0.001404	0.00016	-3.98	CHTF18	CTF18, chromosome transmission fidelity factor 18 homolog (S. cerevisia	Downregulated
A_24_P273	0.003795	0.000809	-3.98	TP63	tumor protein p63	Downregulated
A_33_P335	0.000543	0.0000146	-3.99	PVT1	Pvt1 oncogene (non-protein coding)	Downregulated
A_21_P000	0.001195	0.000118	-3.99	NBPF15	neuroblastoma breakpoint family, member 15	Downregulated
A_33_P339	0.002727	0.000491	-3.99	SNX22	sorting nexin 22	Downregulated
A_32_P462	0.001847	0.000261	-4	SLC9A9	solute carrier family 9, subfamily A (NHE9, cation proton antiporter 9), m	Downregulated
A_22_P000	0.002957	0.000555	-4	lnc-C7orf13-1	lnc-C7orf13-1:1	Downregulated
A_23_P348	0.000483	3.19E-06	-4.01	TCEANC2	transcription elongation factor A (SII) N-terminal and central domain con	Downregulated
A_33_P337	0.001107	0.0001	-4.01	BTN1A1	butyrophilin, subfamily 1, member A1	Downregulated
A_33_P333	0.000483	3.14E-06	-4.02	SLC44A2	solute carrier family 44 (choline transporter), member 2	Downregulated
A_23_P516	0.000995	0.0000791	-4.02	MUTYH	mutY homolog	Downregulated
A_33_P341	0.000483	2.91E-06	-4.03	CLCC1	chloride channel CLIC-like 1	Downregulated
A_33_P332	0.000589	0.0000204	-4.04	SMIM12	small integral membrane protein 12	Downregulated
A_33_P337	0.000707	0.0000337	-4.04	PAPD5	PAP associated domain containing 5	Downregulated
A_23_P129	0.000707	0.0000337	-4.04	VASN	vasorin	Downregulated
A_23_P137	0.001099	0.0000989	-4.04	IGSF8	immunoglobulin superfamily, member 8	Downregulated
A_22_P000	0.001406	0.00016	-4.04	lnc-ZNF687-1	lnc-ZNF687-1:1	Downregulated
A_22_P000	0.004121	0.000911	-4.04	lnc-MFSD4-1	lnc-MFSD4-1:1	Downregulated
A_22_P000	0.000537	0.000014	-4.05	FAM184B	family with sequence similarity 184, member B	Downregulated
A_24_P123	0.000778	0.0000442	-4.05	SEC63	SEC63 homolog (S. cerevisiae)	Downregulated
A_22_P000	0.000526	0.0000122	-4.06	LIMD1-AS1	LIMD1 antisense RNA 1	Downregulated
A_32_P206	0.00056	0.0000156	-4.06	QSOX2	quiescin Q6 sulfhydryl oxidase 2	Downregulated
A_22_P000	0.000695	0.0000312	-4.06	lnc-MYL4-2	lnc-MYL4-2:1	Downregulated
A_23_P397	0.000483	4.78E-06	-4.07	GLS	glutaminase	Downregulated
A_22_P000	0.000515	9.48E-06	-4.07	SLFNL1-AS1	SLFNL1 antisense RNA 1	Downregulated
A_22_P000	0.000677	0.0000295	-4.07	lnc-STUB1-1	lnc-STUB1-1:1	Downregulated
A_33_P333	0.000483	2.42E-06	-4.08	SNX29	sorting nexin 29	Downregulated
A_32_P224	0.000483	3.82E-06	-4.08	SLC25A23	solute carrier family 25 (mitochondrial carrier; phosphate carrier), membe	Downregulated
A_21_P000	0.000483	5.49E-06	-4.08	ZBTB24	zinc finger and BTB domain containing 24	Downregulated
A_33_P329	0.000513	9.02E-06	-4.08	KCNQ1OT1	KCNQ1 opposite strand/antisense transcript 1 (non-protein coding)	Downregulated
A_33_P327	0.000779	0.0000443	-4.08	SIRT2	sirtuin 2	Downregulated
A_33_P350	0.000788	0.0000458	-4.08	LOC780529	uncharacterized LOC780529	Downregulated
A_22_P000	0.001009	0.0000815	-4.08	lnc-ERAP1-1	lnc-ERAP1-1:1	Downregulated
A_33_P341	0.001727	0.000232	-4.08	RAP2C-AS1	RAP2C antisense RNA 1	Downregulated
A_33_P323	0.000684	0.00003	-4.09	CHCHD6	coiled-coil-helix-coiled-coil-helix domain containing 6	Downregulated

A_33_P321	0.000987	0.0000765	-4.09	TLE1	transducin-like enhancer of split 1 (E(sp1) homolog, Drosophila)	Downregulated
A_22_P000	0.001213	0.000121	-4.09	lnc-CEP120-2	lnc-CEP120-2:1	Downregulated
A_23_P961	0.001658	0.000215	-4.09	C11orf80	chromosome 11 open reading frame 80	Downregulated
A_23_P843	0.000483	0.0000028	-4.1	SIGIRR	single immunoglobulin and toll-interleukin 1 receptor (TIR) domain	Downregulated
A_21_P000	0.000535	0.0000137	-4.1	lnc-PEAK1.1-	lnc-PEAK1.1-1:3	Downregulated
A_23_P406	0.000691	0.0000307	-4.1	SRPK2	SRSF protein kinase 2	Downregulated
A_21_P000	0.000499	0.000008	-4.11	PEAK1	pseudopodium-enriched atypical kinase 1	Downregulated
A_21_P000	0.000515	9.19E-06	-4.11	lnc-TAF1A-1	lnc-TAF1A-1:1	Downregulated
A_23_P999	0.000483	6.11E-06	-4.12	LRRK1	leucine-rich repeat kinase 1	Downregulated
A_33_P681	0.000521	0.0000107	-4.12	LOC1005068	uncharacterized LOC100506844	Downregulated
A_21_P001	0.000542	0.0000145	-4.12	LOC1019278	uncharacterized LOC101927811	Downregulated
A_23_P155	0.00093	0.0000661	-4.12	ABHD14B	abhydrolase domain containing 14B	Downregulated
A_23_P308	0.001443	0.000167	-4.13	MAP3K15	mitogen-activated protein kinase kinase kinase 15	Downregulated
A_24_P228	0.001597	0.000201	-4.13	CYB5D2	cytochrome b5 domain containing 2	Downregulated
A_22_P000	0.000537	0.0000139	-4.14	KDM4C	lysine (K)-specific demethylase 4C	Downregulated
A_23_P638	0.000596	0.000021	-4.14	CTBP2	C-terminal binding protein 2	Downregulated
A_23_P208	0.000602	0.0000219	-4.14	ZNF407	zinc finger protein 407	Downregulated
A_23_P514	0.000662	0.0000279	-4.14	GBP3	guanylate binding protein 3	Downregulated
A_23_P153	0.000788	0.0000457	-4.14	ARMC7	armadillo repeat containing 7	Downregulated
A_22_P000	0.002601	0.000457	-4.14	LINC00173	long intergenic non-protein coding RNA 173	Downregulated
A_21_P000	0.000483	2.27E-06	-4.15	lnc-PIGF-3	lnc-PIGF-3:1	Downregulated
A_23_P218	0.000521	0.0000107	-4.16	SERPINA1	serpin peptidase inhibitor, clade A (alpha-1 antiproteinase, antitrypsin),	Downregulated
A_33_P327	0.000577	0.0000185	-4.16	HSD17B7	hydroxysteroid (17-beta) dehydrogenase 7	Downregulated
A_22_P000	0.000486	6.73E-06	-4.17	lnc-ADAM19-	lnc-ADAM19-1:1	Downregulated
A_33_P330	0.000739	0.0000392	-4.17	BRCC3	BRCA1/BRCA2-containing complex, subunit 3	Downregulated
A_33_P330	0.000483	5.13E-06	-4.18	PSMD5	proteasome (prosome, macropain) 26S subunit, non-ATPase, 5	Downregulated
A_23_P119	0.000483	5.79E-06	-4.18	KLF2	Kruppel-like factor 2	Downregulated
A_24_P245	0.000485	6.54E-06	-4.18	TNFSF12	tumor necrosis factor (ligand) superfamily, member 12	Downregulated
A_23_P164	0.000526	0.0000118	-4.18	CDC34	cell division cycle 34	Downregulated
A_33_P333	0.000526	0.0000124	-4.18	ALDH3A2	aldehyde dehydrogenase 3 family, member A2	Downregulated
A_33_P341	0.000578	0.0000192	-4.18	DGCR11	DiGeorge syndrome critical region gene 11 (non-protein coding)	Downregulated
A_21_P000	0.000584	0.00002	-4.18	LOC1002870	uncharacterized LOC100287036	Downregulated
A_19_P008	0.000516	0.0000101	-4.19	TAPT1-AS1	TAPT1 antisense RNA 1 (head to head)	Downregulated
A_23_P937	0.000765	0.0000424	-4.19	PMS2	PMS2 postmeiotic segregation increased 2 (S. cerevisiae)	Downregulated
A_23_P250	0.00129	0.000136	-4.19	HSD17B6	hydroxysteroid (17-beta) dehydrogenase 6	Downregulated
A_23_P217	0.000486	6.89E-06	-4.2	CCNL2	cyclin L2	Downregulated
A_22_P000	0.000525	0.0000114	-4.2	LOC1020313	uncharacterized LOC102031319	Downregulated

A_23_P612	0.000483	4.11E-06	-4.21	HGH1	HGH1 homolog (S. cerevisiae)	Downregulated
A_33_P344	0.000498	7.77E-06	-4.21	LOC389641	uncharacterized LOC389641	Downregulated
A_24_P297	0.001702	0.000227	-4.21	CDK14	cyclin-dependent kinase 14	Downregulated
A_23_P112	0.003387	0.000683	-4.22	DGAT1	diacylglycerol O-acyltransferase 1	Downregulated
A_24_P696	0.000501	8.22E-06	-4.23	ZNF25	zinc finger protein 25	Downregulated
A_23_P584	0.000578	0.0000191	-4.23	ANKHD1-EIF4	ANKHD1-EIF4EBP3 readthrough	Downregulated
A_21_P000	0.000578	0.0000191	-4.23	lnc-CHD1L-1	lnc-CHD1L-1:3	Downregulated
A_32_P214	0.00072	0.0000372	-4.23	lnc-AKIRIN1-1	lnc-AKIRIN1-1:2	Downregulated
A_32_P119	0.00056	0.0000161	-4.24	TPM3	tropomyosin 3	Downregulated
A_23_P169	0.000909	0.000063	-4.24	DGKI	diacylglycerol kinase, iota	Downregulated
A_23_P441	0.001141	0.000108	-4.24	DCXR	dicarbonyl/L-xylulose reductase	Downregulated
A_23_P152	0.000483	2.64E-06	-4.25	NUP88	nucleoporin 88kDa	Downregulated
A_33_P320	0.000515	9.83E-06	-4.25	WDFY2	WD repeat and FYVE domain containing 2	Downregulated
A_23_P211	0.000498	7.96E-06	-4.26	SETD4	SET domain containing 4	Downregulated
A_21_P001	0.000511	8.77E-06	-4.27	LOC1027251	uncharacterized LOC102725134	Downregulated
A_23_P419	0.000718	0.0000368	-4.27	KIAA1407	KIAA1407	Downregulated
A_33_P324	0.000801	0.0000478	-4.27	KIF13B	kinesin family member 13B	Downregulated
A_23_P653	0.000483	3.05E-06	-4.28	SYNJ2BP	synaptojanin 2 binding protein	Downregulated
A_22_P000	0.000521	0.0000104	-4.28	lnc-CAST-2	lnc-CAST-2:1	Downregulated
A_32_P159	0.000541	0.0000144	-4.28	KAT2B	K(lysine) acetyltransferase 2B	Downregulated
A_23_P105	0.000585	0.0000201	-4.28	HRASLS2	HRAS-like suppressor 2	Downregulated
A_24_P749	0.003099	0.000598	-4.28	KDM1A	lysine (K)-specific demethylase 1A	Downregulated
A_22_P000	0.001734	0.000233	-4.29	lnc-CERK-1	lnc-CERK-1:1	Downregulated
A_33_P337	0.000483	2.36E-06	-4.3	ABCC10	ATP-binding cassette, sub-family C (CFTR/MRP), member 10	Downregulated
A_21_P001	0.001602	0.000201	-4.3	NUBP1	nucleotide binding protein 1	Downregulated
A_22_P000	0.000483	4.11E-06	-4.31	lnc-TFEC-2	lnc-TFEC-2:4	Downregulated
A_33_P334	0.000521	0.0000102	-4.31	UBE4B	ubiquitination factor E4B	Downregulated
A_23_P890	0.000525	0.0000114	-4.33	C16orf95	chromosome 16 open reading frame 95	Downregulated
A_24_P244	0.000532	0.0000134	-4.33	NLRX1	NLR family member X1	Downregulated
A_33_P335	0.00096	0.0000716	-4.33	CD99	CD99 molecule	Downregulated
A_23_P916	0.000501	8.18E-06	-4.34	EWSR1	EWS RNA-binding protein 1	Downregulated
A_33_P341	0.000494	7.53E-06	-4.35	ALS2	amyotrophic lateral sclerosis 2 (juvenile)	Downregulated
A_24_P168	0.000521	0.0000107	-4.35	ZMYM3	zinc finger, MYM-type 3	Downregulated
A_23_P119	0.000532	0.0000133	-4.35	NUDT5	nudix (nucleoside diphosphate linked moiety X)-type motif 5	Downregulated
A_33_P328	0.000621	0.0000243	-4.35	FAM207A	family with sequence similarity 207, member A	Downregulated
A_22_P000	0.000486	0.000007	-4.36	lnc-SPINK9-1	lnc-SPINK9-1:1	Downregulated
A_21_P000	0.000605	0.0000226	-4.36	BTN2A1	butyrophilin, subfamily 2, member A1	Downregulated

A_19_P003	0.001084	0.0000956	-4.36	PVT1	Pvt1 oncogene (non-protein coding)	Downregulated
A_21_P001	0.000486	6.82E-06	-4.37	LOC1005074	uncharacterized LOC100507412	Downregulated
A_21_P001	0.000483	3.33E-06	-4.38	XLOC_l2_006025		Downregulated
A_24_P627	0.001263	0.000132	-4.38	EDEM2	ER degradation enhancer, mannosidase alpha-like 2	Downregulated
A_33_P332	0.000525	0.000011	-4.39	ARID5B	AT rich interactive domain 5B (MRF1-like)	Downregulated
A_23_P110	0.000634	0.0000253	-4.39	NICN1	nicolin 1	Downregulated
A_23_P125	0.000939	0.000068	-4.39	RBM10	RNA binding motif protein 10	Downregulated
A_22_P000	0.002949	0.000552	-4.39	LOC1019269	uncharacterized LOC101926933	Downregulated
A_22_P000	0.000483	3.45E-06	-4.4	lnc-TM2D2-3	lnc-TM2D2-3:1	Downregulated
A_24_P681	0.000526	0.0000128	-4.4	HIPK2	homeodomain interacting protein kinase 2	Downregulated
A_23_P486	0.000928	0.0000656	-4.4	PYGL	phosphorylase, glycogen, liver	Downregulated
A_22_P000	0.000483	5.95E-06	-4.41	ALG14	ALG14, UDP-N-acetylglucosaminyltransferase subunit	Downregulated
A_24_P181	0.000486	7.07E-06	-4.41	CATSPER2	cation channel, sperm associated 2	Downregulated
A_24_P275	0.000743	0.0000398	-4.41	PGAP3	post-GPI attachment to proteins 3	Downregulated
A_32_P105	0.000483	2.26E-06	-4.42	FTCDNL1	formiminotransferase cyclodeaminase N-terminal like	Downregulated
A_23_P678	0.000502	8.33E-06	-4.42	SCN3A	sodium channel, voltage gated, type III alpha subunit	Downregulated
A_23_P840	0.000995	0.0000779	-4.42	CDK5	cyclin-dependent kinase 5	Downregulated
A_23_P897	0.001297	0.000138	-4.43	CEP192	centrosomal protein 192kDa	Downregulated
A_23_P157	0.000483	5.49E-06	-4.44	DDHD2	DDHD domain containing 2	Downregulated
A_23_P160	0.000701	0.0000325	-4.44	AKT3	v-akt murine thymoma viral oncogene homolog 3	Downregulated
A_23_P254	0.003126	0.000607	-4.44	SGSH	N-sulfoglucosamine sulfohydrolase	Downregulated
A_33_P331	0.00056	0.0000156	-4.45	CRACR2A	calcium release activated channel regulator 2A	Downregulated
A_33_P325	0.000483	6.04E-06	-4.46	JADE1	jade family PHD finger 1	Downregulated
A_23_P124	0.000515	9.59E-06	-4.47	RGS14	regulator of G-protein signaling 14	Downregulated
A_24_P145	0.000526	0.0000122	-4.47	ZNF609	zinc finger protein 609	Downregulated
A_32_P121	0.00067	0.0000287	-4.49	ANAPC1	anaphase promoting complex subunit 1	Downregulated
A_23_P470	0.000711	0.0000355	-4.49	HHEX	hematopoietically expressed homeobox	Downregulated
A_22_P000	0.000991	0.0000772	-4.5	lnc-HSP90AA	lnc-HSP90AA1-4:1	Downregulated
A_24_P945	0.00221	0.000356	-4.5	CHMP3	charged multivesicular body protein 3	Downregulated
A_22_P000	0.000483	2.04E-06	-4.51	lnc-NT5E-1	lnc-NT5E-1:1	Downregulated
A_21_P000	0.000584	0.00002	-4.51	SNORD35B	small nucleolar RNA, C/D box 35B	Downregulated
A_21_P000	0.000609	0.000023	-4.51	SNORD56B	small nucleolar RNA, C/D box 56B	Downregulated
A_21_P000	0.001021	0.0000831	-4.51	LOC115110	uncharacterized LOC115110	Downregulated
A_33_P339	0.000483	2.17E-06	-4.52	BET1	Bet1 golgi vesicular membrane trafficking protein	Downregulated
A_24_P106	0.000483	1.65E-06	-4.53	PKD2	polycystic kidney disease 2 (autosomal dominant)	Downregulated
A_23_P590	0.000498	7.86E-06	-4.53	TRERF1	transcriptional regulating factor 1	Downregulated
A_23_P246	0.000707	0.0000344	-4.53	ELP4	elongator acetyltransferase complex subunit 4	Downregulated

A_33_P329	0.000523	0.0000109	-4.54	ZBTB20	zinc finger and BTB domain containing 20	Downregulated
A_33_P337	0.000537	0.0000141	-4.54	OSTN	osteocrin	Downregulated
A_23_P319	0.000483	1.63E-06	-4.55	FAM118B	family with sequence similarity 118, member B	Downregulated
A_33_P336	0.000532	0.0000133	-4.55	PHF8	PHD finger protein 8	Downregulated
A_24_P232	0.000577	0.0000184	-4.56	GRAMD4	GRAM domain containing 4	Downregulated
A_33_P328	0.002167	0.000346	-4.56	TSC2	tuberous sclerosis 2	Downregulated
A_23_P413	0.000483	6.35E-06	-4.57	SH3TC1	SH3 domain and tetratricopeptide repeats 1	Downregulated
A_23_P249	0.000664	0.0000282	-4.57	TSPAN31	tetraspanin 31	Downregulated
A_23_P156	0.000728	0.000038	-4.57	GZMK	granzyme K (granzyme 3; tryptase II)	Downregulated
A_33_P332	0.000999	0.00008	-4.57	MOSPD3	motile sperm domain containing 3	Downregulated
A_23_P386	0.00093	0.0000662	-4.6	INAFM1	InaF-motif containing 1	Downregulated
A_22_P000	0.002169	0.000347	-4.6	lnc-NKX6-3-1	lnc-NKX6-3-1:1	Downregulated
A_33_P333	0.000513	9.05E-06	-4.61	LOC728392	uncharacterized LOC728392	Downregulated
A_33_P330	0.000532	0.0000133	-4.61	LAMA4	laminin, alpha 4	Downregulated
A_22_P000	0.000999	0.00008	-4.61	lnc-GGCT-1	lnc-GGCT-1:4	Downregulated
A_23_P140	0.000483	1.54E-06	-4.62	EVL	Enah/Vasp-like	Downregulated
A_21_P001	0.000773	0.0000434	-4.62	ULK4P3	ULK4 pseudogene 3	Downregulated
A_33_P335	0.000513	8.85E-06	-4.63	TANGO6	transport and golgi organization 6 homolog (Drosophila)	Downregulated
A_33_P336	0.000483	5.48E-06	-4.64	ICAM4	intercellular adhesion molecule 4 (Landsteiner-Wiener blood group)	Downregulated
A_33_P335	0.00056	0.000016	-4.65	PATL2	protein associated with topoisomerase II homolog 2 (yeast)	Downregulated
A_33_P338	0.000483	6.08E-06	-4.66	BCAS3	breast carcinoma amplified sequence 3	Downregulated
A_21_P001	0.000578	0.0000192	-4.66	LOC1005071	uncharacterized LOC100507195	Downregulated
A_23_P243	0.000489	7.39E-06	-4.67	CCDC88B	coiled-coil domain containing 88B	Downregulated
A_33_P331	0.000483	6.35E-06	-4.68	RAB37	RAB37, member RAS oncogene family	Downregulated
A_23_P404	0.000939	0.0000679	-4.68	BIK	BCL2-interacting killer (apoptosis-inducing)	Downregulated
A_19_P003	0.000485	6.49E-06	-4.69	LINC00673	long intergenic non-protein coding RNA 673	Downregulated
A_21_P000	0.000639	0.0000257	-4.69	OVAAL	ovarian adenocarcinoma amplified long non-coding RNA	Downregulated
A_23_P126	0.000483	3.81E-06	-4.7	ANGPTL1	angiopoietin-like 1	Downregulated
A_33_P334	0.000501	8.13E-06	-4.7	CALHM2	calcium homeostasis modulator 2	Downregulated
A_21_P000	0.000515	9.58E-06	-4.7	SNORD12B	small nucleolar RNA, C/D box 12B	Downregulated
A_23_P121	0.000577	0.0000183	-4.7	TNFSF10	tumor necrosis factor (ligand) superfamily, member 10	Downregulated
A_33_P334	0.000695	0.0000311	-4.7	TTLL1	tubulin tyrosine ligase-like family member 1	Downregulated
A_23_P352	0.000964	0.000073	-4.7	DCAF5	DDB1 and CUL4 associated factor 5	Downregulated
A_19_P008	0.000486	6.75E-06	-4.72	lnc-RPRML-3	lnc-RPRML-3:2	Downregulated
A_23_P822	0.000571	0.0000173	-4.72	GNB2	guanine nucleotide binding protein (G protein), beta polypeptide 2	Downregulated
A_33_P338	0.001071	0.0000938	-4.72	DHX30	DEAH (Asp-Glu-Ala-His) box helicase 30	Downregulated
A_23_P150	0.000483	2.46E-06	-4.73	ESYT1	extended synaptotagmin-like protein 1	Downregulated

A_23_P208	0.000483	5.49E-06	-4.73	AXL	AXL receptor tyrosine kinase	Downregulated
A_22_P000	0.000483	5.51E-06	-4.73	LOC1019289	uncharacterized LOC101928915	Downregulated
A_23_P386	0.000683	0.0000299	-4.74	C4orf26	chromosome 4 open reading frame 26	Downregulated
A_23_P348	0.000526	0.0000118	-4.75	AK9	adenylate kinase 9	Downregulated
A_23_P864	0.000483	4.24E-06	-4.77	CH25H	cholesterol 25-hydroxylase	Downregulated
A_23_P210	0.001158	0.000111	-4.77	ELMO2	engulfment and cell motility 2	Downregulated
A_33_P329	0.002479	0.000423	-4.77	ZXDC	ZXD family zinc finger C	Downregulated
A_22_P000	0.000525	0.0000114	-4.79	LINC00421	long intergenic non-protein coding RNA 421	Downregulated
A_33_P349	0.000625	0.0000246	-4.79	CCDC141	coiled-coil domain containing 141	Downregulated
A_23_P101	0.000792	0.0000463	-4.79	KRI1	KRI1 homolog (S. cerevisiae)	Downregulated
A_23_P264	0.000938	0.0000672	-4.79	HBA2	hemoglobin, alpha 2	Downregulated
A_23_P551	0.000483	4.09E-06	-4.8	COX10	COX10 heme A:farnesyltransferase cytochrome c oxidase assembly fac	Downregulated
A_33_P322	0.000483	0.0000054	-4.8	SLC16A4	solute carrier family 16, member 4	Downregulated
A_23_P257	0.000483	5.94E-06	-4.81	ALG14	ALG14, UDP-N-acetylglucosaminyltransferase subunit	Downregulated
A_24_P135	0.000483	5.99E-06	-4.81	TSPAN14	tetraspanin 14	Downregulated
A_33_P325	0.00056	0.0000158	-4.81	PLIN2	perilipin 2	Downregulated
A_23_P215	0.000582	0.0000198	-4.81	ABCF2	ATP-binding cassette, sub-family F (GCN20), member 2	Downregulated
A_22_P000	0.000483	5.24E-06	-4.82	lnc-HYI-1	lnc-HYI-1:1	Downregulated
A_23_P146	0.00064	0.0000259	-4.82	RNF170	ring finger protein 170	Downregulated
A_23_P209	0.000697	0.0000321	-4.82	TRIM68	tripartite motif containing 68	Downregulated
A_33_P336	0.000483	4.43E-06	-4.85	UBA5	ubiquitin-like modifier activating enzyme 5	Downregulated
A_24_P854	0.000486	7.03E-06	-4.86	MIAT	myocardial infarction associated transcript (non-protein coding)	Downregulated
A_32_P135	0.000515	9.62E-06	-4.86	RBM41	RNA binding motif protein 41	Downregulated
A_24_P683	0.000532	0.0000132	-4.86	SYNE2	spectrin repeat containing, nuclear envelope 2	Downregulated
A_33_P325	0.000483	1.83E-06	-4.87	HBA2	hemoglobin, alpha 2	Downregulated
A_23_P137	0.000483	3.02E-06	-4.87	S100A10	S100 calcium binding protein A10	Downregulated
A_19_P003	0.000504	8.51E-06	-4.87	KIF9-AS1	KIF9 antisense RNA 1	Downregulated
A_33_P340	0.000537	0.0000139	-4.87	LRRC8C	leucine rich repeat containing 8 family, member C	Downregulated
A_22_P000	0.000608	0.000023	-4.87	ZNRD1-AS1	ZNRD1 antisense RNA 1	Downregulated
A_33_P336	0.002733	0.000492	-4.87	C6orf57	chromosome 6 open reading frame 57	Downregulated
A_21_P000	0.000483	3.86E-06	-4.9	LOC1027239	uncharacterized LOC102723931	Downregulated
A_23_P300	0.000709	0.000035	-4.9	SMCR8	Smith-Magenis syndrome chromosome region, candidate 8	Downregulated
A_24_P380	0.000486	6.98E-06	-4.91	SAPCD1	suppressor APC domain containing 1	Downregulated
A_23_P109	0.00124	0.000127	-4.91	THAP7	THAP domain containing 7	Downregulated
A_23_P345	0.000483	3.95E-06	-4.92	EPHX1	epoxide hydrolase 1, microsomal (xenobiotic)	Downregulated
A_24_P288	0.000483	4.55E-06	-4.92	DPP9	dipeptidyl-peptidase 9	Downregulated
A_24_P383	0.002102	0.000327	-4.92	C19orf70	chromosome 19 open reading frame 70	Downregulated

A_33_P326	0.000622	0.0000244	-4.93	EFCAB3	EF-hand calcium binding domain 3	Downregulated
A_33_P341	0.002853	0.000525	-4.93	CCDC124	coiled-coil domain containing 124	Downregulated
A_32_P158	0.000525	0.0000111	-4.94	KLRF1	killer cell lectin-like receptor subfamily F, member 1	Downregulated
A_22_P000	0.001364	0.000151	-4.96	lnc-DKK4-1	lnc-DKK4-1:1	Downregulated
A_33_P335	0.00056	0.0000156	-4.97	CLOCK	clock circadian regulator	Downregulated
A_21_P001	0.000483	1.18E-06	-4.99	XLOC_l2_010405		Downregulated
A_21_P000	0.000483	1.98E-06	-5.01	SNORD74	small nucleolar RNA, C/D box 74	Downregulated
A_21_P001	0.000483	5.13E-06	-5.01	LOC1019273	uncharacterized LOC101927365	Downregulated
A_23_P216	0.000578	0.0000193	-5.01	MAPKAP1	mitogen-activated protein kinase associated protein 1	Downregulated
A_33_P339	0.000596	0.0000211	-5.01	POLR3G	polymerase (RNA) III (DNA directed) polypeptide G (32kD)	Downregulated
A_22_P000	0.000483	6.16E-06	-5.02	lnc-HNMT-2	lnc-HNMT-2:1	Downregulated
A_33_P322	0.001069	0.0000934	-5.02	SLC4A8	solute carrier family 4, sodium bicarbonate cotransporter, member 8	Downregulated
A_21_P000	0.000525	0.0000109	-5.03	lnc-GPR183-2	lnc-GPR183-2:3	Downregulated
A_23_P387	0.000766	0.0000426	-5.04	ZBTB40	zinc finger and BTB domain containing 40	Downregulated
A_22_P000	0.000589	0.0000206	-5.06	lnc-NSMCE1-	lnc-NSMCE1-1:1	Downregulated
A_23_P666	0.002262	0.000371	-5.07	HOXB6	homeobox B6	Downregulated
A_24_P228	0.000589	0.0000205	-5.08	ARHGEF6	Rac/Cdc42 guanine nucleotide exchange factor (GEF) 6	Downregulated
A_33_P342	0.001141	0.000108	-5.08	TTL	tubulin tyrosine ligase	Downregulated
A_22_P000	0.002113	0.00033	-5.08	LOC1019284	uncharacterized LOC101928461	Downregulated
A_23_P105	0.000603	0.0000225	-5.09	GPRC5D	G protein-coupled receptor, class C, group 5, member D	Downregulated
A_33_P337	0.000515	9.33E-06	-5.1	SLC9A3R1	solute carrier family 9, subfamily A (NHE3, cation proton antiporter 3), m	Downregulated
A_23_P647	0.000611	0.0000232	-5.1	ZNF641	zinc finger protein 641	Downregulated
A_23_P482	0.000743	0.0000397	-5.13	APOLD1	apolipoprotein L domain containing 1	Downregulated
A_33_P335	0.002043	0.000312	-5.13	LOC728903	uncharacterized LOC728903	Downregulated
A_23_P874	0.00102	0.000083	-5.17	APOF	apolipoprotein F	Downregulated
A_23_P785	0.000515	9.91E-06	-5.18	CEACAM21	carcinoembryonic antigen-related cell adhesion molecule 21	Downregulated
A_23_P359	0.000483	4.25E-06	-5.19	PDDC1	Parkinson disease 7 domain containing 1	Downregulated
A_24_P941	0.000483	4.73E-06	-5.19	FAM65B	family with sequence similarity 65, member B	Downregulated
A_22_P000	0.002012	0.000304	-5.19	lnc-PLEKHA3	lnc-PLEKHA3-4:1	Downregulated
A_23_P667	0.000483	4.03E-06	-5.2	DHRS13	dehydrogenase/reductase (SDR family) member 13	Downregulated
A_22_P000	0.000575	0.0000177	-5.2	lnc-MKRN2-A	lnc-MKRN2-AS1-1:1	Downregulated
A_23_P388	0.000483	3.47E-06	-5.21	KAT6B	K(lysine) acetyltransferase 6B	Downregulated
A_22_P000	0.00155	0.000191	-5.22	LOC1019273	uncharacterized LOC101927391	Downregulated
A_22_P000	0.000483	4.16E-06	-5.23	LOC1019296	uncharacterized LOC101929648	Downregulated
A_33_P330	0.000526	0.0000119	-5.23	BLOC1S5	biogenesis of lysosomal organelles complex-1, subunit 5, muted	Downregulated
A_23_P122	0.000693	0.000031	-5.23	C6orf47	chromosome 6 open reading frame 47	Downregulated
A_21_P001	0.001404	0.00016	-5.23	MIR4435-1H	MIR4435-1 host gene (non-protein coding)	Downregulated

A_22_P000	0.002369	0.000396	-5.23	SNRPD1	small nuclear ribonucleoprotein D1 polypeptide 16kDa	Downregulated
A_24_P174	0.000537	0.0000142	-5.24	TTC30A	tetratricopeptide repeat domain 30A	Downregulated
A_23_P720	0.001626	0.000208	-5.24	SLC25A20	solute carrier family 25 (carnitine/acylcarnitine translocase), member 20	Downregulated
A_23_P152	0.001751	0.000236	-5.25	RMDN3	regulator of microtubule dynamics 3	Downregulated
A_23_P330	0.000605	0.0000227	-5.26	PIP5K1C	phosphatidylinositol-4-phosphate 5-kinase, type I, gamma	Downregulated
A_24_P337	0.000483	5.26E-06	-5.27	TTC7A	tetratricopeptide repeat domain 7A	Downregulated
A_23_P329	0.001108	0.000101	-5.28	DPH7	diphthamide biosynthesis 7	Downregulated
A_23_P537	0.000483	5.54E-06	-5.3	CLSTN3	calsyntenin 3	Downregulated
A_33_P324	0.000526	0.0000116	-5.3	ACSM6	acyl-CoA synthetase medium-chain family member 6	Downregulated
A_21_P000	0.000883	0.0000591	-5.32	LINC00944	long intergenic non-protein coding RNA 944	Downregulated
A_23_P592	0.000483	3.52E-06	-5.34	TAF11	TAF11 RNA polymerase II, TATA box binding protein (TBP)-associated f	Downregulated
A_24_P272	0.000486	6.85E-06	-5.34	ASH1L-AS1	ASH1L antisense RNA 1	Downregulated
A_22_P000	0.000483	4.05E-06	-5.35	lnc-RP11-467	lnc-RP11-467M13.1.1-3:1	Downregulated
A_33_P334	0.000483	3.51E-06	-5.37	LCLAT1	lysocardiolipin acyltransferase 1	Downregulated
A_33_P341	0.000483	3.58E-06	-5.37	MEN1	multiple endocrine neoplasia I	Downregulated
A_23_P430	0.000621	0.0000242	-5.37	LYSMD1	LysM, putative peptidoglycan-binding, domain containing 1	Downregulated
A_21_P000	0.000483	4.37E-06	-5.38	LOC1005071	uncharacterized LOC100507195	Downregulated
A_33_P338	0.000949	0.0000698	-5.38	ABLIM1	actin binding LIM protein 1	Downregulated
A_23_P490	0.001301	0.000139	-5.39	LPCAT4	lysophosphatidylcholine acyltransferase 4	Downregulated
A_23_P308	0.000483	5.01E-06	-5.41	AMER1	APC membrane recruitment protein 1	Downregulated
A_33_P337	0.000599	0.0000215	-5.44	SOCS2-AS1	SOCS2 antisense RNA 1	Downregulated
A_33_P338	0.000483	0.0000034	-5.45	STK10	serine/threonine kinase 10	Downregulated
A_23_P656	0.000483	3.93E-06	-5.46	SPG11	spastic paraplegia 11 (autosomal recessive)	Downregulated
A_23_P356	0.000483	0.0000061	-5.46	ZNF451	zinc finger protein 451	Downregulated
A_23_P397	0.001453	0.00017	-5.46	TIRAP	toll-interleukin 1 receptor (TIR) domain containing adaptor protein	Downregulated
A_33_P342	0.000483	0.0000025	-5.47	CRELD1	cysteine-rich with EGF-like domains 1	Downregulated
A_22_P000	0.000893	0.0000604	-5.47	LOC1019273	uncharacterized LOC101927355	Downregulated
A_22_P000	0.000483	0.0000044	-5.49	lnc-ANKRD42	lnc-ANKRD42-1:1	Downregulated
A_24_P119	0.000483	8.34E-07	-5.5	ITPKB	inositol-trisphosphate 3-kinase B	Downregulated
A_33_P339	0.000483	0.0000032	-5.5	C20orf27	chromosome 20 open reading frame 27	Downregulated
A_23_P214	0.000483	5.53E-06	-5.51	GPR63	G protein-coupled receptor 63	Downregulated
A_21_P000	0.000483	0.0000033	-5.52	LOC389641	uncharacterized LOC389641	Downregulated
A_23_P136	0.000483	1.01E-06	-5.53	AP3B1	adaptor-related protein complex 3, beta 1 subunit	Downregulated
A_23_P393	0.000483	0.0000048	-5.54	INPP4A	inositol polyphosphate-4-phosphatase, type I, 107kDa	Downregulated
A_33_P337	0.000483	5.03E-06	-5.54	SZT2	seizure threshold 2 homolog (mouse)	Downregulated
A_24_P916	0.00055	0.000015	-5.54	PRKCA	protein kinase C, alpha	Downregulated
A_22_P000	0.000483	2.63E-06	-5.55	LOC728730	uncharacterized LOC728730	Downregulated

A_33_P330	0.000537	0.0000138	-5.56	RIPK3	receptor-interacting serine-threonine kinase 3	Downregulated
A_24_P150	0.000597	0.0000214	-5.56	ZNF490	zinc finger protein 490	Downregulated
A_24_P222	0.000483	4.91E-06	-5.58	S100PBP	S100P binding protein	Downregulated
A_33_P338	0.000483	7.75E-07	-5.59	ATP6V1C1	ATPase, H+ transporting, lysosomal 42kDa, V1 subunit C1	Downregulated
A_23_P203	0.000483	4.16E-06	-5.61	IMMP1L	IMP1 inner mitochondrial membrane peptidase-like (S. cerevisiae)	Downregulated
A_23_P347	0.001698	0.000225	-5.65	KIF2C	kinesin family member 2C	Downregulated
A_33_P339	0.000483	5.46E-06	-5.68	CMTR1	cap methyltransferase 1	Downregulated
A_23_P338	0.000483	1.92E-06	-5.73	S100PBP	S100P binding protein	Downregulated
A_23_P377	0.000483	0.0000028	-5.73	HEXIM2	hexamethylene bis-acetamide inducible 2	Downregulated
A_22_P000	0.000537	0.0000139	-5.74	lnc-RP11-712	lnc-RP11-712L6.5.1-1:1	Downregulated
A_21_P000	0.000483	3.11E-06	-5.8	lnc-BOD1-1	lnc-BOD1-1:3	Downregulated
A_33_P342	0.000563	0.0000167	-5.8	LOC1001292	uncharacterized LOC100129275	Downregulated
A_23_P200	0.000483	7.38E-07	-5.82	AGL	amylo-alpha-1, 6-glucosidase, 4-alpha-glucanotransferase	Downregulated
A_23_P348	0.000483	4.17E-06	-5.83	LETM2	leucine zipper-EF-hand containing transmembrane protein 2	Downregulated
A_32_P198	0.000526	0.0000124	-5.83	NEURL1B	neuralized E3 ubiquitin protein ligase 1B	Downregulated
A_24_P108	0.000792	0.0000464	-5.88	GRAP2	GRB2-related adaptor protein 2	Downregulated
A_23_P256	0.000483	3.66E-06	-5.89	KIF20A	kinesin family member 20A	Downregulated
A_23_P163	0.000483	2.56E-06	-5.91	SCAPER	S-phase cyclin A-associated protein in the ER	Downregulated
A_22_P000	0.000483	3.94E-06	-5.92	lnc-SFN-1	lnc-SFN-1:1	Downregulated
A_23_P393	0.000525	0.0000111	-5.93	KLHL26	kelch-like family member 26	Downregulated
A_23_P979	0.000578	0.0000193	-5.94	MSRB2	methionine sulfoxide reductase B2	Downregulated
A_23_P215	0.000483	3.81E-06	-5.96	VPS41	vacuolar protein sorting 41 homolog (S. cerevisiae)	Downregulated
A_23_P138	0.00055	0.0000151	-6	ATN1	atrophin 1	Downregulated
A_32_P363	0.001243	0.000128	-6	VTI1A	vesicle transport through interaction with t-SNAREs 1A	Downregulated
A_22_P000	0.000483	3.21E-06	-6.01	LOC1005076	uncharacterized LOC100507616	Downregulated
A_23_P317	0.000483	7.23E-07	-6.04	BLK	BLK proto-oncogene, Src family tyrosine kinase	Downregulated
A_23_P735	0.000483	5.85E-06	-6.06	CCDC120	coiled-coil domain containing 120	Downregulated
A_33_P339	0.000483	0.0000014	-6.1	ZNF562	zinc finger protein 562	Downregulated
A_23_P422	0.000483	2.27E-06	-6.1	APOM	apolipoprotein M	Downregulated
A_23_P206	0.000483	2.42E-06	-6.11	KIAA0513	KIAA0513	Downregulated
A_23_P142	0.000483	3.12E-06	-6.11	ARHGAP25	Rho GTPase activating protein 25	Downregulated
A_33_P330	0.000483	3.51E-06	-6.11	RNF207	ring finger protein 207	Downregulated
A_23_P359	0.000483	3.55E-06	-6.11	KDM7A	lysine (K)-specific demethylase 7A	Downregulated
A_23_P170	0.000518	0.0000101	-6.12	SMYD2	SET and MYND domain containing 2	Downregulated
A_33_P326	0.000483	2.61E-06	-6.21	HS2ST1	heparan sulfate 2-O-sulfotransferase 1	Downregulated
A_23_P105	0.000483	2.86E-06	-6.21	BIVM	basic, immunoglobulin-like variable motif containing	Downregulated
A_33_P322	0.000483	0.0000045	-6.27	OAS2	2'-5'-oligoadenylate synthetase 2, 69/71kDa	Downregulated

A_23_P133	0.000483	2.79E-06	-6.31	HUS1B	HUS1 checkpoint homolog b (S. pombe)	Downregulated
A_24_P406	0.000483	2.08E-06	-6.33	FAM53B	family with sequence similarity 53, member B	Downregulated
A_23_P468	0.000486	6.98E-06	-6.34	SLC29A3	solute carrier family 29 (equilibrative nucleoside transporter), member 3	Downregulated
A_33_P329	0.000549	0.0000149	-6.35	TSC22D1	TSC22 domain family, member 1	Downregulated
A_24_P382	0.000483	1.49E-06	-6.36	NHP2L1	NHP2 non-histone chromosome protein 2-like 1 (S. cerevisiae)	Downregulated
A_23_P489	0.000483	1.67E-06	-6.36	SMAD3	SMAD family member 3	Downregulated
A_33_P322	0.000483	2.93E-06	-6.36	GSDMB	gasdermin B	Downregulated
A_23_P109	0.000483	2.67E-06	-6.37	NR1I2	nuclear receptor subfamily 1, group I, member 2	Downregulated
A_33_P341	0.000483	0.0000021	-6.39	LOC1001286	uncharacterized LOC100128607	Downregulated
A_23_P352	0.000483	1.98E-06	-6.43	NEK2	NIMA-related kinase 2	Downregulated
A_33_P332	0.000483	1.36E-06	-6.49	ATP2B4	ATPase, Ca++ transporting, plasma membrane 4	Downregulated
A_24_P409	0.000501	0.0000082	-6.52	PPAPDC2	phosphatidic acid phosphatase type 2 domain containing 2	Downregulated
A_33_P332	0.000483	1.74E-06	-6.56	SAMD12	sterile alpha motif domain containing 12	Downregulated
A_23_P552	0.000483	2.31E-06	-6.61	CCL18	chemokine (C-C motif) ligand 18 (pulmonary and activation-regulated)	Downregulated
A_23_P250	0.000998	0.0000798	-6.62	PARVG	parvin, gamma	Downregulated
A_23_P328	0.000483	2.17E-06	-6.66	FHDC1	FH2 domain containing 1	Downregulated
A_21_P000	0.000483	2.35E-06	-6.66	LYRM4	LYR motif containing 4	Downregulated
A_23_P224	0.000483	1.53E-06	-6.9	GNL3L	guanine nucleotide binding protein-like 3 (nucleolar)-like	Downregulated
A_23_P322	0.000483	3.46E-07	-6.98	TET1	tet methylcytosine dioxygenase 1	Downregulated
A_23_P253	0.000483	1.93E-06	-7.05	ASCL1	achaete-scute family bHLH transcription factor 1	Downregulated
A_23_P164	0.000483	3.32E-07	-7.12	MINK1	misshapen-like kinase 1	Downregulated
A_21_P000	0.000483	0.0000029	-7.17	PDCD6	programmed cell death 6	Downregulated
A_32_P126	0.000483	1.19E-06	-7.21	SLC38A9	solute carrier family 38, member 9	Downregulated
A_33_P330	0.000483	0.0000021	-7.28	PSMD5	proteasome (prosome, macropain) 26S subunit, non-ATPase, 5	Downregulated
A_24_P900	0.000912	0.0000634	-7.3	ADD3	adducin 3 (gamma)	Downregulated
A_22_P000	0.000691	0.0000306	-7.35	lnc-FBXO31-1	lnc-FBXO31-1:1	Downregulated
A_23_P145	0.000483	0.0000015	-7.45	ADRB2	adrenoceptor beta 2, surface	Downregulated
A_32_P226	0.000483	7.78E-07	-7.69	LOC1001297	uncharacterized LOC100129781	Downregulated

Supplementary Table 2. Honokiol (HNK)-mediated proteins (HMPs)
, as retrieved from Swiss target prediction, STITCH, canSAR Black, and SEA.

SwissTargetPrediction	
Gene Name	Gene Symbol
Arachidonate 5-lipoxygenase	ALOX5
Cannabinoid receptor 1	CNR1
Cannabinoid receptor 2	CNR2
Carbonic anhydrase II	CA2
Calcium-activated potassium channel subunit alpha-1	KCNMA1
Monoamine oxidase B	MAOB
Vanilloid receptor	TRPV1
Pyruvate dehydrogenase kinase isoform 1	PDK1
Catechol O-methyltransferase	COMT
Histone deacetylase 8	HDAC8
Cholesteryl ester transfer protein	CETP
Heat shock protein HSP 90-alpha	HSP90AA1
Quinone reductase 2	NQO2
Glucocorticoid receptor	NR3C1
Phospholipase A2 group IIA	PLA2G2A
Receptor protein-tyrosine kinase erbB-2	ERBB2
Epidermal growth factor receptor erbB1	EGFR
Heat shock protein HSP 90-beta	HSP90AB1
Steryl-sulfatase	STS
GABA-A receptor; GABA-A site (alpha1/beta2 interface)	GABRA1
Poly [ADP-ribose] polymerase-1	PARP1
Cytochrome b-c1 complex subunit 7	UQCRB
Mu opioid receptor	OPRM1
Delta opioid receptor	OPRD1
Arachidonate 15-lipoxygenase	ALOX15
Epoxide hydratase	EPHX2
Histone deacetylase 6	HDAC6
Macrophage migration inhibitory factor	MIF
MAP kinase p38 alpha	MAPK14
Anandamide amidohydrolase	FAAH
Acyl coenzyme A:cholesterol acyltransferase	CES1
Dual-specificity tyrosine-phosphorylation regulated kinase 1A	DYRK1A
Kinesin-like protein 1	KIF11
Glycogen synthase kinase-3 beta	GSK3B
Glycogen synthase kinase-3 alpha	GSK3A
Transthyretin	TTR
Carbonic anhydrase I	CA1
Carbonic anhydrase IX	CA9
Serine/threonine-protein kinase PAK 1	PAK1
5-lipoxygenase activating protein	ALOX5AP
Nitric-oxide synthase, brain	NOS1

Dual specificity phosphatase Cdc25B	CDC25B
Adenosine A2a receptor	ADORA2A
Serine/threonine-protein kinase Aurora-B	AURKB
Tyrosine-protein kinase SRC	SRC
Focal adhesion kinase 1	PTK2
Vascular endothelial growth factor receptor 2	KDR
Hepatocyte growth factor receptor	MET
ALK tyrosine kinase receptor	ALK
Serine/threonine-protein kinase NEK6	NEK6
Heat shock protein 75 kDa, mitochondrial	TRAP1
NADPH oxidase 4	NOX4
NADPH oxidase 1	NOX1
Glutamate NMDA receptor; GRIN1/GRIN2B	GRIN1 GRIN2B
Bile acid receptor FXR	NR1H4
Matrix metalloproteinase 9	MMP9
Carbonic anhydrase XII	CA12
Matrix metalloproteinase 1	MMP1
Matrix metalloproteinase 2	MMP2
Protein kinase C gamma (by homology)	PRKCG
Estradiol 17-beta-dehydrogenase 1	HSD17B1
Serine/threonine-protein kinase B-raf	BRAF
Prostanoid EP4 receptor	PTGER4
Monoglyceride lipase	MGLL
11-beta-hydroxysteroid dehydrogenase 1	HSD11B1
Cyclin-dependent kinase 2/cyclin E1	CCNE1 CDK2
Tyrosine-protein kinase receptor FLT3	FLT3
Phosphodiesterase 4B	PDE4B
Nischarin	NISCH
NAD-dependent deacetylase sirtuin 2	SIRT2
G-protein coupled bile acid receptor 1	GPBAR1
Cytochrome P450 11B1	CYP11B1
Cytochrome P450 19A1	CYP19A1
Acetylcholinesterase	ACHE
Cytochrome P450 11B2	CYP11B2
Testis-specific androgen-binding protein	SHBG
Dual specificity tyrosine-phosphorylation-regulated kinase 1B	DYRK1B
Kinesin-1 heavy chain/ Tyrosine-protein kinase receptor RET	RET
Progesterone receptor	PGR
Histone deacetylase 1	HDAC1
Fibroblast growth factor receptor 1	FGFR1
Macrophage colony stimulating factor receptor	CSF1R
Serine/threonine-protein kinase PIM1	PIM1
3-phosphoinositide dependent protein kinase-1	PDPK1
Cyclin-dependent kinase 2	CDK2
Prostaglandin E synthase	PTGES
Potassium-transporting ATPase	ATP4B ATP4A
Alpha-2a adrenergic receptor	ADRA2A
Uridine phosphorylase 1 (by homology)	UPP1

Estradiol 17-beta-dehydrogenase 2	HSD17B2
Vascular endothelial growth factor receptor 1	FLT1
Platelet-derived growth factor receptor beta	PDGFRB
Stem cell growth factor receptor	KIT
Serine/threonine-protein kinase Sgk1	SGK1
Dual specificty protein kinase CLK1	CLK1
CDC7/DBF4 (Cell division cycle 7-related protein kinase/Activa	CDC7
Serine/threonine-protein kinase RAF	RAF1
G-protein coupled receptor 84	GPR84
Tyrosinase	TYR
Protein kinase C (PKC)	PRKCZ

STITCH	
Peptidylprolyl Isomerase F	PPIF
Calpain 11	CAPN11
ATP Binding Cassette Subfamily A	ABCA
Calpain 1	CAPN1
Calpain 3	CAPN3
Calpain 9	CAPN9
BEN Domain Containing 3	BEND3
Nitric Oxide Synthase 3	NOS3
Vascular Endothelial Growth Factor A	VEGFA
Interleukin 4	IL4
Caspase 3	CASP3
Nitric Oxide Synthase 2	NOS2
Cytochrome P450 2B6	CYP2B6
Calpain 2	CAPN2
Caspase 9	CASP9
Caspase 8	CASP8
CASP8 And FADD Like Apoptosis Regulator	CFLAR
Cyclin D1	CCND1

CANSAR BLACK	
Retinoid X Receptor Alpha	RXRA
Gamma-Aminobutyric Acid Type A Receptor Subunit Beta2	GABRB2
Gamma-Aminobutyric Acid Type A Receptor Subunit Alpha5	GABRA5
Gamma-Aminobutyric Acid Type A Receptor Subunit Beta1	GABRB1
Gamma-Aminobutyric Acid Type A Receptor Subunit Alpha2	GABRA2
Gamma-Aminobutyric Acid Type A Receptor Subunit Alpha3	GABRA3
Gamma-Aminobutyric Acid Type A Receptor Subunit Beta3	GABRB3
Gamma-Aminobutyric Acid Type A Receptor Subunit Gamma	GABRG2

SEA.BKSLAB.ORG	
Prostaglandin-Endoperoxide Synthase 2	PTGS2
Prostaglandin-Endoperoxide Synthase 1	PTGS1

Supplementary Table 3. KEGG pathway enrichment analysis of OGs from both HMP and DEGs.

Term	Count	%	PValue	Genes
hsa05200:Pathways in cancer	9	33.3333333	1.11E-05	PTGER4, CASP9, CSF1R, HSP90AA1, RXRA, HSP90AB1, CCND1, HDAC1, VEGFA
hsa04151:PI3K-Akt signaling pathway	8	29.6296296	4.74E-05	CASP9, IL4, CSF1R, HSP90AA1, RXRA, HSP90AB1, CCND1, VEGFA
hsa05215:Prostate cancer	4	14.8148148	0.00227757	CASP9, HSP90AA1, HSP90AB1, CCND1
hsa04919:Thyroid hormone signaling pathway	4	14.8148148	0.00486019	CASP9, RXRA, CCND1, HDAC1
hsa05223:Non-small cell lung cancer	3	11.1111111	0.01237913	CASP9, RXRA, CCND1
hsa04141:Protein processing in endoplasmic re	4	14.8148148	0.01399389	HSP90AA1, HSP90AB1, CAPN2, CAPN1
hsa04210:Apoptosis	3	11.1111111	0.01503458	CASP9, CAPN2, CAPN1
hsa05212:Pancreatic cancer	3	11.1111111	0.01644674	CASP9, CCND1, VEGFA
hsa04510:Focal adhesion	4	14.8148148	0.02365066	PAK1, CCND1, CAPN2, VEGFA
hsa05222:Small cell lung cancer	3	11.1111111	0.02721576	CASP9, RXRA, CCND1
hsa04723:Retrograde endocannabinoid signalir	3	11.1111111	0.03739244	FAAH, CNR1, MGLL

Supplementary Fig. 1

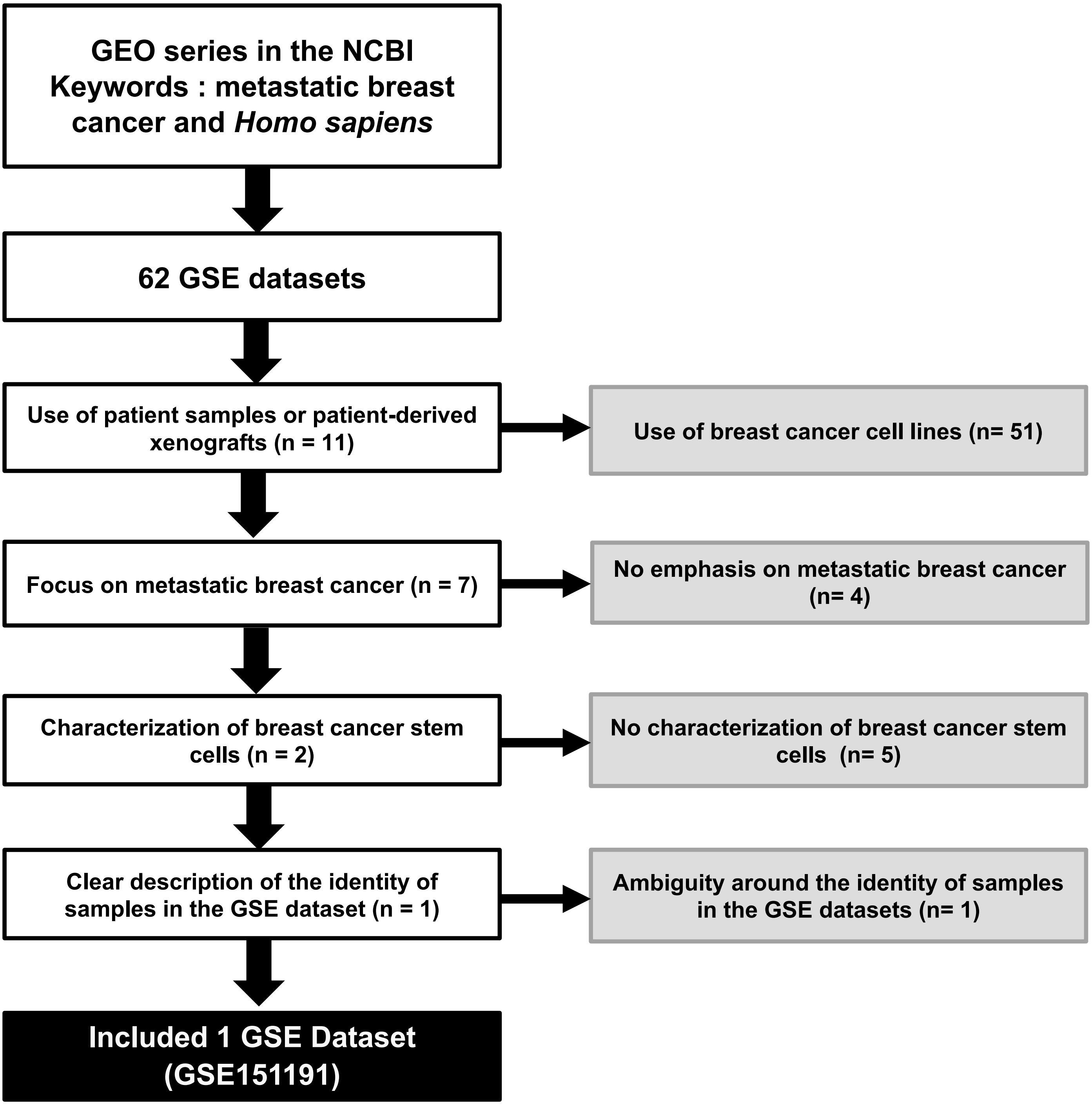

Supplement: Supplementary Materials — Supplementary Table 1. Differentially expressed genes (DEGs) in metastatic breast cancer stem cells (mBCSCs) from the GSE 151191 dataset. Supplementary Table 2. Honokiol (HNK)-mediated proteins (HMPs), as retrieved from Swiss target prediction, STITCH, canSAR Black, and SEA. Supplementary Table 3. KEGG pathway enrichment analysis of OGs from both HMP and DEGs. Supplementary Figure 1. Flowchart for the screening of datasets. [file 4172531.f1.pdf]
